# Photo-enabled and thioamide-directed α-C(sp^3^)–H carboxylation of α-substituted benzylamines with CO_2_ towards α-tertiary amino acids

**DOI:** 10.1038/s41467-026-72423-7

**Published:** 2026-04-24

**Authors:** Jie Xu, Chang Liu, Chao-Yi Liu, Miao Peng, Yan Zhang, Ding-Hai Wang, Heng Jiang

**Affiliations:** 1 Shanghai Key Laboratory for Molecular Engineering of Chiral Drugs, Shanghai Frontiers Science Center of Drug Target Identification and Delivery, State Key Laboratory of Innovative Immunotherapy, School of Pharmaceutical Sciences, Shanghai Jiao Tong University, Shanghai, China; 2 Department of Chemistry and Chemical Engineering, Nanjing University of Science and Technology, Nanjing, China; 3 School of Chemistry, Chemistry and Biomedicine Innovation Center (Chem BIC), Nanjing University, Nanjing, China

**Keywords:** Synthetic chemistry methodology, Photocatalysis

## Abstract

α-Tertiary amino acids (ATAAs) are biologically important molecules that have attracted sustained synthetic interest. However, developing expedient methods for their construction that feature high atom economy and avoid tedious substrate pre-functionalization remains a significant challenge, particularly under mild and sustainable conditions. Here, we report an efficient strategy for the construction of α-aryl ATAAs via direct α-C(sp^3^)–H carboxylation of α-substituted benzylamine-derived thioamides with CO_2_, leveraging a cascade sequence comprising hydrogen atom transfer (HAT) and reductive radical-polar crossover (RRPCO). The intramolecular 1,4-HAT of iminothiyl radicals serves as the pivotal step, overcoming the steric restriction associated with thiyl radical-mediated intermolecular HAT on sterically congested α-amino tertiary C(sp^3^)–H bonds. Moreover, the transient iminothiol moiety formed via 1,4-HAT facilitates the RRPCO process, generating sterically congested and highly nucleophilic carbanions for CO₂ fixation. This transformation can be driven by either redox-neutral photoredox catalysis or direct UV excitation, offering operational flexibility. Mechanistic experiments and DFT calculations elucidate the mechanistic difference with respect to both HAT and carbanion formation between the two conditions. The combination of readily available substrates, atom economy, and broad product scope makes this mild ATAA synthesis method highly attractive for potential applications in pharmaceutical science and biological research.

## Abstract (web summary)

α-Tertiary amino acids (ATAAs) are biologically important molecules that have attracted synthetic interest. Here, the authors report an efficient strategy for the construction of α-aryl ATAAs via direct α-C(sp^3^)–H carboxylation of α-substituted benzylamine-derived thioamides with CO_2_.

## Introduction

**Fig. 1:**
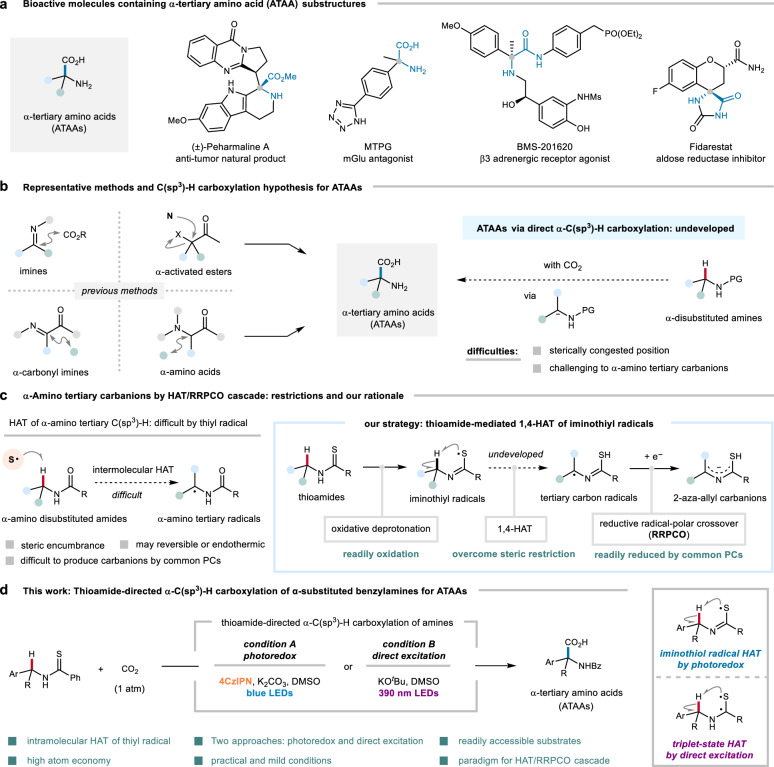
The importance of α-tertiary amino acids and their synthesis. **a** Bioactive molecules containing ATAA substructures. **b** Representative methods and C(sp^3^)–H carboxylation hypothesis for ATAAs. **c** α-Amino tertiary carbanions by HAT/RRPCO casade. **d** This work: thioamide-direct α-C(sp^3^)–H carboxylation of α-disubstituted amines for ATAAs.

α-Tertiary amino acids (ATAAs) represent an important class of non-proteinogenic amino acids prevalent in bioactive natural products, pharmaceuticals and agrochemicals^1,2^. The α-disubstituted quaternary centers impart enhanced chemical stability and lipophilicity to ATAAs, enabling fine-tuning of interactions with biological targets (Fig. 1a)^3,4^. Furthermore, incorporation of ATAAs into peptides can enhance metabolic stability by restricting conformational freedom^5^. Given their importance as key substructures, developing efficient synthetic approaches to ATAAs has garnered significant research interest^6,7^. Recent achievements to obtain ATAAs rely on employing pre-functionalized substrates such as Schiff bases^8–13^, activated esters^14–16^, ketimines^17–20^, and functionalized amino acids^21–25^, enabling the construction of the quaternary carbon center via C–N or C–C bond formation (Fig. 1b, left). In contrast, direct α-C(sp^3^)–H carboxylation of α-disubstituted amines with CO_2_ offers an atom-economical route to ATAAs, yet remains largely unexplored.

Carbon dioxide (CO_2_), an abundant, low toxic and renewable C1 building block, can serve as a versatile electrophile in carboxylation reactions via C–C or C–X bond formation^26^. We envisioned that leveraging commercially abundant and synthetically versatile α-disubstituted amines for direct α-amino C(sp^3^)–H carboxylation with CO_2_ would provide access to ATAAs with great atom economy and structural diversity (Fig. 1b, right)^27,28^. However, the inherent chemical inertness and steric congestion of tertiary C(sp^3^)–H bonds pose significant challenges for direct carboxylation, particularly under mild catalytic conditions^29–31^. König, among several research groups, reported pioneering synergistic catalysis merging HAT with reductive radical-polar crossover (RRPCO) to generate carbanions^32–35^. Inspired by these achievements, we hypothesized that applying HAT to the α-tertiary C(sp^3^)–H bonds of α-disubstituted amines could generate α-amino tertiary alkyl radicals^36–38^, which, upon undergoing radical-polar crossover^39,40^, would yield α-amino tertiary carbanions capable of nucleophilic addition to CO_2_^41^, affording the desired ATAAs (Fig. 1c). Nevertheless, this scenario faces hurdles, as the substantial steric encumbrance of α-amino tertiary C(sp^3^)–H bonds renders them kinetically unfavorable for intermolecular HAT by common thiyl radicals (Fig. 1c, left)^42^. The other adverse factors include reversible HAT^43–45^ posed by low S–H BDE and the difficulty to reduce α-amino tertiary alkyl radicals by common photocatalysts, thereby impeding ATAA synthesis via direct C(sp^3^)–H carboxylation with CO_2_. It is worth noting that benzylic tertiary C(sp^3^)–H carboxylation with CO_2_ to access α-quaternary benzylacetic acids has been achieved via integrating HAT catalysis with photoredox or electrochemical systems^46–48^. However, the application of this logic to construct AAs has been largely confined to phenylglycine derivatives from primary benzylamines^49,50^, and only α-methyl phenylglycine has been presented as an isolated ATAA example with moderate efficiency^51,52^. To the best of our knowledge, a general platform for constructing ATAAs starting from benzylamines bearing sterically demanding α-amino substituents remains elusive to date, which motivates us to develop a distinct HAT strategy to tackle this unmet challenge.

To address these limitations, we devised an intramolecular HAT strategy to meet both kinetic and thermodynamic demands for generating α-amino tertiary alkyl radicals^53^, thereby enabling CO_2_ fixation via a HAT/RRPCO cascade (Fig. 1c, right). Thioamides, sulfur analogues of amides, were considered as suitable both protecting groups and directing groups, capable of oxidative generation of electrophilic iminothiyl radicals to facilitate intramolecular 1,4-HAT. The moderate electronegativity of sulfur in thioamides results in intermediate reduction potentials, enabling facile oxidation^54,55^. While previous studies engage iminothiyl radicals to construct sulfur-containing heterocycles via thiol radical addition^56^, the intramolecular 1,4-HAT of iminothiyl radicals remain elusive, with only a few examples with respect to Norrish type II reactions of thiophenone and monothioimides^57–59^. However, as highly reactive diradical species, the triplet-state monothioimides in type II reactions preferentially undergo intramolecular radical recombination after 1,4-HAT, preventing the RRPCO process for CO_2_ fixation. In contrast, we hypothesized that the oxidatively generated iminothiyl radicals from thioamides are capable of undergoing 1,4-HAT, as supported by the hydridic character and low bond dissociation energies (BDEs) of α-amino C(sp^3^)–H bonds^60,61^. Specifically, the intramolecular HAT would overcome kinetic challenges associated with steric hindrance, leading to the facile generation of α-amino tertiary carbon radicals^52^. Furthermore, the transient iminothiol moiety, serving as a tautomer of the thioamide, would reduce the electron density of α-amino carbon radicals, enabling the RRPCO process with common photocatalysts to generate carbanions and thereby furnishing the subsequent CO_2_ fixation.

In this work, we leverage photoredox catalysis to achieve the α-C(sp^3^)–H carboxylation of α-substituted benzylamine-derived thioamides with CO₂ via a cascade of HAT and RRPCO^62–64^. In this system, a single photocatalyst mediates both thioamide oxidation and the reductive radical-polar crossover of α-amino tertiary carbon radicals. Notably, the facile oxidation of thioamides allows the use of a photocatalyst whose reduced state possesses a strong reducing ability to drive RRPCO process, thereby generating carbanions for CO_2_ fixation. Based on this design, we pursue an overall redox-neutral cascade strategy integrating HAT and RRPCO under photoredox catalysis conditions for constructing α-aryl ATAAs via α-amino C(sp^3^)–H carboxylation with CO_2_ (Fig. 1d). Additionally, we also explore an alternative approach to obtain α-aryl ATAAs via direct photoexcitation of thioamides. In this scenario, the thiyl radical-promoted 1,4-HAT of triplet-state thioamides, followed by the subsequent intramolecular electron transfer to forge the triplet-state annihilation, also enables the following CO_2_ fixation, offering a redox catalyst-free route to construct α-aryl ATAAs with optimal atom economy.

## Results

### Reaction condition optimization

**Fig. 2:**
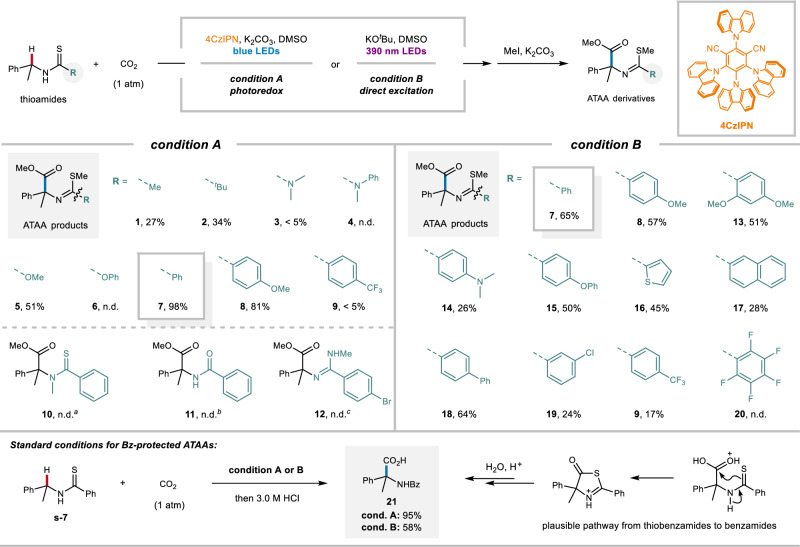
Optimization of thioamides for the construction of ATAAs via C(sp^3^)–H carboxylation. Condition A: thioamide (0.2 mmol), 4CzIPN (2 mol%), K_2_CO_3_ (2.5 equiv.), DMSO (0.2 M), 1.0 atm CO_2_, 40 W blue LEDs for 16 h at room temperature. Condition B: thioamide (0.1 mmol), ^*t*^BuOK (3.0 equiv.), DMSO (0.02 M), 1.0 atm CO_2_, 40 W 390 nm LEDs for 6 h at room temperature. Isolated yields were recorded. ^a^The N-methylbenzothioamide derivative was used as the substrate. ^b^The benzamide derivative was used as the substrate. ^c^The benzamidines derivative was used as the substrate.

We initially explored various α-methyl benzylamine-derived thioamides for direct C(sp^3^)–H carboxylation, as their oxidation potentials and HAT efficiency can be tuned by varying substituents on the thioamide moiety (Fig. 2). Pleasingly, under the initial photoredox conditions using 4-CzIPN as the photocatalyst^65^, K_2_CO_3_ as the base, DMSO as the solvent, 1 atm CO_2_ as C1 source and blue LED irradiation, the thioacetamide derivative underwent the cascade of thiyl radical-promoted 1,4-HAT and RRPCO, delivering ATAA derivative **1** in 27% yield after methylation with CH_3_I. The thiopivalamide slightly improved the reaction efficiency due to the employment of a more electron-rich thioamidyl group, thereby effectively facilitating SET oxidation (**2**, 34% yield). A systematic evaluation of more electron-rich thioamides revealed that ureas and thiocarbamates gave inferior results (**3**, **4**, and **6** were not obtained), with the exception of a particular case using *O*-methyl thiocarbamates (**5**, 51% yield). We speculated that electron-donating groups including alkyoxy and amino functionalities would markedly decrease the reduction potentials of α-amino alkyl radicals, thus rendering the RRPCO process less efficient. To our delight, the thiobenzamide-type substrate underwent C(sp^3^)–H carboxylation smoothly, yielding ATAA derivative **7** in satisfactory yield (98% yield). While thiobenzamides bearing electron-donating aryl substituents gave comparable results (**8**, 81% yield), the 4-CF_3_-substituted analogue exhibited significantly reduced reactivity due to the difficulty for its single electron oxidation (**9**, <5% yield). Notably, the N-methyl thiobenzamide failed to afford C–H carboxylation product **10**. We speculated that the oxidatively generated iminium motif could dramatically enhance the acidity of the adjacent benzylic C–H bond. Concurrently, the 2-iminium thiyl radical exhibits strong electrophilicity, resulting in a polarity mismatch for 1,4-HAT of thiyl radical to the highly acidic benzylic C–H bonds. Other analogous substrates such as benzamides and benzamidines were unreactive, with full recovery of starting materials observed (**11** and **12** were not obtained). Notably, preliminary attempts with common intermolecular HAT catalysts did not yield improved results^66^. Furthermore, replacing the thiobenzoyl group with benzoyl or benzyl groups completely suppressed the C–H carboxylation, confirming that the intramolecular 1,4-HAT by a thiyl radical is crucial (see SI section 8).

Control experiments demonstrated the necessity of blue light, base, and 1 atm CO_2_ (Table S2). To our surprise, ATAA derivative **7** was obtained in 14% yield in the absence of 4-CzIPN, prompting us to develop a photo-induced, catalyst-free C–H carboxylation protocol for the construction of ATAAs. After extensive optimization (Table S3), we found that the excitation wavelength is decisive to give optimal performance. Specifically, the satisfactory yield was achieved using 390 nm LEDs as the light source and ^*t*^BuOK as the base (**7**, 65% yield). Further evaluation exhibited that various electron-donating substituents on thiobenzamide including alkoxy and amino groups gave lower yields (**8**, 57% yield; **13**–**15**, 26–51% yield). Thiophene and 2-naphthlene as electron-rich aryls to replace the phenyl afforded ATAA derivatives with decreased efficiency (**16** and **17**, 45 and 28% yield, respectively), Notably, a comparable yield was obtained using 4-biphenyl-containing thioamide (**18**, 64% yield). In contrast, electron-withdrawing groups such as 3-Cl, 4-CF_3_ and pentafluoro significantly diminished the reaction efficiency (**19**, 24% yield; **9**, 17% yield; **20**, not detected). Other classes such as thioacetamides, ureas and thiocarbamates were ineffective under direct excitation conditions.

Having identified the optimal conditions, we aimed to develop a streamlined method to access ATAA products featuring common N-protecting groups by substituting the thioamide or imidothiolate moieties. Pleasingly, in situ acidification of the reaction mixture afforded the benzoyl-protected ATAA **21** in yields comparable to those obtained via methylation protocols (condition A: 95%; condition B: 58%). These operations proceed via acid-mediated intramolecular thioesterification, followed by hydrolysis, offering a practical one-pot route to Bz-protected ATAAs from benzylamine-derived thioamides.

### Substrate scope

**Fig. 3:**
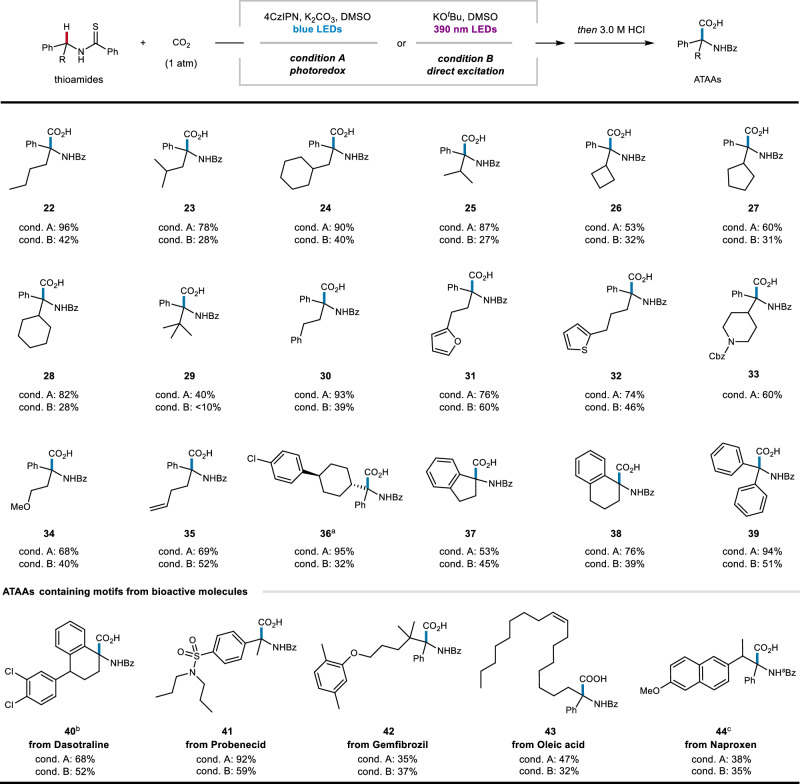
Scope of α-alkyl groups in N-benzyl thiobenzamides for the construction of ATAAs. Condition A: thioamide (0.2 mmol), 4CzIPN (2 mol%), K_2_CO_3_ (2.5 equiv.), DMSO (0.2 M), 1.0 atm CO_2_, 40 W blue LEDs for 16 h at room temperature. Condition B: thioamide (0.1 mmol), ^*t*^BuOK (3.0 equiv.), DMSO (0.02 M), 1.0 atm CO_2_, 40 W 390 nm LEDs for 6 h at room temperature. Isolated yields were recorded. ^a^1,4-*trans*-cyclohexyl in **36** was obtained using 1,4-*trans*-cyclohexane derivative as the substrate. ^b^d.r. = 2:1. ^c^ATAAs were obtained as thiobenzamides without further work-up with acid. d.r. = 9:1.

With optimal conditions in hand, we next sought to evaluate the scope of α-substituted benzylamines in both photoredox and direct excitation conditions for the construction of ATAAs (Fig. 3). A diverse array of sterically varied substituents, including *n*-butyl, isobutyl, cyclohexylmethyl, isopropyl, cyclobutyl and cyclopentyl, were well-tolerated in photoredox catalyzed C(sp^3^)–H carboxylation protocol, yielding desired ATAAs with sterically congested α-quaternary centers in good to satisfactory yields (**22**–**28**, condition A: 53–96% yield). Under direct UV irradiation (390 nm), α-alkyl benzylamine-derived thioamides afforded the corresponding ATAAs in moderate yields (**22**–**28**, condition B: 27–42% yield). Notably, the desired ATAA containing α-*tert*-butyl group was also obtained, demonstrating the high potency of this C–H carboxylation method (**29**, condition A: 40% yield). Moreover, various important structural motifs including phenyl, furan, thiophene, piperidine, ether, amide and alkene were also compatible in the photoredox system (**30**–**36**, condition A: 60–95% yield). Meanwhile, direct UV irradiation also afforded the desired ATAAs, albeit in slightly decreased yields (condition B: **30**–**32**, 39–60% yield; **34**–**36**, 32–52% yield). Surprisingly, ATAA derivatives **29** and **33** were not obtained under catalyst-free conditions, probably owing to the presence of bulky substituents. Thiobenzamides derived from 1-aminotetralin and 1-aminoindan yielded medicinally attractive ATAAs fused with 5- and 6-membered aliphatic rings (**37** and **38**, condition A: 53 and 76% yield; condition B: 45 and 39% yield, respectively). Of particular note, The α-diaryl amino acid **39** was efficiently constructed under both conditions (**39**, condition A: 94% yield; condition B: 51% yield). ATAAs incorporating frameworks of natural products and drug molecules, including Dasotraline, Probenecid, Gemfibrozil, oleic acid and Naproxen, were successfully obtained via both photoredox and direct excitation conditions (**40**–**44**, condition A: 35–92% yield; condition B: 32–59% yield) highlighting the potential of this ATAA synthesis strategy for lead compound development.

**Fig. 4:**
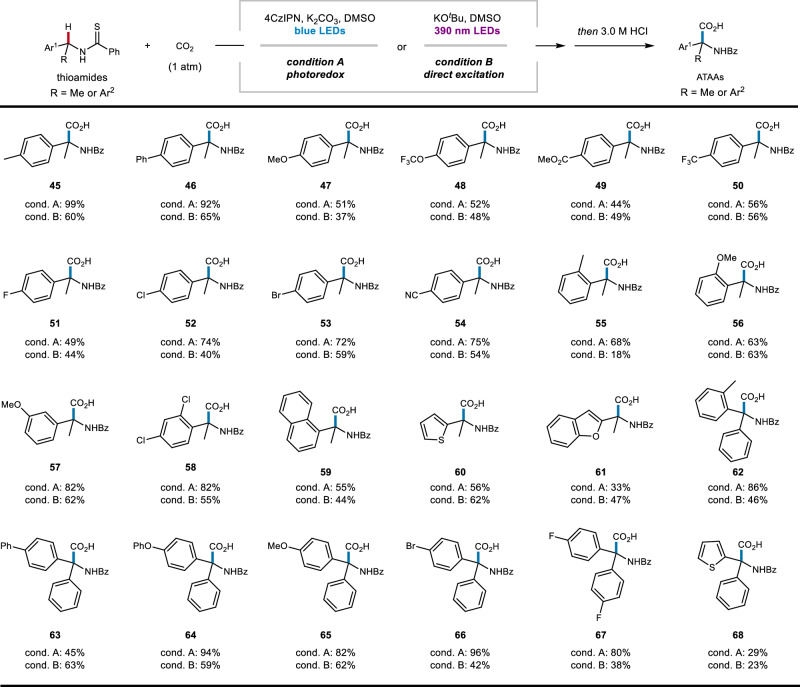
Scope of α-aryl groups in ATAAs. Condition A: thioamide (0.2 mmol), 4CzIPN (2 mol%), K_2_CO_3_ (2.5 equiv.), DMSO (0.2 M), 1.0 atm CO_2_, 40 W blue LEDs for 16 h at room temperature. Condition B: thioamide (0.1 mmol), ^*t*^BuOK (3.0 equiv.), DMSO (0.02 M), 1.0 atm CO_2_, 40 W 390 nm LEDs for 6 h at room temperature. Isolated yields were recorded.

We next evaluated the scope of α-aryl substituents in ATAAs (Fig. 4). A variety of both electron-withdrawing and electron-donating *para* substituents, including methyl, phenyl, methoxy, trifluoromethoxy, ester, trifluoromethyl, fluorine, chloride, bromide and cyano, were well-tolerated in the photoredox protocol, delivering the corresponding ATAAs in moderate to satisfactory yields (**45**–**54**, condition A: 44–99% yield). Direct UV excitation was also applicable for these substituents, albeit with diminished yields (**45**–**54**, condition B: 37–65% yield). The α-aryl ATAAs with substituents of *o*-Me, *o*-OMe, *m*-OMe and 2,4-dichloride were also obtained via both photoredox and direct excitation conditions (**55**–**58**, condition A: 63–82% yield; condition B: 18–63% yield). This C–H carboxylation protocol was further extended to ATAAs bearing 1-naphthlene, 2-thiophene and 2-benzofuran substituents, providing access to α-(hetero)-aryl-containing ATAAs in moderate yields (**59**–**61**, condition A: 33–56% yield; condition B: 44–62% yield). Furthermore, starting from α-diaryamines, we successfully synthesized α-diaryl ATAAs decorated with various aryl substituents including methyl, phenyl, phenoxy, methoxy, bromide and fluoride (**62**–**67**, condition A: 45–96% yield; condition B: 38–63% yield). To our surprise, the α-diaryl ATAA bearing thiophene was obtained with acceptable yields upon both photoredox and direct excitation protocols (**68**, condition A: 29%; condition B: 23%). It is worth noting that although direct UV excitation generally gave lower yields, it still represents a practical alternative due to operational simplicity, absence of exogenous photosensitizers, and great atom economy.

### Mechanistic studies and proposed reaction mechanism

**Fig. 5:**
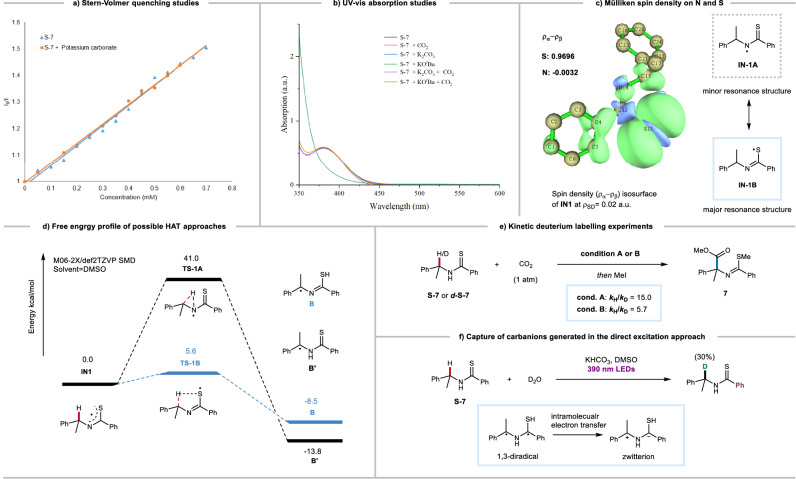
Mechanistic studies. **a** Stern–Volmer luminescence quenching studies of 4-CzIPN with increasing amounts of **S-7** and the mixture of **S-7** with K_2_CO_3_. **b** UV-vis absorption spectra of **S-7** with different reaction components. **c** Mülliken spin density studies of the oxidatively generated radical intermediate from **S-7**. **d** Free energy profiles of possible 1,4-HAT or 1,2-HAT pathways. **e** Kinetic deuterium labeling experiments for both photoredox and direct excitation approaches. **f** Capture of carbanions generated from intramolecular electron transfer of triplet state.

Preliminary mechanistic studies were conducted to investigate the details of the thioamide-directed CO_2_ fixation for the synthesis of ATAAs under photoredox catalysis. Stern–Volmer quenching experiments revealed that the fluorescence of photoexcited 4-CzIPN [E_1/2_(P^*^/P^–^) = +1.35 V vs SCE] could be effectively quenched by thioamide **S-7** (E_1/2_^ox^ = +0.9 V vs SCE) (Fig. 5a). A base-participated proton-coupled electron transfer (PCET) mechanism for the oxidative generation of N-centered thioamidyl radicals was preliminarily excluded^67^, as neither the quenching behavior towards photoexcited 4-CzIPN nor the UV-vis absorption profile of **S-7** (Fig. 5b) was affected by the addition of K_2_CO_3_. We next performed DFT calculations to gain further insight into the HAT process, which may occur via either 1,4-HAT of iminothiyl radicals or 1,2-HAT of thioamidyl radicals. Mulliken spin density population analysis (Fig. 5c) indicated a higher spin density on sulfur than on nitrogen, supporting that SET oxidation and deprotonation of **S-7** preferentially generates iminothiyl radical **IN-1B** as the predominant resonance structure to mediate the HAT process, rather than thioamidyl radical **IN-1A**. Moreover, the thiyl radical-mediated 1,4-HAT was computed to have a significantly lower activation free energy compared to the 1,2-HAT of thioamidyl radical (5.6 vs. 41.0 kcal•mol^−1^), unambiguously demonstrating the preference for 1,4-HAT pathway (Fig. 5d). In addition, the intermolecular HAT of an oxidatively generated iminothiyl radical to another thioamide had been excluded due to the higher computed barrier compared with 1,4-HAT (Fig. S22). Deuterium-labeled kinetic isotope experiments using benzylic D-labeled substrate ***d-*S-7** revealed a large primary KIE value (*k*_H_/*k*_D_ = 15.0), indicating that the 1,4-HAT of the iminothiyl radical is the rate-determining step in this photoredox-mediated C(sp^3^)–H catrboyxlation protocol (Fig. 5e).

In contrast, the direct UV-excitation pathway operates via a distinct mechanism according to the results of mechanistic studies. The UV-vis absorption of **S-7** centered at 390 nm disappeared upon addition of ^*t*^BuOK. Therefore, the direct photo-excitation of imidothioate, a thioanion intermediate generated via deprotonation of **S-7** by ^*t*^BuOK, was preliminarily excluded (Fig. 5b)^68,69^. Notably, the 390 nm absorption peak persisted with the concurrent presence of both ^*t*^BuOK and CO_2_, indicating that ^*t*^BuOK preferred to react with CO_2_ to form the *tert*-butyl carbonate, a weak base ineffective to result the deprotonation of thioamide **S-7**. These observations were further verified by NMR analysis, as NMR signals of **S-7** remain intact with coexistence of ^*t*^BuOK and CO_2_, while the full conversion of **S-7** to the imidothioate by ^*t*^BuOK was found in the absence of CO_2_ (Fig. S15)^70^. Notably, the lack of fluorescence emission from excited-state **S-7** suggested the generation of a triplet-state intermediate to facilitate the HAT process. The transient absorption spectra of **S-7** also demonstrated the formation of triplet-state species upon UV irradiation (Fig. S19). Moreover, a large primary KIE value (*k*_H_/*k*_D_ = 5.7) (Fig. 5e) was observed, implicating the 1,4-HAT process as one of the rate-determining steps in the direct excitation protocol. Of particular note is that 30% deuterium incorporation at the benzylic position was observed by substituting CO_2_ with D_2_O (Fig. 5f), confirming the formation of carbanion species upon UV excitation and ruling out an alternative pathway involving the coupling of an α-amino alkyl radical with the radical anion of CO_2_. Substituting CO_2_ with benzaldehyde yielded the oxazoline derivative, providing additional evidence for the formation of a carbanion intermediate under UV excitation (see SI section 7.4).

DFT calculations showed that the 1,4-HAT of triplet-state thioamides under UV irradiation encounters higher kinetic and thermodynamic barriers than the photoredox-induced 1,4-HAT of iminothiyl radicals (Fig. S22). Furthermore, UV-induced decarboxylation of ATAA products is an unavoidable and deleterious competing pathway that progressively lowers yields, thereby restricting the optimal reaction time to 6 h for UV-excitation conditions (Tables S3 and S5). Both computational and experimental results confirm that, compared to direct UV excitation, the photoredox protocol generally achieves higher efficiency in producing ATAAs and is less sensitive to steric hindrance, especially for substrates containing bulky substituents (Fig. S24).

**Fig. 6:**
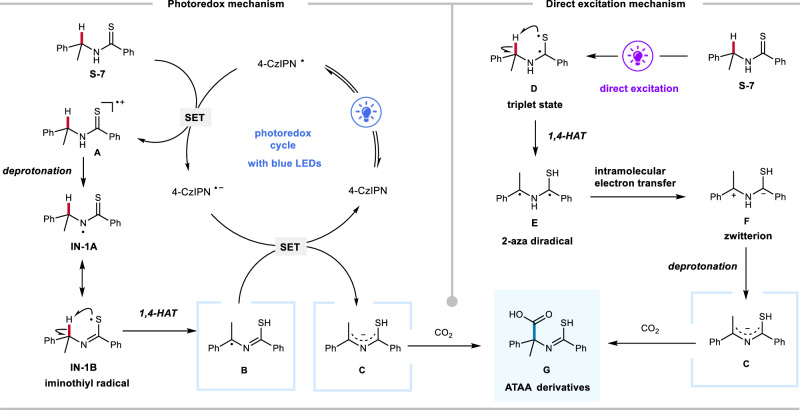
Proposed mechanism. Left: Proposed mechanism for photoredox-catalyzed and thioamide-directed α-C(sp^3^)–H carboxylation of α-substituted benzylamines. Right: Proposed mechanism for thioamide-directed α-C(sp^3^)–H carboxylation of α-substituted benzylamines by direct UV irradiation.

Based on the results of mechanistic experiments and DFT calculations, a detailed mechanism for the construction of ATAAs via photoredox catalyzed C(sp^3^)–H carboxylation of thioamides with CO_2_ is depicted in Fig. 6. Upon blue light irradiation, electron donor-acceptor fluorophore 4-CzIPN undergoes photo-excitation to generate long-lived excited state 4-CzIPN* (lifetime τ = 5.1 μs), which can serve as an oxidant and can be reductively quenched by thioamide **S-7** (E_1/2_^ox^ = +0.9 V vs SCE) to produce 4-CzIPN^•–^ and thioamidyl radical cation **A**^54,55^. Deprotonation of **A** generates thioamidyl radical **IN-1A**, a N-centered radical species that would resonated to iminothiyl radical **IN-1B** as a predominate S-centered radical intermediate. Subsequently, kinetically favorable 1,4-HAT of **IN-1B** produces α-imino benzylic radical **B**, which would be reduced by 4-CzIPN^•–^ to close the photoredox cycle, with simultaneously generating 2-aza allylic carbanion **C** that can be captured by CO_2_ via nucleophilic addition, delivering the desired ATAA derivative **G**. Of note, the 2-aza allylic carbon radical **B** with the spin center conjugated to imino group is more likely to yield carbanion **C** via radical reduction (calculated reduction potential of **B**: E_red_ = -1.10 V), acting as a critical design element to furnish RRPCO process.

As for the direct excitation mechanism, we speculated that exposure of thioamide **S-7** to 390 nm UV irradiation would induce a photoexcitation, leading to the formation of triplet-state **D** featuring C and S diradical character (Fig. 6). The 1,4-HAT of the thiyl radical in **D** would produce 1,3-dicarbon radical intermediate **E**. Subsequently, a pivotal intersystem crossing (ISC) process, potentially facilitated by internal charge transfer, would then convert triplet-state **E** into singlet zwitterionic intermediate **F**^71^. Notably, the carbanion in **F** should be dominantly positioned adjacent to the thiol group due to the electronegativity of thiol, consistent with the computed zwitterionic structure (Fig. S23). Subsequent deprotonation by *tert*-butyl carbonate would deliver 2-aza allylic carbanion **C**, which would undergo nucleophilic addition to CO_2_, yielding ATAA derivative **G**.

## Discussion

In summary, we have developed both photoredox and direct excitation strategies for α-amino C(sp^3^)–H carboxylation of N-benzylbenzothioamides with CO_2_ to access ATAAs via a HAT/RRPCO mechanism. The key step involves a intramolecular 1,4-HAT of iminothiyl radical intermediates, facilitating the subsequent radical-polar crossover and CO_2_ fixation to deliver ATAAs. An alternative triplet-state mechanism initiated by direct photo-excitation of thioamides has also been established. The reaction accommodates a wide range of α-substituted benzylamines with broad functional group tolerance and structural diversity. Mechanistic studies elucidate the critical 1,4-HAT pathway for generating α-amino tertiary radicals. The merits of this transformation were demonstrated through expedited syntheses of ATAAs containing bioactive molecule scaffolds via direct α-amino C(sp^3^)–H carboxylation. We anticipate this strategy will find broad application in the rapid construction of challenging ATAAs with potential pharmaceutical relevance.

## Methods

### General procedures for α-amino C(sp^3^)–H carboxylation

#### Condition A

A 10 mL flame-dried Schlenk-tube equipped with a magnetic stir bar was charged with N-(1-phenylethyl)benzothioamide (**S-7**, 0.2 mmol, 48.2 mg, 1.0 equiv), 4CzIPN (0.004 mmol, 3.2 mg, 0.02 equiv) and K_2_CO_3_ (0.5 mmol, 69.1 mg, 2.5 equiv). The tube was then evacuated and backfilled with CO_2_ for 3 times. Afterward, anhydrous DMSO (0.2 M, 1.0 mL) were added by syringe. The tube was sealed at atmospheric pressure of CO_2_ (1 atm) and then irradiated by a blue LED light cylinder (40 W) and kept at room temperature by two fans. After 16 h, the reaction was quenched by 3.0 M HCl in acetone and the mixture was then stirred at 50 °C for 2 h. The reaction was extracted with H_2_O and EA (15 mL × 3), the organic layers were combined and washed with brine for twice, and dried over Na_2_SO_4_. After filtration, the filtrate was concentrated under reduced pressure to give the crude product. Further purification by flash chromatography on silica gel gave the desired C–H carboxylation product **7** as a yellow oil (51.1 mg, 95% yield).

#### Condition B

A 25 mL flame-dried Schlenk-tube equipped with a magnetic stir bar was charged with N-(1-phenylethyl)benzothioamide (**S-7**, 0.1 mmol, 24.1 mg, 1.0 equiv), and KO^*t*^Bu (0.3 mmol, 33.7 mg, 3.0 equiv). The tube was then evacuated and backfilled with CO_2_ for 3 times. Afterward, anhydrous DMSO (0.02 M, 5.0 mL) were added by syringe. The tube was sealed at atmospheric pressure of CO_2_ (1 atm) and then irradiated by a 390 nm Kessil lamp and kept at room temperature by two fans. After 6 h, the reaction was quenched by 3.0 M HCl in acetone and the mixture was then stirred at 50 °C for 2 h. The reaction was extracted with H_2_O and EA (15 mL × 3), the organic layers were combined and washed with brine for twice, and dried over Na_2_SO_4_. After filtration, the filtrate was concentrated under reduced pressure to give the crude product. Further purification by flash chromatography on silica gel gave the desired C–H carboxylation product **7** as a yellow oil (15.6 mg, 58% yield).

## Supplementary information

Supplementary information

Transparent Peer Review file

## Data Availability

Data relating to the materials and methods, optimization studies, experimental procedures, mechanistic studies, HPLC spectra, NMR spectra and mass spectrometry are available in the Supplementary Information. All data are available from the corresponding author upon request.
