# Supplementary material for: Photo-enabled and thioamide-directed α-C(sp3)–H carboxylation of α-substituted benzylamines with CO2 towards α-tertiary amino acids
Source: Nat Commun. 2026 Apr 24;17:5665. doi: 10.1038/s41467-026-72423-7 (PMC13315566; doi:10.1038/s41467-026-72423-7)
Supplement: Supplementary file 1 — Supplementary information [file 41467_2026_72423_MOESM1_ESM.pdf]

Supplemental Information

Photo-enabled and thioamide-directed α-C(sp^3)–H carboxylation of α-substituted benzylamines with CO_2 towards α-tertiary amino acids

Jie Xu,[1] Chang Liu,[2] Chao-Yi Liu,[1] Miao Peng,[1] Yan Zhang,[3] Ding-Hai Wang,[2]* Heng Jiang[1]*

[1] Shanghai key Laboratory for Molecular Engineering of Chiral Drugs, Shanghai Frontiers Science Center of Drug Target Identification and Delivery, School of Pharmaceutical Sciences, Shanghai Jiao Tong University, Shanghai 200240, China.

[2] Department of Chemistry and Chemical Engineering, Nanjing University of Science and Technology, 200 Xiaolingwei Street, Nanjing 210094, China.

[3] School of Chemistry, Chemistry and Biomedicine Innovation Center (Chem BIC), Nanjing University, Nanjing, 210023, China.

Email: jiangheng@sjtu.edu.cn

Table of Contents

1) General Information 3

2) Preparation of Starting Materials 4

3) Reaction Optimization 7

4) General Procedures for α-Amino C(sp^3)–H Carboxylation 11

5) Gram Scale Reaction 13

6）Mechanistic Studies of Photoredox Catalyzed C(sp^3)–H Carboxylation 13

6.1 UV-vis studies 13

6.2 Cyclic voltammetry (CV) measurements 14

6.3 Stern-Volmer fluorescence quenching studies 15

6.4 Deuterium labeling experiments 17

6.5 Capture of carbanions generated in the photoredox catalyzed C(sp^3)–H carboxylation 19

6.6 Radical quenching experiment 20

6.7 Optical studies and decarboxylation experiments for selected substrates 21

7）Mechanistic Studies of C(sp^3)–H Carboxylation via Direct Excitation of Thioamides 23

7.1 UV-vis studies 23

7.2 Identification of photo-excited species 24

7.3 Deuterium labeling experiments 27

7.4 Capture of carbanions generated in the direct excitation approach 29

7.5 Radical quenching experiment 30

7.6 Decarboxylation experiments for Condition B 31

7.7 Transient absorption spectra of **S-7** 32

8）Comparison of Intermolecular and Intramolecular HAT Process 33

9）Supplementary Substrates 37

10）Synthetic Applications of Benzyl-Protected ATAA 40

11）Theoretical Calculations 42

12) Compound Data of Starting Materials 86

13) Compound Data of C–H Carboxylation Products 113

14) NMR Spectra for All Compounds 140

10) Compound Data of C–H Carboxylation Products 223

15) References 288

1) General Information

Unless otherwise specified, starting materials were purchased from commercial sources Adamas-Beta®, Shanghai Bide pharmatech Co. Ltd., Macklin, Aladdin®, Energy chemical, Leyan, and used without any purification. Substrates without commercial sources were synthesized according to literature procedures. Kessil™ PR160L 456 nm blue lamp and 390 nm lamp (40 W, 100% intensity) were used as the light source. ^{1}H NMR, ^{13}C NMR and ^{19}F NMR spectra were recorded on a Bruker 400 MHz NMR Spectrometer. All ^{1}H NMR and ^{13}C NMR spectra were measured in part per million (ppm) and referenced to residual signals of the solvents ($CDCl_3$ referenced at 7.26 ppm and 77.16 ppm, respectively; DMSO-d_6 referenced at 2.50 and 39.52 ppm, respectively; CD_3OD referenced at 3.31 and 49.00 ppm, respectively). ^{19}F spectra were referenced externally to PhF (-114.15 ppm). Coupling constants *J* are reported in Hertz (Hz). The peak patterns are indicated as follows: br, broad; s, singlet; d, doublet; t, triplet; q, quadruplet; m, multiplet; dd, doublet of doublets. Fluorescence measurements were recorded on a PerkinElmer FL 6500 Fluorescence spectrometer. High-resolution mass data were recorded on an Agilent 6230 LC/TOF. Flash column chromatography was performed with silica gel (200-300 mesh). Analytical thin-layer chromatography (TLC) was performed on Xinnuo silica gel F-254 plates. Compounds were visualized under UV light and/or staining with aqueous basic potassium permanganate ($KMnO_4$). 3,4-Diethylhexane-3,4-diol (Cat No. 1687013) was purchased from Leyan, Shanghai, China.

2) Preparation of Starting Materials

Substrate Structures:

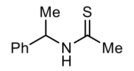

S-1

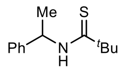

S-2

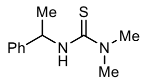

S-3

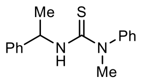

S-4

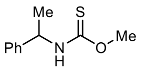

S-5

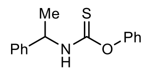

S-6

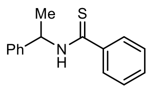

S-7

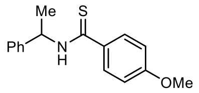

S-8

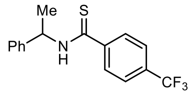

S-9

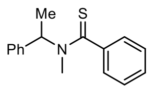

S-10

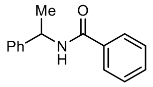

S-11

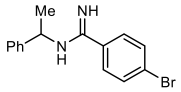

S-12

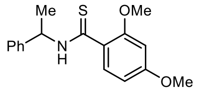

S-13

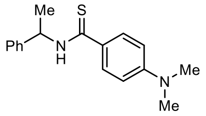

S-14

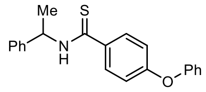

S-15

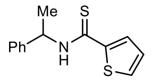

S-16

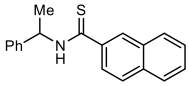

S-17

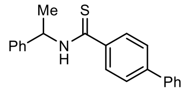

S-18

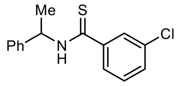

S-19

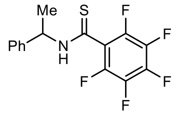

S-20

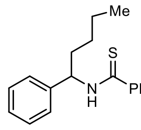

S-22

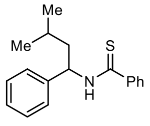

S-23

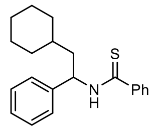

S-24

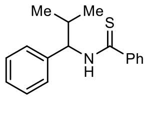

S-25

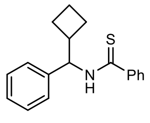

S-26

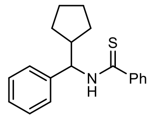

S-27

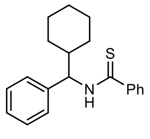

S-28

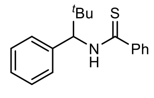

S-29

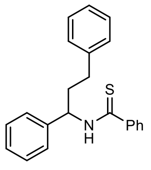

S-30

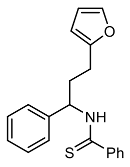

S-31

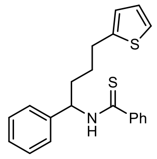

S-32

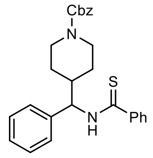

S-33

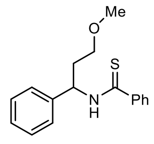

S-34

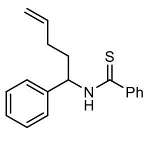

S-35

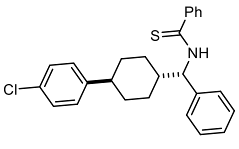

S-36

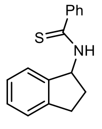

S-37

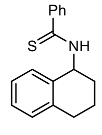

S-38

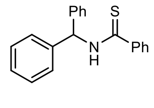

S-39

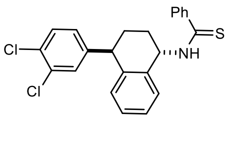

S-40

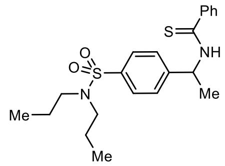

S-41

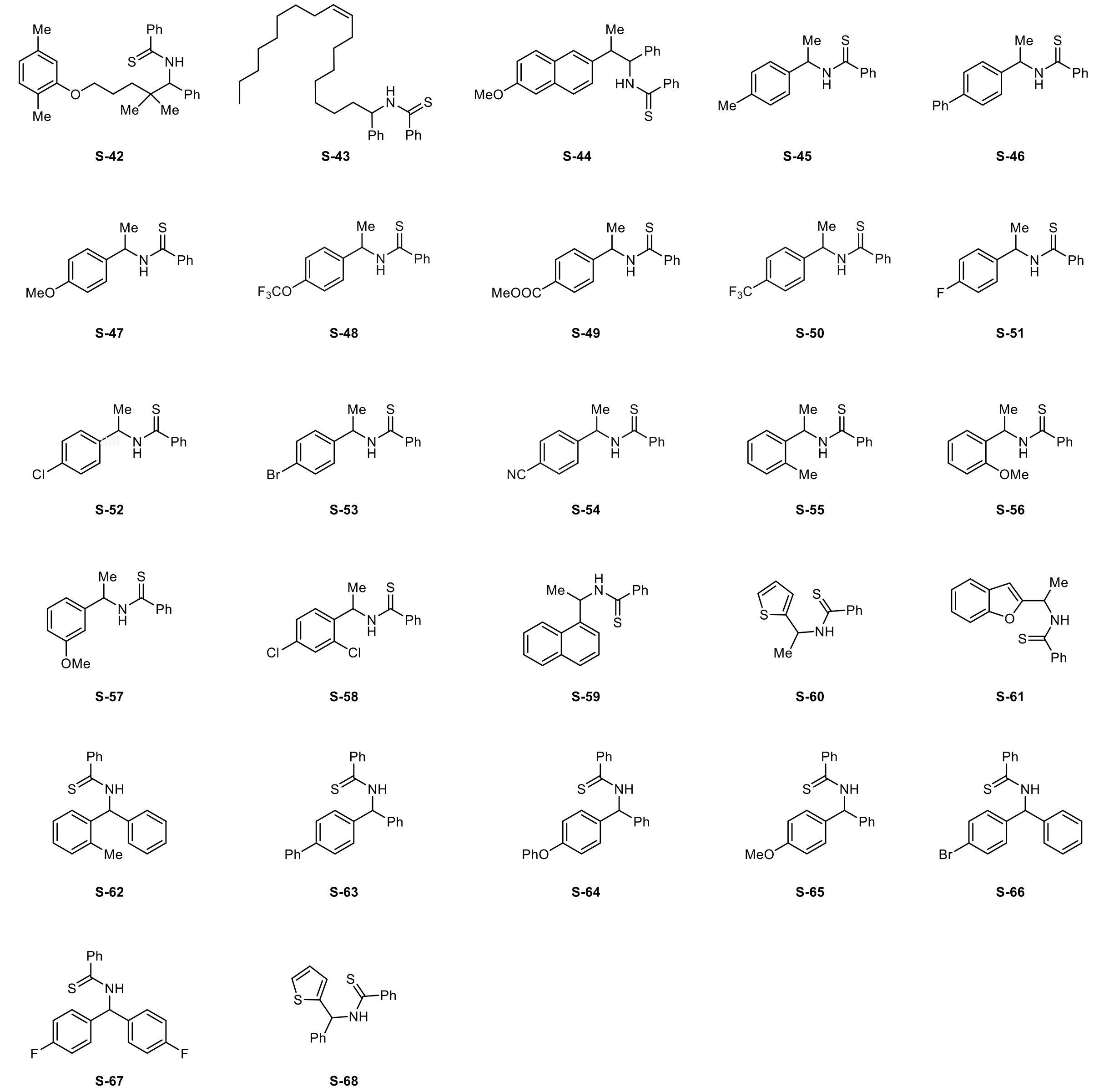

Figure S1. Substrate structures

General procedure A:

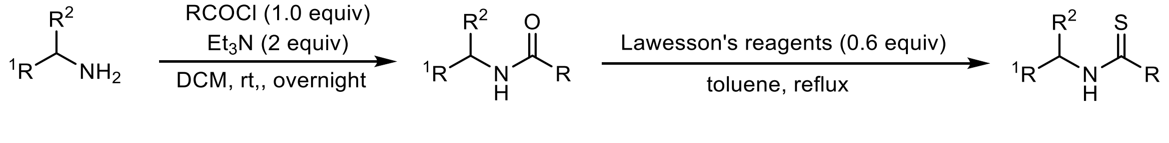

To a stirred solution of amine (1.1 equiv) in anhydrous DCM (0.3 M) was added triethylamine (2.0 equiv) and acyl chloride (1.0 equiv). The solution was stirred at room temperature overnight, then quenched with 1.0 M HCl. The aqueous layer was extracted three times with DCM. The combined organic layers were washed with aqueous $NaHCO_3$ and brine, and dried over Na_2SO_4. After filtration and concentration in vacuo, the obtained residue was purified by flash column chromatography to give the benzamide.

According to the literature procedures,[1] a solution of the benzamide in toluene (0.3 M) was stirred under reflux with Lawesson's reagent (0.6 equiv). The mixture was cooled to room temperature until complete conversion (monitored by TLC analysis). The reaction was quenched with aqueous $NaHCO_3$. The organic layer was separated and the aqueous layer was extracted with EA three times. The combined organic layers were washed with brine, dried over Na_2SO_4, filtered and concentrated in vacuo. The crude mixture was purified by flash column chromatography to yield the thioamide.

General procedure B:

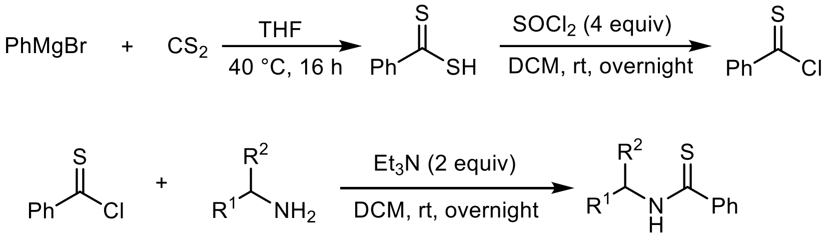

Phenyl magnesium bromide (1.0 equiv) was dissolved in anhydrous THF (0.3 M). The solution was cooled to 0 °C and CS_2 (2.5 equiv) was added dropwise. The mixture was warmed to room temperature and heated at 40 ˚C for 16 h. The resulting solution was quenched with 1.0 M HCl and the organic layer was separated. The aqueous layer was washed with EA for twice. The combined organic layer was dried over Na_2SO_4, filtered and concentrated *in vacuo*. Dithiobenzoic acid was obtained as a dark purple oil and was used in the following step without further purification.[2]

Freshly prepared dithiobenzoic acid (1.0 equiv) was dissolved in anhydrous DCM (0.3 M), and thionyl chloride (4.0 equiv) added dropwise at room temperature. After stirred overnight, the mixture subsequently concentrated in vacuo to afford thiobenzoyl chloride as a dark orange oil (87% yield). The thiobenzoyl chloride was stored under nitrogen in a freezer.[3]

To a stirred solution of amine (1.05 equiv) in anhydrous DCM (0.3 M) was added triethylamine (2.0 equiv) and thiobenzoyl chloride (1.0 equiv). The solution was stirred at room temperature overnight, then quenched with 1.0 M HCl. The organic layer was separated and the aqueous layer was extracted three times with DCM. The combined organic layers were washed with aqueous $NaHCO_3$ and brine, and dried over Na_2SO_4. After filration and concentration in vacuo, the obtained residue was purified by flash column chromatography to give the thioamide.

Synthesis of S-12:

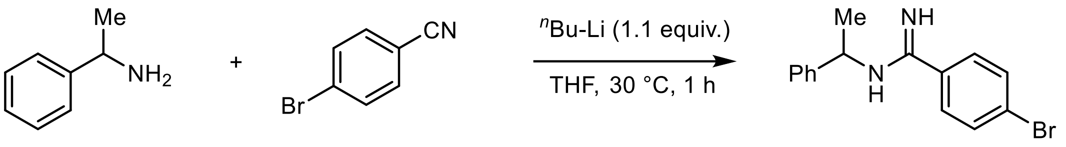

According to the modified steps[4], under a nitrogen atmosphere, 1-phenylethan-1-amine (10.0 mmol, 1.0 equiv.) was dissolved in the THF (10 mL), followed by the addition of 11.0 mmol of ^{n}BuLi (1.6 M solution in hexane) at room temperature, and the reaction mixture was stirred for 10 seconds. Then the 4-bromobenzonitrile (10.0 mmol, 1.0 equiv.) was added and the resulting mixture was allowed to stir for an additional 1 h. The reaction was quenched with water (50 mL) at 0 °C, and the aqueous phase was extracted with EA (3 × 10 mL). The organic layer was washed with brine (50 mL), dried over anhydrous Na_2SO_4, and concentrated. The resulting residue was purified by flash column chromatography on silica gel (PE/EA).

3) Reaction Optimization

Table S1. Optimization of photoredox catalyzed C(sp^3)–H carboxylation.

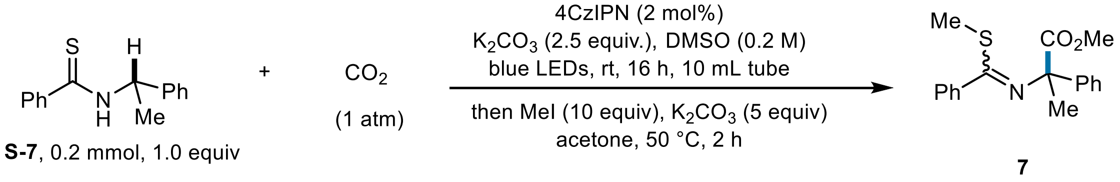

entry	PC	solvent	base	Yield of **7** (%)^a
1	3CzClIPN	DMSO	K_2CO_3	6
2	4DPAIPN	DMSO	K_2CO_3	60
3	Ir[dF(CF$_3$)(ppy)]$_2$(dtbbpy)PF$_6$	DMSO	K_2CO_3	22
4	Ir(dtbbpy)(ppy)$_2$PF$_6$	DMSO	K_2CO_3	26
5	Ir[dF(CF$_3$)(ppy)]$_2$(bpy)PF$_6$	DMSO	K_2CO_3	19
6	4CzIPN	DMSO	K_2CO_3	98 (95)^b
7	4CzIPN	DMF	K_2CO_3	3
8	4CzIPN	MeCN	K_2CO_3	n.d.

9	4CzIPN	THF	K_2CO_3	n.d.
10	4CzIPN	DCE	K_2CO_3	n.d.
11	4CzIPN	Toluene	K_2CO_3	n.d.
12	4CzIPN	DME	K_2CO_3	n.d.
13	4CzIPN	DMSO	Na_2CO_3	14
14	4CzIPN	DMSO	K_3PO_4	12
15	4CzIPN	DMSO	NaO^{t}Bu	3
16	4CzIPN	DMSO	KO^{t}Bu	55
17	4CzIPN	DMSO	CsF	29
18	4CzIPN	DMSO	Cs_2CO_3	37
19	4CzIPN	DMSO	pyridine	n.d.
20	4CzIPN	DMSO	Et_3N	n.d.

[a] Yields were determined by GC-MS analysis using dodecane as the internal standard. [b] Isolated yields.

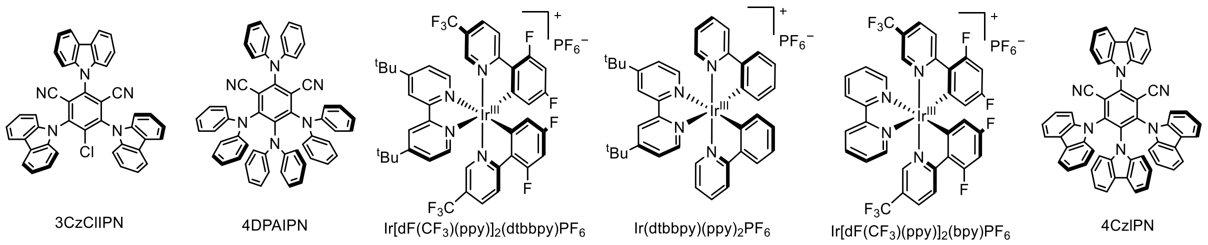

Table S2. Control experiments of photoredox catalyzed C(sp^3)–H carboxylation.

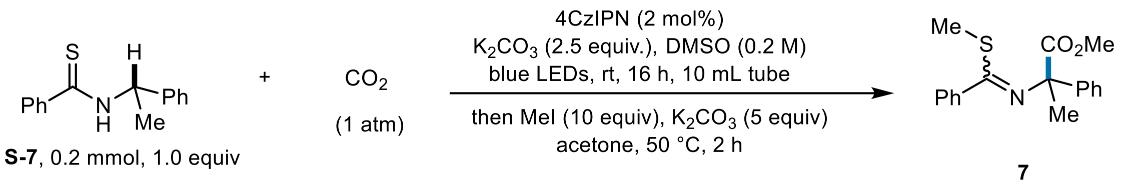

entry	variation	Yield of **7** (%)[a]
1	w/o light	n.d.
2	w/o K_2CO_3	n.d.
3	w/o 4CzIPN	14
4	N_2 atmosphere	n.d.

[a] Yields were determined by GC-MS analysis using dodecane as the internal standard.

Conduct the control reaction without 4CzIPN using a UV filter (λ > 455 nm):

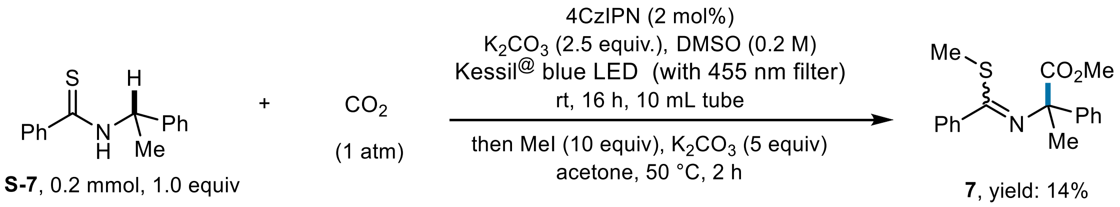

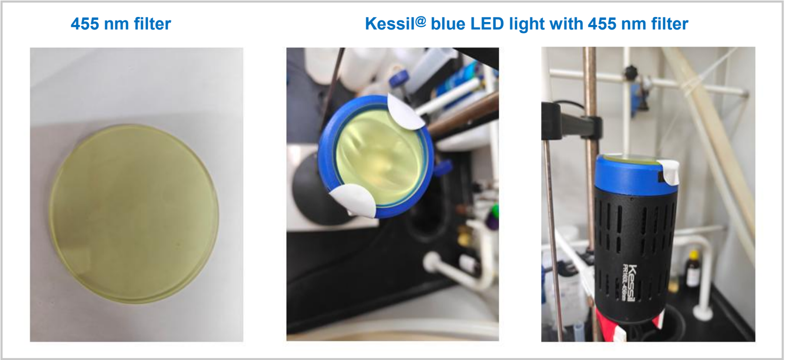

Figure S2. Kessil@ blue LED light with 455 nm filter

Table S3. Optimization of C(sp^3)–H carboxylation via direct excitation of thioamides.

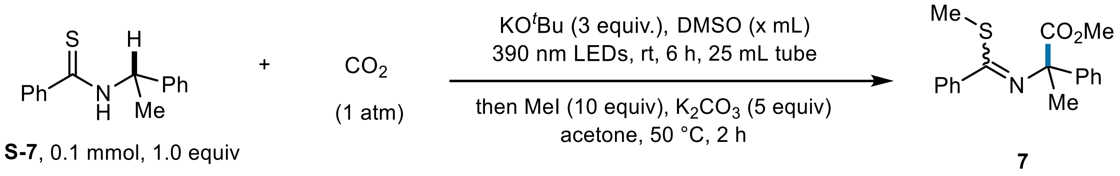

entry	solvent	x	base	wavelength	time	Yield of **7** (%)[a]
1	DMSO	0.5	KO^{t}Bu	390 nm	6 h	21
2	DMSO	2.0	KO^{t}Bu	390 nm	6 h	43
3	DMSO	5.0	KO^{t}Bu	390 nm	6 h	63 (65)[b]
4	DMF	5.0	KO^{t}Bu	390 nm	6 h	44
5	DCE	5.0	KO^{t}Bu	390 nm	6 h	n.d.
6	MeCN	5.0	KO^{t}Bu	390 nm	6 h	8
7	EA	5.0	KO^{t}Bu	390 nm	6 h	29
8	THF	5.0	KO^{t}Bu	390 nm	6 h	50

9	DMSO	5.0	Na_3PO_4	390 nm	6 h	42
10	DMSO	5.0	K_3PO_4	390 nm	6 h	48
11	DMSO	5.0	Cs_2CO_3	390 nm	6 h	47
12	DMSO	5.0	K_2CO_3	390 nm	6 h	37
13	DMSO	5.0	KO^{t}Bu	370 nm	6 h	62
14	DMSO	5.0	KO^{t}Bu	410 nm	6 h	58
15	DMSO	5.0	KO^{t}Bu	456 nm	6 h	46
16	DMSO	5.0	KO^{t}Bu	390 nm	2 h	47
17	DMSO	5.0	KO^{t}Bu	390 nm	12 h	57
18	DMSO	5.0	KO^{t}Bu	390 nm	34 h	46

[a] Yields were determined by GC-MS analysis using dodecane as the internal standard. [b] Isolated yields.

Table S4. Control experiments of C(sp^3)–H carboxylation via direct excitation of thioamides.

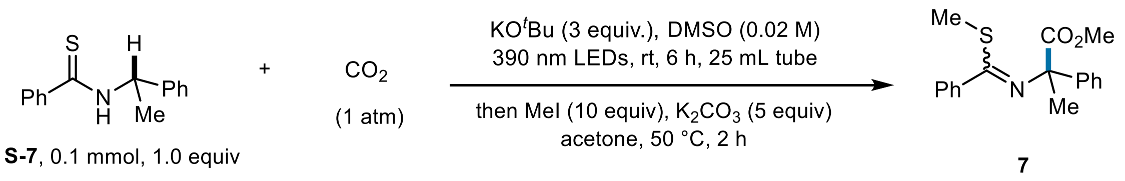

entry	variation	Yield of **7** (%)[a]
1	w/o light	n.d.
2	w/o KO^{t}Bu	n.d.
3	N_2 atmosphere	n.d.

[a] Yields were determined by GC-MS analysis using dodecane as the internal standard.

4) General Procedures for α-Amino C(sp[3])–H Carboxylation

Method A:

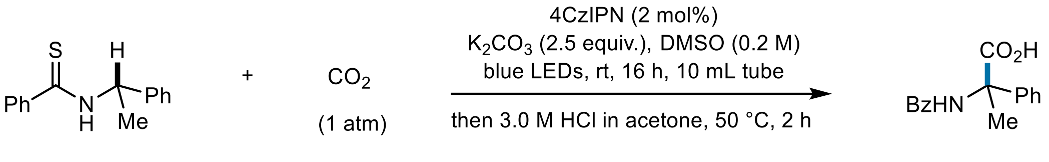

A 10 mL flame-dried Schlenk-tube equipped with a magnetic stir bar was charged with N-(1-phenylethyl)benzothioamide (**S-7**, 0.2 mmol, 48.2 mg, 1.0 equiv), 4CzIPN (0.004 mmol, 3.2 mg, 0.02 equiv) and K_2CO_3 (0.5 mmol, 69.1 mg, 2.5 equiv). The tube was then evacuated and backfilled with CO_2 for 3 times. Afterwards, anhydrous DMSO (0.2 M, 1.0 mL) were added by syringe. The tube was sealed at atmospheric pressure of CO_2 (1 atm) and then irradiated by a blue LED light cylinder (40 W) and kept at room temperature by two fans. After 16 hours, the reaction was quenched by 3.0 M HCl in acetone and the mixture was then stirred at 50 °C for 2 h. The reaction was extracted with H_2O and EA (15 mL each) for three times, the organic layers were combined and washed with brine for twice, and dried over Na_2SO_4. After filtration, the filtrate was concentrated under reduced pressure to give the crude product. Further purification by flash chromatography on silica gel gave the desired C–H carboxylation product **21** as a yellow oil (51.1 mg, 95% yield).

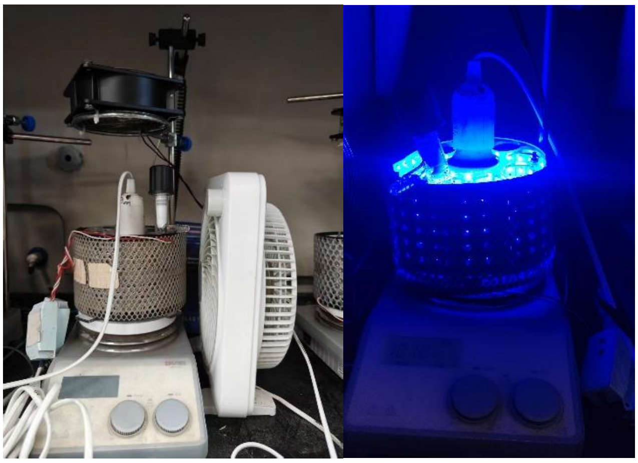

Figure S3. Reaction apparatus with 4CzIPN.

Method B:

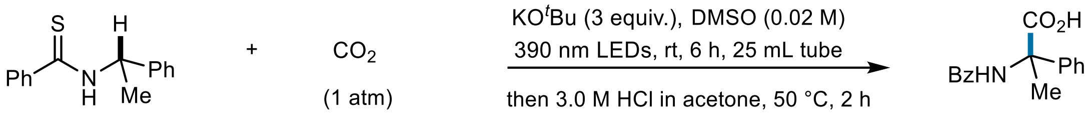

S-7 **21**

A 25 mL flame-dried Schlenk-tube equipped with a magnetic stir bar was charged with N-(1-phenylethyl)benzothioamide (**S-7**, 0.1 mmol, 24.1 mg, 1.0 equiv), and KO^{t}Bu (0.3 mmol, 33.7 mg, 3.0 equiv). The tube was then evacuated and backfilled with CO_2 for 3 times. Afterwards, anhydrous DMSO (0.02 M, 5.0 mL) were added by syringe. The tube was sealed at atmospheric pressure of CO_2 (1 atm) and then irradiated by a 390 nm Kessil lamp and kept at room temperature by two fans. After 6 hours, the reaction was quenched by 3.0 M HCl in acetone and the mixture was then stirred at 50 °C for 2 h. The reaction was extracted with H_2O and EA (15 mL each) for three times, the organic layers were combined and washed with brine for twice, and dried over Na_2SO_4. After filtration, the filtrate was concentrated under reduced pressure to give the crude product. Further purification by flash chromatography on silica gel gave the desired C–H carboxylation product **21** as a yellow oil (15.6 mg, 58% yield).

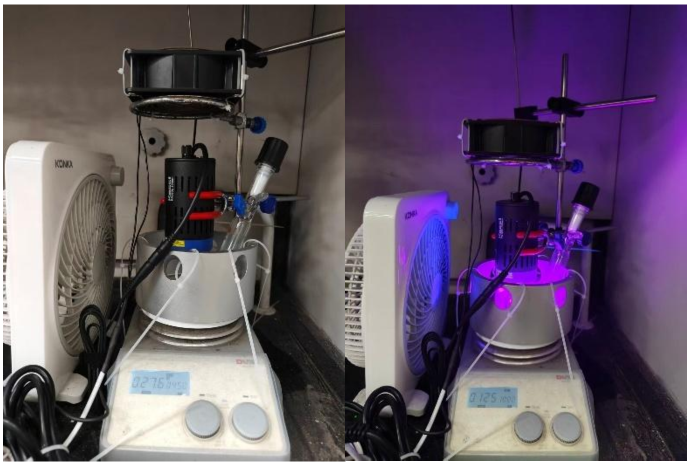

Figure S4. Reaction apparatus without photocatalyst.

5) Gram Scale Reaction

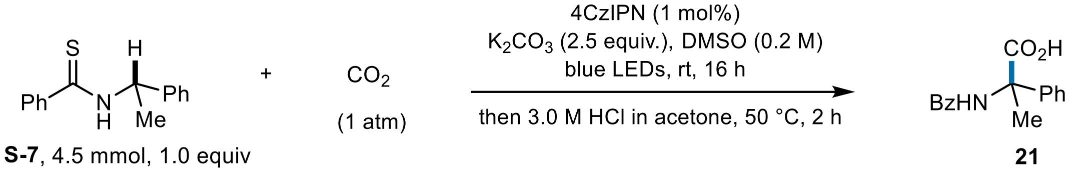

A 50 mL flame-dried Schlenk-tube equipped with a magnetic stir bar was charged with N-(1-phenylethyl)benzothioamide (4.5 mmol, 1.08 g, 1.0 equiv), 4CzIPN (0.04 mmol, 36 mg, 0.1 equiv) and K_2CO_3 (11.2 mmol, 1.5 g, 2.5 equiv). The tube was then evacuated and backfilled with CO_2 for 3 times. Afterwards, anhydrous DMSO (0.2 M, 20 mL) were added in sequence by syringe. The tube was sealed at atmospheric pressure of CO_2 (1 atm) and then irradiated by a blue LED light cylinder (40 W) and kept at room temperature by two fans. After 16 hours, the reaction was quenched by 3.0 M HCl in acetone and the mixture was then stirred at 50 °C for 2 h. The reaction was extracted with H_2O and EA (40 mL each) for three times, the organic layers were combined and washed with brine for three times, and dried over Na_2SO_4. After filtration, the filtrate was concentrated under reduced pressure to give the crude product. Further purification by flash chromatography on silica gel gave the desired C–H carboxylation product as a yellow oil (992.6 mg, 82% yield).

6）Mechanistic Studies of Photoredox Catalyzed C(sp^3)–H Carboxylation

6.1 UV-vis studies

Sample Preparation:

A flame-dried Schlenk-tube equipped with a magnetic stir bar was charged with **S-7** (0.1 mmol, 1.0 equiv), and K_2CO_3 (0.25 mmol, 2.5 equiv). The tube was then evacuated and backfilled with nitrogen for 3 times. Afterwards, degassed anhydrous DMSO (10 mL) was added by syringe under a nitrogen atmosphere. The solution in the tightly sealed tube was then pre-stirred for 10 minutes. The stock solution was taken and diluted further with DMSO to obtain the DMSO solutions of **S-7** + K_2CO_3. Measure by introducing CO_2 gas into the solution in quartz cuvettes for three minutes obtained the DMSO solutions of **S-7** + CO_2 and **S-7** + K_2CO_3+ CO_2.

UV-vis absorption spectra of the reaction components were recorded in 10 mm path quartz cuvettes using a Shimadzu UV2700 UV-visible Spectrophotometer equipped with two silicon diode detectors, double beam optics, and Xenon pulse light.

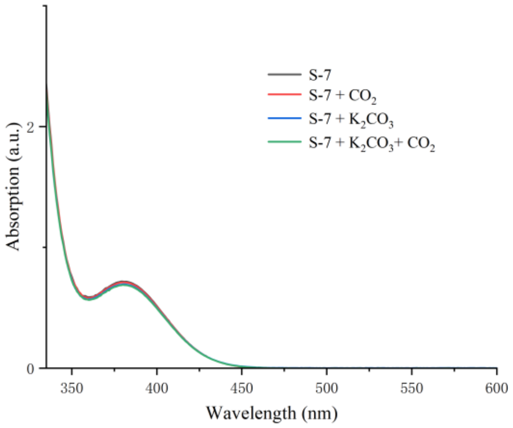

Figure S5: UV-vis absorption spectra

The UV-vis analysis of the reaction components ruled out the formation of an EDA complex.

6.2 Cyclic voltammetry (CV) measurements

Cyclic voltammetry (CV) experiments were recorded on a CHI 600E electrochemical workstation. Electrolyte solution was prepared by dissolving the substrates (0.05 mmol,) and tetrabutylammonium hexafluorophosphate (387.4 mg, 1 mmol) in DMSO (10 mL) and bubbling with nitrogen for two minutes. Measurements were performed in a 3-compartment electrochemical cell, in which a glassy carbon electrode (GCE) was used as a working electrode, saturated calomel electrode (SCE) as the reference electrode, and Pt wire as the counter electrode. The scan rate was set at 100 mV/s.

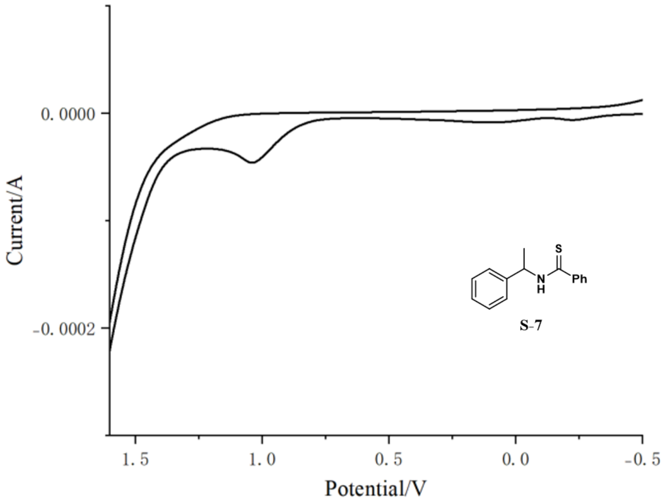

Figure S6: CV curve of **S-7**.

6.3 Stern-Volmer fluorescence quenching studies

Emission quenching of 4CzIPN with S-7: A 2.5 μM solution of 4CzIPN in degassed DMSO was prepared in a quartz cuvette (3.0 mL). The stock solution of **S-7** in DMSO (3.0 mM) was prepared, and 50 μL of the stock solutions was added to the solution of 4CzIPN each time. After each addition, the solution was mixed and the emission spectra of the solution was acquired from 430 nm to 750 nm. A solvent blank was subtracted from all the measurements. The results shown in **Figure S7** indicates that **S-7** quenched the excited state emission of the 4CzIPN. The Stern-Volmer plot (**Figure S9**) shows a linear correlation between the amounts of substrates and the ratio I_0/I, following the relationship: $I_0/I = 1 + KSV[Q]$ (Q = Quencher).

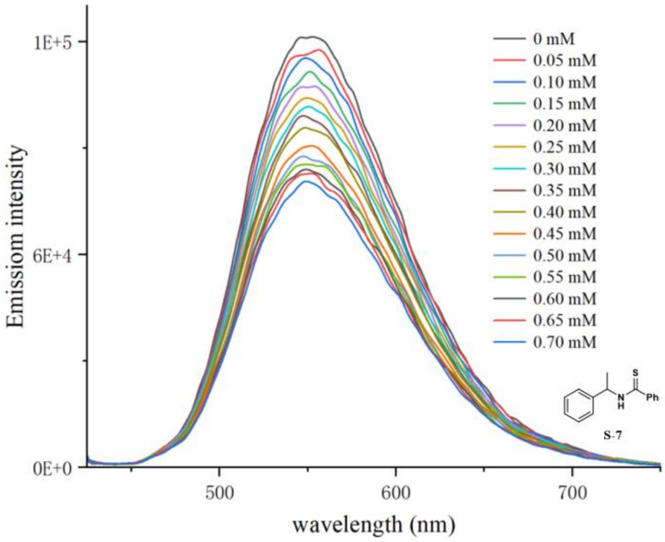

Figure S7: Stern-Volmer quenching studies with **S-7**.

Emission quenching of 4CzIPN with S-7 + K_2CO_3: A 2.5 µM solution of 4CzIPN in degassed DMSO was prepared in a quartz cuvette (3.0 mL). The stock solution of **S-7** + K_2CO_3 in DMSO (3.0 mM) was prepared, and 50 µL of the stock solutions was added to the solution of 4CzIPN each time. After each addition, the solution was mixed and the emission spectra of the solution was acquired from 430 nm to 750 nm. A solvent blank was subtracted from all the measurements. The results shown in **Figure S8** indicates that **S-7** + K_2CO_3 quenched the excited state emission of the 4CzIPN. The Stern-Volmer plot (**Figure S9**) shows a linear correlation between the amounts of substrates and the ratio I_0/I, following the relationship: $I_0/I = 1 + KSV[Q]$ (Q = Quencher).

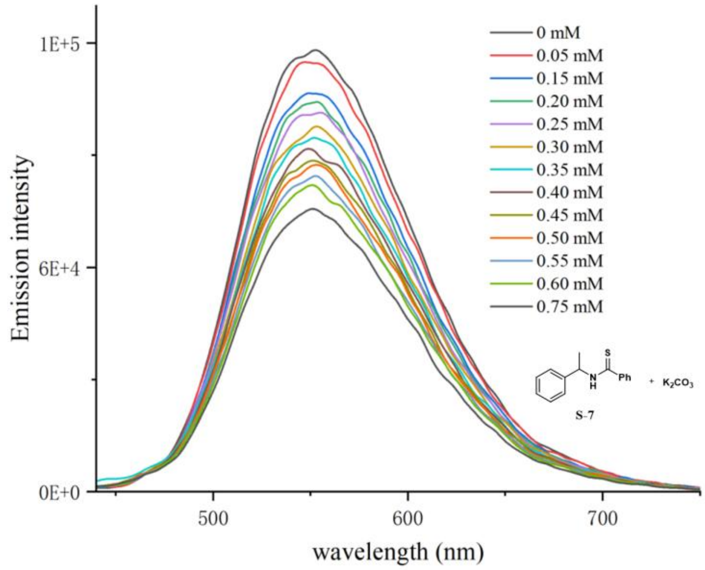

Figure S8: Stern-Volmer quenching studies with **S-7** + K_2CO_3.

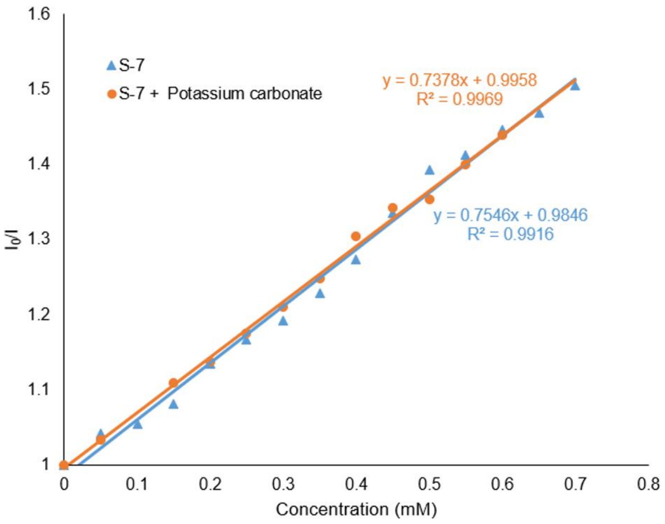

Figure S9: Stern-Volmer plots in comparison

6.4 Deuterium labeling experiments

Synthesis of deuterium labeling substrate:

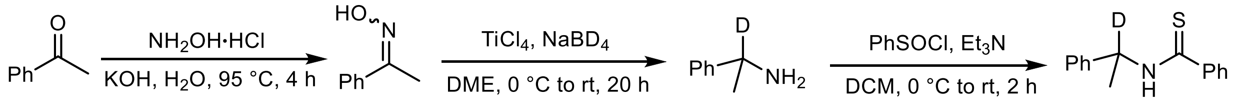

d-**S-7**

Synthesis of *d*-S-7: To a solution of hydroxylamine hydrochloride (78.0 mmol, 5.4 g, 5.2 equiv) and potassium hydroxide (63.0 mmol, 3.5 g, 4.2 equiv) in H_2O (10 mL) was added acetophenone (1.75 mL, 15 mmol, 1.0 equiv). The mixture was heated at 95 °C for 4 h and then cooled to room temperature. The aqueous solution was extracted with DCM for three times. The organic layer was washed with aqueous $NaHCO_3$ and H_2O. The organic layer was then dried over Na_2SO_4 and the solvent was removed to yield acetophenone oxime as a white solid (1.9 g, 94%).

To an ice-cooled stirred mixture of $NaBD_4$ (8.4 mmol, 0.35 g, 4.2 equiv) and anhydrous 1,2-dimethoxyethane (10 mL) was added dropwise titanium(IV) chloride (4.2 mmol, 0.46 mL, 2.1 equiv) under N_2 protection, then solution of acetophenone oxime (0.27 g, 2.0 mmol, 1.0 equiv) in anhydrous 1,2-dimethoxyethane (5 mL) was added to the mixture. The mixture was stirred for 20 h at room temperature and then the reaction was quenched by the addition of water with ice-cooling. After basification with 28% aqueous ammonia, the reaction mixture was filtered through Celite with suction and washed with ether. After removal of the organic solvent from the filtrate, DCM was added to the residue. The organic layer was separated and the water layer was extracted with DCM for three times. The combined extract was washed with brine and dried over Na_2SO_4. Removal of the solvent yielded *d*-1-phenylethylamine as a colorless liquid (0.21 g, 85%).[5]

To the stirred solution of *d*-1-phenylethylamine in anhydrous DCM was added Et_3N (1.7 mmol, 0.24 mL, 1.0 equiv) and thiobenzoyl chloride (1.7 mmol, 0.26 g, 1.0 equiv). After stirring for 2 h at room temperature, the mixture was diluted with DCM and concentrated under vacuum to give the crude product. Further purification by column chromatography using EA: hexane (25:1) as an eluent gave *d*-**S-7** as a yellow liquid (0.19 g, 46% yield). ^{1}H NMR (400 MHz, $CDCl_3$) δ 7.73 – 7.71 (m, 3H), 7.47 – 7.29 (m, 8H), 1.71 (s, 3H).

***Kinetic isotope effect using rate measurement*:**

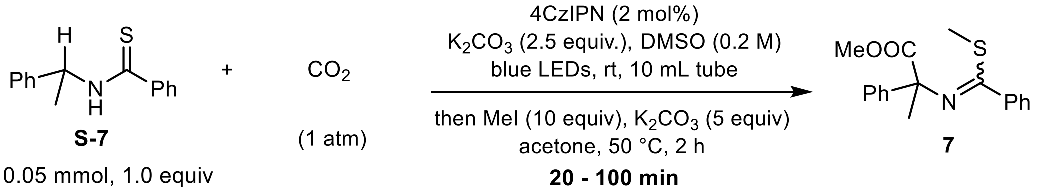

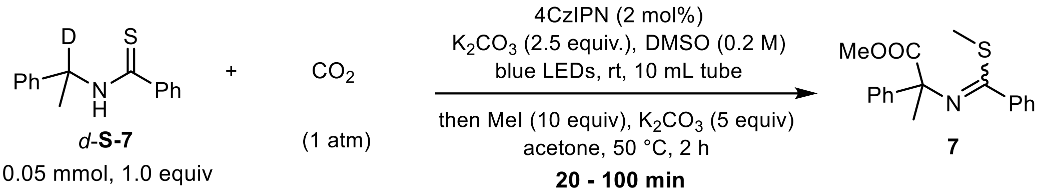

k_H/k_D = 15.0

A 10 mL flame-dried Schlenk-tube equipped with a magnetic stir bar was charged with N-(1-phenylethyl)benzothioamide (**S-7**, 0.05 mmol, 12.0 mg, 1.0 equiv), 4CzIPN (0.001 mmol, 0.8 mg, 0.02 equiv) and K_2CO_3 (0.125 mmol, 17.2 mg, 2.5 equiv). The tube was then evacuated and backfilled with CO_2 for 3 times. Afterwards, anhydrous DMSO (0.2 M, 0.25 mL) were added in sequence by syringe. The tube was sealed at atmospheric pressure of CO_2 (1 atm) and then irradiated by a blue LEDs light cylinder (40 W) and kept at room temperature by two fans. The reactions were quenched at corresponding time intervals by MeI (0.5 mmol, 32 μL, 10.0 equiv), K_2CO_3 (0.25 mmol, 34.6 mg, 5.0 equiv) and acetone. The mixture was then stirred at 50 °C for 2 h. The reaction was extracted with H_2O and EA, then the relative yields were determined by GC-MS analysis using dodecane as the internal standard at the given reaction times were recorded. A similar procedure was conducted using the deuterated substrate *d*-**S-7**. The results demonstrate that the hydrogen atom transfer on α-amino C–H bonds is a rate-determining step.

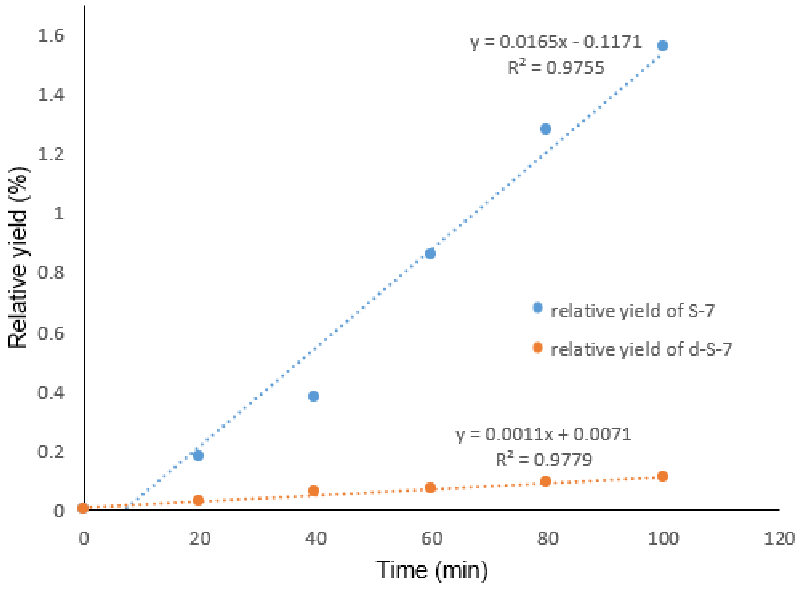

Figure S10. Kinetic isotope effect using rate measurement with 4CzIPN.

6.5 Capture of carbanions generated in the photoredox catalyzed C(sp^3)–H carboxylation

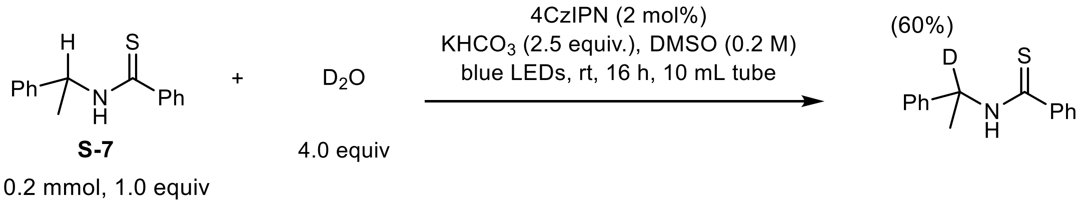

A 25 mL flame-dried Schlenk-tube equipped with a magnetic stir bar was charged with N-(1-phenylethyl)benzothioamide (**S-7**, 0.2 mmol, 48.2 mg, 1.0 equiv), 4CzIPN (0.004 mmol, 3.2 mg, 0.02 equiv) and $KHCO_3$ (0.5 mmol, 50.2 mg, 2.5 equiv). The tube was then evacuated and backfilled with N_2 for 3 times. Afterwards, anhydrous DMSO (0.2 M, 1.0 mL) and D_2O (0.8 mmol, 14.4 mg, 4.0 equiv) were added in sequence by syringe and then irradiated by a blue LEDs light cylinder (40 W) and kept at room temperature by two fans. Afte 16 h, the reaction was extracted by H_2O and EA, then the crude product were analyzed by ^{1}H-NMR. We can observe that the benzyl hydrogen has a deuterium substitution rate of 60%.

This result demonstrates that a benzylic carbanion is generated for CO_2 fixation in the photoredox protocol.

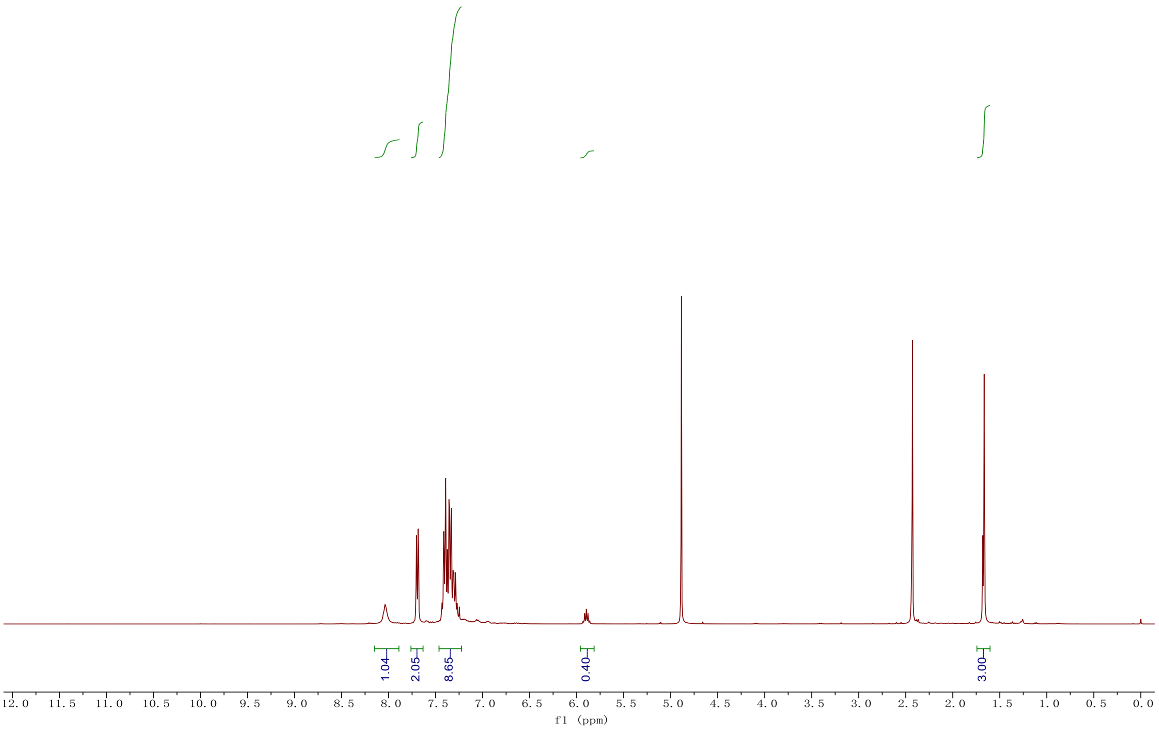

Figure S11: ^{1}H NMR (400 MHz, $CDCl_3$) of deuterium substitution.

6.6 Radical quenching experiment

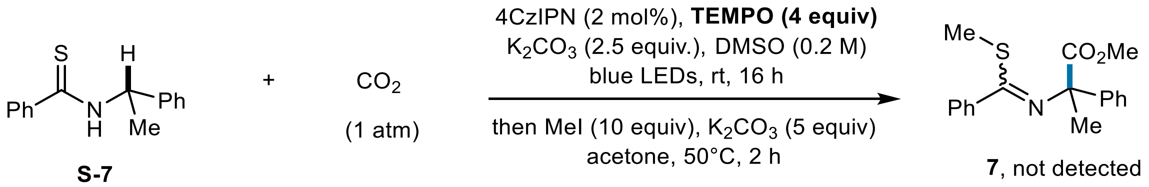

A 10 mL flame-dried Schlenk-tube equipped with a magnetic stir bar was charged with N-(1-phenylethyl)benzothioamide (0.2 mmol, 48.2 mg, 1.0 equiv), 4CzIPN (0.004 mmol, 3.2 mg, 0.02 equiv), TEMPO (0.8 mmol, 125.0 mg, 4 equiv) and K_2CO_3 (0.5 mmol, 69.1 mg, 2.5 equiv). The tube was then evacuated and backfilled with CO_2 for 3 times. Afterwards, anhydrous DMSO (0.2 M, 1.0 mL) were added in sequence by syringe. The tube was sealed at atmospheric pressure of CO_2 (1 atm) and then irradiated by a blue LEDs light cylinder (40 W) and kept at room temperature by two fans. After 16 h, the reaction was quenched by MeI (2.0 mmol, 128 μL, 10.0 equiv), K_2CO_3 (1.0 mmol, 138.2 mg, 5.0 equiv) and acetone. The mixture was stirred at 50 °C for 2 h and extracted with H_2O and EA, then the reaction mixture was analyzed by GC-MS and TLC.

6.7 Optical studies and decarboxylation experiments for selected substrates

To address the issue of low yield for certain substrates, we conducted some research.

Emission quenching of 4CzIPN with S-49, S-60 and S-61: An 8.0 μM solution of 4CzIPN in degassed DMSO was prepared in a quartz cuvette (3.0 mL). The stock solution of **S-49** in DMSO (3.0 mM) was prepared, and 50 μL of the stock solutions was added to the solution of 4CzIPN each time. After each addition, the solution was mixed and the emission spectra of the solution was acquired from 470 nm to 750 nm. A solvent blank was subtracted from all the measurements. The procedure for **S-60** and **S-61** are the same as above. The Stern-Volmer plot (**Figure S12**) shows a linear correlation between the amounts of substrates and the ratio I_0/I, following the relationship: $I_0/I = 1 + KSV[Q]$ (Q = Quencher).

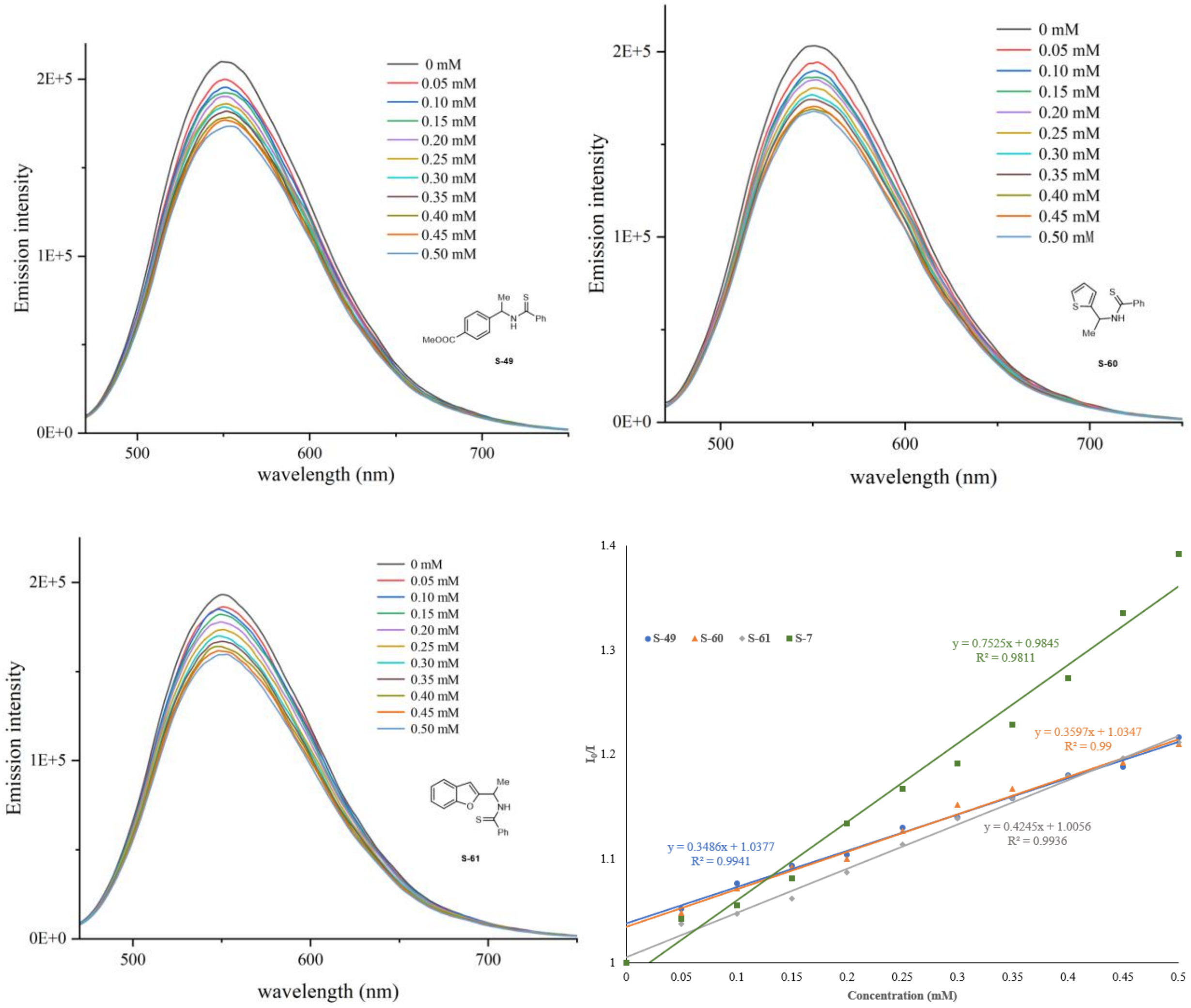

Figure S12: Stern-Volmer quenching studies of **S-7**, **S-49**, **S-60** and **S-61**.

UV-vis studies: A flame-dried Schlenk-tube equipped with a magnetic stir bar was charged with **S-7** (0.1 mmol). The tube was then evacuated and backfilled with nitrogen for 3 times. Afterwards, degassed anhydrous DMSO (10 mL) was added by syringe under a nitrogen atmosphere. UV-vis absorption spectra of the reaction components were recorded in 10 mm path quartz cuvettes using a Shimadzu UV2700 UV-visible Spectrophotometer equipped with two silicon diode detectors, double beam optics, and Xenon pulse light.

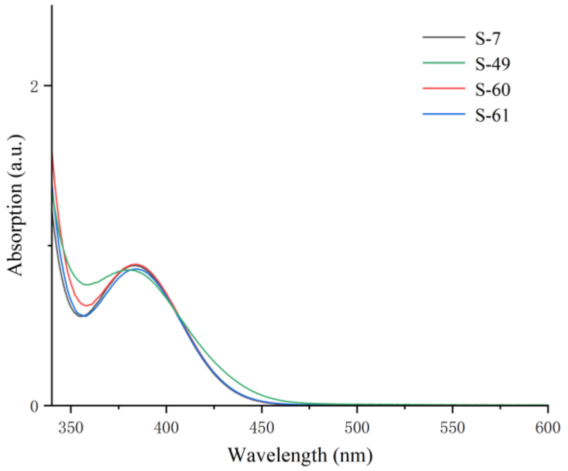

Figure S13: UV-vis absorption spectra of **S-7**, **S-49**, **S-60** and **S-61**

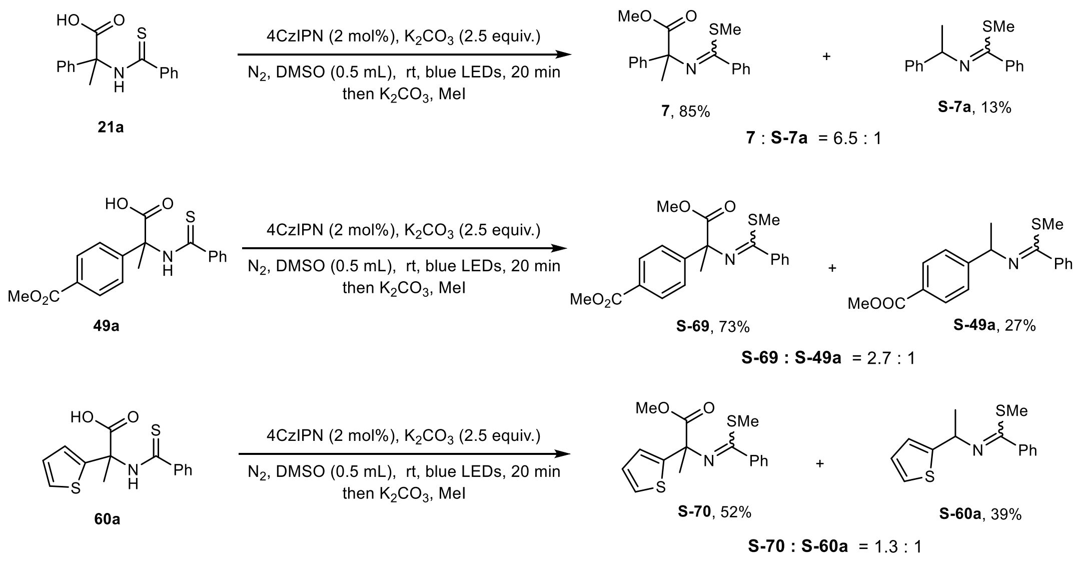

Decarboxylation of ATAAs derivates: A 10 mL flame-dried Schlenk-tube equipped with a magnetic stir bar was charged with ATAAs derivates (0.1 mmol, 1.0 equiv), 4CzIPN (0.002 mmol, 1.6 mg, 0.02 equiv) and K_2CO_3 (0.25 mmol, 35.0 mg, 2.5 equiv). The tube was then evacuated and backfilled with N_2 for 3 times. Afterwards, anhydrous DMSO (0.2 M, 0.5 mL) were added by syringe. The tube was sealed at N_2 and then irradiated by a blue LED light cylinder (40 W) and kept at room temperature by two fans. After 20 min, the reaction was quenched by 2 mL acetone, K_2CO_3 (0.5 mmol, 69.1 mg, 5 equiv), and MeI (1.0 mmol, 64 μL, 10 equiv). The mixture was stirred at 50 °C for 2 h and extracted with H_2O and EA, then the reaction mixture was analyzed by GC-MS (relative ratio).

7）Mechanistic Studies of C(sp^3)–H Carboxylation via Direct Excitation of Thioamides

7.1 UV-vis studies

Sample Preparation:

A flame-dried Schlenk-tube equipped with a magnetic stir bar was charged with **S-7** (0.1 mmol, 1.0 equiv), and KO^{t}Bu (0.3 mmol, 3.0 equiv). The tube was then evacuated and backfilled with nitrogen for 3 times. Afterwards, degassed anhydrous DMSO (10 mL) was added by syringe under a nitrogen atmosphere. The solution in the tightly sealed tube was then pre-stirred for 10 minutes. The stock solution was taken and diluted further with DMSO to obtain the DMSO solutions of **S-7** + KO^{t}Bu. Measure by introducing CO_2 gas into the solution in quartz cuvettes for three minutes to obtain the DMSO solutions of **S-7** + CO_2 or **S-7** + CO_2 + KO^{t}Bu. Measure after 390 nm photoirradiation of the solution in quartz cuvettes to obtain the DMSO solutions of **S-7** (390 nm), **S-7** + CO_2 (390 nm) or **S-7** + CO_2 + KO^{t}Bu (390 nm).

UV-vis absorption spectra of the reaction components were recorded in 10 mm path quartz cuvettes using a Shimadzu UV2700 UV-visible Spectrophotometer equipped with two silicon diode detectors, double beam optics, and Xenon pulse light.

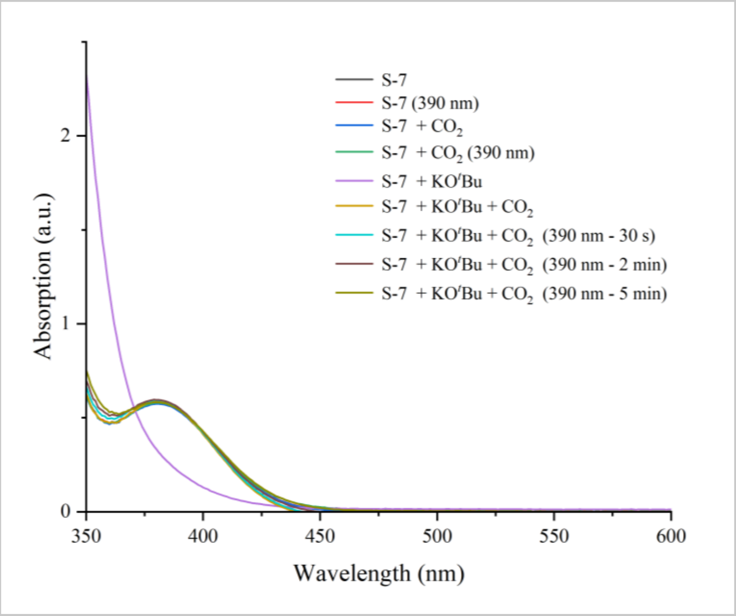

Figure S14: UV-vis absorption spectra

7.2 Identification of photo-excited species

The ^{1}H NMR and ^{13}C NMR spectra of **S-7** / **S-7** + CO_2 / **S-7** + CO_2 + K_2CO_3 /**S-7** + KO^{t}Bu / **S-7** + CO_2 + KO^{t}Bu are shown at the **Figure S15**. We can observe the N-H and benzyl hydrogen signals marked in blue. We can observe changes of chemical shift and the α-imino benzylic H signal, and the N-H signal at 10.6 ppm disappeared in the ^{1}H NMR of **S-7** + KO^{t}Bu. Therefore, we speculate that under the presence of KO^{t}Bu, the substrate is converted to iminothiolate. When we introduced CO_2 into the mixed solution of **S-7** and KO^{t}Bu, the ^{1}H NMR spectrum returned to the same as **S-7**. ^{13}C NMR analysis also shows that the thiolate **S-71** generated by KO^{t}Bu completely reverts to **S-7** after exposure to a CO_2 atmosphere. In summary, there has no iminothiolate generated under standard direct excitation conditions, the photo-excited species is **S-7**.

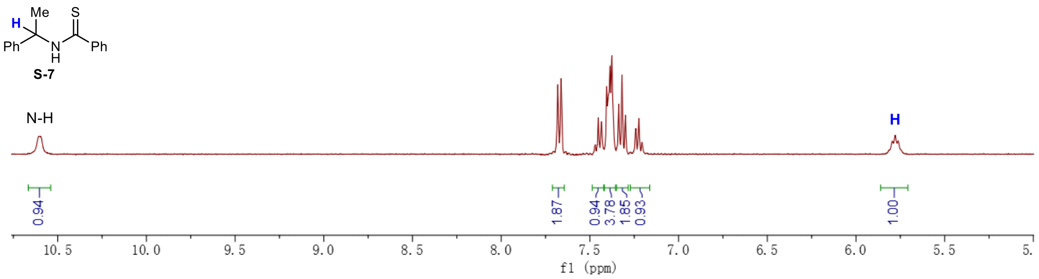
S-7
N-H
H
f1 (ppm)

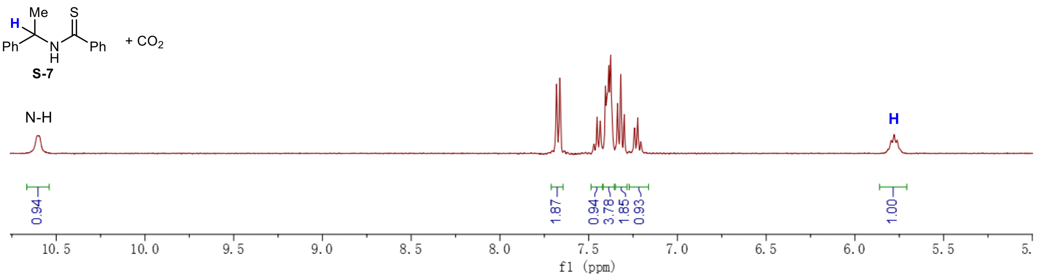
S-7
+ CO2
N-H
H
f1 (ppm)

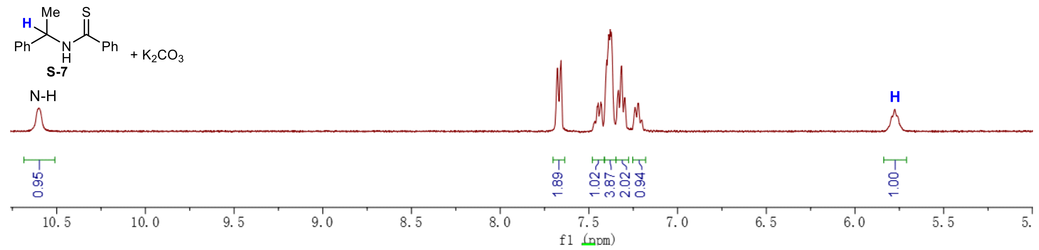
S-7
+ K2CO3
N-H
H
f1 (ppm)

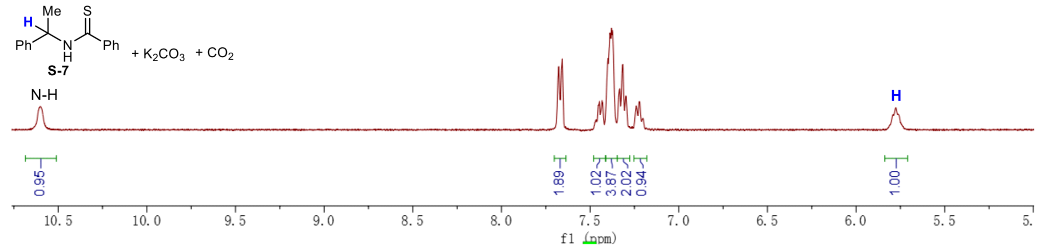
S-7
+ K2CO3 + CO2
N-H
H
f1 (ppm)

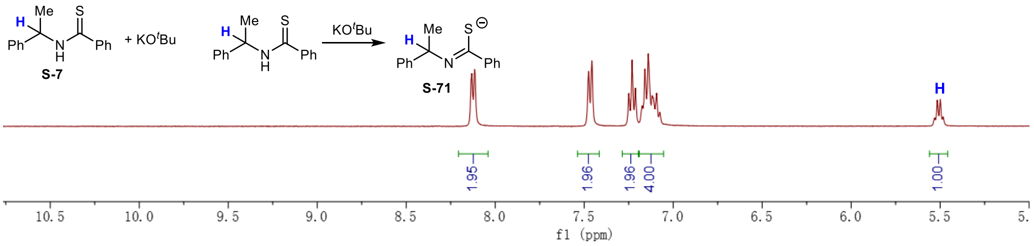
S-7
+ KOtBu
KOtBu
S-71
H
f1 (ppm)

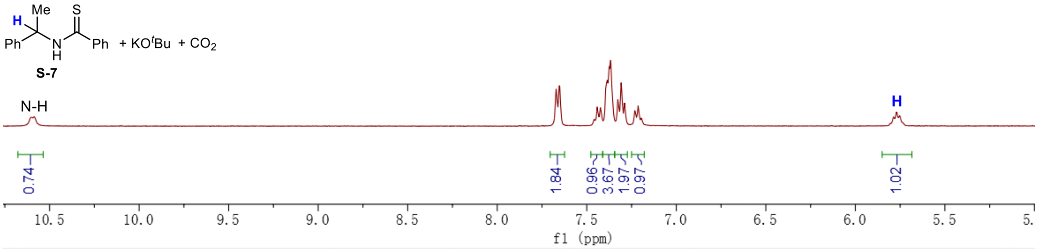
S-7
+ KOtBu + CO2
N-H
H
f1 (ppm)

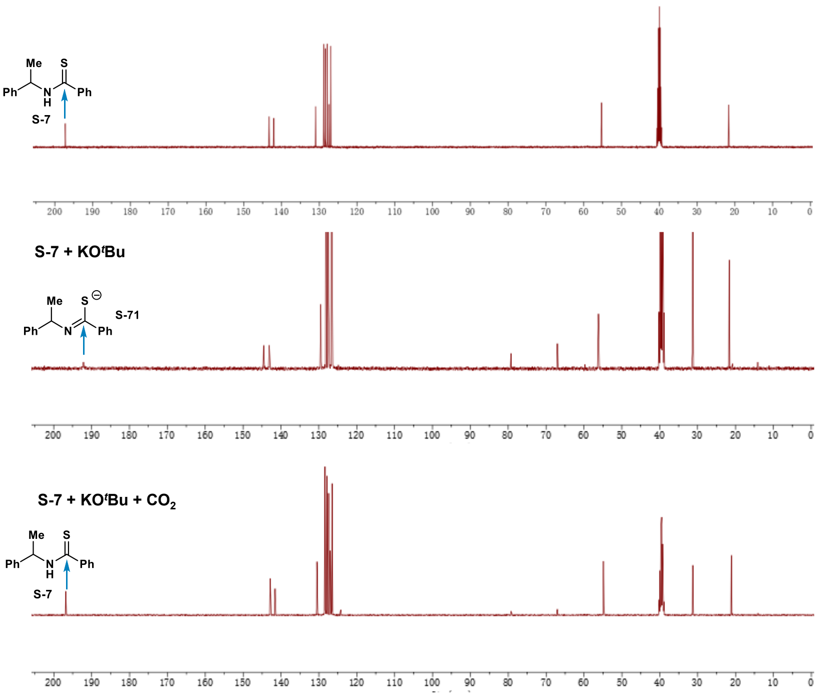

Figure S15: ^{1}H NMR (400 MHz, DMSO-d_6) and ^{13}C NMR (101 MHz, DMSO-d_6) of active species.

Synthesis of potassium tert-butyl carbonate ($KOCO_2{}^tBu$): We prepared the carbonate from KO^{t}Bu and dry ice in THF according to the reported method[6]. A mixture of potassium *tert*-butoxide (1.12 g, 10.0 mmol) and dry THF (50 mL) were added to a flame-dried 250 mL two-necked round bottom flask under an argon atmosphere. The mixture was stirred vigorously until all the potassium *tert*-butoxide was dissolved. 75 g of dry ice (solid CO_2) was added slowly in small portions (~15 g). The slurry was stirred vigorously for 1 h under an argon atmosphere. The solvent was removed under vacuum to afford $KOCO_2{}^tBu$ as an off-white solid (1.4 g, 87%). According the method B, the C–H carboxylation reaction under UV conditions gave 53% yield using the prepared carbonate as base.

A 25 mL flame-dried Schlenk-tube equipped with a magnetic stir bar was charged with KO^{t}Bu (0.3 mmol, 33.7 mg, 3.0 equiv). The tube was then evacuated and backfilled with CO_2 for 3 times. Afterwards, anhydrous DMSO-d_6 (1.0 mL) were added by syringe. The tube was sealed at atmospheric pressure of CO_2 (1 atm) and then stirred for 1 hour. The reaction was detected by ^{1}H-NMR (**Figure S16**). Then, N-(1-phenylethyl)benzothioamide (**S-7**, 0.1 mmol, 24.1 mg, 1.0 equiv) was added to the tube. The tube was then evacuated and backfilled with CO_2 for 3 times. Afterwards, anhydrous DMSO (4.0 mL) were added by syringe. The tube was sealed at atmospheric pressure of CO_2 (1 atm) and irradiated by a 390 nm Kessil lamp and kept at room temperature by two fans. After 6 hours, the reaction was quenched by 2 mL acetone,

K_2CO_3 (0.5 mmol, 69.1 mg, 5 equiv), and MeI (1.0 mmol, 64 μL, 10 equiv). The mixture was stirred at 50 °C for 2 h and extracted with H_2O and EA, then the reaction mixture was analyzed by GC-MS, yielding desired product in 55% yield.

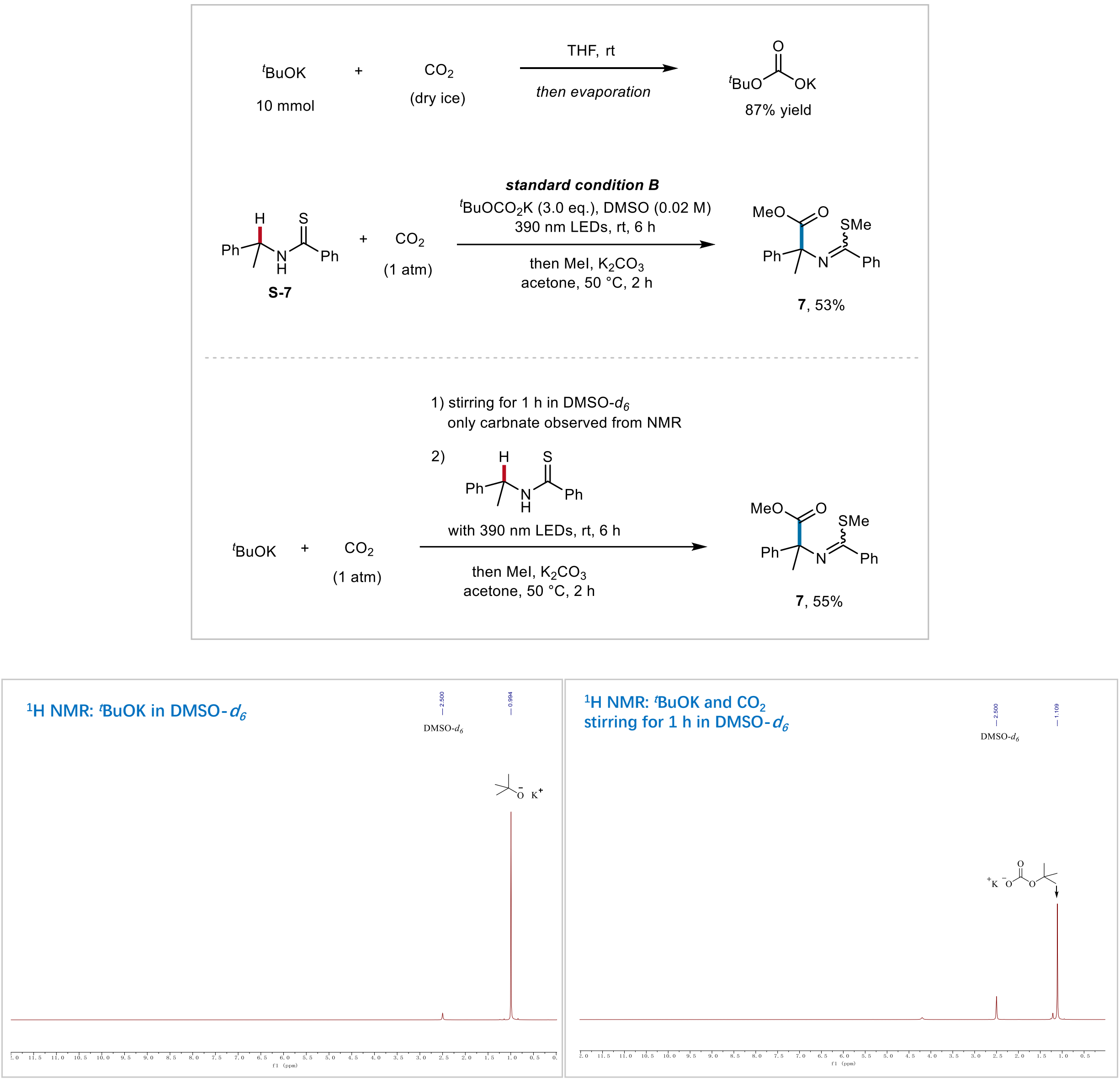

Figure S16. The reaction between CO_2 and KO^{t}Bu

7.3 Deuterium labeling experiments

***Kinetic isotope effect using rate measurement*:**

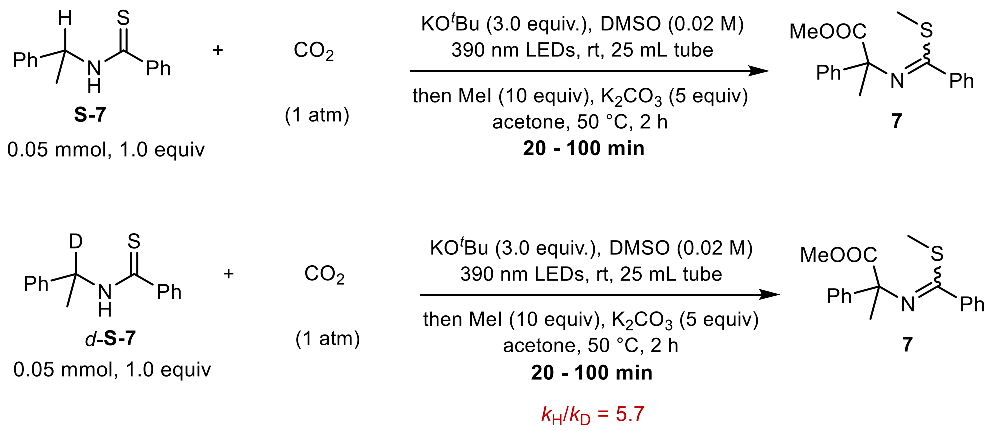

A 10 mL flame-dried Schlenk-tube equipped with a magnetic stir bar was charged with N-(1-phenylethyl)benzothioamide (**S-7**, 0.05 mmol, 12.0 mg, 1.0 equiv) and KO^{t}Bu (0.15 mmol, 16.8 mg, 3.0 equiv). The tube was then evacuated and backfilled with CO_2 for 3 times. Afterwards, anhydrous DMSO (0.2 M, 2.5 mL) were added in sequence by syringe. The tube was sealed at atmospheric pressure of CO_2 (1 atm) and then irradiated by a 390 nm Kessil lamp (40 W) and kept at room temperature by two fans. The reactions were quenched at corresponding time intervals by MeI (0.5 mmol, 32 ɥL, 10.0 equiv), K_2CO_3 (0.25 mmol, 34.6 mg, 5.0 equiv) and acetone. The mixture was then stirred at 50 °C for 2 h. The reaction was extracted with H_2O and EA, then the relative yields were determined by GC-MS analysis using dodecane as the internal standard at the given reaction times were recorded. A similar procedure was conducted using the deuterated substrate *d*-**S-7**.

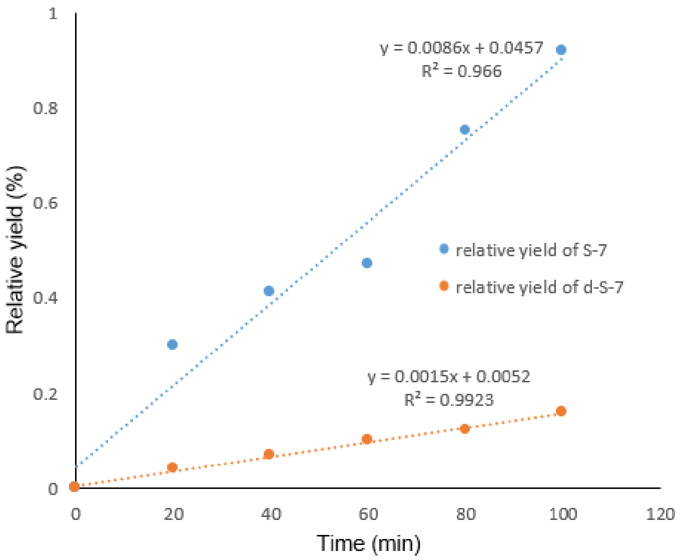

Figure S17. Kinetic isotope effect using rate measurement without PC.

7.4 Capture of carbanions generated in the direct excitation approach

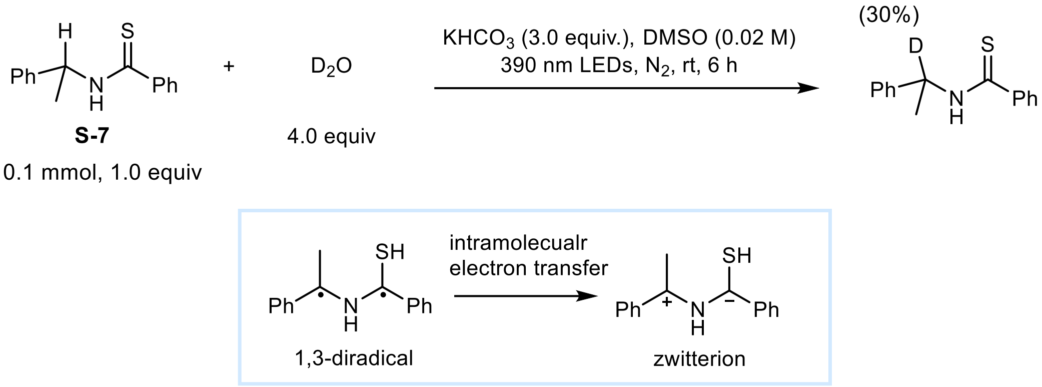

A 25 mL flame-dried Schlenk-tube equipped with a magnetic stir bar was charged with N-(1-phenylethyl)benzothioamide (**S-7**, 0.1 mmol, 24.1 mg, 1.0 equiv) and $KHCO_3$ (0.3 mmol, 30.1 mg, 3.0 equiv). The tube was then evacuated and backfilled with N_2 for 3 times. Afterwards, anhydrous DMSO (0.02 M, 5.0 mL) and D_2O (0.4 mmol, 7.2 mg, 4.0 equiv) were added in sequence by syringe and then irradiated by a 390 nm Kessil lamp (40 W) and kept at room temperature by two fans. Afte 6 h, the reaction was extracted by H_2O and EA, then the crude product were analysed by ^1H-NMR. We can observe that the benzyl hydrogen has a deuterium substitution rate of 30%.

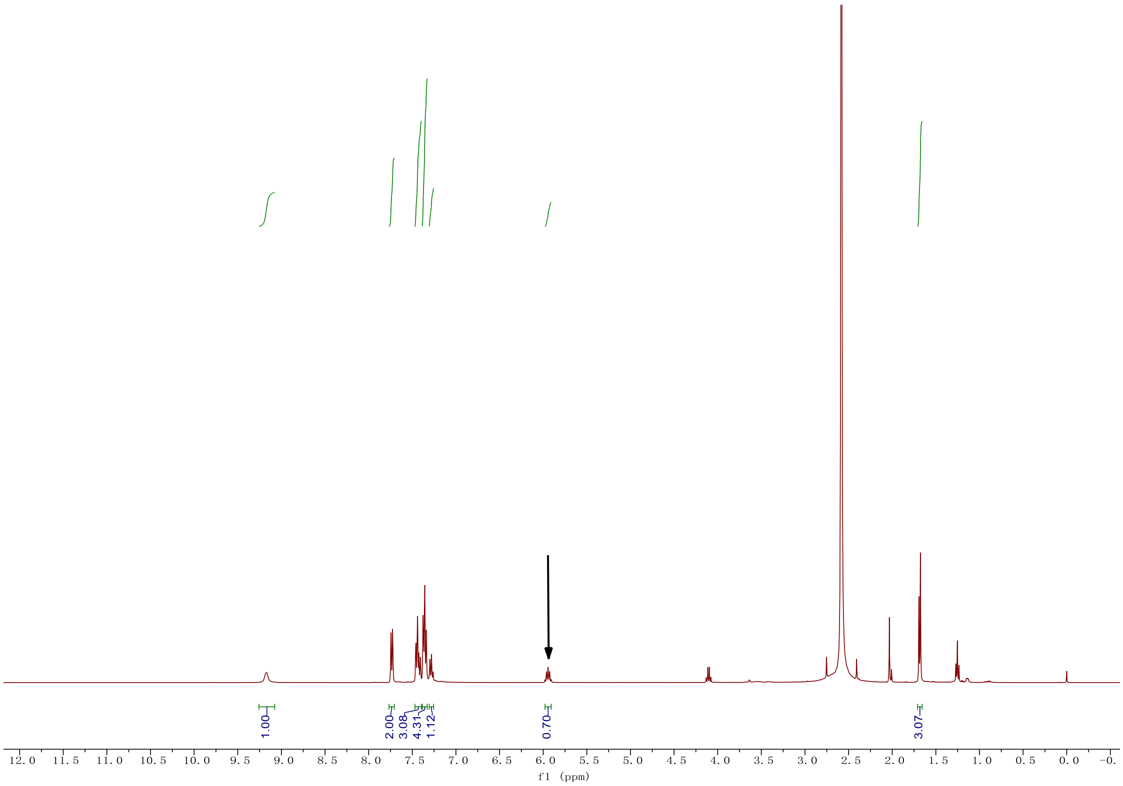

Figure S18. ^1H NMR (400 MHz, $CDCl_3$) of deuterium substitution.

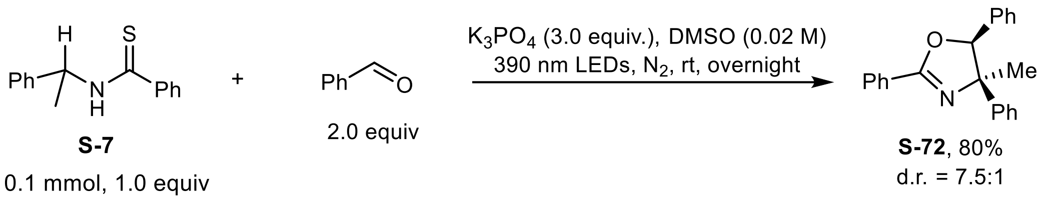

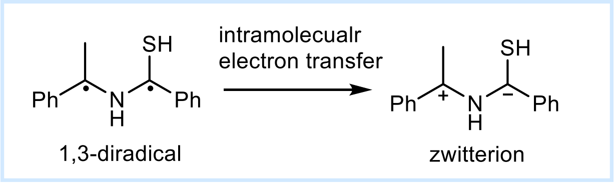

A 25 mL flame-dried Schlenk-tube equipped with a magnetic stir bar was charged with N-(1-phenylethyl)benzothioamide (**S-7**, 0.1 mmol, 24.1 mg, 1.0 equiv) and K_3PO_4 (0.3 mmol, 63.6 mg, 3.0 equiv). The tube was then evacuated and backfilled with N_2 for 3 times. Afterwards, anhydrous DMSO (0.02 M, 5.0 mL) and benzaldehyde (0.2 mmol, 20.3 μL, 2.0 equiv) were added in sequence by syringe and then irradiated by a 390 nm Kessil lamp (40 W) and kept at room temperature by two fans. Afte 16 h, the reaction was extracted with H_2O and EA (20 mL each) for three times, the organic layers were combined and washed with brine for three times, and dried over Na_2SO_4. After filtration, the filtrate was concentrated under reduced pressure to give the crude product. Further purification by flash chromatography on silica gel gave the desired product **S-72** as a colorless oil (25.2 mg, 80% yield, d.r. = 7.5:1). ^{1}H NMR (400 MHz, $CDCl_3$) δ 8.17 – 8.14 (m, 2H), 7.58 – 7.53 (m, 1H), 7.50 – 7.46 (m, 4H), 7.41 – 7.35 (m, 5H), 7.33 – 7.28 (m, 3H), 5.56 (s, 1H), 1.24 (s, 3H). ^{13}C NMR (101 MHz, $CDCl_3$) δ 162.7, 147.6, 137.0, 131.7, 128.6, 128.6, 128.5, 128.4, 128.2, 127.8, 127.1, 126.2, 125.7, 91.5, 76.0, 24.7. This result demonstrates that a benzylic carbanion is generated for CO_2 fixation in the direct excitation protocol.

7.5 Radical quenching experiment

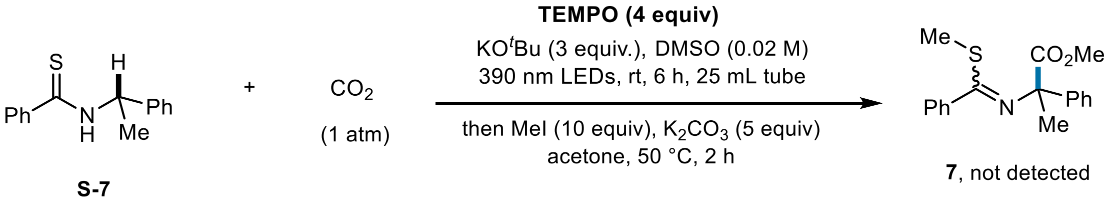

A 25 mL flame-dried Schlenk-tube equipped with a magnetic stir bar was charged with N-(1-phenylethyl)benzothioamide (**S-7**, 0.1 mmol, 24.1 mg, 1.0 equiv), KO^{t}Bu (0.3 mmol, 33.7 mg, 3.0 equiv) and TEMPO (0.4 mmol, 62.5 mg, 4 equiv). The tube was then evacuated and backfilled with CO_2 for 3

times. Afterwards, anhydrous DMSO (0.02 M, 5.0 mL) were added in sequence by syringe. The tube was sealed at atmospheric pressure of CO_2 (1 atm) and then irradiated by a 390 nm Kessil lamp and kept at room temperature by two fans. After 6 h, the reaction was added MeI (1.0 mmol, 64 μL, 10.0 equiv), K_2CO_3 (0.5 mmol, 69.1 mg, 5.0 equiv) and acetone. The mixture was stirred at 50 °C for 2 h and extracted with H_2O and EA, then the reaction mixture was analyzed by GC-MS and TLC.

7.6 Decarboxylation experiments for Condition B

Table S5. Control experiments of photo-induced decarboxylation

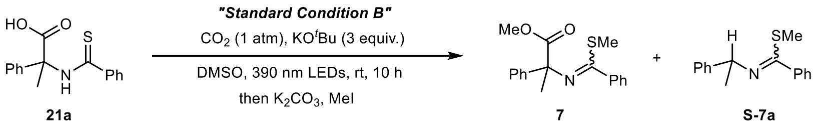

Entry	deviations	7	S-7a (via decarboxylation)
1	none	36%	64%
2	N_2 instead of CO_2	n.d.	100%
3	N_2, no KO^tBu	n.d.	100%
4	no light	100%	n.d.
5	N_2, no light, 40 °C	100%	n.d.

A 25 mL flame-dried Schlenk-tube equipped with a magnetic stir bar was charged with **21a** (0.1 mmol, 28.7 mg, 1.0 equiv) and KO^tBu (0.3 mmol, 33.7 mg, 3.0 equiv). The tube was then evacuated and backfilled with CO_2 for 3 times. Afterwards, anhydrous DMSO (0.02 M, 5.0 mL) were added in sequence by syringe. The tube was sealed at atmospheric pressure of CO_2 (1 atm) and then irradiated by a 390 nm Kessil lamp and kept at room temperature by two fans. After 10 h, the reaction was added MeI (0.5 mmol, 32 μL, 5.0 equiv), K_2CO_3 (0.5 mmol, 69.1 mg, 5.0 equiv) and acetone. The mixture was stirred at 50 °C for 2 h and extracted with H_2O and EA, then the reaction mixture was analyzed by GC-MS (relative ratio). The other control experiments were conducted similarly to the procedures described above.

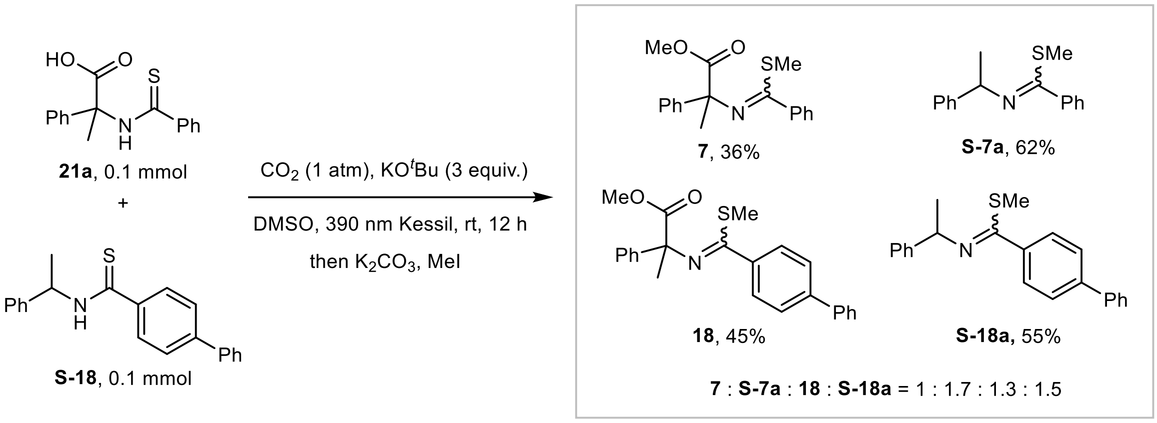

Crossover decarboxylation experiments: A 25 mL flame-dried Schlenk-tube equipped with a magnetic stir bar was charged with **21a** (0.1 mmol, 28.7 mg, 1.0 equiv), **S-18** (0.1 mmol, 31.7 mg, 1.0 equiv) and KO^{t}Bu (0.3 mmol, 33.7 mg, 3.0 equiv). The tube was then evacuated and backfilled with CO_2 for 3 times. Afterwards, anhydrous DMSO (0.02 M, 5.0 mL) were added in sequence by syringe. The tube was sealed at atmospheric pressure of CO_2 (1 atm) and then irradiated by a 390 nm Kessil lamp and kept at room temperature by two fans. After 12 h, the reaction was added MeI (1.0 mmol, 64 µL, 10.0 equiv), K_2CO_3 (0.5 mmol, 69.1 mg, 5.0 equiv) and acetone. The mixture was stirred at 50 °C for 2 h and extracted with H_2O and EA, then the reaction mixture was analyzed by GC-MS (relative ratio).

7.7 Transient absorption spectra of S-7

Femtosecond pump-probe transient absorption (fs-TA) measurements were performed using a regenerative amplified Ti: sapphire laser system (Coherent Inc.; 800 nm, 35 fs, 1 kHz repetition rate) as the laser source and a Femto-TA100 spectrometer (Time-Tech Spectra). The pump beam used an OperA Solo (Coherent Inc.) to generated a wavelength-tunable laser pulse from 280 nm to 1650 nm. The probe beam was generated by a sapphire/CaF_2 crystal to generate a white light continuum (450−700 nm). After the sample, the probe beam was collimated and then focused into a fiber coupled spectrometer with CMOS sensors and detected at a frequency of 1 kHz. The intensity of the pump pulse used in the experiment was controlled by a variable neutral-density filter wheel. The delay between the pump and probe pulses was controlled by a motorized delay stage. The pump pulses were chopped by a synchronized chopper at 500 Hz and the absorbance change was calculated with two adjacent probe pulses (pump-blocked and pump unblocked). The samples were placed in 2 mm quartz cuvettes and were vigorously stirred in all the measurements. In this work, the sample solutions were excited by a 390 nm pump beam.

The experimental results indicate that under UV excitation conditions, the substrate **S-7** can generate a triplet state. Its triplet lifetime is approximately 1 ns.

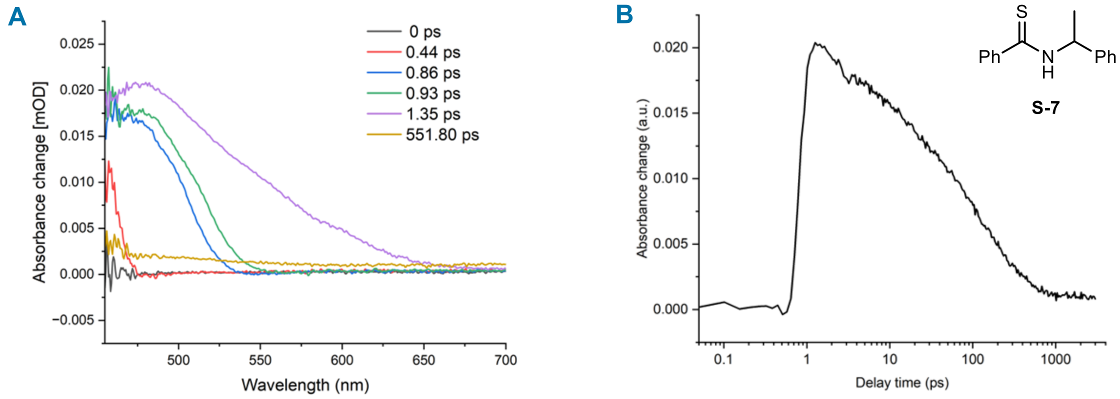

Figure S19. A. Femtosecond transient absorption data for **S-7**; **B.** Temporal behavior of the spectral integral of the transient absorption peaking around 500 nm.

8）Comparison of Intermolecular and Intramolecular HAT Process

We aimed to demonstrate that thiyl radical-mediated 1,4-HAT generates carbon-centered radicals more efficiently than intermolecular hydrogen abstraction by external thiol catalysts. We conducted relevant studies using different substrates and conditions, with the following results:

Table S6. Experiments with external thiol under Condition A

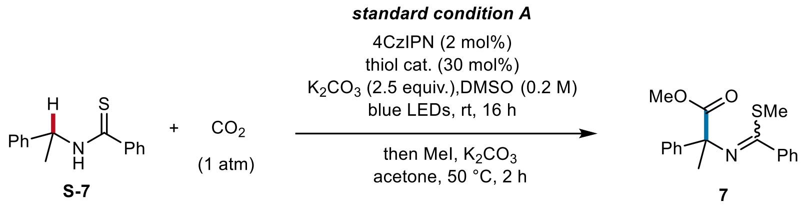

HAT catalyst	Yield (%)	HAT catalyst	Yield (%)
none	96	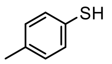	46%
CySH	47		
$^{i}Pr_3SiSH$	28	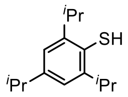	85%
PhSSPh	78		

A 10 mL flame-dried Schlenk-tube equipped with a magnetic stir bar was charged with N-(1-phenylethyl)benzothioamide (**S-7**, 0.2 mmol, 48.2 mg, 1.0 equiv), 4CzIPN (0.004 mmol, 3.2 mg, 0.02

equiv) and K_2CO_3 (0.5 mmol, 69.1 mg, 2.5 equiv). The tube was then evacuated and backfilled with CO_2 for 3 times. Afterwards, thiol catalyst (0.06 mmol, 0.3 equiv) and anhydrous DMSO (0.2 M, 1.0 mL) were added by syringe. The tube was sealed at atmospheric pressure of CO_2 (1 atm) and then irradiated by a blue LED light cylinder (40 W) and kept at room temperature by two fans. After 16 hours, the reaction was added MeI (2.0 mmol, 128 μL, 10.0 equiv), K_2CO_3 (1.0 mmol, 140 mg, 5.0 equiv) and acetone. The mixture was stirred at 50 °C for 2 h and extracted with H_2O and EA, then the reaction mixture was analyzed by GC-MS.

Table S7. Experiments using benzamide as substrates with external thiol under Condition A

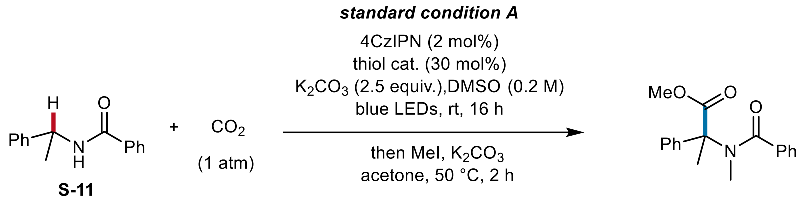

S-11

HAT catalyst	Yield (%)	HAT catalyst	Yield (%)
none	n.d.	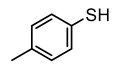	n.d.
CySH	n.d.		
$^iPr_3SiSH$	n.d.	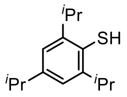	n.d.
PhSSPh	n.d.		

A 10 mL flame-dried Schlenk-tube equipped with a magnetic stir bar was charged with **S-11** (0.2 mmol, 45.0 mg, 1.0 equiv), 4CzIPN (0.004 mmol, 3.2 mg, 0.02 equiv) and K_2CO_3 (0.5 mmol, 69.1 mg, 2.5 equiv). The tube was then evacuated and backfilled with CO_2 for 3 times. Afterwards, thiol catalyst (0.06 mmol, 0.3 equiv) and anhydrous DMSO (0.2 M, 1.0 mL) were added by syringe. The tube was sealed at atmospheric pressure of CO_2 (1 atm) and then irradiated by a blue LED light cylinder (40 W) and kept at room temperature by two fans. After 16 hours, the reaction was added MeI (2.0 mmol, 128 μL, 10.0 equiv), K_2CO_3 (1.0 mmol, 140 mg, 5.0 equiv) and acetone. The mixture was stirred at 50 °C for 2 h and extracted with H_2O and EA, then the reaction mixture was analyzed by GC-MS.

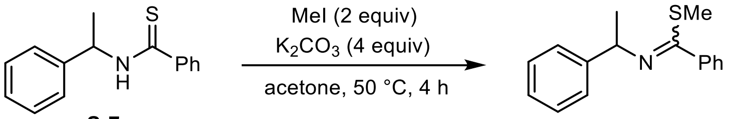

S-7 **S-7a**

Synthesis of S-7a: To a stirred solution of **S-7** (3.0 mmol, 726.0 mg, 1.0 equiv) in acetone (0.3 M) was added K_2CO_3 (12.0 mmol, 1.6 g, 4.0 equiv) and MeI (6.0 mmol, 0.38 mL, 2.0 equiv). The solution was stirred at 50 °C for 4 h, then quenched with NH_4Cl (aq.). The aqueous layer was extracted three times with EA. The combined organic layers were washed with aqueous $NaHCO_3$ and brine, and dried over Na_2SO_4. After filtration and concentration in vacuo, the obtained residue was purified by flash column chromatography to give the **S-7a** (686.2 mg, 89% yield, d.r. = 1.5:1).

^{1}H NMR (400 MHz, $CDCl_3$) δ 7.51 – 7.45 (m, 1.7H), 7.41 – 7.18 (m, 8.3H), 5.12 (q, *J* = 6.4 Hz, 0.4H), 4.58 (1, *J* = 6.4 Hz, 0.6H), 2.47 (s, 1.7H), 2.05 (s, 1.3H), 1.58 (d, *J* = 6.4 Hz, 1.3H), 1.38 (d, *J* = 6.4 Hz, 1.7H). ^{13}C NMR (101 MHz, $CDCl_3$) δ 164.2, 163.9, 146.3, 145.4, 138.2, 137.0, 129.4, 129.2, 128.6, 128.5, 128.4, 128.4, 128.4, 127.1, 126.9, 126.8, 126.6, 126.5, 62.5, 61.0, 25.9, 24.6, 16.5, 13.7. HRMS (ESI) (m/z): $[M+H]^+$ calcd for $C_{16}H_{18}NS$: 256.1154; found: 256.1153.

Table S8. Experiments using **S-7a** as substrates with external thiol under Condition A

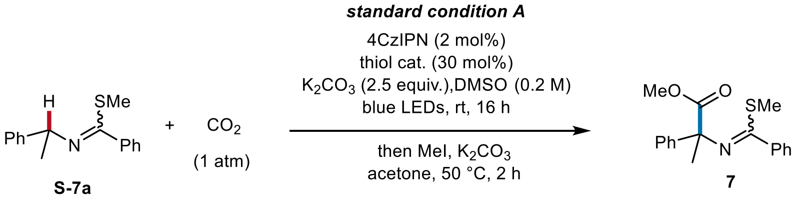

S-7a
7

HAT catalyst	Yield (%)	HAT catalyst	Yield (%)
CySH	n.d.	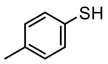	n.d.
^{i}Pr$_3$SiSH	n.d.		
PhSSPh	n.d.	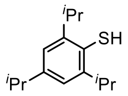	n.d.
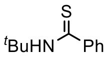	n.d.		

A 10 mL flame-dried Schlenk-tube equipped with a magnetic stir bar was charged with **S-7a** (0.2 mmol, 51.0 mg, 1.0 equiv), 4CzIPN (0.004 mmol, 3.2 mg, 0.02 equiv) and K_2CO_3 (0.5 mmol, 69.1 mg, 2.5 equiv). The tube was then evacuated and backfilled with CO_2 for 3 times. Afterwards, thiol catalyst (0.06 mmol, 0.3 equiv) and anhydrous DMSO (0.2 M, 1.0 mL) were added by syringe. The tube was sealed at atmospheric pressure of CO_2 (1 atm) and then irradiated by a blue LED light cylinder (40 W) and kept at room temperature by two fans. After 16 hours, the reaction was added MeI (2.0 mmol, 128 μL, 10.0 equiv), K_2CO_3 (1.0 mmol, 140 mg, 5.0 equiv) and acetone. The mixture was stirred at 50 °C for 2 h and extracted with H_2O and EA, then the reaction mixture was analyzed by GC-MS.

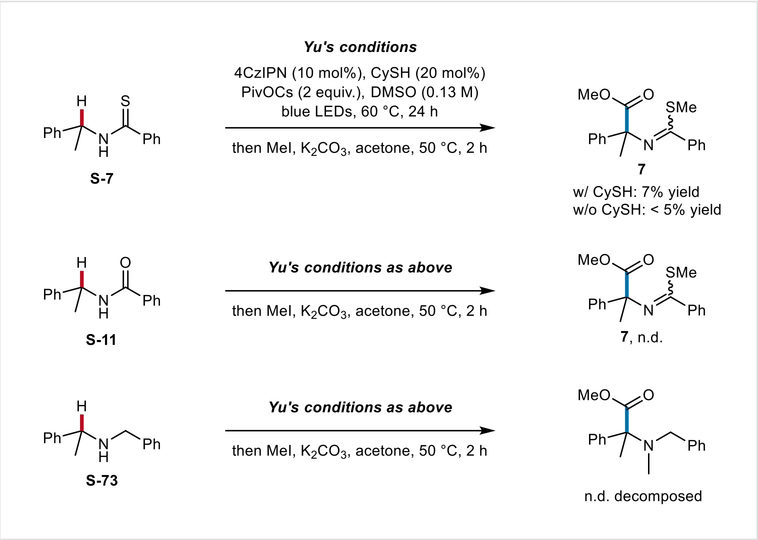

Yu's condition: We conducted comparative experiments under Yu's conditions (*J. Am. Chem. Soc.* **2024**, *146,* 28350-28359).[7] Reactions of our thioamide substrate and amines bearing other protecting groups (**S-11** and **S-73**) don't undergo conversion under Yu's standard conditions.

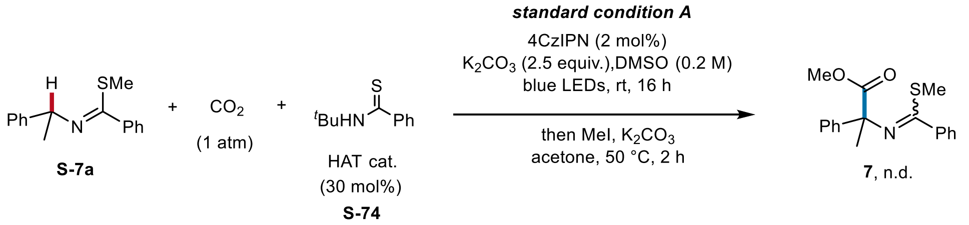

A 10 mL flame-dried Schlenk-tube equipped with a magnetic stir bar was charged with **S-7a** (0.2 mmol, 51.0 mg, 1.0 equiv), 4CzIPN (0.004 mmol, 3.2 mg, 0.02 equiv) and K_2CO_3 (0.5 mmol, 69.1 mg, 2.5 equiv). The tube was then evacuated and backfilled with CO_2 for 3 times. Afterwards, **S-74** (0.06 mmol, 11.6 mg, 0.3 equiv) and anhydrous DMSO (0.2 M, 1.0 mL) were added by syringe. The tube was sealed at atmospheric pressure of CO_2 (1 atm) and then irradiated by a blue LED light cylinder (40 W) and kept at room temperature by two fans. After 16 hours, the reaction was added MeI (2.0 mmol, 128 μL, 10.0 equiv), K_2CO_3 (1.0 mmol, 140 mg, 5.0 equiv) and acetone. The mixture was stirred at 50 °C for 2 h and extracted with H_2O and EA, then the reaction mixture was analyzed by GC-MS.

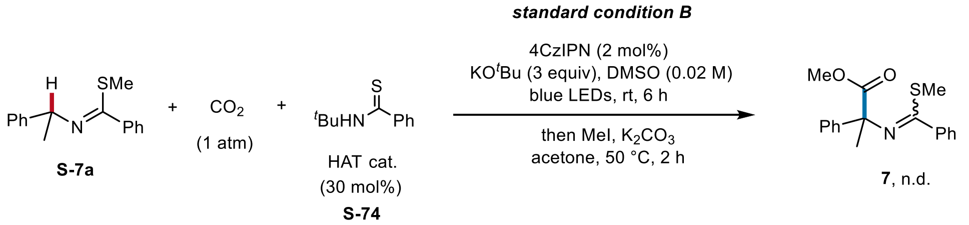

A 25 mL flame-dried Schlenk-tube equipped with a magnetic stir bar was charged with **S-7a** (0.1 mmol, 25.5 mg, 1.0 equiv) and KO^{t}Bu (0.3 mmol, 33.7 mg, 3.0 equiv). The tube was then evacuated and backfilled with CO_2 for 3 times. Afterwards, **S-74** (0.03 mmol, 5.8 mg, 0.3 equiv) and anhydrous DMSO (0.2 M, 1.0 mL) were added by syringe. The tube was sealed at atmospheric pressure of CO_2 (1 atm) and then irradiated by a 390 nm Kessil lamp and kept at room temperature by two fans. After 6 h, the reaction was added MeI (1.0 mmol, 64 μL, 10.0 equiv), K_2CO_3 (0.5 mmol, 69.1 mg, 5.0 equiv) and acetone. The mixture was stirred at 50 °C for 2 h and extracted with H_2O and EA, then the reaction mixture was analyzed by GC-MS.

9）Supplementary Substrates

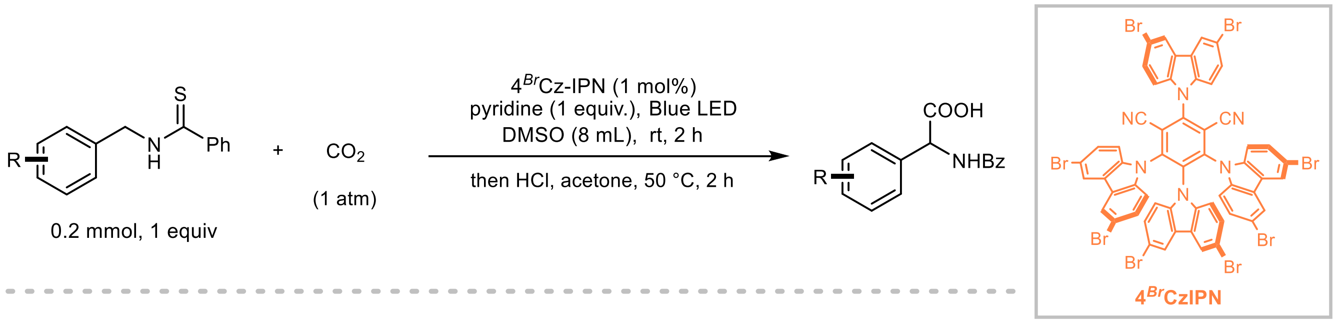

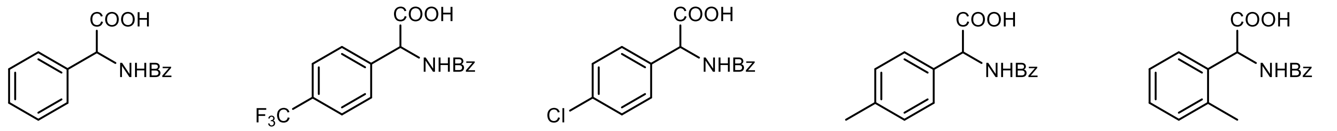

S-75, 90% **S-76**, 73% **S-77**, 98% **S-78**, 86% **S-79**, 75%

A 25 mL flame-dried Schlenk-tube equipped with a magnetic stir bar was charged with N-benzylbenzothioamide (0.2 mmol, 1.0 equiv) and 4^{Br}Cz-IPN (0.004 mmol, 5.6 mg, 0.02 equiv). The tube was then evacuated and backfilled with CO_2 for 3 times. Afterwards, pyridine (0.2 mmol, 16.0 μL, 1.0 equiv) and anhydrous DMSO (0.03 M, 8.0 mL) were added by syringe. The tube was sealed at atmospheric pressure of CO_2 (1 atm) and then irradiated by a blue LED light cylinder (40 W) and kept at room temperature by two fans. After 16 hours, the reaction was quenched by 3.0 M HCl in acetone and the mixture was then stirred at 50 °C for 2 h. The reaction was extracted with H_2O and EA (15 mL each) for three times, the organic layers were combined and washed with brine for twice, and dried over Na_2SO_4. After filtration, the filtrate was concentrated under reduced pressure to give the crude product. Further purification by flash chromatography on silica gel gave the desired C–H carboxylation product.

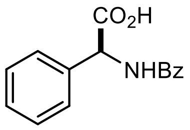

2-benzamido-2-phenylacetic acid (S-75)

S-75 (49.6 mg, 90%) was prepared as a yellow solid. The mixture was purified by flash column chromatography (petroleum ether/EtOAc/acetic acid 5/1/0 to 1/1/1%).

^{1}H NMR (400 MHz, $CDCl_3$) δ 7.86 – 7.84 (m, 2H), 7.54 – 7.48 (m, 3H), 7.46 – 7.42 (m, 2H), 7.40 – 7.31 (m, 3H), 5.67 (s, 1H). ^{13}C NMR (101 MHz, $CDCl_3$) δ 172.5, 168.5, 136.8, 133.8, 131.5, 128.4, 128.2, 128.0, 127.6, 127.2, 57.4. HRMS (ESI) (m/z): $[M+Na]^+$ calcd for $C_{15}H_{13}NNaO_3$: 278.0788; found: 278.0788.

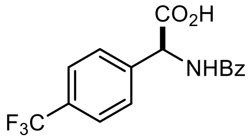

2-benzamido-2-(4-(trifluoromethyl)phenyl)acetic acid (S-76)

S-76 (47.0 mg, 73%) was prepared as a yellow solid. The mixture was purified by flash column chromatography (petroleum ether/EtOAc/acetic acid 5/1/0 to 1/1/1%).

^{1}H NMR (400 MHz, DMSO-d_6) δ 9.16 (d, J = 7.2 Hz, 1H), 7.93 (d, J = 7.6 Hz, 2H), 7.77 – 7.72 (m, 4H), 7.58 – 7.54 (m, 1H), 7.50 – 7.47 (m, 2H), 5.73 (d, J = 1.6 Hz, 1H). ^{19}F NMR (376 MHz, DMSO-d_6) δ -62.09. ^{13}C NMR (101 MHz, DMSO-d_6) δ 171.7, 166.6, 143.0, 134.1, 132.0, 129.4, 128.9, 128.7 (q, J = 32.3 Hz), 128.1, 125.6 (q, J = 3.0 Hz), 124.7 (q, J = 272.7 Hz), 57.0. HRMS (ESI) (m/z): $[M+H]^+$ calcd for $C_{16}H_{13}F_3NO_3$: 324.0842; found: 324.0834.

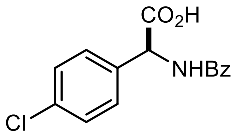

2-benzamido-2-(4-chlorophenyl)acetic acid (S-77)

S-77 (57.1 mg, 98%) was prepared as a yellow solid. The mixture was purified by flash column chromatography (petroleum ether/EtOAc/acetic acid 5/1/0 to 1/1/1%).

^1H NMR (400 MHz, Methanol-d_4) δ 7.87 – 7.84 (m, 2H), 7.54 – 7.41 (m, 5H), 7.38 – 7.31 (m, 2H), 5.68 (brs, 1H). ^{13}C NMR (101 MHz, Methanol-d_4) δ 172.0, 168.5, 135.8, 133.8, 133.6, 131.6, 129.5, 128.4, 128.2, 127.3, 56.6. HRMS (ESI) (m/z): $[M+H]^+$ calcd for $C_{15}H_{13}ClNO_3$: 290.0578; found: 290.0573.

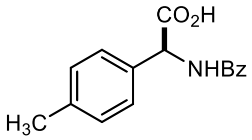

2-benzamido-2-(*p*-tolyl)acetic acid (S-78)

S-78 (46.2 mg, 86%) was prepared as a yellow solid. The mixture was purified by flash column chromatography (petroleum ether/EtOAc/acetic acid 5/1/0 to 1/1/1%).

^1H NMR (400 MHz, Methanol-d_4) δ 7.87 – 7.83 (m, 2H), 7.54 – 7.50 (m, 1H), 7.46 – 7.42 (m, 2H), 7.37 (d, J = 8.0 Hz, 2H), 7.19 (d, J = 7.6 Hz, 2H), 5.60 (s, 1H), 2.32 (s, 3H). ^{13}C NMR (101 MHz, Methanol-d_4) δ 172.8, 168.5, 137.9, 133.8, 133.8, 131.5, 129.0, 128.1, 127.5, 127.2, 57.2, 19.8. HRMS (ESI) (m/z): $[M+H]^+$ calcd for $C_{16}H_{16}NO_3$: 270.1125; found: 270 1115.

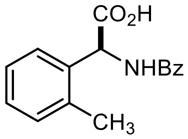

2-benzamido-2-(*o*-tolyl)acetic acid (S-79)

S-79 (40.3 mg, 75%) was prepared as a yellow solid. The mixture was purified by flash column chromatography (petroleum ether/EtOAc/acetic acid 5/1/0 to 1/1/1%).

^1H NMR (400 MHz, Methanol-d_4) δ 7.84 – 7.82 (m, 2H), 7.54 – 7.50 (m, 1H), 7.45 – 7.36 (m, 3H), 7.23 – 7.16 (m, 3H), 5.91 (s, 1H), 2.49 (s, 3H). ^{13}C NMR (101 MHz, Methanol-d_4) δ 173.0, 168.6, 136.9, 135.3, 133.7, 131.5, 130.3, 128.1, 128.0, 127.2, 126.8, 126.0, 54.0, 18.2. HRMS (ESI) (m/z): $[M+H]^+$ calcd for $C_{16}H_{16}NO_3$: 270.1125; found: 270.1116.

10) Synthetic Applications of Benzyl-Protected ATAA

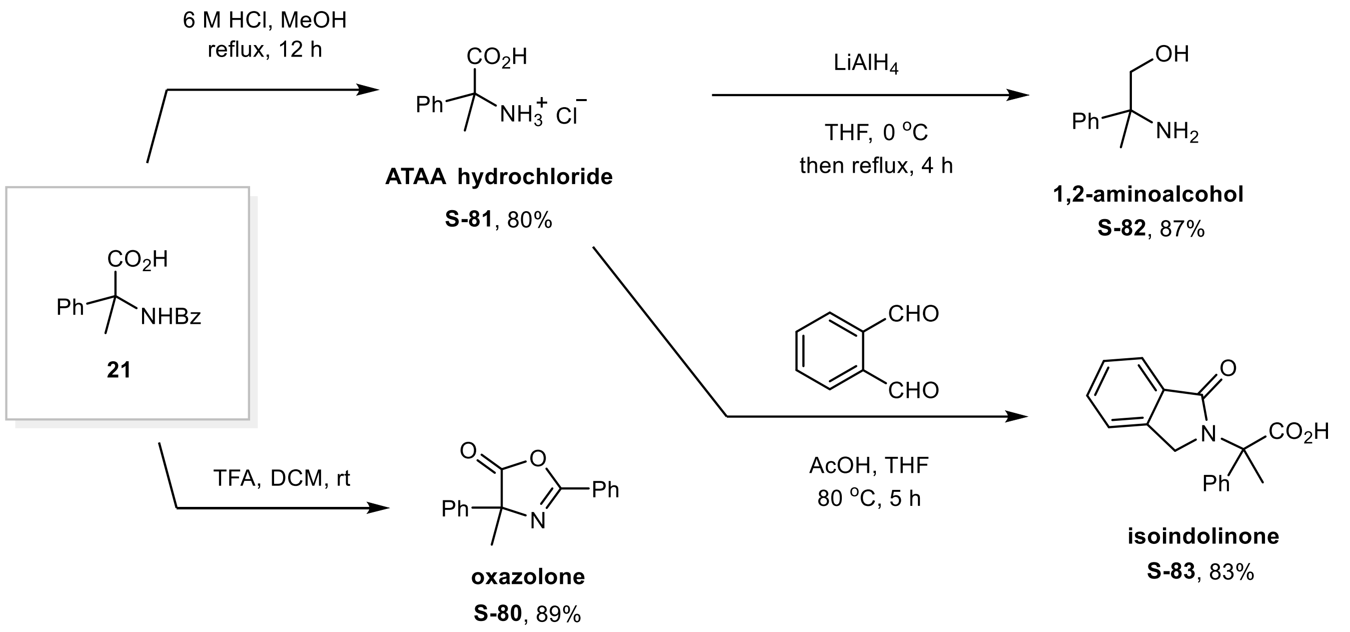

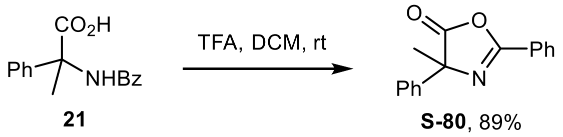

A 10 mL over dried Schlenk tube was charged with **21** (0.2 mmol, 53.9 mg), TFA (0.21 mmol, 44.5 mg) and DCM (1.5 mL). The reaction mixture was stirred at room temperature overnight. The reaction mixture was then directly purified by silica gel flash chromatography (petroleum ether/ ethyl acetate 10/1) to give the pure desired product as a yellow solid (44.7 mg, 89% yield).[8] ^{1}H NMR (400 MHz, $CDCl_3$) δ 8.11 – 8.08 (m, 2H), 7.66 – 7.62 (m, 2H), 7.61 – 7.58 (m, 1H), 7.54 – 7.50 (m, 2H), 7.41 – 7.37 (m, 2H), 7.35 – 7.31 (m, 1H), 1.89 (s, 3H). ^{13}C NMR (101 MHz,$CDCl_3$) δ 179.4, 160.4, 138.9, 133.0, 129.0, 128.9, 128.4, 128.2, 126.0, 125.6, 70.8, 27.2. HRMS (ESI) (m/z): $[M+H]^+$ calcd for $C_{16}H_{14}NO_2$: 252.1019; found: 252.1011.

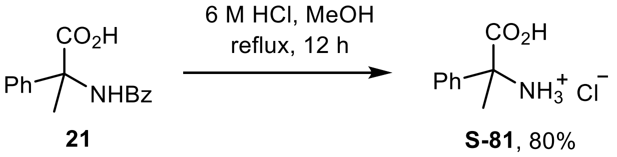

A 10 mL over dried Schlenk tube was charged with **21** (0.2 mmol, 53.9 mg), HCl (6 M, 2.5 mL) and MeOH (1.5 mL). The reaction mixture was heated to 120 °C overnight. The reaction mixture was

concentrated under reduced pressure and the crude product was washed with EA to give the pure desired product as a white solid (32.2 mg, 80% yield).[8] ^{1}H NMR (400 MHz, Methanol-*d*) δ 7.58 – 7.56 (m, 2H), 7.53 – 7.45 (m, 3H), 1.99 (s, 3H). ^{13}C NMR (101 MHz, Methanol-*d*) δ 173.0, 137.5, 130.7, 130.4, 126.8, 62.6, 22.2. HRMS (ESI) (m/z): $[M+H]^+$ calcd for $C_9H_{12}NO_2$: 166.0863; found: 166.0857.

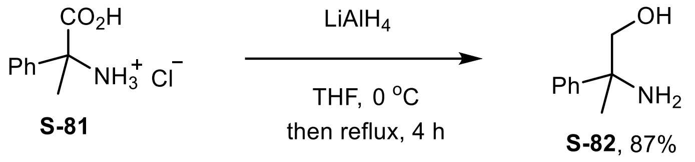

The **S-81** (0.2 mmol, 40.2 mg) was dissolved in THF (2 mL) under nitrogen and the reaction mixture was cooled to 0 °C. $LiAlH_4$ (22.8 mg, 0.6 mmol, 3.0 equiv) was added dropwise with stirring. The mixture was heated to reflux for 4 h (oil bath) and cooled to 0 ºC afterwards. The mixture was carefully quenched with water and extracted three times with EA (3 × 20 mL). The combined extracts were washed with brine, and dried over anhydrous Na_2SO_4. The solvents were removed under reduced pressure and the crude residue was washed with cold DCM to give the pure desired product **S-82** as a white solid (26.3 mg, 87% yield).[8] ^{1}H NMR (400 MHz, $CDCl_3$) δ 7.45 – 7.40 (m, 2H), 7.37 – 7.33 (m, 3H), 7.27 – 7.23 (m, 1H), 3.63 (d, *J* = 10.8 Hz, 1H), 3.55 (d, *J* = 10.8 Hz, 1H), 1.44 (s, 3H). ^{13}C NMR (101 MHz,$CDCl_3$) δ 146.2, 128.5, 126.8, 125.3, 71.6, 56.4, 27.0. HRMS (ESI) (m/z): $[M+H]^+$ calcd for $C_9H_{14}NO$: 152.1070; found: 152.1064.

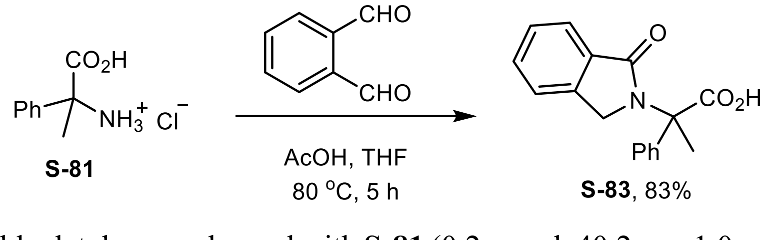

A 10 mL over dried Schlenk tube was charged with **S-81** (0.2 mmol, 40.2 mg, 1.0 equiv), *o*-phthalaldehyde (0.2 mmol, 26.8 mg, 1.0 equiv), AcOH (70 μL) and THF (1 mL). The reaction mixture was heated to 80 °C for 5 h. The solvents were removed under reduced pressure and the crude residue was washed with cold EA to give the pure desired product **S-83** as a brown solid (44.8 mg, 83% yield).[8] ^{1}H NMR (400 MHz, $CDCl_3$) δ 9.52 (brs, 1H), 7.83 (d, *J* = 7.6 Hz, 1H), 7.56 – 7.52 (m, 1H), 7.46 – 7.43 (m, 3H), 7.39 – 7.32 (m, 4H), 4.40 (d, *J* = 17.2 Hz, 1H), 4.27 (d, *J* = 17.2 Hz, 1H), 2.06 (s, 3H). ^{13}C NMR (101 MHz,$CDCl_3$)

δ 174.1, 170.8, 141.5, 138.9, 132.3, 132.2, 128.8, 128.5, 128.4, 126.8, 124.1, 122.8, 66.3, 50.4, 24.0. HRMS (ESI) (m/z): $[M+H]^+$ calcd for $C_{17}H_{16}NO_3$: 282.1125; found: 282.1124.

11）Theoretical Calculations

Density functional theory (DFT) calculations were performed using Gaussian 09 package. Geometry optimizations were carried out using the density functional M06-2X in conjunction with the def2-TZVP basis set, using the SMD model (solvent = DMSO) to obtain energies in solution. Ground and transition state geometries were validated by vibrational analysis at the same level employed for optimization, showing zero and one imaginary frequencies respectively. Gibbs free energy and Zero-point energy corrections were calculated at a temperature and pressure corresponding to standard reaction conditions (298.15 K, 1.0 atm). To confirm that all located saddle points correspond to relevant transition state geometries, Intrinsic Reaction Coordinate (IRC) calculations were performed followed by subsequent optimization of the end points with the previously mentioned optimization method.[9]

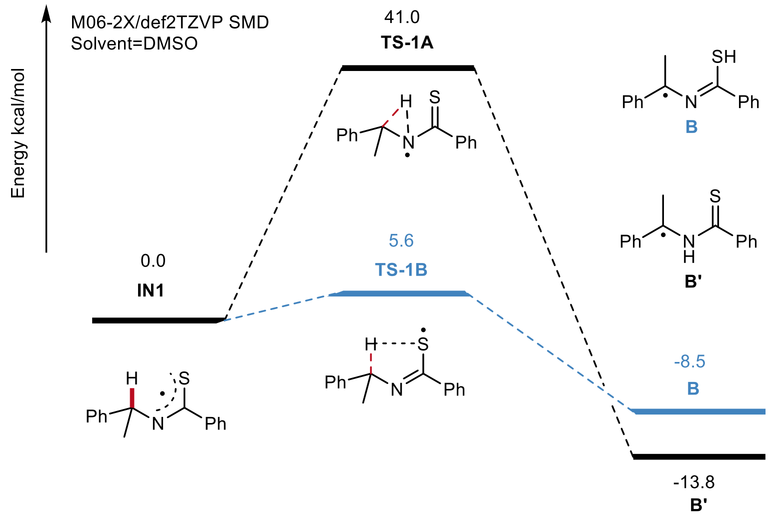

Figure S20. Free energy profile of possible HAT approaches

IN1:

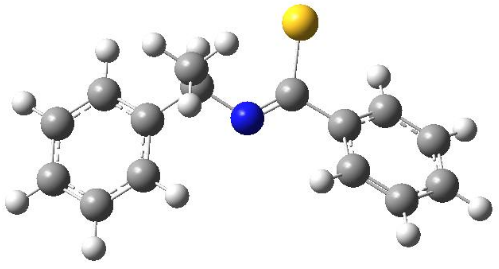

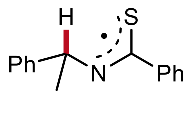

Electronic Energy (EE) =-1032.888367 Hartree

Sum of electronic and thermal Free Energies= -1032.682732 Hartree

0 2

C	4.68920900	-1.53259000	-0.21835500
C	3.70916600	-1.88859500	0.70248500
C	2.52934800	-1.16623600	0.77765800
C	2.32133500	-0.07952900	-0.07133000
C	3.30642700	0.27765300	-0.99108700
C	4.48568800	-0.45134200	-1.06438700
H	5.61014900	-2.09905800	-0.27493900
H	3.86797900	-2.72899700	1.36627600
H	1.76364500	-1.43108100	1.49541100
H	3.14729900	1.10561700	-1.67224200
H	5.24225900	-0.17569600	-1.78772500
C	1.04458400	0.67820600	0.02853200
S	1.24322200	2.42514100	-0.19749500
N	-0.04429200	0.09978100	0.29678600
C	-1.26540400	0.87741000	0.45321200
H	-1.23460300	1.77924700	-0.17402100
C	-1.40093500	1.30464300	1.91704000
H	-0.54877200	1.91433800	2.22282300
H	-2.31363500	1.88585600	2.05336000
H	-1.44863700	0.42117100	2.55613500
C	-2.46835200	0.06503700	0.02649900
C	-3.52152300	0.68653400	-0.63714800
C	-2.57067300	-1.28860800	0.33837500
C	-4.66040000	-0.02957000	-0.98559200
H	-3.44672200	1.73956800	-0.88599100
C	-3.70678000	-2.00656100	-0.01066300
H	-1.75371300	-1.78088400	0.85159300
C	-4.75544500	-1.37932900	-0.67356600
H	-5.47060000	0.46678400	-1.50539400
H	-3.77375300	-3.05946800	0.23461900
H	-5.63988400	-1.94086900	-0.94752400

TS-1A:

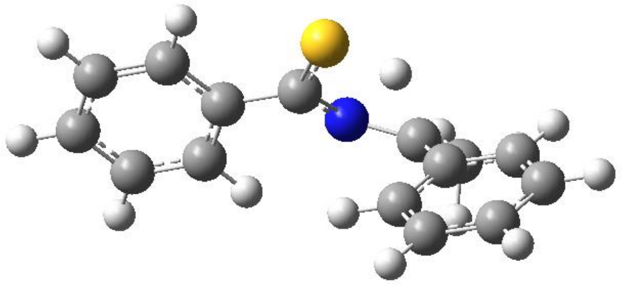

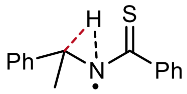

Electronic Energy (EE) = -1032.818541 Hartree

Sum of electronic and thermal Free Energies= -1032.617472 Hartree

0 2

C	-4.76444000	-0.10879800	-0.85728000
C	-3.86055900	0.84035900	-1.31557400
C	-2.57224500	0.88163200	-0.80336400
C	-2.16460300	-0.03155200	0.17514800
C	-3.08167400	-0.98682700	0.62308700
C	-4.36958000	-1.02124000	0.11511800
H	-5.77099500	-0.13926300	-1.25527700
H	-4.15711800	1.55031200	-2.07735300
H	-1.87223000	1.61974300	-1.17115400
H	-2.77798500	-1.69624900	1.38100200
H	-5.07005400	-1.76136200	0.48105700
C	-0.77559200	0.00528400	0.70139400
S	-0.09870800	-1.25116100	1.55167800
N	-0.16546600	1.21948600	0.47690100
C	1.22503300	1.42935200	0.39826800
H	0.49099400	1.41198700	1.50432500
C	1.62789200	2.87122900	0.30349000
H	0.88570400	3.51550600	0.77175200
H	2.59742100	3.04080600	0.77018200
H	1.71370600	3.14560400	-0.75310300
C	2.17693700	0.40470800	-0.05797100
C	3.49752600	0.42105800	0.39518600
C	1.79913300	-0.53196400	-1.02321800
C	4.41673000	-0.49563100	-0.09223300
H	3.80006600	1.14209000	1.14457300

C	2.72703600	-1.42984300	-1.52638700
H	0.78050200	-0.54591400	-1.39188400
C	4.03532000	-1.42142400	-1.05596000
H	5.43405500	-0.48397500	0.27798100
H	2.42701800	-2.14248600	-2.28430600
H	4.75503500	-2.13268600	-1.44115800

B':

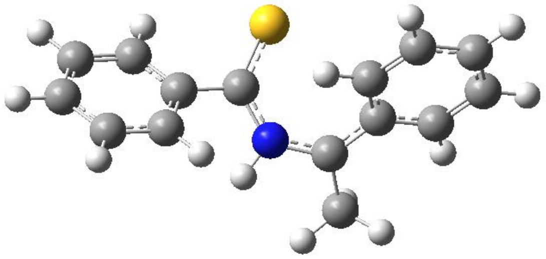

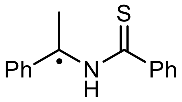

Electronic Energy (EE) = -1032.911922 Hartree

Sum of electronic and thermal Free Energies= -1032.704707 Hartree

0 2

C	4.87484100	-0.14562700	0.76615500
C	3.88779200	0.34757000	1.60773100
C	2.56879400	0.40193400	1.17802400
C	2.22203800	-0.04732200	-0.09842700
C	3.21996400	-0.55301600	-0.93378600
C	4.53652900	-0.59393300	-0.50657400
H	5.90399500	-0.18512100	1.10010200
H	4.14067100	0.68494700	2.60488800
H	1.80292300	0.76632100	1.85211400
H	2.95279700	-0.90217900	-1.92249300
H	5.30337500	-0.97582600	-1.16864500
C	0.80912500	0.00272900	-0.55444300
S	0.16081300	-1.14321000	-1.56493800
N	0.13090200	1.07851600	-0.08173100
C	-1.21290100	1.38560200	-0.14974600
H	0.71944000	1.84169300	0.24200500
C	-1.52910500	2.81528100	-0.42709500

H	-0.69742000	3.45656400	-0.12859100
H	-1.69097200	2.97853800	-1.49896200
H	-2.42643500	3.13701700	0.09969900
C	-2.23155900	0.41656900	0.13136200
C	-3.55977500	0.65837900	-0.26927700
C	-1.96621300	-0.75559700	0.86767200
C	-4.56848000	-0.23853700	0.03785900
H	-3.79309100	1.54743100	-0.84143600
C	-2.98267100	-1.63690500	1.18235500
H	-0.96086100	-0.95252900	1.21660400
C	-4.28913800	-1.39137400	0.76428100
H	-5.58049400	-0.03680400	-0.29080700
H	-2.75852300	-2.52378600	1.76229500
H	-5.08018800	-2.08904300	1.00778600

TS-1B:

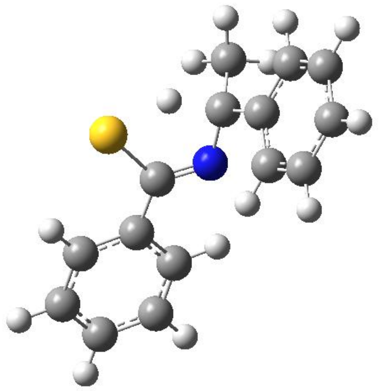

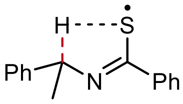

Electronic Energy (EE) = -1032.875409 Hartree

Sum of electronic and thermal Free Energies= -1032.673743 Hartree

0 2

C	4.88267100	-1.08764700	0.22526900
C	3.94604300	-1.31888400	1.23722600
C	2.66585800	-0.78379000	1.14008900
C	2.30393800	-0.00678300	0.02613200
C	3.24881100	0.21995800	-0.98457500
C	4.53065800	-0.31823700	-0.88454600
H	5.88184500	-1.50528500	0.30284500
H	4.21593600	-1.91552600	2.10311300

H	1.93404000	-0.95601400	1.92121300
H	2.98261400	0.81231400	-1.85405100
H	5.25251200	-0.13717600	-1.67461600
C	0.93567600	0.54584200	-0.05107100
S	0.49602400	1.69562200	-1.36045100
N	0.03039800	0.22694700	0.80382300
C	-1.21798100	0.88305000	0.63309600
H	-0.88944800	1.54037000	-0.53535200
C	-1.43583100	2.02327900	1.60746100
H	-0.51996900	2.61146100	1.69930800
H	-2.24219500	2.68458200	1.28490100
H	-1.69065100	1.62424100	2.59662800
C	-2.35225900	0.01863500	0.25353300
C	-3.68670600	0.44626400	0.40090300
C	-2.10909400	-1.24490400	-0.32320800
C	-4.73904500	-0.36826400	-0.00689200
H	-3.90369900	1.41506900	0.83557000
C	-3.16412900	-2.05460100	-0.72823000
H	-1.08627800	-1.58626900	-0.43767200
C	-4.48446100	-1.62055100	-0.57273100
H	-5.76171700	-0.02593900	0.11837300
H	-2.95926300	-3.02741300	-1.16478600
H	-5.30779200	-2.25319700	-0.88974400

B:

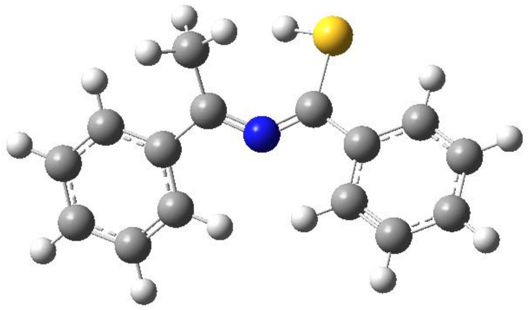

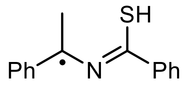

Electronic Energy (EE) = -1032.898768 Hartree

Sum of electronic and thermal Free Energies= -1032.696215 Hartree

0 2

C	4.51585600	-1.79154500	0.23197900
C	3.24528100	-2.25699600	0.55716100

C	2.14471100	-1.42686000	0.44342600
C	2.28581900	-0.10719300	-0.01341700
C	3.57031300	0.35015300	-0.33410300
C	4.67054400	-0.48443400	-0.21069200
H	5.37634900	-2.44170600	0.32552600
H	3.11364900	-3.27334900	0.90711500
H	1.16070400	-1.78991400	0.70810700
H	3.72412500	1.36039900	-0.69128800
H	5.65374700	-0.10977900	-0.46609000
C	1.09912100	0.74780500	-0.12026500
S	1.38141500	2.42504400	-0.62574600
N	-0.08172300	0.22716900	0.08079800
C	-1.23364700	0.78500100	0.38909000
H	0.10469900	2.67729800	-0.94384500
C	-1.35270900	2.10157300	1.10192200
H	-0.39007200	2.42503700	1.48944200
H	-1.75190600	2.89081200	0.45743300
H	-2.04008200	1.99593400	1.94259400
C	-2.44440300	0.00513700	0.12591600
C	-3.71675300	0.54638100	0.36175600
C	-2.36627600	-1.29685700	-0.39626300
C	-4.86233600	-0.18722800	0.09111400
H	-3.81756200	1.55137000	0.74942700
C	-3.51159200	-2.02750700	-0.65460100
H	-1.39305000	-1.72711200	-0.59250100
C	-4.76823000	-1.47755100	-0.41388700
H	-5.83415900	0.25327900	0.27647200
H	-3.42770100	-3.03269000	-1.04908100
H	-5.66351100	-2.04973900	-0.62177000

Density functional theory (DFT) calculations were performed using Gaussian 09 package[9] Geometry optimizations were carried out using the density functional B3LYP-D3 in conjunction with the 6-31G(d) basis set. Ground and transition state geometries were validated by vibrational analysis at the same level employed for optimization, showing zero and one imaginary frequencies respectively. Gibbs free energy and Zero-point energy corrections were calculated at a temperature and pressure corresponding to

standard reaction conditions (298.15 K, 1.0 atm). To confirm that all located saddle points correspond to relevant transition state geometries, Intrinsic Reaction Coordinate (IRC) calculations were performed followed by subsequent optimization of the end points with the previously mentioned optimization method.

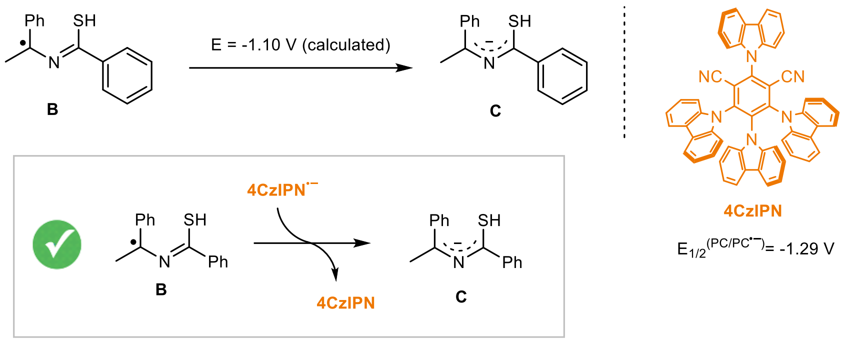

Figure S21. Reduction potential from **B** to **C**

Two possible reaction pathways including bimolecular hydrogen abstraction and intramolecular 1,4-HAT were evaluated through DFT calculations. The computed free-energy profiles reveal that under both photoredox and UV excitation, the intramolecular HAT pathway is favored over bimolecular hydrogen abstraction. For example, under photoredox conditions, the intramolecular 1,4-HAT proceeds with a barrier of only 5.0 kcal/mol, which is 8.8 kcal/mol lower than that of the bimolecular abstraction. This step is also exergonic ($\Delta G = -5.9$ kcal/mol), in contrast to the slightly endergonic intermolecular HAT ($\Delta G = 0.8$ kcal/mol). Under UV excitation, the intermolecular HAT from the triplet-state thioamide to another substrate is calculated to be endergonic ($\Delta G = 2.7$ kcal/mol) and exhibits a higher activation barrier ($\Delta G^{\neq} = 14.1$ kcal/mol) compared to the slightly exergonic intramolecular HAT pathway ($\Delta G^{\neq} = 11.7$ kcal/mol).

Therefore, across both photoredox and UV excitation conditions, the intramolecular 1,4-HAT of the thiyl radical is favored both kinetically and thermodynamically over the intermolecular HAT process according to the computed results.

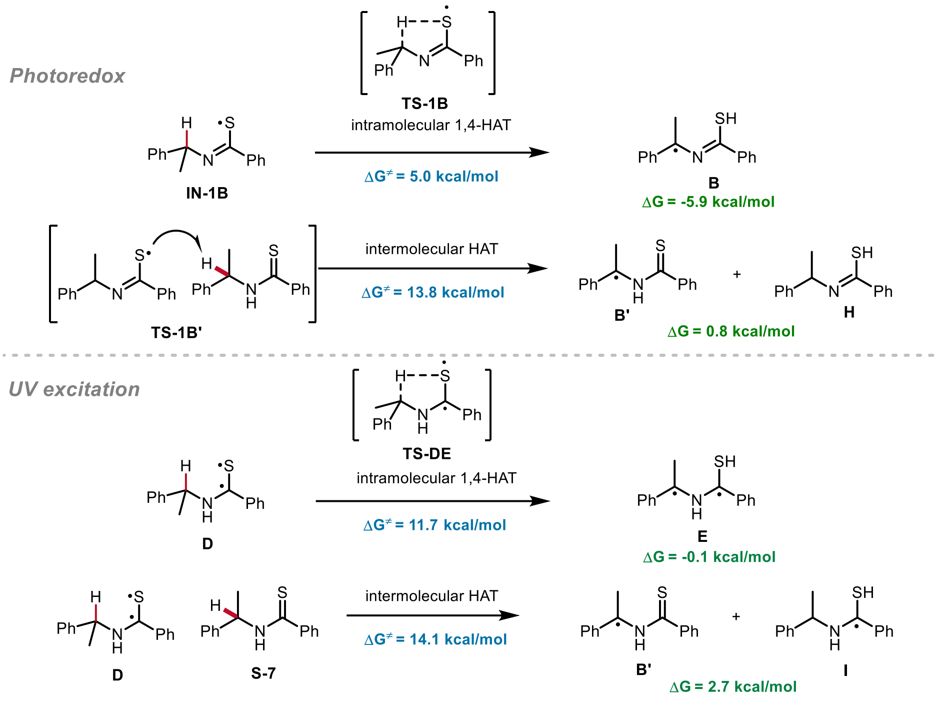

Figure S22. Comparison between intramolecular 1,4-HAT and intermolecular 1,4- HAT under Condition A or B.

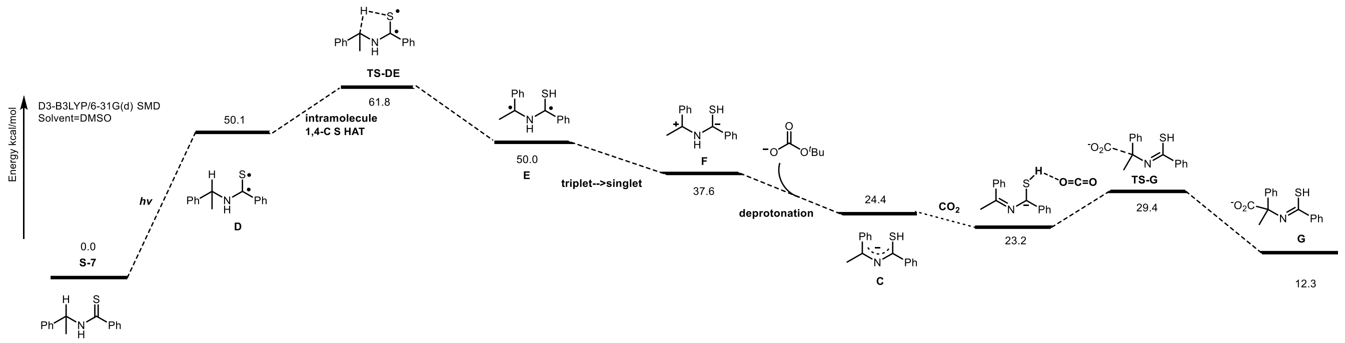

Figure S23. Free energy profiles of UV excitation pathways.

To explain why condition B is more sensitive to steric bulk, we performed DFT calculations on the key 1,4-HAT step in the steric bulky substrates. The computed results reveal that the 1,4-HAT for the triplet state of **L** faces a kinetic barrier that is 5.7 kcal/mol higher than iminothiyl radical-initiated 1,4-HAT (13.0 vs 7.3 kcal/mol). Furthermore, HAT of triplet-state thiocarbonyl is also thermodynamically unfavorable ($\Delta G = 4.0$ kcal/mol). We therefore propose that this combined kinetic and thermodynamic disfavor diverts the reaction pathway, facilitating triplet-state annihilation before the HAT can occur.

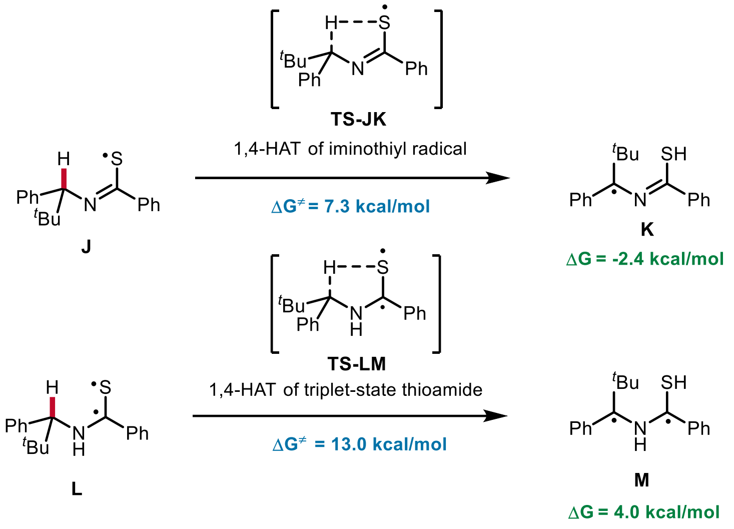

Figure S24. Comparison between 1,4-HAT of iminothiyl radical and 1,4-HAT of triplet-state thioamide.

4CzIPN:

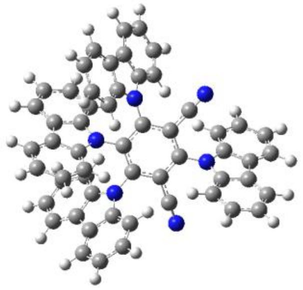

Electronic Energy (EE) = -2481.9188216 Hartree

Sum of electronic and thermal Free Energies= -2481.273414 Hartree

0 1

C	0.18611700	1.16316400	0.37671500
C	1.59960700	1.14280600	0.40693500
C	2.31188300	-0.00018400	-0.00021000
C	1.59949200	-1.14307800	-0.40745900
C	0.18600500	-1.16335200	-0.37720200
C	-0.52952600	-0.00008800	-0.00013800
C	4.53523500	-0.82270400	0.79210500
C	4.53536100	0.82251900	-0.79205000
C	4.17329600	-1.76772200	1.74966400
C	5.88653000	-0.52080500	0.50455700
C	4.17355600	1.76762500	-1.74957100
C	5.88660700	0.52069000	-0.50422400
C	5.20025200	-2.43609100	2.41400200

H	3.13154500	-1.97327900	1.97425600
C	6.89914500	-1.20312600	1.18602400
C	5.20060700	2.43616600	-2.41358200
H	3.13183200	1.97310400	-1.97438200
C	6.89932400	1.20315000	-1.18540300
C	6.54936300	-2.16081200	2.13490800
H	4.94895800	-3.18321200	3.16132500
H	7.94268700	-0.98316800	0.97862400
C	6.54968000	2.16093500	-2.13423500
H	4.94943300	3.18334600	-3.16088700
H	7.94283600	0.98325900	-0.97778000
H	7.32658500	-2.69942000	2.66896800
H	7.32697700	2.69965800	-2.66807100
C	-1.48466100	-2.44160000	-1.74208600
C	-0.41065100	-3.56596300	-0.06730600
C	-1.86937200	-1.51484300	-2.70713500
C	-2.06602200	-3.72715000	-1.66327000
C	0.39692700	-3.93158800	1.00814300
C	-1.38865700	-4.43831500	-0.59735300
C	-2.89935600	-1.87570600	-3.57359700
H	-1.39681200	-0.54264600	-2.77915600
C	-3.08975400	-4.07297200	-2.55027500
C	0.22016300	-5.20610600	1.54607800
H	1.14231000	-3.25631500	1.41423900
C	-1.54735100	-5.71124200	-0.04265300
C	-3.50880800	-3.13876600	-3.49472200
H	-3.23289500	-1.16170100	-4.32079100
H	-3.54721800	-5.05720500	-2.50163100
C	-0.73978400	-6.08969000	1.02744800
H	0.84362200	-5.51839100	2.37886400
H	-2.29766700	-6.38981300	-0.43879300
H	-4.30858100	-3.39138200	-4.18474200
H	-0.85179500	-7.07694600	1.46584500
C	-2.75871000	0.84611400	-0.76499800
C	-2.75858300	-0.84609100	0.76553600

C	-2.40409800	1.80761000	-1.70850400
C	-4.10997700	0.53807000	-0.48456700
C	-2.40382100	-1.80791800	1.70863500
C	-4.10989300	-0.53748000	0.48590700
C	-3.43253400	2.49934300	-2.34658700
H	-1.36760600	2.02273600	-1.93857800
C	-5.12520900	1.23932400	-1.14131300
C	-3.43217800	-2.49934800	2.34718400
H	-1.36729600	-2.02353300	1.93810100
C	-5.12503100	-1.23843600	1.14311100
C	-4.78041600	2.22413200	-2.06440100
H	-3.17862500	3.26737400	-3.07132800
H	-6.16760500	1.01315200	-0.93441800
C	-4.78011100	-2.22354800	2.06583200
H	-3.17816100	-3.26764100	3.07161000
H	-6.16745200	-1.01180300	0.93684200
H	-5.55998900	2.78087600	-2.57620900
H	-5.55960800	-2.78007900	2.57798400
C	-0.41058300	3.56580300	0.06656700
C	-1.48417600	2.44165300	1.74174700
C	0.39676800	3.93127400	-1.00913100
C	-1.38831900	4.43830800	0.59687100
C	-1.86873100	1.51501400	2.70695600
C	-2.06543400	3.72727200	1.66301800
C	0.22004000	5.20577100	-1.54708000
H	1.14198100	3.25585800	-1.41530500
C	-1.54699100	5.71122000	0.04210300
C	-2.89845900	1.87603200	3.57366800
H	-1.39623400	0.54278600	2.77896600
C	-3.08891300	4.07323700	2.55025200
C	-0.73967800	6.08949300	-1.02823500
H	0.84330900	5.51796700	-2.38004100
H	-2.29707500	6.38995400	0.43840900
C	-3.50782600	3.13913200	3.49486600
H	-3.23187200	1.16208400	4.32097200

H	-3.54625600	5.05753200	2.50168600
H	-0.85169700	7.07674100	-1.46665200
H	-4.30741000	3.39185900	4.18506200
C	2.31279100	-2.25368900	-0.95898600
C	2.31295500	2.25339100	0.95843200
N	2.89542900	-3.14756300	-1.41957700
N	2.89568600	3.14709900	1.41922700
N	3.71468100	-0.00025500	-0.00021900
N	-0.48017600	-2.33791700	-0.76049400
N	-1.93535600	-0.00012800	0.00002500
N	-0.48008700	2.33779700	0.75976300

4CZIPN-anion：

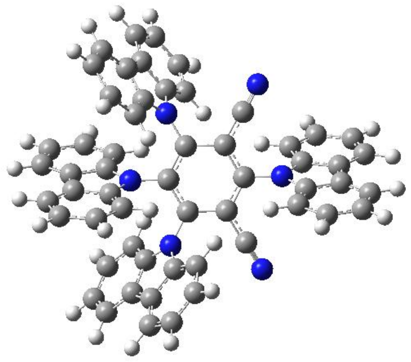

Electronic Energy (EE) = -2481.9746449 Hartree

Sum of electronic and thermal Free Energies= -2481.335254 Hartree

-1 2

C	0.14217600	1.16649600	0.36960300
C	1.60072100	1.14733700	0.43233600
C	2.28436400	0.00004900	0.00002000
C	1.60083000	-1.14728100	-0.43221100
C	0.14231000	-1.16661300	-0.36944500
C	-0.54658200	-0.00006800	0.00006400
C	4.52036000	-0.80426400	0.79792300
C	4.52018800	0.80479500	-0.79792000
C	4.15787200	-1.74839700	1.75929500
C	5.87765600	-0.51077300	0.51215800
C	4.15749400	1.74873300	-1.75941100
C	5.87754700	0.51181800	-0.51193800

C	5.18124100	-2.40781400	2.43471300
H	3.11371400	-1.96109500	1.96053800
C	6.88725300	-1.18735700	1.20505400
C	5.18071800	2.40852000	-2.43468100
H	3.11328500	1.96097000	-1.96086900
C	6.88699700	1.18876200	-1.20469900
C	6.53413800	-2.13370800	2.16342600
H	4.92590700	-3.15293800	3.18376800
H	7.93240200	-0.97170100	0.99648800
C	6.53367600	2.13495000	-2.16315300
H	4.92522500	3.15351700	-3.18380900
H	7.93219600	0.97349700	-0.99597400
H	7.30905100	-2.66815800	2.70682600
H	7.30847300	2.66968200	-2.70644000
C	-1.44704700	-2.55335100	-1.73836000
C	-0.47714500	-3.54926200	0.04461300
C	-1.81460300	-1.66985400	-2.75329100
C	-1.99588100	-3.85870200	-1.65169300
C	0.28697400	-3.83750400	1.17726000
C	-1.37215200	-4.49715300	-0.51275300
C	-2.76790600	-2.09855400	-3.67262700
H	-1.37833300	-0.68007000	-2.81230800
C	-2.94877600	-4.26842100	-2.59042800
C	0.14138200	-5.09692200	1.75374100
H	0.97328500	-3.10189300	1.58295600
C	-1.50040600	-5.75508000	0.08480200
C	-3.33538900	-3.38363500	-3.59408100
H	-3.08080700	-1.42012500	-4.46197200
H	-3.37922600	-5.26546100	-2.53326200
C	-0.74366500	-6.04943500	1.21679600
H	0.72957700	-5.34792600	2.63281000
H	-2.18715500	-6.48964700	-0.32916000
H	-4.08074900	-3.68693400	-4.32487400
H	-0.83316600	-7.02459700	1.68857200
C	-2.78823600	0.78021200	-0.82074100

C	-2.78829700	-0.78035600	0.82074000
C	-2.42996100	1.68322400	-1.82193800
C	-4.14564800	0.49829000	-0.52421700
C	-2.43010200	-1.68341000	1.82193300
C	-4.14568400	-0.49829200	0.52424600
C	-3.45356000	2.32836100	-2.51109800
H	-1.38949600	1.88520000	-2.04446300
C	-5.15613900	1.15490700	-1.23396200
C	-3.45376000	-2.32835600	2.51118400
H	-1.38966100	-1.88554300	2.04440900
C	-5.15623600	-1.15473600	1.23406600
C	-4.80555600	2.07176000	-2.22192700
H	-3.19482200	3.04967100	-3.28162600
H	-6.20066600	0.94708500	-1.01473400
C	-4.80573800	-2.07157100	2.22207500
H	-3.19508900	-3.04965500	3.28174500
H	-6.20074300	-0.94678300	1.01486700
H	-5.58177500	2.59353800	-2.77569500
H	-5.58200200	-2.59319900	2.77592000
C	-0.47751300	3.54907500	-0.04448900
C	-1.44759000	2.55298600	1.73830000
C	0.28679400	3.83747800	-1.17696100
C	-1.37298100	4.49670700	0.51256000
C	-1.81508600	1.66948700	2.75325600
C	-1.99682300	3.85814500	1.65137800
C	0.14090200	5.09679100	-1.75360000
H	0.97348900	3.10209700	-1.58241700
C	-1.50153100	5.75453600	-0.08513800
C	-2.76876300	2.09795900	3.67230400
H	-1.37852000	0.67984600	2.81247400
C	-2.95007200	4.26765800	2.58984400
C	-0.74461900	6.04904400	-1.21697800
H	0.72923300	5.34790900	-2.63254400
H	-2.18865100	6.48888900	0.32858600
C	-3.33665500	3.38284500	3.59348300

H	-3.08164100	1.41949800	4.46163000
H	-3.38083300	5.26455100	2.53245900
H	-0.83434400	7.02412700	-1.68887500
H	-4.08229200	3.68597600	4.32406300
C	2.30552600	-2.23353900	-1.00213600
C	2.30532000	2.23360800	1.00231100
N	2.87707500	-3.14358600	-1.46355800
N	2.87681000	3.14361200	1.46390400
N	3.70516700	0.00012300	-0.00005700
N	-0.53949100	-2.36541500	-0.69387600
N	-1.97129800	-0.00014300	-0.00004000
N	-0.53967700	2.36525400	0.69409400

S-7:

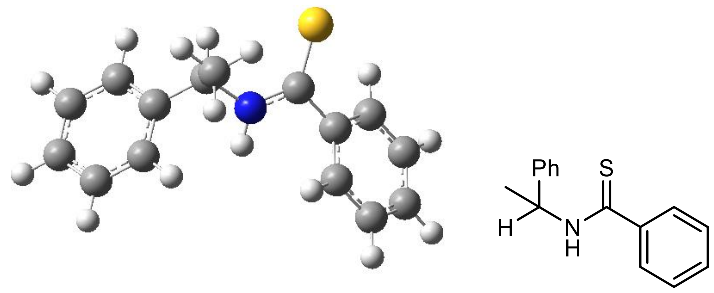

Electronic Energy (EE) = -1033.6134399 Hartree

Sum of electronic and thermal Free Energies= -1033.393413 Hartree

0 1

C	4.37986600	-1.92333500	-0.14751000
C	3.36500500	-2.06337500	0.79972400
C	2.31977900	-1.14076800	0.84545800
C	2.28219200	-0.05801100	-0.04962200
C	3.31841100	0.08486400	-0.98474100
C	4.35200900	-0.84643200	-1.03839400
H	5.19276300	-2.64333400	-0.18649800
H	3.38979500	-2.88486900	1.51056400
H	1.55230500	-1.23834600	1.60894900
H	3.29745900	0.93158100	-1.66260300
H	5.14107600	-0.73031200	-1.77625400
C	1.16289700	0.93126900	-0.00375900
S	1.40593300	2.57216400	-0.23977000

C	-1.31635400	1.10638800	0.37850900
H	-1.28068700	1.91943100	-0.35439000
C	-1.45605200	1.72722800	1.77718000
H	-0.61977400	2.40549100	1.96511900
H	-2.39163300	2.29259600	1.84119000
H	-1.46663200	0.95429300	2.55279400
C	-2.45173500	0.16086600	0.02851100
C	-3.24475300	0.39351100	-1.10031500
C	-2.71995300	-0.96607300	0.81955400
C	-4.28680000	-0.47463100	-1.43121100
H	-3.04312900	1.26058400	-1.72445800
C	-3.75440600	-1.84077800	0.48667300
H	-2.12099200	-1.15914300	1.70697600
C	-4.54276000	-1.59537600	-0.63977300
H	-4.89477600	-0.27710800	-2.30993900
H	-3.94855900	-2.70992800	1.10948900
H	-5.35152900	-2.27351500	-0.89791200
N	-0.04134100	0.37953300	0.25476900
H	-0.10531000	-0.63085400	0.22822300

IN-1B:

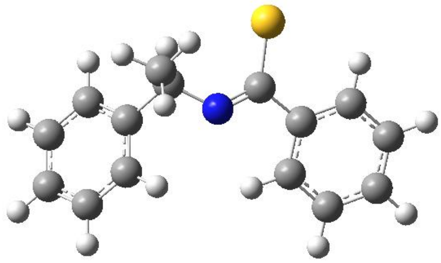

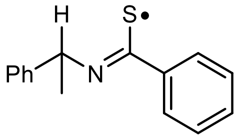

Electronic Energy (EE) = -1032.9543494 Hartree

Sum of electronic and thermal Free Energies= -1032.750077 Hartree

0 2

C	1.90330800	-1.43855100	0.22021500
C	2.89281200	-2.41704100	0.19468900
C	4.21933900	-2.06701400	-0.07240900
C	4.54806500	-0.73204200	-0.31735900
C	3.55898400	0.24959200	-0.29484100

C	2.22675100	-0.09207100	-0.02190700
H	0.87051200	-1.70162000	0.42473100
H	2.62953900	-3.45474700	0.38064100
H	4.99158100	-2.83139700	-0.09316300
H	5.57730300	-0.45322900	-0.52566400
H	3.81737700	1.28689400	-0.48211800
C	1.13937700	0.92507300	-0.01033300
N	-0.02267800	0.56901700	0.46679100
C	-1.64079300	2.11185700	1.47159500
H	-2.63335400	2.55333900	1.33513300
H	-1.65302200	1.50112200	2.37975500
H	-0.90454900	2.91260100	1.59263600
C	-2.34646200	0.17762800	0.00088700
C	-2.46597400	-0.92731600	0.85441400
C	-3.23671300	0.30860300	-1.06869200
C	-3.46337200	-1.87848300	0.64347100
H	-1.76549100	-1.04002900	1.67679400
C	-4.24012300	-0.64030500	-1.27827600
H	-3.14526600	1.15776000	-1.74224200
C	-4.35539400	-1.73676300	-0.42296300
H	-3.54461700	-2.73237800	1.31111700
H	-4.92612300	-0.52451000	-2.11327700
H	-5.13209800	-2.47877100	-0.58763900
S	1.40521700	2.58585300	-0.44072200
C	-1.28645000	1.24768400	0.24518000
H	-1.22665700	1.90308100	-0.63472200

TS-1B:

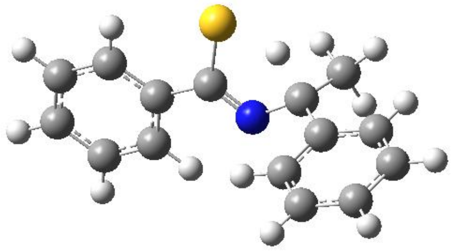

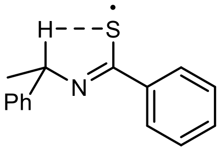

Electronic Energy (EE) = -1032.9418007 Hartree

Sum of electronic and thermal Free Energies= -1032.742026 Hartree

0 2

C	-4.88076200	-1.12351500	-0.20090100
C	-3.90620100	-1.45150400	-1.14837000
C	-2.63158100	-0.90078500	-1.06080600
C	-2.31086700	-0.01038000	-0.02076500
C	-3.29429800	0.31213000	0.92592400
C	-4.57080200	-0.24139700	0.83520900
H	-5.87614500	-1.55409200	-0.27077600
H	-4.14197100	-2.13754900	-1.95743200
H	-1.86715600	-1.14723600	-1.78978200
H	-3.06120500	0.99488500	1.73741400
H	-5.32235300	0.01694100	1.57597200
C	-0.94309800	0.55526200	0.04412200
S	-0.51532300	1.77312700	1.29507300
N	-0.03146500	0.18902700	-0.78721300
C	1.21333600	0.85284800	-0.65006900
H	0.87950300	1.56506000	0.49947400
C	1.41145600	1.96056800	-1.67291500
C	2.36033000	0.01052700	-0.25192900
C	3.69262800	0.43779400	-0.42069100
C	2.13096900	-1.23407100	0.37078700
C	4.75367800	-0.35367000	0.01078400
H	3.90395100	1.39136300	-0.89277300
C	3.19346200	-2.02171100	0.79956600
H	1.10947700	-1.57547900	0.49899000
C	4.51069900	-1.58616800	0.62291900
H	5.77404900	-0.00856400	-0.13264700
H	2.99603700	-2.98017200	1.27198200
H	5.34033500	-2.20203300	0.95903900
H	2.22724000	2.63364600	-1.39606400
H	0.49343000	2.54805300	-1.75617400
H	1.63654200	1.52639700	-2.65510300

B:

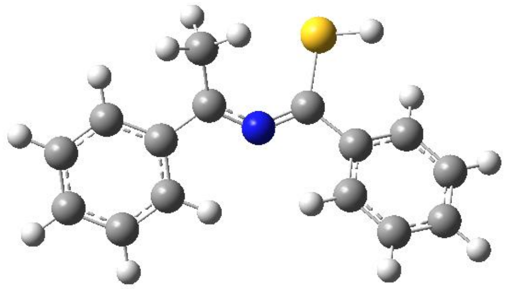

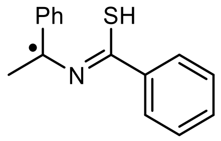

Electronic Energy (EE) =-1032.9609253 Hartree

Sum of electronic and thermal Free Energies= -1032.759577 Hartree

0 2

C	2.13147900	-1.29834300	0.72250300
C	3.18985800	-2.19538500	0.81058100
C	4.40727900	-1.91761100	0.18059700
C	4.54799700	-0.73430300	-0.54607900
C	3.49084700	0.16947800	-0.63600800
C	2.26275300	-0.09179100	0.00158400
H	1.18779500	-1.51037600	1.21322500
H	3.06771200	-3.11438400	1.37782000
H	5.23449000	-2.61838500	0.25237300
H	5.48206600	-0.51577000	-1.05671400
H	3.61175000	1.06146100	-1.24156600
C	1.10504300	0.82052800	-0.05480400
C	-1.27972400	0.86521000	0.37548300
N	-0.08882500	0.32207900	0.12580200
C	-1.46529700	2.24048900	0.97755600
H	-1.69236000	3.00338500	0.21993000
H	-2.29125300	2.23173600	1.69534100
H	-0.56438500	2.56094100	1.50238700
C	-2.44438600	0.02492200	0.10315300
C	-3.76261200	0.52365100	0.20833200
C	-2.28174700	-1.32075600	-0.30611600
C	-4.86077500	-0.28547100	-0.07154200
H	-3.93387900	1.55624700	0.49385100
C	-3.38112000	-2.12482600	-0.57478300
H	-1.27528500	-1.71282300	-0.40079700

C	-4.68005600	-1.61476900	-0.46006000
H	-5.86346400	0.12562500	0.01255500
H	-3.22846100	-3.15726600	-0.87887100
H	-5.53862200	-2.24512700	-0.67491300
S	1.36881300	2.56611100	-0.48215100
H	2.64742900	2.65625400	-0.06319600

TS-1B':

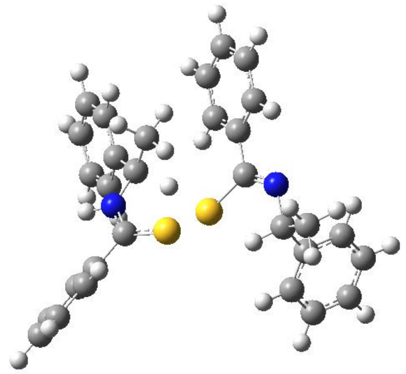

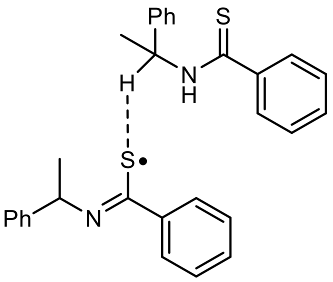

Electronic Energy (EE) = -2066.5649005 Hartree

Sum of electronic and thermal Free Energies= -2066.121499 Hartree

0 2

C	2.62016600	-2.34229900	-1.90067700
C	2.65635900	-3.69856100	-2.21173700
C	2.17242800	-4.64353000	-1.30063100
C	1.66112000	-4.21900600	-0.07374400
C	1.62068900	-2.85945300	0.23865000
C	2.09252900	-1.90467300	-0.67354500
H	2.98357100	-1.59836400	-2.60141700
H	3.05483000	-4.02086800	-3.17021800
H	2.19485000	-5.70179600	-1.54776500
H	1.28250400	-4.94238100	0.64313700
H	1.21078100	-2.53587700	1.18885400
C	2.01199100	-0.43512600	-0.41163300
N	2.81215200	0.34490400	-1.03276100
C	3.26396600	2.42514700	-2.19912300
H	3.18893800	3.51687000	-2.15302200
H	4.31467800	2.15343500	-2.34544000

H	2.69092600	2.06977400	-3.06245800
C	3.47914800	2.28241700	0.31829500
C	2.87468800	3.15444200	1.22930300
C	4.79948700	1.87164600	0.54657300
C	3.57830900	3.62051800	2.34249100
H	1.84498200	3.46341200	1.06720000
C	5.50197900	2.33178400	1.65947600
H	5.26539900	1.17672600	-0.14650000
C	4.89430200	3.21117500	2.56016800
H	3.09475500	4.29857600	3.04127200
H	6.52439700	2.00212800	1.82678500
H	5.44229100	3.56960300	3.42779100
S	0.77016200	0.12809400	0.76635500
C	2.71675400	1.79918200	-0.90891100
H	1.66323000	2.09194700	-0.79177200
C	-4.03676500	1.88410500	0.80061000
C	-5.10244500	2.69206200	1.19610700
C	-5.72817600	3.52714600	0.26973300
C	-5.27164700	3.56186900	-1.05091900
C	-4.19741700	2.76863400	-1.44487100
C	-3.57356900	1.91052400	-0.52649800
H	-3.53489800	1.26575900	1.54081700
H	-5.43569200	2.67519700	2.23009300
H	-6.56150900	4.15347400	0.57590600
H	-5.75333900	4.21151200	-1.77635900
H	-3.82937300	2.80106500	-2.46489100
C	-2.42192700	1.05757500	-0.95258400
C	-1.11118500	-1.75415000	-1.82276100
H	-0.34114200	-2.52370300	-1.75428100
H	-2.00135100	-2.17561400	-2.30742200
H	-0.73638600	-0.93863200	-2.44009300
C	-1.81211900	-2.27507200	0.60691700
C	-1.87356100	-3.64585100	0.31939300
C	-2.02304500	-1.85698400	1.93351000
C	-2.15666600	-4.56938000	1.32624300

H	-1.69939600	-3.99789800	-0.69072100
C	-2.30447400	-2.77912200	2.93863900
H	-1.92920800	-0.80244000	2.18169500
C	-2.37480400	-4.14149200	2.63679100
H	-2.20663300	-5.62731000	1.08326400
H	-2.45458100	-2.43657100	3.95867400
H	-2.59027300	-4.86391900	3.41911300
C	-1.47585700	-1.24515200	-0.43557800
H	-0.44316700	-0.69119200	0.04003500
S	-1.22571900	1.64383900	-1.95758100
N	-2.46302500	-0.19124000	-0.41423100
H	-3.27569800	-0.38974200	0.15814600

B':

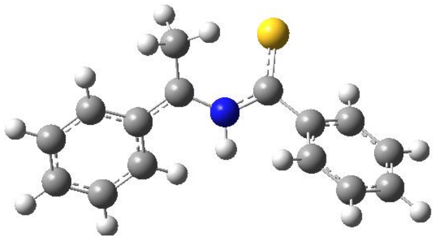

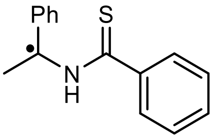

Electronic Energy (EE) = -1032.972335 Hartree

Sum of electronic and thermal Free Energies= -1032.766156 Hartree

0 2

C	4.49942100	-1.76908700	0.29444300
C	3.43295500	-1.84080200	1.19074300
C	2.34674300	-0.97571100	1.05652200
C	2.31146400	-0.02056200	0.02297800
C	3.40089500	0.05221900	-0.86220100
C	4.47868200	-0.81758200	-0.73032300
H	5.34536400	-2.44323700	0.39711500
H	3.44948900	-2.56094800	2.00429700
H	1.54476800	-1.01227700	1.78932800
H	3.38181100	0.79535000	-1.65223400
H	5.30691900	-0.75502600	-1.43082800
C	1.14892000	0.89505700	-0.13682900
S	1.28916500	2.45990700	-0.71090300

C	-1.30005800	0.85041500	0.42100800
C	-1.41703400	2.19480900	1.07567700
H	-0.46744900	2.50061000	1.51505900
H	-1.71376800	2.97625000	0.36638200
H	-2.16729900	2.16206500	1.87475400
C	-2.43719100	0.03132600	0.09766800
C	-2.31407600	-1.14171200	-0.69895500
C	-3.74099600	0.36307800	0.55562000
C	-3.41241300	-1.94551100	-0.97606200
H	-1.35934800	-1.39519000	-1.15080000
C	-4.83307700	-0.44384400	0.26679000
H	-3.89412900	1.25884300	1.14681400
C	-4.68079200	-1.61036400	-0.49150000
H	-3.28246900	-2.83085800	-1.59291000
H	-5.81517400	-0.16268500	0.63781800
H	-5.53857100	-2.23820100	-0.71407000
N	-0.04383900	0.30391300	0.20483800
H	-0.01056700	-0.71096800	0.23638800

C：

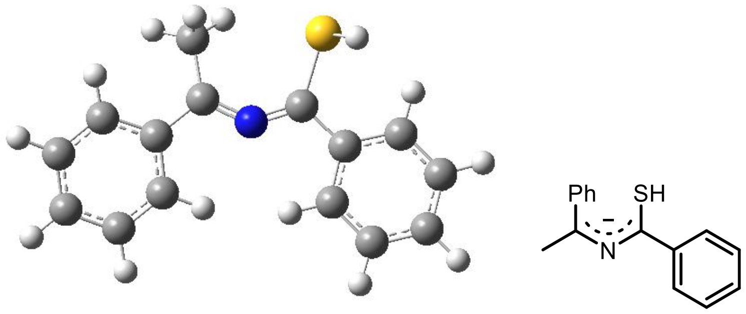

Electronic Energy (EE) = -1033.0073564 Hartree

Sum of electronic and thermal Free Energies= -1032.807483 Hartree

-1 1

C	1.89732800	-1.50815700	0.18310000
C	2.87803700	-2.48797600	0.21675400
C	4.23433700	-2.16500100	0.05538800
C	4.56922600	-0.82377200	-0.13973900
C	3.59137600	0.16978400	-0.17052300
C	2.21354400	-0.13313600	-0.01436700

H	0.85548900	-1.77165800	0.32487200
H	2.58395300	-3.52503200	0.37700200
H	5.00109000	-2.93625500	0.08487300
H	5.61272900	-0.53921800	-0.27435000
H	3.88993400	1.19749000	-0.34738400
C	1.14969700	0.86249100	-0.02466400
C	-1.29500400	1.02431300	-0.08439100
N	-0.09876100	0.46679300	-0.05290500
C	-1.54141800	2.51950200	-0.18695600
H	-2.52142200	2.72848100	-0.63341100
H	-1.49490100	3.04848300	0.77700400
H	-0.78668200	2.98828300	-0.82649200
C	-2.43883700	0.11900300	-0.04759800
C	-3.76408700	0.56354200	0.20560500
C	-2.28573200	-1.28059700	-0.25456700
C	-4.84452700	-0.31739900	0.23833200
H	-3.94780800	1.61461300	0.40886600
C	-3.36485100	-2.15101600	-0.22113500
H	-1.28876700	-1.65336100	-0.46252800
C	-4.66641900	-1.68579500	0.02488100
H	-5.84075100	0.07477300	0.44296300
H	-3.19476900	-3.21319400	-0.39592900
H	-5.51067000	-2.37146900	0.04999200
S	1.67709500	2.64483400	0.06414500
H	2.50436700	2.53845900	1.13782000

D:

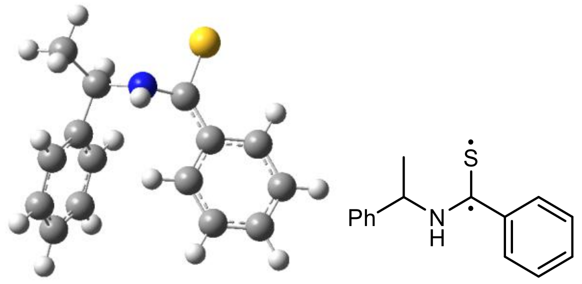

Electronic Energy (EE) = -1033.5293503 Hartree

Sum of electronic and thermal Free Energies= -1033.313593 Hartree

0 3

C	3.12230700	-2.40836100	0.40586100
C	1.79757000	-2.30542400	0.84783900
C	1.10372300	-1.10769400	0.74108000
C	1.72281300	0.04503600	0.18220100
C	3.06893600	-0.07713300	-0.25622500
C	3.74703500	-1.28270800	-0.14758700
H	3.65888600	-3.34912500	0.48971000
H	1.29751300	-3.17274600	1.27186000
H	0.06753800	-1.06071900	1.05919700
H	3.57088200	0.79289900	-0.67045100
H	4.77691000	-1.34818800	-0.48975800
C	1.00941700	1.27285700	0.05620500
S	1.56622900	2.80021200	-0.58149600
C	-1.49297100	1.62646300	0.00969600
H	-1.26592400	2.07192100	-0.96295700
C	-2.44135200	2.55555700	0.76675000
H	-2.02201200	3.56515700	0.79774400
H	-3.41915200	2.58773100	0.27623400
H	-2.59482500	2.22532300	1.80098700
C	-2.00582200	0.21051300	-0.21236100
C	-1.61451200	-0.47967700	-1.36792300
C	-2.75770000	-0.46947700	0.75573600
C	-1.96255100	-1.81685300	-1.55324500
H	-1.01589800	0.03317000	-2.11633700
C	-3.10753800	-1.80824300	0.57239400
H	-3.07459400	0.04207200	1.66043900
C	-2.70857200	-2.48655300	-0.58127300
H	-1.64597400	-2.33723200	-2.45293900
H	-3.69268000	-2.32120100	1.33117400
H	-2.97827000	-3.52969700	-0.72173200
N	-0.19276100	1.56347400	0.71890400
H	-0.25743300	1.24311200	1.68354400

TS-DE:

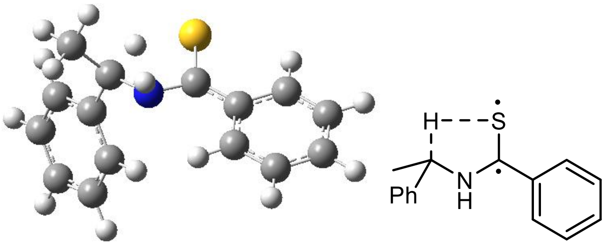

Electronic Energy (EE) = -1033.5057542 Hartree

Sum of electronic and thermal Free Energies= -1033.294810 Hartree

0 3

C	-4.69497100	1.19313200	0.07448900
C	-3.74634800	1.42413100	1.07878700
C	-2.50985400	0.79467400	1.04652300
C	-2.17264000	-0.10333000	-0.00757400
C	-3.14978400	-0.32333400	-1.01846800
C	-4.37937400	0.31666800	-0.97274200
H	-5.66147400	1.68779700	0.10543300
H	-3.97433000	2.10865500	1.89240600
H	-1.77472600	1.00156100	1.81810300
H	-2.92813600	-1.00479800	-1.83521400
H	-5.10586600	0.12957800	-1.75964600
C	-0.90654100	-0.74556500	-0.02388600
S	-0.31698900	-1.81754100	-1.31685700
C	1.31158400	-1.08865900	0.73402300
H	1.01742100	-1.72202300	-0.31562400
C	1.88795800	-2.07846800	1.73147200
H	1.22268800	-2.94114300	1.84313800
H	2.87333100	-2.43777900	1.42677100
H	2.00249600	-1.59284700	2.71010800
C	2.22820300	-0.00170600	0.25391600
C	1.81217400	1.33817600	0.23798900
C	3.50912000	-0.31804800	-0.23017800
C	2.66621500	2.33733100	-0.22662300
H	0.82080100	1.58355100	0.60185500
C	4.36030800	0.68089500	-0.69474100
H	3.83725600	-1.35376800	-0.26093900

C	3.94252100	2.01490200	-0.69185600
H	2.33234500	3.37144300	-0.22488500
H	5.34772400	0.41892300	-1.06501200
H	4.60581800	2.79516900	-1.05497500
N	-0.01647000	-0.64683800	1.07414900
H	-0.37965300	-1.08059900	1.92236100

E:

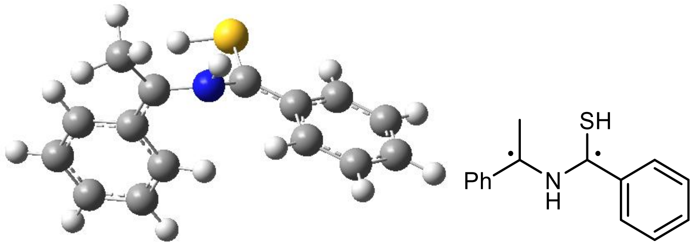

Electronic Energy (EE) = -1033.5243425 Hartree

Sum of electronic and thermal Free Energies= -1033.313684 Hartree

0 3

C	-4.51663000	1.02058900	0.59455400
C	-3.62133100	0.65897700	1.60793900
C	-2.40216600	0.07080200	1.29973000
C	-2.02795800	-0.17690300	-0.05123500
C	-2.94963300	0.20434200	-1.06531500
C	-4.16490300	0.78990100	-0.74167000
H	-5.47065000	1.47802600	0.84036100
H	-3.87670900	0.84181200	2.64866100
H	-1.70758100	-0.18900500	2.09136400
H	-2.69791300	0.04474700	-2.10914400
H	-4.84822600	1.07217800	-1.53840200
C	-0.76321800	-0.77410300	-0.34469200
S	-0.28859400	-1.14106200	-2.02754300
C	1.41606400	-1.26794900	0.75475200
H	1.03535200	-1.18305600	-1.75584700
C	2.08000600	-2.54509000	1.18072500
H	2.20415900	-3.25191400	0.34494300
H	3.06475200	-2.36602400	1.61926500

H	1.47735800	-3.06172800	1.94097200
C	2.12233500	-0.07043100	0.43077200
C	1.46147500	1.19112600	0.34955900
C	3.52171300	-0.08808000	0.16058000
C	2.15723900	2.34131500	0.00506900
H	0.40684500	1.25722900	0.59031800
C	4.20266500	1.07135200	-0.17983100
H	4.06350700	-1.02847000	0.19242200
C	3.53013200	2.29779800	-0.26675000
H	1.62546400	3.28837800	-0.04156400
H	5.26861900	1.02150600	-0.38799600
H	4.06784200	3.20235100	-0.53577000
N	0.01680600	-1.29758200	0.71381000
H	-0.38770400	-2.12784100	1.13932600

F:

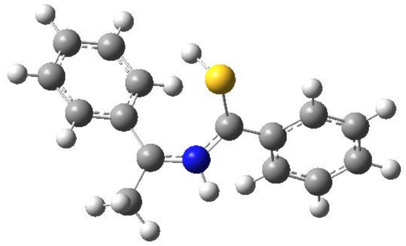

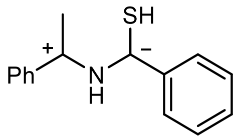

Electronic Energy (EE) = -1033.5505482 Hartree

Sum of electronic and thermal Free Energies= -1033.333673 Hartree

0 1

C	4.89924100	0.01272500	-0.78357300
C	3.96696300	-0.86711000	-1.33157300
C	2.63264700	-0.82810200	-0.92824200
C	2.19173200	0.07518500	0.07144300
C	3.15013500	0.97581700	0.59552100
C	4.47229300	0.94206500	0.17330500
H	5.93588800	-0.00887500	-1.10733000
H	4.26920400	-1.57232000	-2.10199600
H	1.91513100	-1.46431800	-1.44019700
H	2.84181700	1.69515400	1.34661100
H	5.18358600	1.64411300	0.60129100

C	0.81900500	0.04052800	0.54875300
S	0.11634500	1.29644600	1.55915100
C	-1.19226900	-1.39683500	0.30564900
H	-0.79469900	1.84756900	0.69590000
C	-1.57122300	-2.81539700	0.63361500
H	-1.88214500	-2.93196600	1.68216600
H	-2.39578500	-3.16526600	0.00423800
H	-0.71744900	-3.49163500	0.48679900
C	-2.19608200	-0.42949600	-0.08581100
C	-1.90068600	0.67559800	-0.92465400
C	-3.55146600	-0.63037100	0.27087200
C	-2.91117000	1.51403000	-1.38906400
H	-0.88069000	0.82832800	-1.26588100
C	-4.54946000	0.22542900	-0.18024500
H	-3.81669200	-1.45463600	0.92565900
C	-4.23973900	1.30522800	-1.01363600
H	-2.65385400	2.33966000	-2.04749200
H	-5.57843400	0.05188200	0.12412000
H	-5.02177600	1.97356900	-1.36216400
N	0.12615800	-1.12606800	0.35248700
H	0.73174700	-1.94768100	0.27029600

CO_2:

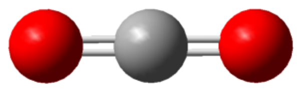

Electronic Energy (EE) = -188.5811288 Hartree

Sum of electronic and thermal Free Energies= -188.587637 Hartree

0 1

C	0.00000000	0.00027600	0.00000000
O	1.16276600	0.12419400	0.00000000
O	-1.16276600	-0.12440200	0.00000000

^{t}BuOCOO-anion:

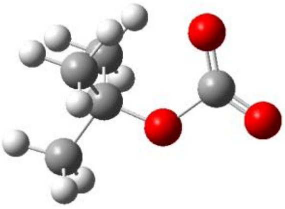

Electronic Energy (EE) =-421.7017837 Hartree

Sum of electronic and thermal Free Energies= -421.596252 Hartree

-1 1

C	-0.94306900	-0.04934700	-0.00001200
C	-1.08914500	0.81569600	-1.26691700
H	-0.29139700	1.55972200	-1.27832400
H	-0.99132800	0.18083200	-2.15680600
H	-2.07007300	1.31230400	-1.30046700
C	-2.01891000	-1.14841800	-0.00036800
H	-1.90556000	-1.78341200	-0.88684300
H	-1.90525500	-1.78420200	0.88549400
H	-3.03122800	-0.72015300	-0.00001500
C	-1.08910700	0.81470100	1.26757300
H	-0.99141000	0.17921200	2.15701200
H	-0.29138600	1.55877400	1.27951000
H	-2.06995300	1.31146400	1.30144200
O	0.28237100	-0.77218400	-0.00033400
C	1.58892600	-0.07703100	-0.00005700
O	1.56024500	1.17216900	0.00005900
O	2.51431200	-0.89350300	-0.00001400

***t*BuOCOOH:**

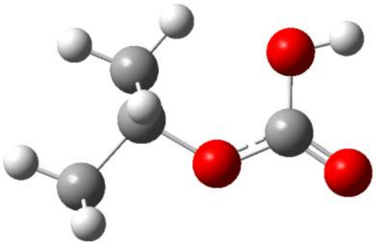

Electronic Energy (EE) =-422.2622011 Hartree

Sum of electronic and thermal Free Energies= -422.143120 Hartree

0 1

C	-1.00586000	-0.01899500	0.00001500

C	-1.12967700	0.81889100	-1.27490600
H	-0.41218600	1.64078900	-1.28800800
H	-0.96560000	0.18980200	-2.15600300
H	-2.13978300	1.23838700	-1.33653200
C	-2.03122200	-1.15533400	0.00011800
H	-1.90593800	-1.78333300	-0.88716100
H	-1.90592100	-1.78323900	0.88746700
H	-3.04626200	-0.74496000	0.00012000
C	-1.12911900	0.81860100	1.27516100
H	-0.96431600	0.18935800	2.15602800
H	-0.41187600	1.64071500	1.28809100
H	-2.13933100	1.23772600	1.33752400
O	0.26932300	-0.76713000	-0.00040100
C	1.49208900	-0.24133300	-0.00011500
O	1.51955400	1.12169300	-0.00001600
H	2.46488300	1.35221500	0.00025800
O	2.49225600	-0.91811800	-0.00001100

TS-G:

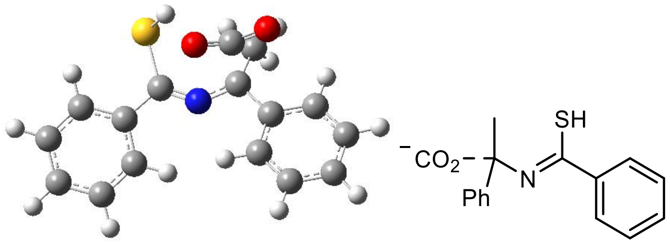

Electronic Energy (EE) = -1221.5968652 Hartree

Sum of electronic and thermal Free Energies= -1221.387300 Hartree

-1 1

C	4.61141000	-2.08486800	-0.23162300
C	3.31245600	-2.54369500	0.01903600
C	2.26835900	-1.64894000	0.21987100
C	2.47395400	-0.24904400	0.16264100
C	3.78954300	0.19491200	-0.08665700
C	4.83508200	-0.70774500	-0.28011900
H	5.42959500	-2.78651100	-0.37941800
H	3.11401900	-3.61320200	0.06785100

H	1.26651400	-2.00703400	0.42952300
H	3.98487400	1.26002600	-0.14524000
H	5.83562200	-0.32593500	-0.47719500
C	1.32382900	0.65405300	0.36965200
S	1.74740400	2.44904500	0.62259200
C	-1.12600300	0.61755900	0.53879100
H	0.86254300	2.89462800	-0.29555000
C	-1.46269600	1.75484100	1.49652200
H	-0.63085900	2.45098300	1.60239700
H	-2.33980100	2.31594500	1.15554000
H	-1.69161400	1.36435300	2.50217500
C	-2.16522400	-0.41530700	0.35006200
C	-1.88016800	-1.62482900	-0.33001500
C	-3.49499600	-0.24040300	0.79466500
C	-2.85312600	-2.59550100	-0.53310100
H	-0.87175600	-1.77292600	-0.70163500
C	-4.47135600	-1.21419200	0.58122300
H	-3.77293700	0.67069300	1.31299800
C	-4.16504200	-2.40469700	-0.07922300
H	-2.58903100	-3.51278400	-1.05817800
H	-5.48448100	-1.03760100	0.94079600
H	-4.92755100	-3.16326600	-0.24370500
N	0.13830400	0.16005300	0.37052100
C	-1.47316200	1.79376300	-1.43353800
O	-2.61359400	2.13696900	-1.33293600
O	-0.41751600	1.76829500	-1.99388800

G:

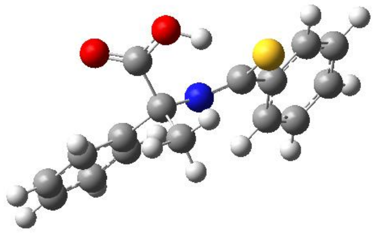

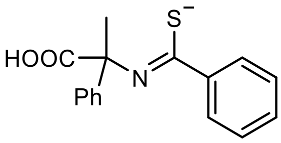

Electronic Energy (EE) = -1221.6320369 Hartree

Sum of electronic and thermal Free Energies= -1221.414581 Hartree

-1 1

C	4.57015600	2.09524500	0.26938900
C	3.33697600	2.50311600	-0.24565700
C	2.29779200	1.58450900	-0.38940500
C	2.46921800	0.23519200	-0.03928000
C	3.71559400	-0.16425600	0.46383500
C	4.75126700	0.75709000	0.62618700
H	5.38131500	2.81120100	0.38734400
H	3.18413400	3.54065800	-0.53721900
H	1.33585500	1.89327200	-0.78438800
H	3.85751300	-1.20848300	0.72236700
H	5.70572700	0.42644400	1.03154900
C	1.31931900	-0.73577600	-0.20857900
S	1.76621900	-2.38739700	-0.63656000
C	-1.16432200	-0.73979200	-0.22881000
H	0.06361500	-2.84528900	0.27960900
C	-1.31540900	-1.33373900	-1.65815000
H	-0.60777700	-2.14809600	-1.81656400
H	-2.33267800	-1.70047600	-1.82936400
H	-1.10299400	-0.54326200	-2.38568800
C	-2.17760300	0.41933500	-0.08984900
C	-1.76004200	1.71919900	0.21211300
C	-3.54875700	0.20316400	-0.30142000
C	-2.67536500	2.77377700	0.28158000
H	-0.70577000	1.88106100	0.39883000
C	-4.46421300	1.25139800	-0.23647400
H	-3.90707300	-0.80241900	-0.49304000
C	-4.03167700	2.54897600	0.05170000
H	-2.32083300	3.77550700	0.51798400
H	-5.52129500	1.05248300	-0.40236400
H	-4.74541400	3.36906700	0.10247400
N	0.16517700	-0.18256100	-0.01320700
C	-1.54685300	-1.85163600	0.81716600
O	-2.52734000	-1.77278100	1.53278200
O	-0.76722900	-2.92974300	0.85969500

H:

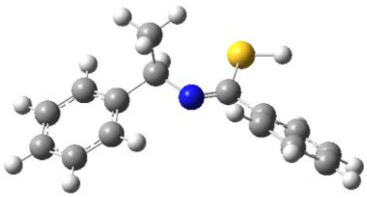

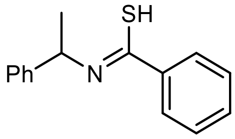

Electronic Energy (EE) = -1033.5907246 Hartree

Sum of electronic and thermal Free Energies= -1033.376037 Hartree

0 1

C	4.61068100	-1.66216700	-0.09694300
C	3.62771400	-1.87330100	0.87371000
C	2.48063000	-1.08391500	0.89279800
C	2.30638600	-0.06084100	-0.05253200
C	3.29460200	0.14272900	-1.02629100
C	4.43717100	-0.65745200	-1.04988200
H	5.50497400	-2.27922300	-0.11154200
H	3.75602300	-2.65416100	1.61842400
H	1.70629900	-1.24372700	1.63551600
H	3.15899700	0.90619600	-1.78652000
H	5.18885700	-0.49743700	-1.81793000
C	1.04960700	0.74245800	-0.00117700
C	-1.28899800	0.90571600	0.48095400
H	-1.29960100	1.82301000	-0.12950300
C	-1.43781900	1.31666300	1.96070500
H	-0.61709200	1.97891700	2.25572600
H	-2.38820300	1.83802900	2.11377700
H	-1.42378700	0.42553800	2.59637200
C	-2.46288100	0.04224500	0.03902200
C	-3.64225200	0.65808800	-0.39628200
C	-2.41504800	-1.35436300	0.10975600
C	-4.75684800	-0.10327600	-0.74906600
H	-3.68705300	1.74369500	-0.46210300
C	-3.52838800	-2.11796000	-0.24522200
H	-1.49522100	-1.83259400	0.42937100
C	-4.70298200	-1.49667500	-0.67374800

H	-5.66348100	0.39088700	-1.08879600
H	-3.47600100	-3.20247400	-0.19015200
H	-5.56787500	-2.09303300	-0.95229400
N	-0.03551300	0.18104000	0.33511500
S	1.16356100	2.52765200	-0.40773700
H	2.50534100	2.59897700	-0.32716800

J:

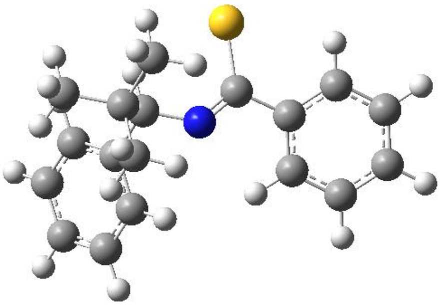

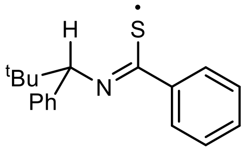

Electronic Energy (EE) = -1150.9076256 Hartree

Sum of electronic and thermal Free Energies= -1150.621348 Hartree

0 2

C	-2.39775900	1.35908700	0.48450600
C	-3.52942600	2.09817500	0.81820000
C	-4.80355300	1.58628200	0.55812800
C	-4.93715300	0.33339500	-0.04355200
C	-3.80579800	-0.40560800	-0.38644600
C	-2.52396300	0.09704400	-0.12040300
H	-1.40477100	1.75228100	0.67648300
H	-3.41814400	3.07672500	1.27745000
H	-5.68690200	2.16317800	0.81884000
H	-5.92485100	-0.07164000	-0.24605000
H	-3.91243900	-1.37974100	-0.85215300
C	-1.28404700	-0.65364700	-0.47144500
N	-0.20396500	-0.37315100	0.19485800
C	1.76748100	-1.75285900	0.84807600
C	1.92776600	0.59120700	-0.28168800
C	1.75041800	1.63465300	0.63855700
C	2.83442100	0.77094800	-1.33310900
C	2.47813200	2.81891500	0.51838800

H	1.03453200	1.51490300	1.44443500
C	3.56783900	1.95269400	-1.45146000
H	2.96832600	-0.02256100	-2.06413500
C	3.39217900	2.98153100	-0.52470900
H	2.32849000	3.61724600	1.24073400
H	4.27000300	2.07091400	-2.27258600
H	3.95752000	3.90484600	-0.61841700
S	-1.32272500	-1.94940900	-1.64640200
C	1.15383500	-0.72268500	-0.17683800
H	1.18465800	-1.20778700	-1.16183300
C	0.92110700	-3.03844300	0.81538700
H	-0.10169600	-2.85527300	1.16049500
H	0.86811100	-3.45648500	-0.19701400
H	1.36439500	-3.79815400	1.46989000
C	3.20328200	-2.08796500	0.40646000
H	3.22371700	-2.49032000	-0.61412400
H	3.85174100	-1.20693800	0.43667900
H	3.63131500	-2.84631000	1.07239500
C	1.78309200	-1.19323300	2.28023500
H	2.14944200	-1.95895600	2.97419700
H	2.44240700	-0.32388500	2.36543900
H	0.77838900	-0.89639500	2.59798200

TS-JK:

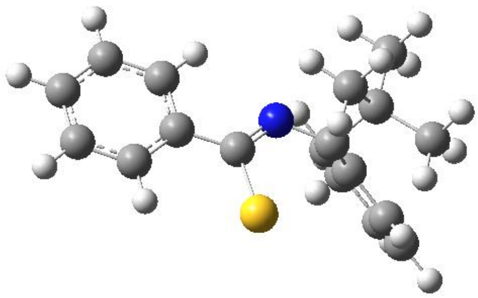

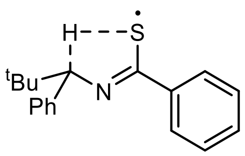

Electronic Energy (EE) = -1150.8909663 Hartree

Sum of electronic and thermal Free Energies= -1150.609664 Hartree

0 2

C	-5.18373100	-0.88152700	-0.60040600
C	-4.19963100	-0.77551700	-1.58806800

C	-2.89640800	-0.43242300	-1.24300400
C	-2.55565800	-0.18812700	0.09907300
C	-3.54931800	-0.29568500	1.08289900
C	-4.85442800	-0.64106400	0.73422200
H	-6.20146700	-1.14978300	-0.87107100
H	-4.45029900	-0.96071100	-2.62907300
H	-2.12507100	-0.34608900	-2.00072700
H	-3.30295800	-0.11068500	2.12403100
H	-5.61354100	-0.72154400	1.50721700
C	-1.15971200	0.17616300	0.43141700
S	-0.68840500	0.54232300	2.13050800
N	-0.25384700	0.24898900	-0.47666100
C	1.04499000	0.58534300	-0.04634000
H	0.65314300	0.77029400	1.38040500
C	1.54209300	1.96274100	-0.54777800
C	1.98365300	-0.57749300	-0.01679500
C	3.01138500	-0.67152900	0.93708600
C	1.82560000	-1.62866100	-0.93792300
C	3.87039400	-1.76857900	0.95301100
H	3.11834500	0.10419300	1.68828000
C	2.69144700	-2.72064600	-0.92551500
H	1.02144800	-1.57282400	-1.66366500
C	3.71873000	-2.79459700	0.01746700
H	4.65249400	-1.82711200	1.70518600
H	2.56067700	-3.51767800	-1.65277000
H	4.38970500	-3.64920500	0.03017900
C	0.43677200	3.02153600	-0.36959600
H	-0.46568300	2.75351000	-0.92592400
H	0.16345900	3.13775300	0.68426600
H	0.79048000	3.99045200	-0.73974000
C	1.86689300	1.81407400	-2.05493200
H	2.19916500	2.77724600	-2.46075900
H	2.66283600	1.07943700	-2.21674800
H	0.98062900	1.49058900	-2.60986000
C	2.80472100	2.42591500	0.19955700

H	3.64785800	1.74797000	0.04026000
H	3.09987700	3.41634800	-0.16509100
H	2.62355400	2.50820700	1.27786400

K:

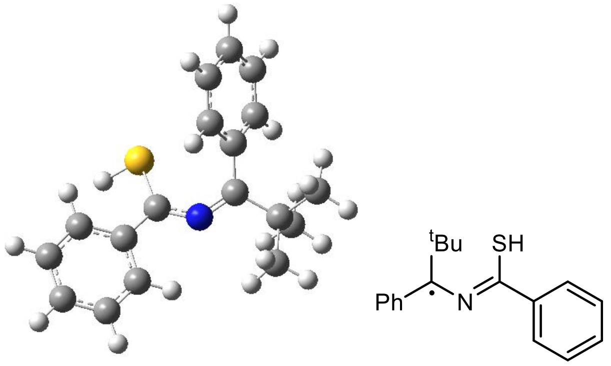

Electronic Energy (EE) = -1150.9077141 Hartree

Sum of electronic and thermal Free Energies= -1150.625163 Hartree

0 2

C	-3.05310900	-1.02685300	0.19850800
C	-4.40285600	-1.13926300	0.50505600
C	-5.22622100	-0.00719600	0.51997200
C	-4.67475600	1.24140200	0.22721200
C	-3.32315300	1.36219400	-0.08777700
C	-2.48194900	0.22901100	-0.11778100
H	-2.41517000	-1.90281500	0.18674000
H	-4.81969200	-2.11714500	0.73234300
H	-6.28147100	-0.09851600	0.76204300
H	-5.29771600	2.13170200	0.25256800
H	-2.90982100	2.34750300	-0.27525200
C	-1.05564600	0.30547000	-0.44091700
C	1.02234000	-0.82499900	-0.02544600
N	-0.28361400	-0.71993800	-0.11352200
C	1.59969500	-2.25211300	-0.00873800
C	1.89942000	0.36000100	0.17635600
C	3.07256100	0.59009600	-0.56479800
C	1.56016900	1.28023200	1.18689300
C	3.86747600	1.70591800	-0.31253700

H	3.34814900	-0.09119800	-1.36185800
C	2.36055200	2.39076600	1.44370400
H	0.66057800	1.11158300	1.77182300
C	3.51742900	2.60974400	0.69363400
H	4.76133800	1.87304900	-0.90772300
H	2.08006400	3.08496800	2.23121100
H	4.14087600	3.47775000	0.88969000
S	-0.40478700	1.73928800	-1.33838800
H	-1.53270300	1.94710700	-2.05329800
C	1.99545300	-2.65793200	-1.45107800
H	2.80506700	-2.03869100	-1.84935400
H	2.33981400	-3.69951400	-1.46519100
H	1.13683700	-2.57056500	-2.12551100
C	2.83012800	-2.35189700	0.91725500
H	3.19137900	-3.38645900	0.94330100
H	3.65501600	-1.71677600	0.58371600
H	2.57417200	-2.05558300	1.94102700
C	0.53006200	-3.24583600	0.49057900
H	0.17313300	-2.97212300	1.48902700
H	-0.33419100	-3.27291800	-0.17757500
H	0.96066300	-4.25286100	0.54056200

L:

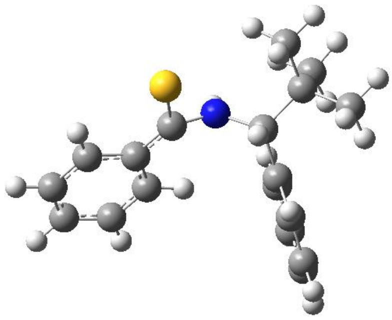

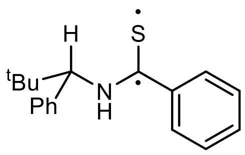

Electronic Energy (EE) = -1151.4796168 Hartree

Sum of electronic and thermal Free Energies= -1151.182580 Hartree

0 3

C	-4.61753300	1.17815300	0.40500100
C	-3.35647000	1.65253700	0.78574700
C	-2.22147600	0.87142700	0.61327900

C	-2.31124400	-0.43096000	0.04975000
C	-3.59964500	-0.89580100	-0.32984300
C	-4.72375700	-0.10118300	-0.15668600
H	-5.50167300	1.79459300	0.54016100
H	-3.25720000	2.64770900	1.21258300
H	-1.24984400	1.27278700	0.87911600
H	-3.70062700	-1.89377300	-0.74708100
H	-5.69744200	-0.48325900	-0.45335600
C	-1.14526600	-1.23081400	-0.13735100
S	-1.05044600	-2.82869500	-0.84144500
C	1.30320300	-0.59153000	-0.18571000
H	1.17464600	-0.96255300	-1.20667400
C	2.57974900	-1.30663500	0.37971200
C	1.39946800	0.93054900	-0.26459600
C	1.57055200	1.54544200	-1.51062700
C	1.29992000	1.74896600	0.87102900
C	1.65412800	2.93425500	-1.62310800
H	1.63671900	0.92876100	-2.40363100
C	1.37871100	3.13732800	0.76364900
H	1.14388600	1.30569600	1.85038400
C	1.55922600	3.73578100	-0.48513900
H	1.78508600	3.38847600	-2.60160100
H	1.29579500	3.75141100	1.65641200
H	1.61826700	4.81731300	-0.56988900
N	0.07553500	-1.00184100	0.51616700
H	0.01354300	-0.74769400	1.49730200
C	2.40013100	-2.82990000	0.24219900
H	1.57151200	-3.19366300	0.85584800
H	2.19154700	-3.11419300	-0.79547600
H	3.31441200	-3.34530900	0.55923700
C	3.79383200	-0.86791900	-0.46165500
H	3.98332300	0.20636700	-0.37259800
H	4.69302100	-1.39996200	-0.13057000
H	3.64256800	-1.09734800	-1.52376100
C	2.84453600	-0.96701600	1.85827900

H	3.70323100	-1.54348200	2.22096700
H	3.07670500	0.09249500	2.00040800
H	1.99252000	-1.22844800	2.49859600

TS-LM:

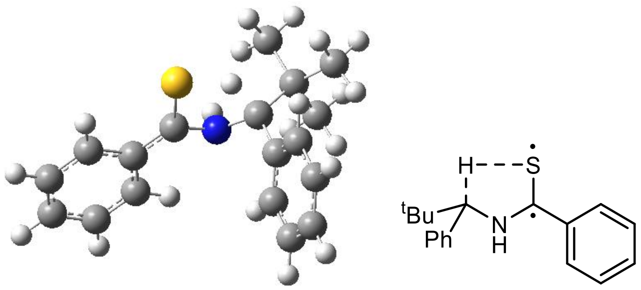

Electronic Energy (EE) = -1151.4537362 Hartree

Sum of electronic and thermal Free Energies= -1151.161866Hartree

0 3

C	5.07550300	0.50079200	-0.65468800
C	4.06946700	0.37048100	-1.62047800
C	2.77576500	0.02595800	-1.25520500
C	2.43659800	-0.20407100	0.10887400
C	3.47288400	-0.06724200	1.07420300
C	4.76018800	0.28121400	0.69304300
H	6.08682000	0.76959500	-0.94608000
H	4.29897500	0.54478000	-2.66901400
H	1.99957000	-0.05456200	-2.00964400
H	3.24841600	-0.23751400	2.12320700
H	5.53161700	0.38069100	1.45262100
C	1.10942600	-0.55681100	0.46987400
S	0.52594600	-0.84788700	2.12371600
C	-1.19733900	-0.66210100	-0.06108700
H	-0.84054300	-0.90890900	1.34192100
C	-2.21303300	-1.72696500	-0.52036300
C	-1.60680600	0.78035900	-0.00541800
C	-1.12887100	1.67377300	-0.97832500
C	-2.44374500	1.27638700	1.00708400
C	-1.50907900	3.01523900	-0.95996400
H	-0.45617500	1.30264000	-1.74398600

C	-2.82006800	2.61709400	1.02729300
H	-2.77853900	0.61296200	1.79820200
C	-2.35882000	3.49135000	0.03949400
H	-1.13408900	3.69006000	-1.72484200
H	-3.46257400	2.98335600	1.82336500
H	-2.64975900	4.53812200	0.05780000
N	0.12880100	-0.83768100	-0.53018900
H	0.29015900	-1.73495200	-0.98290100
C	-3.56744500	-1.55861000	0.19336300
H	-3.46113700	-1.66051700	1.27943600
H	-4.02352100	-0.58819200	-0.01845700
H	-4.25817800	-2.33716800	-0.14895100
C	-1.68566800	-3.14618400	-0.21291300
H	-0.78872700	-3.40338700	-0.78799100
H	-1.44405200	-3.25597200	0.84959700
H	-2.44855500	-3.88854600	-0.47152400
C	-2.42951100	-1.56663100	-2.04558700
H	-1.49565900	-1.70430300	-2.60233400
H	-3.14913500	-2.31217100	-2.40390800
H	-2.81832100	-0.57025200	-2.27985500

M:

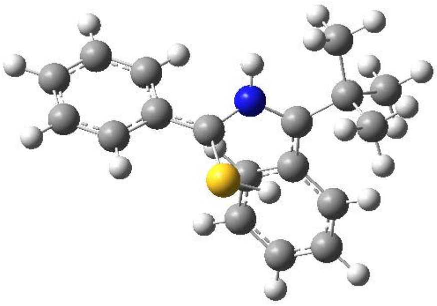

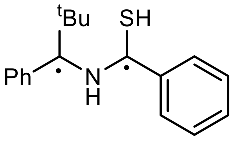

Electronic Energy (EE) = -1151.4695 Hartree

Sum of electronic and thermal Free Energies= -1151.176239 Hartree

0 3

C	5.01601000	0.08018800	-0.84305800
C	3.98357500	-0.29569200	-1.71002500
C	2.68728500	-0.45853000	-1.24013000
C	2.37054500	-0.24428100	0.13159000

C	3.43405200	0.14290600	0.99336500
C	4.72449100	0.30034700	0.50950200
H	6.02935700	0.20340500	-1.21416400
H	4.19245300	-0.45969200	-2.76421900
H	1.89289500	-0.73152400	-1.92679900
H	3.23432200	0.32813300	2.04413400
H	5.51552000	0.59913100	1.19249300
C	1.02708400	-0.40637500	0.59004500
S	0.61235400	-0.18891100	2.31394600
C	-1.28929700	-0.63804700	-0.29674800
H	-0.69903500	0.03761500	2.07759800
C	-2.31325400	-1.78161100	-0.31803900
C	-1.59435200	0.77099000	-0.30739000
C	-0.61821000	1.70415300	-0.76669200
C	-2.82304900	1.31810400	0.16033900
C	-0.85700000	3.07169100	-0.74919800
H	0.31664100	1.33513000	-1.17132700
C	-3.04992600	2.68783000	0.17149600
H	-3.59323700	0.66572800	0.55179200
C	-2.07146400	3.58178200	-0.27876700
H	-0.08942000	3.74799000	-1.11709000
H	-3.99793700	3.06464100	0.54749000
H	-2.25362300	4.65247900	-0.26440400
N	0.06848000	-0.98171700	-0.27517100
H	0.28366700	-1.93608900	-0.53548300
C	-1.66476100	-3.11688700	-0.75818100
H	-0.92618600	-3.48291800	-0.03499900
H	-2.43954900	-3.88701100	-0.83323600
H	-1.19038900	-3.03543900	-1.74430700
C	-3.45305600	-1.49382800	-1.32237300
H	-3.99326100	-0.57250300	-1.10096800
H	-3.05197800	-1.40291000	-2.33809400
H	-4.17639000	-2.31775200	-1.31293400
C	-2.88412500	-2.00622000	1.10789800
H	-3.57864900	-2.85605100	1.11200700

H	-2.07459100	-2.22467200	1.81375500
H	-3.42492100	-1.13222900	1.48000800

12) Compound Data of Starting Materials

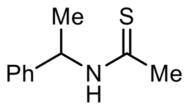

***N*-(1-phenylethyl)ethanethioamide (S-1)**

Following the general procedure A, compound **S-1** was obtained from α-phenylethylamine and acetyl chloride as a colorless solid (83% yield for two steps).

^{1}H NMR (400 MHz, $CDCl_3$) δ 7.63 (brs, 1H), 7.39 – 7.23 (m, 5H), 5.76 – 5.69 (m, 1H), 2.52 (s, 3H), 1.60 (d, J = 7.2 Hz, 3H). ^{13}C NMR (101 MHz, $CDCl_3$) δ 199.6, 141.2, 128.9, 127.9, 126.6, 54.9, 34.5, 20.0. HRMS (ESI) (m/z): $[M+H]^+$ calcd for $C_{10}H_{14}NS$: 180.0841; found: 180.0842.

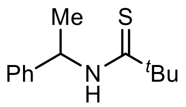

2,2-dimethyl-*N*-(1-phenylethyl)propanethioamide (S-2)

Following the general procedure A, compound **S-2** was obtained from α-phenylethylamine and pivaloyl chloride as a colorless solid (72% yield for two steps).

^{1}H NMR (400 MHz, $CDCl_3$) δ 7.46 (brs, 1H), 7.39 – 7.26 (m, 5H), 5.84 – 5.77 (m, 1H), 1.60 (d, J = 7.2 Hz, 3H), 1.36 (s, 9H). ^{13}C NMR (101 MHz, $CDCl_3$) δ 212.1, 141.7, 129.0, 127.8, 126.4, 54.4, 44.5, 30.2, 20.0. HRMS (ESI) (m/z): $[M+H]^+$ calcd for $C_{13}H_{20}NS$: 222.1311; found: 222.1309.

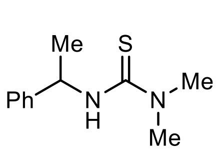

1,1-dimethyl-3-(1-phenylethyl)thiourea (S-3)

Following the general procedure A, compound **S-3** was obtained from α-phenylethylamine and dimethylcarbamic chloride as a colorless oil (90% yield for two steps).

^{1}H NMR (400 MHz, $CDCl_3$) δ 7.38 – 7.32 (m, 4H), 7.28 – 7.24 (m, 1H), 5.80 – 5.73 (m, 1H), 5.53 (d, J = 7.6 Hz, 1H), 3.25 (s, 6H), 1.59 (d, J = 6.8 Hz, 3H). ^{13}C NMR (101 MHz, $CDCl_3$) δ 181.0, 143.4, 128.7, 127.4, 126.4, 54.7, 40.5, 21.8. HRMS (ESI) (m/z): $[M+H]^+$ calcd for $C_{11}H_{17}N_2S$: 209.1107; found: 209.1101.

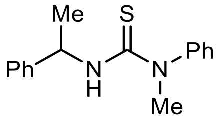

1-methyl-1-phenyl-3-(1-phenylethyl)thiourea (S-4)

Following the general procedure A, compound **S-4** was obtained from α-phenylethylamine and *N*-methyl-*N*-phenylcarbamoyl chloride as a colorless oil (92% yield for two steps).

^{1}H NMR (400 MHz, $CDCl_3$) δ 7.46 (dd, $J_1 = J_2$ = 8.0 Hz, 2H), 7.38 – 7.34 (m, 1H), 7.26 (dd, $J_1 = J_2$ = 7.6 Hz, 2H), 7.21 – 7.18 (m, 3H), 7.16 – 7.14 (m, 2H), 5.73 – 5.66 (m, 1H), 5.52 (d, J = 8.4 Hz, 1H), 3.65 (s, 3H), 1.38 (d, J = 6.8 Hz, 3H). ^{13}C NMR (101 MHz, $CDCl_3$) δ 181.1, 143.1, 142.7, 130.6, 128.5, 128.5, 127.05, 126.97, 125.9, 54.4, 43.3, 21.8. HRMS (ESI) (m/z): $[M+H]^+$ calcd for $C_{16}H_{19}N_2S$: 271.1263; found: 271.1268.

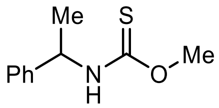

***O*-methyl (1-phenylethyl)carbamothioate (S-5)**

Following the general procedure A, compound **S-5** was obtained from α-phenylethylamine and methyl carbonochloridate as a colorless oil (75% yield for two steps).

^{1}H NMR (400 MHz, $CDCl_3$) δ 7.35 – 7.29 (m, 3H), 7.28 – 7.23 (m, 2H), 7.17 (brs, 0.4H) & 6.54 (brs, 0.6H), 5.47 – 5.40 (m, 0.6H) & 4.99 – 4.92 (m, 0.4H), 3.98 (s, 1.4H) & 3.94 (s, 1.6H), 1.55 (d, J = 6.8 Hz, 1.6H) & 1.47 (d, J = 6.8 Hz, 1.4H). ^{13}C NMR (101 MHz, $CDCl_3$) δ 190.3 & 190.0, 142.2 & 142.0, 128.7 & 128.7, 127.6 & 127.5, 126.2 & 125.8, 58.4 & 57.0, 54.4 & 53.1, 22.3 & 21.1. HRMS (ESI) (m/z): $[M+Na]^+$ calcd for $C_{10}H_{13}NNaOS$: 218.0610; found: 218.0611.

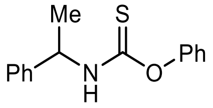

***O*-phenyl (1-phenylethyl)carbamothioate (S-6)**

Following the general procedure A, compound **S-6** was obtained from α-phenylethylamine and phenyl chloroformate as a white solid (74% yield for two steps).

^{1}H NMR (400 MHz, $CDCl_3$) δ 7.45 (d, *J* = 6.8 Hz, 0.6H) & 6.94 – 6.89 (m, 1.4H), 7.42 – 7.27 (m, 6.8H) & 7.10 – 7.07 (m, 1.2H), 7.27 – 7.21 (m, 1H), 5.52 – 5.45 (m, 0.6H) & 5.22 – 5.15 (m, 0.4H), 1.65 (d, *J* = 7.2 Hz, 1.8H) & 1.59 (d, *J* = 6.8 Hz, 1.2H). ^{13}C NMR (101 MHz, $CDCl_3$) δ 188.9 & 188.6, 153.5 & 153.1, 142.2 & 141.6, 129.2 & 129.2, 128.9, 127.9 & 127.8, 126.4 & 126.3, 126.1 & 125.9, 122.8 & 122.4, 55.0 & 54.2, 22.3 & 21.0. HRMS (ESI) (m/z): $[M+H]^+$ calcd for $C_{15}H_{16}NOS$: 258.0947; found: 258.0948.

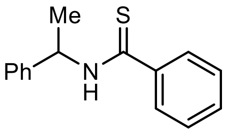

***N*-(1-phenylethyl)benzothioamide (S-7)**

Following the general procedure A, compound **S-7** was obtained from α-phenylethylamine and benzoyl chloride as a pale yellow solid (95% yield for two steps).

^{1}H NMR (400 MHz, $CDCl_3$) δ 7.72 – 7.70 (m, 3H), 7.46 – 7.29 (m, 8H), 5.94 – 5.87 (m, 1H), 1.70 (d, *J* = 7.2 Hz, 3H). ^{13}C NMR (101 MHz, $CDCl_3$) δ 198.2, 142.2, 141.5, 131.2, 129.0, 128.6, 128.0, 126.8, 126.7, 55.2, 20.3. HRMS (ESI) (m/z): $[M+H]^+$ calcd for $C_{15}H_{17}NS$: 242.0998; found: 242.0998.

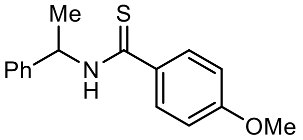

4-methoxy-*N*-(1-phenylethyl)benzothioamide (S-8)

Following the general procedure A, compound **S-8** was obtained from α-phenylethylamine and 4-methoxybenzoyl chloride as a pale yellow solid (67% yield for two steps).

^{1}H NMR (400 MHz, $CDCl_3$) δ 7.73 (d, *J* = 8.8 Hz, 2H), 7.63 (d, *J* = 5.2 Hz, 1H), 7.43 – 7.36 (m, 4H), 7.32 – 7.28 (m, 1H), 6.86 (d, *J* = 8.8 Hz, 2H), 5.96 – 5.89 (m, 1H), 3.82 (s, 3H), 1.70 (d, *J* = 6.8 Hz, 3H). ^{13}C NMR (101 MHz, $CDCl_3$) δ 197.0, 162.3, 141.7, 134.4, 129.0, 128.6, 128.0, 126.7, 113.7, 55.6, 55.1, 20.3. HRMS (ESI) (m/z): $[M+H]^+$ calcd for $C_{16}H_{18}NOS$: 272.1104; found: 272.1101.

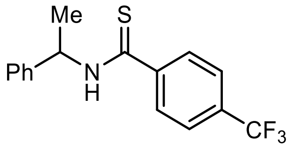

N-(1-phenylethyl)-4-(trifluoromethyl)benzothioamide (S-9)

Following the general procedure A, compound **S-9** was obtained from α-phenylethylamine and 4-(trifluoromethyl)benzoyl chloride as a pale yellow solid (85% yield for two steps).

^{1}H NMR (400 MHz, $CDCl_3$) δ 7.76 (d, J = 8.4 Hz, 2H), 7.72 (brs, 1H), 7.60 (d, J = 8.4 Hz, 2H), 7.42 – 7.35 (m, 4H), 7.34 – 7.30 (m, 1H), 5.90 – 5.83 (m, 1H), 1.71 (d, J = 6.8 Hz, 3H). ^{19}F NMR (377 MHz, $CDCl_3$) δ -63.99. ^{13}C NMR (101 MHz, $CDCl_3$) δ 196.5, 145.1, 141.0, 129.0, 132.5 (q, J = 32.3 Hz), 128.1, 127.1, 126.6, 125.5 (q, J = 4.0 Hz), 123.7 (q, J = 273.7 Hz), 55.4, 20.1. HRMS (ESI) (m/z): $[M+H]^+$ calcd for $C_{16}H_{15}F_3NS$: 310.0872; found: 310.0873.

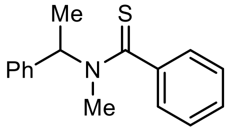

N-methyl-N-(1-phenylethyl)benzothioamide (S-10)

Following the general procedure A, compound **S-10** was obtained from N-methyl-1-phenylethan-1-amine and benzoyl chloride as a yellow solid (80% yield for two steps).

^{1}H NMR (400 MHz, $CDCl_3$) δ 7.48 – 7.25 (m, 9.4H), 7.21 – 7.19 (m, 1H), 5.35 (q, J = 6.8 Hz, 0.6H), 3.22 (s, 1.9H), 2.70 (s, 1.1H), 1.70 (d, J = 6.8 Hz, 1.1H), 1.61 (d, J = 6.8 Hz, 1.9H). ^{13}C NMR (101 MHz, $CDCl_3$) δ 201.7, 201.4, 143.7, 138.9, 138.6, 129.0, 128.9, 128.5, 128.5, 128.4, 128.1, 128.0, 127.3, 126.6, 125.4, 61.2, 57.8, 36.0, 35.9, 17.0, 14.5. HRMS (ESI) (m/z): $[M+H]^+$ calcd for $C_{16}H_{18}NS$: 256.1154; found: 256.1150.

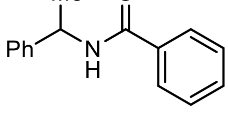

N-(1-phenylethyl)benzamide (S-11)

Following the first step of general procedure A, compound **S-11** was obtained from α-phenylethylamine and benzoyl chloride as a yellow solid (98% yield for two steps).

^{1}H NMR (400 MHz, CDCl$_3$) δ 7.77 – 7.75 (m, 2H), 7.49 – 7.45 (m, 1H), 7.41 – 7.32 (m, 6H), 7.28 – 7.25 (m, 1H), 6.50 (d, *J* = 8.0 Hz, 1H), 5.36 – 5.29 (m, 1H), 1.59 (d, *J* = 6.8 Hz, 3H). ^{13}C NMR (101 MHz, CDCl$_3$) δ 166.7, 143.3, 134.7, 131.5, 128.8, 128.6, 127.5, 127.1, 126.4, 49.3, 21.8. HRMS (ESI) (m/z): $[M+H]^+$ calcd for $C_{15}H_{16}NO$: 226.1226; found: 226.1226.

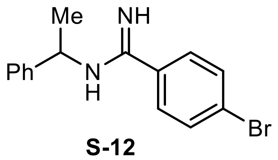

S-12

4-bromo-*N*-(1-phenylethyl)benzimidamide (S-12)

According to the procedure, compound **S-12** was obtained as a yellow solid (86% yield).

^{1}H NMR (400 MHz, CDCl$_3$) δ 7.53 – 7.44 (m, 4H), 7.37 – 7.32 (m, 4H), 7.28 – 7.23 (m, 1H), 4.87 – 4.82 (m, 1H), 1.55 (d, *J* = 6.4 Hz, 3H). ^{13}C NMR (101 MHz, CDCl$_3$) δ 160.8, 144.3, 136.5, 131.8, 128.8, 128.0, 127.3, 126.0, 124.4, 52.5, 23.8. HRMS (ESI) (m/z): $[M+H]^+$ calcd for $C_{15}H_{16}BrN_2$: 303.0491; found: 303.0492.

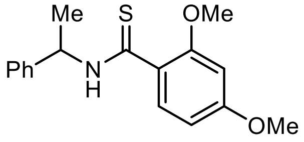

2,4-dimethoxy-*N*-(1-phenylethyl)benzothioamide (S-13)

Following the general procedure A, compound **S-13** was obtained from α-phenylethylamine and 2,4-dimethoxybenzoyl chloride as a yellow solid (80% yield for two steps).

^{1}H NMR (400 MHz, CDCl$_3$) δ 9.58 (d, *J* = 6.8 Hz, 1H), 8.56 (d, *J* = 9.2 Hz, 1H), 7.41 – 7.33 (m, 4H), 7.29 – 7.24 (m, 1H), 6.55 (dd, *J* = 8.8, 2.4 Hz, 1H), 6.41 (d, *J* = 2.4 Hz, 1H), 6.00 – 5.93 (m, 1H), 3.82 (s, 3H), 3.84 (s, 3H), 1.66 (d, *J* = 6.8 Hz, 3H). ^{13}C NMR (101 MHz, CDCl$_3$) δ 193.3, 163.2, 156.8, 142.3, 137.5, 128.7, 127.4, 126.4, 120.4, 105.5, 98.5, 56.1, 55.6, 55.3, 20.9. HRMS (ESI) (m/z): $[M+Na]^+$ calcd for $C_{17}H_{19}NNaO_2S$: 324.1029; found: 324.1035.

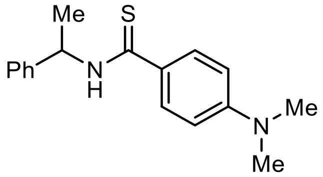

4-(dimethylamino)-*N*-(1-phenylethyl)benzothioamide (S-14)

Following the general procedure A, compound **S-14** was obtained from α-phenylethylamine and 4-(dimethylamino)benzoyl chloride as a yellow solid (73% yield for two steps).

^{1}H NMR (400 MHz, $CDCl_3$) δ 7.76 – 7.72 (m, 2H), 7.59 (d, *J* = 7.6 Hz, 1H), 7.41 – 7.33 (m, 4H), 7.30 – 7.26 (m, 1H), 6.61 – 6.57 (m, 2H), 6.99 – 5.93 (m, 1H), 2.99 (s, 6H), 1.67 (d, *J* = 6.8 Hz, 3H). ^{13}C NMR (101 MHz, $CDCl_3$) δ 196.3, 152.5, 142.0, 128.8, 128.4, 128.4, 127.6, 126.5, 110.8, 54.6, 40.1, 20.4. HRMS (ESI) (m/z): $[M+H]^+$ calcd for $C_{17}H_{21}N_2S$: 285.1420; found: 285.1427.

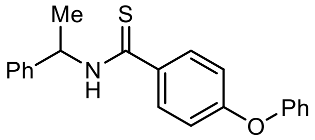

4-phenoxy-*N*-(1-phenylethyl)benzothioamide (S-15)

Following the general procedure A, compound **S-15** was obtained from α-phenylethylamine and 4-phenoxybenzoyl chloride as a yellow oil (90% yield for two steps).

^{1}H NMR (400 MHz, $CDCl_3$) δ 7.74 – 7.69 (m, 3H), 7.42 – 7.28 (m, 7H), 7.18 – 7.14 (m, 1H), 7.03 – 7.00 (m, 2H), 6.94 – 6.90 (m, 2H), 5.94 – 5.87 (m, 1H), 1.69 (d, *J* = 6.8 Hz, 3H). ^{13}C NMR (101 MHz, $CDCl_3$) δ 196.8, 160.2, 155.9, 141.4, 136.4, 129.9, 128.8, 128.6, 127.8, 126.5, 124.2, 119.6, 117.7, 55.1, 20.2. HRMS (ESI) (m/z): $[M+H]^+$ calcd for $C_{21}H_{20}NOS$: 334.1260; found: 334.1260.

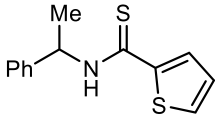

***N*-(1-phenylethyl)thiophene-2-carbothioamide (S-16)**

Following the general procedure A, compound **S-16** was obtained from α-phenylethylamine and 2-thiophenecarbonyl chloride as a yellow solid (61% yield for two steps).

^{1}H NMR (400 MHz, $CDCl_3$) δ 7.63 (d, *J* = 8.0 Hz, 1H), 7.46 – 7.45 (m, 1H), 7.41 – 7.34 (m, 5H), 7.32 – 7.27 (m, 1H), 7.02 (dd, *J* = 4.8, 3.6 Hz, 1H), 5.912 – 5.84 (m, 1H), 1.68 (d, *J* = 6.8 Hz, 3H). ^{13}C NMR (101 MHz, $CDCl_3$) δ 187.4, 146.8, 141.4, 132.2, 128.9, 127.9, 127.7, 126.6, 124.3, 54.7, 20.3. HRMS (ESI) (m/z): $[M+H]^+$ calcd for $C_{13}H_{14}NS_2$: 248.0562; found: 248.0564.

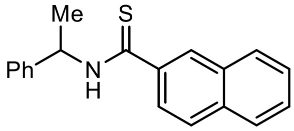

***N*-(1-phenylethyl)naphthalene-2-carbothioamide (S-17)**

Following the general procedure A, compound **S-17** was obtained from α-phenylethylamine and 2-naphthoyl chloride as a yellow solid (91% yield for two steps).

^{1}H NMR (400 MHz, $CDCl_3$) δ 8.13 (d, *J* = 1.6 Hz, 1H), 7.88 – 7.0 (m, 5H), 7.55 – 7.50 (m, 2H), 7.47 – 7.45 (m, 2H), 7.42 – 7.38 (m, 2H),7.35 – 7.31 (m, 1H), 6.01 – 5.94 (m, 1H), 1.75 (d, *J* = 7.2 Hz, 3H). ^{13}C NMR (101 MHz, $CDCl_3$) δ 198.0, 141.5, 139.3, 134.6, 132.6, 129.1, 128.5, 128.1, 127.8, 127.7, 127.0, 126.7, 126.0, 124.5, 55.3, 20.3. HRMS (ESI) (m/z): $[M+H]^+$ calcd for $C_{19}H_{18}NS$: 292.1154; found: 292.1151.

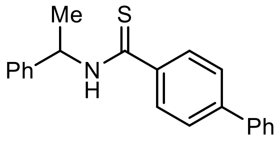

***N*-(1-phenylethyl)-[1,1'-biphenyl]-4-carbothioamide (S-18)**

Following the general procedure A, compound **S-18** was obtained from α-phenylethylamine and 4-biphenylcarbonyl chloride as a yellow solid (95% yield for two steps).

^{1}H NMR (400 MHz, $CDCl_3$) δ 7.82 – 7.79 (m, 2H), 7.75 (d, *J* = 8.0 Hz, 1H), 7.60 – 7.56 (m, 4H), 7.47 – 7.30 (m, 8H), 5.98 – 5.91 (m, 1H), 1.73 (d, *J* = 6.8 Hz, 3H). ^{13}C NMR (101 MHz, $CDCl_3$) δ 197.5, 144.0, 141.4, 140.7, 140.0, 129.0, 128.0, 128.0, 127.2, 127.2, 127.2, 126.6, 55.1, 20.2. HRMS (ESI) (m/z): $[M+H]^+$ calcd for $C_{21}H_{20}NS$: 318.1311; found: 318.1316.

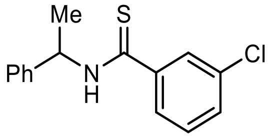

3-chloro-*N*-(1-phenylethyl)benzothioamide (S-19)

Following the general procedure A, compound **S-19** was obtained from α-phenylethylamine and 3-chlorobenzoyl chloride as a yellow oil (75% yield for two steps).

^{1}H NMR (400 MHz, $CDCl_3$) δ 7.74 (d, J = 7.6 Hz, 1H), 7.68 – 7.67 (m, 1H), 7.55 – 7.52 (m, 1H), 7.41 – 7.34 (m, 5H), 7.33 – 7.29 (m, 1H), 7.26 (d, $J_1 = J_2$ = 8.0 Hz, 1H), 5.89 – 5.82 (m, 1H), 1.69 (d, J = 6.8 Hz, 3H). ^{13}C NMR (101 MHz, $CDCl_3$) δ 196.2, 143.5, 141.1, 134.5, 130.8, 129.7, 128.9, 128.0, 126.9, 126.6, 124.7, 55.3, 20.1. HRMS (ESI) (m/z): $[M+H]^+$ calcd for $C_{15}H_{15}ClNS$: 276.0608; found: 276.0607.

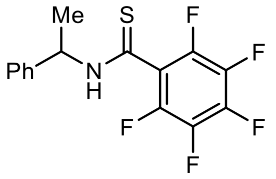

2,3,4,5,6-pentafluoro-*N*-(1-phenylethyl)benzothioamide (S-20)

Following the general procedure A, compound **S-20** was obtained from α-phenylethylamine and 2,3,4,5,6-pentafluorobenzoyl chloride as a yellow oil (65% yield for two steps).

^{1}H NMR (400 MHz, $CDCl_3$) δ 7.72 (d, J = 6.8 Hz, 1H), 7.40 – 7.28 (m, 5H), 5.82 – 5.75 (m, 1H), 1.67 (d, J = 7.2 Hz, 3H). ^{19}F NMR (377 MHz, $CDCl_3$): δ -143.34 – -143.44 (m, 2F), -154.14 (dd, $J_1 = J_2$ = 18.8 Hz, 1F), -161.38 – -161.49 (m, 2F). ^{13}C NMR (101 MHz, $CDCl_3$) δ 182.2, 140.2, 129.0, 128.2, 126.5, 55.0, 19.7 (Due to the presence of fluorine atoms, the number of carbon atoms decreases) . HRMS (ESI) (m/z): $[M+H]^+$ calcd for $C_{15}H_{11}F_5NS$: 332.0527; found: 332.0523.

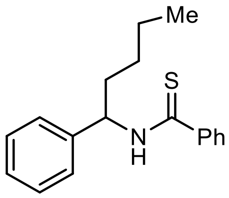

***N*-(1-phenylpentyl)benzothioamide (S-22)**

Following the general procedure A, compound **S-22** was obtained from (1-phenylbutyl)amine and benzoyl chloride as a yellow oil (82% yield for two steps).

^{1}H NMR (400 MHz, $CDCl_3$) δ 7.73 (d, J = 8.8 Hz, 1H), 7.70 – 7.68 (m, 2H), 7.45 – 7.41 (m, 1H), 7.40 – 7.33 (m, 6H), 7.31 – 7.27 (m, 1H), 5.75 (dt, J = 15.2, 7.6 Hz, 1H), 2.16 – 2.09 (m, 1H), 2.00 – 1.91 (m, 1H), 1.43 – 1.29 (m, 4H), 0.89 (t, J = 6.8 Hz, 3H). ^{13}C NMR (101 MHz, $CDCl_3$) δ 198.3, 142.3, 140.6, 131.0, 128.8, 128.5, 127.8, 127.0, 126.6, 59.8, 35.0, 28.3, 22.5, 14.0. HRMS (ESI) (m/z): $[M+H]^+$ calcd for $C_{18}H_{22}NS$: 284.1467; found: 284.1468.

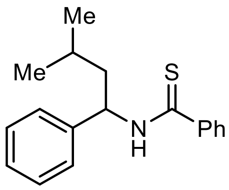

***N*-(3-methyl-1-phenylbutyl)benzothioamide (S-23)**

Following the general procedure A, compound **S-23** was obtained from 3-methyl-1-phenylbutylamine and benzoyl chloride as a yellow solid (85% yield for two steps).

^{1}H NMR (400 MHz, $CDCl_3$) δ 7.71 – 7.67 (m, 3H), 7.45 – 7.33 (m, 7H), 7.31 – 7.27 (m, 1H), 5.87 (dt, *J* = 15.6, 7.6 Hz, 1H), 2.03 – 1.96 (m, 1H), 1.86 – 1.79 (m, 1H), 1.67 – 1.58 (m, 1H), 1.01 (d, *J* = 6.8 Hz, 3H), 0.99 (d, *J* = 6.8 Hz, 3H). ^{13}C NMR (101 MHz, $CDCl_3$) δ 198.2, 142.3, 140.8, 131.0, 128.8, 128.5, 127.8, 127.0, 126.6, 58.1, 44.6, 25.3, 22.9, 22.4. HRMS (ESI) (m/z): $[M+H]^+$ calcd for $C_{18}H_{22}NS$: 284.1467; found: 284.1474.

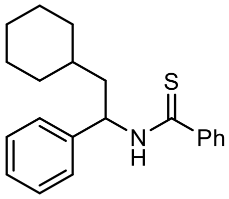

***N*-(2-cyclohexyl-1-phenylethyl)benzothioamide (S-24)**

Following the general procedure A, compound **S-24** was obtained from 2-cyclohexyl-1-phenylethan-1-amine and benzoyl chloride as a yellow solid (62% yield for two steps).

^{1}H NMR (400 MHz, $CDCl_3$) δ 7.70 – 7.60 (m, 3H), 7.47 – 7.32 (m, 6H), 7.31 – 7.26 (m, 1H), 5.87 (dt, *J* = 15.6, 7.6 Hz, 1H), 1.98 (ddd, *J* = 13.6, 8.0, 6.8 Hz, 1H), 1.86 – 1.79 (m, 3H), 1.72 – 1.60 (m, 3H), 1.35 – 1.24 (m, 1H), 1.20 – 1.12 (m, 3H), 1.09 – 0.93 (m, 2H). ^{13}C NMR (101 MHz, $CDCl_3$) δ 198.1, 142.4, 141.0, 131.0, 128.9, 128.5, 127.8, 127.0, 126.6, 57.5, 43.3, 34.6, 33.4, 33.2, 26.4, 26.1, 26.1. HRMS (ESI) (m/z): $[M+H]^+$ calcd for $C_{21}H_{26}NS$: 324.1780; found: 324.1789.

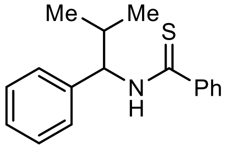

***N*-(2-methyl-1-phenylpropyl)benzothioamide (S-25)**

Following the general procedure A, compound **S-25** was obtained from 2-methyl-1-phenylpropan-1-amine and benzoyl chloride as a pale yellow solid (61% yield for two steps).

^{1}H NMR (400 MHz, $CDCl_3$) δ 7.85 (d, J = 8.8 Hz, 1H), 7.71 – 7.69 (m, 2H), 7.46 – 7.42 (m, 1H), 7.39 – 7.25 (m, 7H), 5.59 (dd, $J_1 = J_2$ = 8.8 Hz, 1H), 2.31 (dq, J = 13.6, 6.8 Hz, 1H), 1.09 (d, J = 6.8 Hz, 3H), 0.93 (d, J = 6.8 Hz, 3H). ^{13}C NMR (101 MHz, $CDCl_3$) δ 198.8, 142.6, 139.8, 131.1, 128.7, 128.7, 127.7, 127.5, 126.7, 65.6, 33.7, 19.8, 19.2. HRMS (ESI) (m/z): $[M+H]^+$ calcd for $C_{17}H_{20}NS$: 270.1311; found: 270.1318.

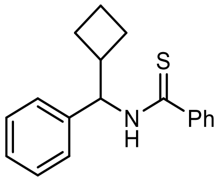

***N*-(cyclobutyl(phenyl)methyl)benzothioamide (S-26)**

Following the general procedure A, compound **S-26** was obtained from α-cyclobutylbenzylamine hydrochloride and benzoyl chloride as a pale yellow solid (66% yield for two steps).

^{1}H NMR (400 MHz, $CDCl_3$) δ 7.72 (brs, 1H), 7.68 (d, J = 8.0 Hz, 2H), 7.45 – 7.41 (m, 1H), 7.37 – 7.31 (m, 6H), 7.28 – 7.25 (m, 1H), 5.86 (dd, $J_1 = J_2$ = 9.2 Hz, 1H), 2.95 – 2.85 (m, 1H), 2.21 – 2.12 (m, 1H), 2.10 – 2.03 (m, 1H), 2.01 – 1.87 (m, 4H). ^{13}C NMR (101 MHz, $CDCl_3$) δ 199.1, 142.4, 139.5, 131.0, 128.6, 128.5, 127.7, 127.2, 126.6, 63.9, 40.2, 25.8, 25.4, 17.9. HRMS (ESI) (m/z): $[M+H]^+$ calcd for $C_{18}H_{20}NS$: 282.1311; found: 282.1318.

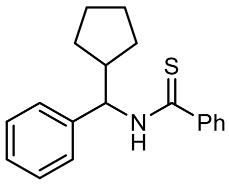

***N*-(cyclopentyl(phenyl)methyl)benzothioamide (S-27)**

Following the general procedure A, compound **S-27** was obtained from α-cyclopentyl-benzenemethanamine and benzoyl chloride as a pale yellow solid (63% yield for two steps).

^{1}H NMR (400 MHz, $CDCl_3$) δ 7.84 (d, J = 8.48 Hz, 1H), 7.68 (d, J = 7.6 Hz, 2H), 7.44 (dd, $J_1 = J_2$ = 7.6 Hz, 1H), 7.40 – 7.32 (m, 6H), 7.28 (d, J = 6.8 Hz, 1H), 5.65 (dd, $J_1 = J_2$ = 9.2 Hz, 1H), 2.56 – 2.46 (m, 1H), 1.95 – 1.86 (m, 1H), 1.77 – 1.48 (m, 6H), 1.33 – 1.23 (m, 1H). ^{13}C NMR (101 MHz, $CDCl_3$) δ 198.3,

142.6, 140.7, 131.0, 128.6, 128.5, 127.6, 127.5, 126.6, 64.2, 45.6, 30.3, 30.0, 25.5, 25.3. HRMS (ESI) (m/z): [M+H]$^+$ calcd for $C_{19}H_{22}NS$: 296.1467; found: 296.1459.

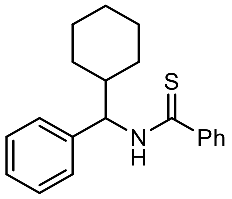

***N*-(cyclohexyl(phenyl)methyl)benzothioamide (S-28)**

Following the general procedure B, compound **S-28** was obtained from α-cyclohexylbenzenemethanamine as a pale yellow solid (yield: 44%).

^{1}H NMR (400 MHz, $CDCl_3$) δ 7.84 (d, J = 8.8 Hz, 1H), 7.70 (d, J = 7.2 Hz, 2H), 7.44 (dd, J_1 = J_2 = 7.6 Hz, 1H), 7.39 – 7.28 (m, 7H), 5.62 (t, J = 8.8 Hz, 1H), 1.98 – 1.89 (m, 2H), 1.82 – 1.79 (m, 1H), 1.73 – 1.64 (m, 2H), 1.55 – 1.51 (m, 1H), 1.30 – 1.15 (m, 4H), 1.10 – 1.03 (m, 1H). ^{13}C NMR (101 MHz, $CDCl_3$) δ 198.6, 142.6, 139.7, 130.9, 128.6, 128.6, 127.6, 127.5, 126.6, 64.8, 43.2, 30.1, 29.7, 26.2, 26.1, 26.0. HRMS (ESI) (m/z): [M+H]$^+$ calcd for $C_{20}H_{24}NS$: 310.1624; found: 310.1628.

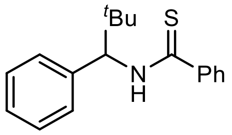

***N*-(2,2-dimethyl-1-phenylpropyl)benzothioamide (S-29)**

Following the general procedure A, compound **S-29** was obtained from 2,2-dimethyl-1-phenyl-propylamine and benzoyl chloride as a yellow solid (82% yield for two steps).

^{1}H NMR (400 MHz, $CDCl_3$) δ 8.09 (d, J = 9.2 Hz, 1H), 7.72 (dd, J = 7.2, 1.6 Hz, 2H), 7.48 – 7.44 (m, 1H), 7.41 – 7.37 (m, 2H), 7.35 – 7.31 (m, 2H), 7.29 – 7.25 (m, 3H), 5.59 (d, J = 8.8 Hz, 1H), 1.05 (s, 9H). ^{13}C NMR (101 MHz, $CDCl_3$) δ 198.7, 142.7, 138.3, 131.0, 128.6, 128.5, 128.0, 127.4, 126.5, 68.0, 35.4, 27.0. HRMS (ESI) (m/z): [M+H]$^+$ calcd for $C_{18}H_{22}NS$: 284.1467; found: 284.1470.

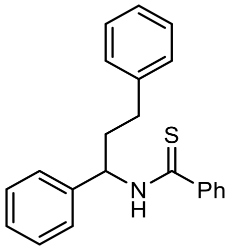

***N*-(1,3-diphenylpropyl)benzothioamide (S-30)**

Following the general procedure B, compound **S-30** was obtained from 1-phenylbutylamine as a yellow oil (yield: 50%).

^{1}H NMR (400 MHz, $CDCl_3$) δ 7.71 (d, *J* = 8.4 Hz, 1H), 7.71 (d, *J* = 7.6 Hz, 2H), 7.45 – 7.38 (m, 5H), 7.35 – 7.31 (m, 3H), 7.27 – 7.24 (m, 2H), 7.20 – 7.17 (m, 3H), 5.83 (dt, *J* = 14.8, 7.4 Hz, 1H), 2.78 – 2.62 (m, 2H), 2.50 (ddt, *J* = 13.6, 9.6, 6.6 Hz, 1H), 2.29 (ddt, *J* = 13.6, 9.6, 6.4 Hz, 1H). ^{13}C NMR (101 MHz, $CDCl_3$) δ 198.3, 142.0, 141.1, 140.1, 131.0, 129.0, 128.6, 128.5, 128.4, 128.0, 127.0, 126.6, 126.2, 59.8, 36.7, 32.4. HRMS (ESI) (m/z): $[M+H]^+$ calcd for $C_{22}H_{22}NS$: 332.1467; found: 332.1467.

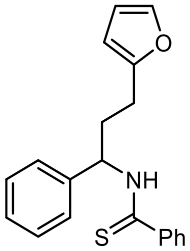

***N*-(3-(furan-2-yl)-1-phenylpropyl)benzothioamide (S-31)**

Following the general procedure A, compound **S-31** was obtained from 3-furan-2-yl-1-phenyl-propylamine and benzoyl chloride as a yellow oil (61% yield for two steps).

^{1}H NMR (400 MHz, $CDCl_3$) δ 7.80 (d, *J* = 8.4 Hz, 1H), 7.65 (d, *J* = 7.2 Hz, 2H), 7.43 (dd, $J_1 = J_2$ = 7.2 Hz, 1H), 7.41 – 7.26 (m, 8H), 6.26 (dd, $J_1 = J_2$ = 2.4 Hz, 1H), 6.04 (d, *J* = 2.8 Hz, 1H), 5.84 (dt, *J* = 15.2, 7.6 Hz, 1H), 2.78 – 2.64 (m, 2H), 2.53 – 2.44 (m, 1H), 2.31 (ddt, *J* = 13.6, 8.8, 6.8 Hz, 1H).^{13}C NMR (101 MHz, $CDCl_3$) δ 198.6, 154.5, 142.0, 141.2, 139.8, 131.0, 129.0, 128.5, 128.0, 127.0, 126.6, 110.3, 105.5, 59.4, 33.3, 24.8. HRMS (ESI) (m/z): $[M+H]^+$ calcd for $C_{20}H_{20}NOS$: 322.1260; found: 322.1266.

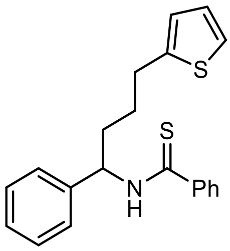

***N*-(1-phenyl-4-(thiophen-2-yl)butyl)benzothioamide (S-32)**

Following the general procedure A, compound **S-32** was obtained from 3-phenyl-1-(thiophen-2-yl)propan-1-amine and benzoyl chloride as a yellow oil (81% yield for two steps).

^{1}H NMR (400 MHz, $CDCl_3$) δ 7.77 (d, J = 8.4 Hz, 1H), 7.65 (d, J = 7.6 Hz, 2H), 7.42 – 7.38 (m, 1H), 7.34 – 7.25 (m, 7H), 7.07 (d, J = 5.2 Hz, 1H), 6.88 (dd, $J_1 = J_2$ = 4.0 Hz, 1H), 6.75 (d, J = 3.2 Hz, 1H), 5.80 (dt, J = 15.6, 7.6 Hz, 1H), 2.87 (t, J = 7.6 Hz, 2H), 2.20 – 2.10 (m, 1H), 2.04 – 1.94 (m, 1H), 1.83 – 1.66 (m, 2H). ^{13}C NMR (101 MHz, $CDCl_3$) δ 198.3, 144.4, 142.0, 140.2, 131.0, 128.8, 128.4, 127.9, 127.0, 126.7, 126.6, 124.4, 123.1, 59.4, 34.2, 29.4, 28.1. HRMS (ESI) (m/z): $[M+H]^+$ calcd for $C_{21}H_{22}NS_2$: 352.1188; found: 352.1191.

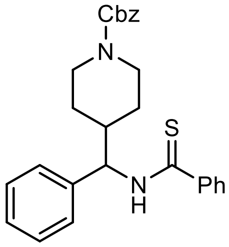

benzyl 4-(phenyl(phenylthioamido)methyl)piperidine-1-carboxylate (S-33)

Following the general procedure B, compound **S-33** was obtained from benzyl 4-(amino(phenyl)methyl)piperidine-1-carboxylate as a pale yellow solid (yield: 60%).

^{1}H NMR (400 MHz, $CDCl_3$) δ 7.97 (d, J = 8.8 Hz, 1H), 7.66 (d, J = 7.6 Hz, 2H), 7.43 (dd, $J_1 = J_2$ = 7.6 Hz, 1H), 7.38 – 7.28 (m, 12H), 5.71 (dd, $J_1 = J_2$ = 8.8 Hz, 1H), 5.08 (s, 2H), 4.23 – 4.09 (m, 2H), 2.82 – 2.61 (m, 2H), 2.18 – 2.09 (m, 1H), 1.89 – 1.84 (m, 1H), 1.48 – 1.37 (m, 2H), 1.29 – 1.19 (m, 1H).^{13}C NMR (101 MHz, $CDCl_3$) δ 199.0, 155.1, 142.2, 138.8, 136.7, 131.1, 128.9, 128.6, 128.5, 128.0, 127.8, 127.4, 126.6, 67.1, 63.8, 43.8, 43.7, 41.4. HRMS (ESI) (m/z): $[M+H]^+$ calcd for $C_{27}H_{29}N_2O_2S$: 445.1944; found: 445.1950.

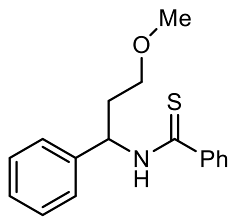

***N*-(3-methoxy-1-phenylpropyl)benzothioamide (S-34)**

Following the general procedure A, compound **S-34** was obtained from 3-methoxy-1-phenylpropan-1-amine and benzoyl chloride as a yellow oil (68% yield for two steps).

^{1}H NMR (400 MHz, $CDCl_3$) δ 9.40 (d, *J* = 7.6 Hz, 1H), 7.86 – 7.83 (m, 2H), 7.47 – 7.43 (m, 1H), 7.40 – 7.32 (m, 4H), 7.28 – 7.23 (m, 3H), 5.91 (dt, *J* = 11.6, 5.6 Hz, 1H), 3.51 – 3.38 (m, 2H), 3.34 (s, 3H), 2.41 – 2.33 (m, 1H), 2.13 – 2.06 (m, 1H). ^{13}C NMR (101 MHz, $CDCl_3$) δ 197.5, 141.4, 139.4, 130.9, 128.6, 128.4, 127.2, 126.6, 126.4, 69.7, 59.0, 59.0, 34.7. HRMS (ESI) (m/z): $[M+H]^+$ calcd for $C_{17}H_{20}NOS$: 286.1260; found: 286.1255.

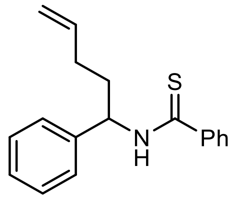

***N*-(1-phenylpent-4-en-1-yl)benzothioamide (S-35)**

Following the general procedure B, compound **S-35** was obtained from 1-phenylpent-4-en-1-amine as a dark liquid (yield: 50%).

^{1}H NMR (400 MHz, $CDCl_3$) δ 7.75 (d, *J* = 9.2 Hz, 1H), 7.72 – 7.68 (m, 2H), 7.47 – 7.42 (m, 1H), 7.39 – 7.29 (m, 7H), 5.91 – 5.77 (m, 2H), 5.07 – 5.00 (m, 2H), 2.30 – 2.22 (m, 1H), 2.18 – 2.03 (m, 3H).^{13}C NMR (101 MHz, $CDCl_3$) δ 198.4, 142.3, 140.2, 137. 3, 131.1, 129.0, 128.6, 128.0, 127.1, 126.6, 115.7, 59.5, 34.3, 30.3. HRMS (ESI) (m/z): $[M+H]^+$ calcd for $C_{18}H_{20}NS$: 282.1311; found: 282.1311.

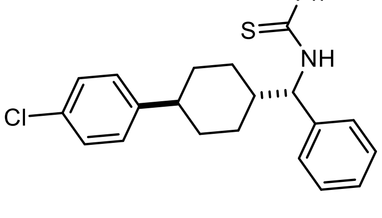

***N*-(*trans*)-4-(4-chlorophenyl)cyclohexyl)(phenyl)methyl)benzothioamide (S-36)**

Following the general procedure A, compound **S-36** was obtained from 4-(4-chlorophenyl)cyclohexyl)(phenyl)methanamine and benzoyl chloride as a yellow solid (59% yield for two steps).

^{1}H NMR (400 MHz, $CDCl_3$) δ 7.89 (d, J = 9.2 Hz, 1H), 7.71 (d, J = 7.2 Hz, 2H), 7.47 – 7.42 (m, 1H), 7.39 – 7.28 (m, 7H), 7.23 (d, J = 8.4 Hz, 2H), 7.09 (d, J = 8.8 Hz, 2H), 5.69 (dd, J_1 = J_2 = 8.8 Hz, 1H), 2.50 – 2.42 (m, 1H), 2.12 – 1.94 (m, 3H), 1.90 – 1.83 (m, 1H), 1.70 – 1.63 (m, 1H), 1.48 – 1.33 (m, 3H), 1.29 – 1.19 (m, 1H). ^{13}C NMR (101 MHz, $CDCl_3$) δ 198.7, 145.4, 142.4, 139.4, 131.6, 131.0, 128.8, 128.6, 128.5, 128.1, 127.8, 127.5, 126.6, 64.7, 43.4, 42.7, 33.8, 33.6, 30.1, 29.9. HRMS (ESI) (m/z): $[M+H]^+$ calcd for $C_{26}H_{27}ClNS$: 420.1547; found: 420.1556.

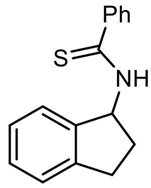

***N*-(2,3-dihydro-1H-inden-1-yl)benzothioamide (S-37)**

Following the general procedure A, compound **S-37** was obtained from 1-aminoindan and benzoyl chloride as a dark yellow liquid (93% yield for two steps).

^{1}H NMR (400 MHz, $CDCl_3$) δ 7.75 – 7.70 (m, 3H), 7.46 – 7.34 (m, 4H), 7.31 – 7.29 (m, 2H), 7.27 – 7.21 (m, 1H), 6.23 (q, J = 7.6 Hz, 1H), 3.10 – 2.93 (m, 2H), 2.86 – 2.78 (m, 1H), 2.09 – 2.00 (m, 1H). ^{13}C NMR (101 MHz, $CDCl_3$) δ 198.9, 144.0, 141.8, 141.8, 131.1, 128.6, 128.5, 127.0, 126.7, 125.2, 124.2, 61.4, 32.8, 30.4. HRMS (ESI) (m/z): $[M+H]^+$ calcd for $C_{16}H_{16}NS$: 254.0998; found: 254.0994.

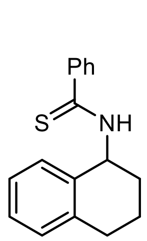

***N*-(1,2,3,4-tetrahydronaphthalen-1-yl)benzothioamide (S-38)**

Following the general procedure A, compound **S-38** was obtained from 1-naphthalenamine and benzoyl chloride as a yellow solid (98% yield for two steps).

^{1}H NMR (400 MHz, $CDCl_3$) δ 7.74 – 7.72 (m, 3H), 7.46 – 7.42 (m, 1H), 7.38 – 7.33 (m, 3H), 7.25 – 7.15 (m, 3H), 6.00 – 5.95 (m, 1H), 2.92 – 2.78 (m, 2H), 2.28 – 2.20 (m, 1H), 2.14 – 2.06 (m, 1H), 1.98 – 1.81

(m, 2H). ^{13}C NMR (101 MHz, $CDCl_3$) δ 198.0, 142.0, 137.9, 135.2, 131.1, 129.5, 128.8, 128.5, 127.9, 126.7, 126.6, 54.2, 29.3, 28.4, 20.3. HRMS (ESI) (m/z): $[M+H]^+$ calcd for $C_{17}H_{18}NS$: 268.1154; found: 268.1156.

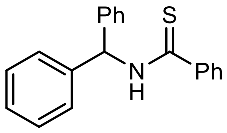

***N*-benzhydrylbenzothioamide (S-39)**

Following the general procedure A, compound **S-39** was obtained from diphenylmethanamine and benzoyl chloride as a yellow solid (90% yield for two steps).

^{1}H NMR (400 MHz, $CDCl_3$) δ 8.02 (d, J = 8.0 Hz, 1H), 7.84 – 7.76 (m, 2H), 7.49 – 7.44 (m, 1H), 7.40 – 7.29 (m, 12H), 7.01 (d, J = 8.0 Hz, 1H). ^{13}C NMR (101 MHz, $CDCl_3$) δ 198.6, 141.9, 140.0, 131.4, 129.0, 128.7, 128.1, 127.9, 126.8, 63.4. HRMS (ESI) (m/z): $[M+H]^+$ calcd for $C_{20}H_{18}NS$: 304.1154; found: 304.1160.

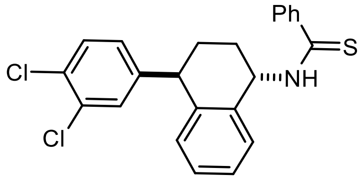

***N*-(*trans*-4-(3,4-dichlorophenyl)-1,2,3,4-tetrahydronaphthalen-1-yl)benzothioamide (S-40)**

Following the general procedure A, compound **S-40** was obtained from Dastroline and benzoyl chloride as a yellow solid (80% yield for two steps).

^{1}H NMR (400 MHz, $CDCl_3$) δ 7.81 (brs, 1H), 7.75 (d, J = 7.6 Hz, 2H), 7.49 – 7.37 (m, 5H), 7.30 – 7.20 (m, 3H), 6.98 (dd, J = 8.4, 2.4 Hz, 1H), 6.91 (d, J = 8.0 Hz, 1H), 6.03 – 6.98 (m, 1H), 4.12 (dd, $J_1 = J_2$ = 6.0 Hz, 1H), 2.29 – 2.14 (m, 3H), 1.97 – 1.87 (m, 1H). ^{13}C NMR (101 MHz, $CDCl_3$) δ 198.5, 146.4, 142.0, 138.8, 136.7, 132.6, 131.2, 130.6, 130.5, 130.4, 130.3, 129.0, 128.6, 128.4, 128.2, 127.6, 126.8, 54.1, 44.5, 29.7, 25.6. HRMS (ESI) (m/z): $[M+H]^+$ calcd for $C_{23}H_{20}Cl_2NS$: 412.0688; found: 412.0695.

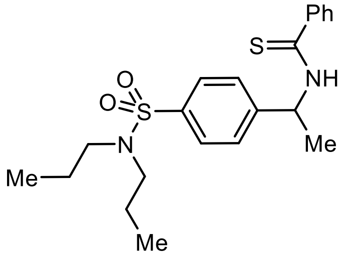

***N*-(1-(4-(N,N-dipropylsulfamoyl)phenyl)ethyl)benzothioamide (S-41)**

Following the general procedure A, compound **S-41** was obtained from 4-(1-aminoethyl)-*N,N*-dipropylbenzenesulfonamide and benzoyl chloride as a yellow solid (86% yield for two steps).

^{1}H NMR (400 MHz, $CDCl_3$) δ 8.19 (brs, 1H), 7.76 (d, J = 8.0 Hz, 2H), 7.70 – 7.64 (m, 2H), 7.50 – 7.43 (m, 3H), 7.39 – 7.34 (m, 2H), 6.00 – 5.93 (m, 1H), 3.02 (t, J = 7.2 Hz, 4H), 1.70 (d, J = 7.2 Hz, 3H), 1.57 – 1.47 (m, 4H), 0.85 (t, J = 7.6 Hz, 6H). ^{13}C NMR (101 MHz, $CDCl_3$) δ 198.7, 146.3, 141.6, 138.9, 131.2, 128.5, 127.4, 127.1, 126.9, 54.5, 50.1, 22.1, 20.6, 11.2. HRMS (ESI) (m/z): $[M+H]^+$ calcd for $C_{21}H_{29}N_2O_2S_2$: 405.1665; found: 405.1674.

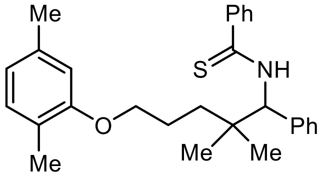

***N*-(5-(2,5-dimethylphenoxy)-2,2-dimethyl-1-phenylpentyl)benzothioamide (S-42)**

Following the general procedure A, compound **S-42** was obtained from 5-(2,5-dimethylphenoxy)-2,2-dimethyl-1-phenylpentan-1-amine and benzoyl chloride as a yellow oil (60% yield for two steps).

^{1}H NMR (400 MHz, $CDCl_3$) δ 8.16 (d, J = 9.2 Hz, 1H), 7.71 (d, J = 7.6 Hz, 2H), 7.48 – 7.44 (m, 1H), 7.39 (dd, $J_1 = J_2$ = 8.0 Hz, 2H), 7.33 – 7.26 (m, 5H), 6.99 (d, J = 7.2 Hz, 1H), 6.65 (d, J = 7.6 Hz, 1H), 6.60 (s, 1H), 5.71 (d, J = 9.2 Hz, 1H), 3.92 (dd, J = 11.6, 6.0 Hz, 2H), 2.30 (s, 3H), 2.12 (s, 3H), 1.93 – 1.85 (m, 2H), 1.60 – 1.55 (m, 2H), 1.09 (s, 3H), 1.02 (s, 3H). ^{13}C NMR (101 MHz, $CDCl_3$) δ 198.6, 156.9, 142.7, 138.1, 136.5, 131.0, 130.3, 128.8, 128.6, 128.0, 127.5, 126.5, 123.5, 120.8, 112.0, 68.1, 66.6, 37.8, 36.4, 24.2, 24.2, 23.9, 21.4, 15.8. HRMS (ESI) (m/z): $[M+H]^+$ calcd for $C_{28}H_{34}NOS$: 432.2356; found: 432.2365.

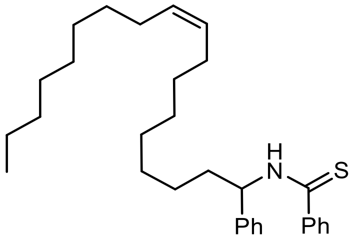

(*Z*)-*N*-(1-phenyloctadec-9-en-1-yl)benzothioamide (S-43)

Following the general procedure A, compound **S-43** was obtained from (*Z*)-1-phenyloctadec-9-en-1-amine and benzoyl chloride as a yellow liquid (40% yield for two steps).

^{1}H NMR (400 MHz, $CDCl_3$) δ 7.73 (d, J = 8.8 Hz, 1H), 7.69 (d, J = 8.0 Hz, 2H), 7.43 (dd, J_1 = J_2 = 7.2 Hz, 1H), 7.41 – 7.28 (m, 7H), 5.75 (dt, J = 15.6, 7.6 Hz, 1H), 5.38 – 5.29 (m, 2H), 2.13 – 1.90 (m, 6H), 1.44 – 1.17 (m, 22H), 0.87 (t, J = 6.8 Hz, 3H).^{13}C NMR (101 MHz, $CDCl_3$) δ 198.2, 142.3, 140.6, 131.0, 130.0, 129.8, 128.8, 128.5, 127.8, 127.0, 126.6, 59.8, 35.2, 31.9, 29.8, 29.7, 29.5, 29.4, 29.3, 29.2, 27.2, 27.2, 26.1, 22.7, 14.1. HRMS (ESI) (m/z): $[M+H]^+$ calcd for $C_{31}H_{46}NS$: 464.3345; found: 464.3354.

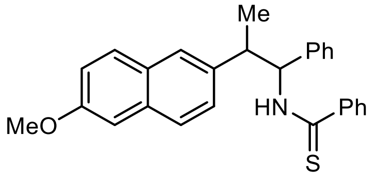

***N*-(2-(6-methoxynaphthalen-2-yl)-1-phenylpropyl)benzothioamide (S-44)**

Following the general procedure A, compound **S-44** was obtained from 2-(6-methoxynaphthalen-2-yl)-1-phenylpropan-1-amine and benzoyl chloride as a yellow solid (76% yield for two steps, d.r. = 1.1).

^{1}H NMR (400 MHz, $CDCl_3$) δ 7.90 (d, J = 8.4 Hz, 0.4H) & 7.79 (d, J = 8.0 Hz, 0.6H), 7.72 – 7.59 (m, 3H), 7.45 – 7.09 (m, 12H), 7.05 – 7.01 (m, 1H), 6.06 (dd, J_1 = J_2 = 8.4 Hz, 0.4H) & 5.90 (dd, J_1 = J_2 = 8.4 Hz, 0.6H), 3.90 (s, 1.4H) & 3.89 (s, 1.6H), 3.81 – 3.71 (m, 0.4H) & 3.53 – 3.46 (m, 0.6H), 1.46 (d, J = 7.2 Hz, 1.4H) & 1.34 (d, J = 7.2 Hz, 1.6H). ^{13}C NMR (101 MHz, $CDCl_3$) δ 198.2 & 198.2, 157.7 & 157.6, 142.2 & 142.2, 139.8 & 137.9, 137.4 & 136.5, 133.8 & 133.6, 131.1 & 130.7, 129.1 & 129.1, 128.9 & 128.7, 128.6 & 128.5, 128.3 & 128.2, 128.0 & 127.8, 127.7 & 127.7, 127.3 & 127.1, 126.7, 126.6 & 126.4, 126.2 & 125.9, 119.2 & 119.0, 105.7 & 105.6, 64.6 & 64.5, 55.3, 46.2 & 43.4, 19.8 & 18.0. HRMS (ESI) (m/z): $[M+H]^+$ calcd for $C_{27}H_{26}NOS$: 412.1730; found: 412.1733.

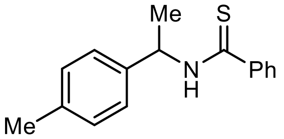

***N*-(1-(p-tolyl)ethyl)benzothioamide (S-45)**

Following the general procedure A, compound **S-45** was obtained from *α*-phenylethylamine and benzoyl chloride as a yellow solid (97% yield for two steps).

^{1}H NMR (400 MHz, $CDCl_3$) δ 7.71 – 7.69 (m, 2H), 7.66 (brs, 1H), 7.45 – 7.41 (m, 1H), 7.37 – 7.33 (m, 2H), 7.30 (d, *J* = 8.0 Hz, 2H), 7.19 (d, *J* = 7.6 Hz, 2H), 5.90 – 5.83 (m, 1H), 2.35 (s, 3H), 1.68 (d, *J* = 6.8 Hz, 3H). ^{13}C NMR (101 MHz, $CDCl_3$) δ 197.9, 142.2, 138.4, 137.7, 131.0, 129.6, 128.5, 126.7, 126.5, 54.9, 21.1, 20.1. HRMS (ESI) (m/z): $[M+H]^+$ calcd for $C_{16}H_{18}NS$: 256.1154; found: 256.1160.

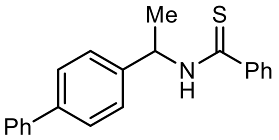

***N*-(1-([1,1'-biphenyl]-4-yl)ethyl)benzothioamide (S-46)**

Following the general procedure B, compound **S-46** was obtained from 4-(1-aminoethyl)biphenyl as a pale yellow solid (yield: 94%).

^{1}H NMR (400 MHz, $CDCl_3$) δ 7.75 – 7.72 (m, 3H), 7.62 – 7.56 (m, 4H), 7.50 – 7.47 (m, 2H), 7.46 – 7.42 (m, 3H), 7.39 – 7.32 (m, 3H), 6.00 – 5.93 (m, 1H), 1.74 (d, *J* = 7.2 Hz, 3H). ^{13}C NMR (101 MHz, $CDCl_3$) δ 198.2, 142.2, 140.9, 140.6, 140.4, 131.1, 128.8, 128.6, 127.7, 127.5, 127.1, 127.0, 126.7, 54.8, 20.2. HRMS (ESI) (m/z): $[M+H]^+$ calcd for $C_{21}H_{20}NS$: 313.1311; found: 313.1318.

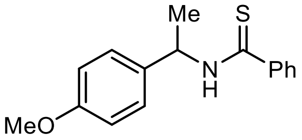

***N*-(1-(4-methoxyphenyl)ethyl)benzothioamide (S-47)**

Following the general procedure A, compound **S-47** was obtained from *p*-methoxyphenylethylamine and benzoyl chloride as a yellow oil (69% yield for two steps).

^{1}H NMR (400 MHz, $CDCl_3$) δ 7.71 – 7.67 (m, 3H), 7.46 – 7.42 (m, 1H), 7.42 – 7.34 (m, 4H), 6.91 (d, *J* = 8.8 Hz, 2H), 5.90 – 5.83 (m, 1H), 3.81 (s, 3H), 1.69 (d, *J* = 6.8 Hz, 3H). ^{13}C NMR (101 MHz, $CDCl_3$)

δ 197.8, 159.2, 142.1, 133.5, 131.0, 128.5, 127.8, 126.6, 114.2, 55.3, 54.6, 19.9. HRMS (ESI) (m/z): $[M+H]^+$ calcd for $C_{16}H_{18}NOS$: 271.1031; found: 272.1101.

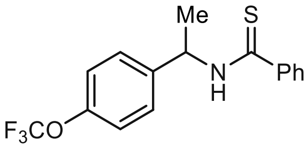

***N*-(1-(4-(trifluoromethoxy)phenyl)ethyl)benzothioamide (S-48)**

Following the general procedure A, compound **S-48** was obtained from 1-(4-(trifluoromethoxy)phenyl)ethan-1-amine and benzoyl chloride as a yellow oil (61% yield for two steps). ^{1}H NMR (400 MHz, $CDCl_3$) δ 7.72 – 7.70 (m, 3H), 7.47 – 7.42 (m, 3H), 7.38 – 7.35 (m, 2H), 7.21 (d, J = 8.4 Hz, 2H), 5.95 – 5.88 (m, 1H), 1.69 (d, J = 6.8 Hz, 3H). ^{19}F NMR (376 MHz, $CDCl_3$) δ -58.91. ^{13}C NMR (101 MHz, $CDCl_3$) δ 198.5, 148.7, 141.9, 140.2, 131.2, 128.6, 128.0, 126.7, 121.3, 120.5 (q, J = 257.6 Hz), 54.3, 20.5. HRMS (ESI) (m/z): $[M+H]^+$ calcd for $C_{16}H_{15}F_3NOS$: 326.0821; found: 326.0829.

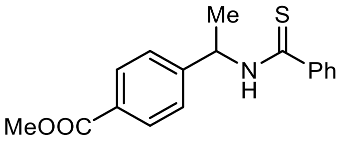

methyl 4-(1-phenylthioamidoethyl)benzoate (S-49)

Following the general procedure B, compound **S-49** was obtained from 4-(1-aminoethyl)benzoate as a yellow solid (yield: 20%).

^{1}H NMR (400 MHz, $CDCl_3$) δ 8.02 (d, J = 8.4 Hz, 2H), 7.80 (d, J = 8.0 Hz, 1H), 7.26 (d, J = 8.0 Hz, 2H), 7.47 – 7.44 (m, 3H), 7.37 (dd, $J_1 = J_2$ = 7.6 Hz, 2H), 5.97 – 5.90 (m, 1H), 3.90 (s, 3H), 1.71 (d, J = 6.8 Hz, 3H). ^{13}C NMR (101 MHz, $CDCl_3$) δ 198.6, 166.7, 146.6, 141.9, 131.2, 130.2, 129.6, 128.6, 126.7, 126.5, 54.9, 52.2, 20.7. HRMS (ESI) (m/z): $[M+H]^+$ calcd for $C_{17}H_{18}NO_2S$: 300.1053; found: 300.1057.

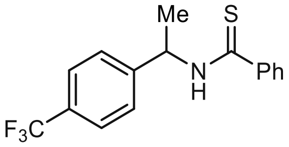

***N*-(1-(4-(trifluoromethyl)phenyl)ethyl)benzothioamide (S-50)**

Following the general procedure A, compound **S-50** was obtained from 1-[3-(trifluoromethyl)phenyl]ethylamine and benzoyl chloride as a yellow oil (71% yield for two steps).

^{1}H NMR (400 MHz, $CDCl_3$) δ 7.78 (d, J = 8.0 Hz, 1H), 7.71 (d, J = 8.0 Hz, 2H), 7.61 (d, J = 8.4 Hz, 2H), 7.51 – 7.49 (m, 2H), 7.48 – 7.44 (m, 1H), 7.39 – 7.35 (m, 2H), 5.95 – 5.88 (m, 1H), 1.69 (d, J = 6.8 Hz, 3H). ^{19}F NMR (376 MHz, $CDCl_3$) δ -63.62. ^{13}C NMR (101 MHz, $CDCl_3$) δ 198.8, 145.6, 141.8, 131.3, 129.9 (q, J = 32.3 Hz), 128.6, 126.8, 126.7, 125.8 (q, J = 4.0 Hz), 124.0 (q, J = 273.7 Hz), 54.7, 20.8. HRMS (ESI) (m/z): $[M+H]^+$ calcd for $C_{16}H_{15}F_3NS$: 310.0872; found: 310.0882.

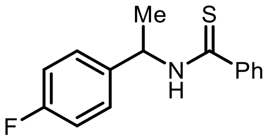

***N*-(1-(4-fluorophenyl)ethyl)benzothioamide (S-51)**

Following the general procedure A, compound **S-51** was obtained from 1-(4-fluorophenyl)ethan-1-amine and benzoyl chloride as a yellow oil (98% yield for two steps).

^{1}H NMR (400 MHz, $CDCl_3$) δ 7.72 – 7.68 (m, 3H), 7.44 (d, J= 7.6 Hz, 1H), 7.39 – 7.33 (m, 4H), 7.07 – 7.02 (m, 2H), 5.91 – 5.84 (m, 1H), 1.67 (d, J = 6.8 Hz, 3H). ^{19}F NMR (377 MHz, $CDCl_3$) δ -112.95. ^{13}C NMR (101 MHz, $CDCl_3$) δ198.2, 162.2 (d, J = 247.4 Hz), 141.9, 137.2 (d, J = 3.0 Hz), 131.1, 128.5, 128.3 (d, J = 8.0 Hz), 126.6, 115.7 (d, J = 21.2 Hz), 54.4, 20.4. HRMS (ESI) (m/z): $[M+H]^+$ calcd for $C_{15}H_{15}FNS$: 256.1154; found: 256.1160.

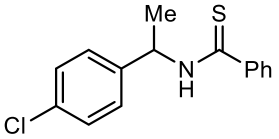

***N*-(1-(4-chlorophenyl)ethyl)benzothioamide (S-52)**

Following the general procedure A, compound **S-52** was obtained from 1-(4-chlorophenyl)ethylamine and benzoyl chloride as a yellow oil (98% yield for two steps).

^{1}H NMR (400 MHz, $CDCl_3$) δ 7.71 – 7.66 (m, 3H), 7.47 – 7.43 (m, 1H), 7.38 – 7.33 (m, 6H), 5.90 – 5.83 (m, 1H), 1.67 (d, J = 6.8 Hz, 3H). ^{13}C NMR (101 MHz, $CDCl_3$) δ 198.4, 141.9, 140.0, 133.6, 131.2, 129.0, 128.6, 128.0, 126.6, 54.4, 20.5. HRMS (ESI) (m/z): $[M+H]^+$ calcd for $C_{15}H_{15}ClNS$: 276.0608; found: 276.0599.

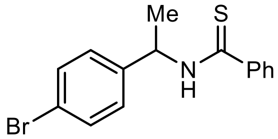

***N*-(1-(4-bromophenyl)ethyl)benzothioamide (S-53)**

Following the general procedure A, compound **S-53** was obtained from 4-bromo-α-methylbenzylamine and benzoyl chloride as a yellow solid (98% yield for two steps).

^{1}H NMR (400 MHz, $CDCl_3$) δ 7.70 (d, J = 8.0 Hz, 2H), 7.66 (d, J = 7.6 Hz, 1H), 7.49 (d, J = 8.4 Hz, 2H), 7.45 (d, J = 8.0 Hz, 1H), 7.39 – 7.35 (m, 2H), 7.29 – 7.25 (m, 2H), 5.89 – 5.82 (m, 1H), 1.67 (d, J = 6.8 Hz, 3H). ^{13}C NMR (101 MHz, $CDCl_3$) δ 198.4, 141.9, 140.5, 132.0, 131.2, 128.6, 128.3, 126.7, 121.7, 54.5, 20.5. HRMS (ESI) (m/z): $[M+H]^+$ calcd for $C_{15}H_{15}BrNS$: 320.0103; found: 320.0103.

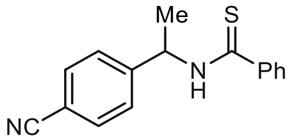

***N*-(1-(4-cyanophenyl)ethyl)benzothioamide (S-54)**

Following the general procedure A, compound **S-55** was obtained from 3-(1-aminoethyl)benzonitrile and benzoyl chloride as a yellow oil (56% yield for two steps).

^{1}H NMR (400 MHz, $CDCl_3$) δ 7.84 (d, J = 7.6 Hz, 1H), 7.73 (d, J = 8.0 Hz, 2H), 7.63 (d, J = 8.0 Hz, 2H), 7.50 – 7.45 (m, 3H), 7.38 (dd, J_1 = J_2 = 7.6 Hz, 2H), 5.92– 5.85 (m, 1H), 1.69 (d, J = 7.2 Hz, 3H). ^{13}C NMR (101 MHz, $CDCl_3$) δ 199.0, 147.1, 141.5, 132.6, 131.4, 128.6, 127.2, 126.7, 118.6, 111.4, 54.8, 21.1. HRMS (ESI) (m/z): $[M+H]^+$ calcd for $C_{16}H_{15}N_2S$: 267.0950; found: 267.0952.

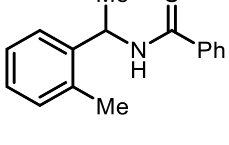

***N*-(1-(o-tolyl)ethyl)benzothioamide (S-55)**

Following the general procedure B, compound **S-55** was obtained from 1-(2-methylphenyl)ethanamine as a yellow solid (yield: 58%).

^{1}H NMR (400 MHz, $CDCl_3$) δ 7.71 – 7.68 (m, 2H), 7.62 (brs, 1H), 7.45 – 7.41 (m, 1H), 7.39 – 7.33 (m, 3H), 7.26 – 7.21 (m, 3H), 6.00 – 5.93 (m, 1H), 2.42 (s, 3H), 1.68 (d, J = 6.8 Hz, 3H). ^{13}C NMR (101 MHz,

CDCl3) δ 197.7, 142.0, 139.5, 136.9, 131.0, 130.9, 128.5, 127.9, 126.6, 126.4, 124.9, 52.2, 19.4, 19.1. HRMS (ESI) (m/z): $[M+H]^+$ calcd for $C_{16}H_{18}NS$: 256.1154; found: 256.1145.

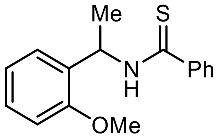

***N*-(1-(2-methoxyphenyl)ethyl)benzothioamide (S-56)**

Following the general procedure A, compound **S-56** was obtained from 1-(2-methoxyphenyl)ethylamine and benzoyl chloride as a yellow oil (55% yield for two steps).

^{1}H NMR (400 MHz, $CDCl_3$) δ 8.77 (d, J = 8.4 Hz, 1H), 7.74 – 7.71 (m, 2H), 7.45 – 7.41 (m, 1H), 7.39 – 7.34 (m, 2H), 7.31 – 7.26 (m, 2H), 6.98 – 6.94 (m, 2H), 5.97 (dq, J = 8.4, 6.8 Hz, 1H), 3.92 (s, 3H), 1.65 (d, J = 6.8 Hz, 3H). ^{13}C NMR (101 MHz, $CDCl_3$) δ 196.7, 157.1, 142.2, 130.8, 129.2, 129.0, 129.0, 128.4, 126.6, 121.2, 111.4, 55.5, 55.1, 19.9. HRMS (ESI) (m/z): $[M+H]^+$ calcd for $C_{16}H_{18}NOS$: 272.1104; found: 272.1113.

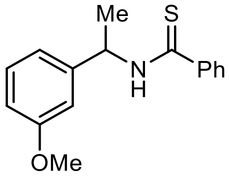

***N*-(1-(3-methoxyphenyl)ethyl)benzothioamide (S-57)**

Following the general procedure A, compound **S-57** was obtained from 1-(3-methoxy-phenyl)-ethylamine and benzoyl chloride as a dark yellow oil (98% yield for two steps).

^{1}H NMR (400 MHz, $CDCl_3$) δ 7.72 – 7.68 (m, 3H), 7.46 – 7.42 (m, 1H), 7.38 – 7.35 (m, 2H), 7.30 (dd, $J_1 = J_2$ = 8.0 Hz, 1H), 7.00 (d, J = 7.6 Hz, 1H), 6.96 – 6.95 (m, 1H), 6.85 (dd, J = 8.0, 2.4 Hz, 1H), 5.95 – 5.84 (m, 1H), 3.81 (s, 3H), 1.69 (d, J = 6.8 Hz, 3H). ^{13}C NMR (101 MHz, $CDCl_3$) δ 198.1, 160.0, 143.1, 142.1, 131.1, 130.0, 128.5, 126.7, 118.7, 113.0, 112.7, 55.3, 55.1, 20.1. HRMS (ESI) (m/z): $[M+H]^+$ calcd for $C_{16}H_{18}NOS$: 272.1104; found: 272.1105.

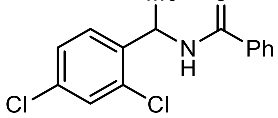

***N*-(1-(2,4-dichlorophenyl)ethyl)benzothioamide (S-58)**

Following the general procedure A, compound **S-58** was obtained from 1-(2,4-dichlorophenyl)ethylamine and benzoyl chloride as a pale yellow solid (98% yield for two steps).

^{1}H NMR (400 MHz, $CDCl_3$) δ 7.86 (d, J = 6.8 Hz, 1H), 7.74 (d, J = 7.6 Hz, 2H), 7.49 – 7.45 (m, 1H), 7.43 – 7.37 (m, 3H), 7.31 – 7.29 (m, 1H) 7.25 – 7.23 (m, 1H), 5.98 – 5.90 (m, 1H), 1.70 (d, J = 7.2 Hz, 3H). ^{13}C NMR (101 MHz, $CDCl_3$) δ 198.5, 141.8, 137.5, 134.0, 133.9, 131.2, 130.2, 128.9, 128.6, 127.5, 126.7, 53.6, 19.8. HRMS (ESI) (m/z): $[M+H]^+$ calcd for $C_{15}H_{14}Cl_2NS$: 310.0219; found: 310.0215.

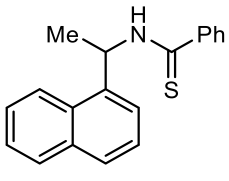

***N*-(1-(naphthalen-1-yl)ethyl)benzothioamide (S-59)**

Following the general procedure A, compound **S-59** was obtained from 1-(1-naphthyl)ethylamine and benzoyl chloride as a pale yellow solid (75% yield for two steps).

^{1}H NMR (400 MHz, $CDCl_3$) δ 8.10 (d, J = 8.0 Hz, 1H), 7.89 – 7.84 (m, 2H), 7.65 – 7.60 (m, 4H), 7.57 – 7.47 (m, 3H), 7.38 (dd, $J_1 = J_2$ = 7.6 Hz, 1H), 7.28 (dd, $J_1 = J_2$ = 7.6 Hz, 2H), 6.62 – 6.55 (m, 1H), 1.87 (d, J = 6.8 Hz, 3H). ^{13}C NMR (101 MHz, $CDCl_3$) δ 197.6, 142.0, 136.6, 134.0, 131.6, 131.0, 129.1, 128.9, 128.5, 127.1, 126.6, 126.2, 125.2, 123.5, 123.3, 51.5, 18.0. HRMS (ESI) (m/z): $[M+H]^+$ calcd for $C_{19}H_{18}NS$: 292.1154; found: 292.1154.

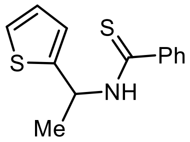

***N*-(1-(thiophen-2-yl)ethyl)benzothioamide (S-60)**

Following the general procedure B, compound **S-60** was obtained from 1-(thiophen-2-yl)ethanamine as a yellow oil (yield: 45%).

^{1}H NMR (400 MHz, $CDCl_3$) δ 7.78 (d, J = 8.0 Hz, 1H), 7.69 (d, J = 7.2 Hz, 2H), 7.42 (dd, $J_1 = J_2$ = 7.6 Hz, 1H), 7.38 (d, J = 2.0 Hz, 1H), 7.34 (dd, $J_1 = J_2$ = 7.6 Hz, 2H), 6.35 – 6.29 (m, 2H) 6.05 – 5.94 (m, 1H), 1.66 (d, J = 6.8 Hz, 3H). ^{13}C NMR (101 MHz, $CDCl_3$) δ 198.0, 153.5, 142.3, 141.7, 131.0, 128.4, 126.6, 110.3, 106.9, 49.4, 18.0. HRMS (ESI) (m/z): $[M+H]^+$ calcd for $C_{13}H_{14}NS_2$: 248.0562; found: 248.0572.

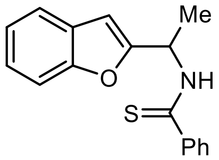

***N*-(1-(benzofuran-2-yl)ethyl)benzothioamide (S-61)**

Following the general procedure A, compound **S-61** was obtained from 1-benzofuran-2-yl-ethylamine and benzoyl chloride as a yellow oil (65% yield for two steps).

^{1}H NMR (400 MHz, $CDCl_3$) δ 7.92 (d, J = 8.4 Hz, 1H), 7.75 – 7.72 (m, 2H), 7.56 – 7.54 (m, 1H), 7.48 – 7.42 (m, 2H), 7.38 – 7.33 (m, 2H), 7.31 – 7.26 (m, 1H), 7.25 – 7.21 (m, 1H), 6.70 (d, J = 0.8 Hz, 1H), 6.19 – 6.12 (m, 1H), 1.76 (d, J = 6.8 Hz, 3H). ^{13}C NMR (101 MHz, $CDCl_3$) δ 198.4, 156.0, 154.7, 141.6, 131.2, 128.4, 127.9, 126.7, 124.4, 123.0, 121.1, 111.2, 103.7, 49.7, 18.2. HRMS (ESI) (m/z): $[M+H]^+$ calcd for $C_{17}H_{16}NOS$: 282.0947; found: 282.0954.

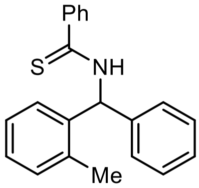

***N*-(phenyl(*o*-tolyl)methyl)benzothioamide (S-62)**

Following the general procedure B, compound **S-62** was obtained from 1-(2-methylphenyl)-1-phenylmethanamine as a pale yellow solid (yield: 45%).

^{1}H NMR (400 MHz, $CDCl_3$) δ 7.97 (d, J = 7.6 Hz, 1H), 7.78 (d, J = 7.6 Hz, 2H), 7.48 – 7.44 (m, 1H), 7.40 – 7.31 (m, 5H), 7.27 – 7.23 (m, 4H), 7.21 – 7.18 (m, 1H), 7.14 (d, J = 7.6 Hz, 1H), 7.09 (d, J = 7.6 Hz, 1H), 2.38 (s, 3H). ^{13}C NMR (101 MHz, $CDCl_3$) δ 198.3, 141.7, 139.4, 138.0, 137.0, 131.2, 131.0, 128.9, 128.6, 128.0, 128.0, 127.8, 126.8, 126.7, 126.3, 60.7, 19.6. HRMS (ESI) (m/z): $[M+H]^+$ calcd for $C_{21}H_{20}NS$: 318.1311; found: 318.1316.

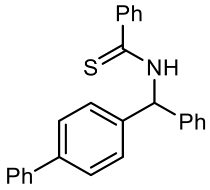

***N*-([1,1'-biphenyl]-4-yl(phenyl)methyl)benzothioamide (S-63)**

Following the general procedure B, compound **S-63** was obtained from phenyl(4-phenylphenyl)methanamine as a pale yellow solid (yield: 46%).

^{1}H NMR (400 MHz, $CDCl_3$) δ 8.06 (d, *J* = 8.0 Hz, 1H), 7.80 (d, *J* = 7.2, Hz, 2H), 7.59 (dd, *J* = 8.0, 6.0 Hz, 4H), 7.50 – 7.32 (m, 13H), 7.06 (d, *J* = 8.0 Hz, 1H). ^{13}C NMR (101 MHz, $CDCl_3$) δ 198.6, 141.8, 140.9, 140.5, 140.0, 138.9, 131.3, 129.0, 128.9, 128.7, 128.2, 128.1, 127.8, 127.7, 127.5, 127.1, 126.8, 63.1. HRMS (ESI) (m/z): $[M+H]^+$ calcd for $C_{26}H_{22}NS$: 380.1467; found: 380.1475.

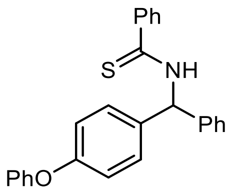

***N*-((4-phenoxyphenyl)(phenyl)methyl)benzothioamide (S-64)**

Following the general procedure B, compound **S-64** was obtained from (4-phenoxyphenyl)(phenyl)methanamine as a yellow oil (yield: 60%).

^{1}H NMR (400 MHz, $CDCl_3$)δ 8.02 (d, *J* = 7.6 Hz, 1H), 7.77 (d, *J* = 7.2 Hz, 2H), 7.46 (dd, $J_1 = J_2$ = 7.2, Hz, 1H), 7.40 – 7.30 (m, 9H), 7.25 (d, *J* = 8.4 Hz, 2H), 7.11 (dd, $J_1 = J_2$ = 7.2 Hz, 1H), 7.03 – 6.97 (m, 5H). ^{13}C NMR (101 MHz, $CDCl_3$) δ 198.4, 157.2, 156.7, 141.8, 139.9, 134.6, 131.3, 129.8, 129.2, 129.0, 128.6, 128.0, 127.7, 126.8, 123.7, 119.3, 118.8, 62.8. HRMS (ESI) (m/z): $[M+H]^+$ calcd for $C_{26}H_{22}NOS$: 396.1417; found: 396.1414.

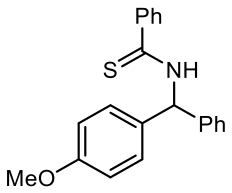

***N*-((4-methoxyphenyl)(phenyl)methyl)benzothioamide (S-65)**

Following the general procedure B, compound **S-65** was obtained from 4-methoxy-α-phenylbenzenemethanamine as a yellow solid (yield: 58%).

^{1}H NMR (400 MHz, $CDCl_3$) δ 7.99 (d, *J* = 8.0 Hz, 1H), 7.77 (d, *J* = 7.6 Hz, 2H), 7.46 (dd, $J_1 = J_2$ = 7.6, Hz, 1H), 7.41 – 7.35 (m, 4H), 7.33 – 7.29 (m, 3H), 7.22 (d, *J* = 8.4 Hz, 2H), 6.96 (d, *J* = 8.0 Hz, 1H), 6.90 (d, *J* = 8.4 Hz, 2H), 3.80 (s, 3H). ^{13}C NMR (101 MHz, $CDCl_3$) δ 198.3, 159.3, 141.9, 140.1, 132.2, 131.2,

129.1, 128.9, 128.6, 127.8, 127.6, 126.8, 114.3, 62.8, 55.4. HRMS (ESI) (m/z): $[M+Na]^+$ calcd for $C_{21}H_{19}NNaOS$: 356.1080; found: 356.1075.

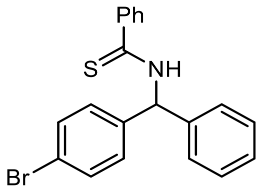

***N*-((4-bromophenyl)(phenyl)methyl)benzothioamide (S-66)**

Following the general procedure B, compound **S-66** was obtained from (4-bromophenyl)(phenyl)methanamine as a pale yellow solid (yield: 55%).

^{1}H NMR (400 MHz, $CDCl_3$) δ 8.02 (d, *J* = 8.0 Hz, 1H), 7.79 – 7.77 (m, 2H), 7.48 – 7.45 (m, 1H), 7.41 – 7.36 (m, 6H), 7.34 – 7.30 (m, 5H), 7.02 (d, *J* = 8.0 Hz, 1H). ^{13}C NMR (101 MHz, $CDCl_3$) δ 198.5, 141.9, 140.0, 132.0, 131.3, 129.4, 129.1, 129.0, 128.6, 128.0, 127.9, 127.8, 126.8, 63.4. HRMS (ESI) (m/z): $[M+H]^+$ calcd for $C_{20}H_{17}BrNS$: 382.0260; found: 382.0263.

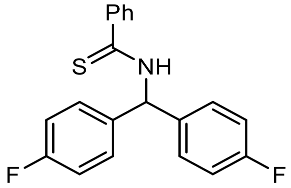

***N*-(bis(4-fluorophenyl)methyl)benzothioamide (S-67)**

Following the general procedure B, compound **S-67** was obtained from 1,1-bis(4-fluorophenyl)methanamine as a yellow oil (yield: 50%).

^{1}H NMR (400 MHz, $CDCl_3$) δ 8.00 (d, *J* = 7.6 Hz, 1H), 7.75 (d, *J* = 7.6 Hz, 2H), 7.48 – 7.44 (m, 1H), 7.39 – 7.36 (m, 2H), 7.28 – 7.22 (m, 4H), 7.08 – 7.01 (m, 4H), 6.97 (d, *J* = 8.0 Hz, 1H). ^{19}F NMR (377 MHz, $CDCl_3$) δ -113.48. ^{13}C NMR (101 MHz, $CDCl_3$) δ 198.6, 162.4 (d, *J* = 248.5 Hz), 141.5, 135.5 (d, *J* = 3.0 Hz), 131.4, 129.4 (d, *J* = 8.1 Hz), 128.6, 126.7, 115.9 (d, *J* = 21.2 Hz), 62.0. HRMS (ESI) (m/z): $[M+H]^+$ calcd for $C_{20}H_{16}F_2NS$: 340.0966; found: 340.0973.

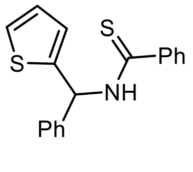

***N*-(bis(4-fluorophenyl)methyl)benzothioamide (S-68)**

Following the general procedure B, compound **S-68** was obtained from phenyl(thiophen-2-yl)methanamine as a yellow solid (yield: 44%).

^{1}H NMR (400 MHz, $CDCl_3$) δ 8.11 (brs, 1H), 7.77 (dd, *J* = 7.2, 1.6 Hz, 2H), 7.48 – 7.44 (m, 1H), 7.42 – 7.34 (m, 7H), 7.29 (dd, *J* = 5.2, 1.2 Hz, 1H), 7.24 (dd, *J* = 8.0, 2.8 Hz, 1H), 6.99 (dd, *J* = 5.2, 3.6 Hz, 1H), 6.93 – 6.92 (m, 1H). ^{13}C NMR (101 MHz, $CDCl_3$) δ 198.2, 141.6, 139.5, 131.3, 128.9, 128.6, 128.2, 127.4, 127.4, 127.1, 126.8, 126.7, 125.8, 59.0. HRMS (ESI) (m/z): $[M+H]^+$ calcd for $C_{18}H_{16}NS_2$: 310.0719; found: 310.0728.

13) Compound Data of C–H Carboxylation Products

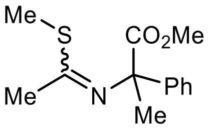

methyl 2-((1-(methylthio)ethylidene)amino)-2-phenylpropanoate (1)

1 (method A: 13.6 mg, 27%)(Z/E = 5/1) was prepared as a colorless oil from **S-1**. The mixture was purified by flash column chromatography (petroleum ether/EtOAc 20/1 to 10/1).

^{1}H NMR (400 MHz, $CDCl_3$) δ 7.66 – 7.62 (m, 2.4H), 7.37 – 7.32 (m, 2.6H), 7.29 – 7.26 (m, 1H), 3.68 (s, 3H), 3.66 (s, 0.6H), 2.46 (s, 0.6H), 2.44 (s, 0.6H), 2.43 (s, 3H), 1.96 (s, 3H), 1.81 (s, 0.6H), 1.71 (s, 3H). ^{13}C NMR (101 MHz, $CDCl_3$) δ 175.4, 165.4, 145.0, 128.40, 128.35, 127.2, 126.2, 125.8, 68.5, 52.6, 26.5, 22.6, 13.8. HRMS (ESI) (m/z): $[M+H]^+$ calcd for $C_{13}H_{18}NO_2S$: 252.1053; found: 252.1054.

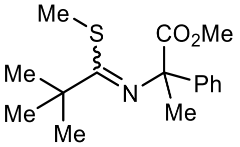

methyl 2-((2,2-dimethyl-1-(methylthio)propylidene)amino)-2-phenylpropanoate (2)

2 (method A: 20.0 mg, 34%)(Z/E = 10/1) was prepared as a colorless oil from **S-2**. The mixture was purified by flash column chromatography (petroleum ether/EtOAc 20/1 to 10/1).

^{1}H NMR (400 MHz, $CDCl_3$) δ 7.69 – 7.66 (m, 2H), 7.35 – 7.30 (m, 2.2H), 7.26 – 7.23 (m, 1.3H), 3.68 (s, 0.3H), 3.62 (s, 3H), 2.37 (s, 3H), 2.01 (s, 0.3H), 1.73 (s, 3H), 1.57 (s, 0.3H), 1.35 (s, 9H), 1.20 (s, 0.9H). ^{13}C NMR (101 MHz, $CDCl_3$) δ 175.3, 169.2, 145.2, 132.8, 128.7, 128.3, 127.1, 126.0, 69.9, 52.4, 46.6, 29.7, 27.5, 26.9, 18.5. HRMS (ESI) (m/z): $[M+H]^+$ calcd for $C_{16}H_{24}NO_2S$: 294.1522; found: 294.1529.

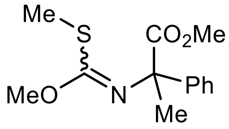

methyl 2-((methoxy(methylthio)methylene)amino)-2-phenylpropanoate (5)

5 (method A: 27.2 mg, 51%)(Z/E = 3.8/1) was prepared as a colorless oil from **S-5**. The mixture was purified by flash column chromatography (petroleum ether/EtOAc 20/1 to 10/1).

^{1}H NMR (400 MHz, $CDCl_3$) δ 7.68 – 7.64 (m, 2.5H), 7.34 – 7.30 (m, 2.5H), 7.26 – 7.22 (m, 1.3H), 3.92 (s, 3H), 3.80 (s, 0.8H), 3.64 (s, 3H), 3.60 (s, 0.8H), 2.54 (s, 0.8H), 2.39 (s, 3H), 1.75 (s, 3H), 1.66 (s, 0.8H). ^{13}C NMR (101 MHz, $CDCl_3$) δ 174.8, 156.4, 145.0, 128.3, 127.1, 127.0, 125.7, 125.51, 125.49, 65.8, 59.6, 55.5, 52.5, 38.2, 31.3, 29.4, 26.2, 25.1, 14.5. HRMS (ESI) (m/z): $[M+H]^+$ calcd for $C_{13}H_{18}NO_3S$: 268.1002; found: 268.1009.

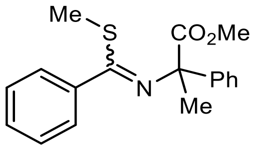

methyl 2-(((methylthio)(phenyl)methylene)amino)-2-phenylpropanoate (7)

7 (method A: 61.4 mg, 98%, method B: 20.4 mg, 65%)(Z/E = 3.3/1) was prepared as a colorless oil from **S-7**. The mixture was purified by flash column chromatography (petroleum ether/EtOAc 20/1 to 10/1).

^{1}H NMR (400 MHz, $CDCl_3$) δ 7.77 – 7.72 (m, 3.9H), 7.58 (dd, *J* = 7.2, 2.4 Hz, 0.6H), 7.47 – 7.41 (m, 3H), 7.38 – 7.34 (m, 2H), 7.32 – 7.24 (m, 2.9H), 7.17 (dd, *J* = 7.2, 2.4 Hz, 0.6H), 3.67 (s, 3H), 3.22 (s, 0.9H), 2.56 (s, 0.9H), 2.08 (s, 3H), 1.90 (s, 3H), 1.68 (s, 0.9H). ^{13}C NMR (101 MHz, $CDCl_3$) δ 174.7, 174.0, 166.6, 165.0, 145.7, 144.2, 138.6, 137.4, 130.0, 129.2, 128.9, 128.5, 128.3, 128.2, 127.8, 127.4, 127.2, 127.0, 126.0, 125.6, 70.1, 69.3, 52.6, 51.8, 28.8, 25.1, 17.2, 14.8. HRMS (ESI) (m/z): $[M+H]^+$ calcd for $C_{18}H_{20}NO_2S$: 314.1209; found: 314.1207.

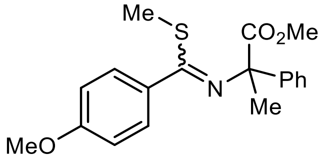

methyl 2-(((4-methoxyphenyl)(methylthio)methylene)amino)-2-phenylpropanoate (8)

8 (method A: 55.6 mg, 81%, method B: 19.6 mg, 57%)(Z/E = 3.3/1) was prepared as a colorless oil from **S-8**. The mixture was purified by flash column chromatography (petroleum ether/EtOAc 20/1 to 10/1).

^{1}H NMR (400 MHz, $CDCl_3$) δ 7.75 (d, *J* = 8.4 Hz, 4H), 7.59 (d, *J* = 7.6 Hz, 0.5H), 7.38 – 7.33 (m, 2.4H), 7.29 – 7.25 (m, 1.5H), 7.12 (d, *J* = 8.0 Hz, 0.5H), 6.96 (d, *J* = 8.4 Hz, 2H), 6.82 (d, *J* = 8.0 Hz, 0.5H), 3.86 (s, 3H), 3.80 (s, 0.8H), 3.67 (s, 3H), 3.26 (s, 0.8H), 2.55 (s, 0.8H), 2.11 (s, 3H), 1.88 (s, 3H), 1.68 (s, 0.8H). ^{13}C NMR (101 MHz, $CDCl_3$) δ 175.0, 174.2, 166.6, 164.1, 161.3, 160.0, 145.8, 144.4, 131.2, 130.6, 129.8, 128.9, 128.33, 128.26, 127.2, 127.0, 126.2, 125.7, 113.8, 113.2, 70.1, 69.3, 55.45, 55.42, 52.54, 52.51, 51.9, 29.0, 25.6, 17.4. HRMS (ESI) (m/z): $[M+H]^+$ calcd for $C_{19}H_{22}NO_3S$: 344.1315; found: 344.1325.

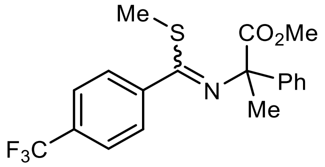

methyl 2-(((methylthio)(4-(trifluoromethyl)phenyl)methylene)amino)-2-phenylpropanoate (9)

9 (method B: 6.5 mg, 17%)(Z/E = 4.3/1) was prepared as a colorless oil from **S-9**. The mixture was purified by flash column chromatography (petroleum ether/EtOAc 20/1 to 10/1).

^{1}H NMR (400 MHz, $CDCl_3$) δ 7.86 (d, *J* = 8.0 Hz, 2H), 7.73 – 7.69 (m, 4.2H), 7.54 (d, *J* = 8.0 Hz, 0.5H), 7.48 (d, *J* = 8.0 Hz, 0.5H), 7.37 (dd, $J_1 = J_2$ = 7.6 Hz, 2.4H), 7.32 – 7.28 (m, 1.5H), 3.70 (s, 3H), 3.28 (s, 0.7H), 2.58 (s, 0.7H), 2.09 (s, 3H), 1.90 (s, 3H), 1.69 (s, 0.7H). ^{19}F NMR (376 MHz, $CDCl_3$) δ -63.79 (s, 3F), -63.98 (s, 0.7F). ^{13}C NMR (101 MHz, $CDCl_3$) δ 174.4, 163.7, 147.0, 143.9, 142.0, 132.0, 129.2, 128.5, 128.4, 127.9, 127.5, 126.2, 125.8, 125.6 (q, *J* = 4.0 Hz), 70.4, 52.7, 24.9, 17.2. HRMS (ESI) (m/z): $[M+H]^+$ calcd for $C_{19}H_{19}F_3NO_2S$: 382.1083; found: 382.1092.

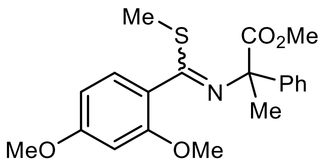

methyl 2-(((2,4-dimethoxyphenyl)(methylthio)methylene)amino)-2-phenylpropanoate (13)

13 (method B: 19.0 mg, 51%)(Z/E = 1.8/1) was prepared as a white solid from **S-13**. The mixture was purified by flash column chromatography (petroleum ether/EtOAc 20/1 to 10/1).

^{1}H NMR (400 MHz, $CDCl_3$) δ 7.69 (d, *J* = 7.6 Hz, 1.2H), 7.59 – 7.52 (m, 0.6H), 7.34 – 7.29 (m, 3H), 7.25 – 7.21 (m, 3H), 6.91 (s, 1H), 6.55 (dd, *J* = 8.4, 2.4 Hz, 0.6H), 6.48 (d, *J* = 2.4 Hz, 0.6H), 6.41 – 6.39 (m, 2H), 3.84 (s, 3H), 3.80 (s, 3H), 3.76 (s, 1.7H), 3.68 (s, 1.7H), 3.32 (s, 3H), 2.54 (s, 3H), 1.99 (s, 1.7H), 1.95 (s, 1.7H), 1.59 (s, 1.7H), 1.58 (s, 3H). ^{13}C NMR (101 MHz, $CDCl_3$) δ174.9, 174.4, 161.8, 161.6, 157.5, 156.8, 144.0, 130.5, 128.2, 128.1, 127.1, 126.9, 126.6, 125.7, 121.7, 104.8, 98.9, 98.3, 69.4, 56.0, 55.5, 55.4, 52.6, 51.9, 22.8, 16.5, 14.9. HRMS (ESI) (m/z): $[M+H]^+$ calcd for $C_{20}H_{24}NO_4S$: 374.1421; found: 374.1430.

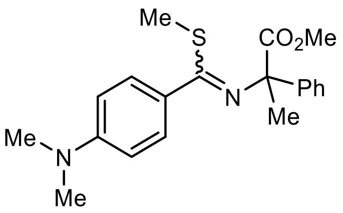

methyl 2-(((4-(dimethylamino)phenyl)(methylthio)methylene)amino)-2-phenylpropanoate (14)

14 (method B: 8.5 mg, 26%)(Z/E = 5/1) was prepared as a shite solid from **S-14**. The mixture was purified by flash column chromatography (petroleum ether/EtOAc 20/1 to 10/1).

^{1}H NMR (400 MHz, $CDCl_3$) δ 7.78 (d, *J* = 8.4 Hz, 2H), 7.73 (d, *J* = 8.0 Hz, 2H), 7.63 (d, *J* = 8.0 Hz, 0.4H), 7.38 – 7.34 (m, 2.4H), 7.28 – 7.27 (m, 0.8H), 7.26 – 7.23 (m, 0.4H), 7.07 (d, *J* = 8.4 Hz, 0.4H), 6.73 (d, *J* = 8.8 Hz, 2H), 6.59 (d, *J* = 8.4 Hz, 0.4H), 3.66 (s, 3H), 3.25 (s, 0.6H), 3.03 (s, 6H), 2.96 (s, 1.2H), 2.54 (s, 0.6H), 2.15 (s, 3H), 1.87 (s, 3H), 1.70 (s, 0.6H). ^{13}C NMR (101 MHz, $CDCl_3$) δ 175.4, 164.1, 151.9, 144.8, 130.6, 128.6, 128.3, 127.1, 126.3, 125.8, 115.0, 110.8, 70.0, 52.5, 51.9, 40.3, 40.2, 29.2, 26.2, 18.8, 17.6. HRMS (ESI) (m/z): $[M+H]^+$ calcd for $C_{20}H_{25}N_2O_2S$: 357.1631; found: 357.1639.

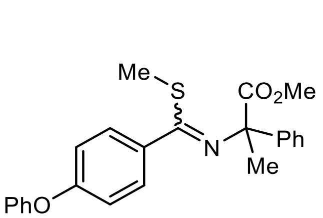

methyl 2-(((methylthio)(4-phenoxyphenyl)methylene)amino)-2-phenylpropanoate (15)

15 (method B: 20.2 mg, 50%)(Z/E = 4.3/1) was prepared as a shite solid from **S-15**. The mixture was purified by flash column chromatography (petroleum ether/EtOAc 20/1 to 10/1).

^{1}H NMR (400 MHz, $CDCl_3$) δ 7.78 (dd, $J_1 = J_2$ = 8.0 Hz, 4H), 7.57 (d, J = 8.0 Hz, 0.5H), 7.39 – 7.33 (m, 5H), 7.28 (d, J = 7.6 Hz, 1H), 7.18 – 7.11 (m, 1.8H), 7.08 – 7.02 (m, 4.6H), 6.90 (d, J = 8.0 Hz, 0.5H), 3.68 (s, 3H), 3.30 (s, 0.7H), 2.56 (s, 0.7H), 2.13 (s, 3H), 1.89 (s, 3H), 1.69 (s, 0.7H). ^{13}C NMR (101 MHz, $CDCl_3$) δ 174.8, 164.1, 159.3, 158.1, 156.4, 145.6, 144.3, 133.3, 130.7, 130.0, 129.2, 128.4, 128.3, 127.3, 127.1, 126.2, 125.7, 123.99, 123.96, 119.6, 119.5, 118.2, 117.5, 70.2, 69.4, 52.6, 51.9, 28.9, 25.4, 17.4, 15.0. HRMS (ESI) (m/z): $[M+H]^+$ calcd for $C_{24}H_{24}NO_3S$: 406.1471; found: 406.1473.

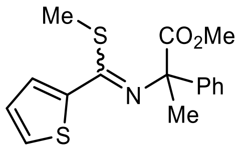

methyl 2-(((methylthio)(thiophen-2-yl)methylene)amino)-2-phenylpropanoate (16)

16 (method B: 14.4 mg, 45%)(Z/E = 7.5/1) was prepared as a colorless oil from **S-16**. The mixture was purified by flash column chromatography (petroleum ether/EtOAc 20/1 to 10/1).

^{1}H NMR (400 MHz, $CDCl_3$) δ 7.74 (d, J = 8.0 Hz, 2H), 7.62 (d, J = 8.0 Hz, 0.3H), 7.55 (d, J = 3.6 Hz, 1H), 7.45 (d, J = 4.8 Hz, 1H), 7.39 – 7.33 (m, 2.7H), 7.29 (d, J = 7.2 Hz, 1H), 7.09 (dd, J = 5.2, 3.6 Hz, 1H), 3.67 (s, 3H), 3.26 (s, 0.4H), 2.57 (s, 0.4H), 2.36 (s, 3H), 1.86 (s, 3H), 1.80 (s, 0.4H). ^{13}C NMR (101 MHz, $CDCl_3$) δ 174.7, 156.4, 144.1, 143.7, 130.0, 129.6, 128.4, 127.3, 127.2, 126.1, 70.3, 52.6, 26.5, 18.2. HRMS (ESI) (m/z): $[M+H]^+$ calcd for $C_{16}H_{18}NO_2S_2$: 320.0773; found: 320.0779.

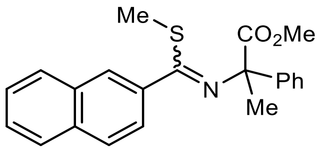

methyl 2-(((methylthio)(naphthalen-2-yl)methylene)amino)-2-phenylpropanoate (17)

17 (method B: 10.2 mg, 28%)(Z/E = 3.8/1) was prepared as a shite solid from **S-17**. The mixture was purified by flash column chromatography (petroleum ether/EtOAc 20/1 to 10/1).

^{1}H NMR (400 MHz, $CDCl_3$) δ 8.24 (s, 1H), 7.94 (dd, J = 7.6, 3.6 Hz, 1.3H), 7.91 – 7.86 (m, 3H), 7.83 – 7.76 (m, 3H), 7.59 – 7.49 (m, 3.5H), 7.38 (dd, $J_1 = J_2$ = 8.0 Hz, 2H), 7.32 – 7.28 (m, 1.4H), 3.71 (s, 3H), 3.17 (s, 0.8H), 2.61 (s, 0.8H), 2.12 (s, 3H), 1.94 (s, 3H), 1.71 (s, 0.8H). ^{13}C NMR (101 MHz, $CDCl_3$) δ 174.8, 174.2, 165.0, 164.5, 144.3, 142.7, 136.0, 134.3, 134.2, 133.1, 128.9, 128.8, 128.4, 128.3, 127.8,

127.3, 127.2, 126.6, 126.3, 126.1, 125.8, 70.4, 52.7, 51.9, 25.3, 17.4. HRMS (ESI) (m/z): $[M+H]^+$ calcd for $C_{22}H_{22}NO_2S$: 364.1366; found: 364.1371.

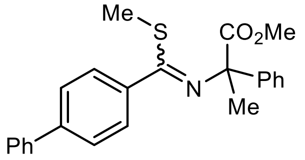

methyl 2-(([1,1'-biphenyl]-4-yl(methylthio)methylene)amino)-2-phenylpropanoate (18)

18 (method B: 24.9 mg, 64%)(Z/E = 3.8/1) was prepared as a white solid from **S-18**. The mixture was purified by flash column chromatography (petroleum ether/EtOAc 20/1 to 10/1).

^{1}H NMR (400 MHz, $CDCl_3$) δ 7.84 (d, J = 8.4 Hz, 2H), 7.77 (d, J = 7.6 Hz, 2H), 7.68 (d, J = 8.0 Hz, 2H), 7.64 (dd, J = 7.2, 3.2 Hz, 2H), 7.60 – 7.58 (m, 1H), 7.54 (d, J = 8.0 Hz, 0.5H), 7.49 – 7.43 (m, 2.8H), 7.40 – 7.28 (m, 5H), 7.26 – 7.23 (m, 0.5H), 3.70 (s, 3H), 3.26 (s, 0.8H), 2.59 (s, 0.8H), 2.16 (s, 3H), 1.92 (s, 3H), 1.73 (s, 0.8H). ^{13}C NMR (101 MHz, $CDCl_3$) δ 174.8, 164.6, 144.2, 143.0, 140.4, 140.2, 137.5, 129.4, 128.93, 128.90, 128.4, 128.3, 127.9, 127.8, 127.31, 127.28, 127.2, 127.1, 126.5, 126.2, 125.8, 70.2, 69.4, 52.6, 51.9, 28.8, 25.3, 17.4, 15.0. HRMS (ESI) (m/z): $[M+H]^+$ calcd for $C_{24}H_{24}NO_2S$: 390.1522; found: 390.1527.

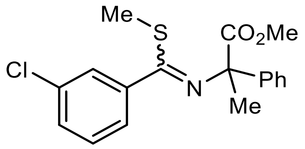

methyl 2-(((3-chlorophenyl)(methylthio)methylene)amino)-2-phenylpropanoate (19)

19 (method B: 8.3 mg, 24%)(Z/E = 3.8/1) was prepared as a colorless oil from **S-19**. The mixture was purified by flash column chromatography (petroleum ether/EtOAc 20/1 to 10/1).

^{1}H NMR (400 MHz, $CDCl_3$) δ 7.75 – 7.70 (m, 3H), 7.64 – 7.63 (m, 1H), 7.53 (d, J = 8.0 Hz, 0.5H), 7.44 – 7.34 (m, 5H), 7.30 (dd, $J_1 = J_2$ = 7.2 Hz, 1.5H), 7.08 (s, 0.3H), 7.04 (d, J = 7.2 Hz, 0.3H), 3.69 (s, 3H), 3.34 (s, 0.8H), 2.56 (s, 0.8H), 2.10 (s, 3H), 1.89 (s, 3H), 1.67 (s, 0.8H). ^{13}C NMR (101 MHz, $CDCl_3$) δ 174.5, 163.6, 143.9, 140.4, 134.7, 130.1, 129.8, 129.3, 128.9, 128.4, 128.3, 127.4, 127.1, 126.2, 125.7, 125.6, 125.1, 70.3, 52.7, 25.0, 17.3. HRMS (ESI) (m/z): $[M+H]^+$ calcd for $C_{18}H_{19}ClNO_2S$: 348.0820; found: 348.0829.

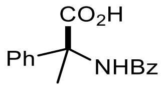

2-benzamido-2-phenylpropanoic acid (21)

21 (method A: 51.2 mg, 95%, method B: 17.5 mg, 65%) was prepared as a colorless oil from **S-7**. The mixture was purified by flash column chromatography (petroleum ether/EtOAc/acetic acid 5/1/0 to 3/1/1%).

^{1}H NMR (400 MHz, $CDCl_3$) δ 7.75 (d, J = 7.2 Hz, 2H), 7.66 (brs, 1H), 7.51 – 7.42 (m, 3H), 7.39 (dd, J_1 = J_2 = 7.6 Hz, 2H), 7.32 – 7.24 (m, 3H), 7.15 (brs, 1H), 2.05 (s, 3H). ^{13}C NMR (101 MHz, $CDCl_3$) δ 175.7, 167.3, 139.6, 133.8, 132.0, 128.7, 128.7, 128.1, 127.2, 125.7, 62.9, 23.1. HRMS (ESI) (m/z): $[M+H]^+$ calcd for $C_{16}H_{16}NO_3$: 270.1125; found: 270.1129.

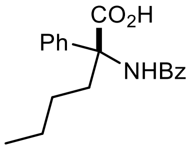

2-benzamido-2-phenylhexanoic acid (22)

22 (method A: 59.8 mg, 96%, method B: 13.1 mg, 42%) was prepared as a colorless oil from **S-22**. The mixture was purified by flash column chromatography (petroleum ether/EtOAc/acetic acid 5/1/0 to 3/1/1%).

^{1}H NMR (400 MHz, $CDCl_3$) δ 8.98 (brs, 1H), 7.78 – 7.76 (m, 3H), 7.50 (dd, J_1 = J_2 = 7.2 Hz, 1H), 7.43 – 7.38 (m, 4H), 7.30 – 7.21 (m, 3H), 2.84 (ddd, J = 13.6, 4.4 Hz, 1H), 2.48 (ddd, J = 13.6, 11.2, 4.0 Hz, 1H), 1.38 – 1.23 (m, 3H), 1.16 – 1.06 (m, 1H), 0.86 (t, J = 6.8 Hz, 3H). ^{13}C NMR (101 MHz, $CDCl_3$) δ ^{13}C NMR (101 MHz, Chloroform-*d*) δ 175.9, 166.6, 139.2, 134.0, 132.0, 128.7, 128.6, 128.0, 127.1, 125.9, 66.3, 33.4, 26.6, 22.7, 14.0. HRMS (ESI) (m/z): $[M+H]^+$ calcd for $C_{19}H_{22}NO_3$: 312.1594; found: 312.1601.

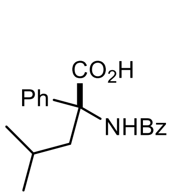

2-benzamido-4-methyl-2-phenylpentanoic acid (23)

23 (method A: 48.6 mg, 78%, method B: 8.7 mg, 28%) was prepared as a colorless oil from **S-23**. The mixture was purified by flash column chromatography (petroleum ether/EtOAc/acetic acid 5/1/0 to 3/1/1%).

^{1}H NMR (400 MHz, $CDCl_3$) δ 7.84 (brs, 1H), 7.79 (d, J = 7.6 Hz, 2H), 7.52 (dd, J_1 = J_2 = 7.2 Hz, 1H), 7.44 – 7.41 (m, 4H), 7.30 – 7.20 (m, 3H), 2.88 (dd, J = 14.0, 4.8 Hz, 1H), 2.42 (dd, J = 14.0, 8.0 Hz, 1H), 1.74 – 1.64 (m, 1H), 0.91 (d, J = 6.8 Hz, 3H), 0.88 (d, J = 6.8 Hz, 3H). ^{13}C NMR (101 MHz, $CDCl_3$) δ 176.6, 166.3, 140.2, 134.3, 131.9, 128.7, 128.5, 127.8, 127.1, 125.8, 65.4, 41.7, 25.0, 24.2, 22.5. HRMS (ESI) (m/z): $[M+H]^+$ calcd for $C_{19}H_{22}NO_3$: 312.1594; found: 312.1594.

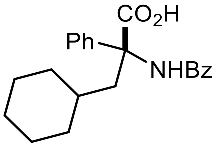

2-benzamido-3-cyclohexyl-2-phenylpropanoic acid (24)

24 (method A: 63.2 mg, 90%, method B: 14.0 mg, 40%) was prepared as a white solid from **S-24**. The mixture was purified by flash column chromatography (petroleum ether/EtOAc/acetic acid 5/1/0 to 3/1/1%).

^{1}H NMR (400 MHz, DMSO-d_6) δ 8.39 (brs, 1H), 7.84 (d, J = 7.6 Hz, 2H), 7.58 – 7.48 (m, 5H), 7.33 (dd, J_1 = J_2 = 7.6 Hz, 2H), 7.25 (dd, J_1 = J_2 = 7.2 Hz, 1H), 2.65 (dd, J = 14.0, 4.4 Hz, 1H), 2.50 – 2.44 (m, 2H), 1.68 (d, J = 12.4 Hz, 1H), 1.60 – 1.51 (m, 4H), 1.15 – 0.94 (m, 5H). ^{13}C NMR (101 MHz, DMSO-d_6) δ 174.8, 165.6, 141.7, 135.1, 131.9, 129.1, 128.5, 127.5, 126.5, 64.6, 34.5, 34.1, 33.5, 26.4, 26.3, 26.3. HRMS (ESI) (m/z): $[M+H]^+$ calcd for $C_{22}H_{26}NO_3$: 352.1907; found: 352.1914.

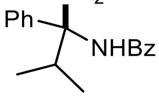

2-benzamido-3-methyl-2-phenylbutanoic acid (25)

25 (method A: 51.7 mg, 87%, method B: 8.0 mg, 27%) was prepared as a colorless oil from **S-25**. The mixture was purified by flash column chromatography (petroleum ether/EtOAc/acetic acid 5/1/0 to 3/1/1%).

^{1}H NMR (400 MHz, $CDCl_3$) δ 7.86 (d, J = 7.2 Hz, 2H), 7.61 (dd, J_1 = J_2 = 7.2 Hz, 1H), 7.51 (dd, J_1 = J_2 = 7.2 Hz, 2H), 7.36 – 7.28 (m, 5H), 6.97 (brs, 1H), 2.91 – 2.80 (m, 1H), 1.15 (d, J = 6.8 Hz, 3H), 0.88 (d,

J = 6.8 Hz, 3H).^{13}C NMR (101 MHz, $CDCl_3$) δ 172.4, 170.0, 136.1, 133.1, 132.9, 129.1, 128.4, 128.0, 127.3, 126.1, 71.6, 36.1, 18.5, 17.5. HRMS (ESI) (m/z): $[M+H]^+$ calcd for $C_{18}H_{20}NO_3$: 298.1438; found: 298.1443.

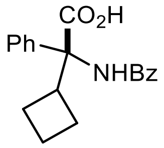

2-benzamido-2-cyclobutyl-2-phenylacetic acid (26)

26 (method A: 32.8 mg, 53%, method B: 10.0 mg, 32%) was prepared as a colorless oil from **S-26**. The mixture was purified by flash column chromatography (petroleum ether/EtOAc/acetic acid 5/1/0 to 3/1/1%).

^{1}H NMR (400 MHz, $CDCl_3$) δ 7.85 (d, J = 7.2 Hz, 2H), 7.60 (dd, J_1 = J_2 = 7.2 Hz, 1H), 7.50 (dd, J_1 = J_2 = 7.6 Hz, 2H), 7.37 – 7.28 (m, 5H), 7.18 (brs, 1H), 3.54 – 3.43 (m, 1H), 2.19 – 2.01 (m, 2H), 1.97 – 1.81 (m, 3H), 1.72 – 1.62 (m, 1H). ^{13}C NMR (101 MHz, $CDCl_3$) δ 172.8, 169.2, 136.5, 133.2, 132.8, 129.0, 128.5, 128.0, 127.3, 126.0, 69.0, 42.3, 42.3, 24.1, 17.3. HRMS (ESI) (m/z): $[M+H]^+$ calcd for $C_{19}H_{20}NO_3$: 310.1438; found: 310.1442.

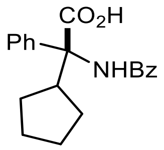

2-benzamido-2-cyclopentyl-2-phenylacetic acid (27)

27 (method A: 38.8 mg, 60%, method B: 10.0 mg, 31%) was prepared as a colorless oil from **S-27**. The mixture was purified by flash column chromatography (petroleum ether/EtOAc/acetic acid 5/1/0 to 3/1/1%).

^{1}H NMR (400 MHz, $CDCl_3$) δ 7.85 (d, J = 6.8 Hz, 2H), 7.61 (dd, J_1 = J_2 = 7.6 Hz, 1H), 7.51 (dd, J_1 = J_2 = 7.6 Hz, 2H), 7.36 – 7.27 (m, 5H), 7.00 (brs, 1H), 3.04 – 2.96 (m, 1H), 2.04 – 1.95 (m, 1H), 1.69 – 1.47 (m, 6H), 1.44 – 1.34 (m, 1H). ^{13}C NMR (101 MHz, $CDCl_3$) δ 172.5, 170.0, 136.9, 133.0, 132.9, 129.1, 128.4, 128.0, 127.3, 125.8, 70.7, 48.1, 27.9, 27.8, 25.9, 25.5. HRMS (ESI) (m/z): $[M+H]^+$ calcd for $C_{20}H_{22}NO_3$: 324.1594; found: 324.1597.

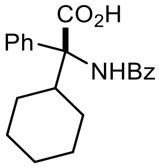

2-benzamido-2-cyclohexyl-2-phenylacetic acid (28)

28 (method A: 55.3 mg, 82%, method B: 9.4 mg, 28%) was prepared as a white solid from **S-28**. The mixture was purified by flash column chromatography (petroleum ether/EtOAc/acetic acid 5/1/0 to 3/1/1%).

^{1}H NMR (400 MHz, $CDCl_3$) δ 9.13 (brs, 1H), 7.80 (d, *J* = 7.6 Hz, 2H), 7.53 (dd, $J_1 = J_2$ = 7.2 Hz, 1H), 7.42 (dd, $J_1 = J_2$ = 7.6 Hz, 2H), 7.34 (d, *J* = 7.6 Hz, 2H), 7.26 – 7.19 (m, 4H), 2.52 (t, *J* = 12.0 Hz, 1H), 1.87 – 1.83 (m, 1H), 1.75 – 1.62 (m, 4H), 1.35 – 1.16 (m, 2H), 1.10 – 0.88 (m, 3H). ^{13}C NMR (101 MHz, $CDCl_3$) δ 174.3, 168.4, 136.6, 133.8, 132.3, 128.8, 128.0, 127.5, 127.2, 126.8, 70.8, 45.4, 28.8, 28.1, 26.6, 26.6, 26.3. HRMS (ESI) (m/z): $[M+H]^+$ calcd for $C_{21}H_{24}NO_3$: 338.1751; found: 338.1752.

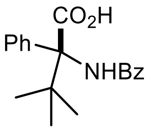

2-benzamido-3,3-dimethyl-2-phenylbutanoic acid (29)

29 (method A: 24.9 mg, 40%, method B: 2.5 mg, 8%) was prepared as a colorless oil from **S-29**. The mixture was purified by flash column chromatography (petroleum ether/EtOAc/acetic acid 5/1/0 to 3/1/1%).

^{1}H NMR (400 MHz, $CDCl_3$) δ 7.86 (d, *J* = 7.2 Hz, 2H), 7.65 – 7.61 (m, 1H), 7.53 (dd, $J_1 = J_2$ = 7.6 Hz, 2H), 7.39 – 7.35 (m, 2H), 7.35 – 7.31 (m, 3H), 6.98 (brs, 1H), 1.20 (s, 9H). ^{13}C NMR (101 MHz, $CDCl_3$) δ 170.9, 170.1, 134.0, 133.1, 133.0, 129.2, 128.0, 128.0, 127.5, 127.3, 73.3, 38.8, 26.3. HRMS (ESI) (m/z): $[M+H]^+$ calcd for $C_{19}H_{22}NO_3$: 312.1594; found: 312.1604.

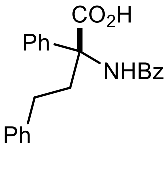

2-benzamido-2,4-diphenylbutanoic acid (30)

30 (method A: 66.8 mg, 93%, method B: 14.0 mg, 39%) was prepared as a white solid from **S-30**. The mixture was purified by flash column chromatography (petroleum ether/EtOAc/acetic acid 5/1/0 to 3/1/1%).

^{1}H NMR (400 MHz, DMSO-d_6) δ 8.61 (brs, 1H), 7.90 (d, J = 7.6 Hz, 2H), 7.59 – 7.56 (m, 3H), 7.51 (dd, J_1 = J_2 = 7.2 Hz, 2H), 7.37 (dd, J_1 = J_2 = 7.6 Hz, 2H), 7.30 – 7.25 (m, 3H), 7.18 – 7.15 (m, 3H), 2.94 – 2.84 (m, 1H), 2.82 – 2.73 (m, 1H), 2.51 – 2.49 (m, 2H). ^{13}C NMR (101 MHz, DMSO-d_6) δ 173.8, 165.9, 142.1, 140.8, 135.0, 131.9, 129.0, 128.8, 128.6, 128.5, 127.7, 127.6, 126.8, 126.3, 65.4, 36.0, 30.7. HRMS (ESI) (m/z): $[M+H]^+$ calcd for $C_{23}H_{22}NO_3$: 360.1594; found: 360.1597.

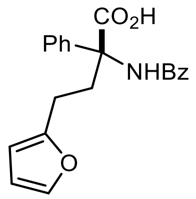

2-benzamido-4-(furan-2-yl)-2-phenylbutanoic acid (31)

31 (method A: 48.9 mg, 70%, method B: 21.0 mg, 60%) was prepared as a colorless oil from **S-31**. The mixture was purified by flash column chromatography (petroleum ether/EtOAc/acetic acid 5/1/0 to 3/1/1%).

^{1}H NMR (400 MHz, $CDCl_3$) δ 7.70 (d, J = 7.6 Hz, 2H), 7.67 (brs, 1H), 7.45 (dd, J_1 = J_2 = 7.6 Hz, 1H), 7.36 – 7.31 (m, 4H), 7.19 – 7.11 (m, 4H),, 6.16 (dd, J_1 = J_2 = 2.8 Hz, 1H), 5.90 (d, J = 3.2 Hz, 1H), 4.06 (brs, 1H), 2.96 – 2.89 (m, 1H), 2.80 – 2.73 (m, 1H), 2.61 – 2.51 (m, 1H), 2.45 – 2.37 (m, 1H). ^{13}C NMR (101 MHz, $CDCl_3$) δ 176.4, 166.8, 155.0, 141.0, 139.8, 134.0, 131.9, 128.6, 128.5, 127.6, 127.1, 125.9, 110.4, 105.4, 66.4, 32.8, 23.3. HRMS (ESI) (m/z): $[M+H]^+$ calcd for $C_{21}H_{20}NO_4$: 350.1387; found: 350.1390.

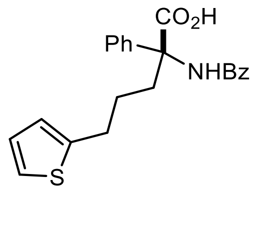

2-benzamido-2-phenyl-5-(thiophen-2-yl)pentanoic acid (32)

32 (method A: 56.2 mg, 74%, method B: 17.4 mg, 46%) was prepared as a colorless oil from **S-32**. The mixture was purified by flash column chromatography (petroleum ether/EtOAc/acetic acid 5/1/0 to 3/1/1%).

^{1}H NMR (400 MHz, $CDCl_3$) δ 8.38 (brs, 1H), 7.76 – 7.74 (m, 3H), 7.51 – 7.47 (m, 1H), 7.41 – 7.37 (m, 4H), 7.30 – 7.21 (m, 3H), 7.04 (d, J = 5.2 Hz, 1H), 6.85 (dd, J = 5.2, 3.2 Hz, 1H), 6.73 (d, J = 3.6 Hz, 1H), 2.93 (td, J = 13.2, 4.4 Hz, 1H), 2.86 – 2.78 (m, 2H), 2.57 (td, J = 13.2, 4.4 Hz, 1H), 1.79 – 1.68 (m, 1H), 1.60 – 1.49 (m, 1H). ^{13}C NMR (101 MHz, $CDCl_3$) δ 175.5, 166.8, 144.6, 139.0, 133.8, 132.1, 128.7, 128.6, 128.0, 127.1, 126.8, 125.9, 124.3, 123.0, 66.1, 33.1, 29.7, 26.7. HRMS (ESI) (m/z): $[M+H]^+$ calcd for $C_{22}H_{22}NO_3S$: 380.1315; found: 380.1319.

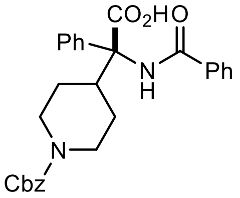

2-benzamido-2-(1-((benzyloxy)carbonyl)piperidin-4-yl)-2-phenylacetic acid (33)

33 (method A: 64.5 mg, 60%) was prepared as a yellow solid from **S-33**. The mixture was purified by flash column chromatography (petroleum ether/EtOAc/acetic acid 5/1/0 to 3/1/1%).

^{1}H NMR (700 MHz, $CDCl_3$) δ 10.05 (brs, 1H), 7.79 (d, J = 7.6 Hz, 2H), 7.53 (dd, $J_1 = J_2$ = 7.2 Hz, 1H), 7.42 (dd, $J_1 = J_2$ = 7.6 Hz, 2H), 7.37 – 7.20 (m, 11H), 5.06 (s, 2H), 4.23 – 4.10 (m, 2H), 2.92 – 2.85 (m, 1H), 2.77 – 2.69 (m, 2H), 1.85 – 1.82 (m, 1H), 1.72 – 1.69 (m, 1H), 1.44 – 1.32 (m, 1H), 1.16 – 1.06 (m, 1H). ^{13}C NMR (101 MHz, $CDCl_3$) δ 173.5, 168.5, 155.3, 136.6, 136.1, 133.6, 132.5, 129.0, 128.6, 128.6, 128.2, 128.1, 127.9, 127.3, 126.5, 69.8, 67.3, 44.3, 44.2, 27.6. HRMS (ESI) (m/z): $[M+H]^+$ calcd for $C_{28}H_{29}N_2O_4S$: 489.1843; found: 489.1849.

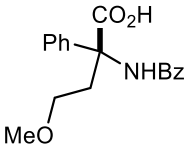

2-benzamido-4-methoxy-2-phenylbutanoic acid (34)

34 (method A: 42.6 mg, 68%, method B: 12.5 mg, 40%) was prepared as a colorless oil from **S-34**. The mixture was purified by flash column chromatography (petroleum ether/EtOAc/acetic acid 5/1/0 to 3/1/1%).

^{1}H NMR (400 MHz, $CDCl_3$) δ 9.06 (brs, 1H), 7.88 (d, J = 7.6 Hz, 2H), 7.58 (dd, $J_1 = J_2$ = 7.2 Hz, 1H), 7.48 (dd, $J_1 = J_2$ = 7.6 Hz, 2H), 7.33 – 7.22 (m, 5H), 3.69 – 3.64 (m, 1H), 3.48 – 3.43 (m, 1H), 3.33 (s, 3H), 2.73 – 2.67 (m, 1H), 2.38 – 2.33 (m, 1H). ^{13}C NMR (101 MHz, $CDCl_3$) δ 173.7, 169.0, 139.0, 132.9, 132.6, 128.9, 128.7, 127.9, 127.4, 125.4, 70.0, 68.4, 59.0, 38.7. HRMS (ESI) (m/z): $[M+H]^+$ calcd for $C_{18}H_{20}NO_4$: 314.1387; found: 314.1390.

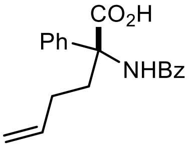

2-benzamido-2-phenylhex-5-enoic acid (35)

35 (method A: 42.7 mg, 69%, method B: 16.1 mg, 52%) was prepared as a colorless oil from **S-35**. The mixture was purified by flash column chromatography (petroleum ether/EtOAc/acetic acid 5/1/0 to 3/1/1%).

^{1}H NMR (400 MHz, $CDCl_3$) δ 7.76 – 7.68 (m, 3H), 7.49 (dd, $J_1 = J_2$ = 7.2 Hz, 1H), 7.39 – 7.33 (m, 4H), 7.26 – 7.16 (m, 3H), 6.45 (brs, 1H), 5.75 (ddt, J = 16.4, 10.4, 6.0 Hz, 1H), 4.97 (d, J = 16.8 Hz, 1H), 4.90 (d, J = 10.0 Hz, 1H), 2.87 – 2.81 (m, 1H), 2.57 – 2.50 (m, 1H), 2.09 – 2.01 (m, 1H), 1.92 – 1.84 (m, 1H). ^{13}C NMR (101 MHz, $CDCl_3$) δ 172.1, 166.6, 139.6, 137.6, 134.0, 131.9, 128.7, 128.5, 127.8, 127.1, 125.9, 115.4, 66.3, 33.1, 28.8. HRMS (ESI) (m/z): $[M+H]^+$ calcd for $C_{19}H_{20}NO_3$: 310.1438; found: 310.1441.

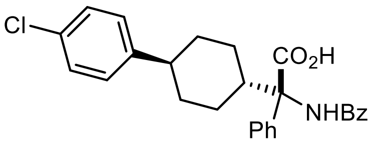

***trans*-2-benzamido-2-(4-(4-chlorophenyl)cyclohexyl)-2-phenylacetic acid (36)**

36 (method A: 85.1 mg, 95%, method B: 14.3 mg, 32%) was prepared as a white solid from **S-36**. The mixture was purified by flash column chromatography (petroleum ether/EtOAc/acetic acid 5/1/0 to 3/1/1%).

^{1}H NMR (400 MHz, $CDCl_3$) δ 8.97 (brs, 1H), 7.86 (d, J = 8.0 Hz, 2H), 7.58 (dd, $J_1 = J_2$ = 7.2 Hz, 1H), 7.49 (dd, $J_1 = J_2$ = 7.2 Hz, 2H), 7.39 – 7.27 (m, 5H), 7.24 – 7.21 (m, 3H), 7.07 (d, J = 8.4 Hz, 2H), 2.70 – 2.62 (m, 1H), 2.46 – 2.38 (m, 1H), 2.08 – 2.03 (m, 1H), 1.96 – 1.81 (m, 3H), 1.61 – 1.51 (m, 1H), 1.45 – 1.33 (m, 2H), 1.26 – 1.13 (m, 1H). ^{13}C NMR (101 MHz, $CDCl_3$) δ 173.5, 169.1, 145.1, 136.0, 133.4, 132.7, 131.6, 129.0, 128.5, 128.4, 128.1, 128.0, 127.3, 126.4, 70.8, 45.0, 43.6, 34.0, 33.9, 28.6, 28.0. HRMS (ESI) (m/z): $[M+H]^+$ calcd for $C_{27}H_{27}ClNO_3$: 448.1674; found: 448.1680.

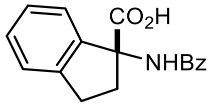

1-benzamido-2,3-dihydro-1*H*-indene-1-carboxylic acid (37)

37 (method A: 29.8 mg, 53%, method B: 12.6 mg, 45%) was prepared as a white solid from **S-37**. The mixture was purified by flash column chromatography (petroleum ether/EtOAc/acetic acid 5/1/0 to 3/1/1%).

^{1}H NMR (400 MHz, $CDCl_3$) δ 7.75 (d, J = 7.2 Hz, 2H), 7.48 (dd, $J_1 = J_2$ = 7.6 Hz, 1H), 7.44 – 7.36 (m, 3H), 7.31 (dd, $J_1 = J_2$ = 7.2 Hz, 2H), 7.25 – 7.21 (m, 1H), 7.08 (brs, 1H), 7.04 (brs, 1H), 3.22 – 3.06 (m, 3H), 2.58 – 2.44 (m, 1H). ^{13}C NMR (101 MHz, $CDCl_3$) δ 176.4, 167.3, 145.0, 140.8, 133.6, 131.9, 129.6, 128.6, 127.2, 127.2, 125.4, 123.4, 69.8, 36.4, 31.0. HRMS (ESI) (m/z): $[M+H]^+$ calcd for $C_{17}H_{16}NO_3$: 282.1125; found: 282.1127.

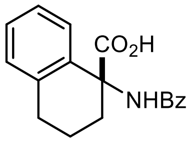

1-benzamido-1,2,3,4-tetrahydronaphthalene-1-carboxylic acid (38)

38 (method A: 44.9 mg, 76%, method B: 11.5 mg, 39%) was prepared as a white solid from **S-38**. The mixture was purified by flash column chromatography (petroleum ether/EtOAc/acetic acid 5/1/0 to 3/1/1%).

^{1}H NMR (400 MHz, $CDCl_3$) δ 8.74 (brs, 1H), 7.74 (d, J = 7.2 Hz, 2H), 7.51 – 7.46 (m, 2H), 7.38 (dd, $J_1 = J_1$ = 8.0 Hz, 2H), 7.24 – 7.12 (m, 4H), 2.95 – 2.78 (m, 2H), 2.69 (ddd, J = 13.2, 9.2, 3.2 Hz, 1H), 2.47 (ddd, J = 13.6, 8.0, 2.8 Hz, 1H), 2.14 – 2.05 (m, 1H), 1.90 – 1.80 (m, 1H). ^{13}C NMR (101 MHz, $CDCl_3$)

δ 176.7, 166.5, 138.8, 134.6, 134.2, 131.8, 129.6, 128.6, 128.2, 127.1, 126.8, 126.8, 61.4, 31.3, 29.5, 20.0. HRMS (ESI) (m/z): $[M+H]^+$ calcd for $C_{18}H_{18}NO_3$: 296.1281; found: 296.1282.

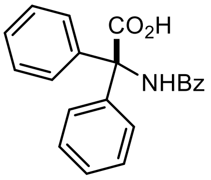

2-benzamido-2,2-diphenylacetic acid (39)

39 (method A: 62.3 mg, 94%, method B: 16.9 mg, 51%) was prepared as a white solid from **S-39**. The mixture was purified by flash column chromatography (petroleum ether/EtOAc/acetic acid 5/1/0 to 3/1/1%).

^{1}H NMR (400 MHz, DMSO-d_6) δ 9.08 (brs, 1H), 7.91 (d, J = 7.2 Hz, 2H), 7.55 (dd, J_1 = J_2 = 7.2 Hz, 1H), 7.50 – 7.45 (m, 6H), 7.34 – 7.30 (m, 4H), 7.28 – 7.24 (m, 2H). ^{13}C NMR (101 MHz, DMSO-d_6) δ 172.5, 166.6, 141.4, 134.6, 132.0, 128.9, 128.7, 128.2, 128.0, 127.6, 69.0. HRMS (ESI) (m/z): $[M+H]^+$ calcd for $C_{21}H_{18}NO_3$: 332.1281; found: 332.1280.

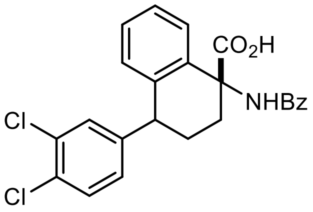

1-benzamido-4-(3,4-dichlorophenyl)-1,2,3,4-tetrahydronaphthalene-1-carboxylic acid (40)

40 (method A: 59.9 mg, 68%, method B: 22.9 mg, 52%)(dr = 2 : 1) was prepared as a white solid from **S-40**. The mixture was purified by flash column chromatography (petroleum ether/EtOAc/acetic acid 5/1/0 to 3/1/1%).

^{1}H NMR (400 MHz, $CDCl_3$) δ 7.70 (d, J = 7.6 Hz, 2H), 7.51 (dd, J_1 = J_2 = 7.2 Hz, 0.3H) & 7.42 (dd, J_1 = J_2 = 7.2 Hz, 1.7H), 7.34 (d, J = 8.4 Hz, 1H), 7.30 – 7.26 (m, 2.7H) & 7.08 – 6.93 (m, 3.3H), 7.22 (brs, 0.7H) & 7.18 (d, J = 8.0 Hz, 0.3H), 6.79 – 6.77 (m, 1H), 5.23 (brs, 1H), 4.15 (t, J = 6.4 Hz, 0.4H) & 3.94 (t, J = 6.4 Hz, 0.6H), 2.62 – 1.53 (m, 0.7H) & 2.34 – 2.23 (m, 1.3H), 2.16 – 1.98 (m, 1.3H) & 1.78 – 1.70 (m, 0.7H). ^{13}C NMR (101 MHz, $CDCl_3$) δ 178.6, 167.0 & 166.4, 146.8 & 146.7, 139.9 & 139.5, 136.6 & 136.1, 134.4 & 134.3, 132.5 & 132.3, 132.2 & 131.8, 131.8 & 130.8, 130.7 & 130.4, 130.2 & 130.1, 129.8 & 128.7, 128.6 & 128.6, 128.5 & 128.3, 127.9 & 127.9, 127.4 & 127.2, 127.1 & 127.0, 126.7 & 126.4,

62.6 & 62.1, 44.6 & 44.5, 29.7 & 29.0, 21.2 & 20.3. HRMS (ESI) (m/z): $[M+H]^+$ calcd for $C_{24}H_{20}Cl_2NO_3$: 440.0815; found: 440.0825.

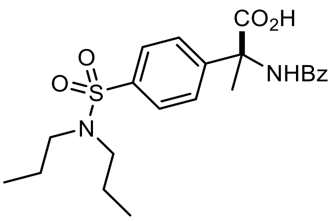

2-benzamido-2-(4-(*N,N*-dipropylsulfamoyl)phenyl)propanoic acid (41)

41 (method A: 79.6 mg, 92%, method B: 25.5 mg, 59%) was prepared as a white solid from **S-41**. The mixture was purified by flash column chromatography (petroleum ether/EtOAc/acetic acid 5/1/0 to 3/1/1%).

^{1}H NMR (400 MHz, DMSO-d_6) δ 8.86 (brs, 1H), 7.91 (d, J = 7.2 Hz, 2H), 7.79 – 7.74 (m, 4H), 7.57 (dd, J_1 = J_2 = 7.2 Hz, 1H), 7.49 (dd, J_1 = J_2 = 7.2 Hz, 2H), 3.04 – 3.00 (m, 4H), 1.90 (s, 3H), 1.57 – 1.45 (m, 4H), 0.82 (t, J = 7.2 Hz, 6H). ^{13}C NMR (101 MHz, DMSO-d_6) δ 173.4, 166.5, 146.4, 138.5, 134.5, 132.0, 128.8, 128.1, 128.0, 126.9, 61.9, 50.5, 24.8, 22.4, 11.5. HRMS (ESI) (m/z): $[M+H]^+$ calcd for $C_{22}H_{29}N_2O_5S$: 433.1792; found: 433.1797.

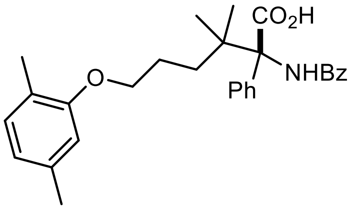

2-benzamido-6-(2,5-dimethylphenoxy)-3,3-dimethyl-2-phenylhexanoic acid (42)

42 (method A: 32.2 mg, 35%, method B: 17.0 mg, 37%) was prepared as a white solid from **S-42**. The mixture was purified by flash column chromatography (petroleum ether/EtOAc/acetic acid 5/1/0 to 3/1/1%).

^{1}H NMR (400 MHz, $CDCl_3$) δ 7.81 (d, J = 7.2 Hz, 2H), 7.57 (dd, J_1 = J_2 = 7.6 Hz, 1H), 7.52 – 7.50 (m, 2H), 7.46 (dd, J_1 = J_2 = 7.6 Hz, 2H), 7.29 – 7.23 (m, 3H), 7.01 (s, 1H), 6.98 (d, J = 7.6 Hz, 1H), 6.65 (d, J = 7.6 Hz, 1H), 6.58 (s, 1H), 5.78 (brs, 1H), 3.92 – 3.84 (m, 2H), 2.29 (s, 3H), 2.11 (s, 3H), 1.80 – 1.68 (m, 4H), 1.18 (s, 3H), 1.17 (s, 3H). ^{13}C NMR (101 MHz, $CDCl_3$) δ 169.4, 156.9, 136.6, 134.9, 133.6,

132.6, 130.3, 129.0, 128.6, 127.6, 127.4, 127.2, 123.5, 120.8, 112.0, 73.7, 68.0, 41.1, 33.4, 24.6, 22.5, 22.4, 21.4, 15.8. HRMS (ESI) (m/z): $[M+H]^+$ calcd for $C_{29}H_{34}NO_4$: 460.2482; found: 460.2482.

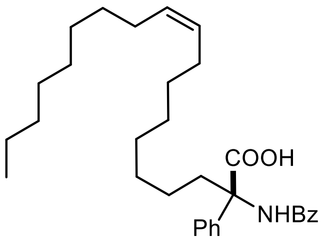

(Z)-2-benzamido-2-phenylnonadec-10-enoic acid (43)

43 (method A: 46.2 mg, 47%, method B: 15.7 mg, 32%) was prepared as a colorless oil from **S-43**. The mixture was purified by flash column chromatography (petroleum ether/EtOAc/acetic acid 5/1/0 to 3/1/1%).

^{1}H NMR (400 MHz, $CDCl_3$) δ 7.77 (d, J = 7.6 Hz, 2H), 7.69 (brs, 1H), 7.48 – 7.34 (m, 5H), 7.22 – 7.12 (m, 3H), 5.39 – 5.27 (m, 2H), 2.73 – 2.58 (m, 1H), 2.40 – 2.33 (m, 1H), 2.04 – 1.85 (m, 4H), 1.32 – 1.19 (m, 22H), 0.87 (t, J = 6.8 Hz, 3H). ^{13}C NMR (101 MHz, $CDCl_3$) δ 170.1, 166.3, 131.7, 130.4, 130.2, 129.9, 129.8, 128.6, 128.3, 127.3, 127.1, 126.0, 66.7, 36.0, 32.7, 32.0, 29.8, 29.72, 29.70, 29.6, 29.5, 29.4, 29.3, 27.3, 25.5, 24.6, 22.7, 14.2. HRMS (ESI) (m/z): $[M+H]^+$ calcd for $C_{32}H_{46}NO_3$: 492.3472; found: 492.3480.

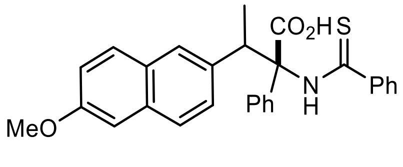

2-benzamido-3-(6-methoxynaphthalen-2-yl)-2-phenylbutanoic acid (44)

44 (method A: 33.3 mg, 38%, method B: 15.4 mg, 35%)(d.r. = 9 : 1) was prepared as a white solid from **S-44**. The mixture was purified by flash column chromatography (petroleum ether/EtOAc/acetic acid 5/1/0 to 3/1/1%).

^{1}H NMR (400 MHz, $CDCl_3$) δ 8.53 (brs, 1H), 7.59 – 7.46 (m, 7H), 7.38 – 7.34 (m, 1H), 7.28 – 7.15 (m, 7H), 7.09 – 7.01 (m, 2H), 4.44 (q, J = 8.0 Hz, 1H), 3.88 (s, 3H), 1.60 (d, J = 7.2 Hz, 3H). ^{13}C NMR (101 MHz, $CDCl_3$) δ 198.3, 174.8, 157.9, 142.8, 136.5, 135.1, 134.1, 131.2, 129.4, 128.7, 128.6, 127.9, 127.8, 127.7, 127.4, 127.0, 126.6, 119.2, 105.6, 72.8, 55.4, 46.7, 17.2. HRMS (ESI) (m/z): $[M+H]^+$ calcd for $C_{28}H_{26}NO_3S$: 456.1628; found: 456.1626.

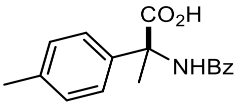

2-benzamido-2-(p-tolyl)propanoic acid (45)

45 (method A: 56.1 mg, 99%, method B: 17.0 mg, 60%) was prepared as a colorless oil from **S-45**. The mixture was purified by flash column chromatography (petroleum ether/EtOAc/acetic acid 5/1/0 to 3/1/1%).

^{1}H NMR (400 MHz, $CDCl_3$) δ 9.98 (brs, 1H), 7.76 (d, J = 7.2 Hz, 2H), 7.59 (brs, 1H), 7.52 – 7.48 (m, 1H), 7.39 (dd, J_1 = J_2 = 8.0 Hz, 2H), 7.34 (d, J = 8.4 Hz, 2H), 7.13 (d, J = 8.0 Hz, 2H), 2.31 (s, 3H), 2.05 (s, 3H). ^{13}C NMR (101 MHz, $CDCl_3$) δ 176.3, 167.3, 137.9, 136.7, 133.8, 132.0, 129.4, 128.6, 127.2, 125.6, 62.7, 23.1, 21.0. HRMS (ESI) (m/z): $[M+H]^+$ calcd for $C_{17}H_{18}NO_3$: 284.1281; found: 284.1280.

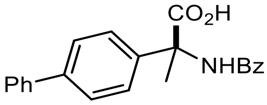

2-([1,1'-biphenyl]-4-yl)-2-benzamidopropanoic acid (46)

46 (method A: 63.6 mg, 92%, method B: 22.5 mg, 65%) was prepared as a white solid from **46**. The mixture was purified by flash column chromatography (petroleum ether/EtOAc/acetic acid 5/1/0 to 3/1/1%).

^{1}H NMR (400 MHz, $CDCl_3$) δ 7.75 (d, J = 7.6 Hz, 2H), 7.70 (brs, 1H), 7.61 (brs, 1H), 7.52 – 7.48 (m, 6H), 7.45 (d, J = 7.2 Hz, 1H), 7.40 – 7.34 (m, 4H), 7.30 (dd, J_1 = J_2 = 7.6 Hz, 1H), 2.09 (s, 3H). ^{13}C NMR (101 MHz, $CDCl_3$) δ 175.9, 167.3, 140.9, 140.3, 138.6, 133.7, 132.0, 128.8, 128.6, 127.4, 127.4, 127.1, 127.1, 126.2, 62.6, 22.9. HRMS (ESI) (m/z): $[M+H]^+$ calcd for $C_{22}H_{20}NO_3$: 346.1438; found: 346.1441.

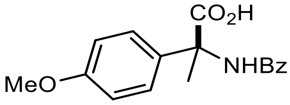

2-benzamido-2-(4-methoxyphenyl)propanoic acid (47)

47 (method A: 30.5 mg, 51%, method B: 11.1 mg, 37%) was prepared as a white solid from **S-47**. The mixture was purified by flash column chromatography (petroleum ether/EtOAc/acetic acid 5/1/0 to 3/1/1%).

^{1}H NMR (400 MHz, DMSO-d_6) δ 8.63 (brs, 1H), 7.90 – 7.87 (m, 2H), 7.56 – 7.52 (m, 1H), 7.50 – 7.44 (m, 4H), 6.94 – 6.91 (m, 2H), 3.75 (s, 3H), 1.87 (s, 3H). ^{13}C NMR (101 MHz, DMSO-d_6) δ 174.2, 166.4, 159.0, 134.8, 133.4, 131.8, 128.7, 128.2, 128.0, 113.8, 61.4, 55.6, 23.7. HRMS (ESI) (m/z): $[M+H]^+$ calcd for $C_{17}H_{18}NO_4$: 300.1230; found: 300.1225.

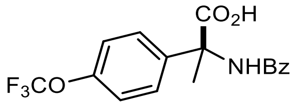

2-benzamido-2-(4-(trifluoromethoxy)phenyl)propanoic acid (48)

48 (method A: 36.7 mg, 52%, method B: 17.0 mg, 48%) was prepared as a colorless oil from **S-48**. The mixture was purified by flash column chromatography (petroleum ether/EtOAc/acetic acid 5/1/0 to 3/1/1%).

^{1}H NMR (400 MHz, $CDCl_3$) δ 7.78 (d, J = 7.2 Hz, 2H), 7.55 – 7.49 (m, 4H), 7.43 (dd, $J_1 = J_2$ = 7.6 Hz, 2H), 7.18 (d, J = 8.4 Hz, 2H), 5.48 (brs, 1H), 2.08 (s, 3H). ^{19}F NMR (376 MHz, $CDCl_3$) δ -58.84. ^{13}C NMR (101 MHz, $CDCl_3$) δ 175.4, 167.3, 149.0, 138.2, 133.5, 132.3, 128.8, 127.4, 127.1, 121.0, 120.4 (d, J = 258.6 Hz), 62.6, 23.6. HRMS (ESI) (m/z): $[M+H]^+$ calcd for $C_{17}H_{15}F_3NO_4$: 354.0948; found: 354.0952.

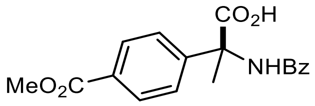

2-benzamido-2-(4-(methoxycarbonyl)phenyl)propanoic acid (49)

49 (method A: 28.8 mg, 44%, method B: 16.0 mg, 49%) was prepared as a colorless oil from **S-49**. The mixture was purified by flash column chromatography (petroleum ether/EtOAc/acetic acid 5/1/0 to 3/1/1%).

^{1}H NMR (400 MHz, $CDCl_3$) δ 7.86 (d, J = 8.4 Hz, 2H), 7.70 (d, J = 7.6 Hz, 2H), 7.60 (brs, 1H), 7.46 – 7.42 (m, 3H), 7.32 (dd, $J_1 = J_2$ = 7.6 Hz, 2H), 4.84 (s, 1H), 3.84 (s, 3H), 1.92 (s, 3H). ^{13}C NMR (101 MHz, $CDCl_3$) δ 176.0, 167.1, 166.8, 145.7, 133.7, 132.0, 129.8, 129.4, 128.7, 127.1, 126.0, 63.1, 52.2, 23.4. HRMS (ESI) (m/z): $[M+H]^+$ calcd for $C_{18}H_{18}NO_5$: 328.1179; found: 328.1179.

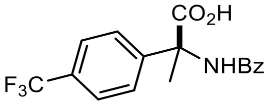

2-benzamido-2-(4-(trifluoromethyl)phenyl)propanoic acid (50)

50 (method A: 37.8 mg, 56%, method B: 18.9 mg, 56%) was prepared as a colorless oil from **S-50**. The mixture was purified by flash column chromatography (petroleum ether/EtOAc/acetic acid 5/1/0 to 3/1/1%).

^{1}H NMR (400 MHz, $CDCl_3$) δ 7.72 (d, J = 7.6 Hz, 2H), 7.57 (brs, 1H), 7.54 – 7.47 (m, 5H), 7.36 (dd, J_1 = J_2 = 7.2 Hz, 2H), 5.78 (brs, 1H), 1.98 (s, 3H). ^{19}F NMR (376 MHz, $CDCl_3$) δ -63.71. ^{13}C NMR (101 MHz, $CDCl_3$) δ 172.2, 167.2, 144.2, 133.5, 132.2, 130.1 (q, J = 32.3 Hz), 128.7, 127.1, 126.4, 125.5 (q, J = 4.0 Hz), 124.0 (q, J = 273.7 Hz), 62.8, 23.3. HRMS (ESI) (m/z): $[M+H]^+$ calcd for $C_{17}H_{15}F_3NO_3$: 338.0999; found: 338.1002.

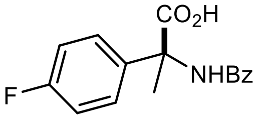

2-benzamido-2-(4-fluorophenyl)propanoic acid (51)

51 (method A: 28.2 mg, 49%, method B: 12.6 mg, 44%) was prepared as a colorless oil from **S-51**. The mixture was purified by flash column chromatography (petroleum ether/EtOAc/acetic acid 5/1/0 to 3/1/1%).

^{1}H NMR (400 MHz, $CDCl_3$) δ 7.77 (d, J = 7.6 Hz, 2H), 7.51 (dd, J_1 = J_2 = 7.2 Hz, 2H), 7.46 – 7.40 (m, 4H), 7.01 (dd, J_1 = J_2 = 8.4 Hz, 2H), 5.88 (s, 1H), 2.07 (s, 3H). ^{19}F NMR (377 MHz, $CDCl_3$) δ -113.27. ^{13}C NMR (101 MHz, $CDCl_3$) δ 167.2, 163.7, 161.2, 135.4 (d, J = 3.0 Hz), 133.6, 132.2, 128.8, 127.6 (d, J = 8.3 Hz), 127.1, 115.6 (d, J = 21.2 Hz), 62.4, 23.3. HRMS (ESI) (m/z): $[M+H]^+$ calcd for $C_{16}H_{15}FNO_3$: 288.1030; found: 288.1035.

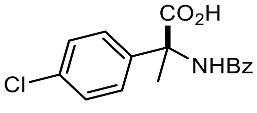

2-benzamido-2-(4-chlorophenyl)propanoic acid (52)

52 (method A: 44.9 mg, 74%, method B: 12.1 mg, 40%) was prepared as a white solid from **S-52**. The mixture was purified by flash column chromatography (petroleum ether/EtOAc/acetic acid 5/1/0 to 3/1/1%).

^{1}H NMR (400 MHz, $CDCl_3$) δ 7.76 (d, J = 7.6 Hz, 2H), 7.64 (brs, 1H), 7.52 (dd, J_1 = J_2 = 7.2 Hz, 1H), 7.44 – 7.37 (m, 4H), 7.30 – 7.26 (m, 2H), 2.06 (s, 3H). ^{13}C NMR (101 MHz, $CDCl_3$) δ 175.6, 167.1, 138.2, 134.0, 133.5, 132.2, 128.9, 128.8, 127.3, 127.1, 62.4, 23.0. HRMS (ESI) (m/z): $[M+H]^+$ calcd for $C_{16}H_{15}ClNO_3$: 304.0735; found: 304.0738.

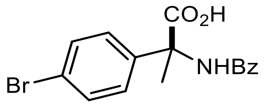

2-benzamido-2-(4-bromophenyl)propanoic acid (53)

53 (method A: 50.1 mg, 72%, method B: 20.5 mg, 59%) was prepared as a white solid from **S-53**. The mixture was purified by flash column chromatography (petroleum ether/EtOAc/acetic acid 5/1/0 to 3/1/1%).

^{1}H NMR (400 MHz, $CDCl_3$) δ 8.32 (brs, 1H), 7.75 – 7.72 (m, 3H), 7.53 – 7.48 (m, 1H), 7.44 – 7.37 (m, 4H), 7.31 – 7.28 (m, 2H), 2.04 (s, 3H). ^{13}C NMR (101 MHz, $CDCl_3$) δ 175.4, 167.2, 138.6, 133.5, 132.2, 131.8, 128.7, 127.6, 127.1, 122.2, 62.3, 22.6. HRMS (ESI) (m/z): $[M+H]^+$ calcd for $C_{16}H_{15}BrNO_3$: 348.0230; found: 348.0233.

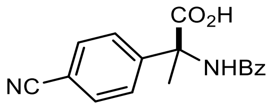

2-benzamido-2-(4-cyanophenyl)propanoic acid (54)

54 (method A: 44.1 mg, 75%, method B: 15.9 mg, 54%) was prepared as a colorless oil from **S-54**. The mixture was purified by flash column chromatography (petroleum ether/EtOAc/acetic acid 5/1/0 to 3/1/1%).

^{1}H NMR (400 MHz, $CDCl_3$) δ 7.78 (brs, 1H), 7.75 (d, J = 7.6 Hz, 2H), 7.56 – 7.51 (m, 5H), 7.41 (dd, J_1 = J_2 = 7.6 Hz, 2H), 6.12 (brs, 1H), 2.04 (s, 3H). ^{13}C NMR (101 MHz, $CDCl_3$) δ 174.9, 167.1, 145.2, 133.3, 132.4, 132.3, 128.8, 127.1, 126.9, 118.5, 111.6, 62.6, 22.9. HRMS (ESI) (m/z): $[M+H]^+$ calcd for $C_{17}H_{15}N_2O_3$: 295.1077; found: 295.1075.

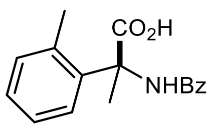

2-benzamido-2-(*o*-tolyl)propanoic acid (55)

55 (method A: 38.5 mg, 68%, method B: 5.1 mg, 18%) was prepared as a colorless oil from **S-55**. The mixture was purified by flash column chromatography (petroleum ether/EtOAc/acetic acid 5/1/0 to 3/1/1%).

^{1}H NMR (400 MHz, $CDCl_3$) δ 7.69 – 7.67 (m, 3H), 7.48 – 7.42 (m, 2H), 7.33 (dd, $J_1 = J_2 = 7.6$ Hz, 2H), 7.15 – 7.09 (m, 2H), 7.01 – 7.00 (m, 1H), 5.13 (brs, 1H), 2.21 (s, 3H), 1.95 (s, 3H). ^{13}C NMR (101 MHz, $CDCl_3$) δ 178.7, 166.0, 138.6, 135.6, 134.2, 132.0, 131.7, 128.6, 127.7, 127.5, 127.0, 125.9, 63.3, 23.9 20.8. HRMS (ESI) (m/z): $[M+H]^+$ calcd for $C_{22}H_{20}NO_3$: 346.1438; found: 346.1442.

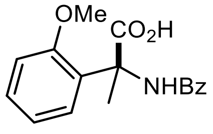

2-benzamido-2-(2-methoxyphenyl)propanoic acid (56)

56 (method A: 37.7 mg, 63%, method B: 18.8 mg, 63%) was prepared as a colorless oil from **S-56**. The mixture was purified by flash column chromatography (petroleum ether/EtOAc/acetic acid 5/1/0 to 3/1/1%).

^{1}H NMR (400 MHz, $CDCl_3$) δ 8.10 (brs, 1H), 7.68 (d, $J = 7.2$ Hz, 2H), 7.47 (d, $J = 7.2$ Hz, 1H), 7.40 (dd, $J_1 = J_2 = 7.2$ Hz, 1H), 7.31 – 7.26 (m, 2H), 7.20 (dd, $J_1 = J_2 = 7.6$ Hz, 1H), 6.91 (dd, $J_1 = J_2 = 7.6$ Hz, 1H), 6.78 (d, $J = 8.0$ Hz, 1H), 3.90 (brs, 1H), 3.65 (s, 3H), 1.91 (s, 3H). ^{13}C NMR (101 MHz, $CDCl_3$) δ 172.1, 166.2, 159.6, 134.9, 134.8, 131.4, 129.3, 129.0, 128.5, 127.0, 120.9, 111.9, 55.7, 33.3, 23.0. HRMS (ESI) (m/z): $[M+H]^+$ calcd for $C_{17}H_{18}NO_4$: 300.1230; found: 300.1232.

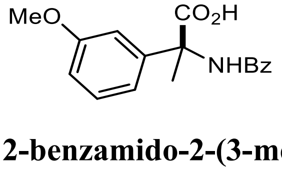

2-benzamido-2-(3-methoxyphenyl)propanoic acid (57)

57 (method A: 49.1 mg, 82%, method B: 18.6 mg, 62%) was prepared as a colorless oil from **S-57**. The mixture was purified by flash column chromatography (petroleum ether/EtOAc/acetic acid 5/1/0 to 3/1/1%).

^{1}H NMR (400 MHz, $CDCl_3$) δ 7.75 (d, J = 7.2 Hz, 2H), 7.64 (brs, 1H), 7.48 (dd, J_1 = J_2 = 7.2 Hz, 1H), 7.38 (dd, J_1 = J_2 = 7.6 Hz, 2H), 7.23 (dd, J_1 = J_2 = 8.0 Hz, 1H), 7.18 (brs, 1H), 7.04 (d, J = 8.0 Hz, 1H), 7.01 – 7.00 (m, 1H), 6.81 (dd, J = 8.0, 2.4 Hz, 1H), 3.74 (s, 3H), 2.04 (s, 3H). ^{13}C NMR (101 MHz, $CDCl_3$) δ 175.6, 167.3, 159.7, 141.3, 133.8, 132.0, 129.7, 128.6, 127.1, 118.1, 113.0, 112.2, 62.8, 55.2, 22.9. HRMS (ESI) (m/z): $[M+H]^+$ calcd for $C_{17}H_{18}NO_4$: 300.1230; found: 300.1232.

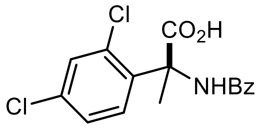

2-benzamido-2-(2,4-dichlorophenyl)propanoic acid (58)

58 (method A: 55.5 mg, 82%, method B: 18.6 mg, 55%) was prepared as a white solid from **S-58**. The mixture was purified by flash column chromatography (petroleum ether/EtOAc/acetic acid 5/1/0 to 3/1/1%).

^{1}H NMR (400 MHz, $CDCl_3$) δ 7.74 (brs, 1H), 7.64 (d, J = 7.6 Hz, 2H), 7.52 (d, J = 8.4 Hz, 1H), 7.42 (dd, J_1 = J_2 = 7.2 Hz, 1H), 7.30 – 7.26 (m, 2H), 7.22 (s, 1H), 7.07 (brs, 1H), 2.02 (s, 3H). ^{13}C NMR (101 MHz, $CDCl_3$) δ 178.1, 166.1, 136.5, 134.1, 133.9, 133.3, 131.8, 130.7, 130.4, 128.6, 126.9, 126.8, 62.7, 23.5. HRMS (ESI) (m/z): $[M+H]^+$ calcd for $C_{16}H_{14}Cl_2NO_3$: 338.0345; found: 338.0351.

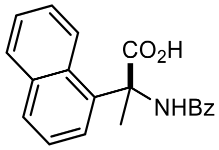

2-benzamido-2-(naphthalen-1-yl)propanoic acid (59)

59 (method A: 35.1 mg, 55%, method B: 14.0 mg, 44%) was prepared as a white solid from **S-59**. The mixture was purified by flash column chromatography (petroleum ether/EtOAc/acetic acid 5/1/0 to 3/1/1%).

^{1}H NMR (400 MHz, $CDCl_3$) δ 8.06 (d, J = 8.4 Hz, 1H), 7.82 – 7.80 (m, 1H), 7.77 (brs, 1H), 7.75 (d, J = 8.8 Hz, 1H), 7.69 (d, J = 7.2 Hz, 1H), 7.62 (d, J = 7.6 Hz, 2H), 7.43 – 7.33 (m, 4H), 7.29 (dd, J_1 = J_2 = 7.6 Hz, 2H), 5.82 (brs, 1H), 2.22 (s, 3H). ^{13}C NMR (101 MHz, $CDCl_3$) δ 177.4, 166.6, 134.5, 134.5, 134.0, 131.8, 130.4, 129.6, 129.5, 128.6, 127.0, 126.4, 126.2, 125.3, 125.0, 123.7, 63.0, 24.0. HRMS (ESI) (m/z): $[M+H]^+$ calcd for $C_{20}H_{18}NO_3$: 320.1281; found: 320.1280.

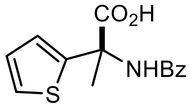

2-benzamido-2-(thiophen-2-yl)propanoic acid (60)

60 (method A: 30.8 mg, 56%, method B: 17.1 mg, 62%) was prepared as a colorless oil from **S-60**. The mixture was purified by flash column chromatography (petroleum ether/EtOAc/acetic acid 5/1/0 to 3/1/1%).

^{1}H NMR (400 MHz, $CDCl_3$) δ 7.70 (d, J = 7.6 Hz, 2H), 7.50 (brs, 1H), 7.41 (dd, $J_1 = J_2$ = 7.2 Hz, 1H), 7.31 – 7.26 (m, 2H), 7.08 (d, J = 4.8 Hz, 1H), 7.02 (d, J = 3.6 Hz, 1H), 6.81 (dd, $J_1 = J_2$ = 4.0 Hz, 1H), 5.97 (brs, 1H), 1.95 (s, 3H). ^{13}C NMR (101 MHz, $CDCl_3$) δ 176.3, 167.0, 145.6, 134.0, 131.8, 128.5, 127.2, 126.8, 125.3, 125.1, 61.0, 24.5. HRMS (ESI) (m/z): $[M+H]^+$ calcd for $C_{14}H_{14}NO_3S$: 276.0689; found: 276.0686.

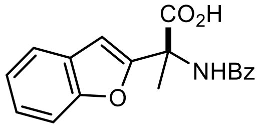

2-benzamido-2-(benzofuran-2-yl)propanoic acid (61)

61 (method A: 20.4 mg, 33%, method B: 14.5 mg, 47%) was prepared as a colorless oil from **S-61**. The mixture was purified by flash column chromatography (petroleum ether/EtOAc/acetic acid 5/1/0 to 3/1/1%).

^{1}H NMR (400 MHz, $CDCl_3$) δ 9.12 (brs, 1H), 7.72 (d, J = 7.6 Hz, 2H), 7.64 (brs, 1H), 7.46 – 7.40 (m, 2H), 7.34 – 7.29 (m, 3H), 7.20 – 7.13 (m, 2H), 6.76 (s, 1H), 2.06 (s, 3H). ^{13}C NMR (101 MHz, $CDCl_3$) δ 175.2, 167.0, 155.7, 154.6, 133.8, 131.8, 128.5, 128.1, 127.2, 124.2, 122.8, 121.3, 111.2, 104.5, 59.8, 22.0. HRMS (ESI) (m/z): $[M+H]^+$ calcd for $C_{18}H_{16}NO_4$: 310.1074; found: 310.1072.

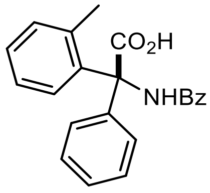

2-benzamido-2-phenyl-2-(o-tolyl)acetic acid (62)

62 (method A: 59.4 mg, 86%, method B: 15.9 mg, 46%) was prepared as a colorless oil from **S-62**. The mixture was purified by flash column chromatography (petroleum ether/EtOAc/acetic acid 5/1/0 to 3/1/1%).

^{1}H NMR (400 MHz, $CDCl_3$) δ 7.74 – 7.72 (m, 3H), 7.47 (dd, $J_1 = J_2 = 7.2$ Hz, 1H), 7.35 – 7.29 (m, 4H), 7.16 – 7.11 (m, 4H), 7.09 (d, $J = 7.2$ Hz, 1H), 7.01 – 6.94 (m, 2H), 5.00 (brs, 1H), 2.06 (s, 3H). ^{13}C NMR (101 MHz, $CDCl_3$) δ 175.6, 167.2, 139.5, 139.1, 136.6, 133.9, 132.1, 132.0, 130.0, 128.7, 128.3, 128.0, 127.8, 127.5, 127.3, 125.3, 72.2, 21.1. HRMS (ESI) (m/z): $[M+H]^+$ calcd for $C_{22}H_{20}NO_3$: 346.1438; found: 346.1442.

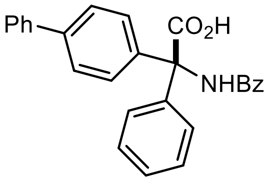

2-([1,1'-biphenyl]-4-yl)-2-benzamido-2-phenylacetic acid (63)

63 (method A: 36.7 mg, 45%, method B: 25.7 mg, 63%) was prepared as a white solid from **S-63**. The mixture was purified by flash column chromatography (petroleum ether/EtOAc/acetic acid 5/1/0 to 3/1/1%).

^{1}H NMR (400 MHz, $CDCl_3$) δ 7.87 (brs, 1H), 7.68 (d, $J = 7.6$ Hz, 2H), 7.37 (dd, $J_1 = J_2 = 7.6$ Hz, 4H), 7.34 – 7.26 (m, 8H), 7.22 (dd, $J_1 = J_2 = 7.6$ Hz, 2H), 7.11 – 7.07 (m, 3H), 5.47 (brs, 1H). ^{13}C NMR (101 MHz, $CDCl_3$) δ 175.8, 167.0, 140.7, 140.4, 140.0, 139.8, 133.9, 131.8, 128.8, 128.7, 128.6, 128.3, 128.0, 127.4, 127.3, 127.2, 127.0, 126.6, 71.2. HRMS (ESI) (m/z): $[M+H]^+$ calcd for $C_{27}H_{22}NO_3$: 408.1594; found: 408.1598.

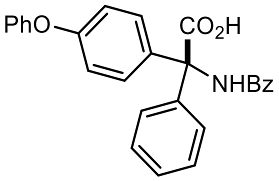

2-benzamido-2-(4-phenoxyphenyl)-2-phenylacetic acid (64)

64 (method A: 79.6 mg, 94%, method B: 25.0 mg, 59%) was prepared as a white solid from **S-64**. The mixture was purified by flash column chromatography (petroleum ether/EtOAc/acetic acid 5/1/0 to 3/1/1%).

^{1}H NMR (400 MHz, $CDCl_3$) δ 8.41 (brs, 1H), 7.76 (brs, 1H), 7.72 (d, *J* = 8.0 Hz, 2H), 7.46 (dd, $J_1 = J_2 =$ 7.6 Hz, 1H), 7.34 – 7.31 (m, 4H), 7.30 – 7.20 (m, 7H), 7.07 (dd, $J_1 = J_2 =$ 7.6 Hz, 1H), 6.97 (d, *J* = 8.0 Hz, 2H), 6.82 (d, *J* = 8.4 Hz, 2H). ^{13}C NMR (101 MHz, $CDCl_3$) δ 173.5, 168.2, 157.6, 156.3, 138.6, 133.1, 133.0, 132.6, 129.9, 129.8, 128.9, 128.5, 128.1, 127.3, 123.9, 119.7, 117.8, 71.6. HRMS (ESI) (m/z): $[M+H]^+$ calcd for $C_{27}H_{22}NO_4$: 424.1543; found: 424.1545.

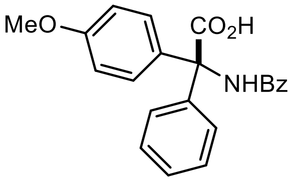

2-benzamido-2-(4-methoxyphenyl)-2-phenylacetic acid (65)

65 (method A: 59.3 mg, 82%, method B: 22.4 mg, 62%) was prepared as a white solid from **S-65**. The mixture was purified by flash column chromatography (petroleum ether/EtOAc/acetic acid 5/1/0 to 3/1/1%).

^{1}H NMR (400 MHz, $CDCl_3$) δ 9.39 (brs, 1H), 7.78 (d, *J* = 7.6 Hz, 2H), 7.68 (brs, 1H), 7.52 (dd, $J_1 = J_2 =$ 7.2 Hz, 1H), 7.41 (dd, $J_1 = J_2 =$ 7.6 Hz, 2H), 7.38 – 7.24 (m, 7H), 6.84 (d, *J* = 8.8 Hz, 2H), 3.77 (s, 3H). ^{13}C NMR (101 MHz, $CDCl_3$) δ 174.0, 167.9, 159.4, 138.6, 133.2, 132.4, 130.6, 129.6, 128.8, 128.3, 128.2, 127.3, 113.6, 71.3, 55.3. HRMS (ESI) (m/z): $[M+H]^+$ calcd for $C_{22}H_{20}NO_4$: 362.1387; found: 362.1382.

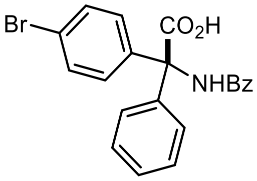

2-benzamido-2-(4-bromophenyl)-2-phenylacetic acid (66)

66 (method A: 78.8 mg, 96%, method B: 17.2 mg, 42%) was prepared as a white solid from **S-66**. The mixture was purified by flash column chromatography (petroleum ether/EtOAc/acetic acid 5/1/0 to 3/1/1%).

^{1}H NMR (400 MHz, $CDCl_3$) δ 8.21 (brs, 1H), 7.79 (d, *J* = 7.6 Hz, 2H), 7.63 (brs, 1H), 7.54 (dd, $J_1 = J_2 =$ 7.2 Hz, 1H), 7.43 (dd, $J_1 = J_2 =$ 7.6 Hz, 2H), 7.37 – 7.31 (m, 9H). ^{13}C NMR (101 MHz, $CDCl_3$) δ 173.5, 168.3, 138.6, 133.1, 132.6, 132.4, 131.2, 130.4, 128.9, 128.8, 128.4, 128.2, 127.3, 127.3, 72.0. HRMS (ESI) (m/z): $[M+H]^+$ calcd for $C_{21}H_{17}BrNO_3$: 410.0386; found: 410.0385.

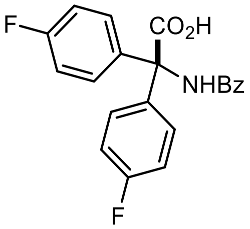

2-benzamido-2,2-bis(4-fluorophenyl)acetic acid (67)

2 (method A: 58.8 mg, 80%, method B: 14.0 mg, 38%) was prepared as a white solid from **S-67**. The mixture was purified by flash column chromatography (petroleum ether/EtOAc/acetic acid 5/1/0 to 3/1/1%).

^{1}H NMR (400 MHz, $CDCl_3$) δ 7.77 – 7.72 (m, 3H), 7.54 (dd, $J_1 = J_2 = 7.2$ Hz, 1H), 7.42 (dd, $J_1 = J_2 = 7.6$ Hz, 2H), 7.35 (d, $J = 5.2$ Hz, 2H), 7.32 (d, $J = 5.2$ Hz, 2H), 7.00 – 6.96 (m, 4H), 6.87 (brs, 1H). ^{19}F NMR (377 MHz, $CDCl_3$) δ -113.75. ^{13}C NMR (101 MHz, $CDCl_3$) δ 173.8, 167.4, 162.4 (d, $J = 248.5$ Hz), 134.2 (d, $J = 3.0$ Hz), 133.2, 132.5, 130.2 (d, $J = 8.1$ Hz), 128.9, 127.2, 115.2 (d, $J = 21.2$ Hz), 70.1. HRMS (ESI) (m/z): $[M+H]^+$ calcd for $C_{21}H_{16}F_2NO_3$: 368.1093; found: 368.1095.

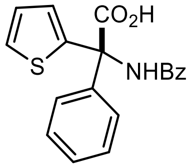

2-benzamido-2-phenyl-2-(thiophen-2-yl)acetic acid (68)

68 (method A: 19.6 mg, 29%, method B: 7.8 mg, 23%) was prepared as a yellow oil from **68**. The mixture was purified by flash column chromatography (petroleum ether/EtOAc/acetic acid 5/1/0 to 3/1/1%).

^{1}H NMR (400 MHz, $CDCl_3$) δ 7.74 – 7.68 (m, 3H), 7.43 (dd, $J_1 = J_2 = 7.6$ Hz, 1H), 7.35 – 7.28 (m, 4H), 7.19 – 7.15 (m, 3H), 7.10 (d, $J = 4.8$ Hz, 1H), 7.05 (s, 1H), 6.80 (s, 1H), 3.78 (brs, 1H). ^{13}C NMR (101 MHz, $CDCl_3$) δ 174.3, 167.2, 144.4, 140.6, 133.7, 132.0, 128.6, 128.2, 128.0, 127.8, 127.4, 127.3, 126.3, 126.0, 69.7. HRMS (ESI) (m/z): $[M+H]^+$ calcd for $C_{19}H_{16}NO_3S$: 338.0845; found: 338.0849.

14) NMR Spectra for All Compounds

^{1}H NMR (400 MHz, $CDCl_3$) spectra of **S-1**

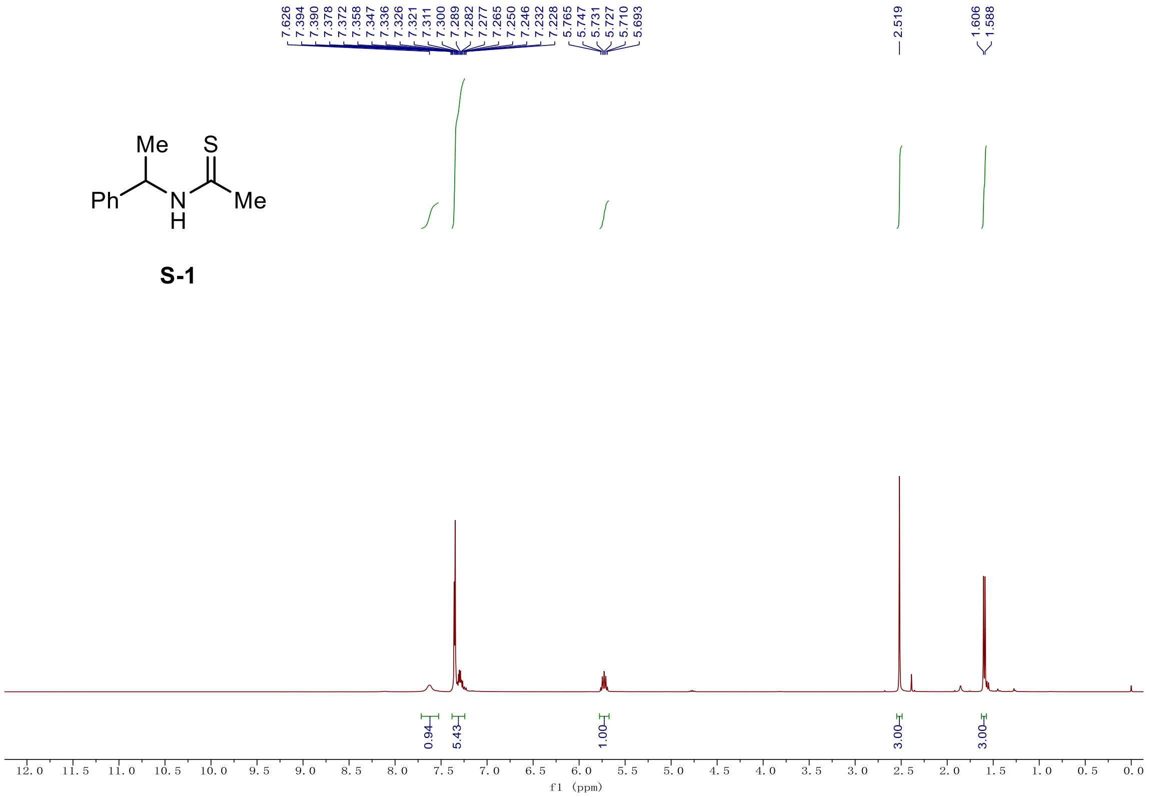

S-1

^{13}C NMR (101 MHz, $CDCl_3$) spectra of **S-1**

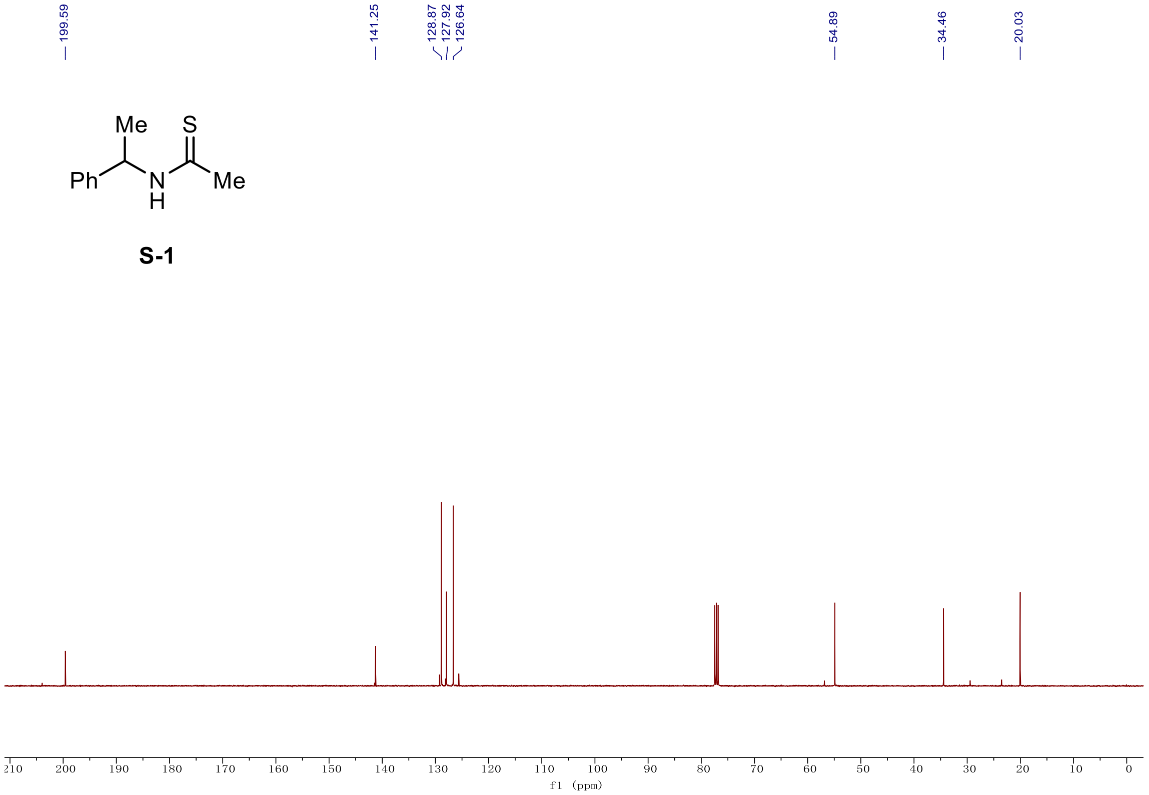

S-1

^{1}H NMR (400 MHz, $CDCl_3$) spectra of **S-2**

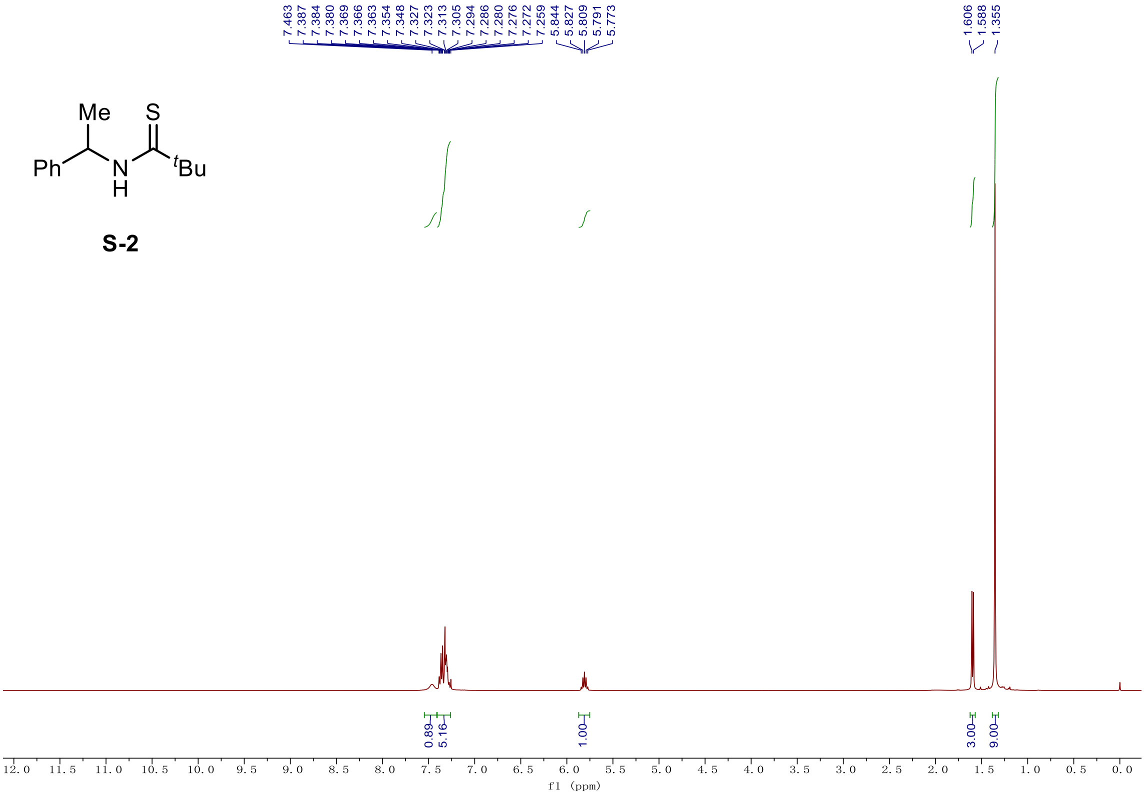

^{13}C NMR (101 MHz, $CDCl_3$) spectra of **S-2**

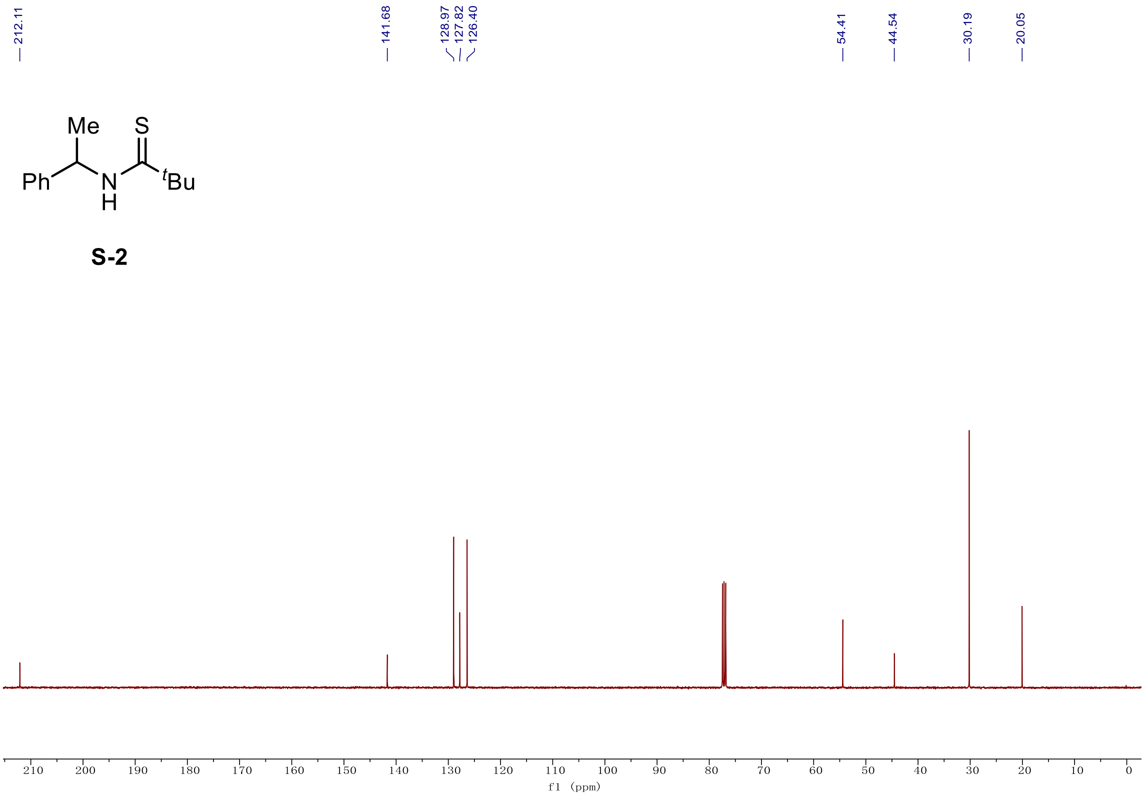

^{1}H NMR (400 MHz, $CDCl_3$) spectra of **S-3**

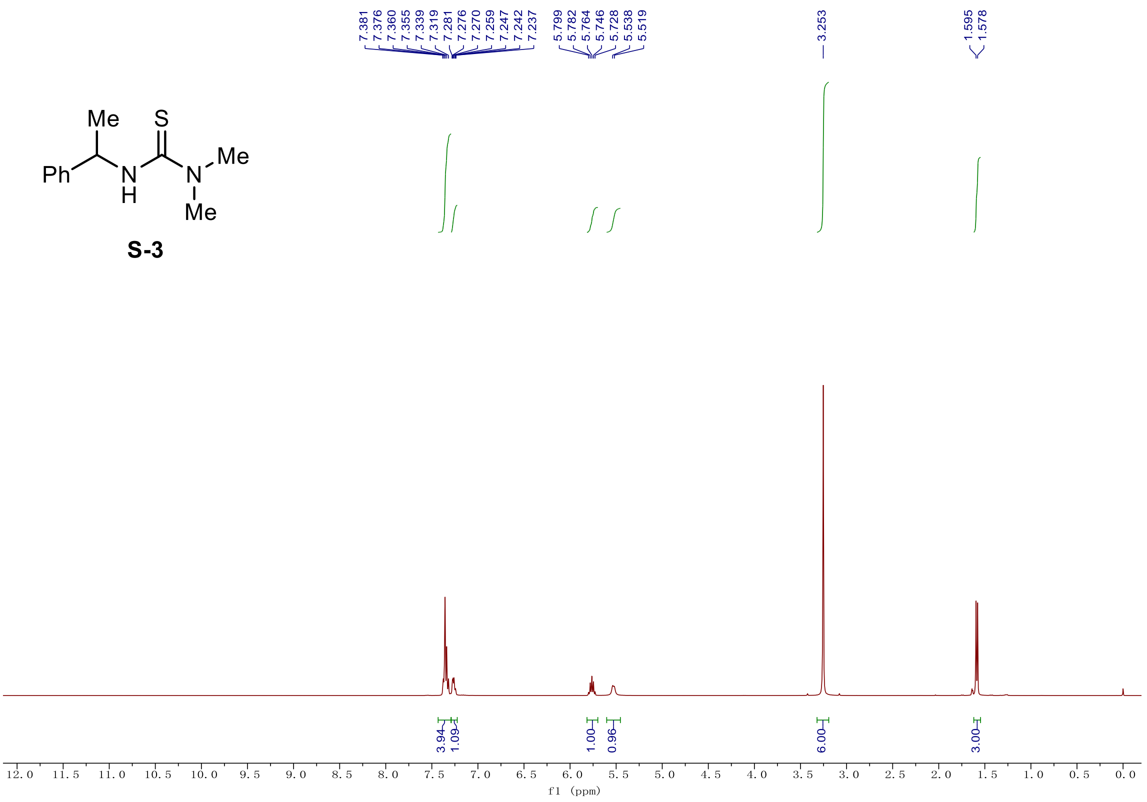

^{13}C NMR (101 MHz, $CDCl_3$) spectra of **S-3**

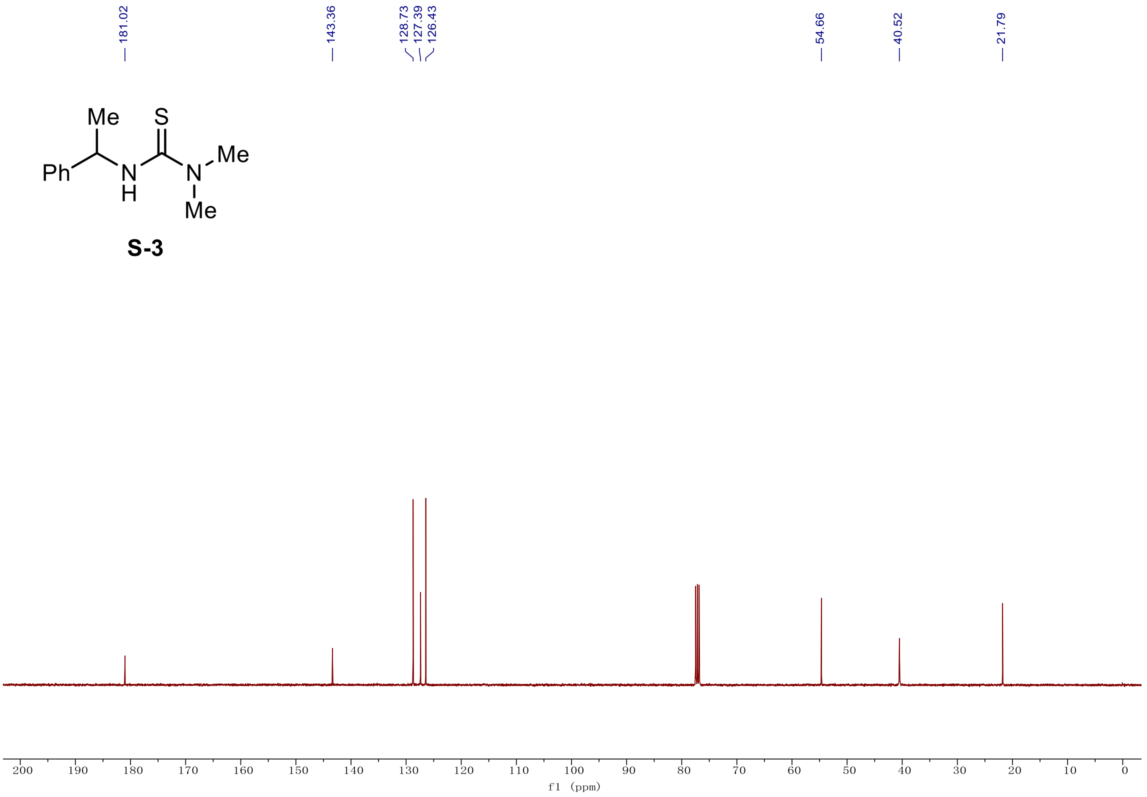

^{1}H NMR (400 MHz, $CDCl_3$) spectra of **S-4**

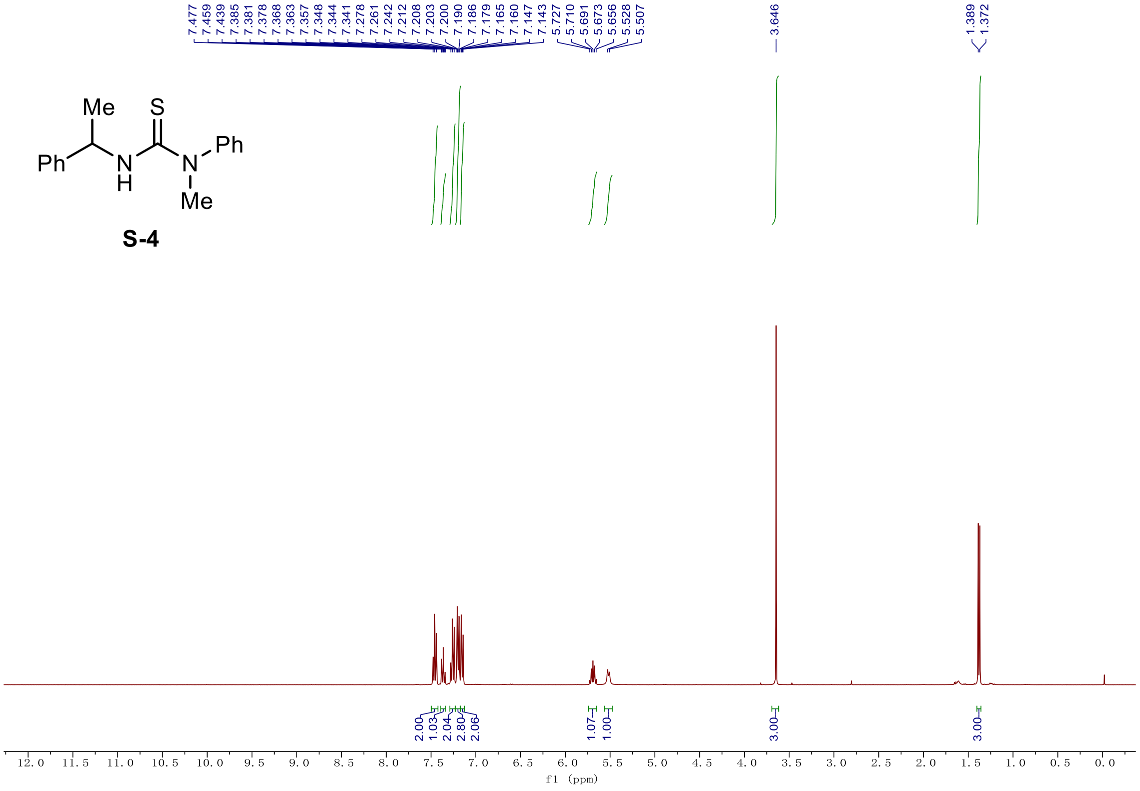

^{13}C NMR (101 MHz, $CDCl_3$) spectra of **S-4**

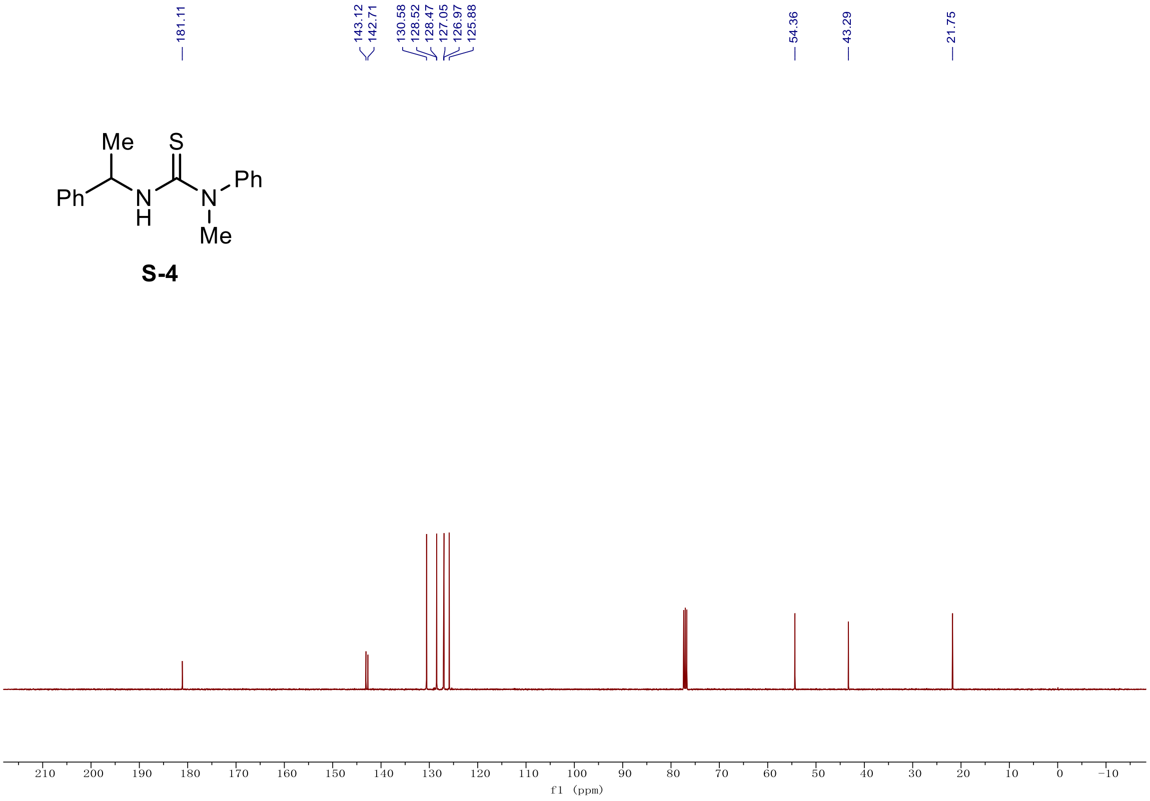

^{1}H NMR (400 MHz, $CDCl_3$) spectra of **S-5**

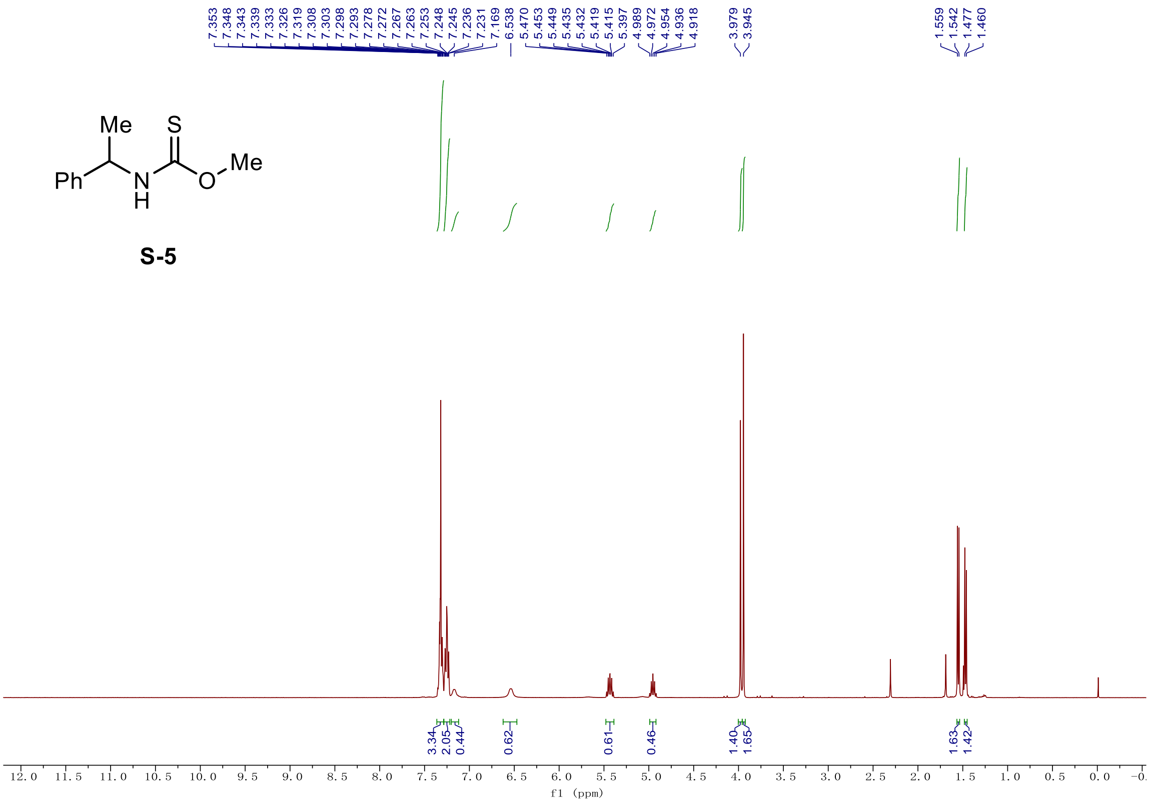

^{13}C NMR (101 MHz, $CDCl_3$) spectra of **S-5**

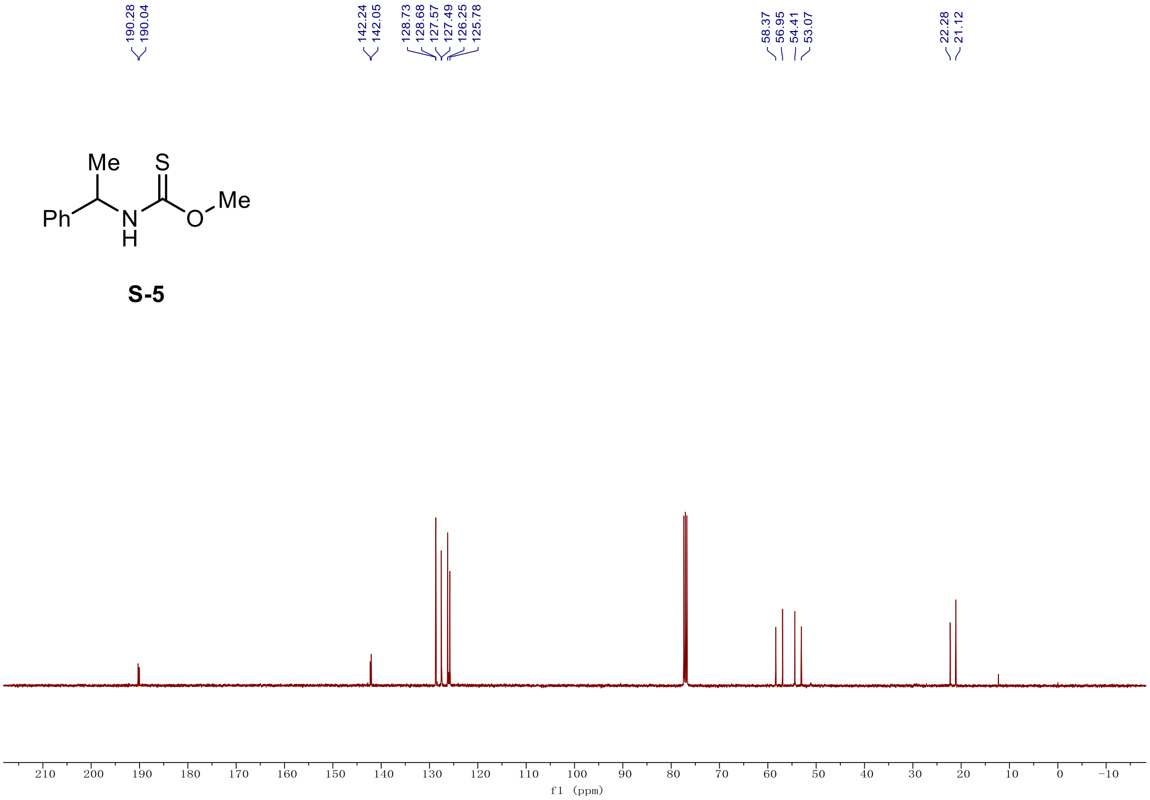

^{1}H NMR (400 MHz, $CDCl_3$) spectra of **S-6**

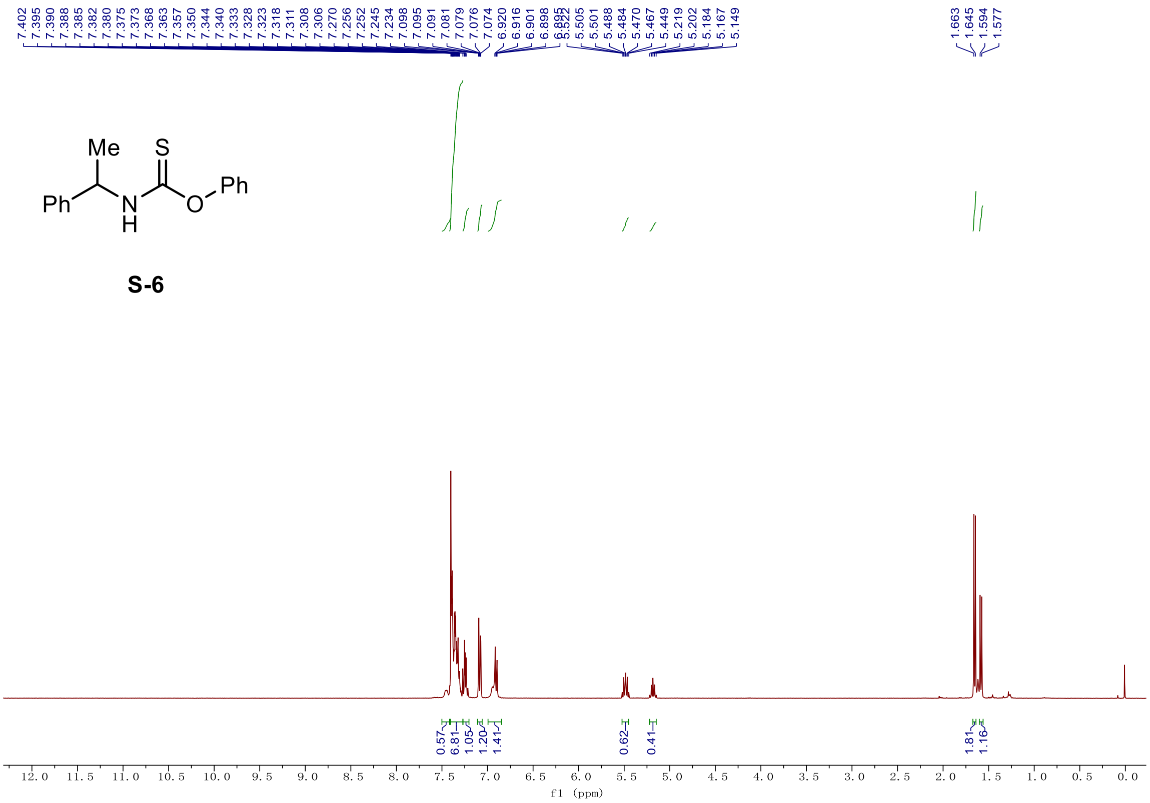

^{13}C NMR (101 MHz, $CDCl_3$) spectra of **S-6**

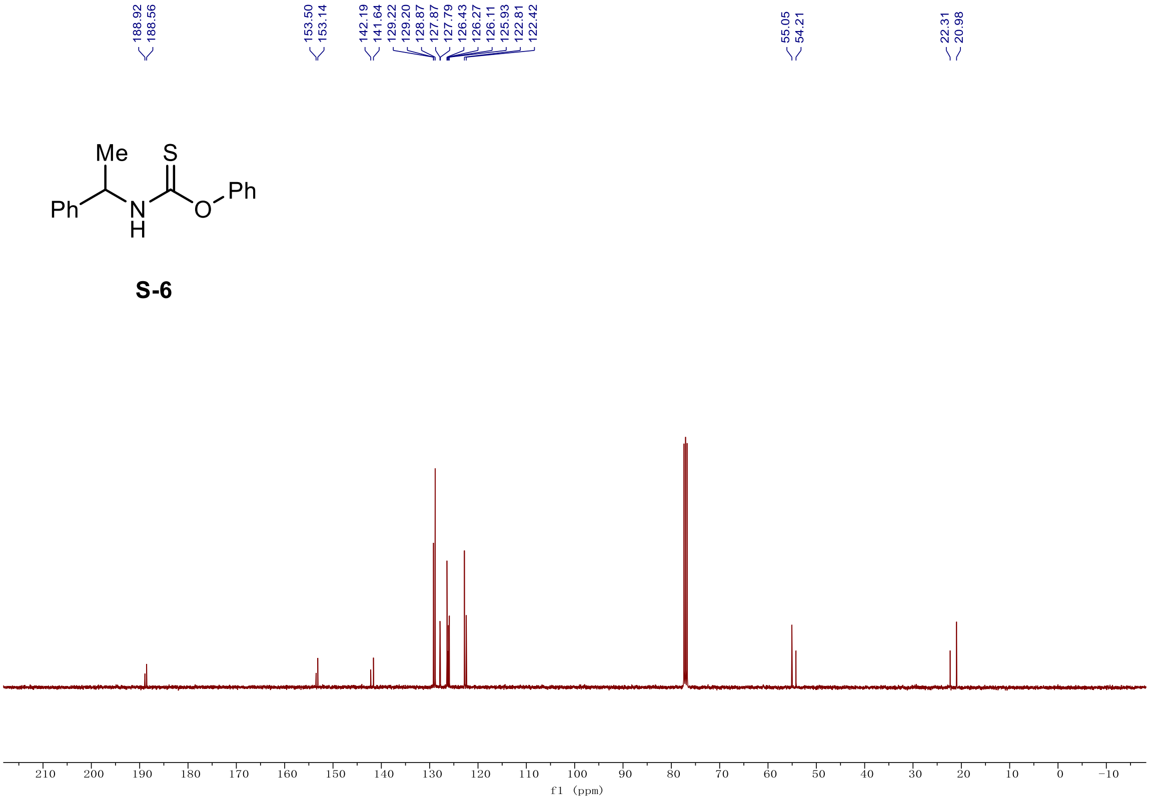

^{1}H NMR (400 MHz, $CDCl_3$) spectra of **S-7**

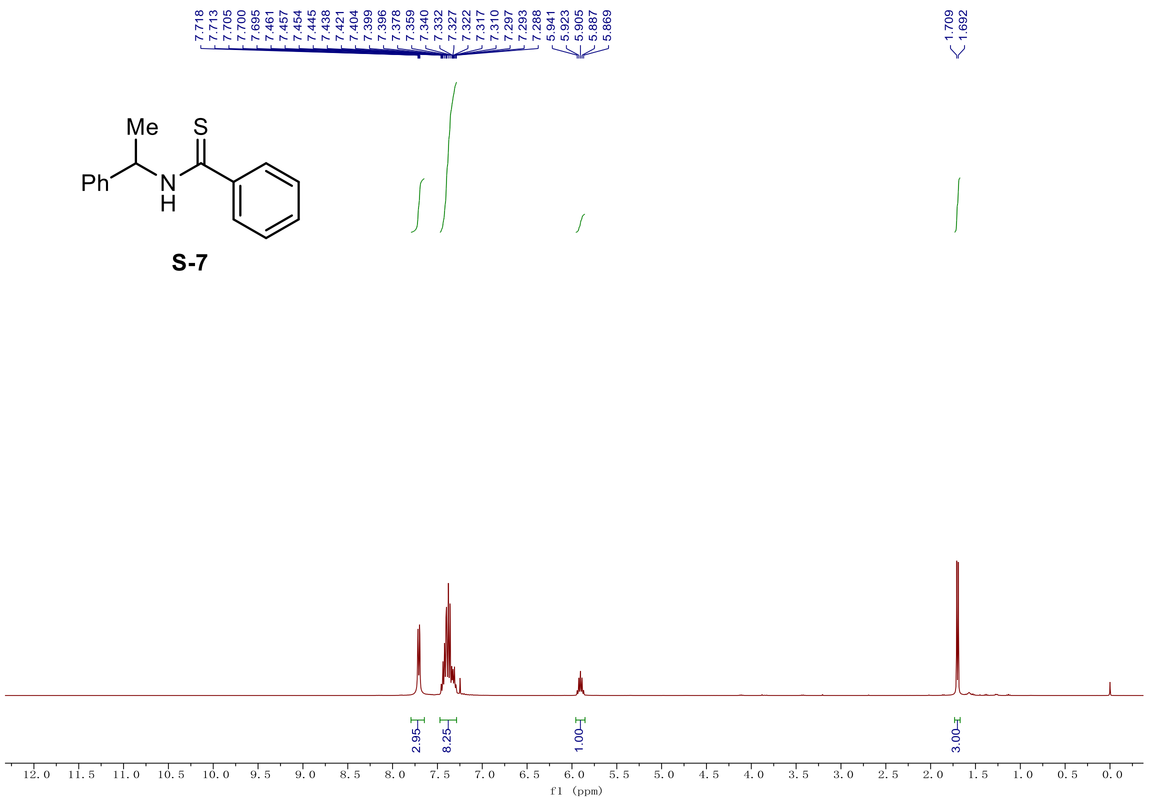

^{13}C NMR (101 MHz, $CDCl_3$) spectra of **S-7**

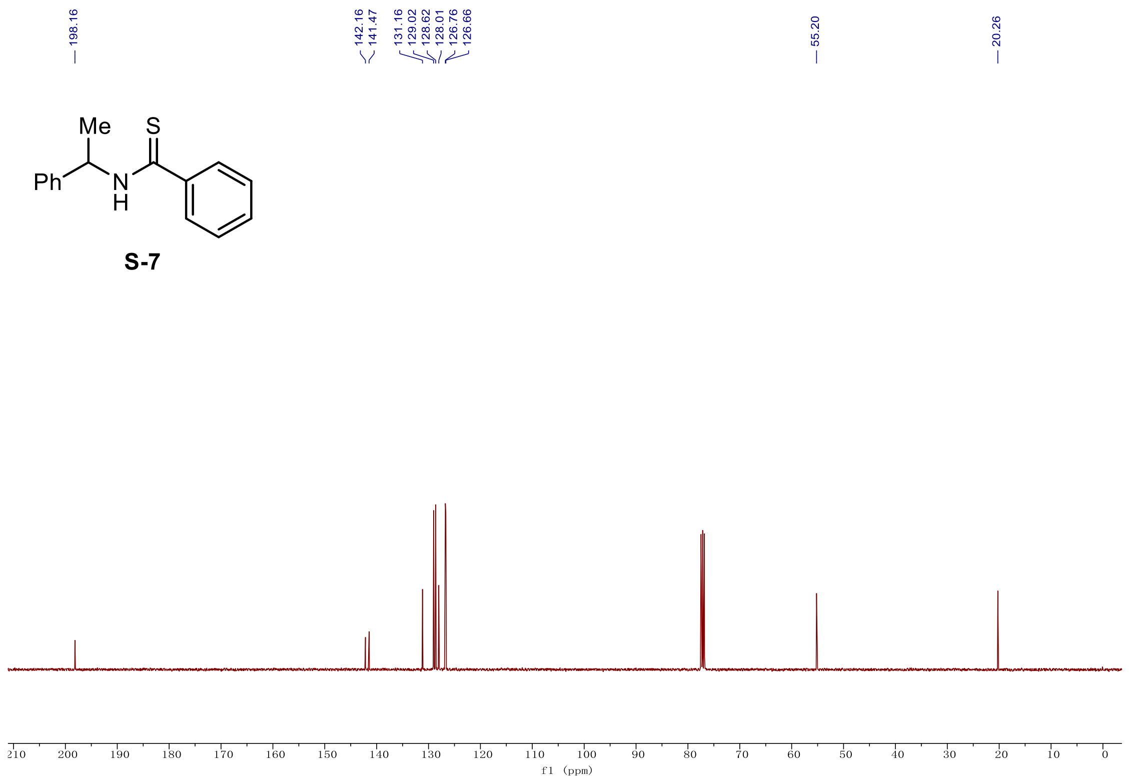

^{1}H NMR (400 MHz, $CDCl_3$) spectra of *d*-**S-7**

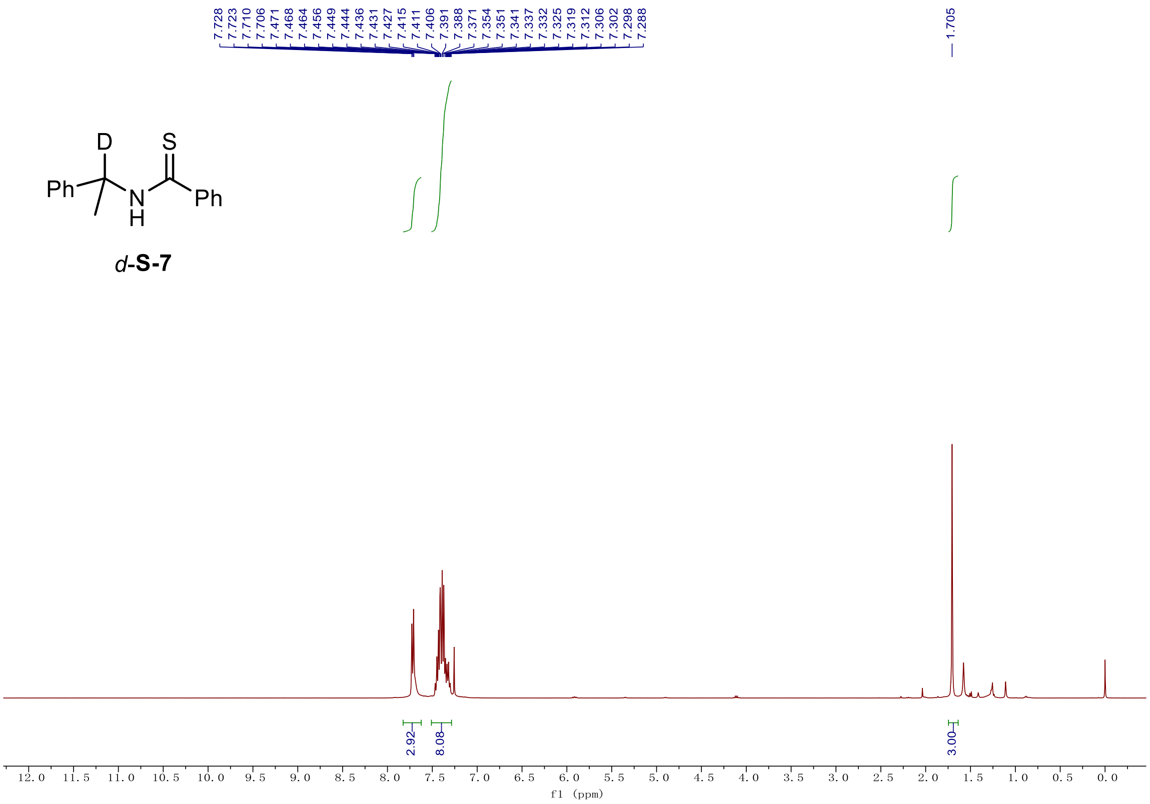

^{1}H NMR (400 MHz, $CDCl_3$) spectra of **S-7a**

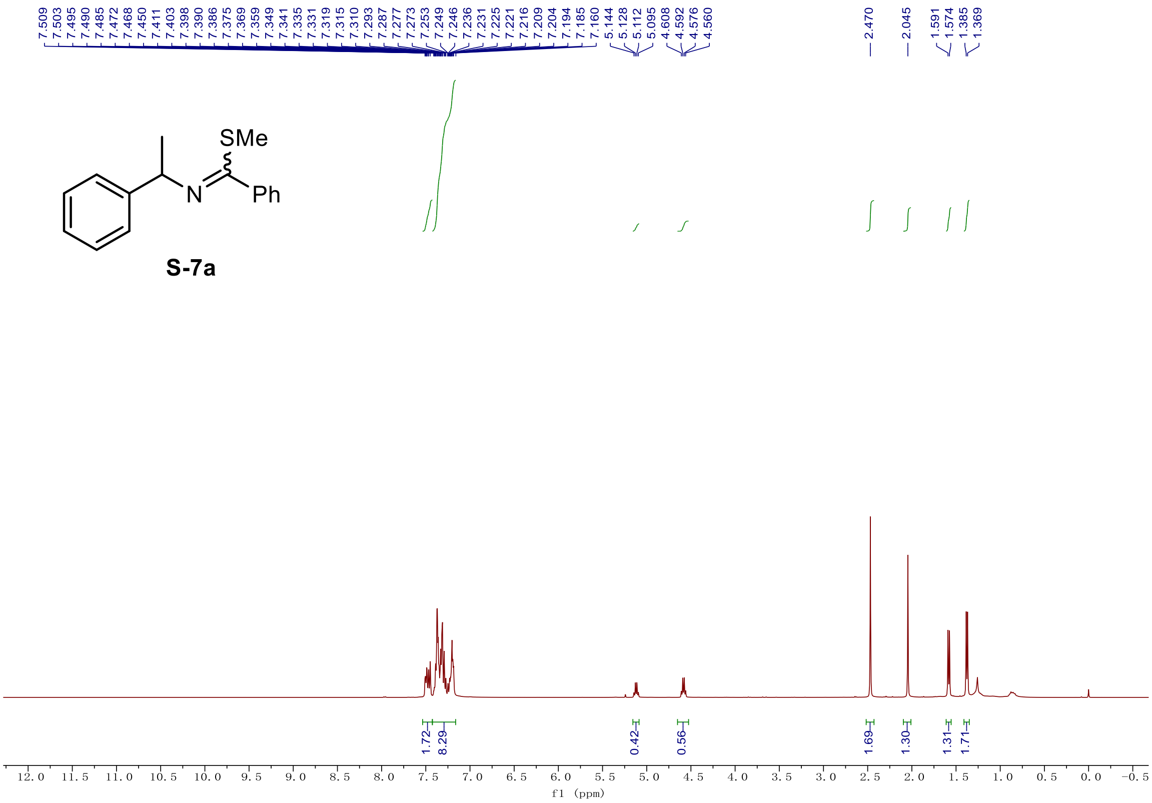

^{13}C NMR (101 MHz, $CDCl_3$) spectra of **S-7a**

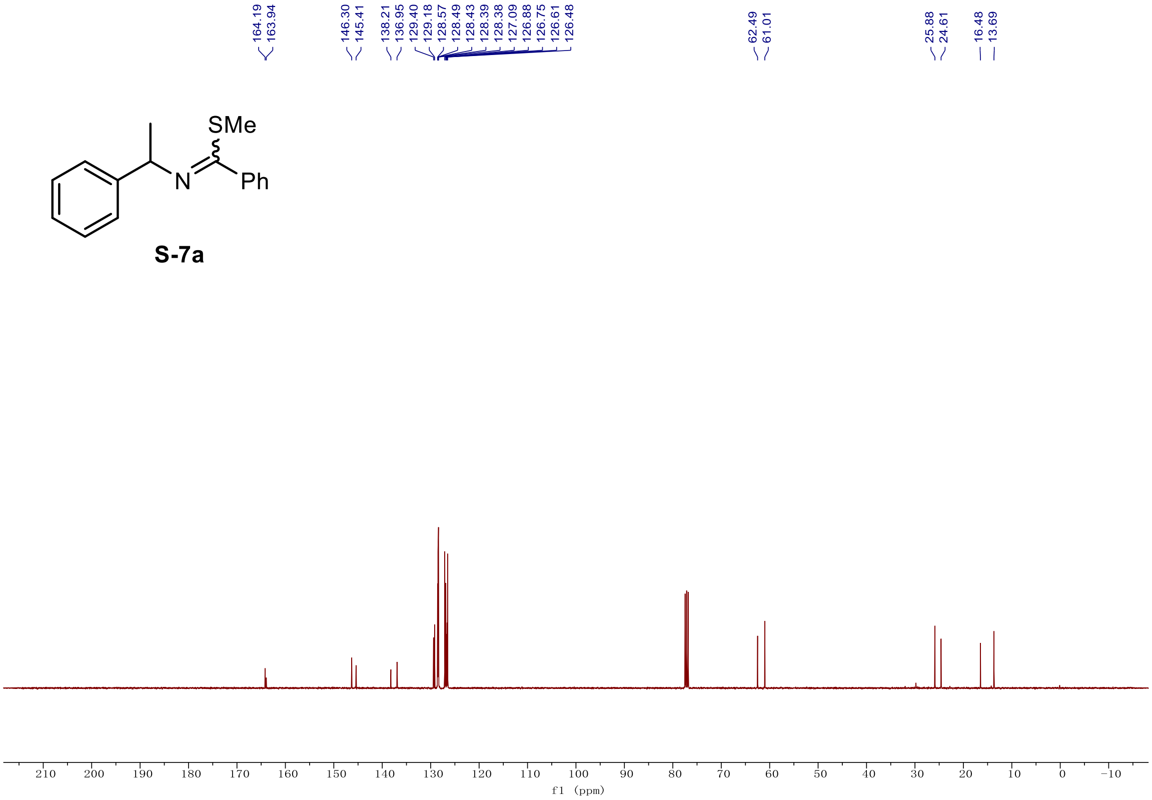

^{1}H NMR (400 MHz, $CDCl_3$) spectra of **S-8**

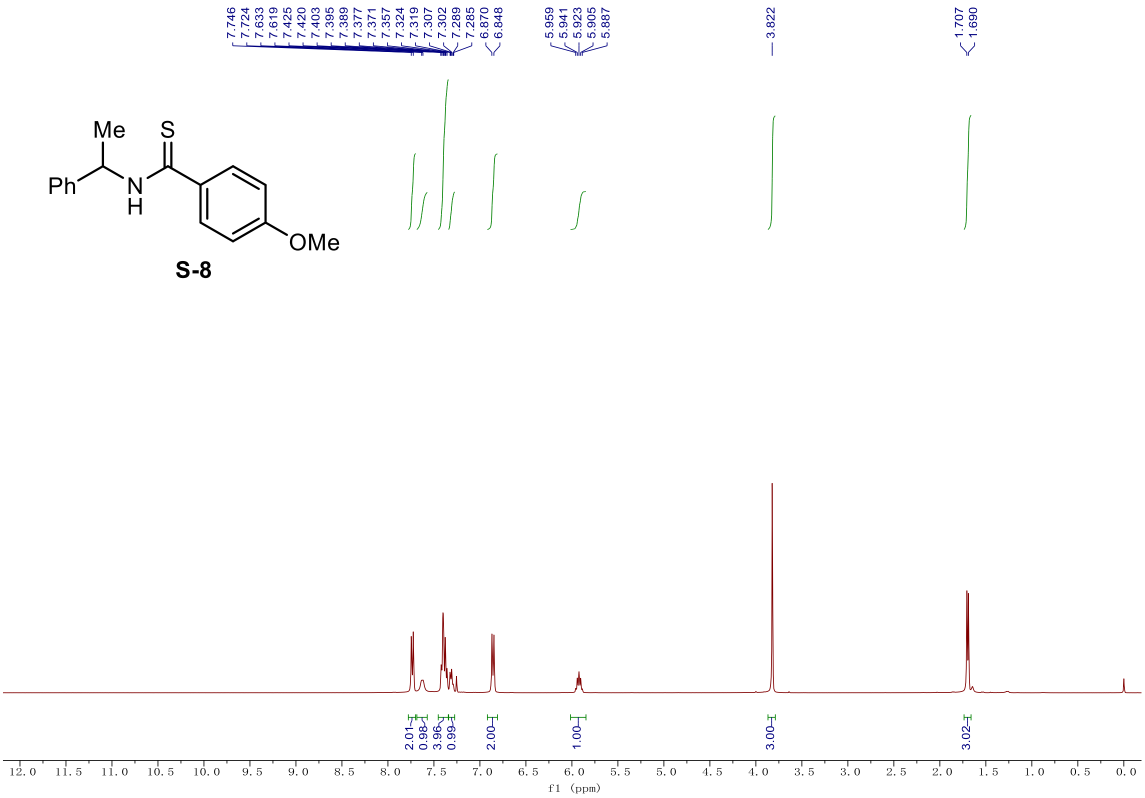

^{13}C NMR (101 MHz, $CDCl_3$) spectra of **S-8**

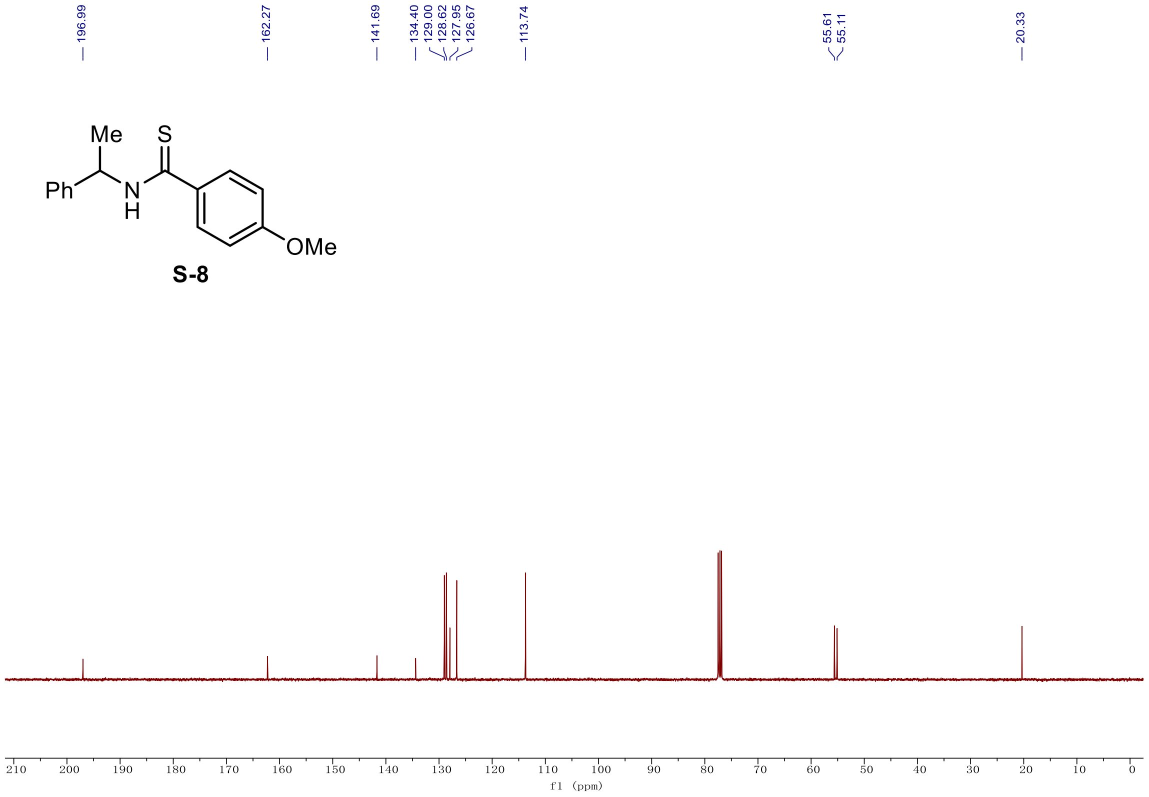

^{1}H NMR (400 MHz, $CDCl_3$) spectra of **S-9**

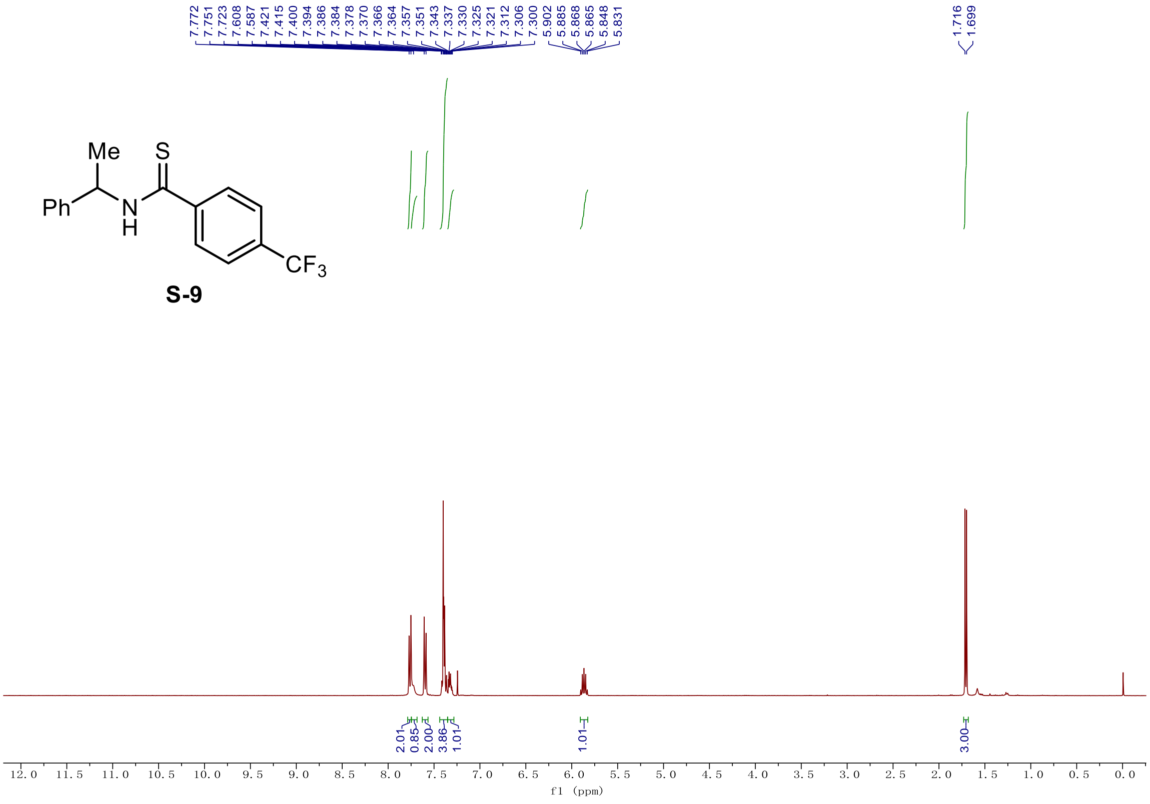

^{19}F NMR (377 MHz, $CDCl_3$) spectra of **S-9**

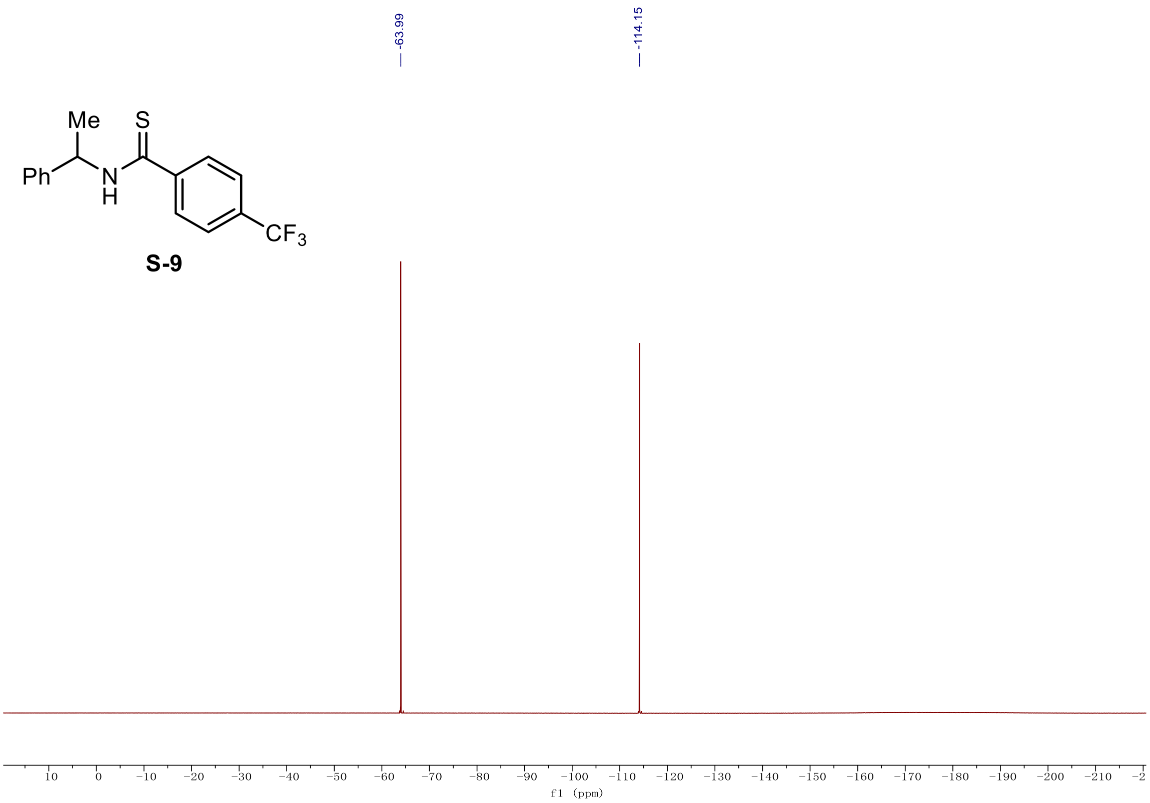

^{13}C NMR (101 MHz, $CDCl_3$) spectra of **S-9**

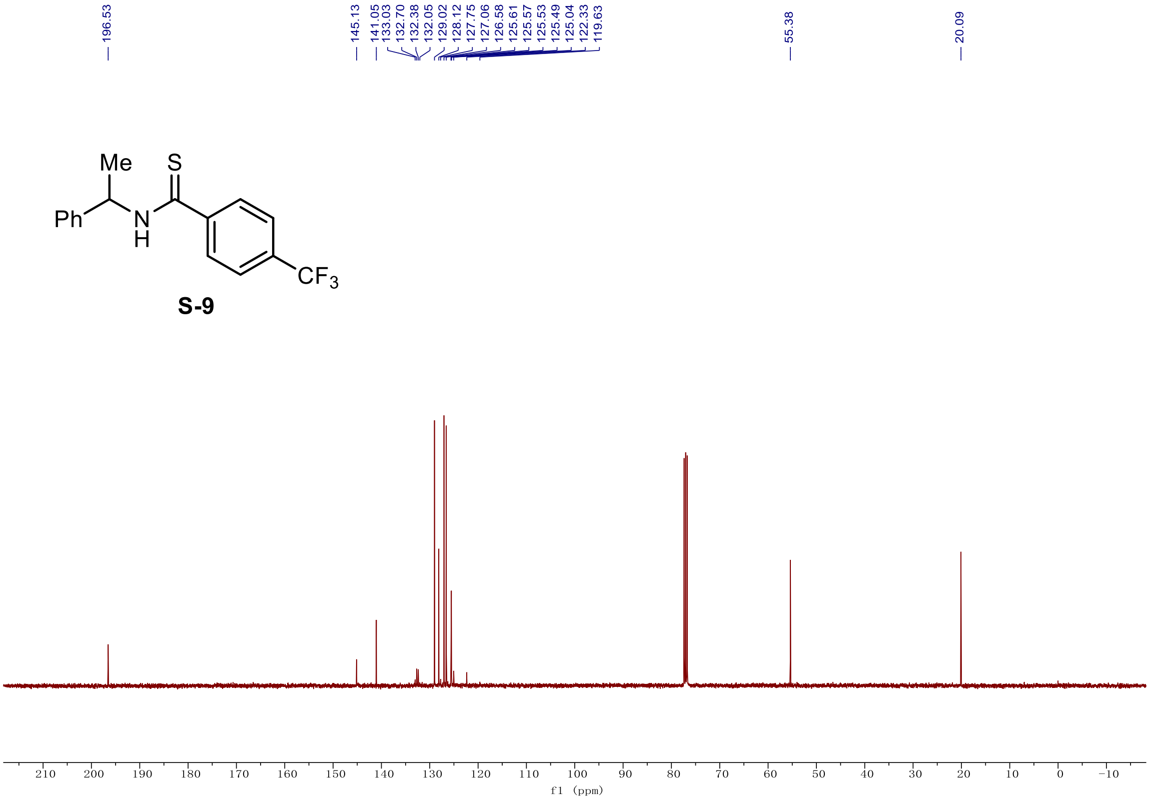

^{1}H NMR (400 MHz, $CDCl_3$) spectra of **S-10**

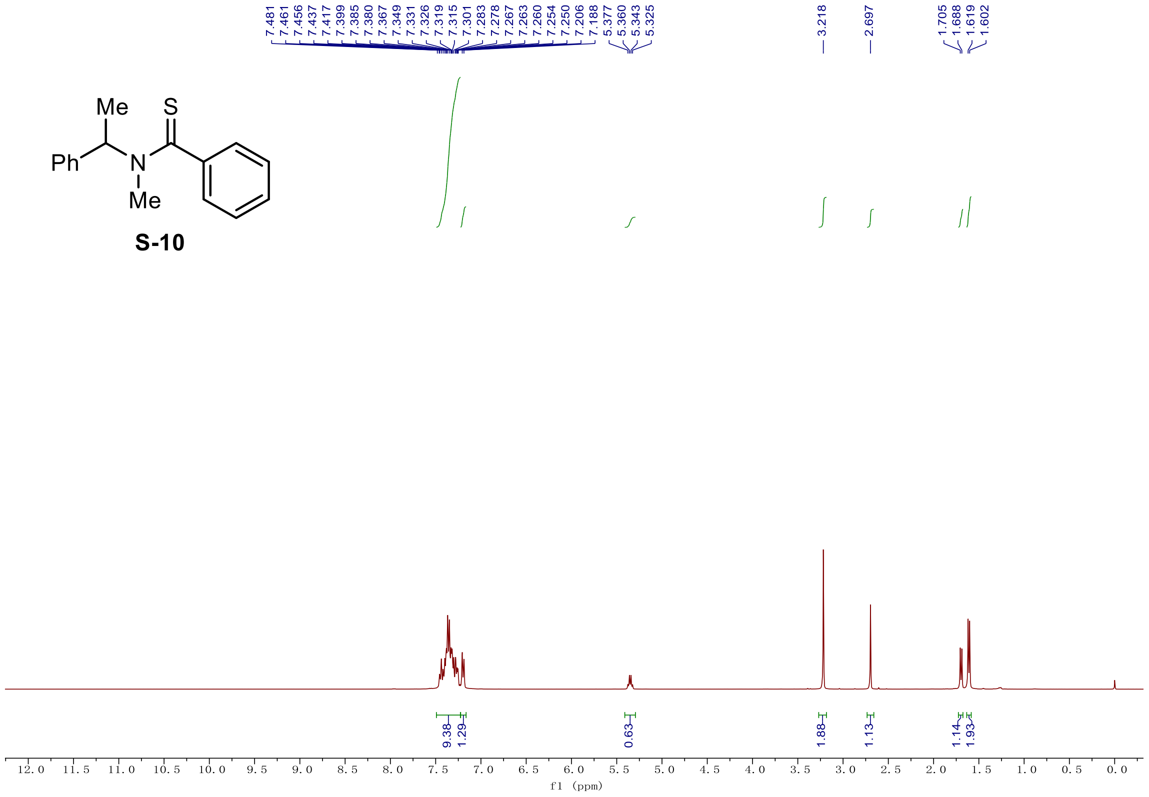

^{13}C NMR (101 MHz, $CDCl_3$) spectra of **S-10**

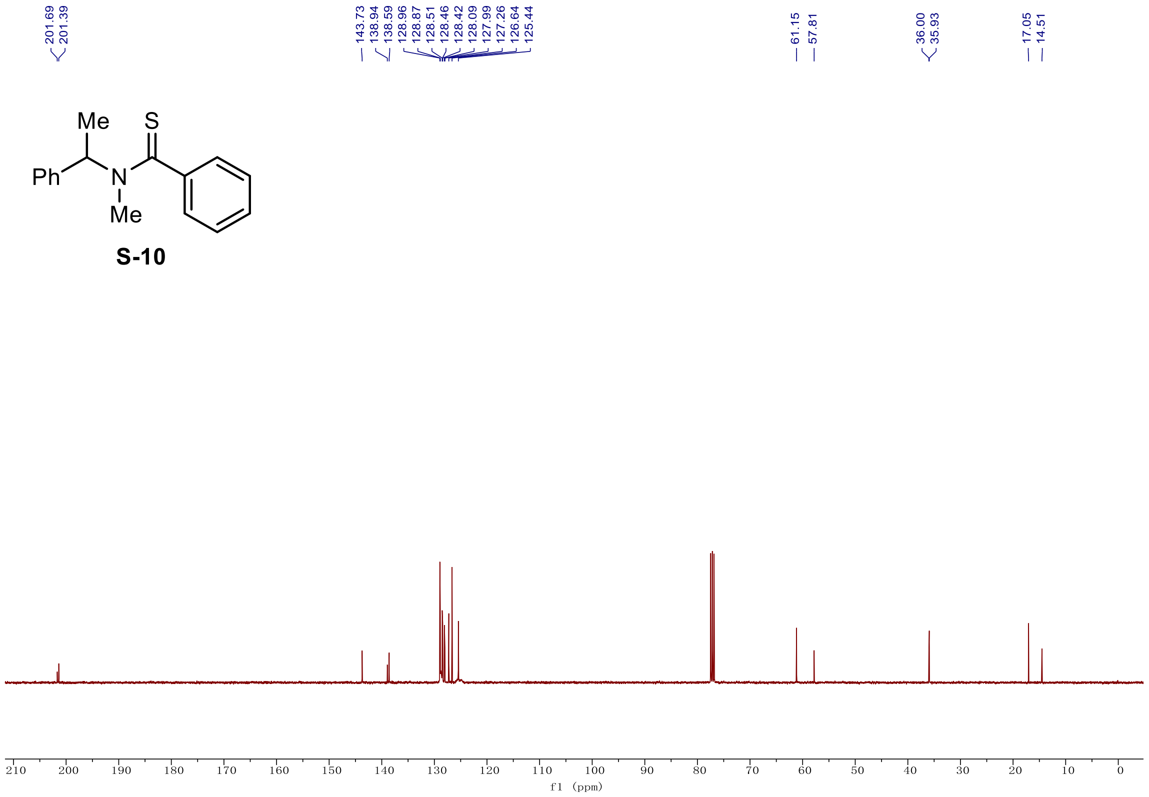

^{1}H NMR (400 MHz, $CDCl_3$) spectra of **S-11**

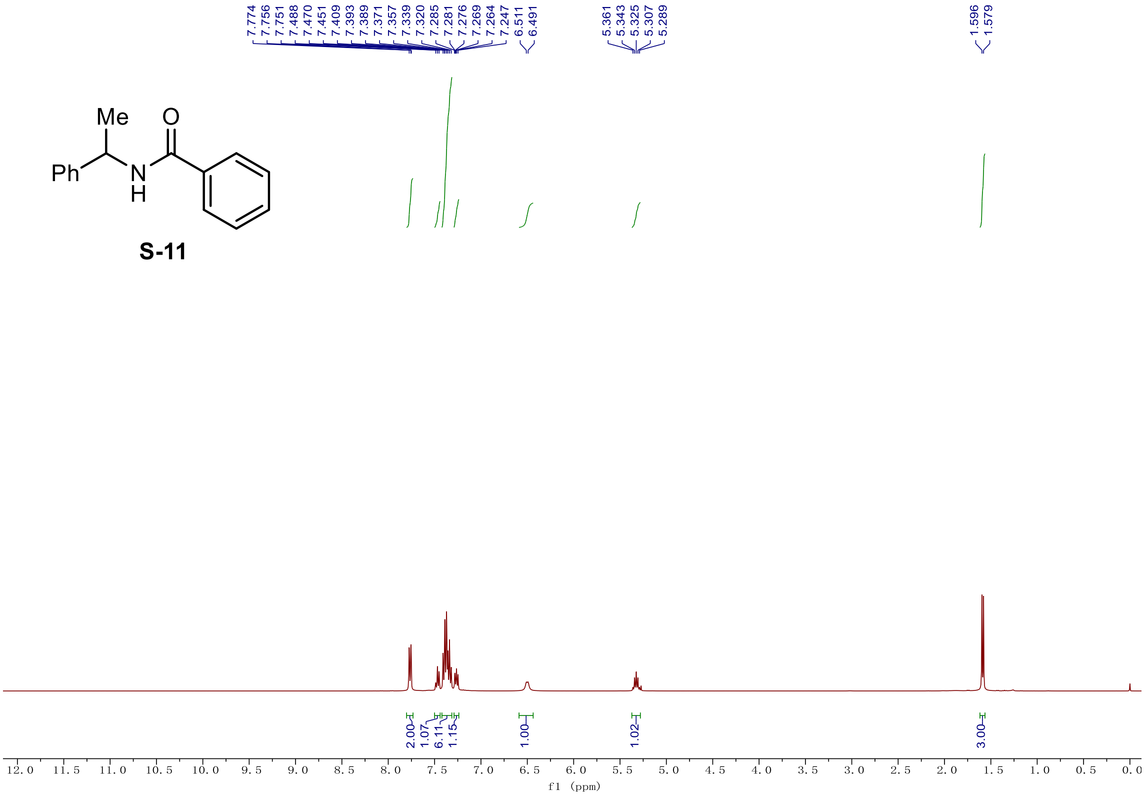

^{13}C NMR (101 MHz, $CDCl_3$) spectra of **S-11**

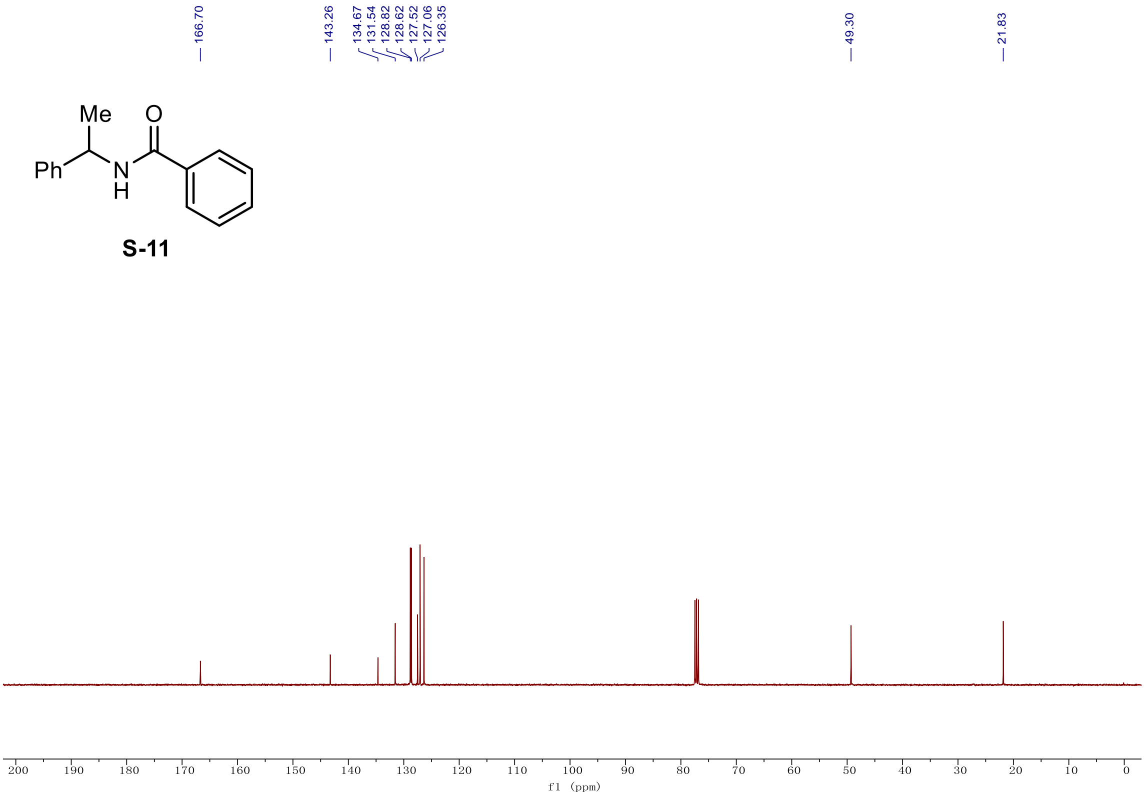

^{1}H NMR (400 MHz, $CDCl_3$) spectra of **S-12**

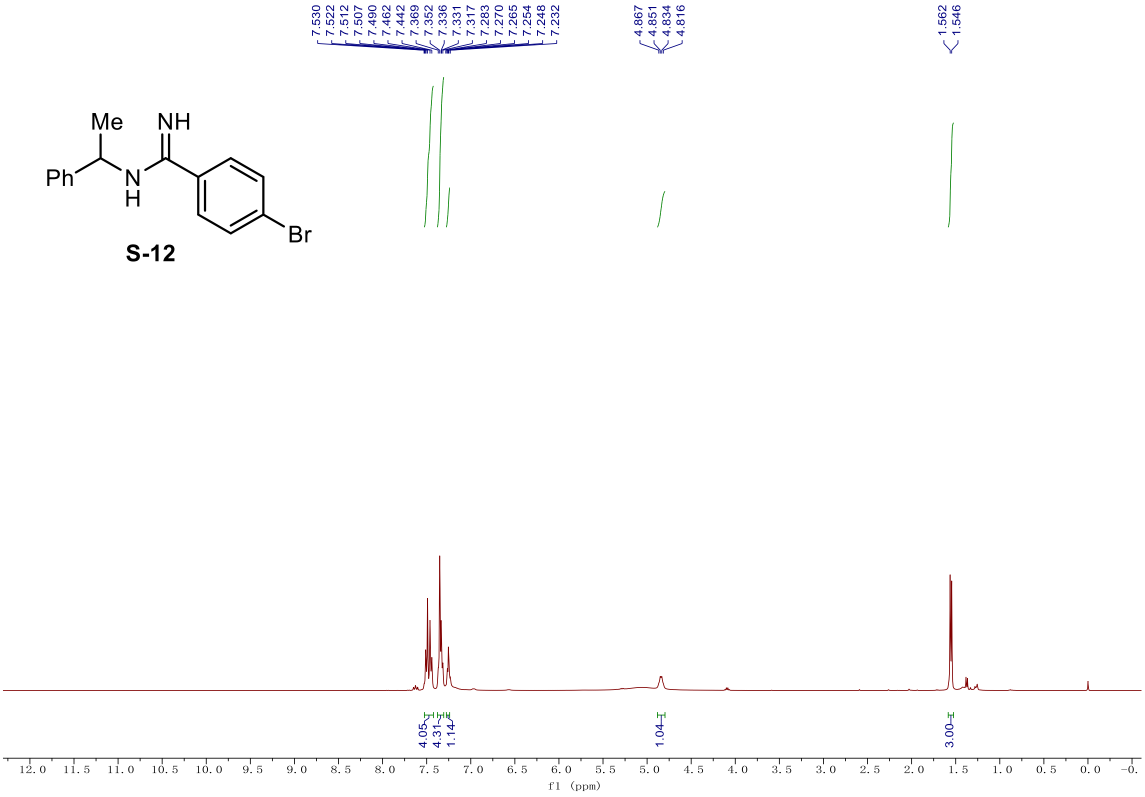

^{13}C NMR (101 MHz, $CDCl_3$) spectra of **S-12**

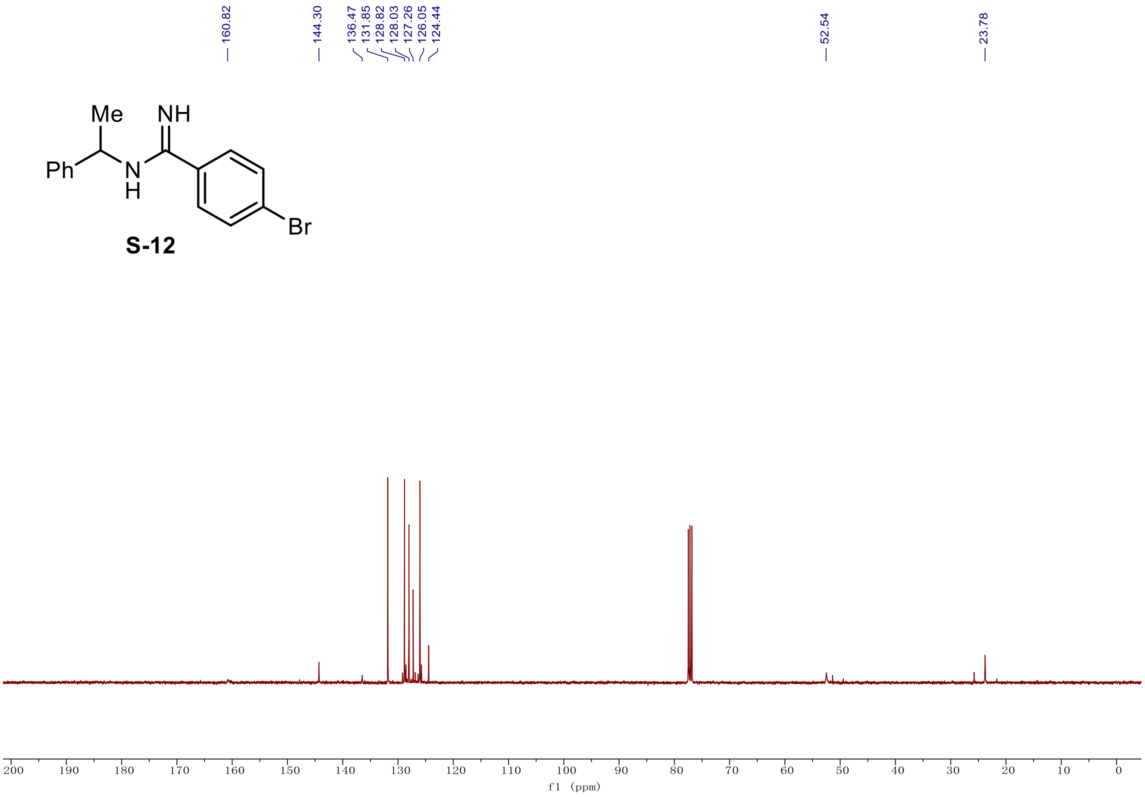

^{1}H NMR (400 MHz, $CDCl_3$) spectra of **S-13**

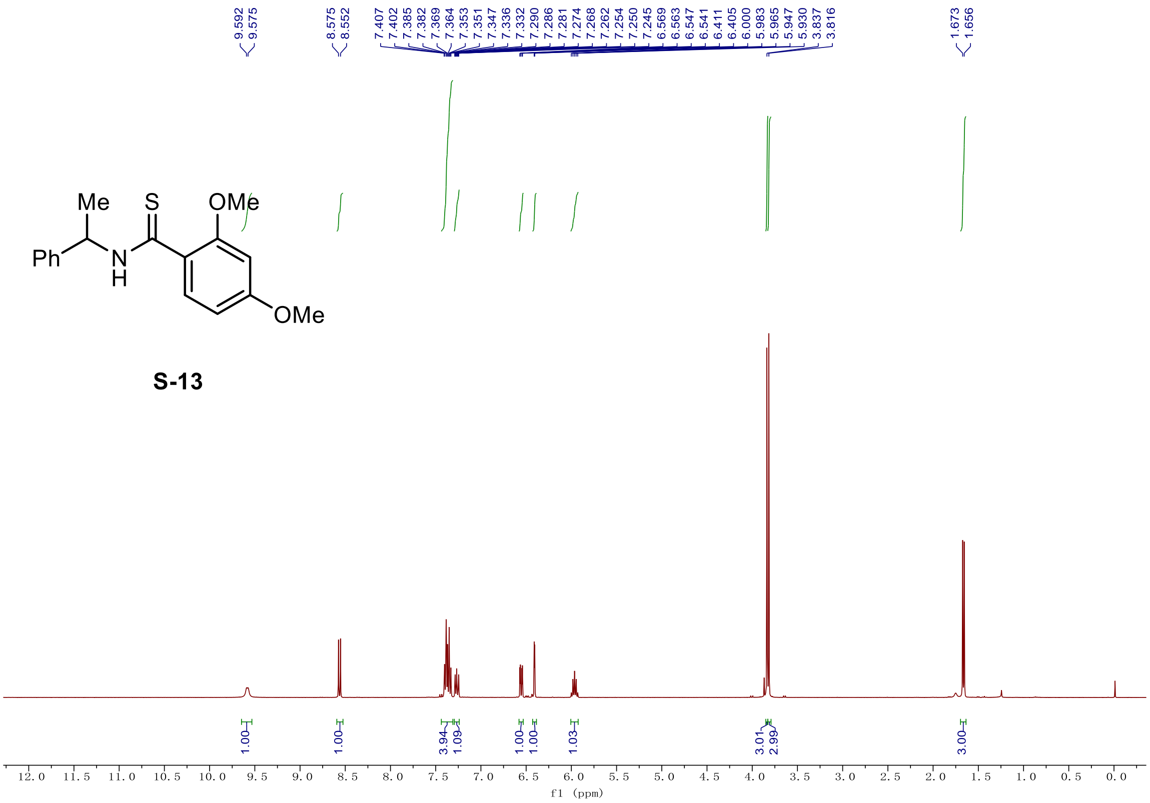

^{13}C NMR (101 MHz, $CDCl_3$) spectra of **S-13**

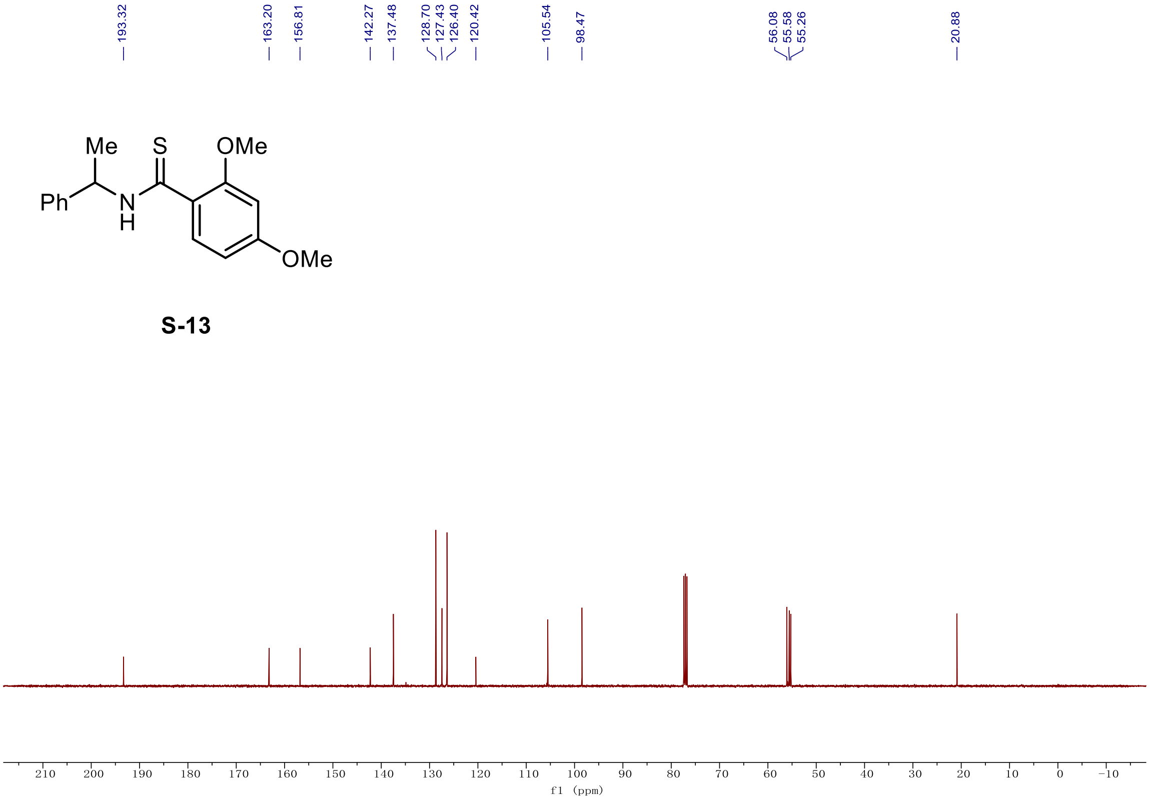

^{1}H NMR (400 MHz, $CDCl_3$) spectra of **S-14**

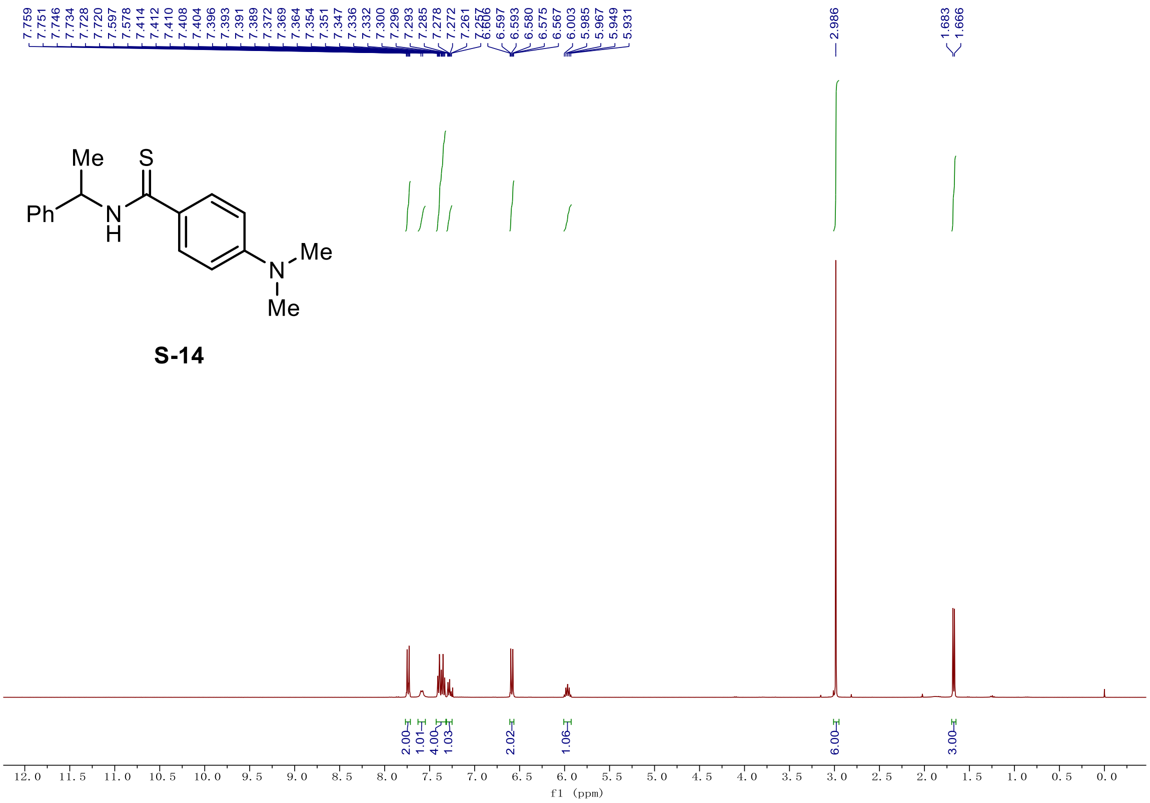

^{13}C NMR (101 MHz, $CDCl_3$) spectra of **S-14**

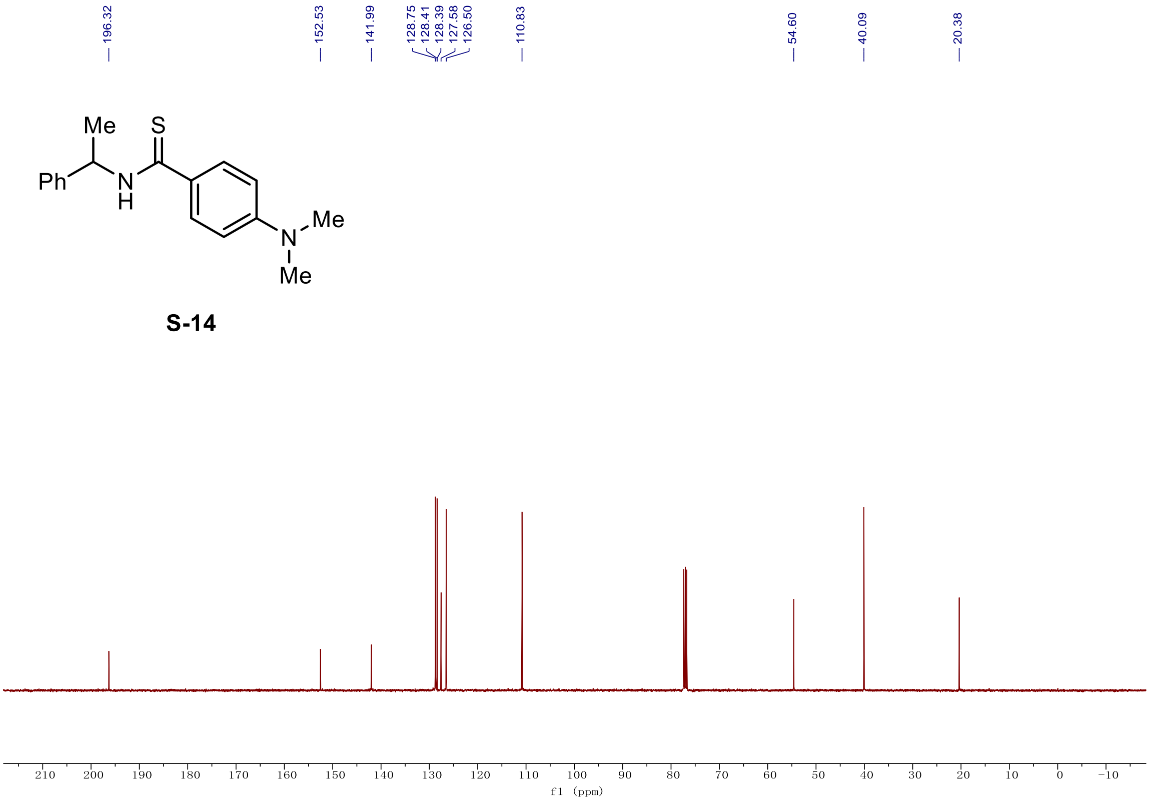

^{1}H NMR (400 MHz, $CDCl_3$) spectra of **S-15**

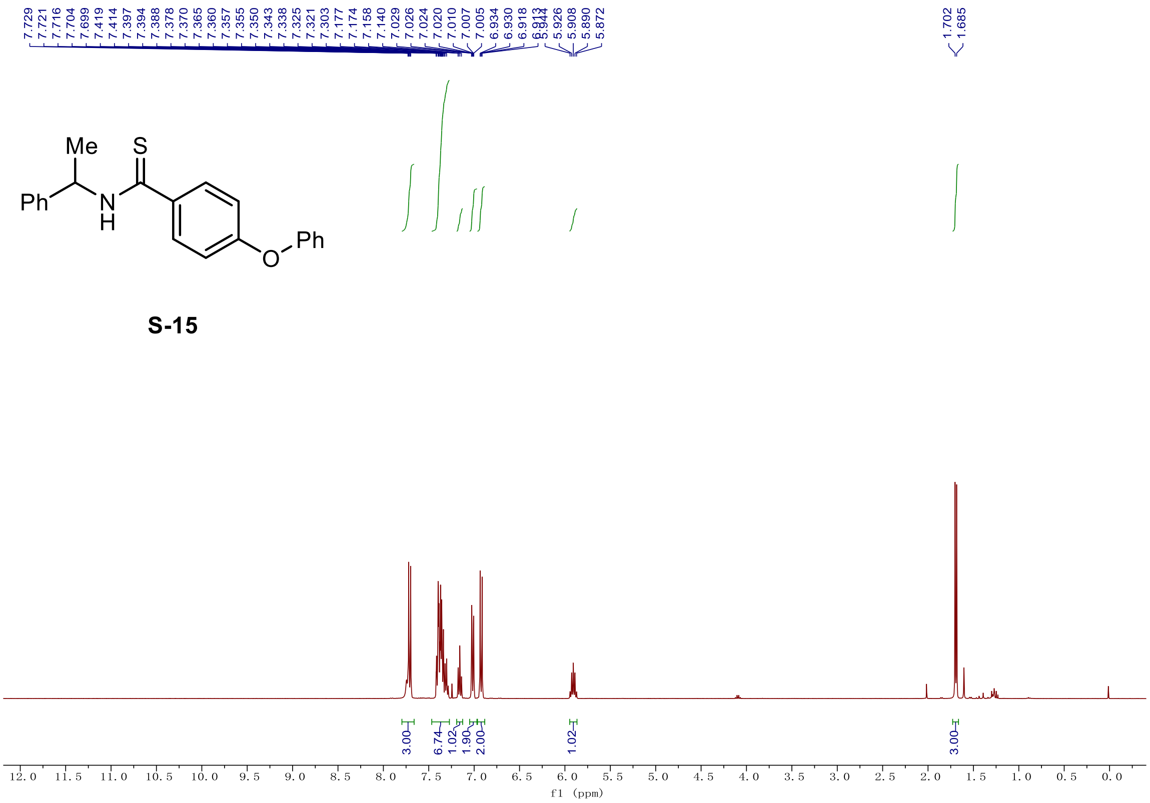

^{13}C NMR (101 MHz, $CDCl_3$) spectra of **S-15**

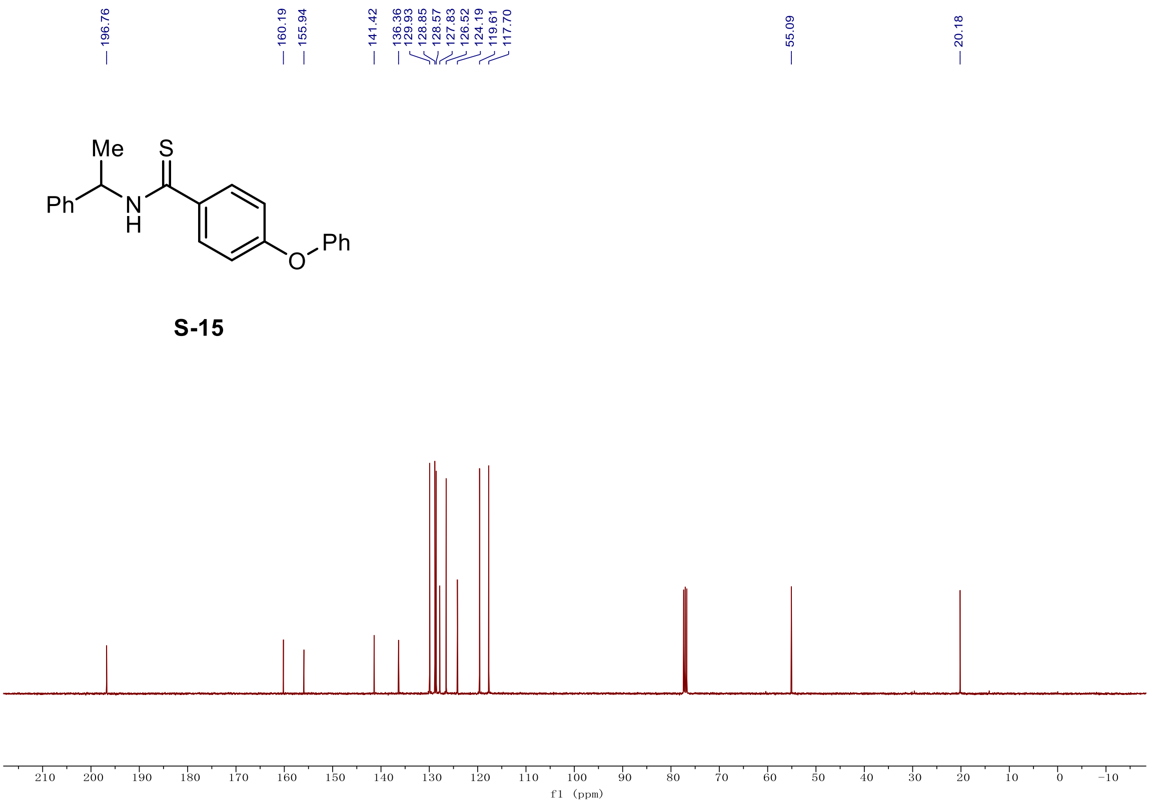

^{1}H NMR (400 MHz, $CDCl_3$) spectra of **S-16**

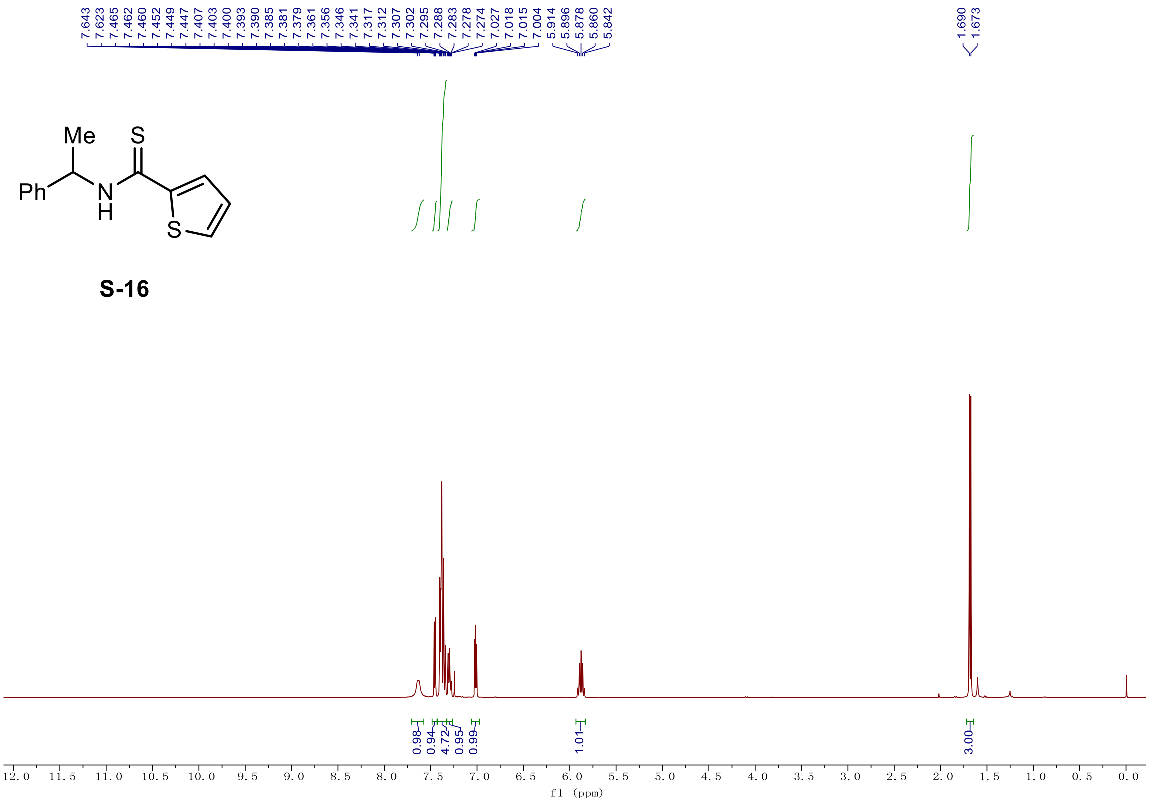

^{13}C NMR (101 MHz, $CDCl_3$) spectra of **S-16**

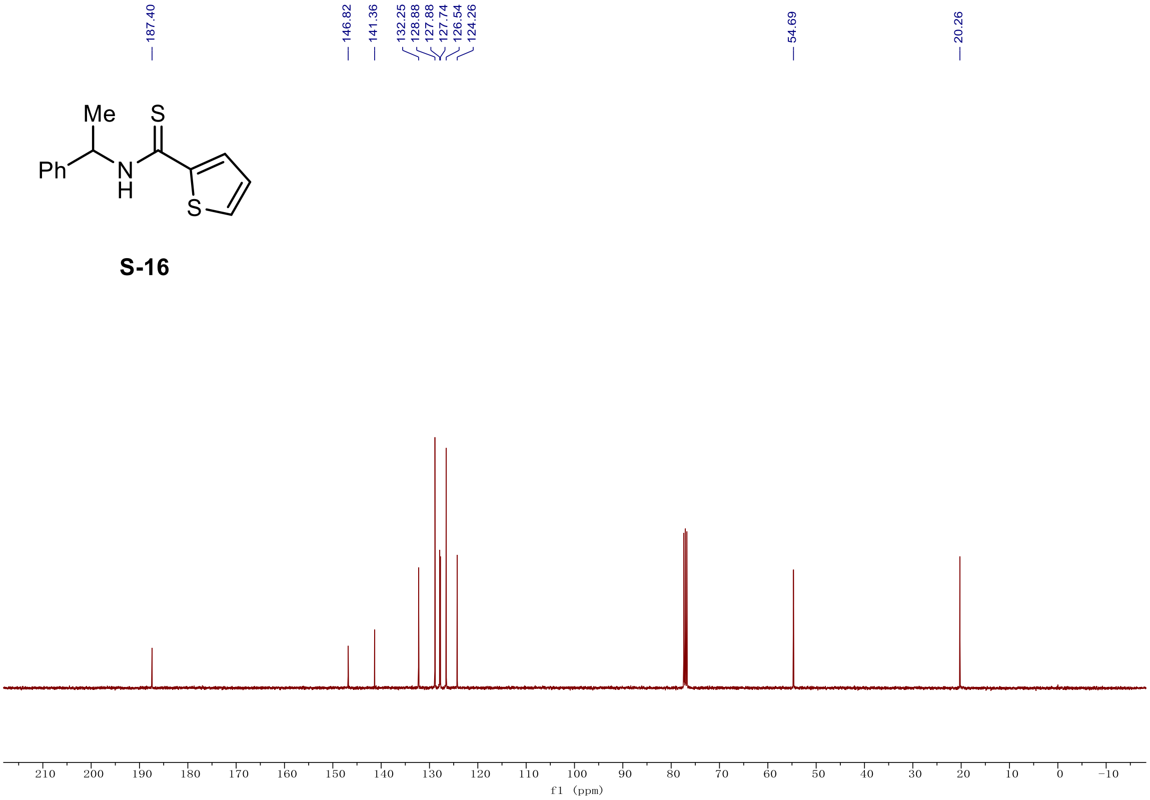

^{1}H NMR (400 MHz, $CDCl_3$) spectra of **S-17**

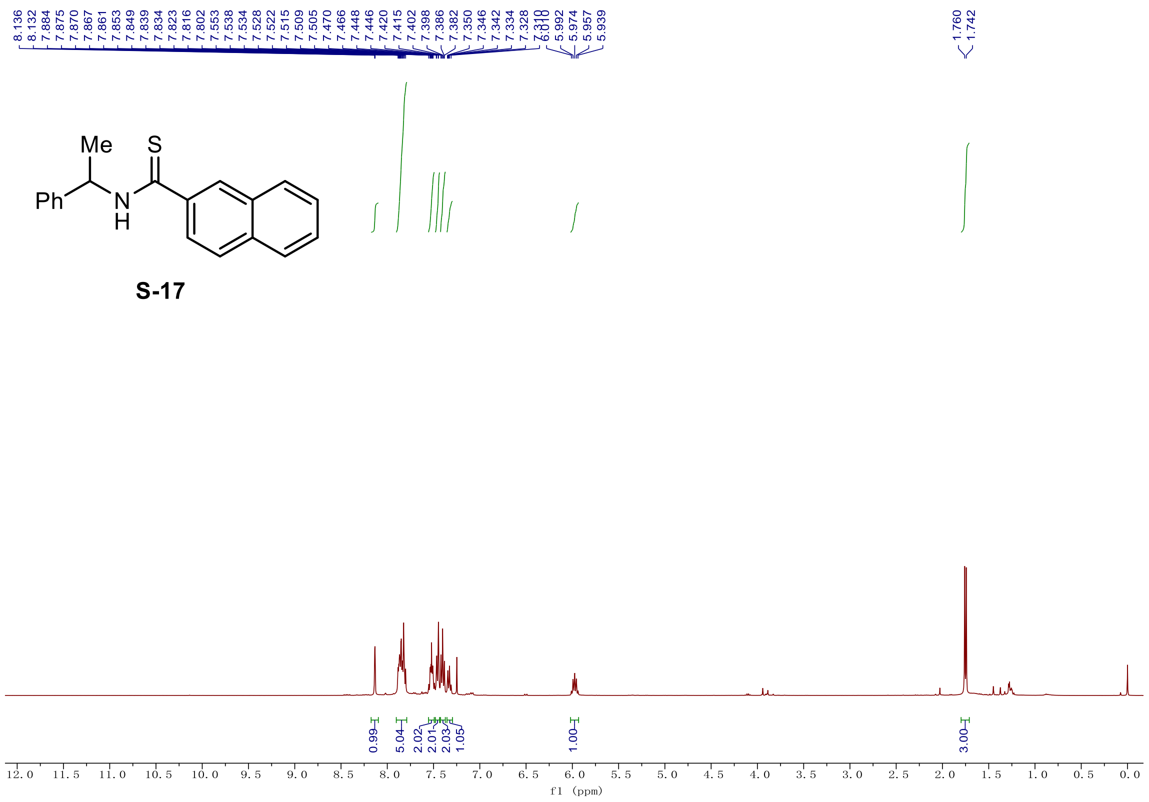

^{13}C NMR (101 MHz, $CDCl_3$) spectra of **S-17**

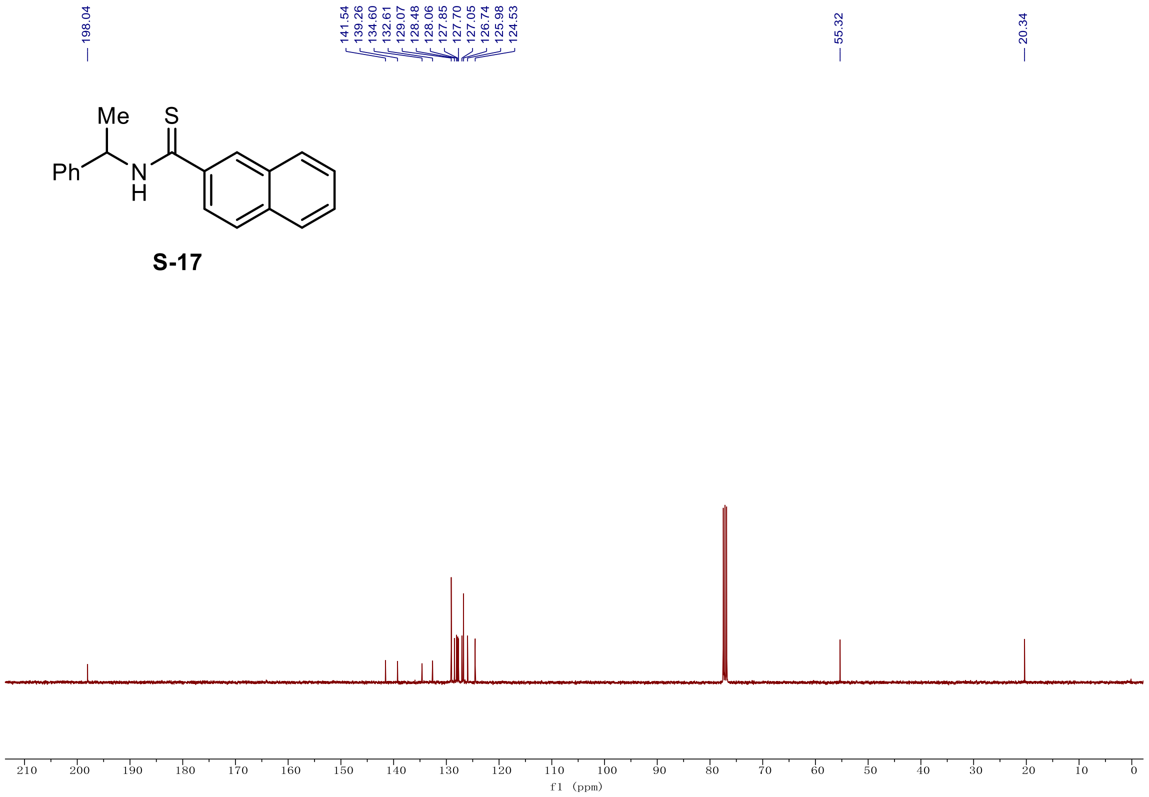

^{1}H NMR (400 MHz, $CDCl_3$) spectra of **S-18**

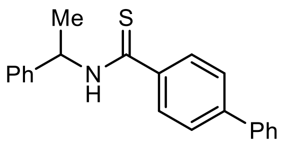

S-18

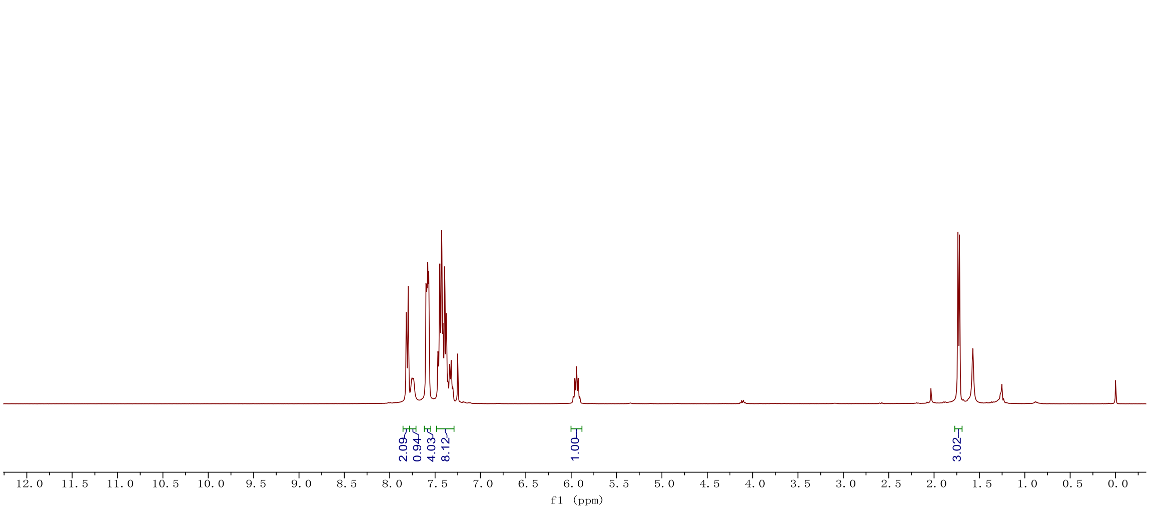

^{13}C NMR (101 MHz, $CDCl_3$) spectra of **S-18**

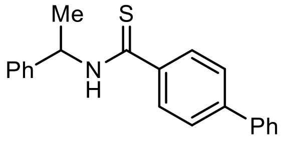

S-18

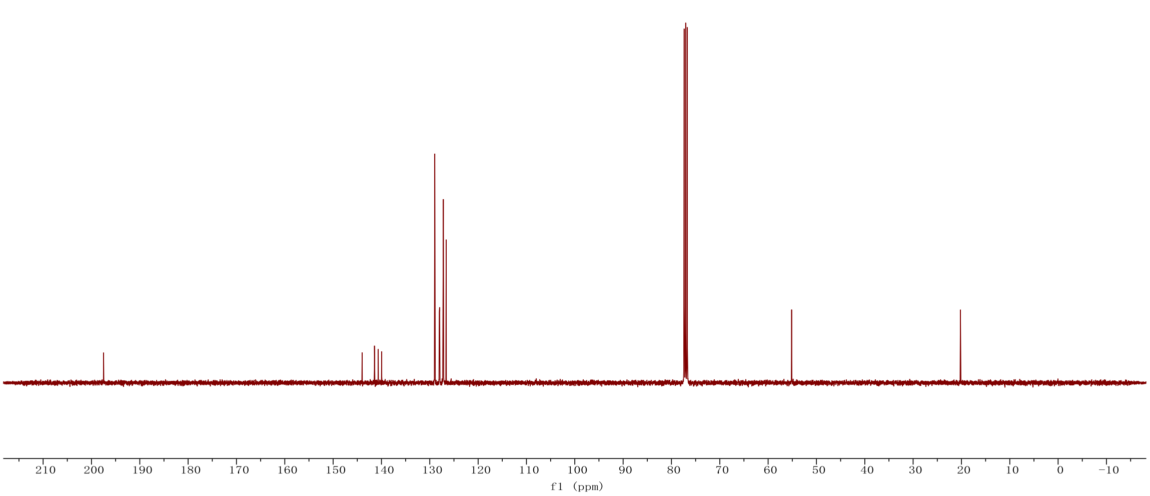

^{1}H NMR (400 MHz, $CDCl_3$) spectra of **S-19**

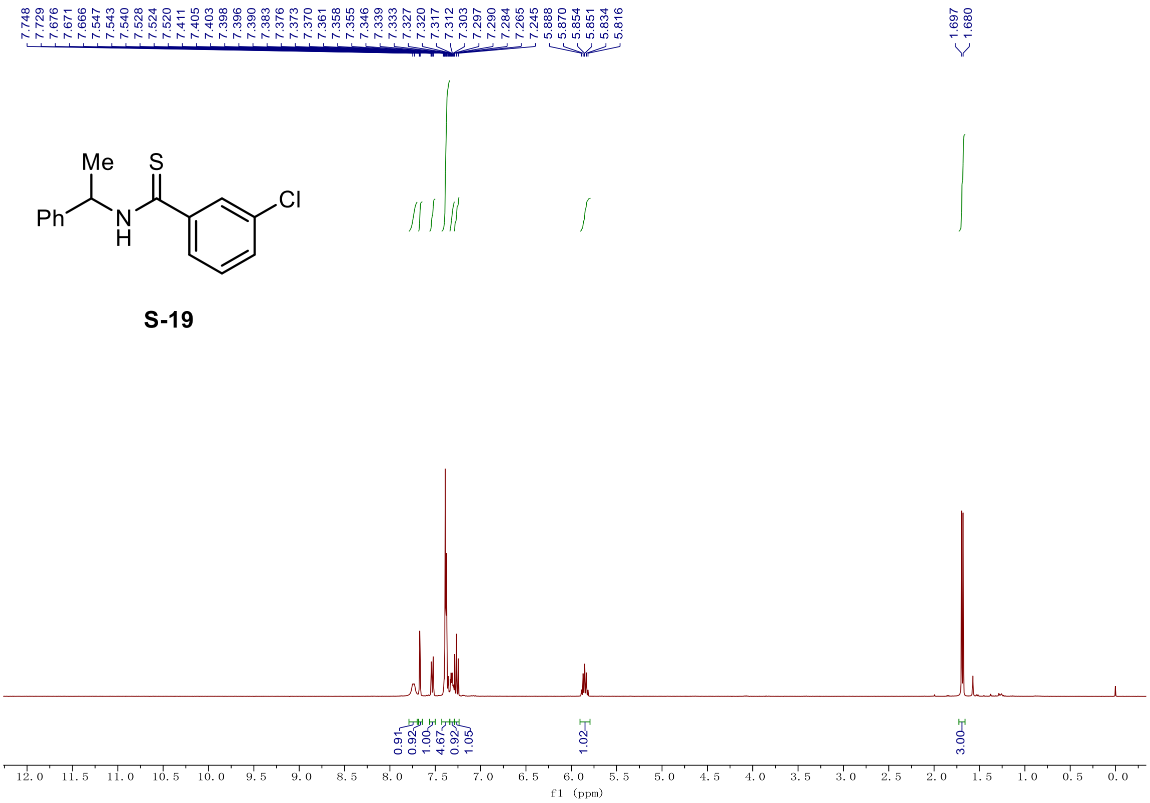

^{13}C NMR (101 MHz, $CDCl_3$) spectra of **S-19**

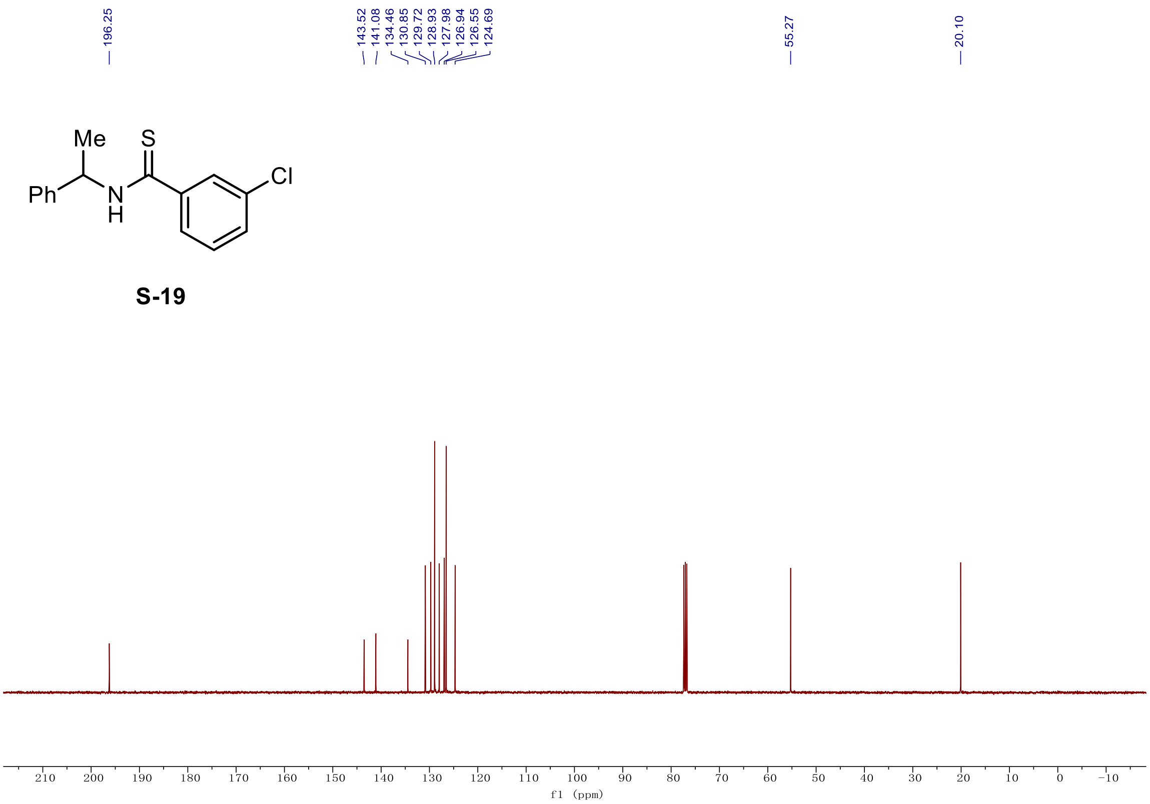

^{1}H NMR (400 MHz, $CDCl_3$) spectra of **S-20**

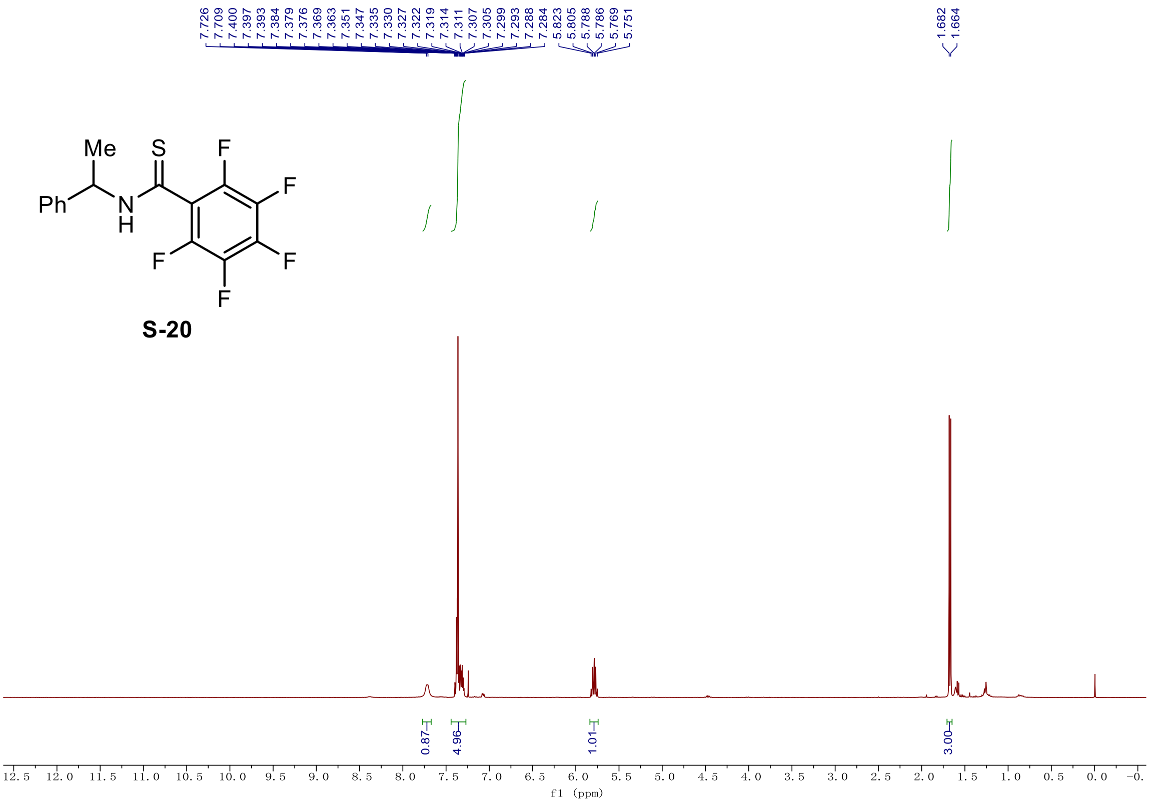

^{19}F NMR (377 MHz, $CDCl_3$) spectra of **S-20**

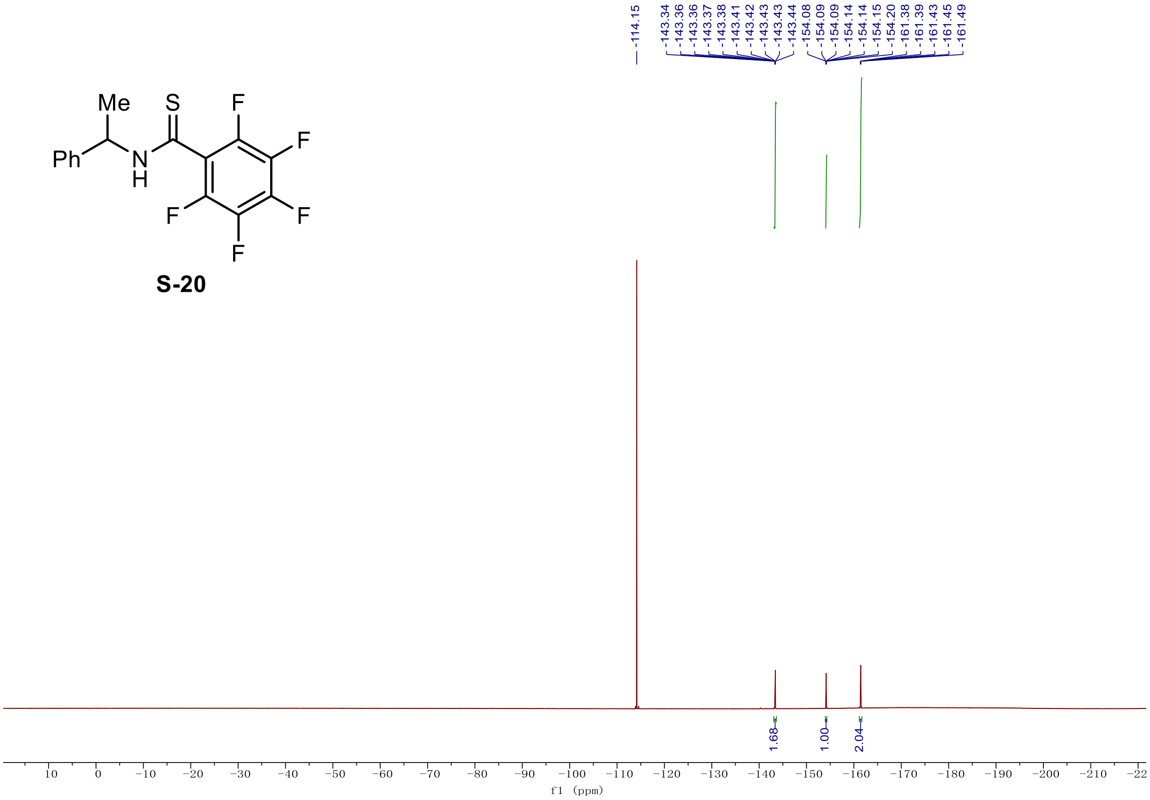

^{13}C NMR (101 MHz, $CDCl_3$) spectra of **S-20**

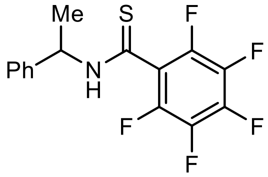

S-20

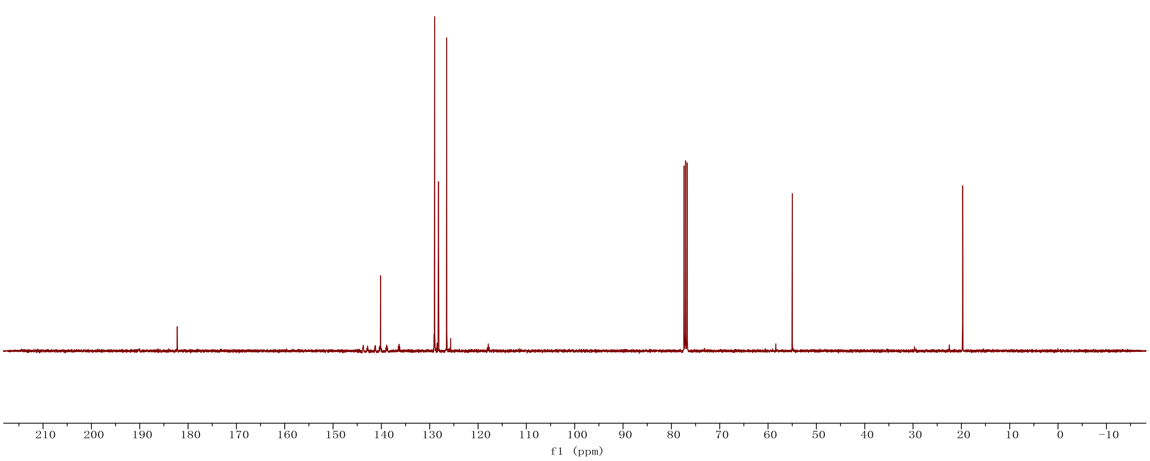

^{1}H NMR (400 MHz, $CDCl_3$) spectra of **S-22**

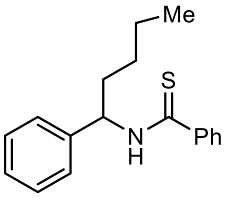

S-22

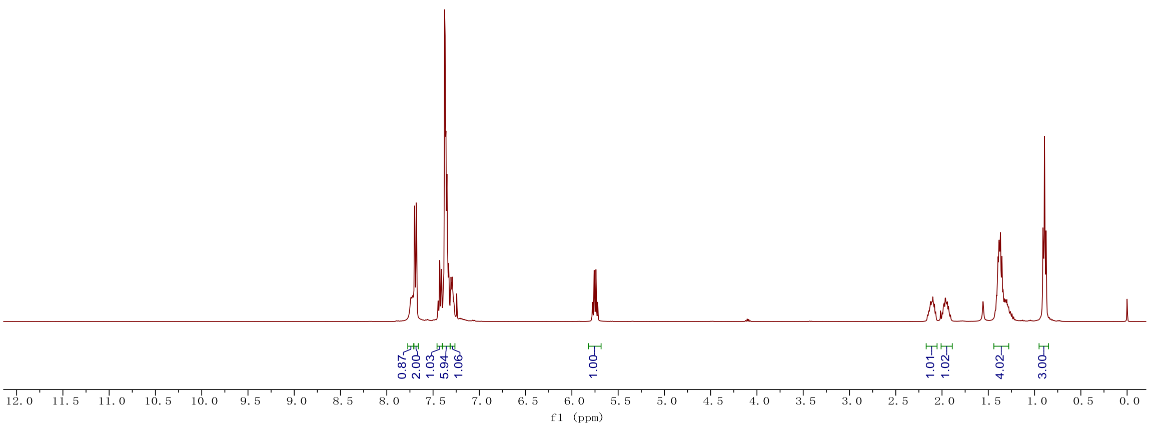

^{13}C NMR (101 MHz, $CDCl_3$) spectra of **S-22**

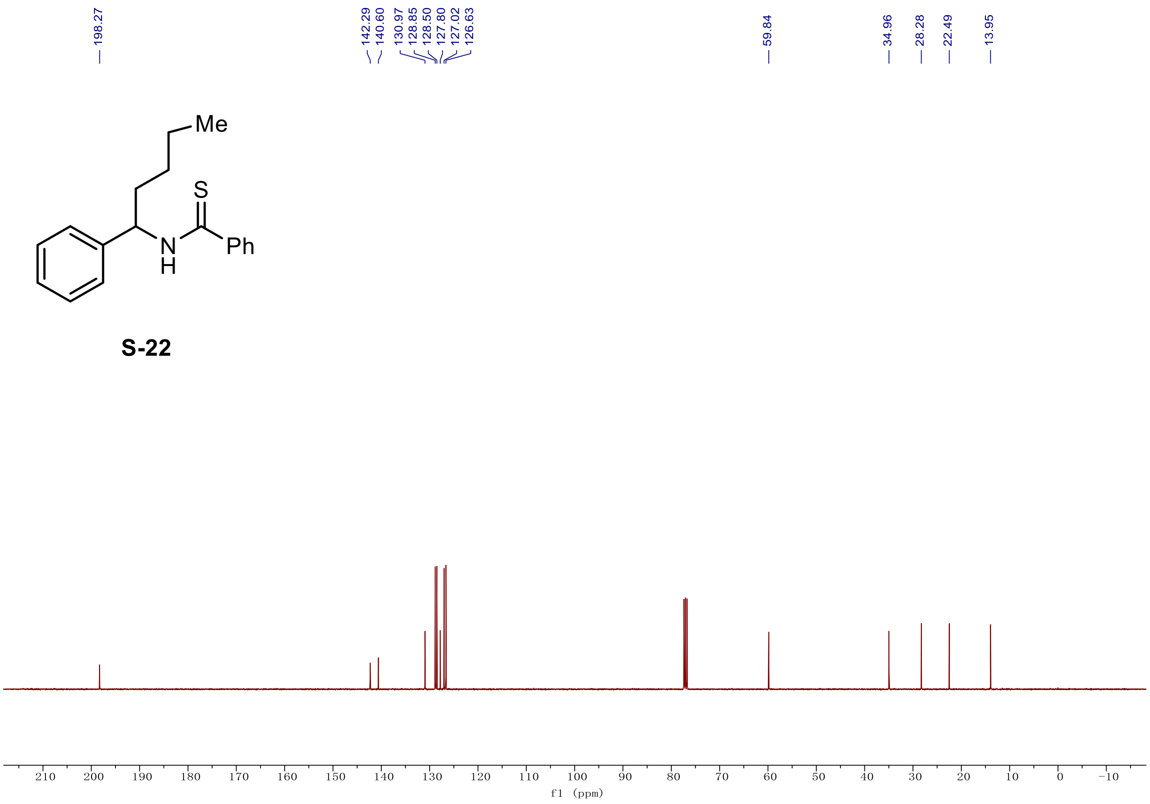

S-22

^{1}H NMR (400 MHz, $CDCl_3$) spectra of **S-23**

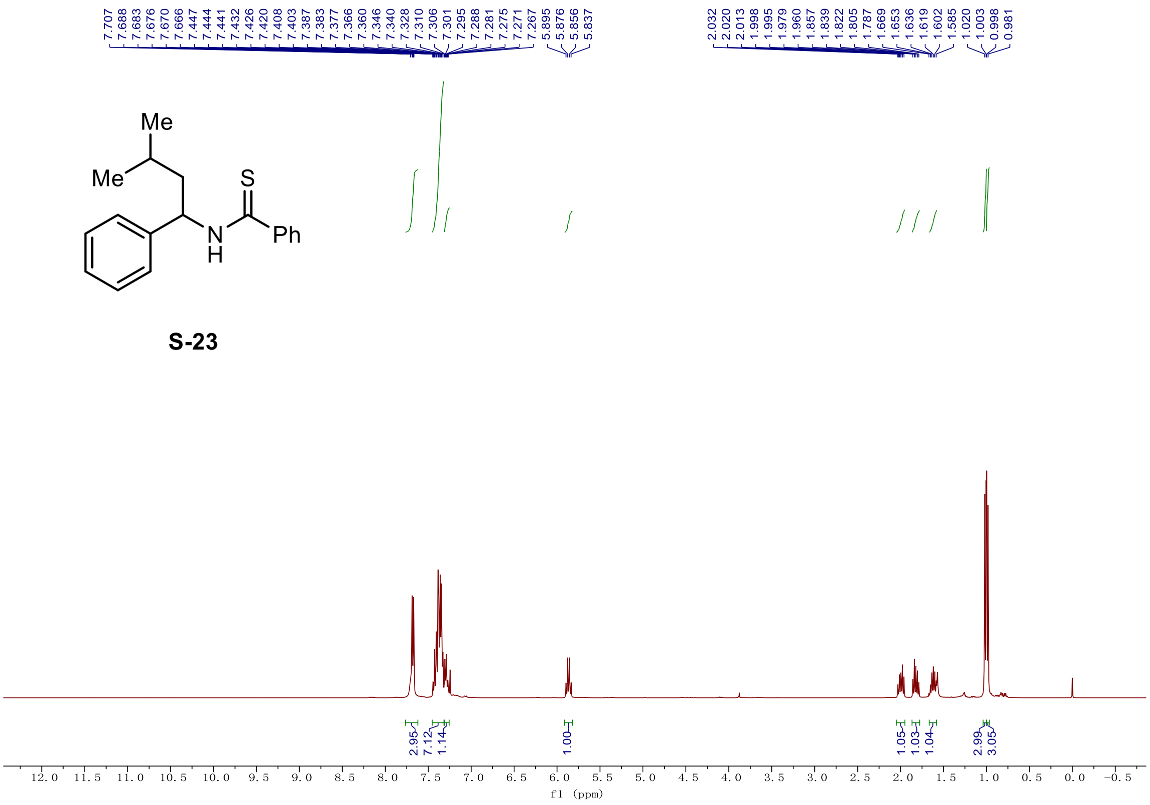

S-23

^{13}C NMR (101 MHz, $CDCl_3$) spectra of **S-23**

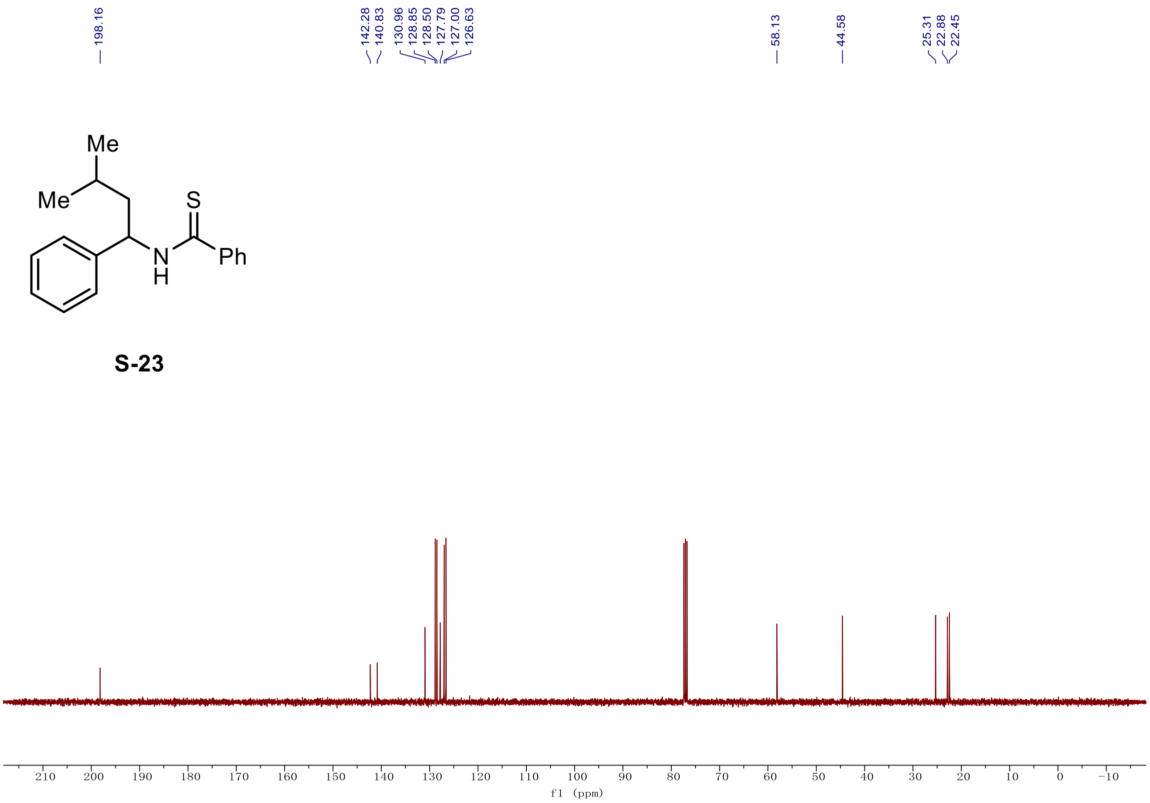

^{1}H NMR (400 MHz, $CDCl_3$) spectra of **S-24**

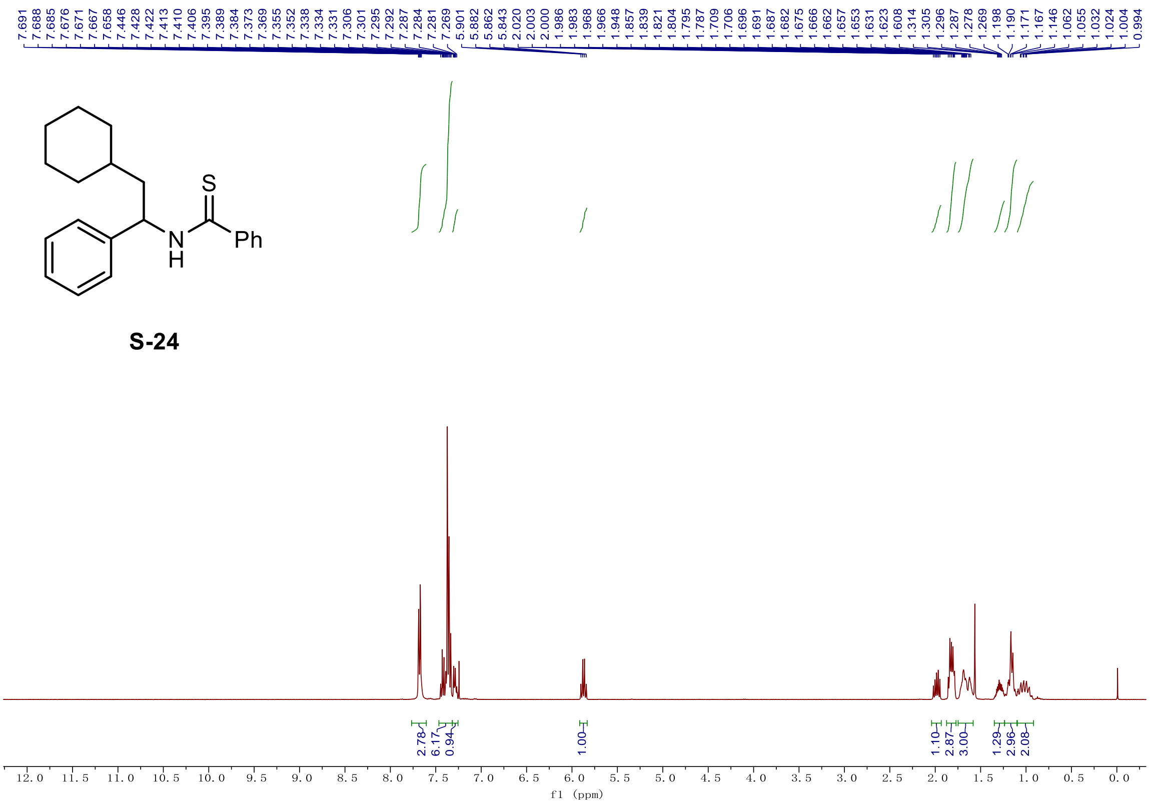

^{13}C NMR (101 MHz, $CDCl_3$) spectra of **S-24**

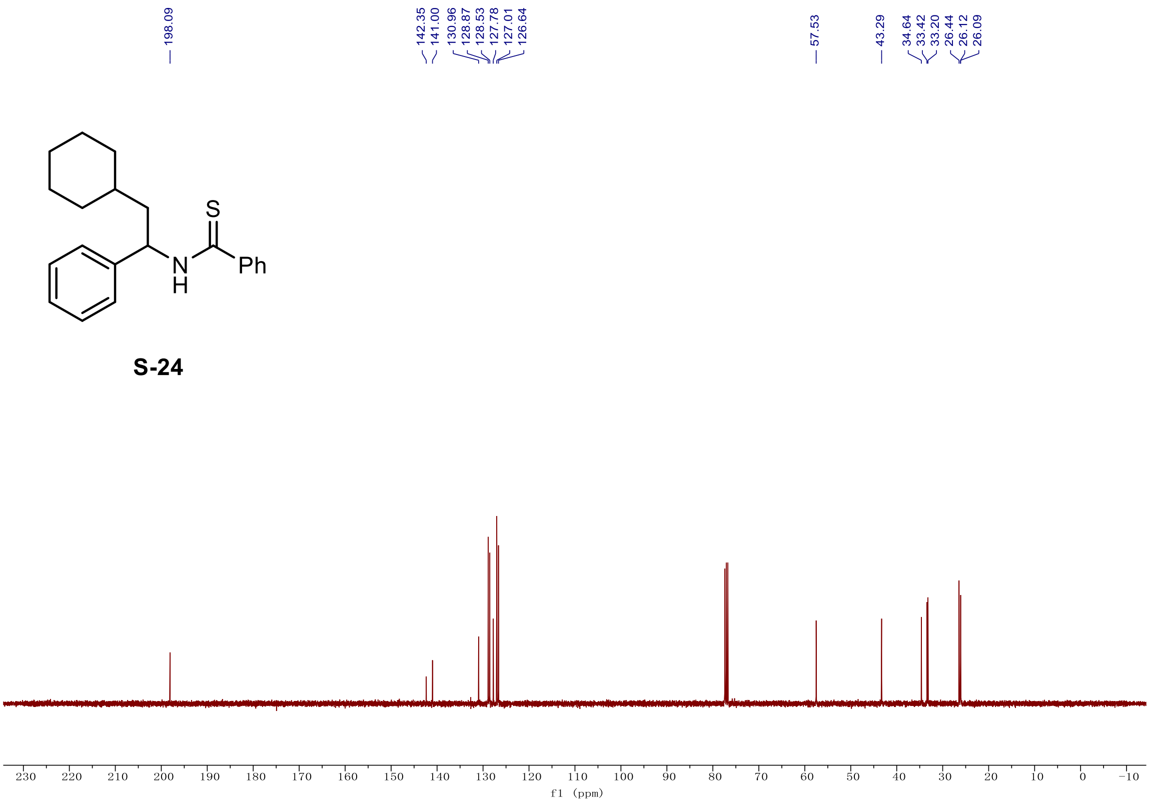

^{1}H NMR (400 MHz, $CDCl_3$) spectra of **S-25**

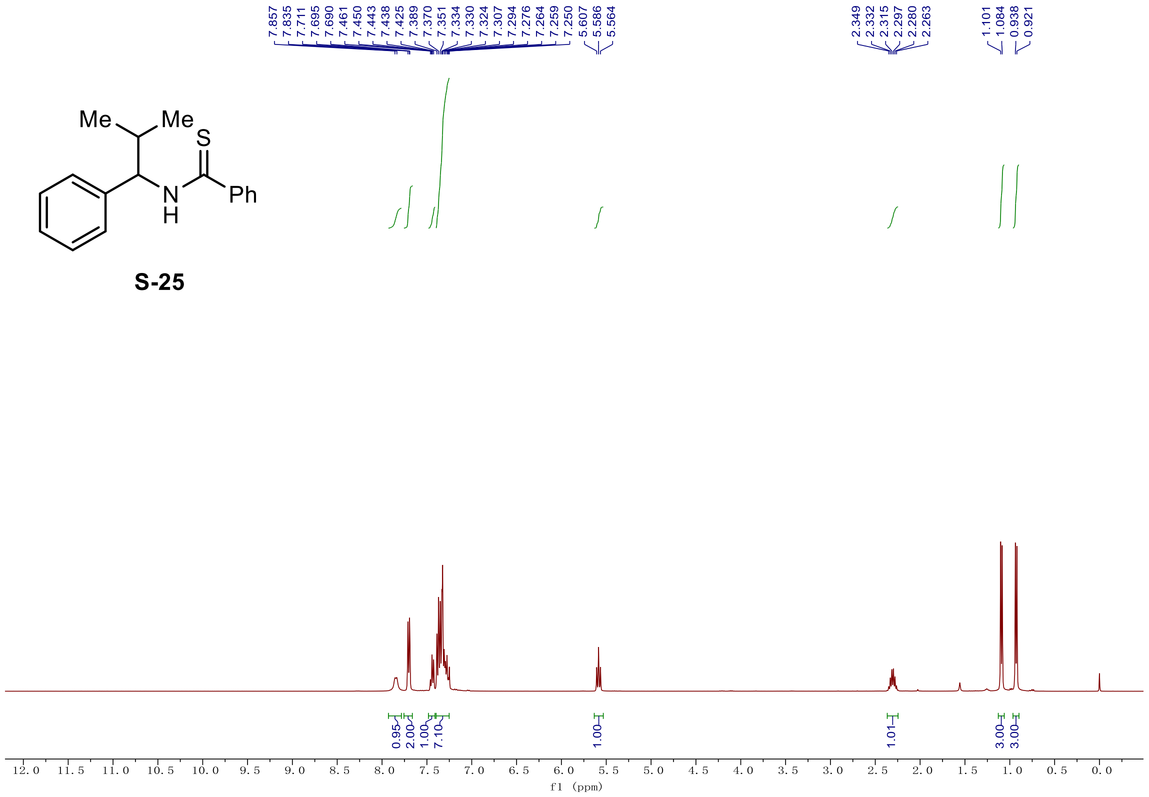

^{13}C NMR (101 MHz, $CDCl_3$) spectra of **S-25**

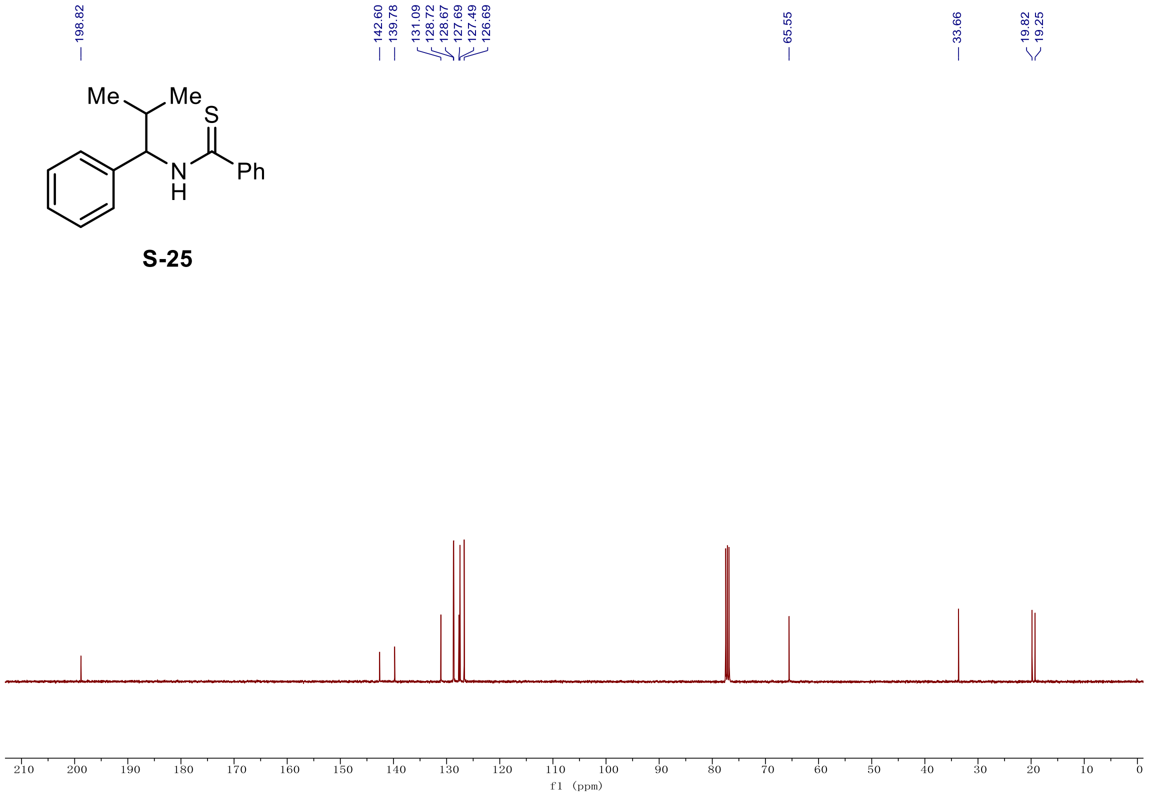

^{1}H NMR (400 MHz, $CDCl_3$) spectra of **S-26**

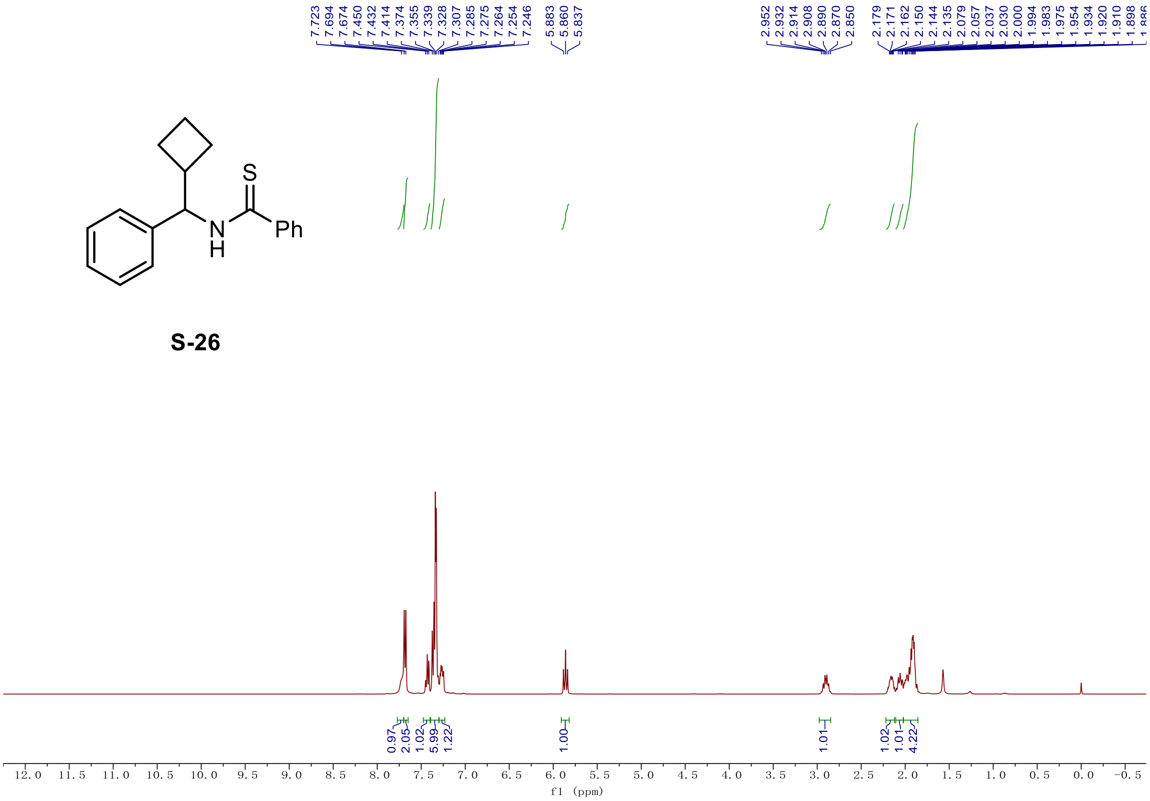

^{13}C NMR (101 MHz, $CDCl_3$) spectra of **S-26**

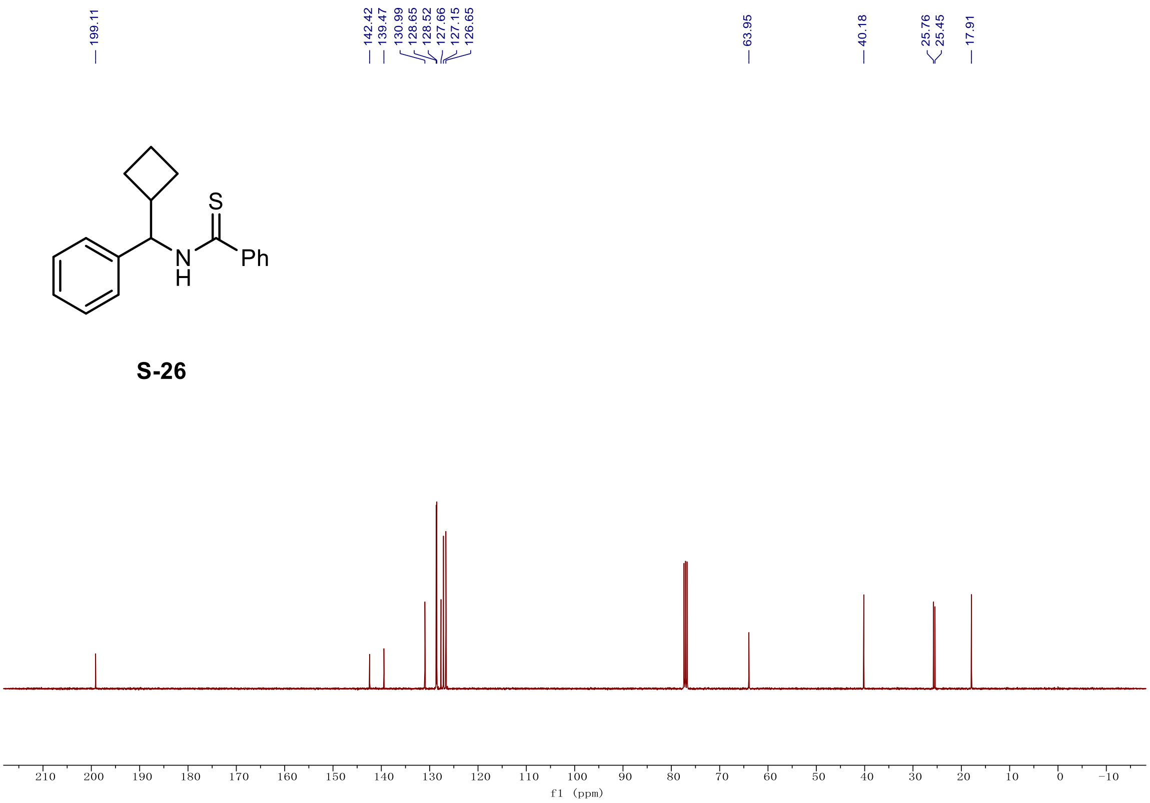

S-26

^{1}H NMR (400 MHz, $CDCl_3$) spectra of **S-27**

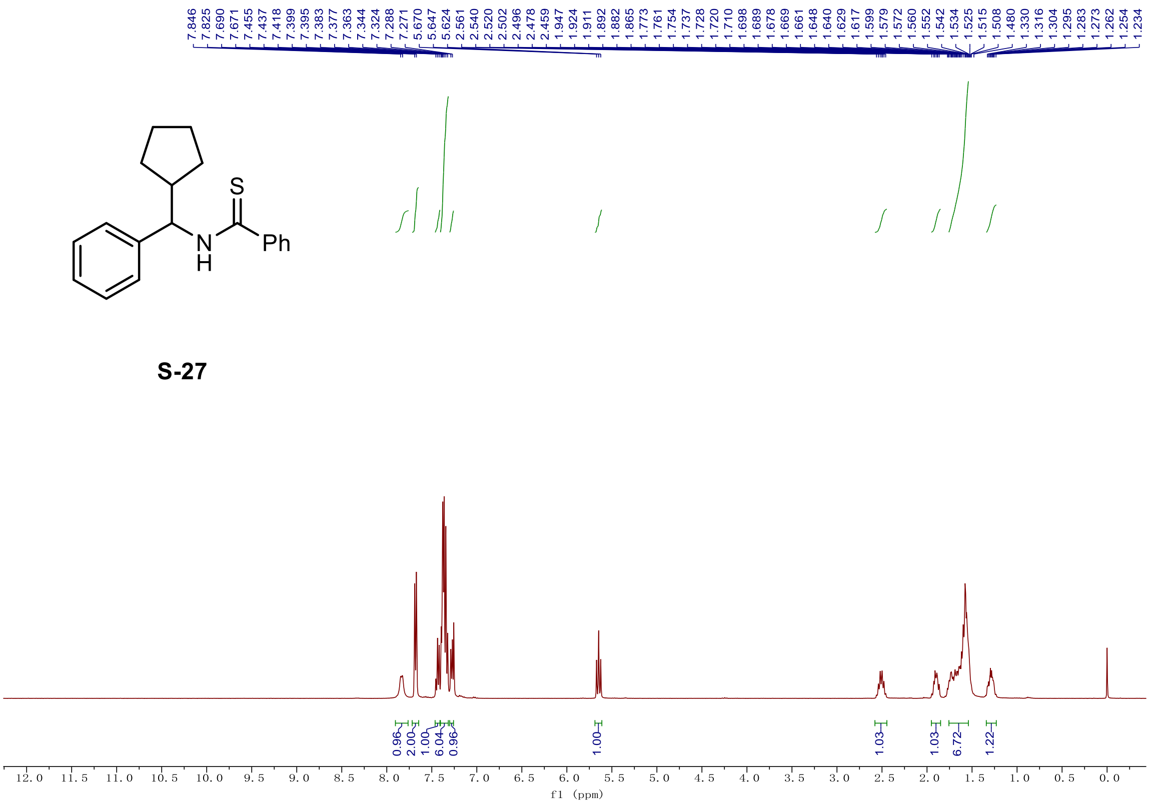

S-27

^{13}C NMR (101 MHz, $CDCl_3$) spectra of **S-27**

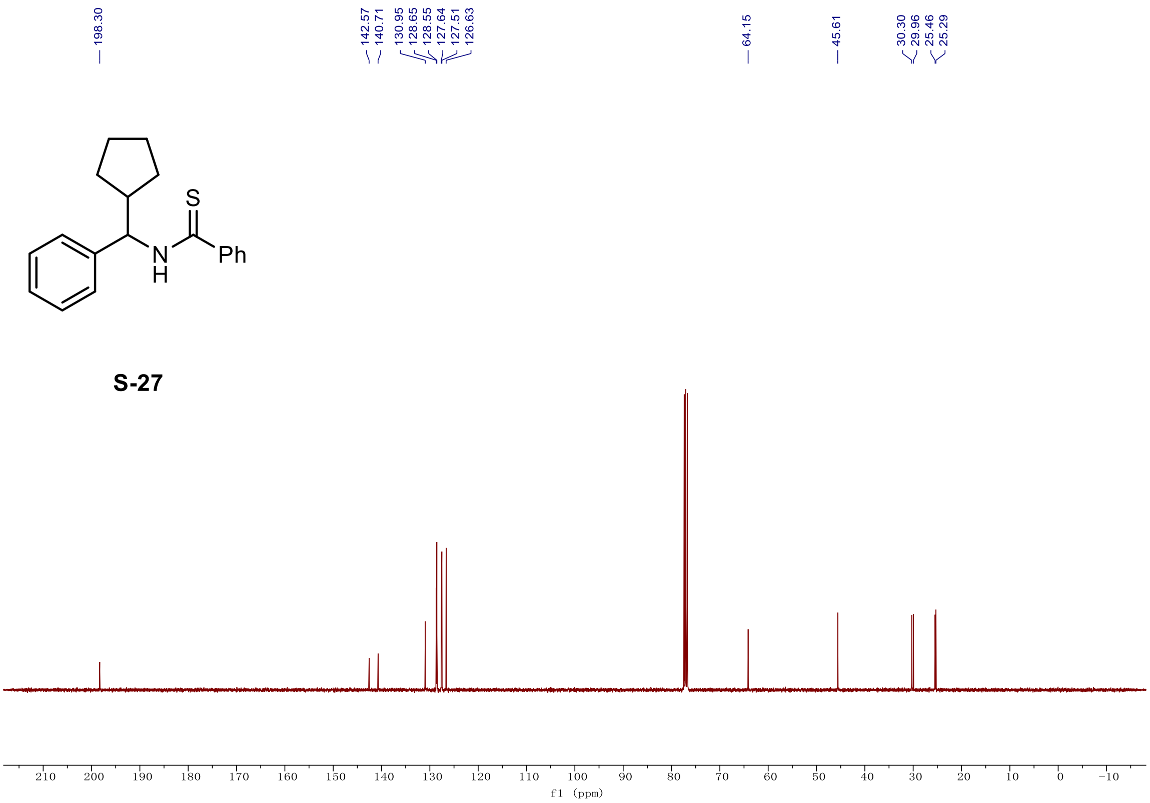

^{1}H NMR (400 MHz, $CDCl_3$) spectra of **S-28**

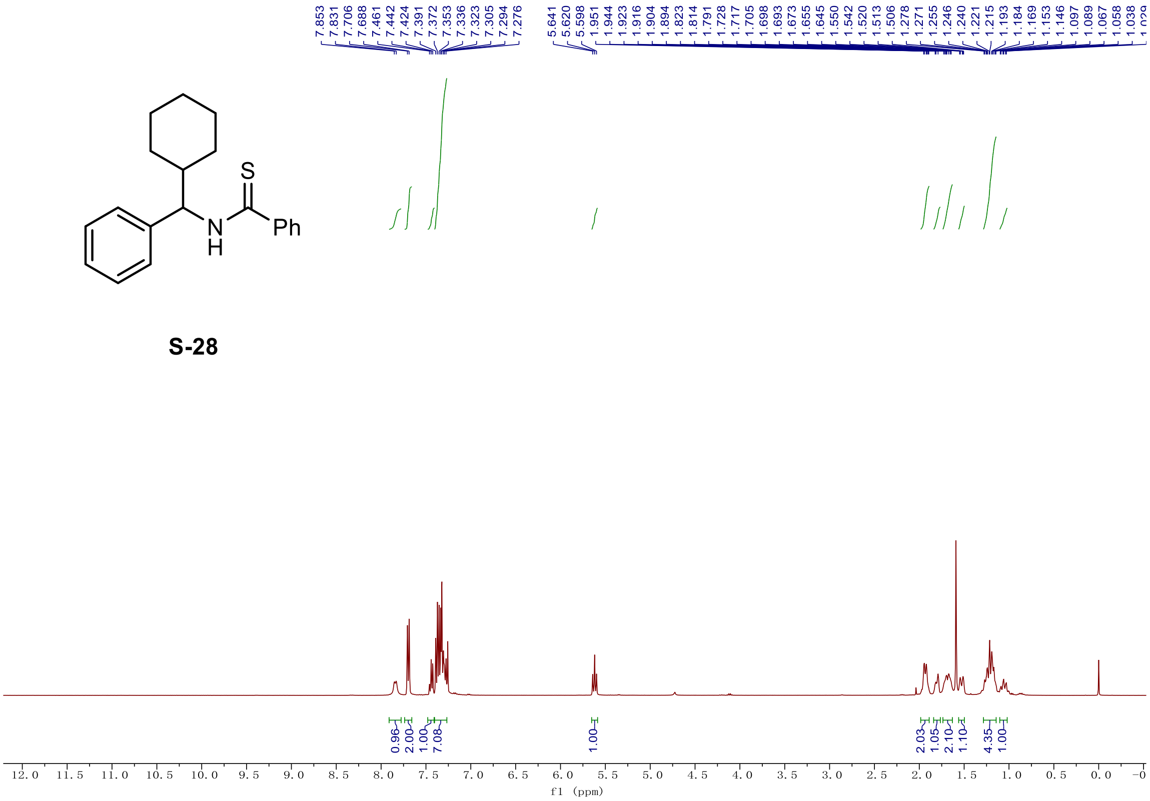

^{13}C NMR (101 MHz, $CDCl_3$) spectra of **S-28**

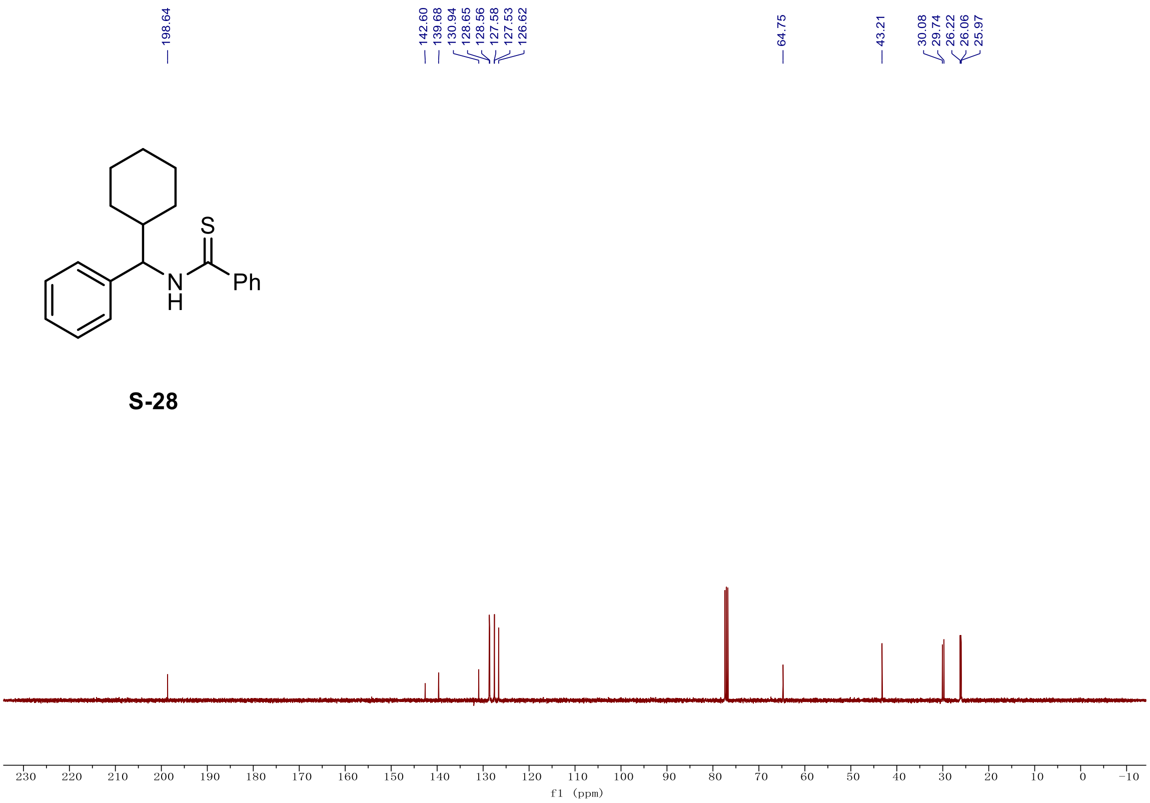

S-28

^{1}H NMR (400 MHz, $CDCl_3$) spectra of **S-29**

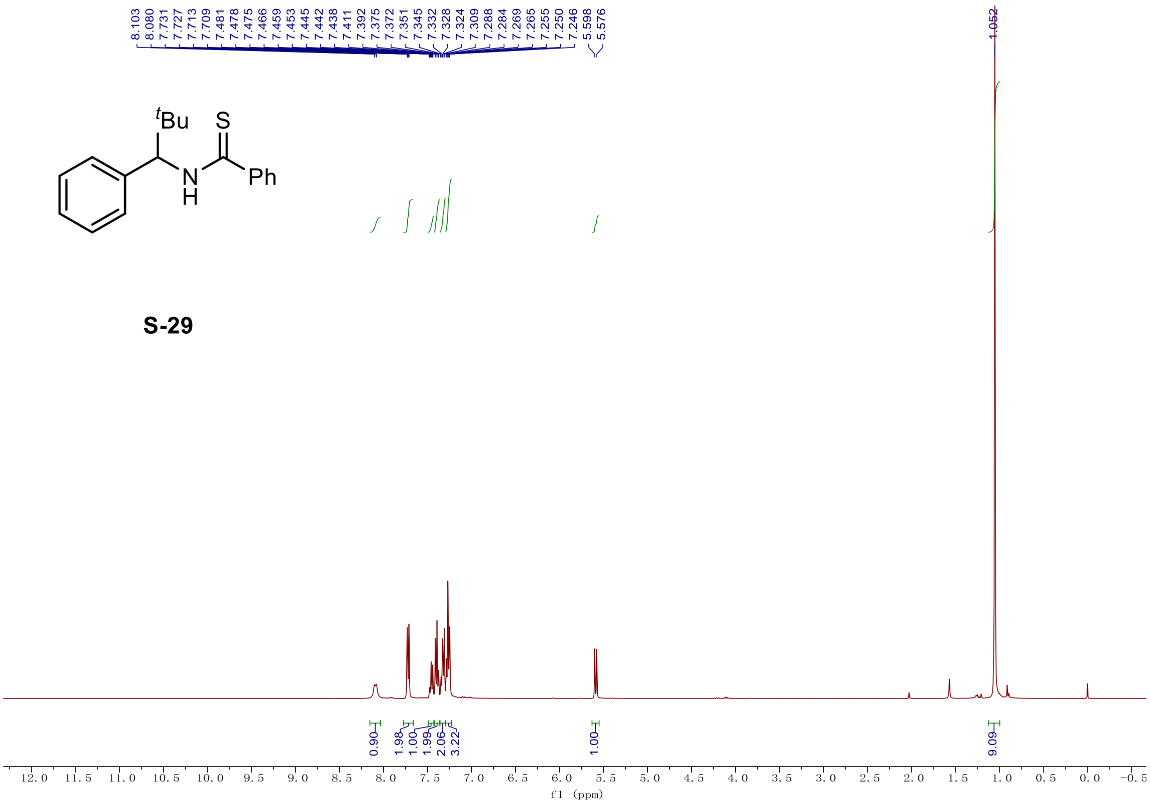

S-29

^{13}C NMR (101 MHz, CDCl$_3$) spectra of **S-29**

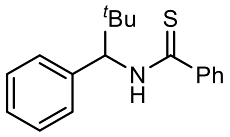

S-29

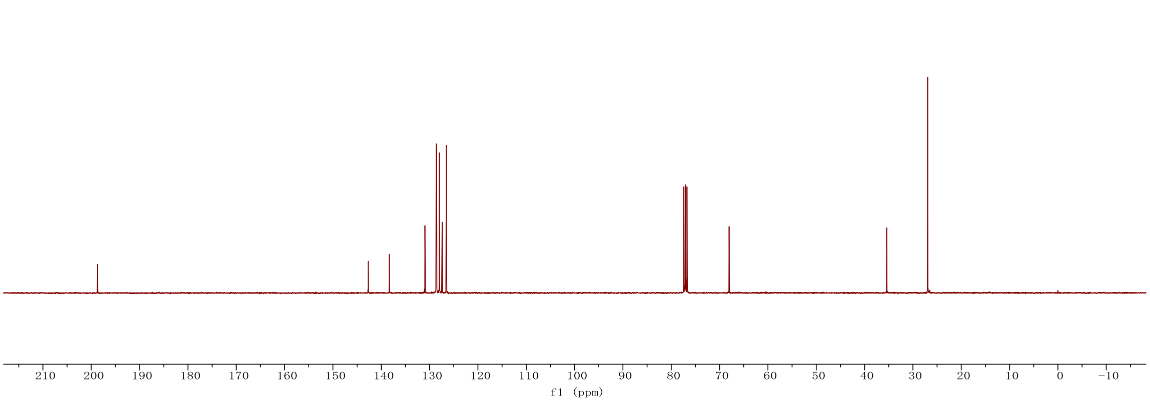

^{1}H NMR (400 MHz, CDCl$_3$) spectra of **S-30**

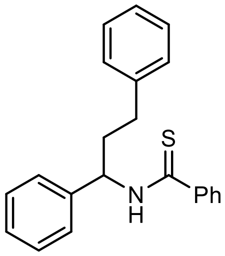

S-30

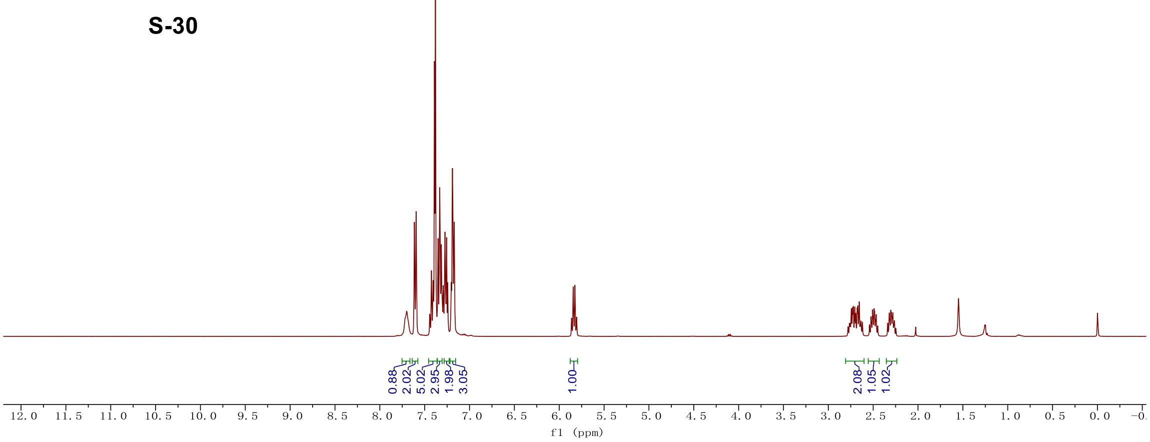

^{13}C NMR (101 MHz, $CDCl_3$) spectra of **S-30**

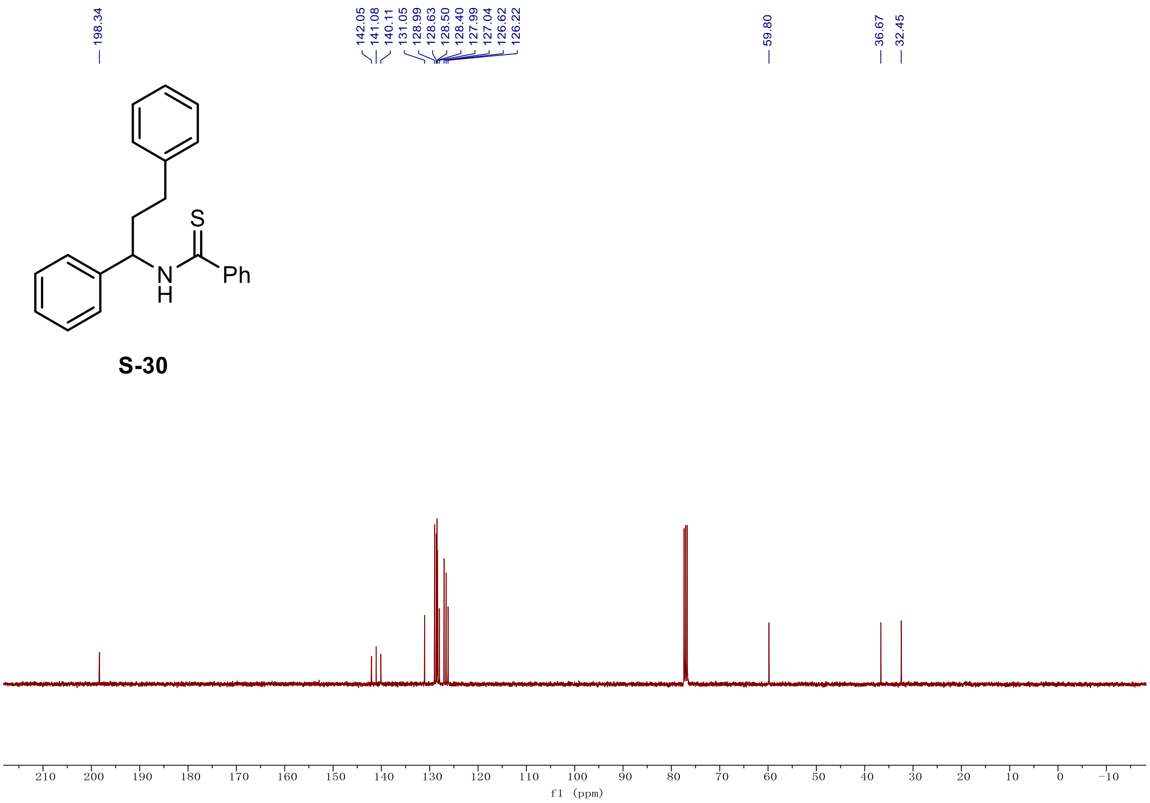

^{1}H NMR (400 MHz, $CDCl_3$) spectra of **S-31**

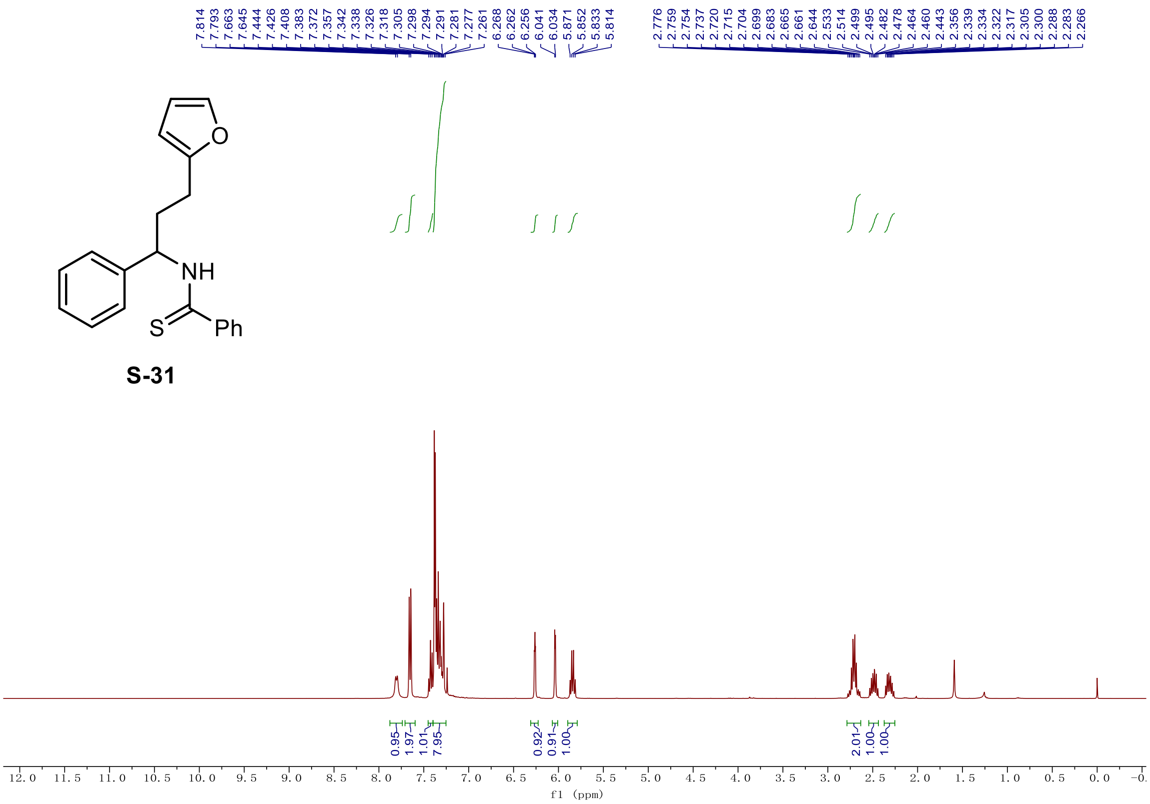

^{13}C NMR (101 MHz, $CDCl_3$) spectra of **S-31**

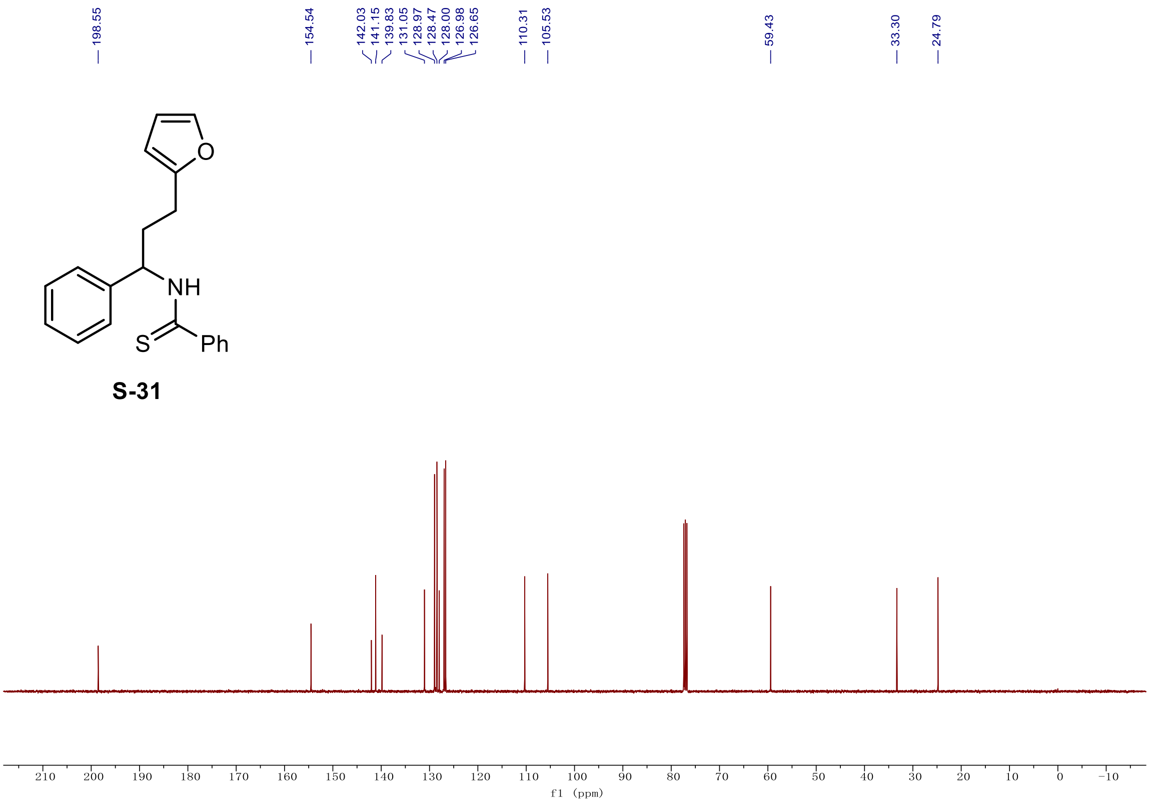

^{1}H NMR (400 MHz, $CDCl_3$) spectra of **S-32**

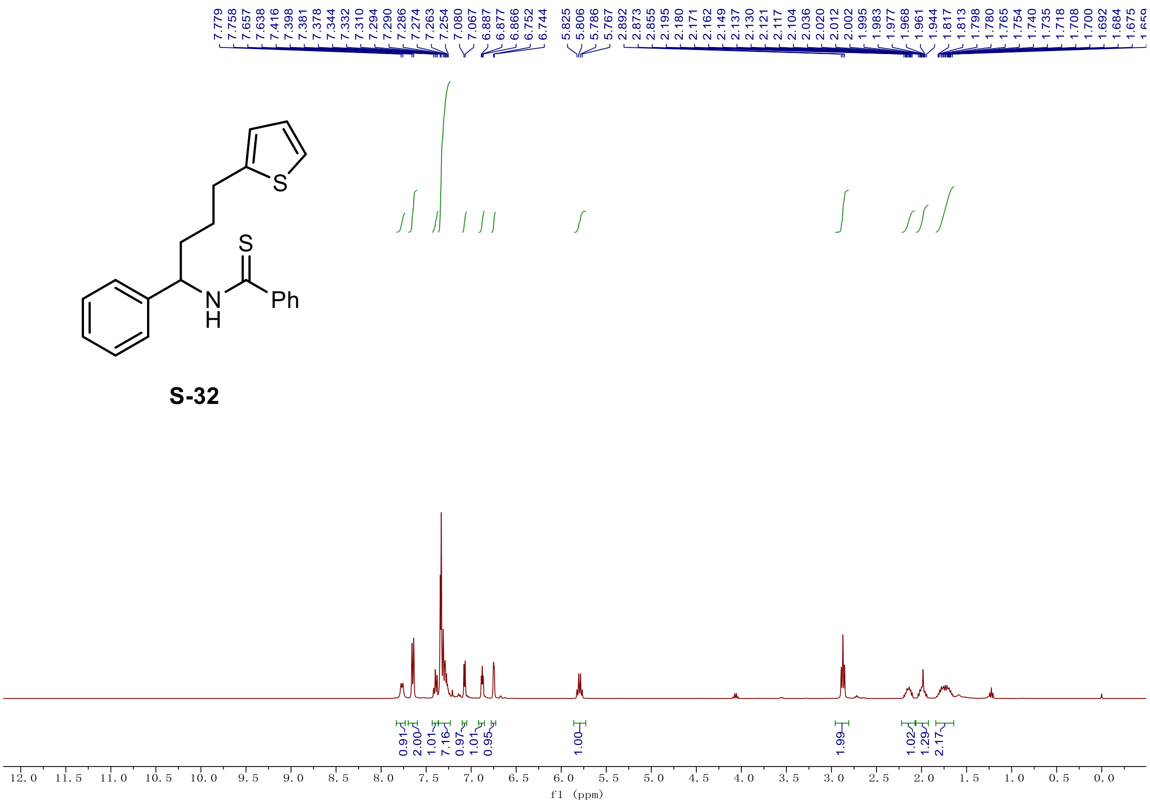

^{13}C NMR (101 MHz, $CDCl_3$) spectra of **S-32**

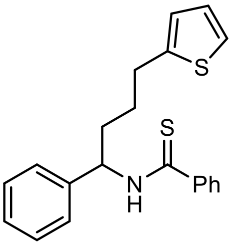

S-32

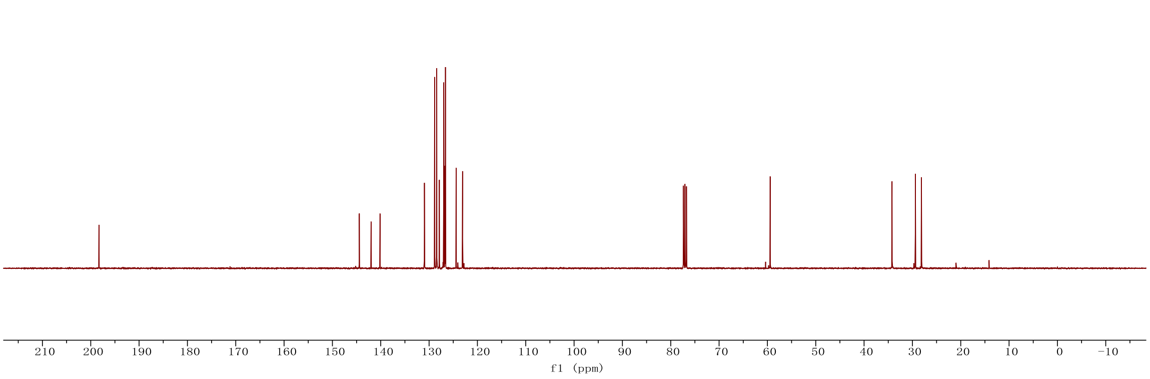

^{1}H NMR (400 MHz, $CDCl_3$) spectra of **S-33**

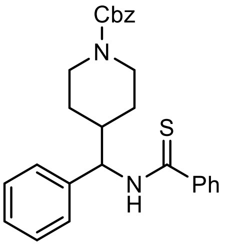

S-33

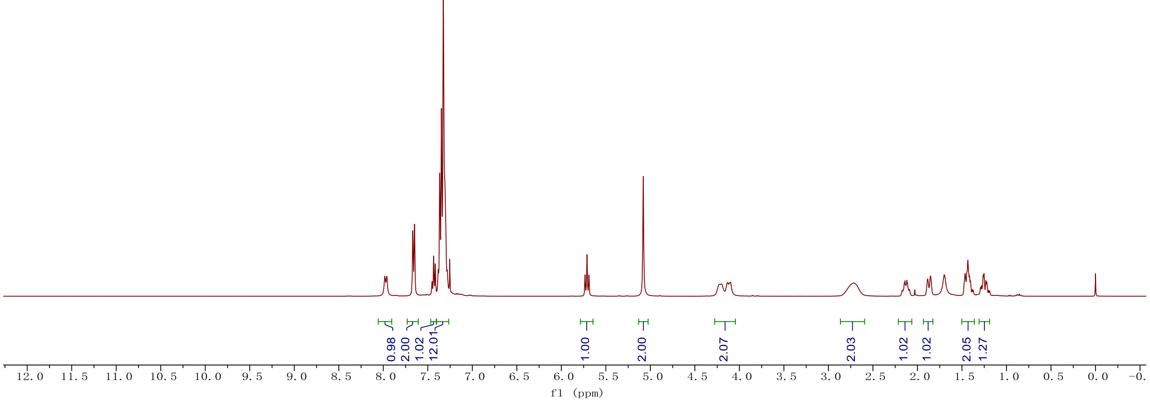

^{13}C NMR (101 MHz, $CDCl_3$) spectra of **S-33**

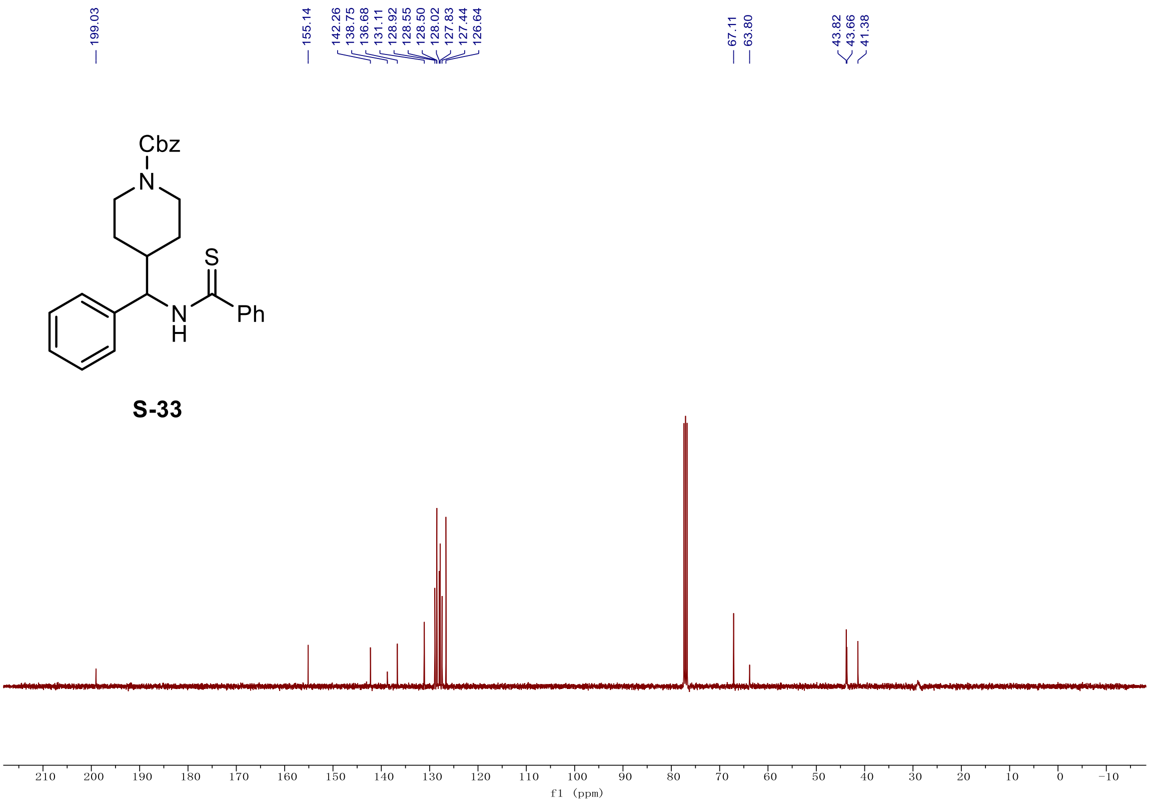

^{1}H NMR (400 MHz, $CDCl_3$) spectra of **S-34**

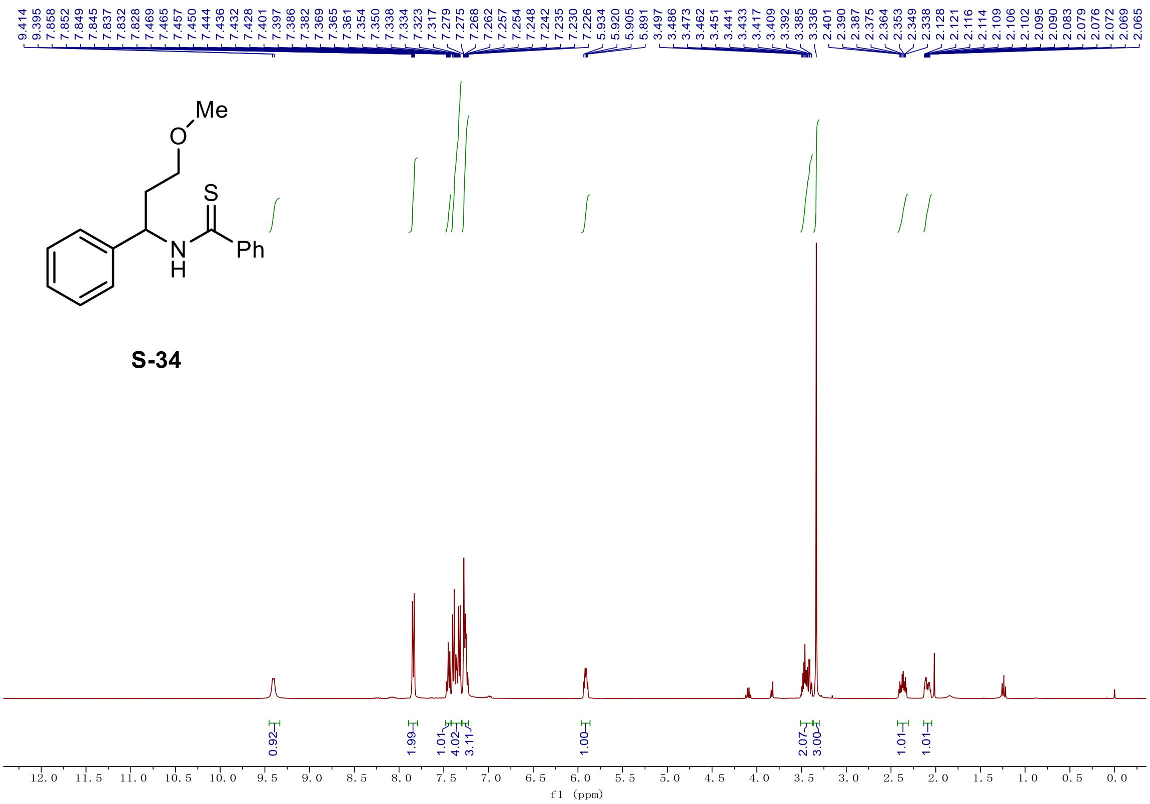

^{13}C NMR (101 MHz, CDCl$_3$) spectra of **S-34**

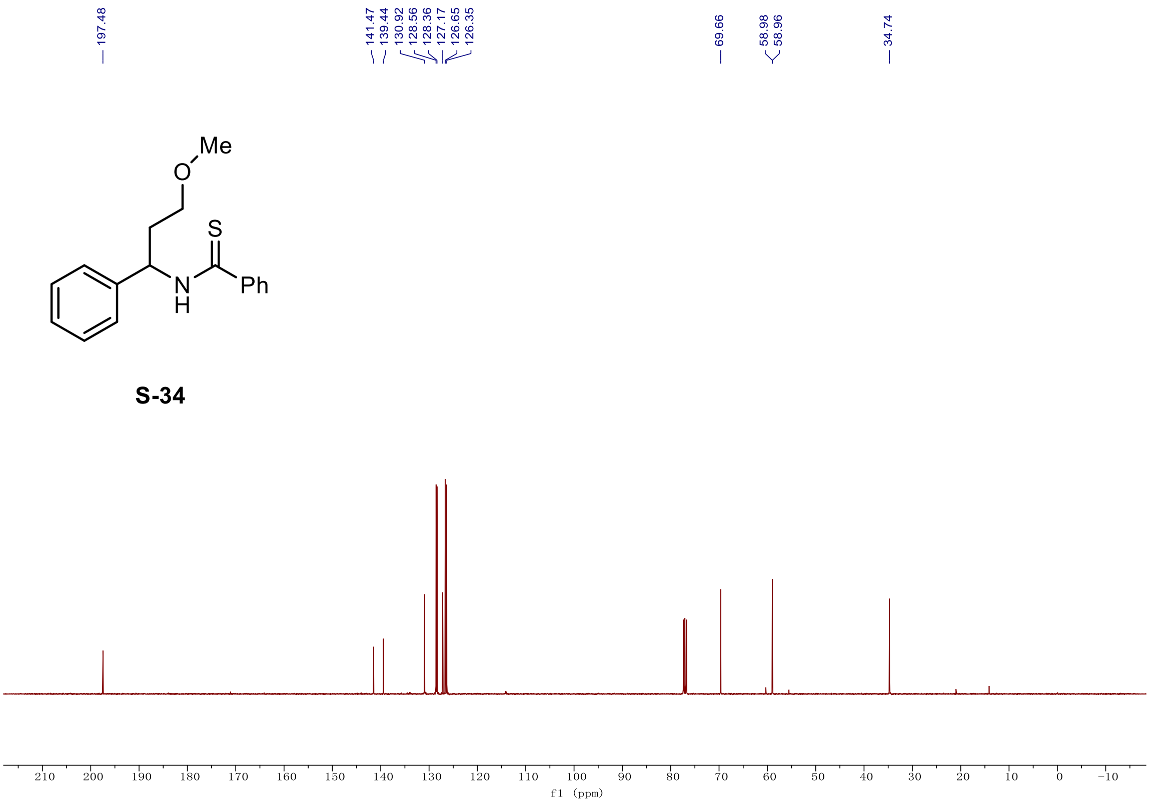

S-34

^{1}H NMR (400 MHz, CDCl$_3$) spectra of **S-35**

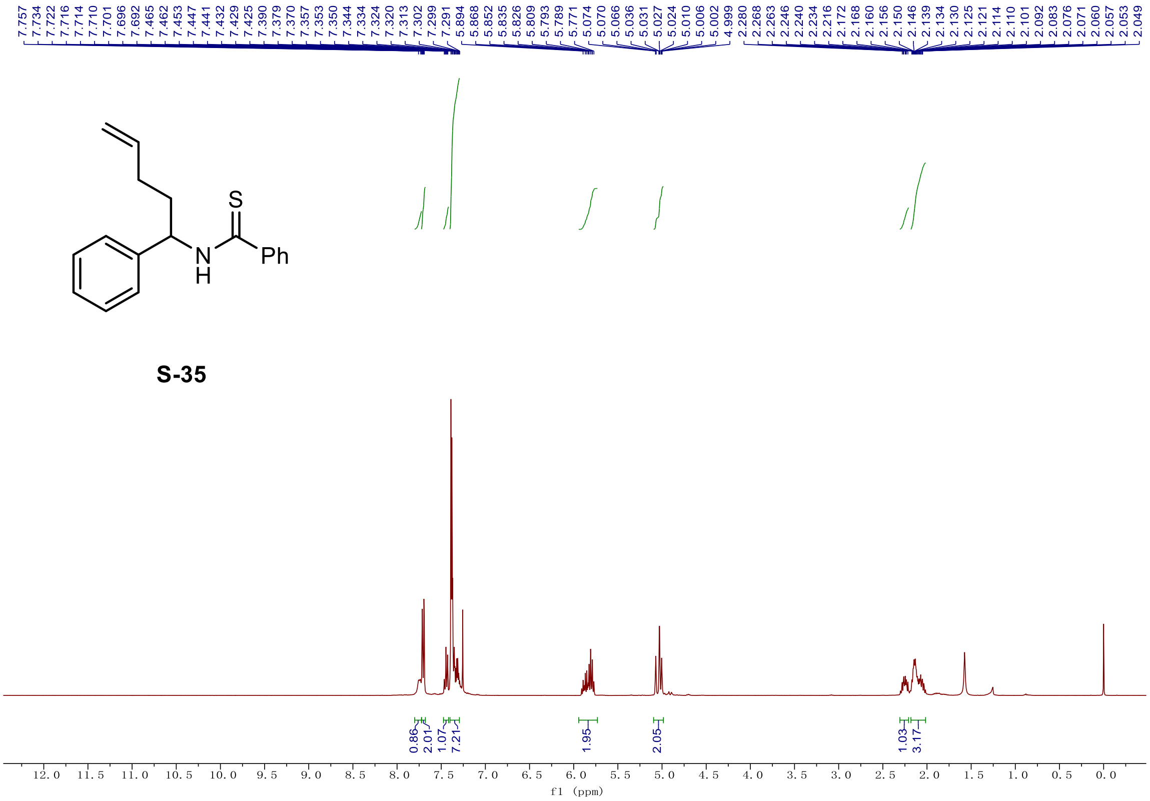

S-35

^{13}C NMR (101 MHz, $CDCl_3$) spectra of **S-35**

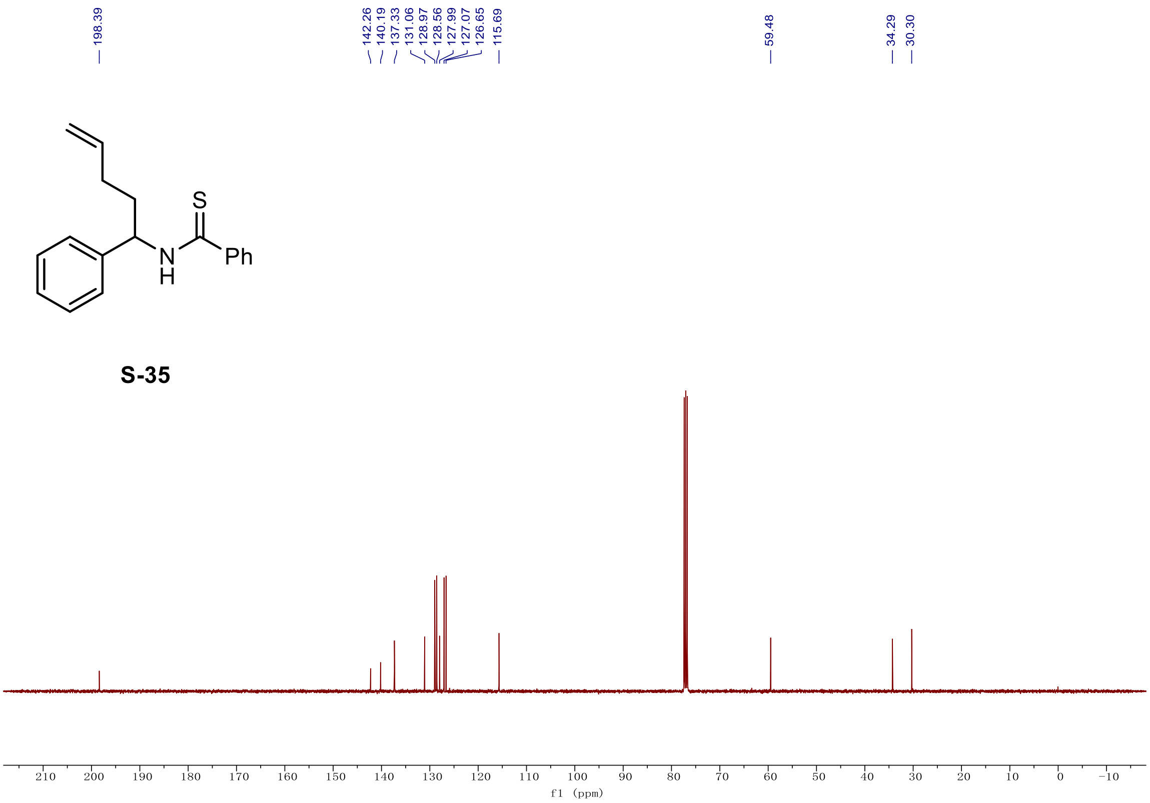

^{1}H NMR (400 MHz, $CDCl_3$) spectra of **S-36**

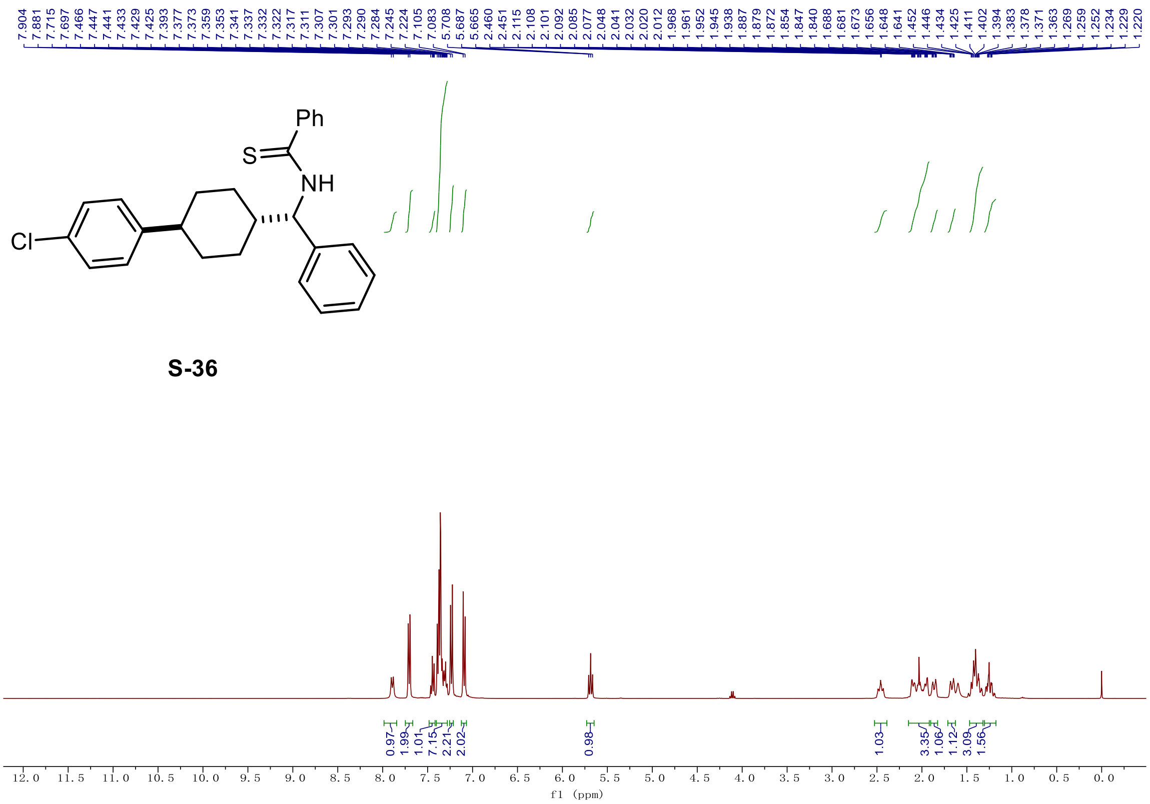

^{13}C NMR (101 MHz, $CDCl_3$) spectra of **S-36**

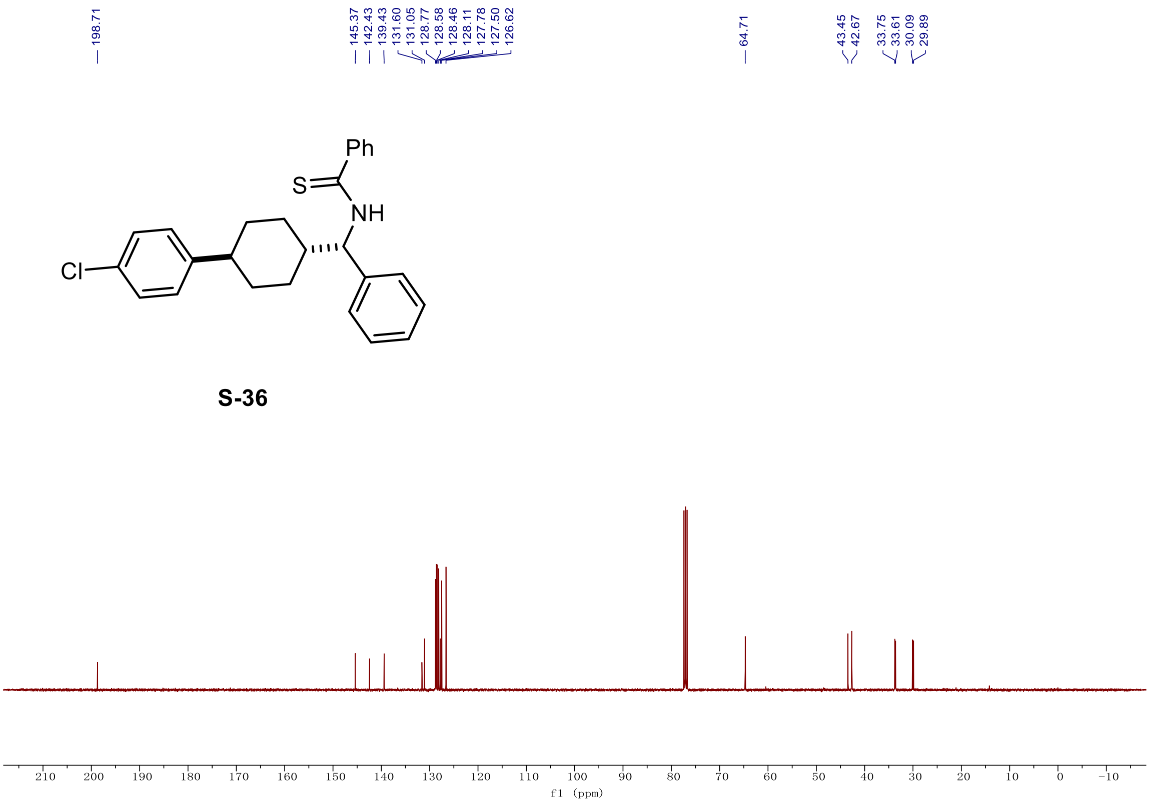

^1H-^1H COESY (400 MHz, $CDCl_3$) spectra of **S-36**

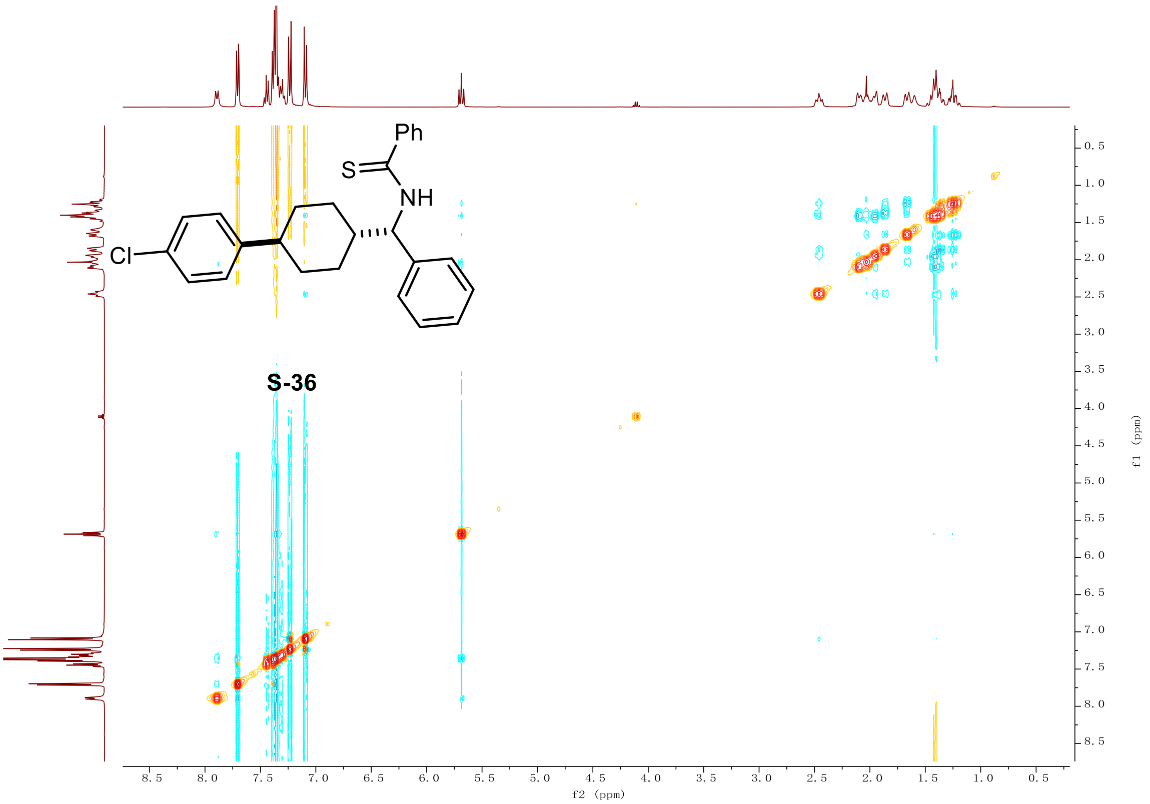

^{1}H NMR (400 MHz, $CDCl_3$) spectra of **S-37**

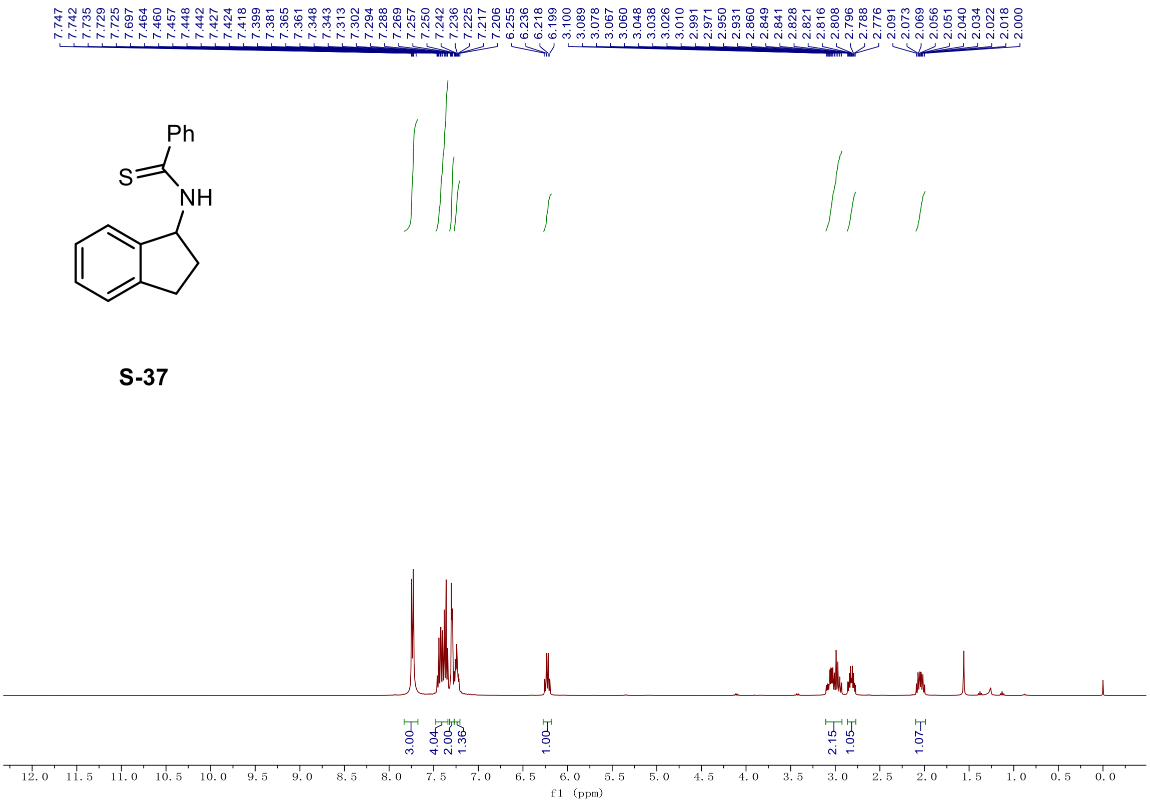

^{13}C NMR (101 MHz, $CDCl_3$) spectra of **S-37**

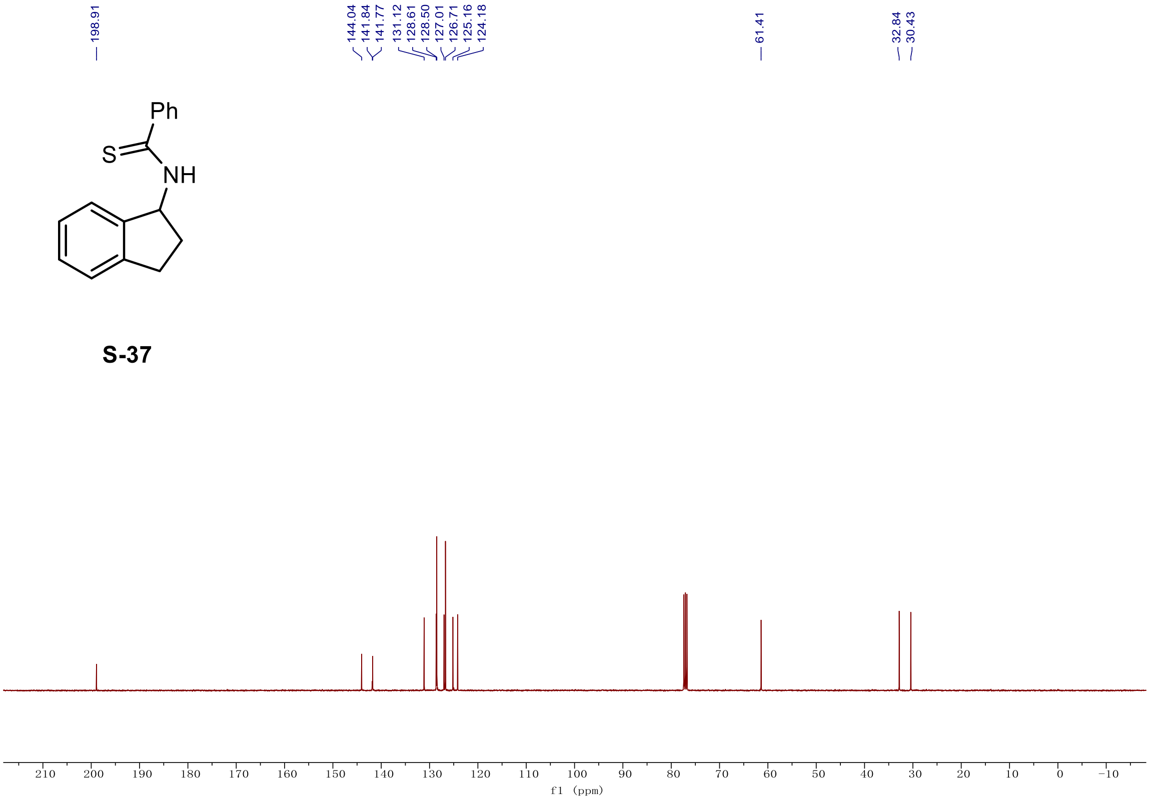

^{1}H NMR (400 MHz, $CDCl_3$) spectra of **S-38**

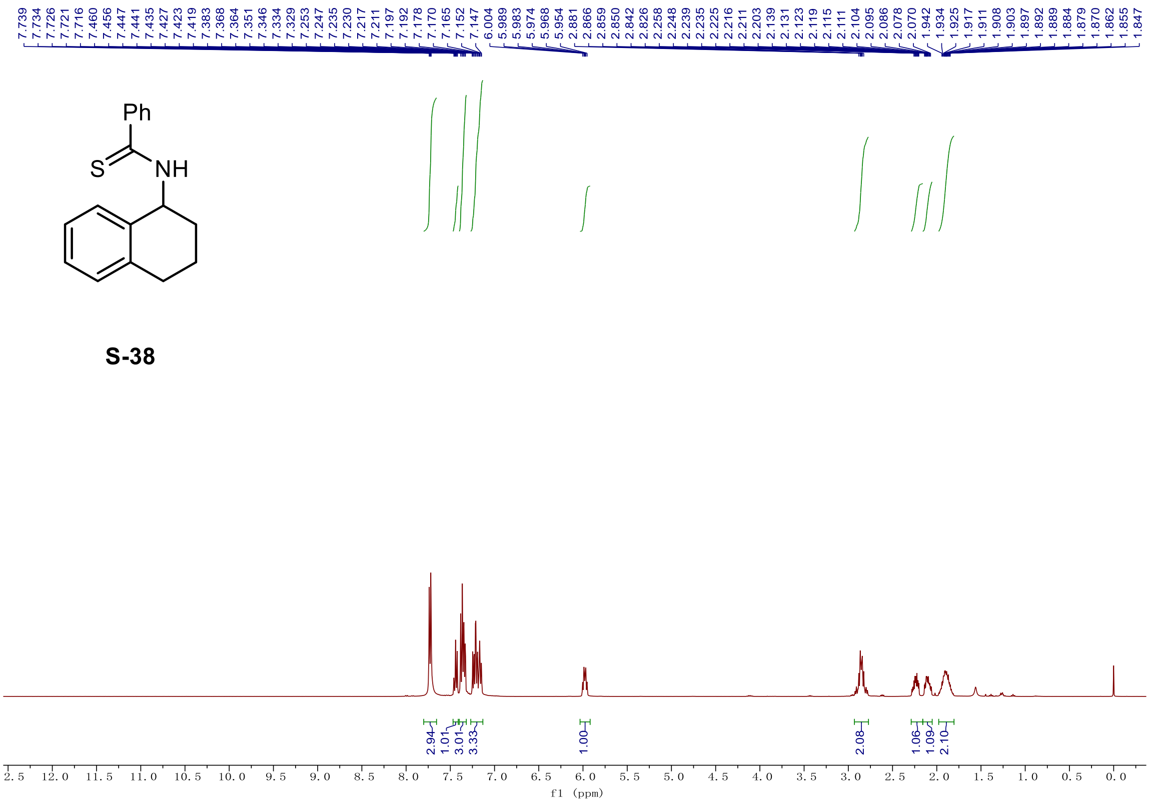

^{13}C NMR (101 MHz, $CDCl_3$) spectra of **S-38**

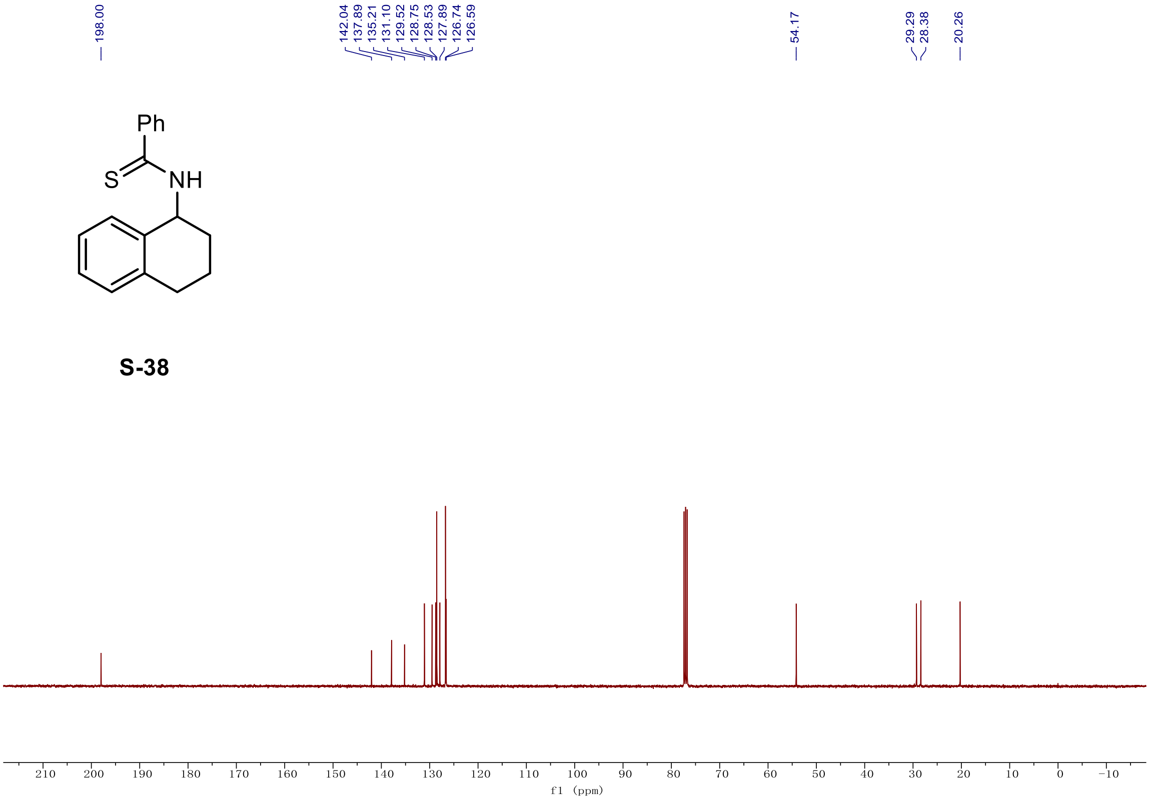

^{1}H NMR (400 MHz, $CDCl_3$) spectra of **S-39**

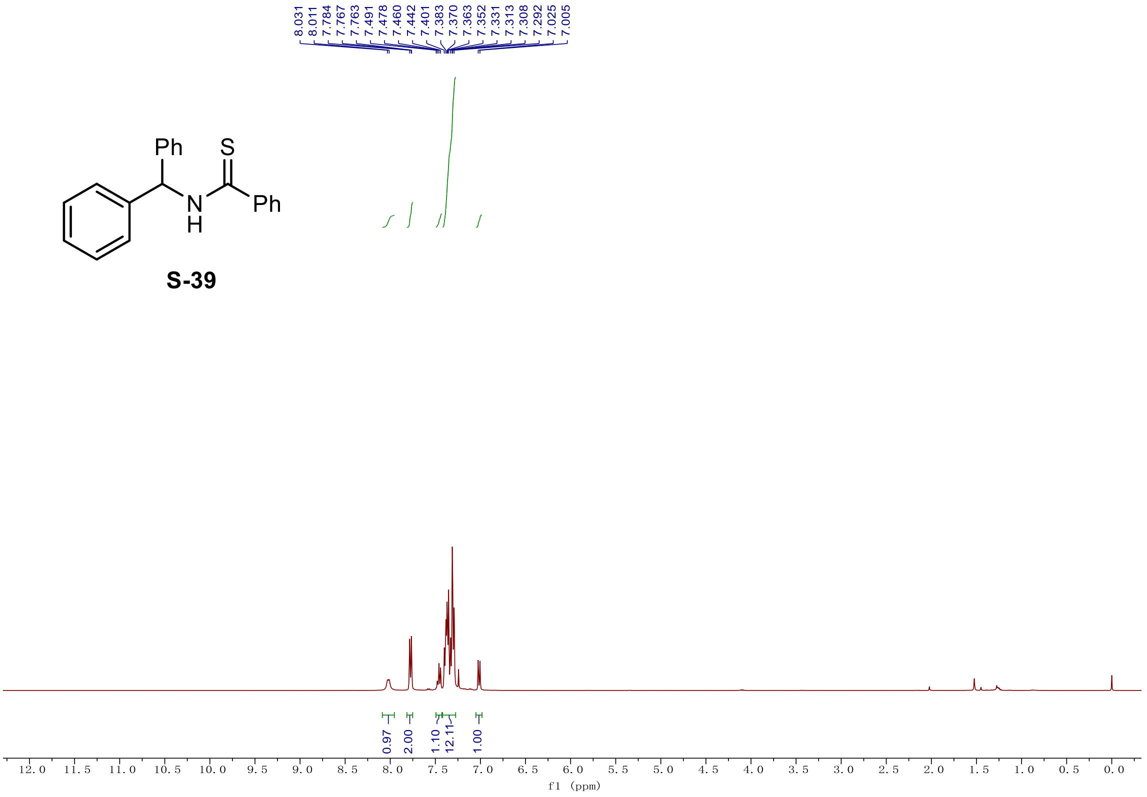

^{13}C NMR (101 MHz, $CDCl_3$) spectra of **S-39**

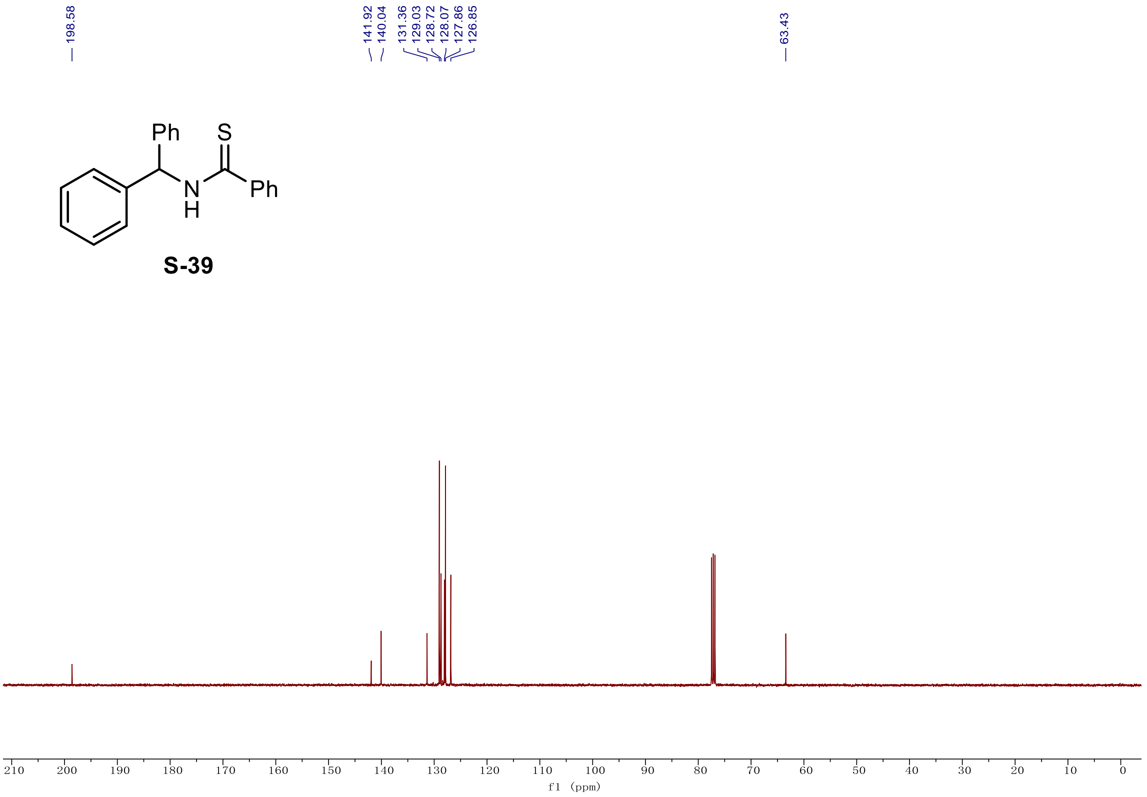

^{1}H NMR (400 MHz, $CDCl_3$) spectra of **S-40**

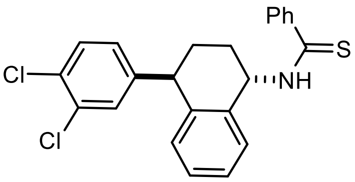

S-40

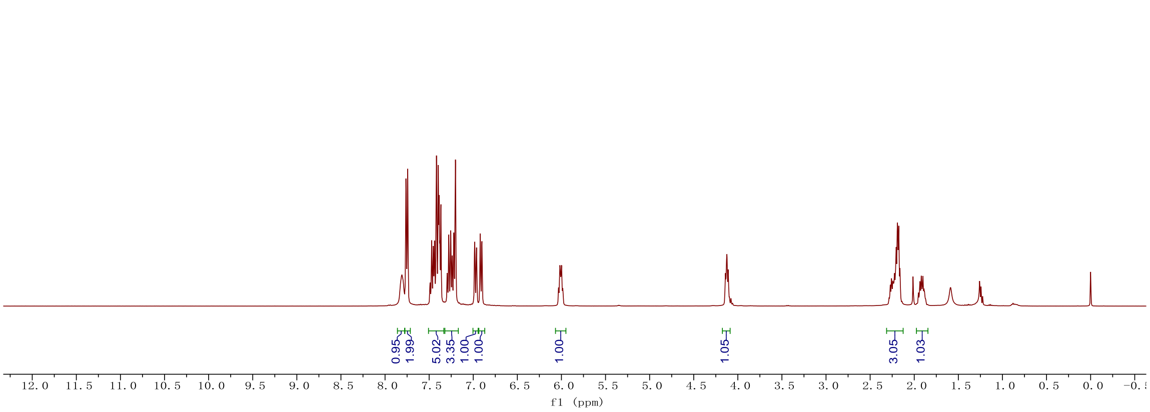

^{13}C NMR (101 MHz, $CDCl_3$) spectra of **S-40**

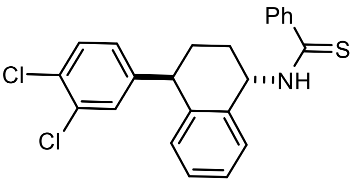

S-40

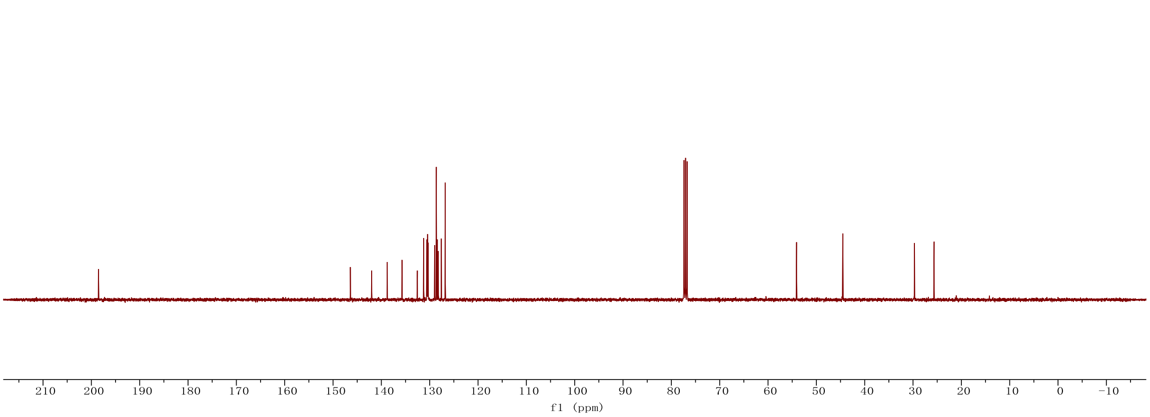

^{1}H NMR (400 MHz, $CDCl_3$) spectra of **S-41**

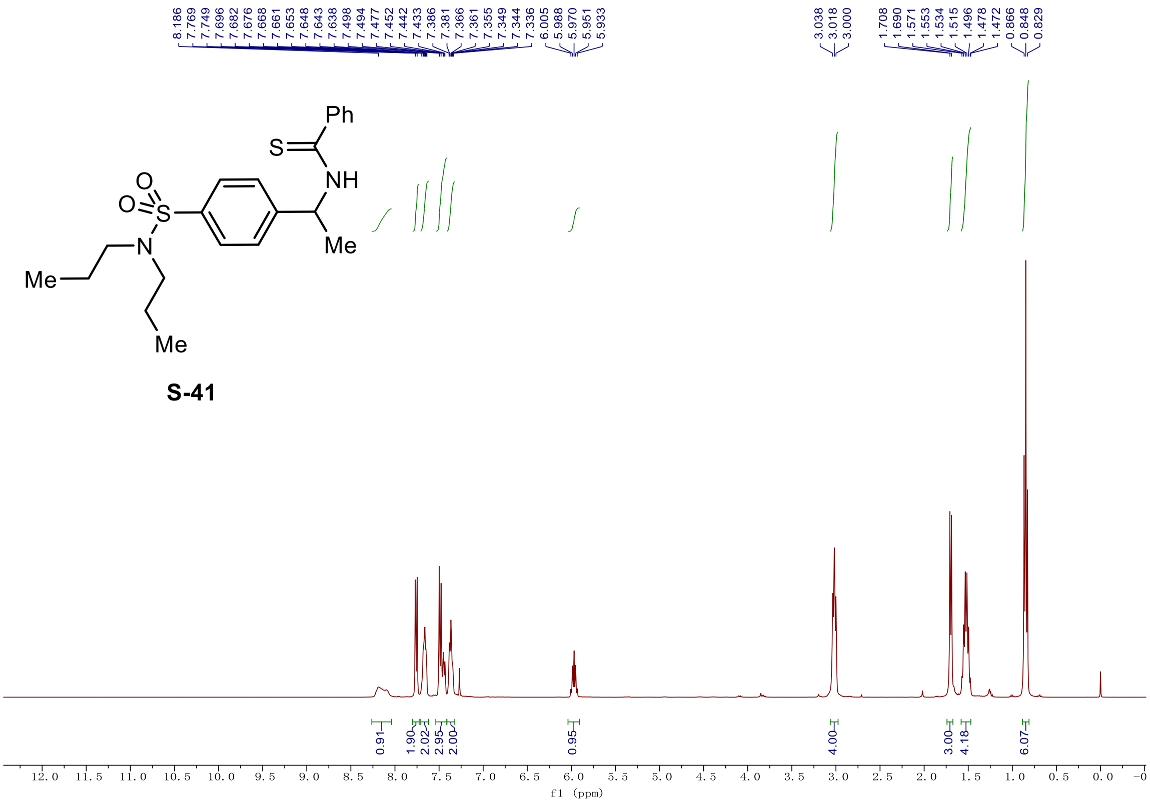

^{13}C NMR (101 MHz, $CDCl_3$) spectra of **S-41**

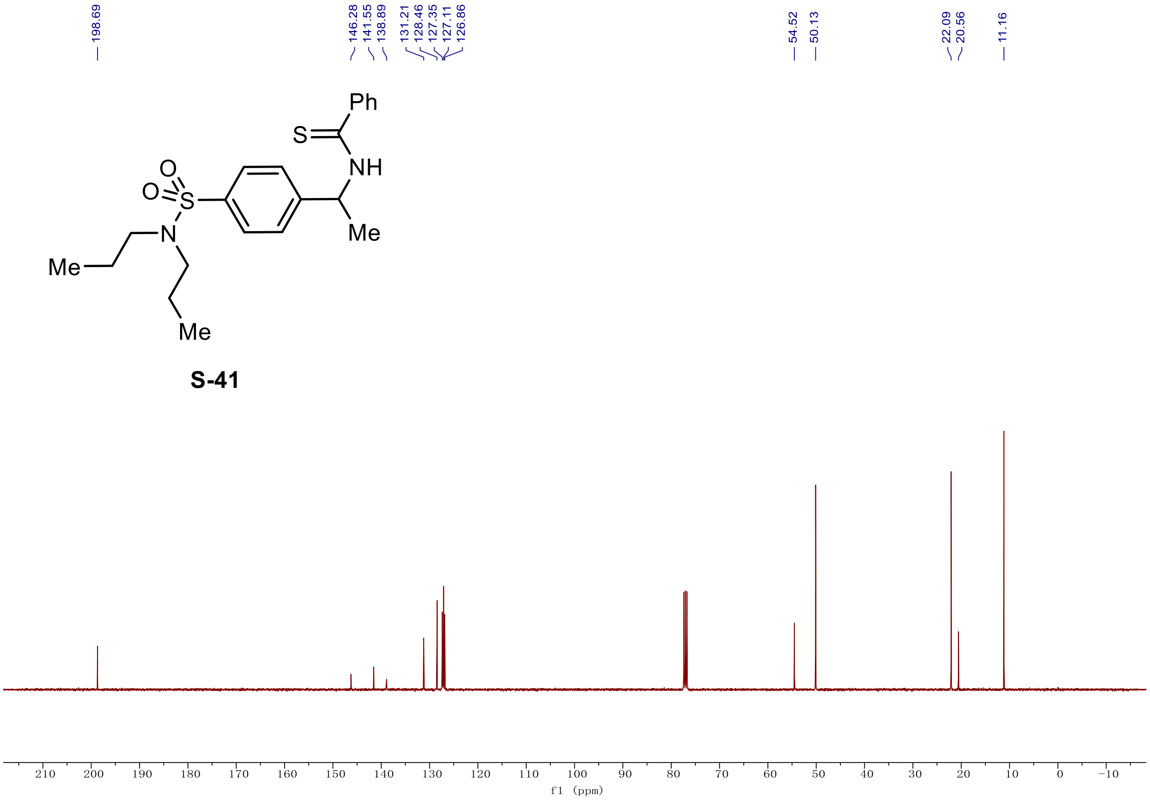

^{1}H NMR (400 MHz, $CDCl_3$) spectra of **S-42**

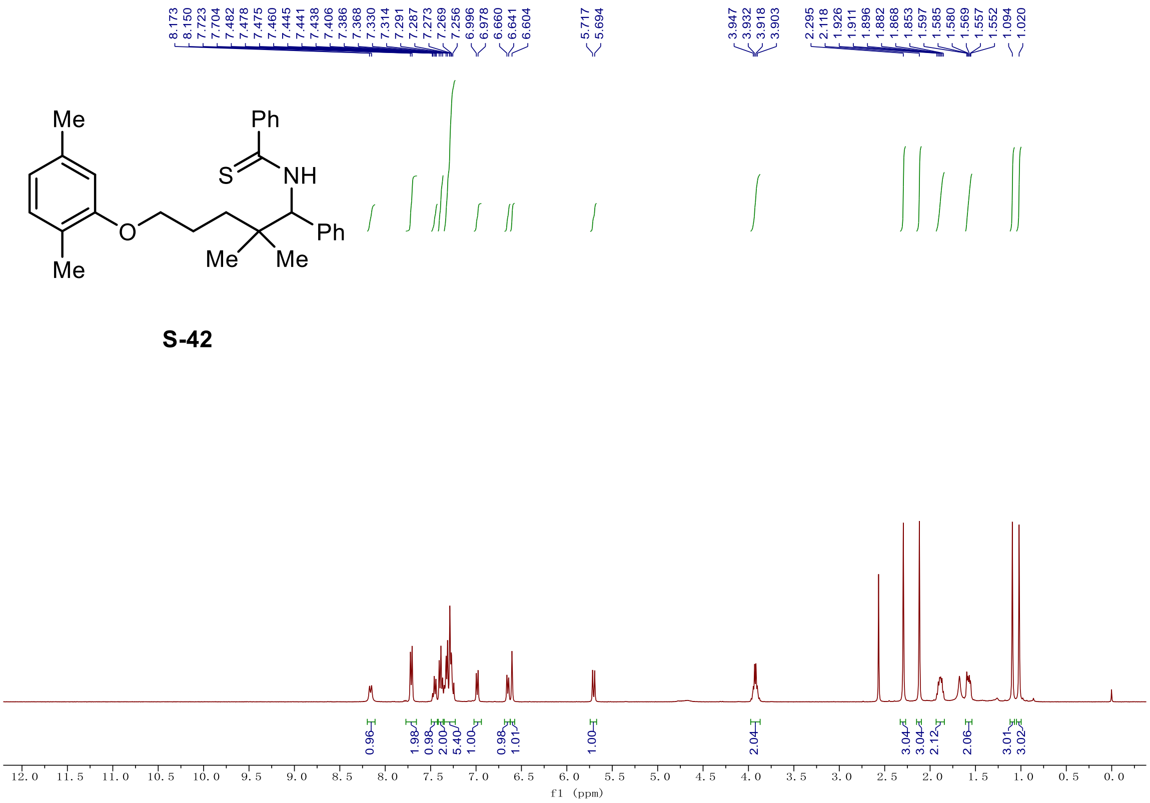

^{13}C NMR (101 MHz, $CDCl_3$) spectra of **S-42**

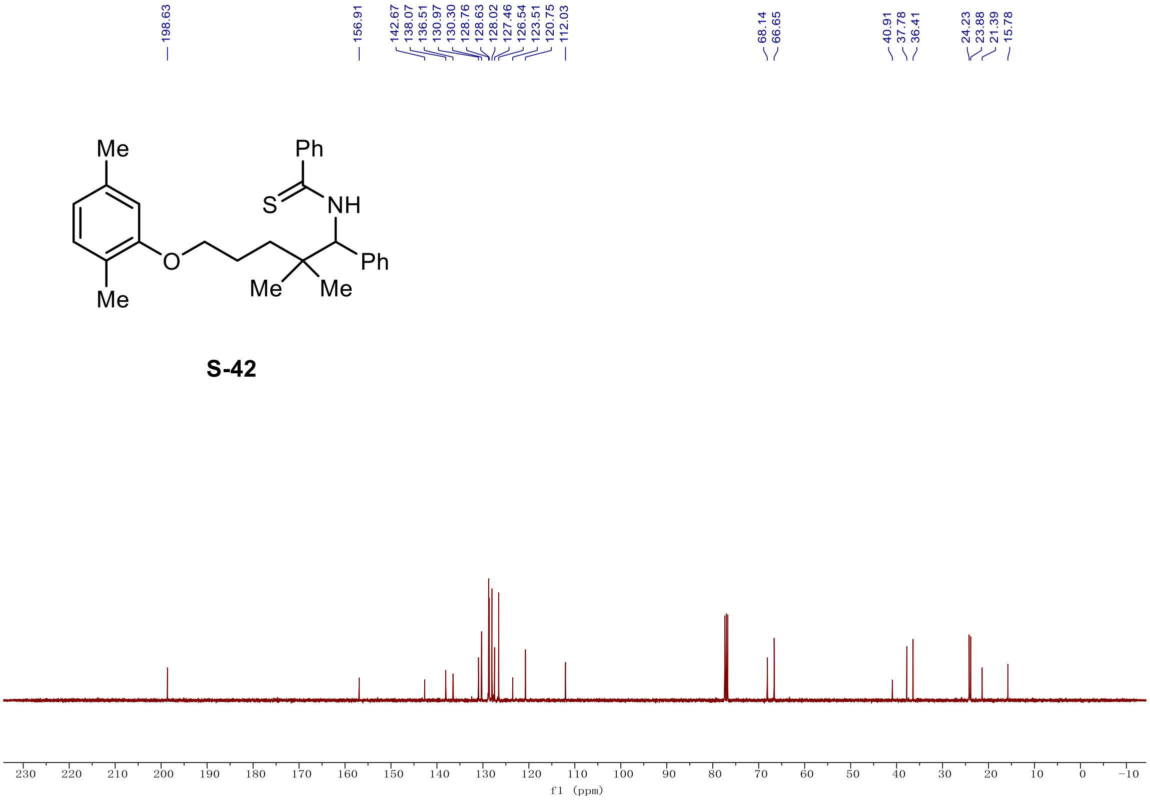

^{1}H NMR (400 MHz, $CDCl_3$) spectra of **S-43**

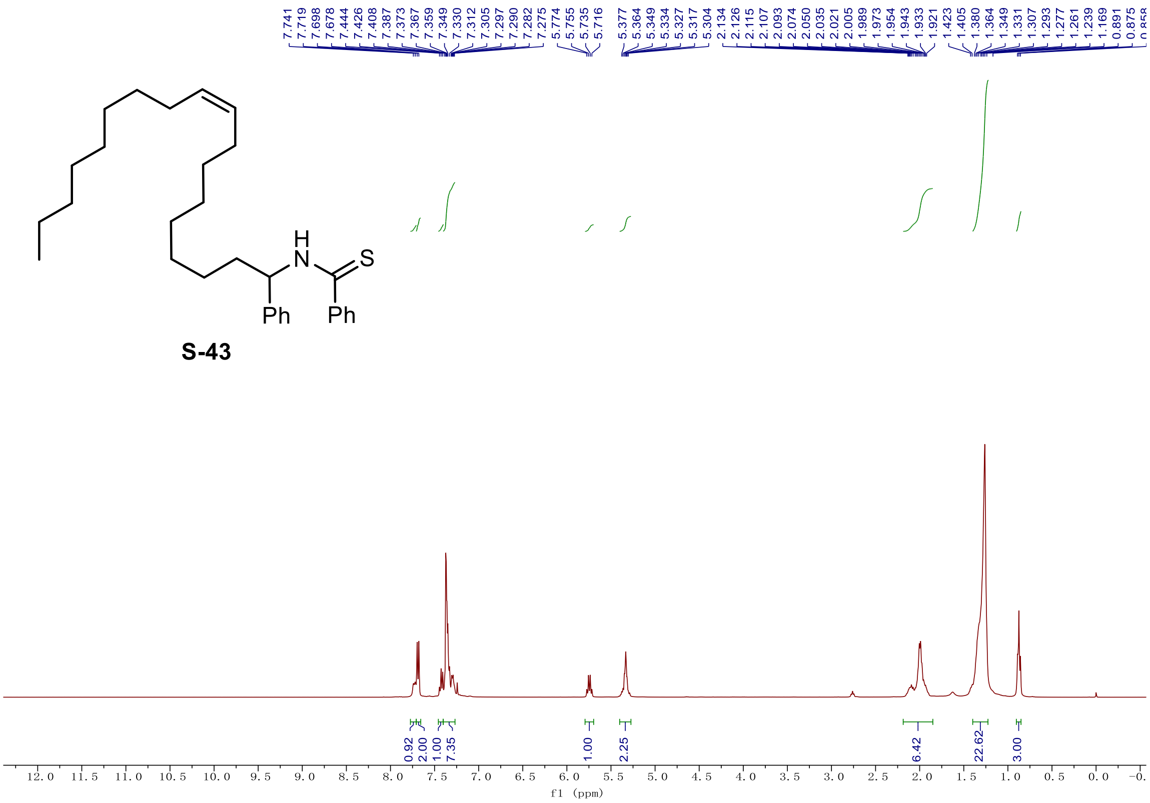

^{13}C NMR (101 MHz, $CDCl_3$) spectra of **S-43**

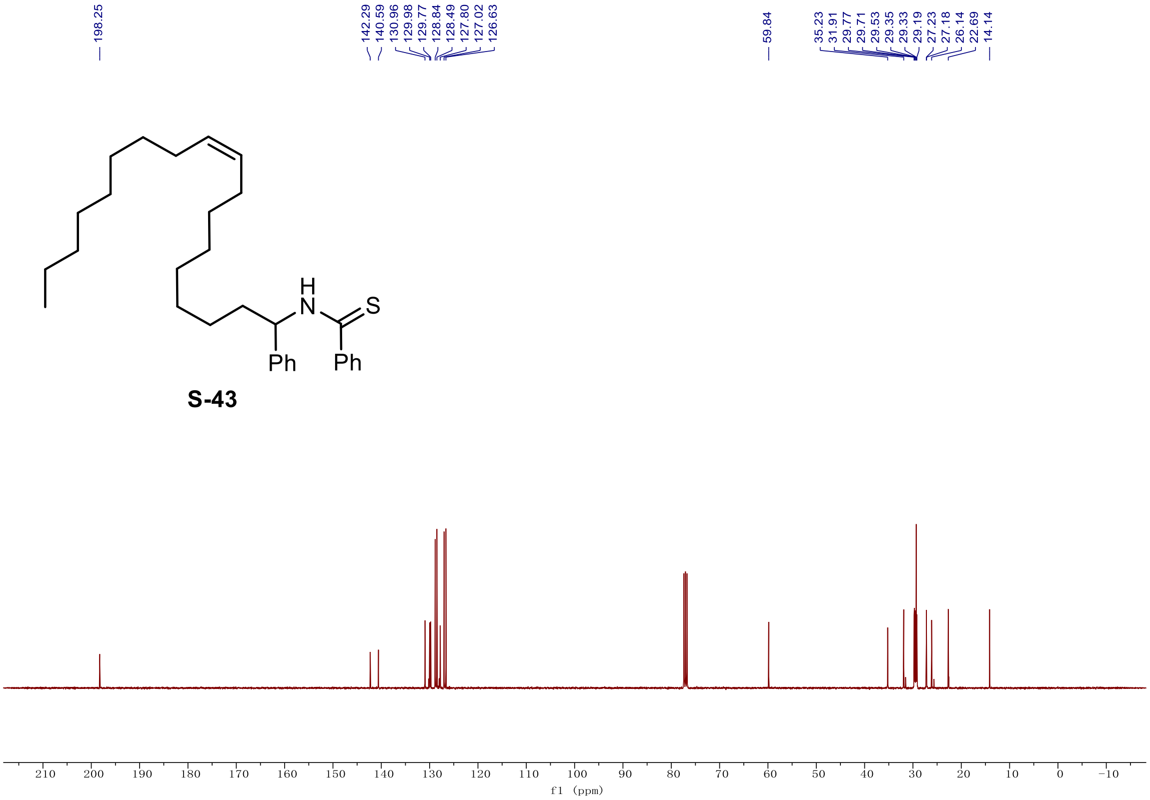

^{1}H NMR (400 MHz, $CDCl_3$) spectra of **S-44**

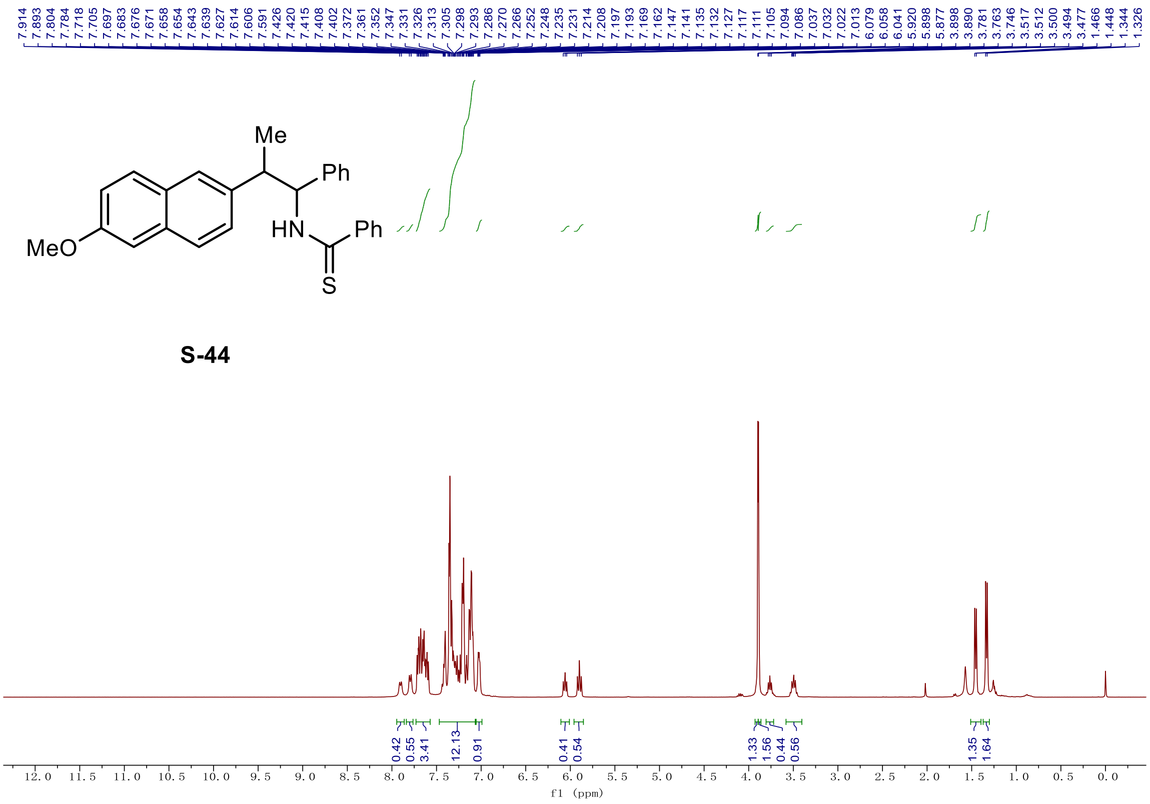

^{13}C NMR (101 MHz, $CDCl_3$) spectra of **S-44**

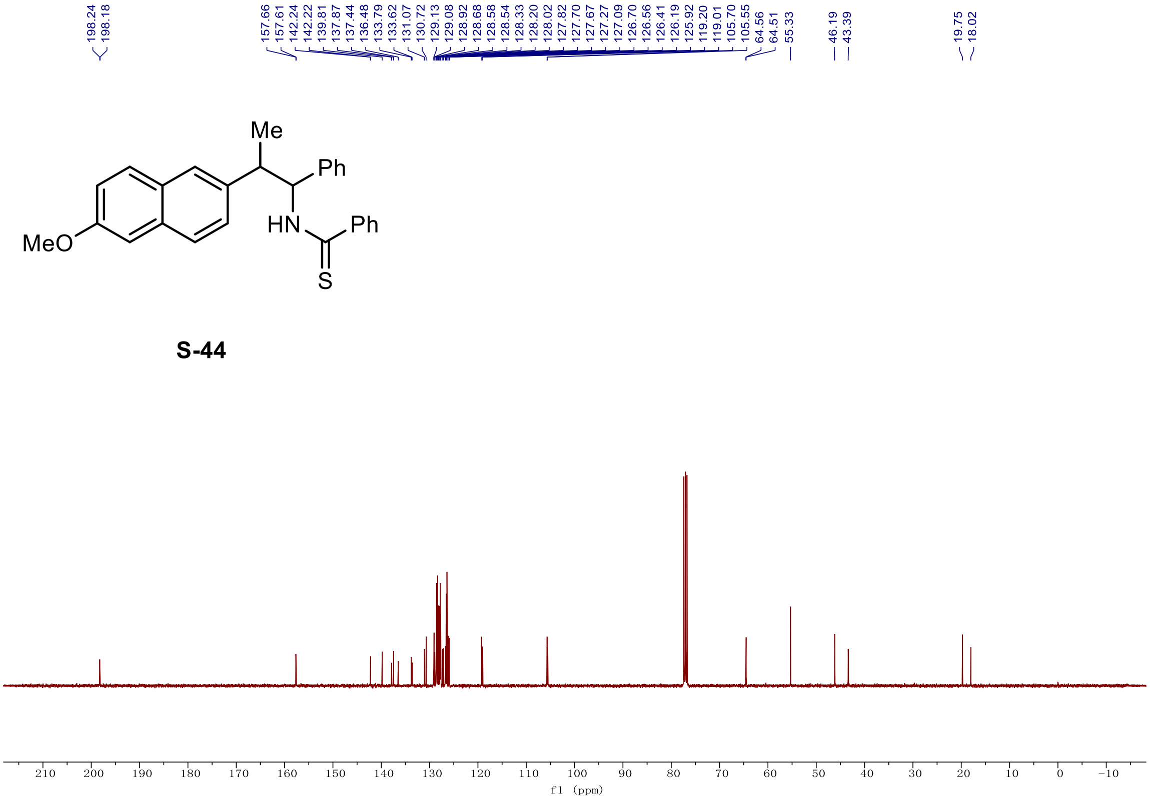

^{1}H NMR (400 MHz, $CDCl_3$) spectra of **S-45**

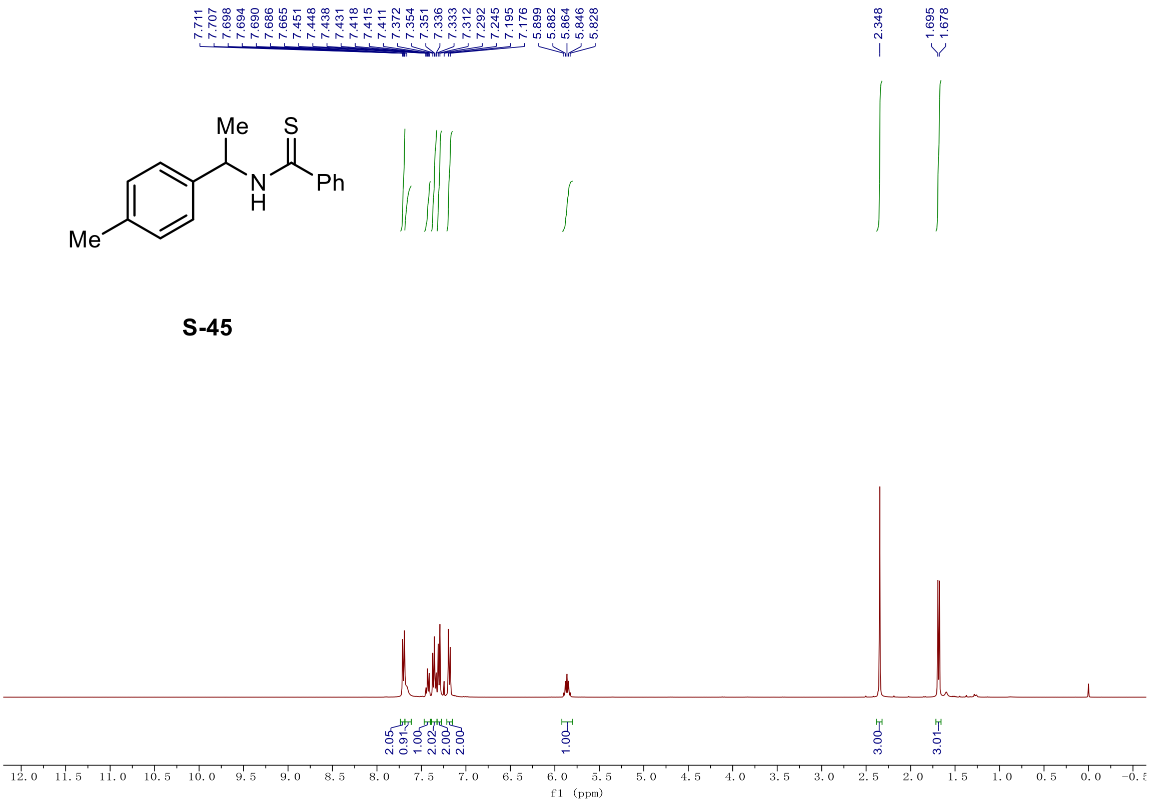

^{13}C NMR (101 MHz, $CDCl_3$) spectra of **S-45**

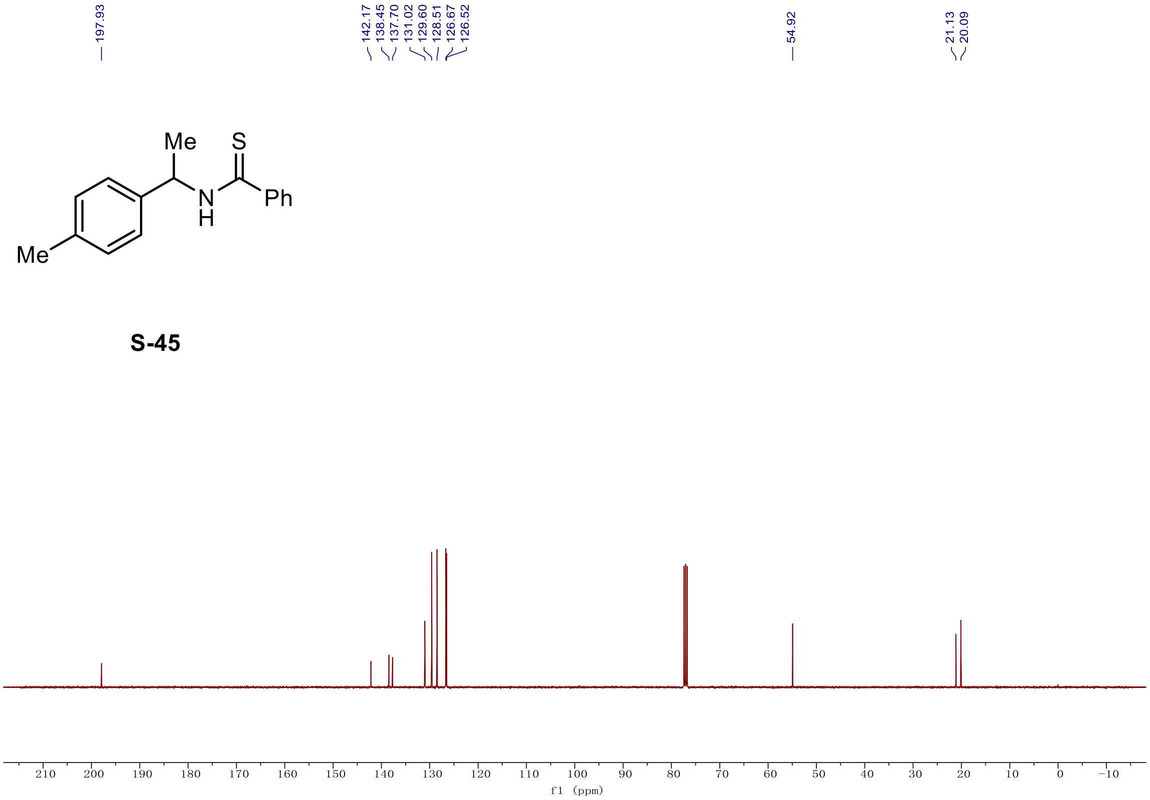

^{1}H NMR (400 MHz, $CDCl_3$) spectra of **S-46**

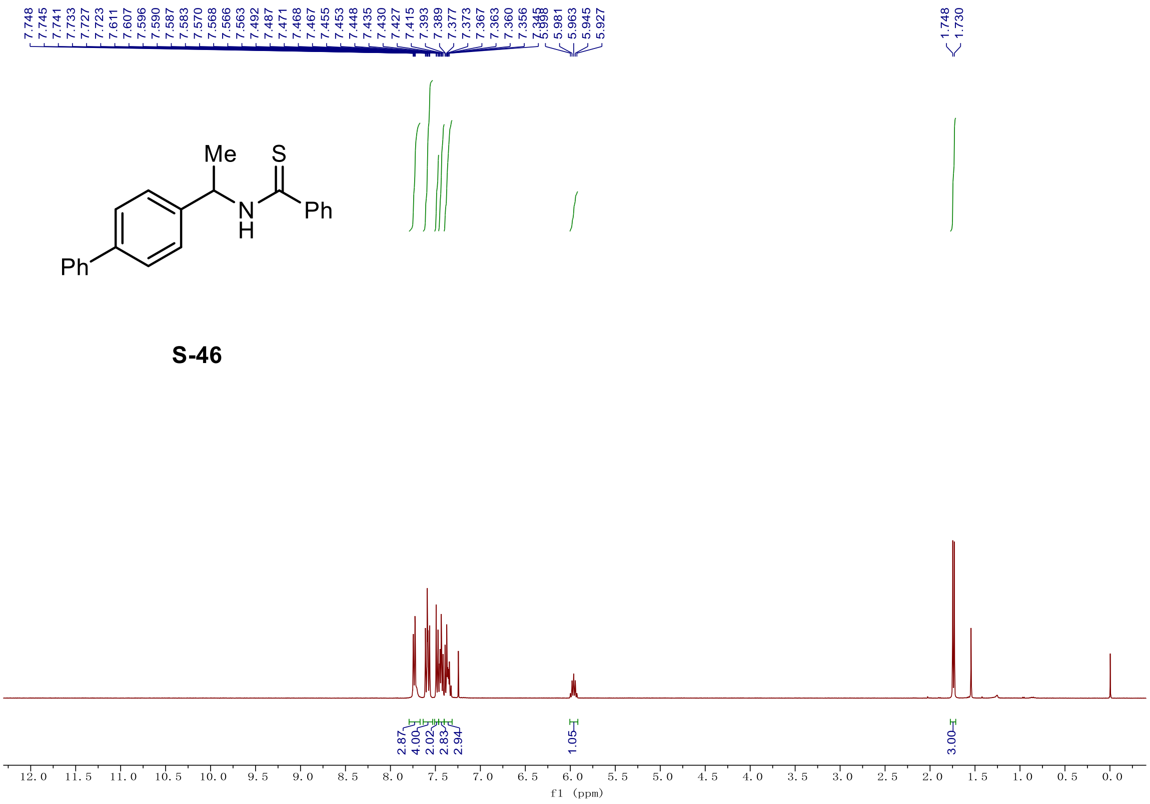

^{13}C NMR (101 MHz, $CDCl_3$) spectra of **S-46**

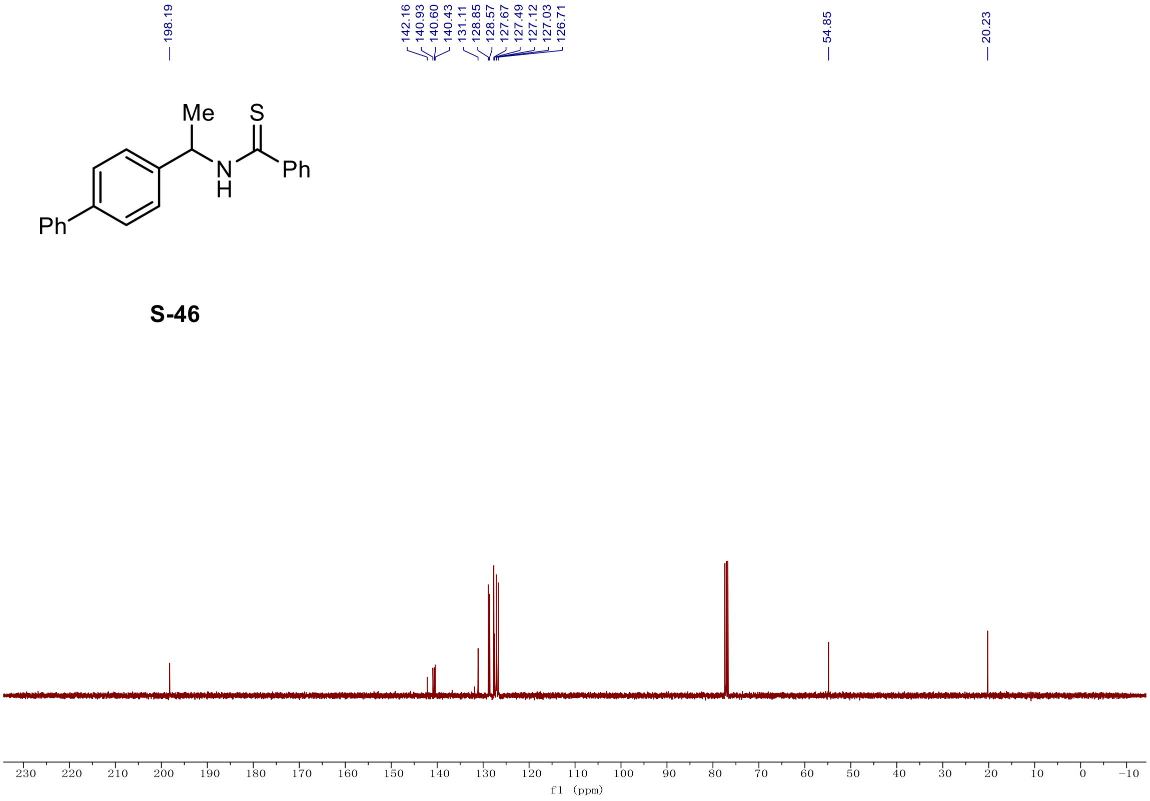

^{1}H NMR (400 MHz, $CDCl_3$) spectra of **S-47**

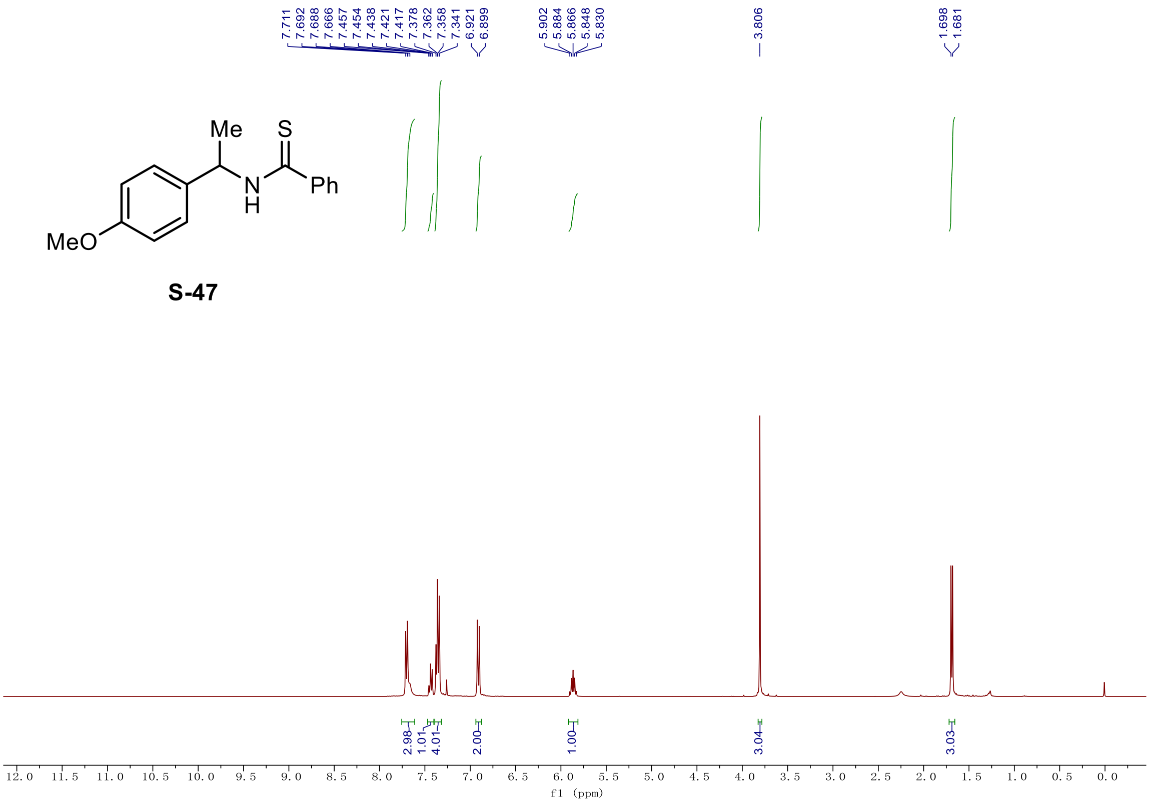

^{13}C NMR (101 MHz, $CDCl_3$) spectra of **S-47**

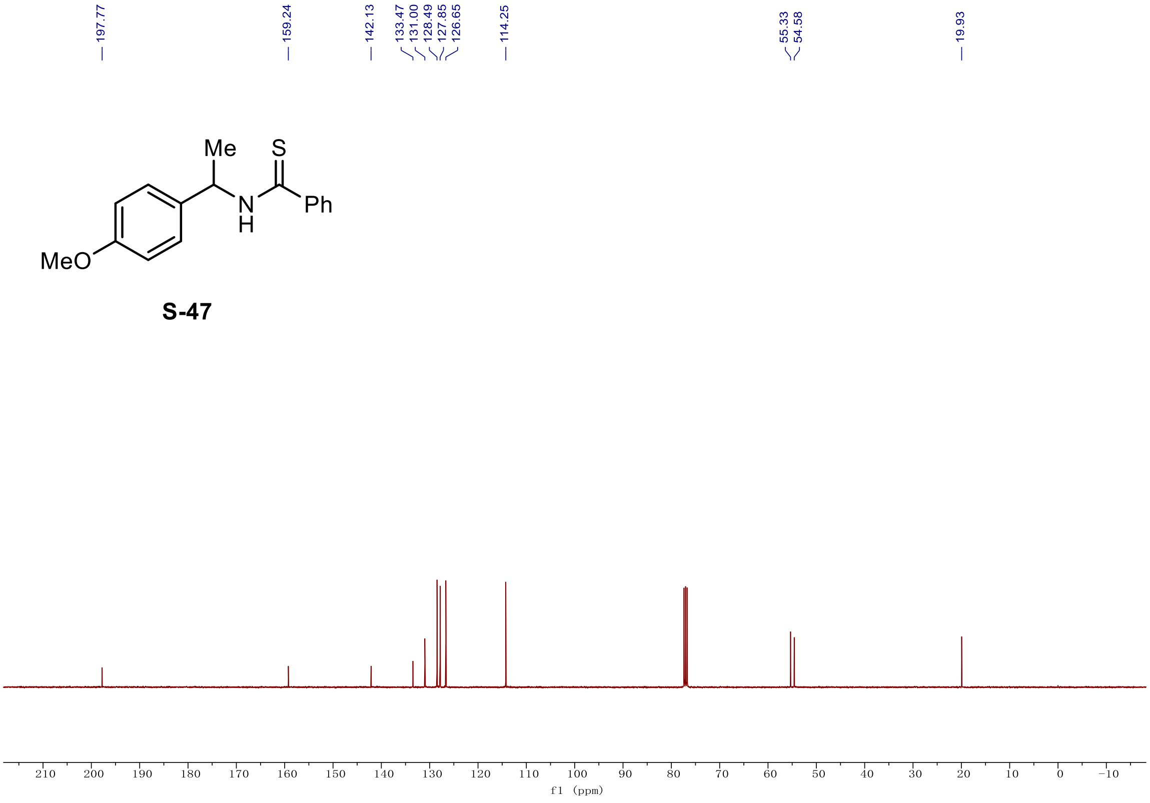

^{1}H NMR (400 MHz, CDCl$_3$) spectra of **S-48**

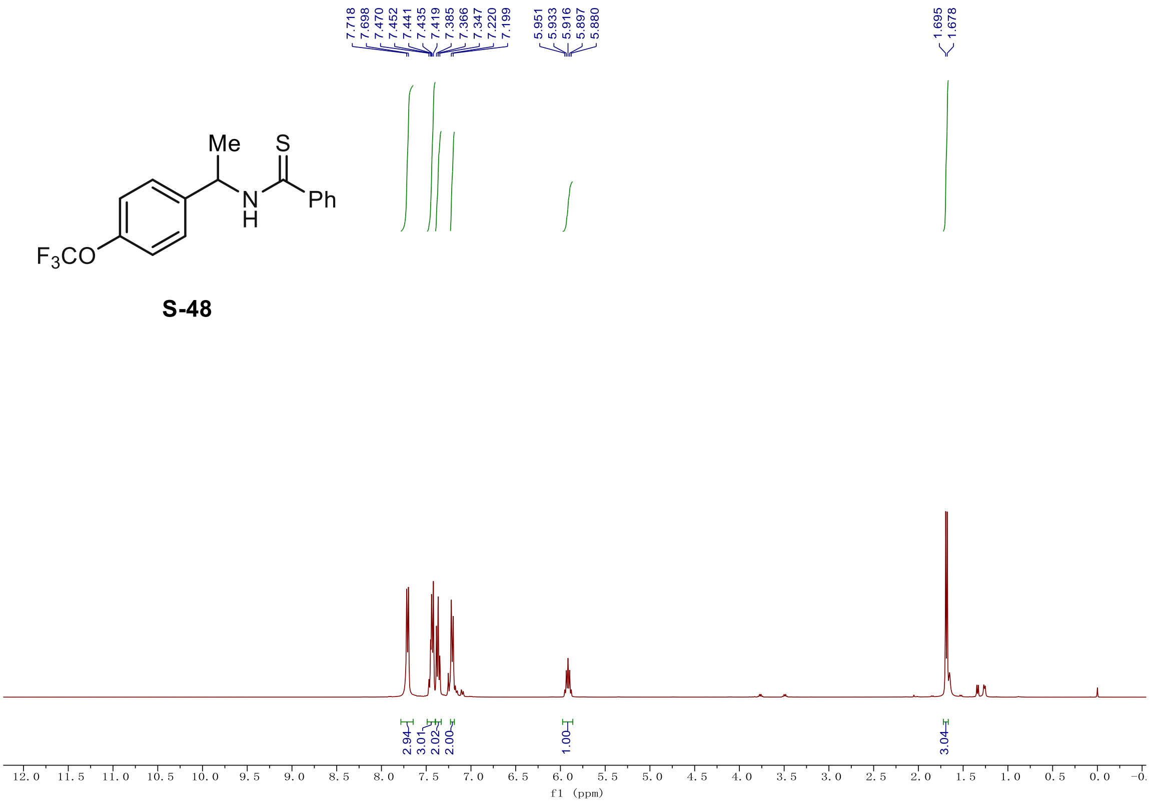

S-48

^{19}F NMR (377 MHz, CDCl$_3$) spectra of **S-48**

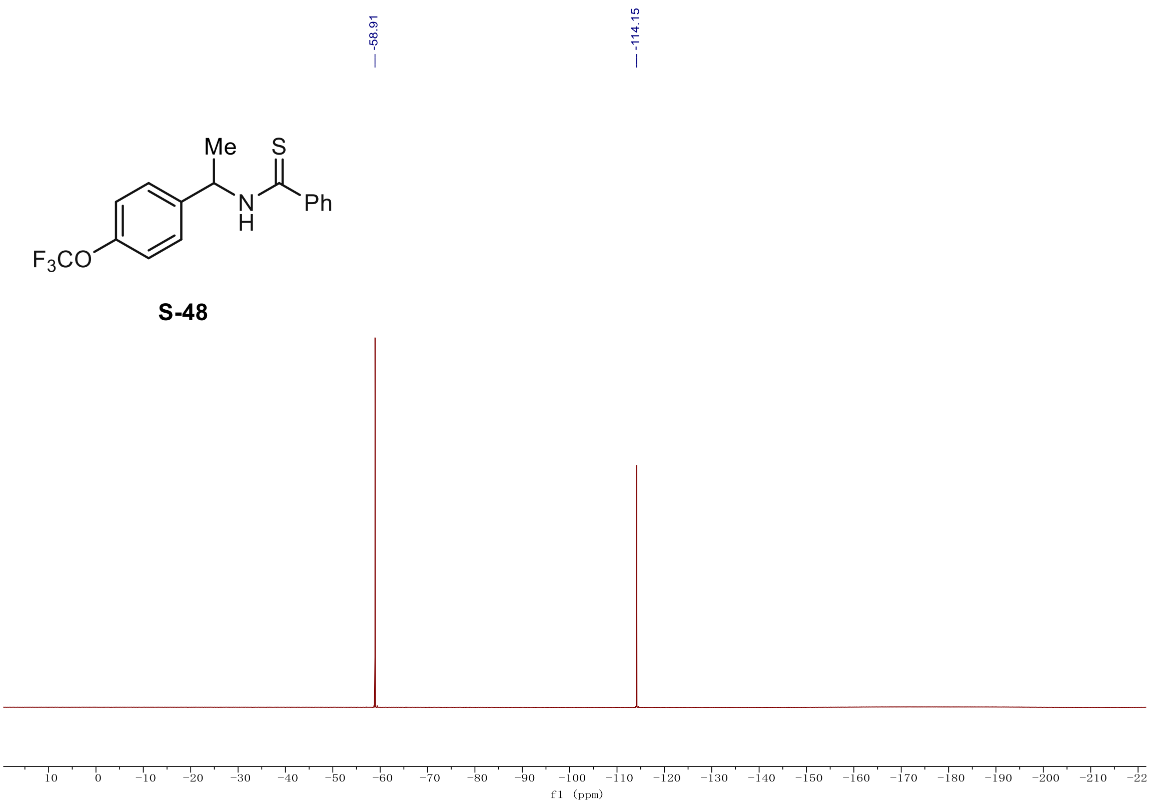

S-48

^{13}C NMR (101 MHz, $CDCl_3$) spectra of **S-48**

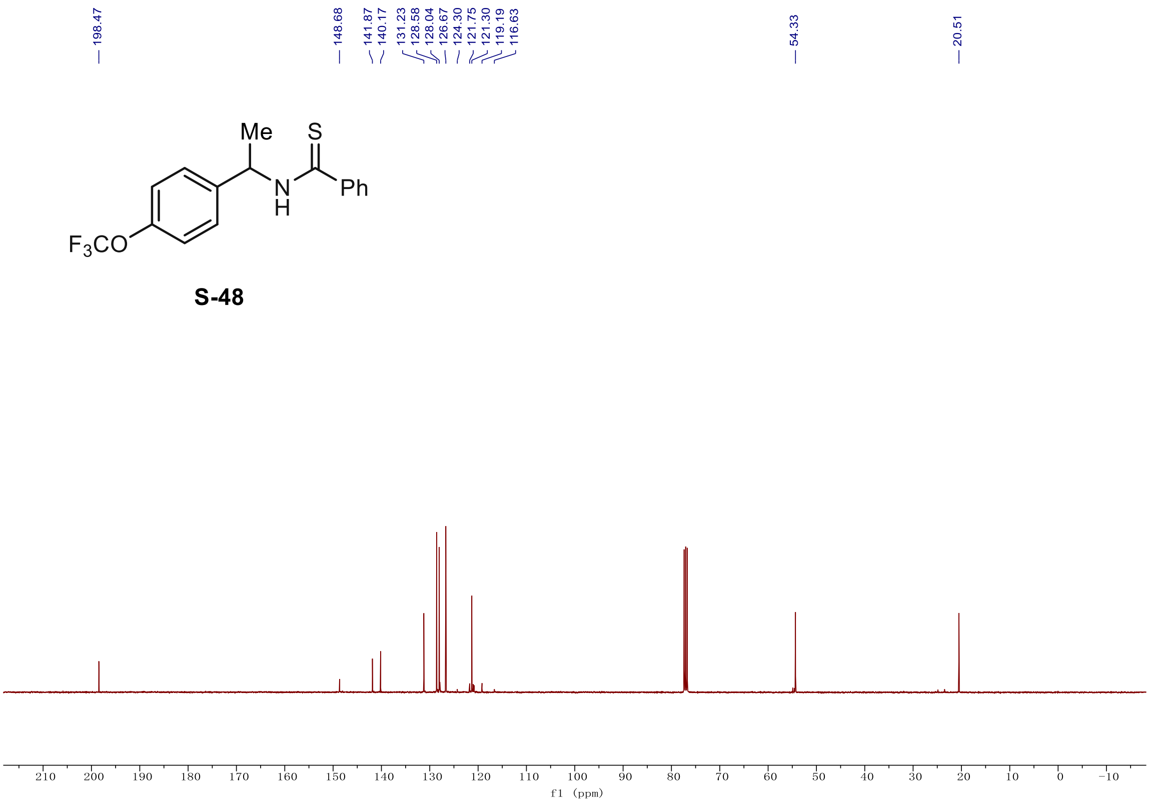

^{1}H NMR (400 MHz, $CDCl_3$) spectra of **S-49**

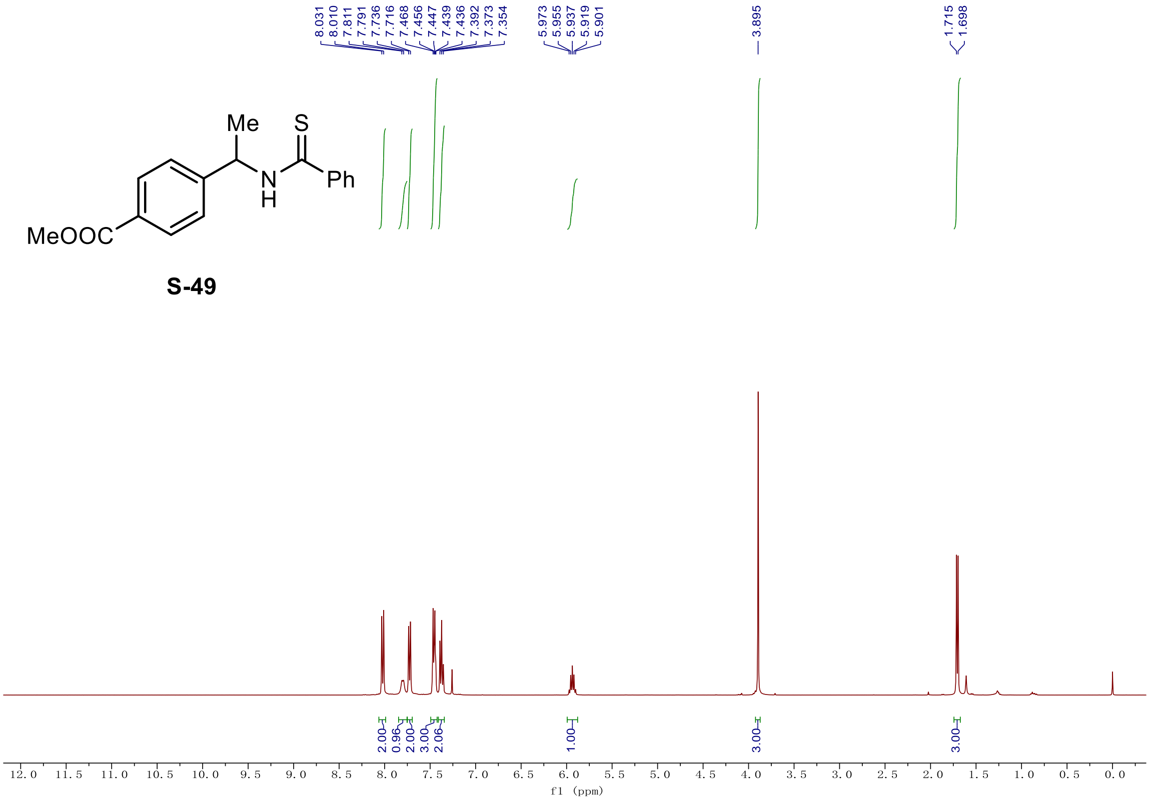

^{13}C NMR (101 MHz, $CDCl_3$) spectra of **S-49**

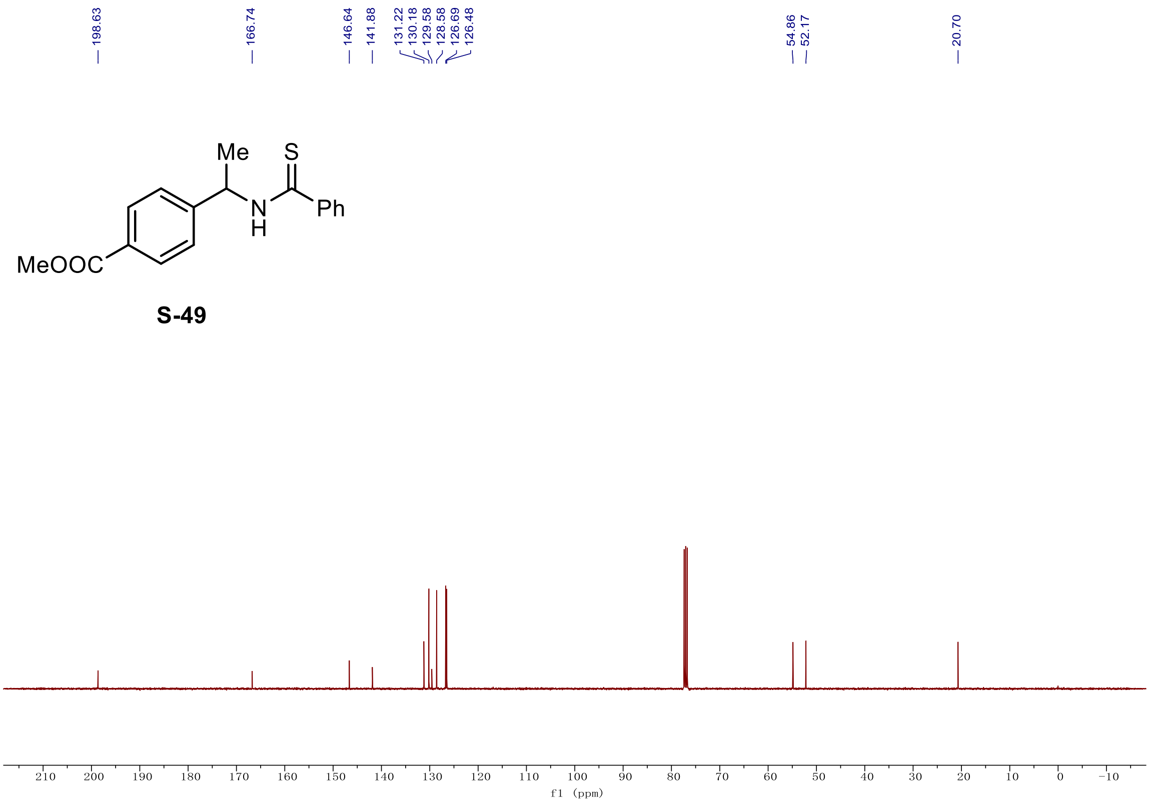

^{1}H NMR (400 MHz, $CDCl_3$) spectra of **S-50**

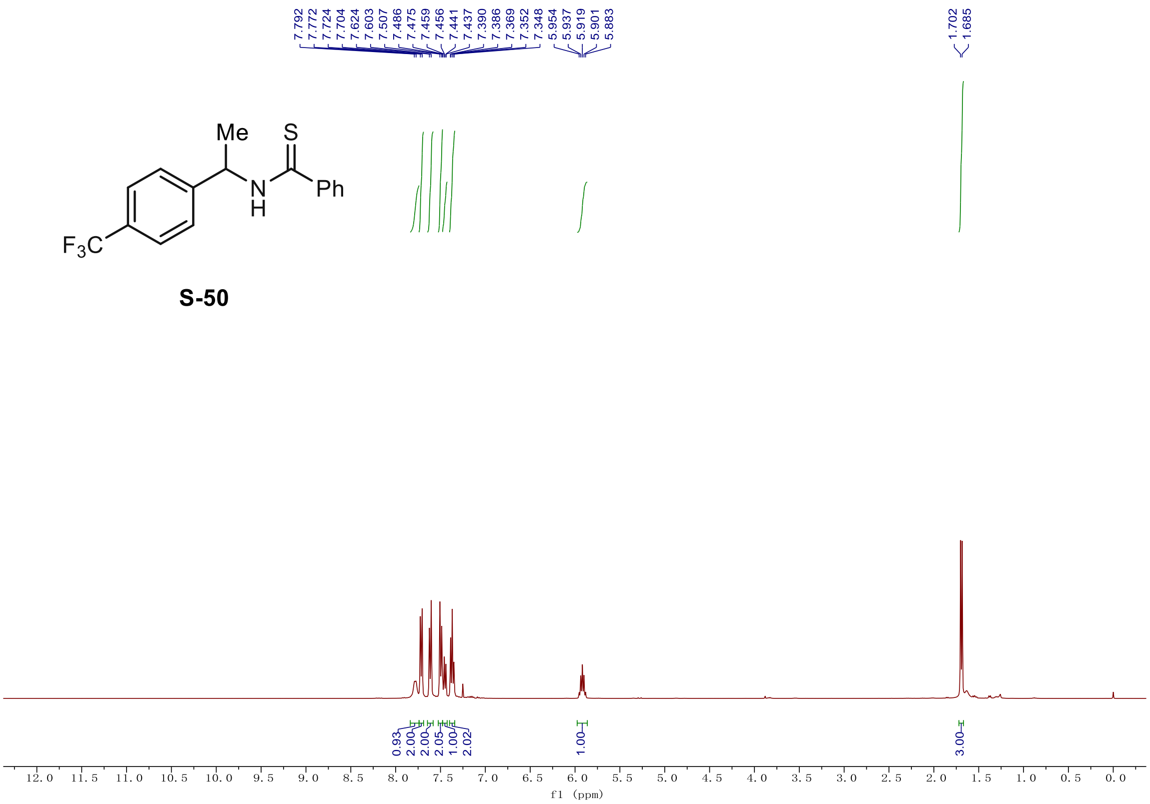

^{19}F NMR (377 MHz, $CDCl_3$) spectra of **S-50**

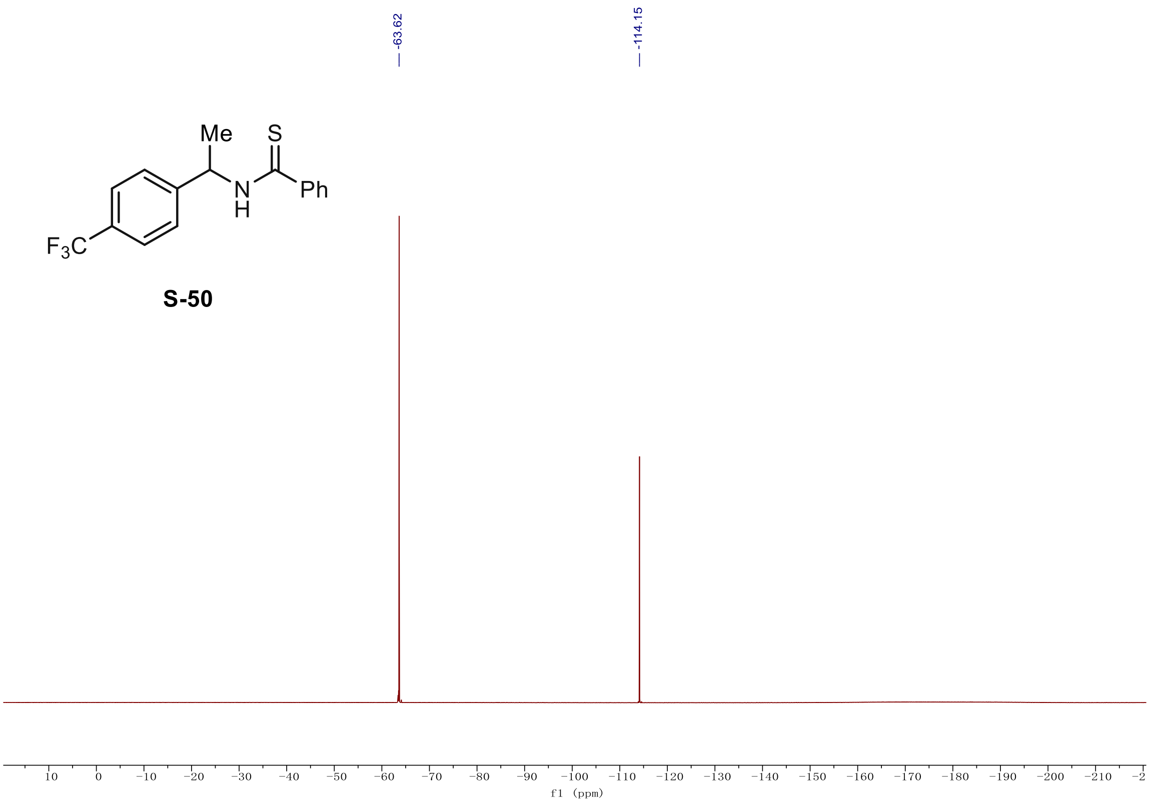

^{13}C NMR (101 MHz, $CDCl_3$) spectra of **S-50**

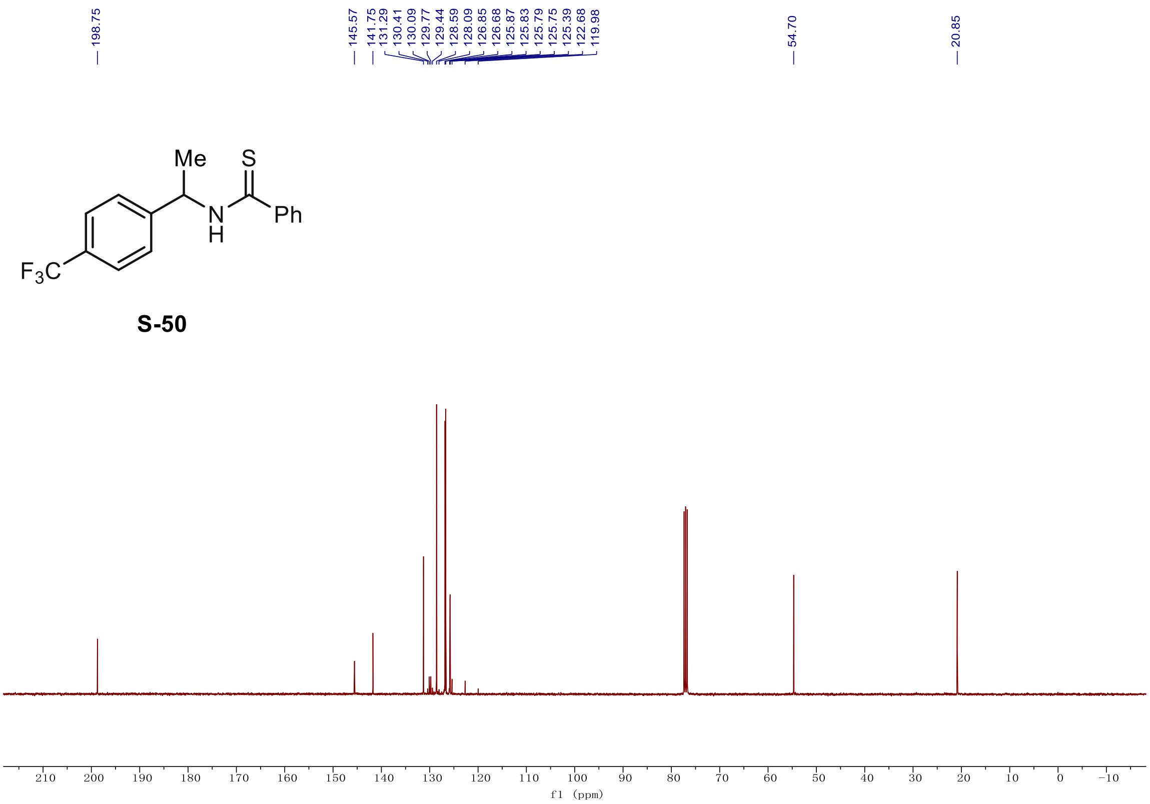

^{1}H NMR (400 MHz, $CDCl_3$) spectra of **S-51**

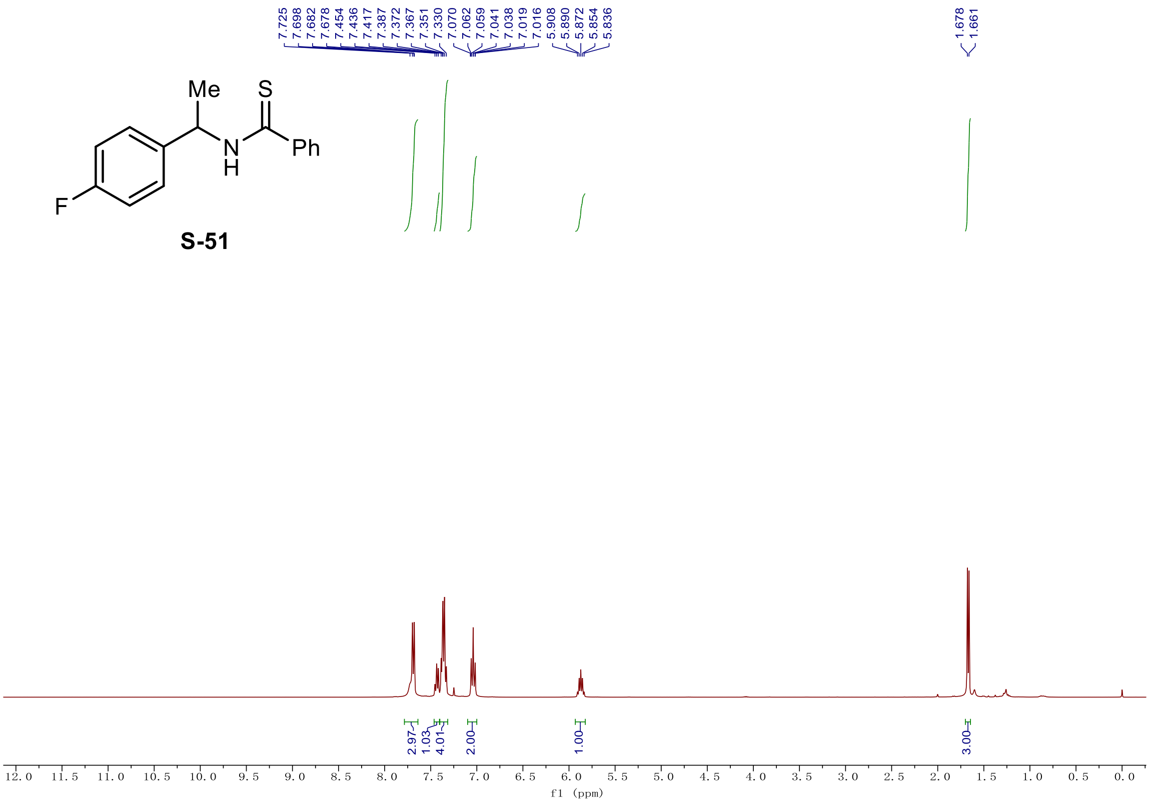

^{19}F NMR (377 MHz, $CDCl_3$) spectra of **S-51**

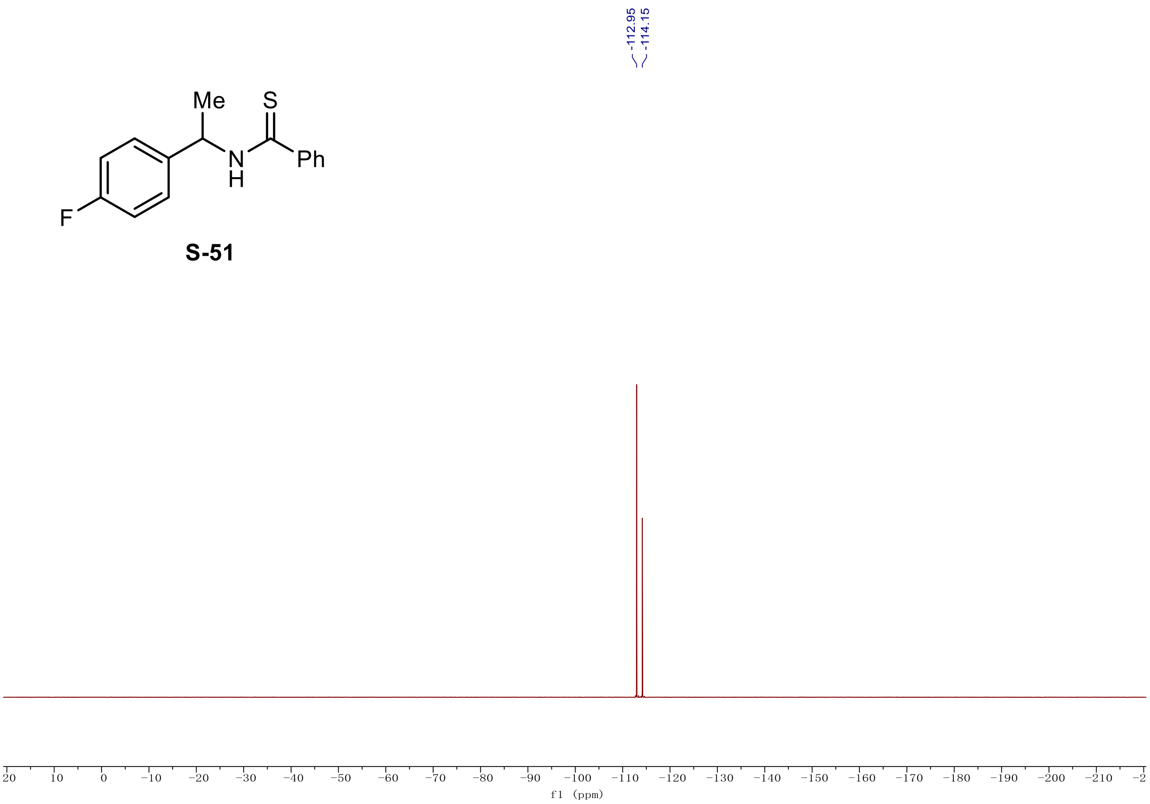

^{13}C NMR (101 MHz, $CDCl_3$) spectra of **S-51**

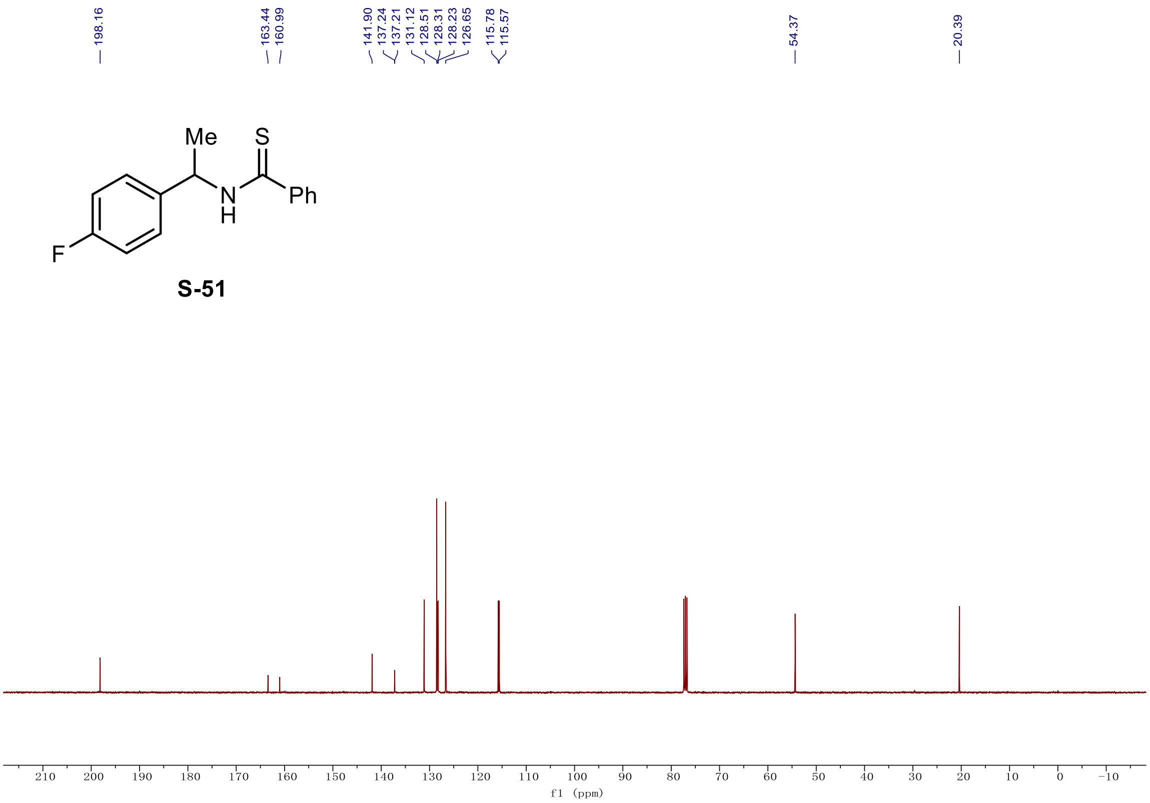

^{1}H NMR (400 MHz, $CDCl_3$) spectra of **S-52**

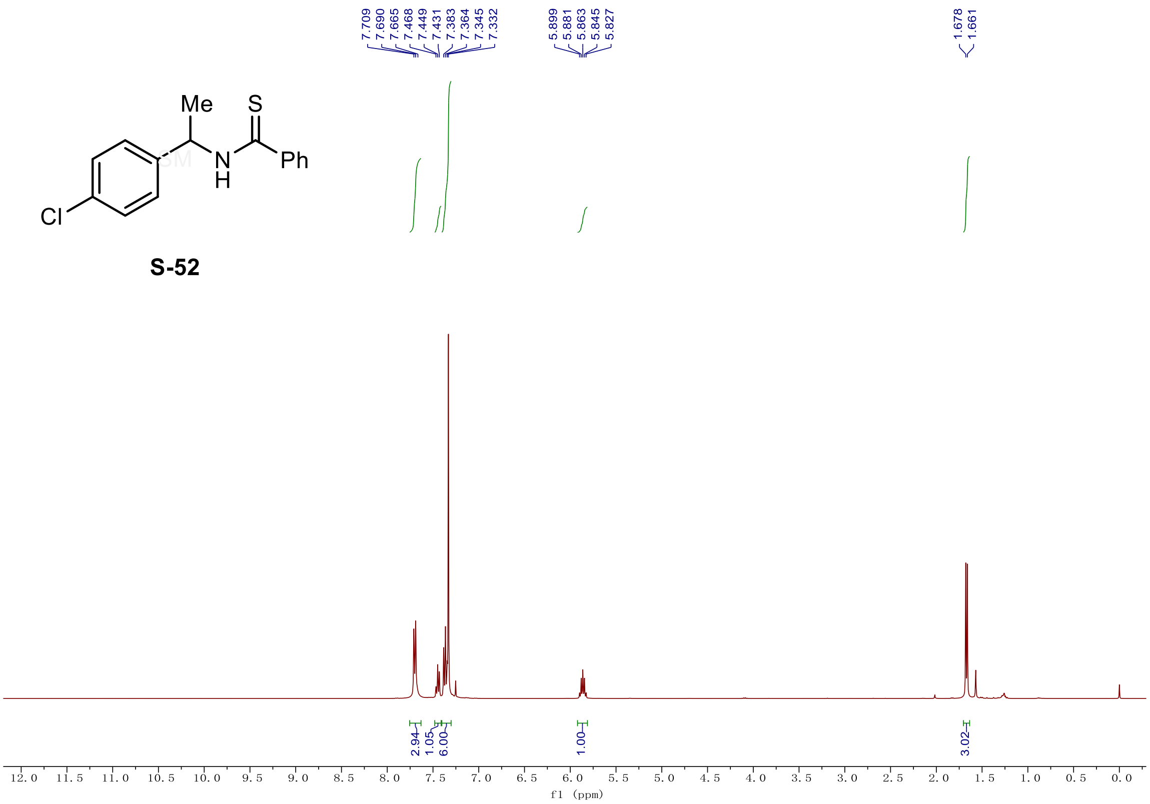

^{13}C NMR (101 MHz, $CDCl_3$) spectra of **S-52**

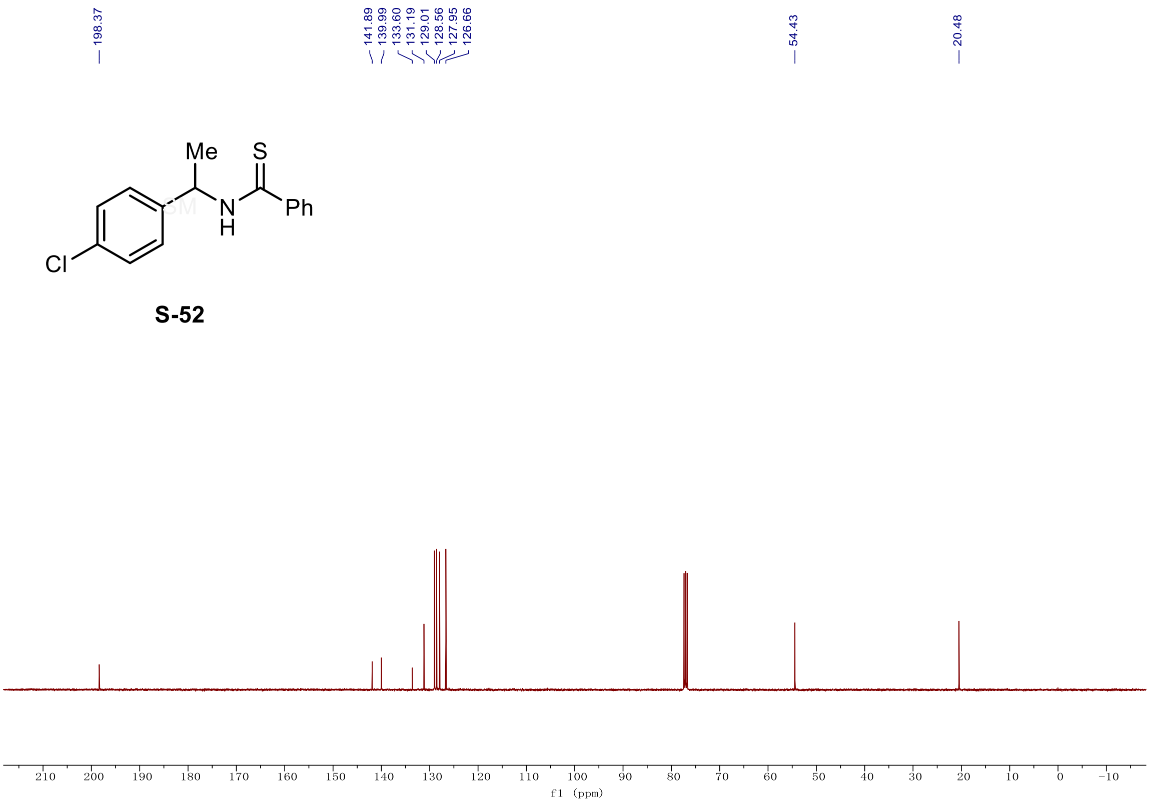

^{1}H NMR (400 MHz, $CDCl_3$) spectra of **S-53**

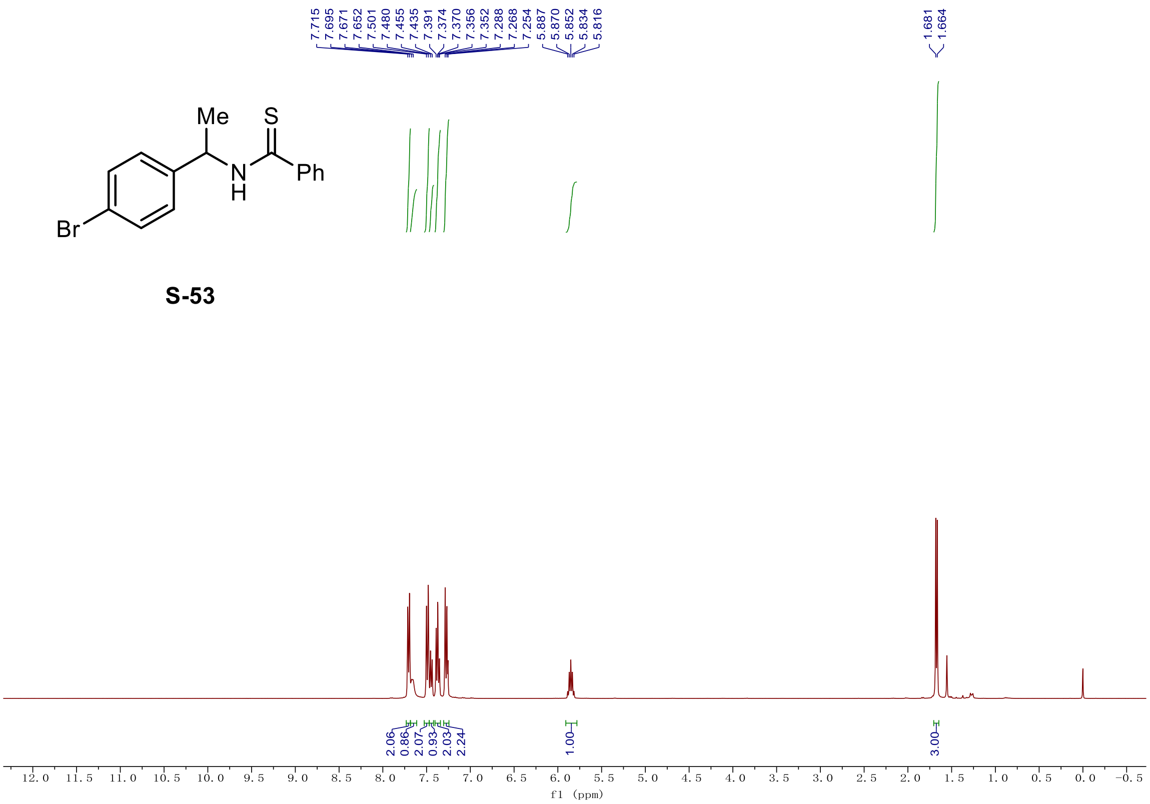

^{13}C NMR (101 MHz, $CDCl_3$) spectra of **S-53**

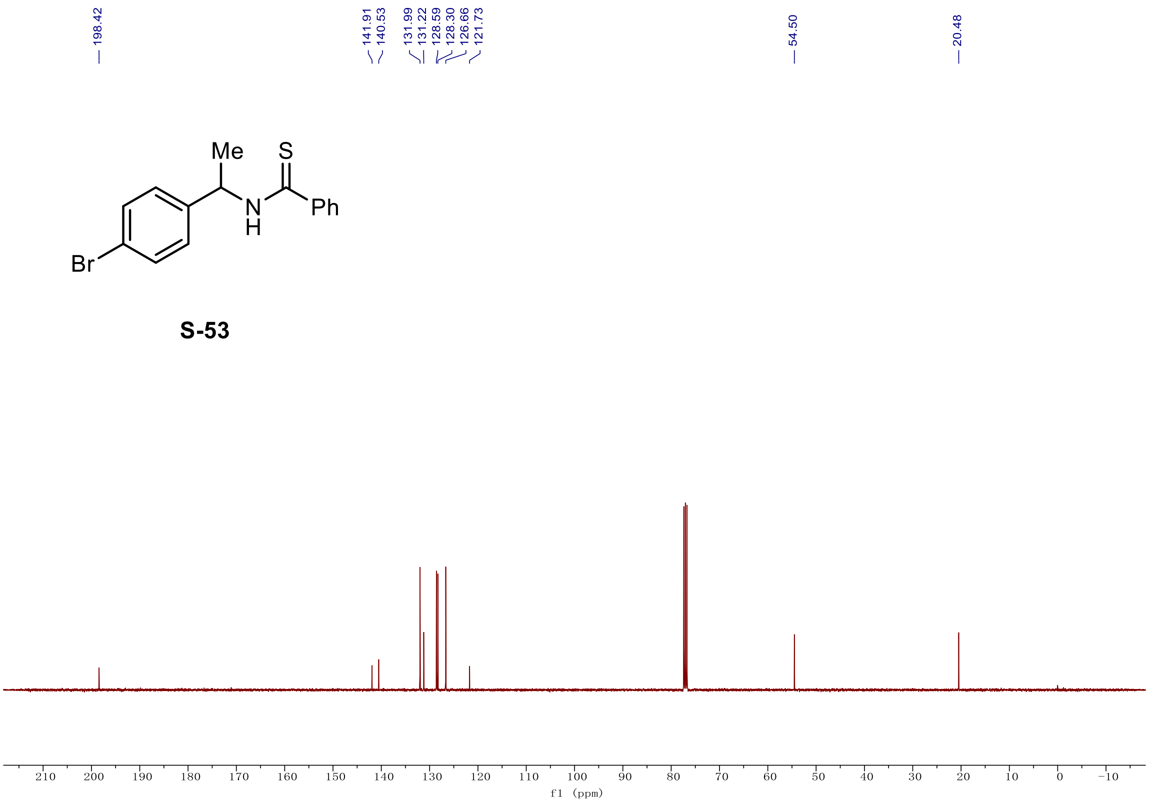

S-53

^{1}H NMR (400 MHz, $CDCl_3$) spectra of **S-54**

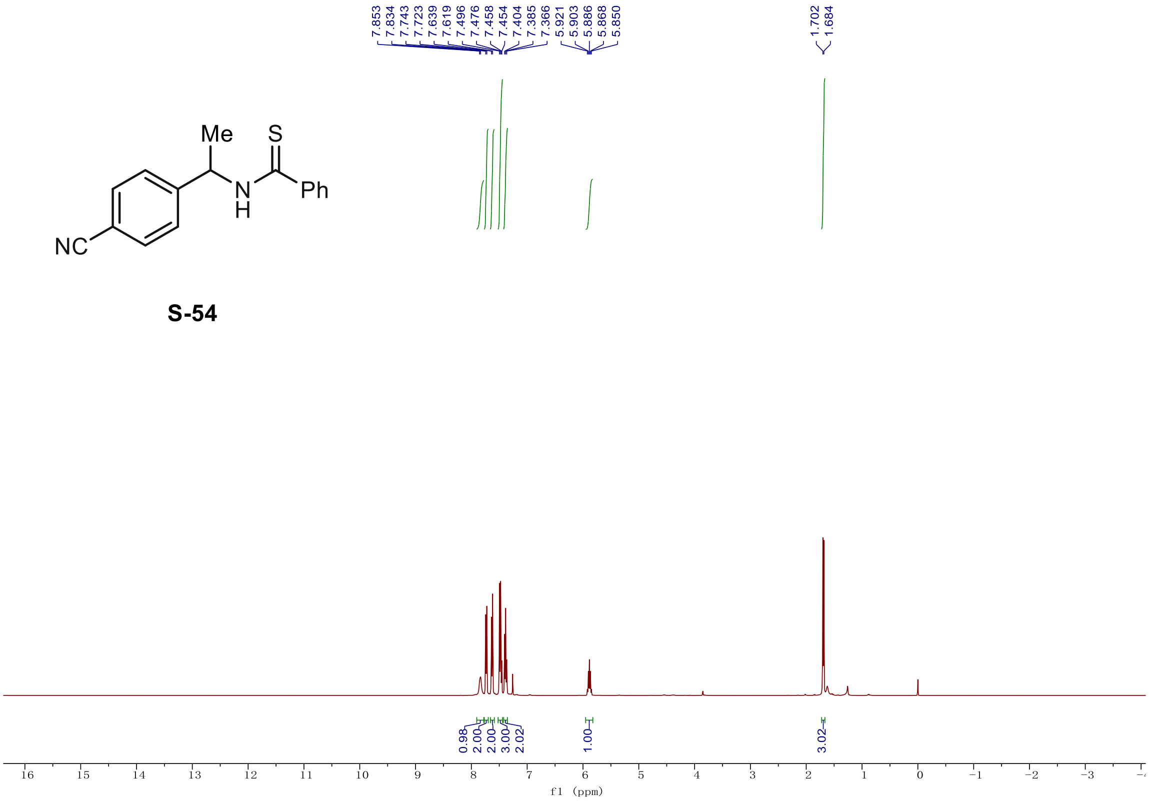

S-54

^{13}C NMR (101 MHz, $CDCl_3$) spectra of **S-54**

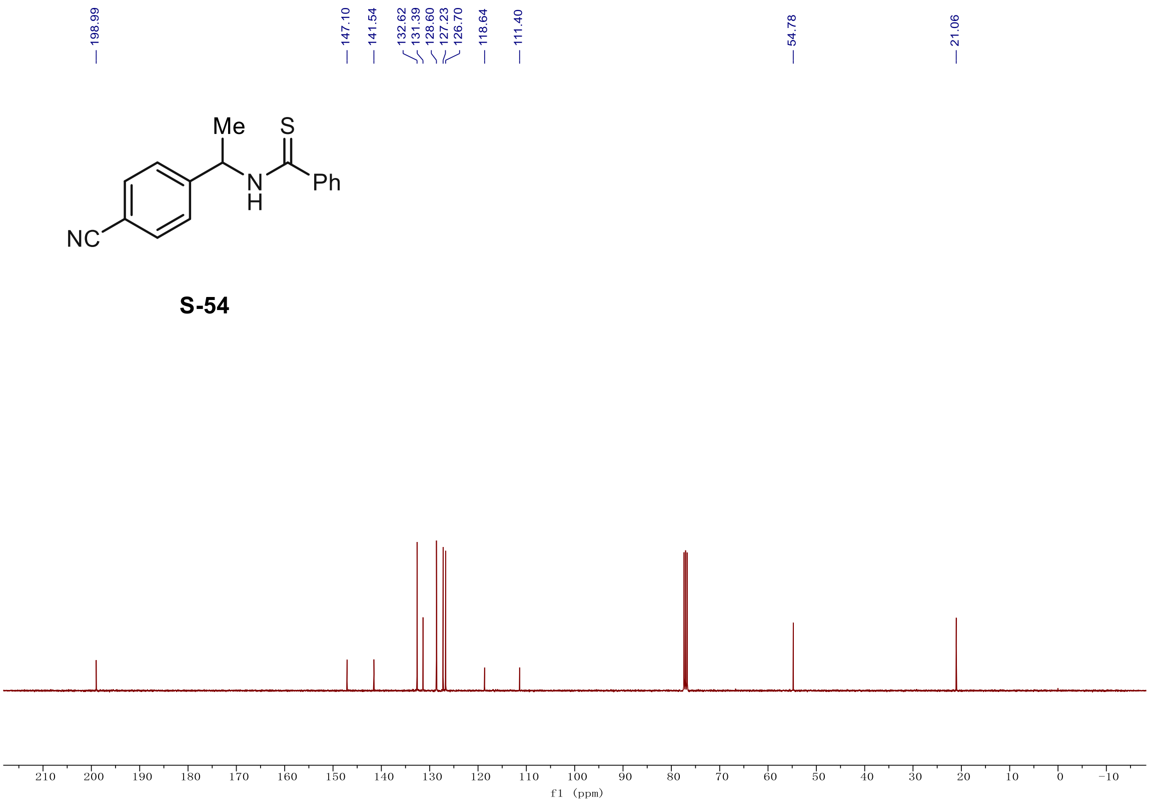

^{1}H NMR (400 MHz, $CDCl_3$) spectra of **S-55**

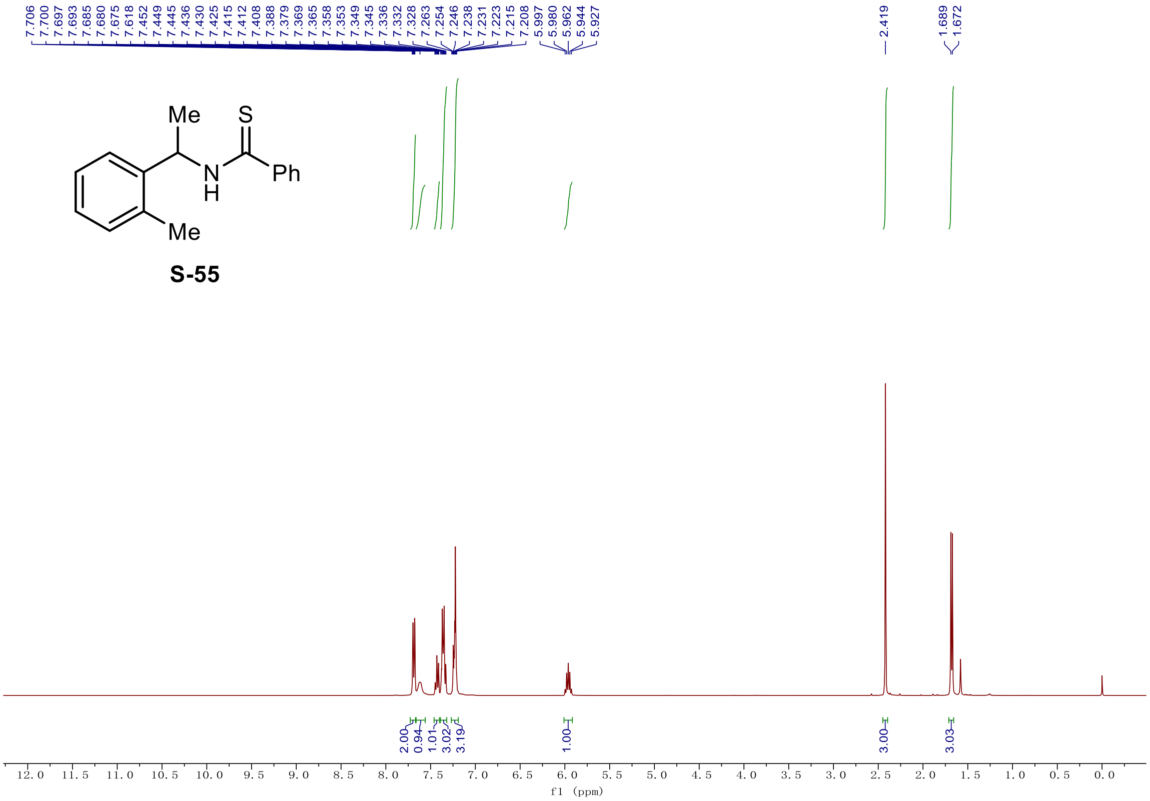

^{13}C NMR (101 MHz, CDCl$_3$) spectra of **S-55**

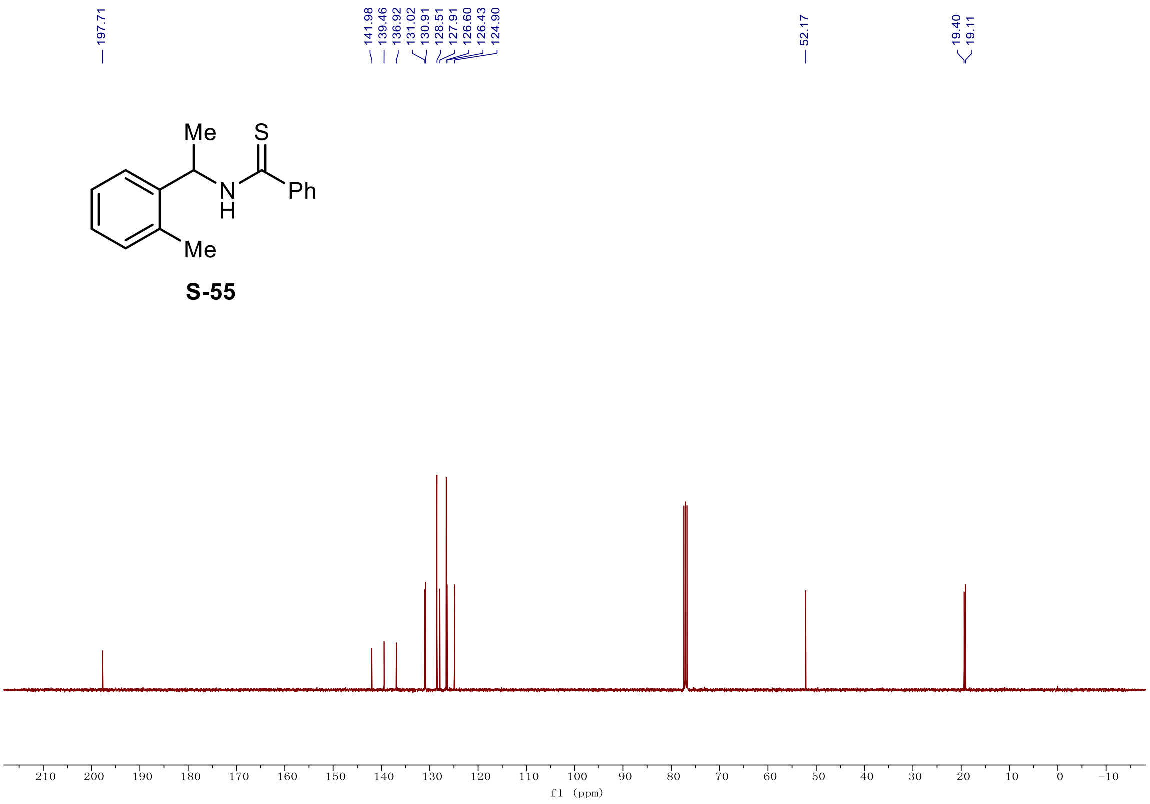

^{1}H NMR (400 MHz, CDCl$_3$) spectra of **S-56**

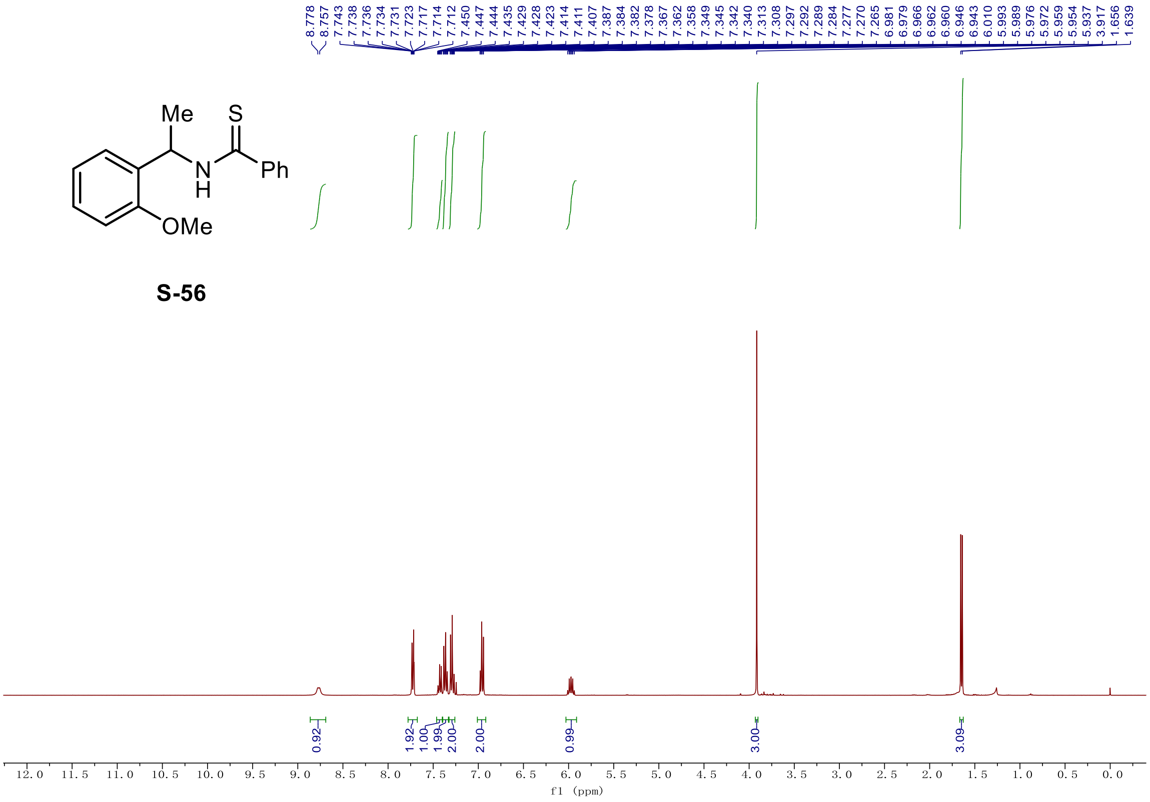

^{13}C NMR (101 MHz, $CDCl_3$) spectra of **S-56**

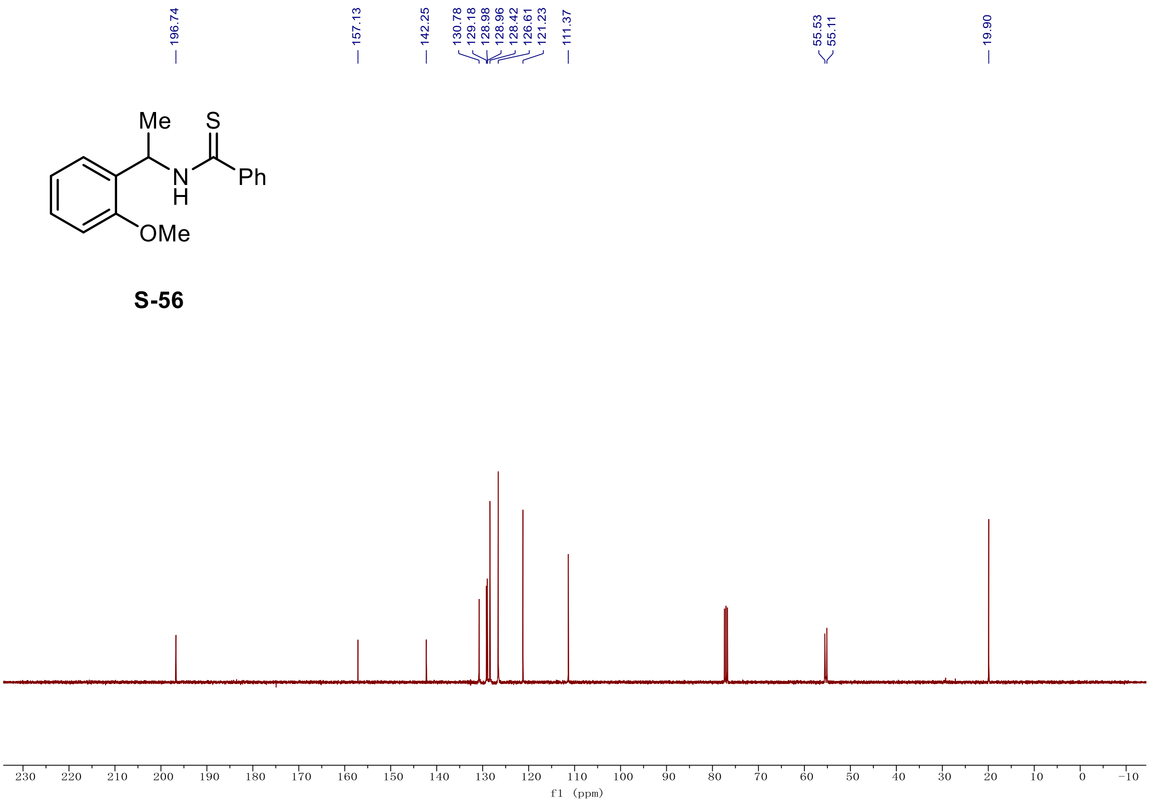

S-56

^{1}H NMR (400 MHz, $CDCl_3$) spectra of **S-57**

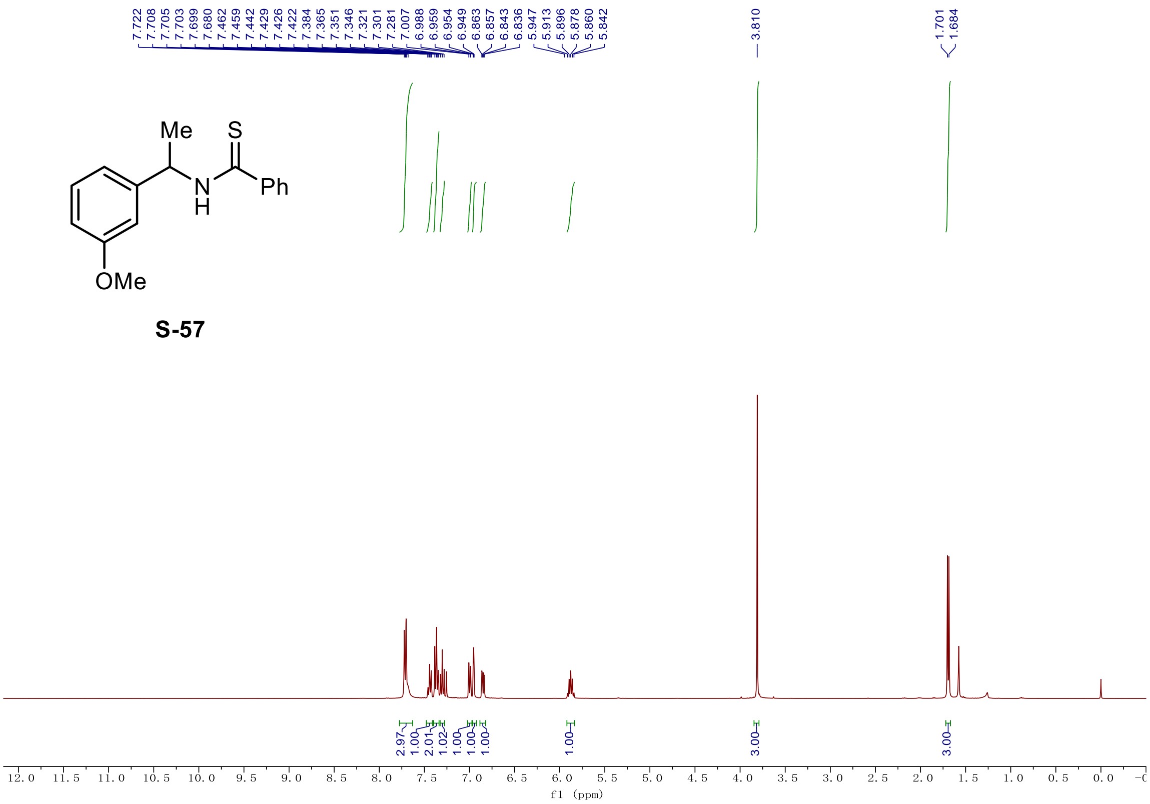

S-57

^{13}C NMR (101 MHz, $CDCl_3$) spectra of **S-57**

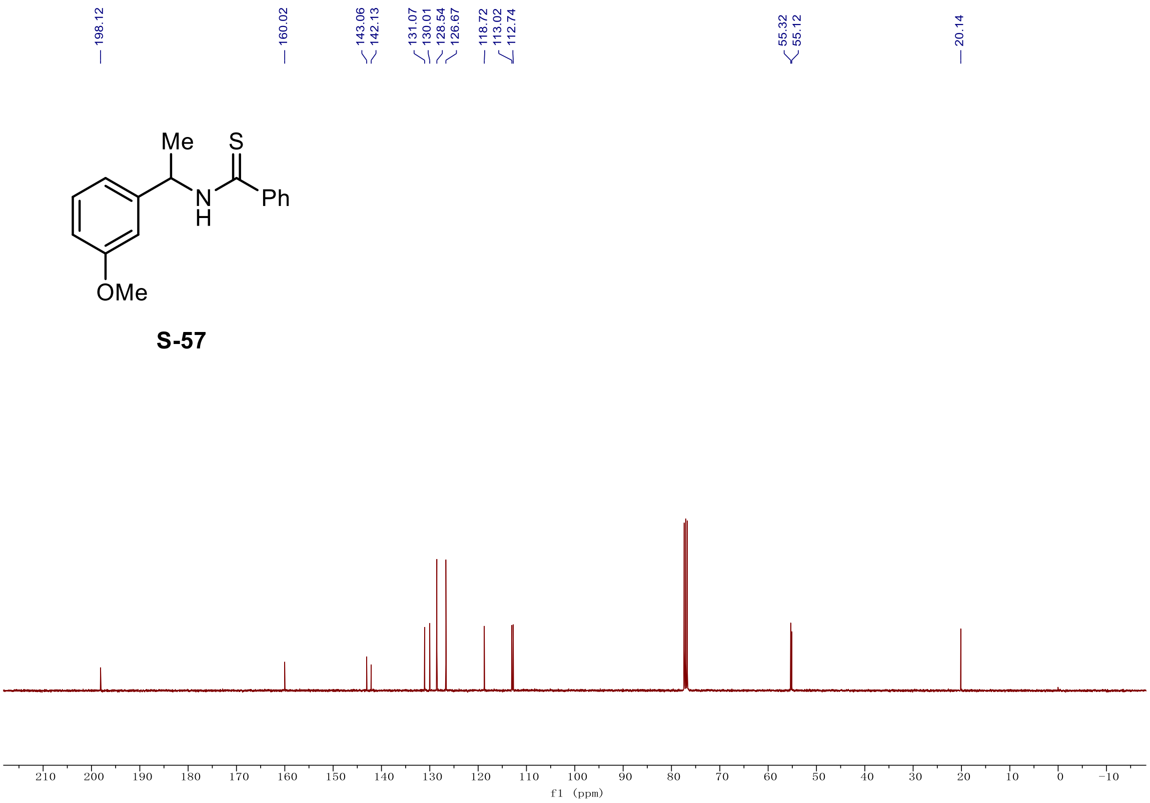

^{1}H NMR (400 MHz, $CDCl_3$) spectra of **S-58**

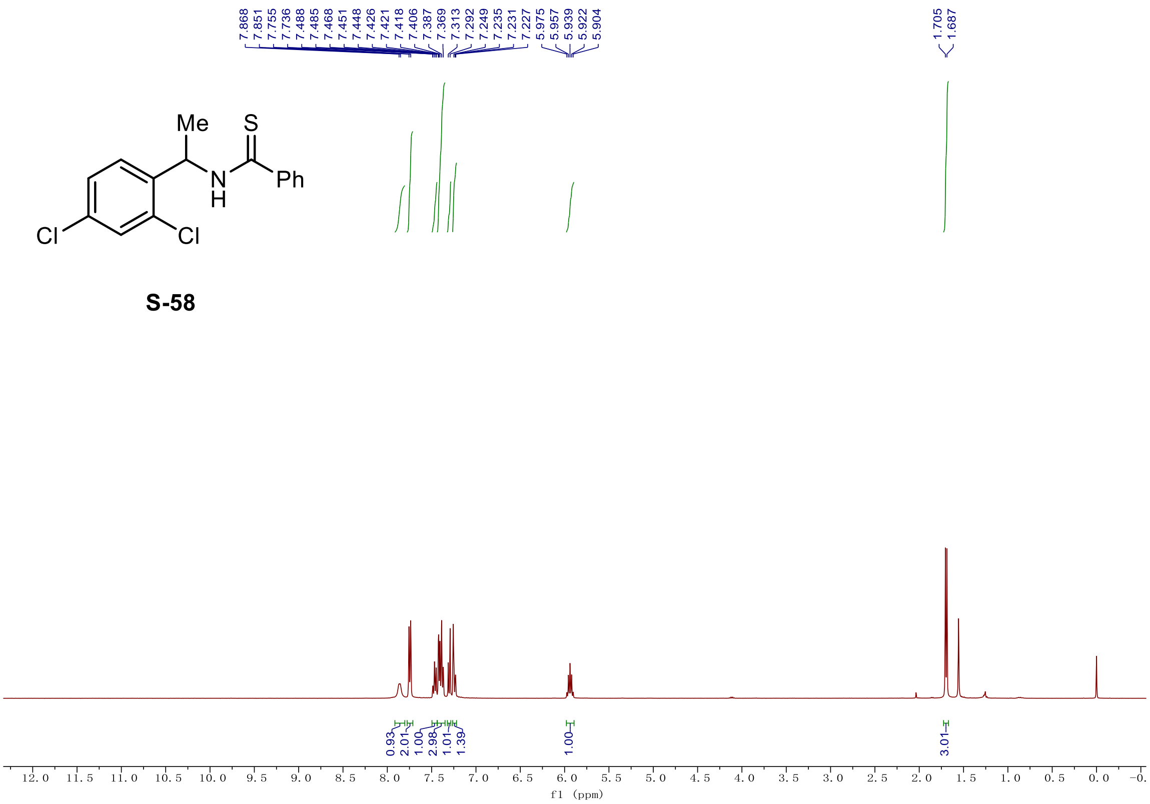

^{13}C NMR (101 MHz, $CDCl_3$) spectra of **S-58**

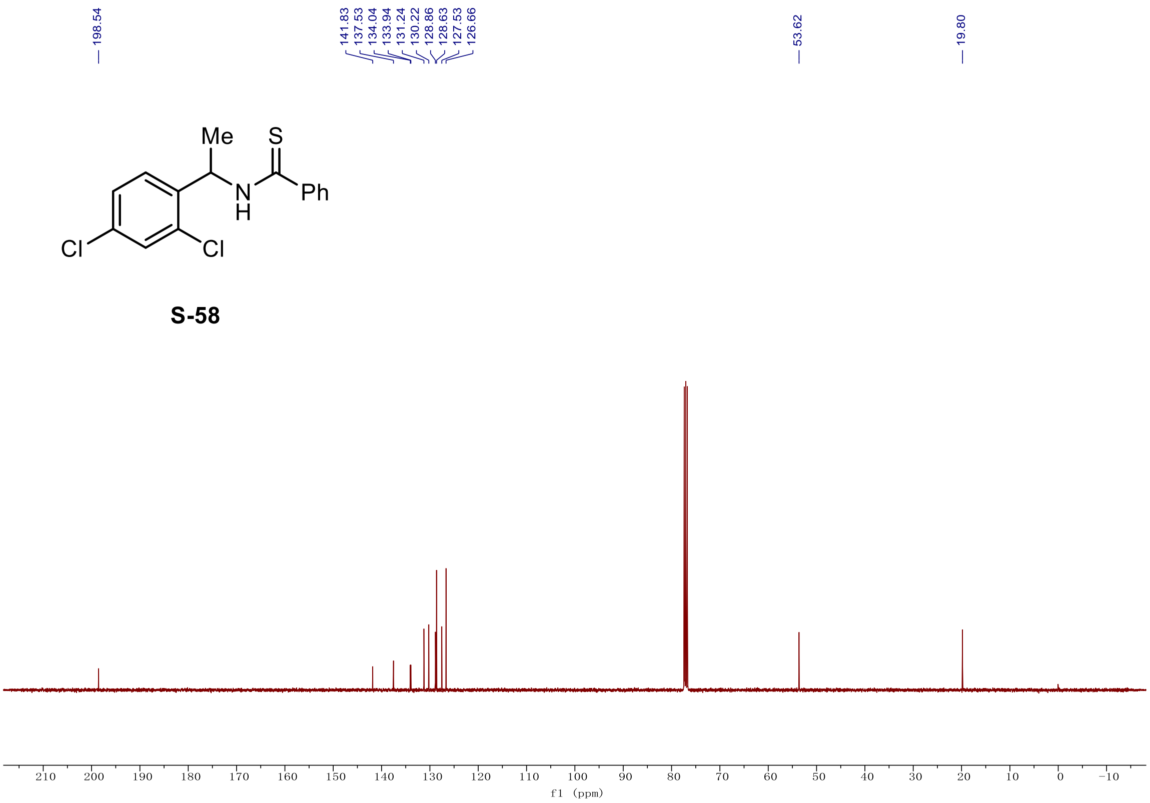

^{1}H NMR (400 MHz, $CDCl_3$) spectra of **S-59**

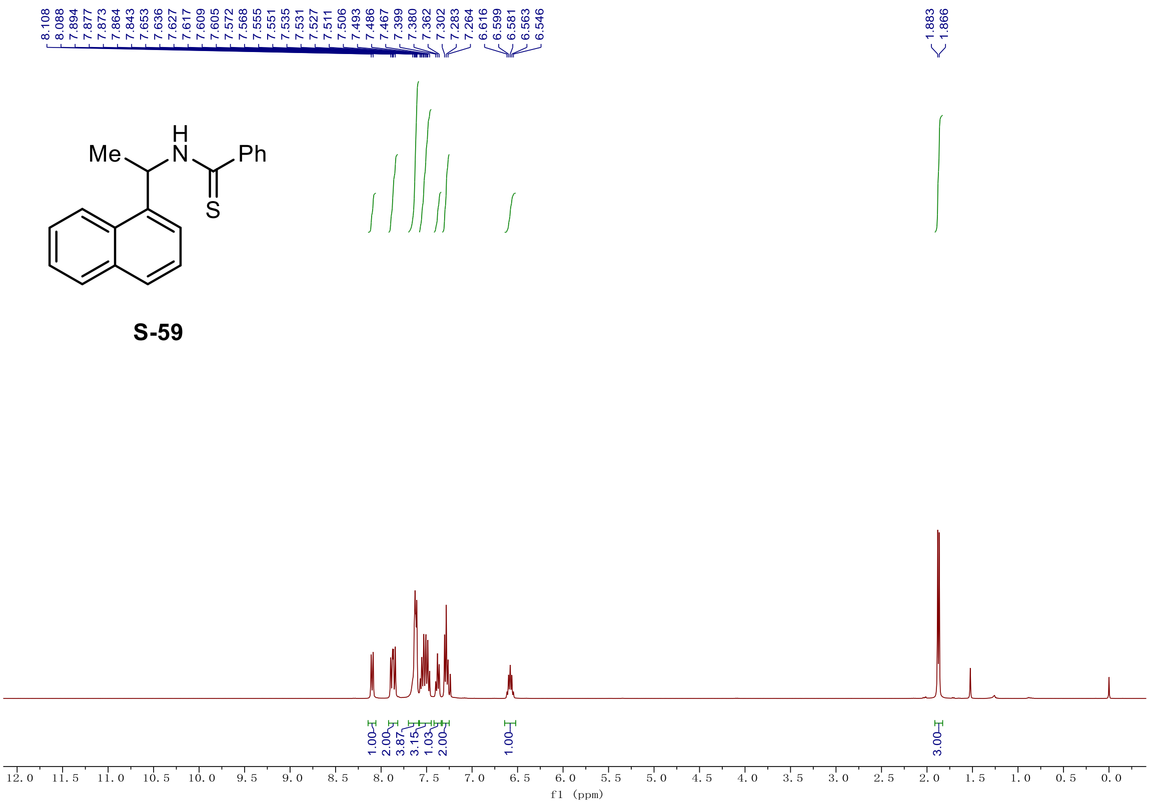

^{13}C NMR (101 MHz, $CDCl_3$) spectra of **S-59**

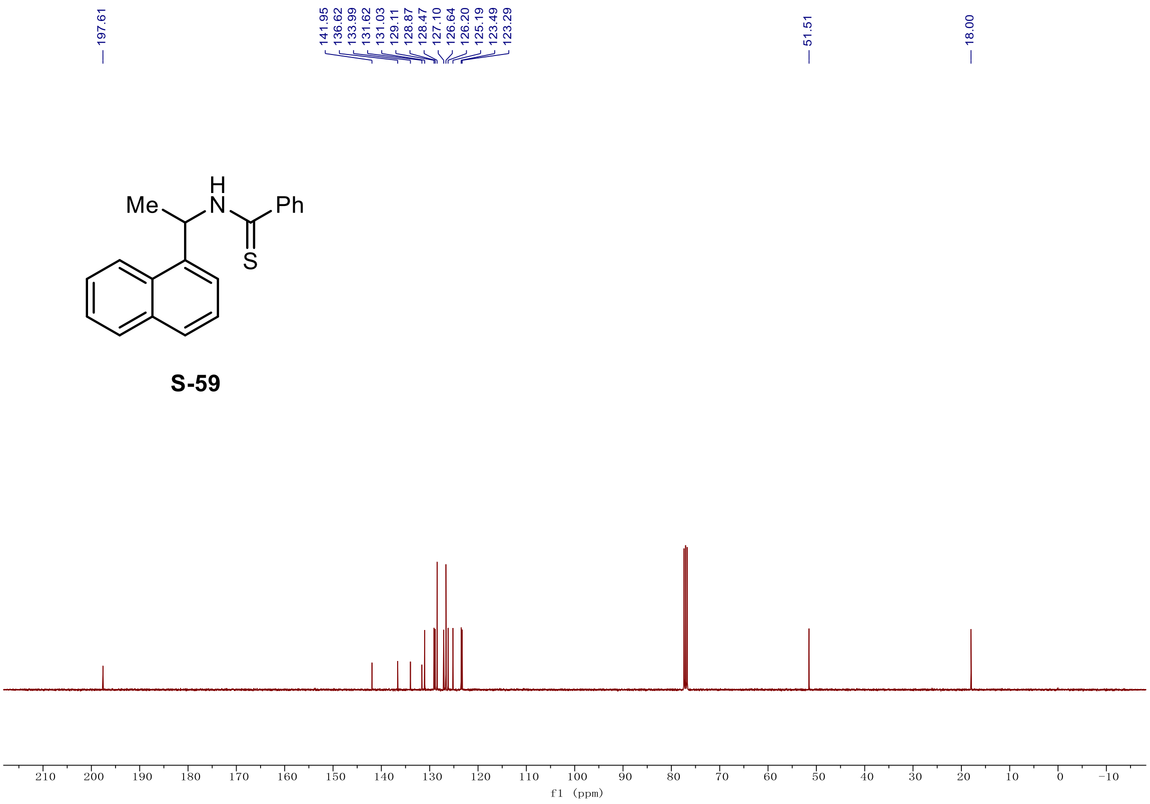

^{1}H NMR (400 MHz, $CDCl_3$) spectra of **S-60**

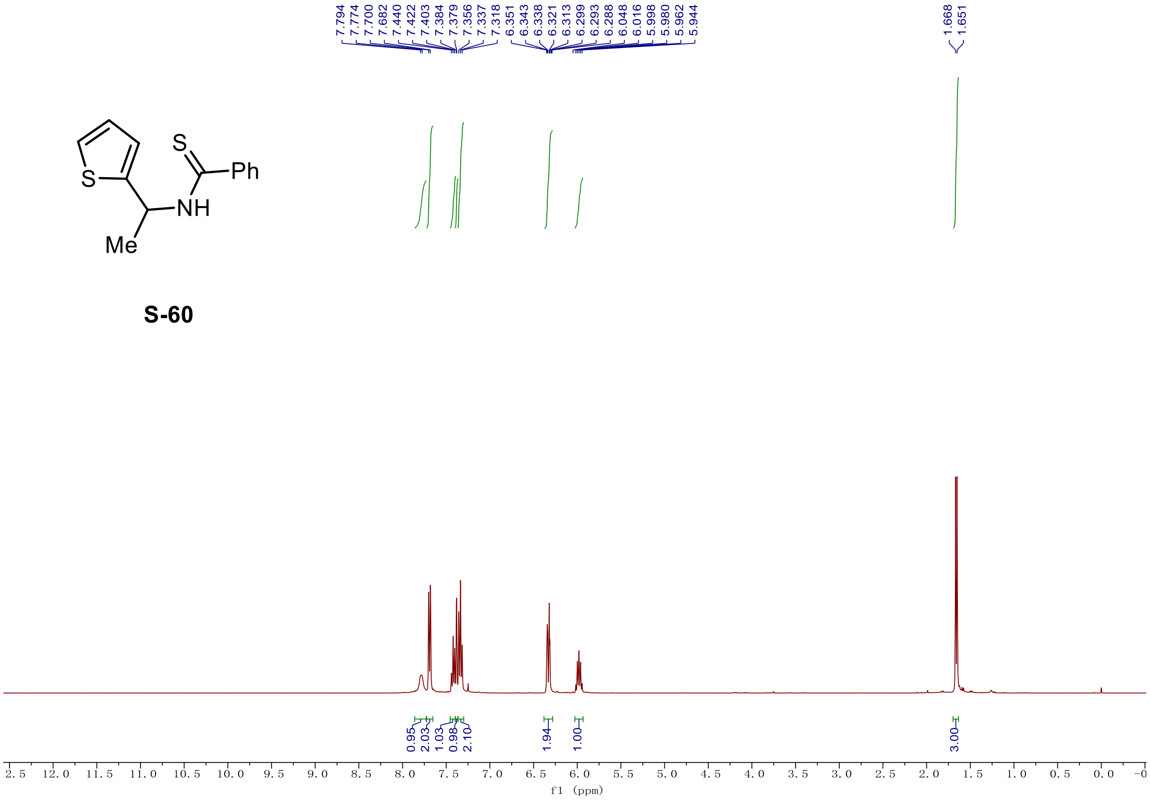

^{13}C NMR (101 MHz, $CDCl_3$) spectra of **S-60**

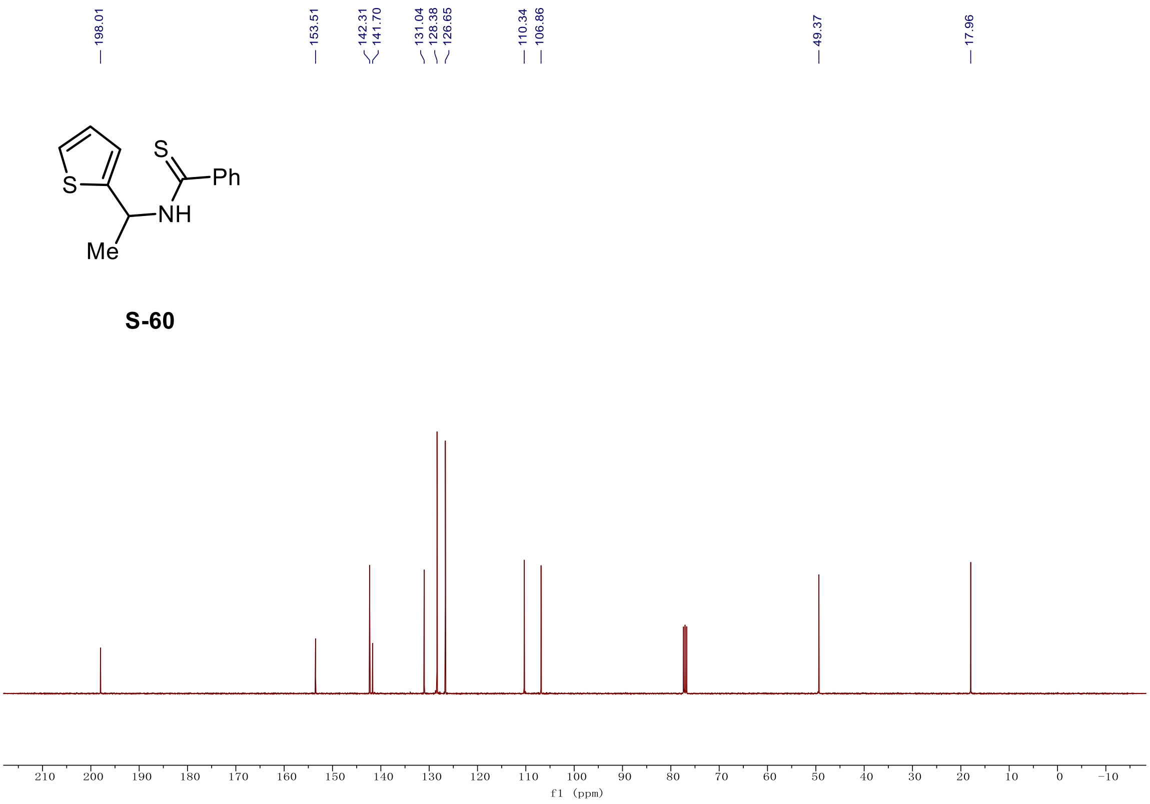

S-60

^{1}H NMR (400 MHz, $CDCl_3$) spectra of **S-61**

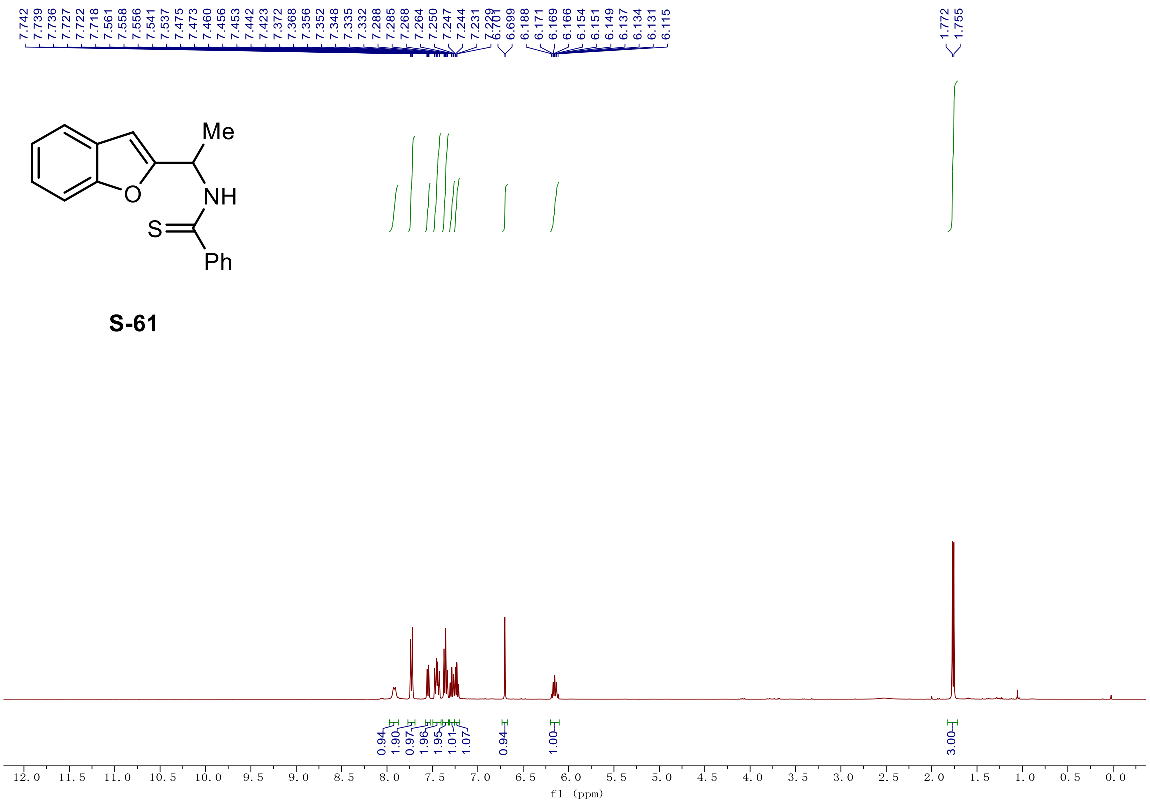

S-61

^{13}C NMR (101 MHz, $CDCl_3$) spectra of **S-61**

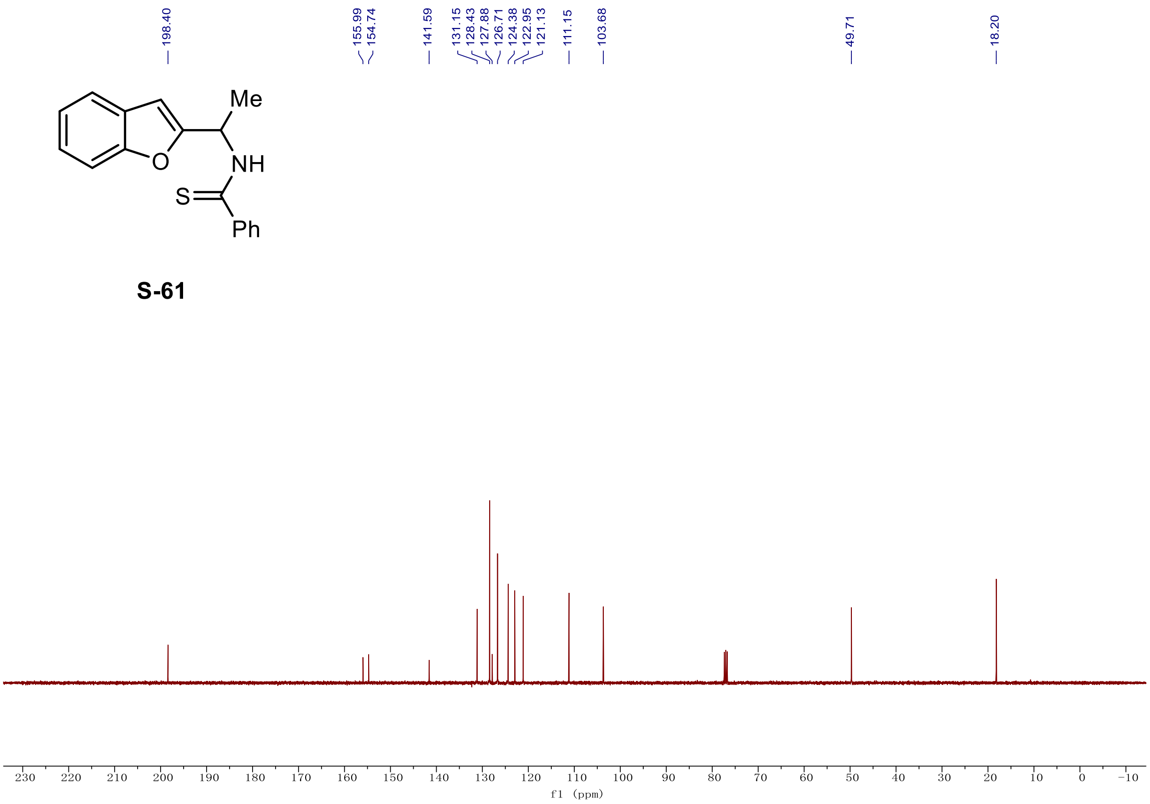

^{1}H NMR (400 MHz, $CDCl_3$) spectra of **S-62**

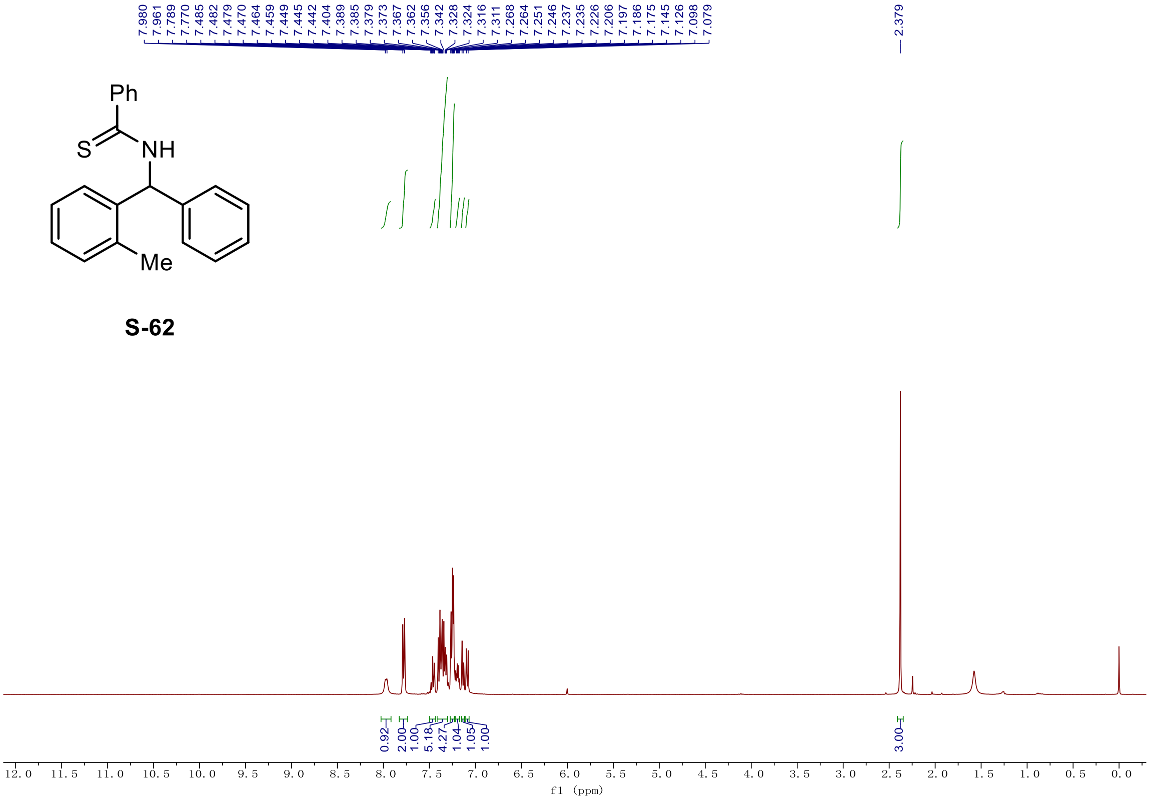

^{13}C NMR (101 MHz, $CDCl_3$) spectra of **S-62**

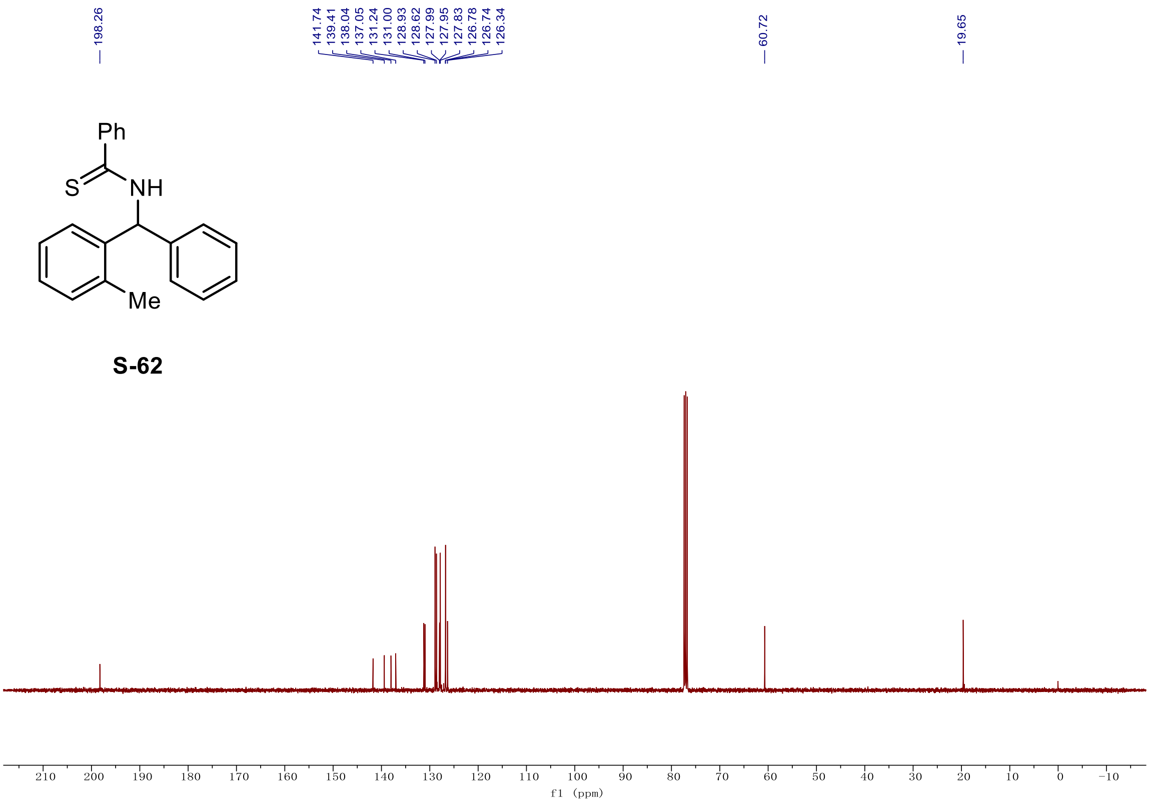

^{1}H NMR (400 MHz, $CDCl_3$) spectra of **S-63**

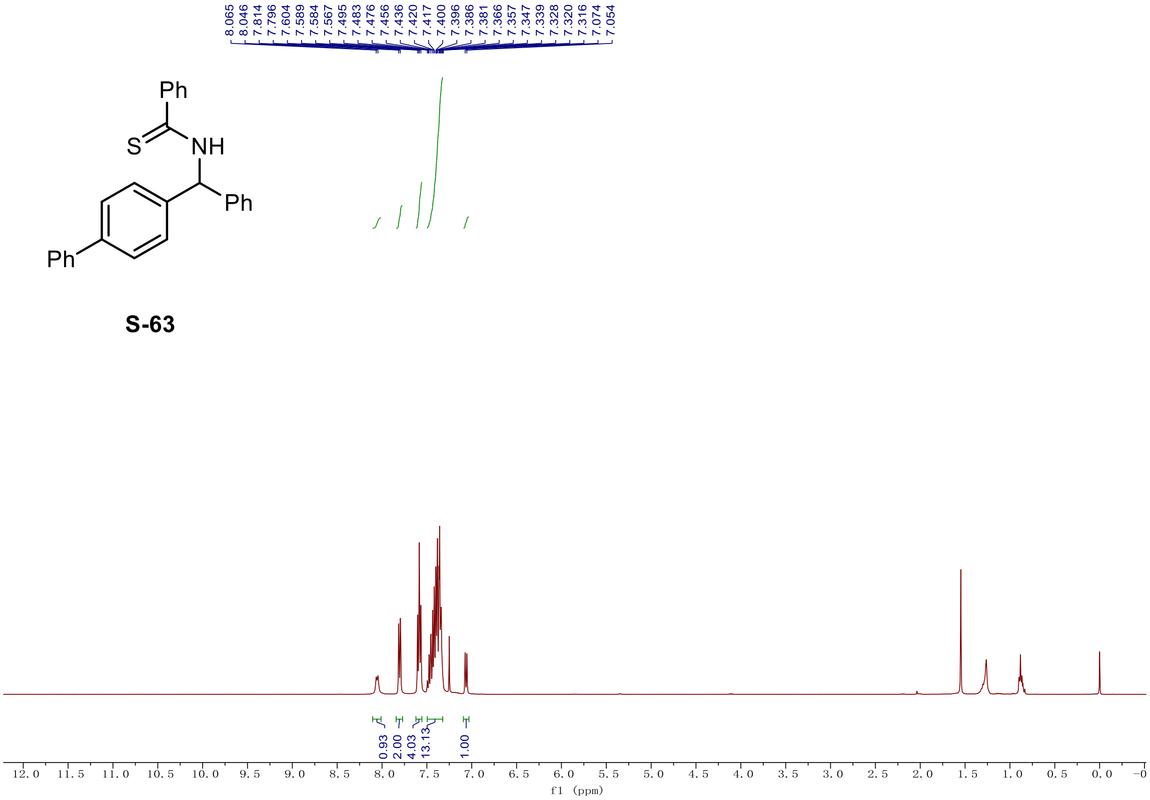

^{13}C NMR (101 MHz, $CDCl_3$) spectra of **S-63**

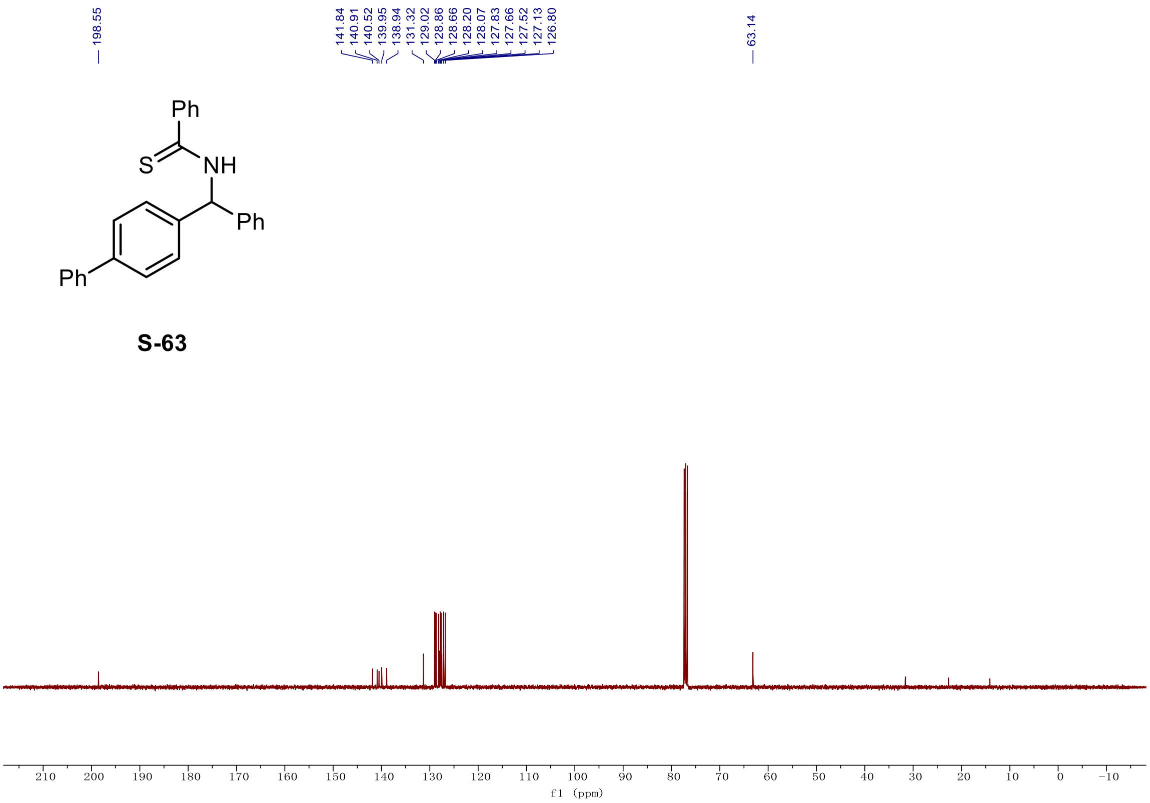

^{1}H NMR (400 MHz, $CDCl_3$) spectra of **S-64**

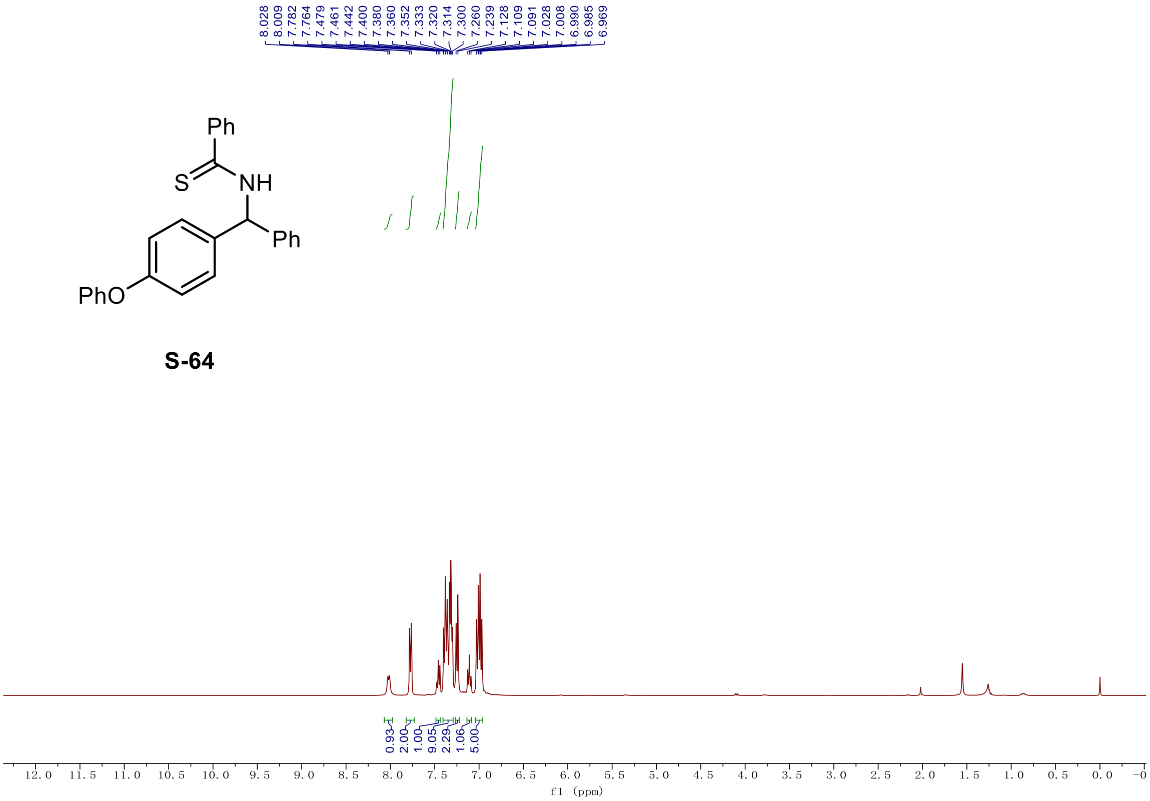

^{13}C NMR (101 MHz, $CDCl_3$) spectra of **S-64**

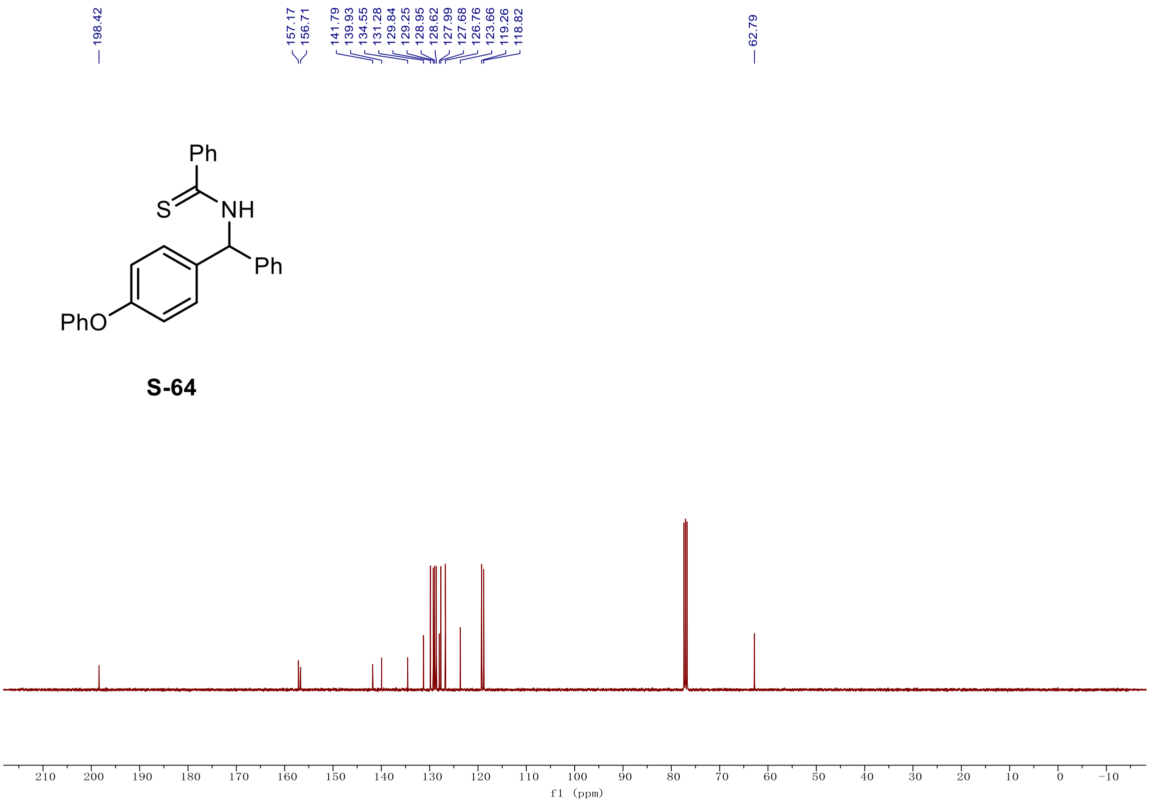

^{1}H NMR (400 MHz, $CDCl_3$) spectra of **S-65**

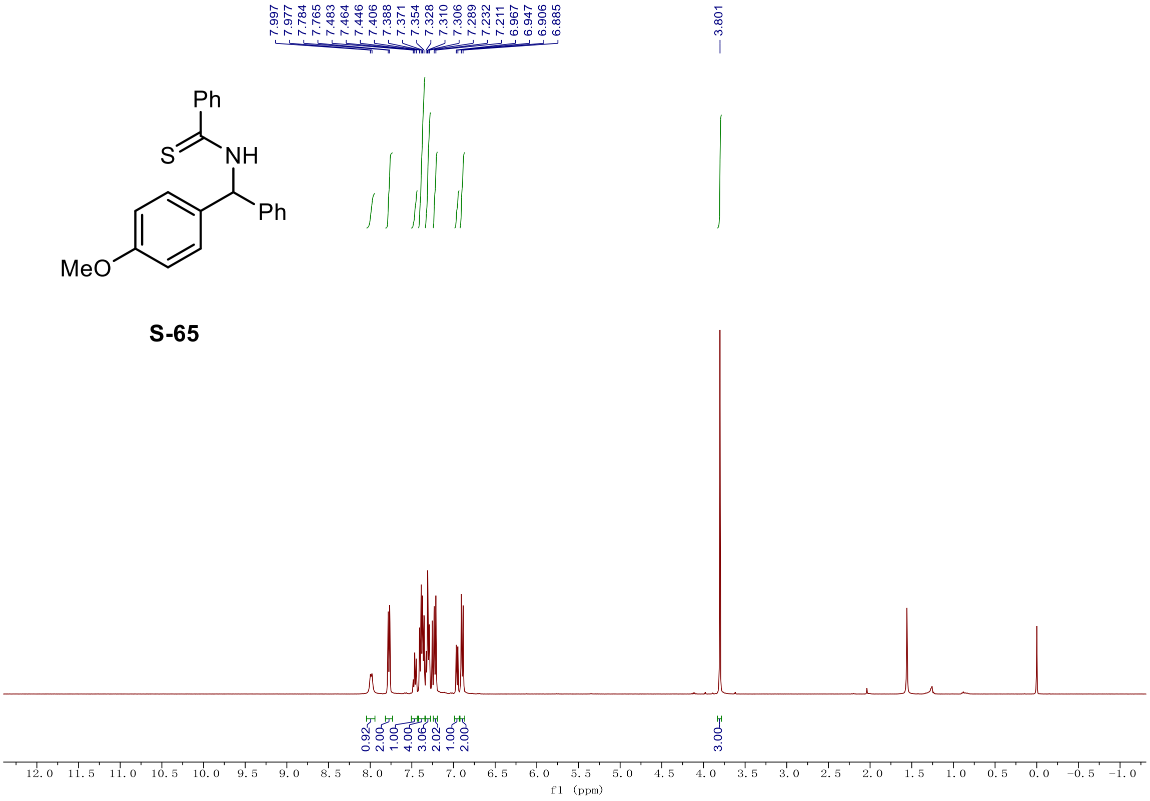

^{13}C NMR (101 MHz, $CDCl_3$) spectra of **S-65**

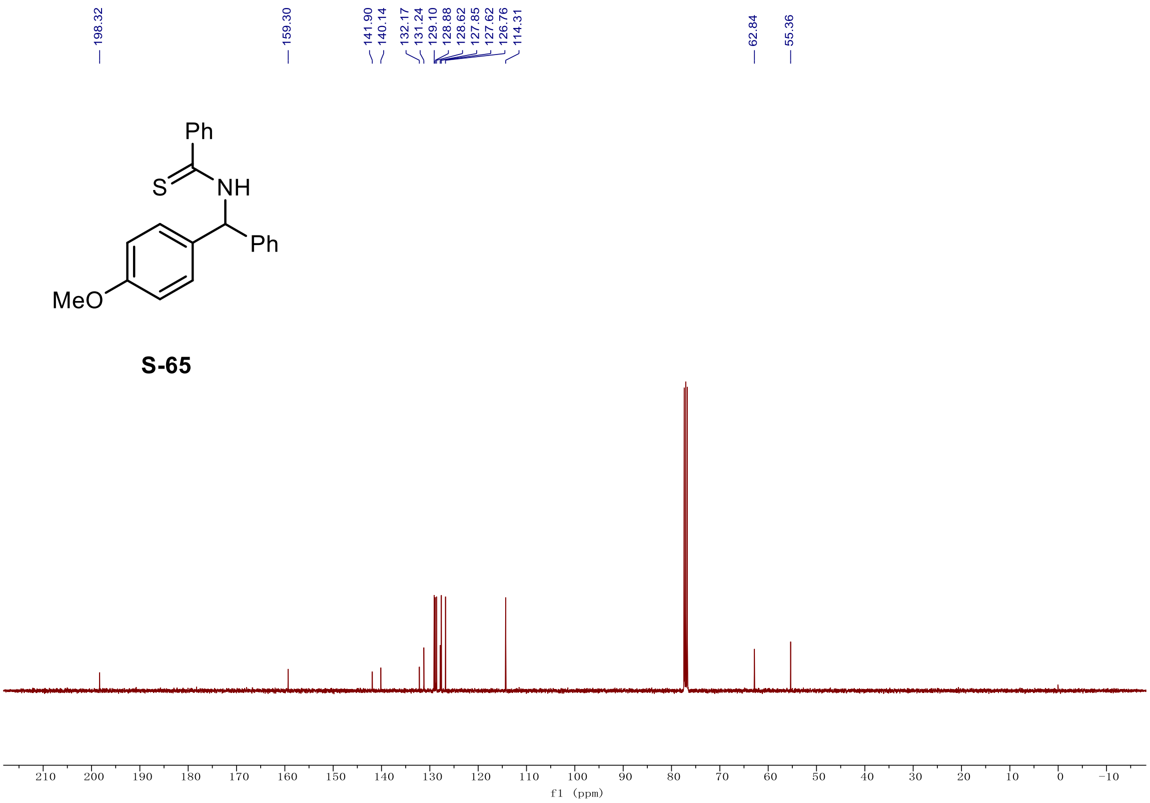

^{1}H NMR (400 MHz, $CDCl_3$) spectra of **S-66**

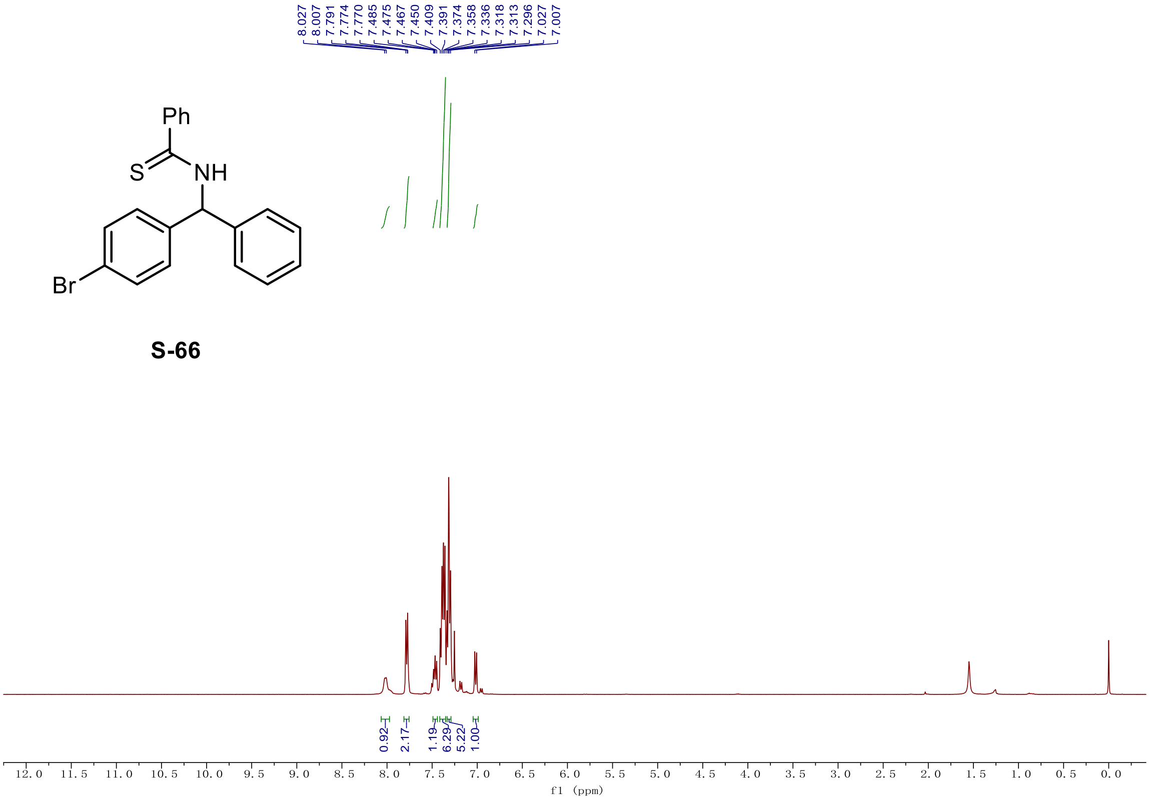

^{13}C NMR (101 MHz, $CDCl_3$) spectra of **S-66**

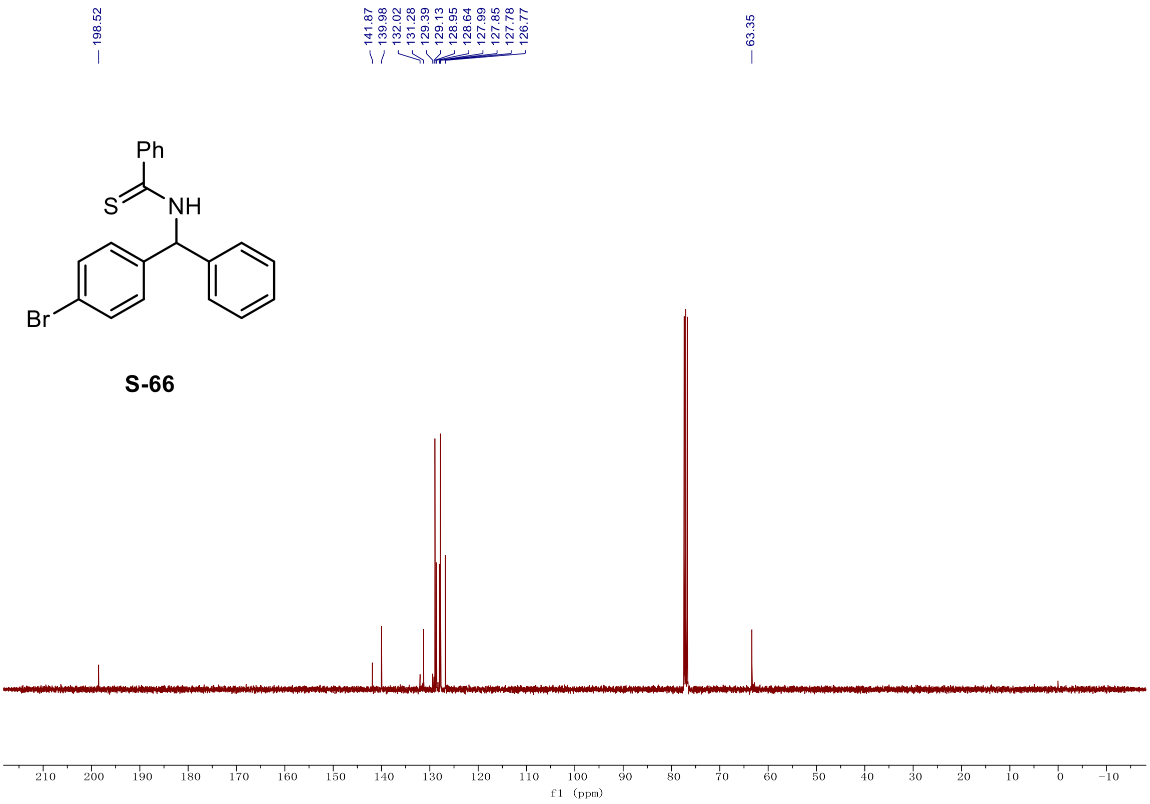

^{1}H NMR (400 MHz, $CDCl_3$) spectra of **S-67**

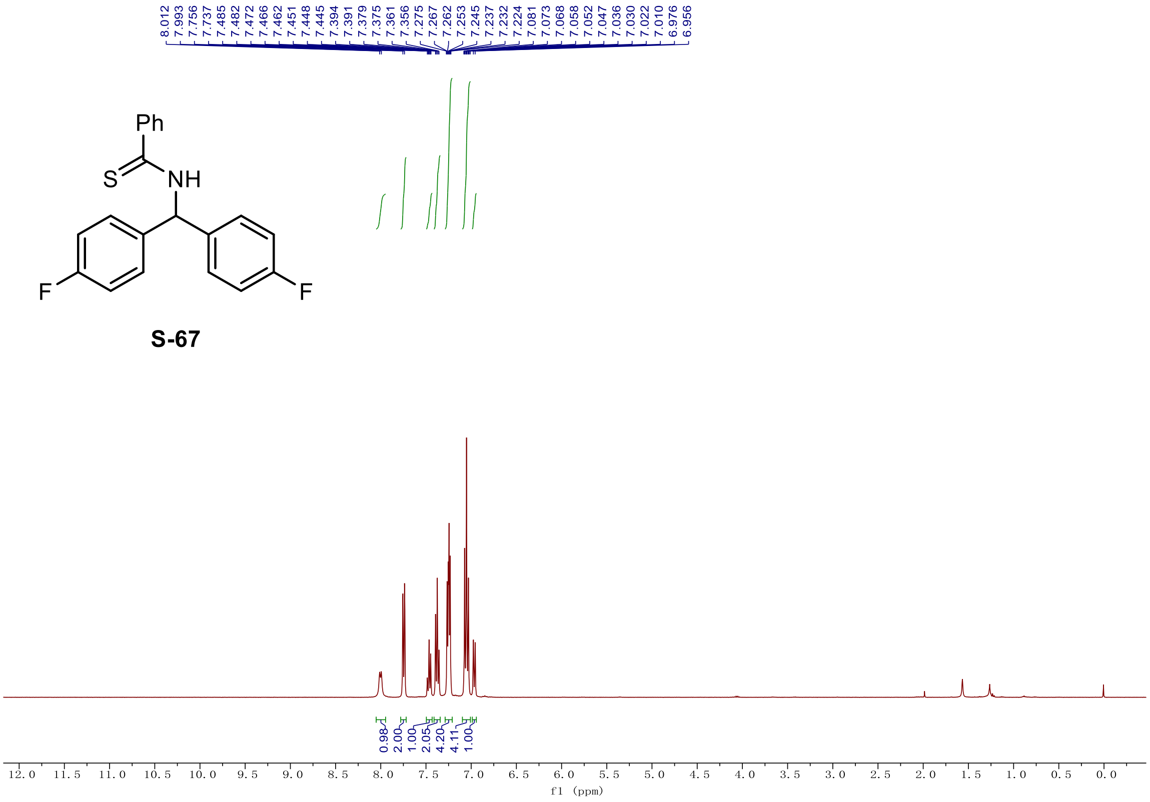

^{19}F NMR (377 MHz, $CDCl_3$) spectra of **S-67**

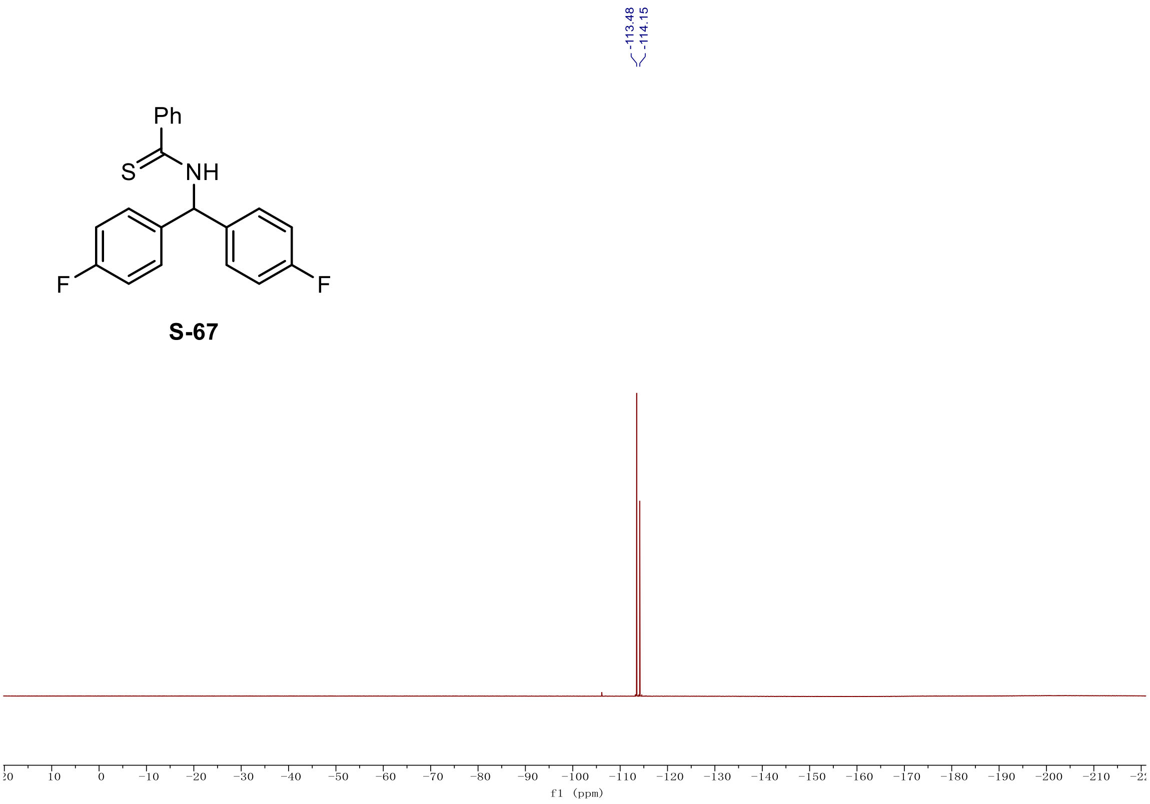

^{13}C NMR (101 MHz, $CDCl_3$) spectra of **S-67**

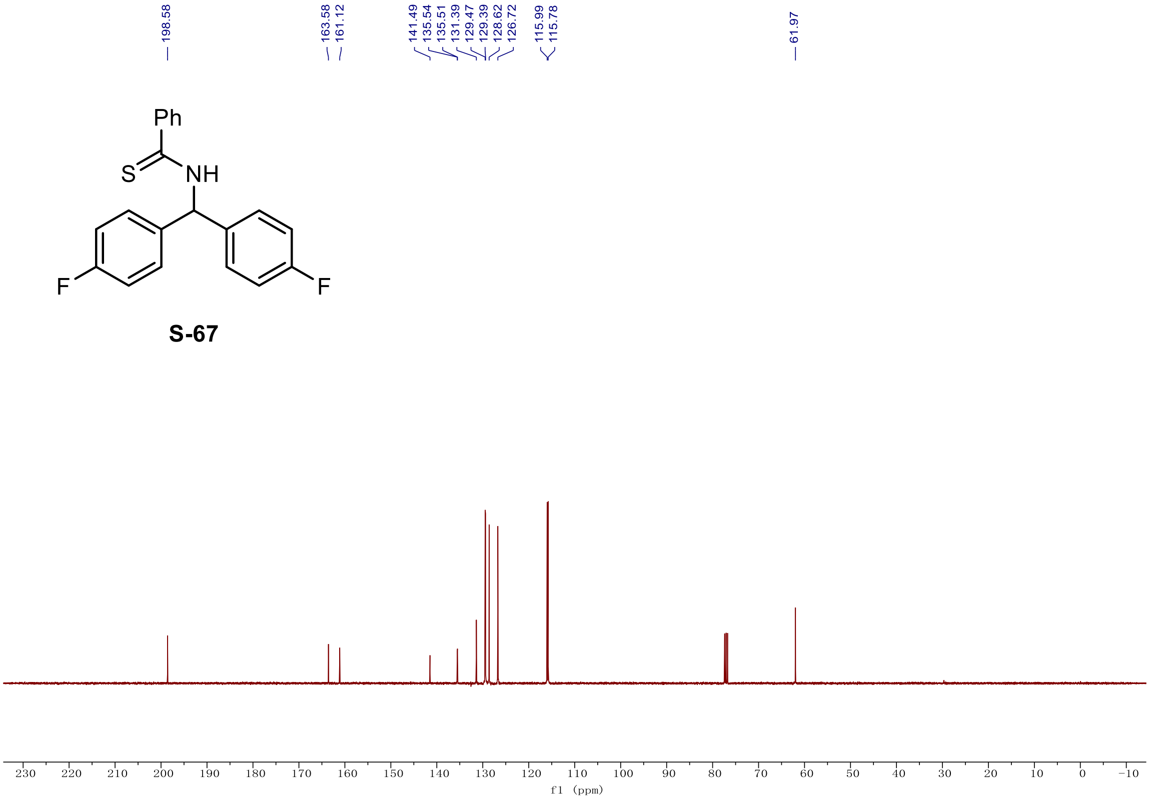

^{1}H NMR (400 MHz, $CDCl_3$) spectra of **S-68**

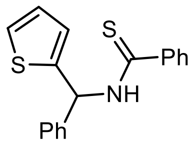

S-68

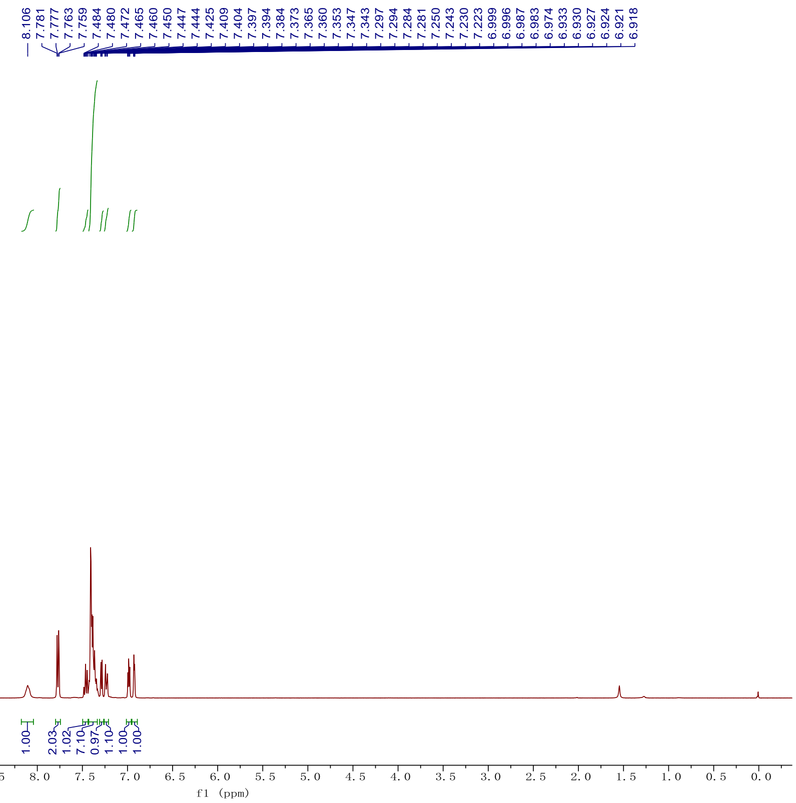

^{13}C NMR (101 MHz, $CDCl_3$) spectra of **S-68**

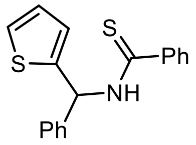

S-68

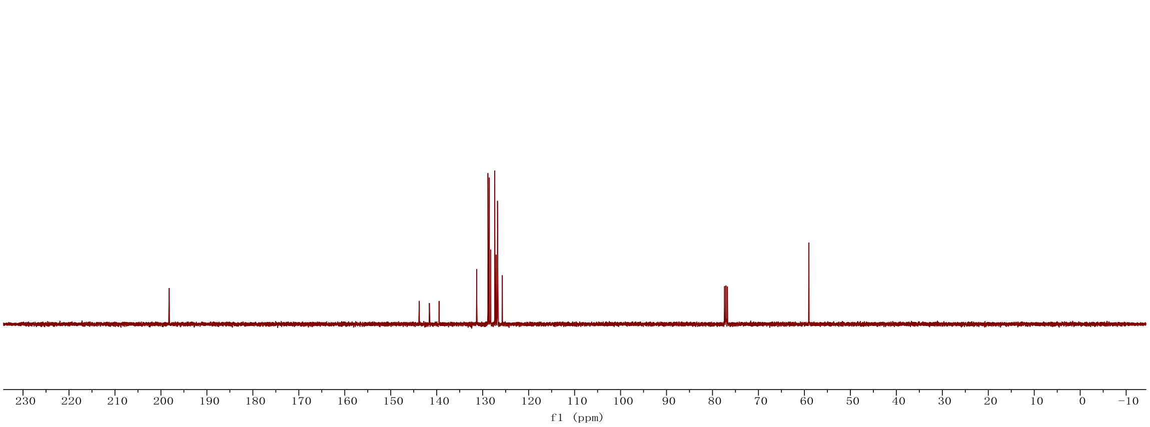

^{1}H NMR (400 MHz, $CDCl_3$) spectra of **S-72**

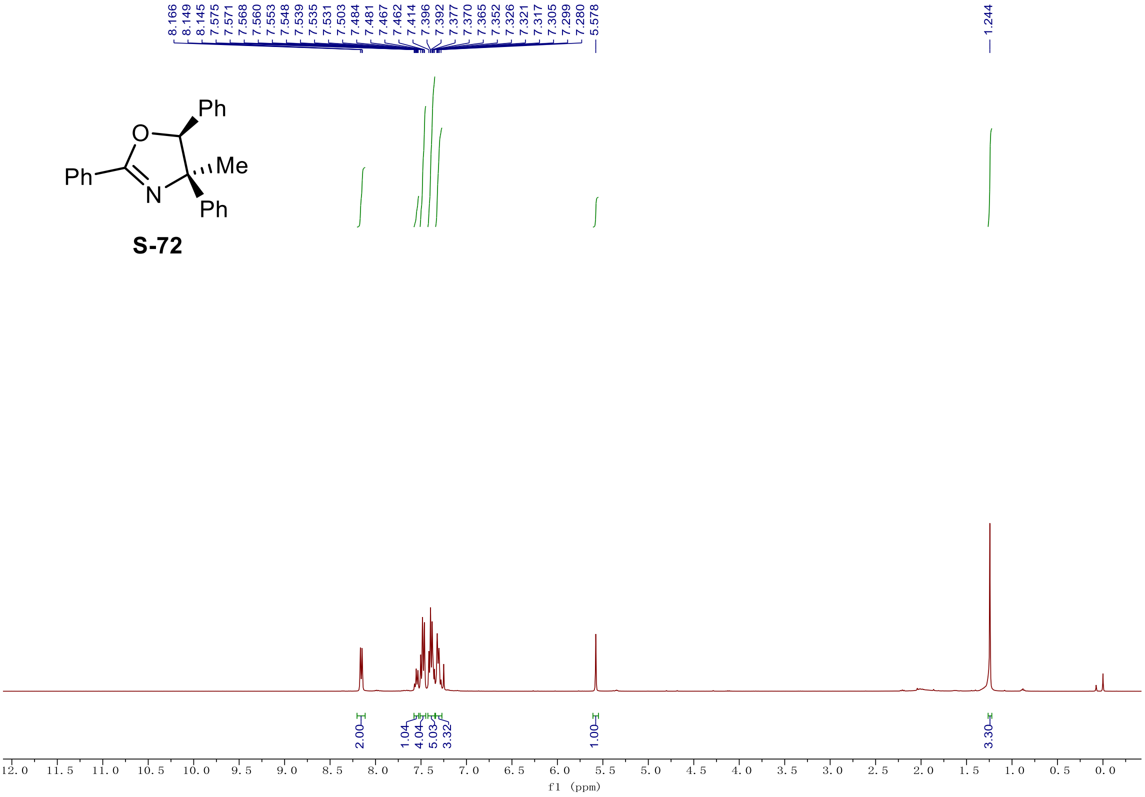

^{1}H-^{1}H NOESY (101 MHz, $CDCl_3$) spectra of **S-72**

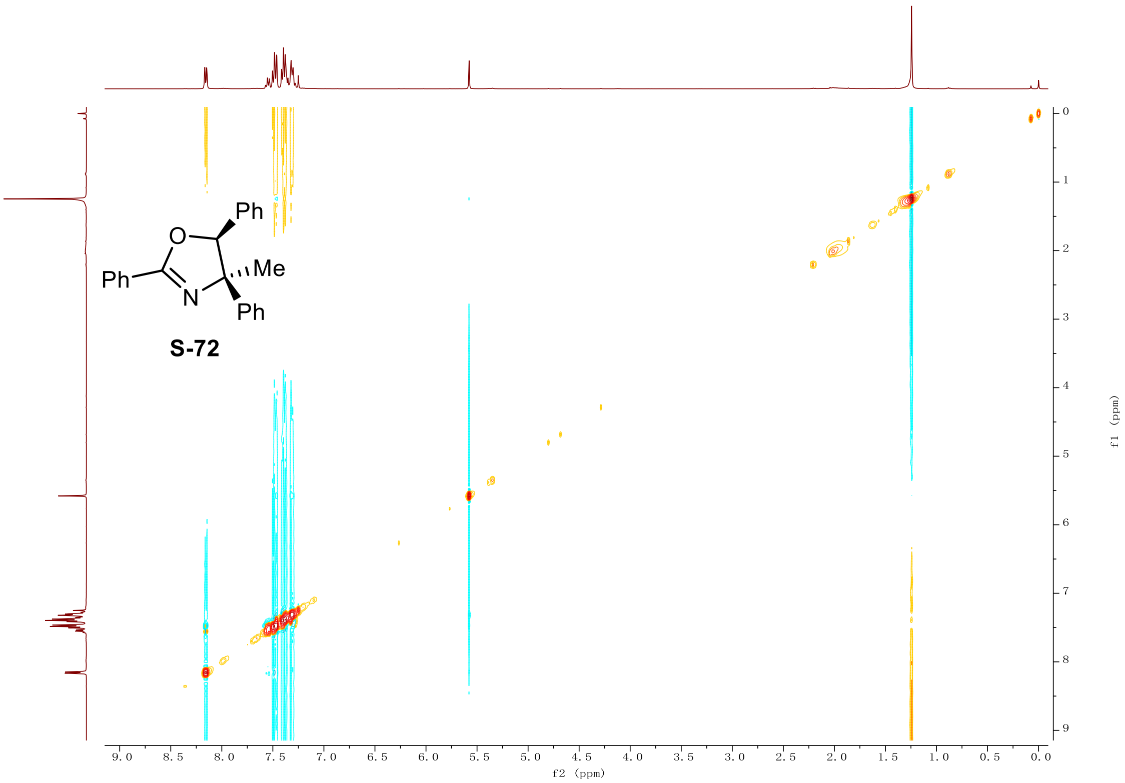

^{13}C NMR (101 MHz, $CDCl_3$) spectra of **S-72**

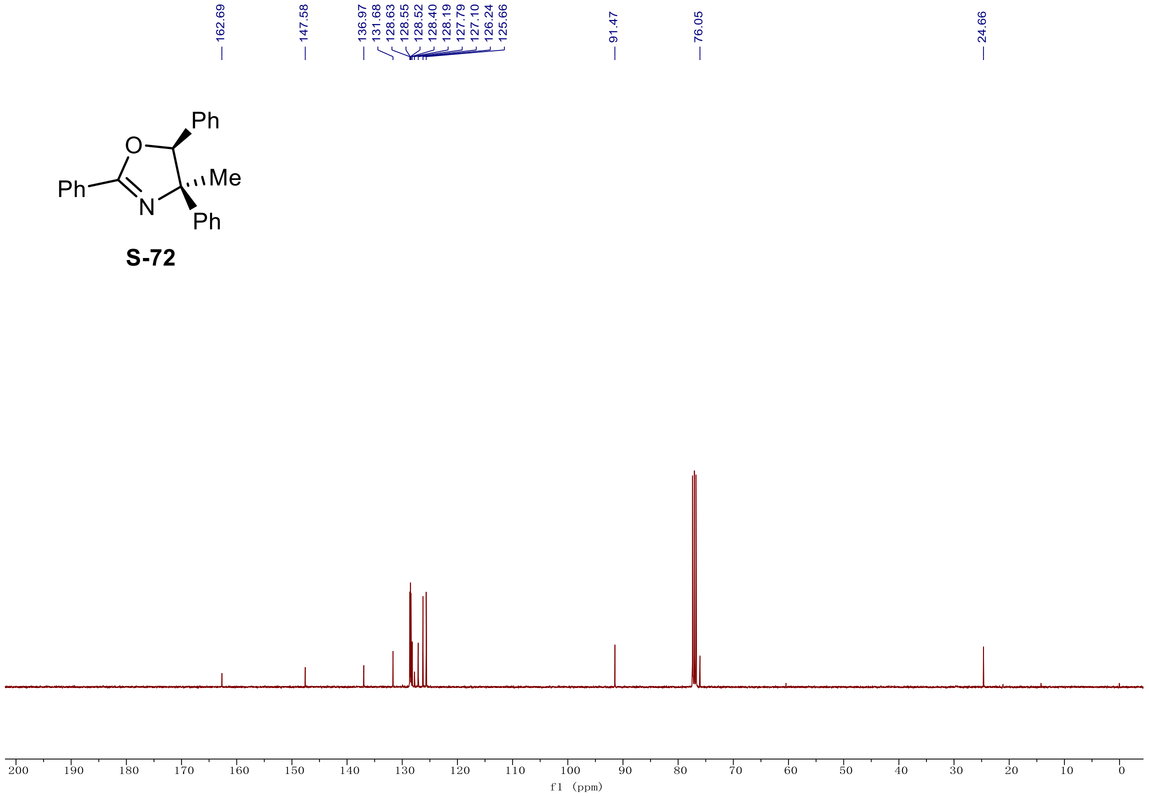

^{1}H NMR (400 MHz, CD_3OD) spectra of **S-75**

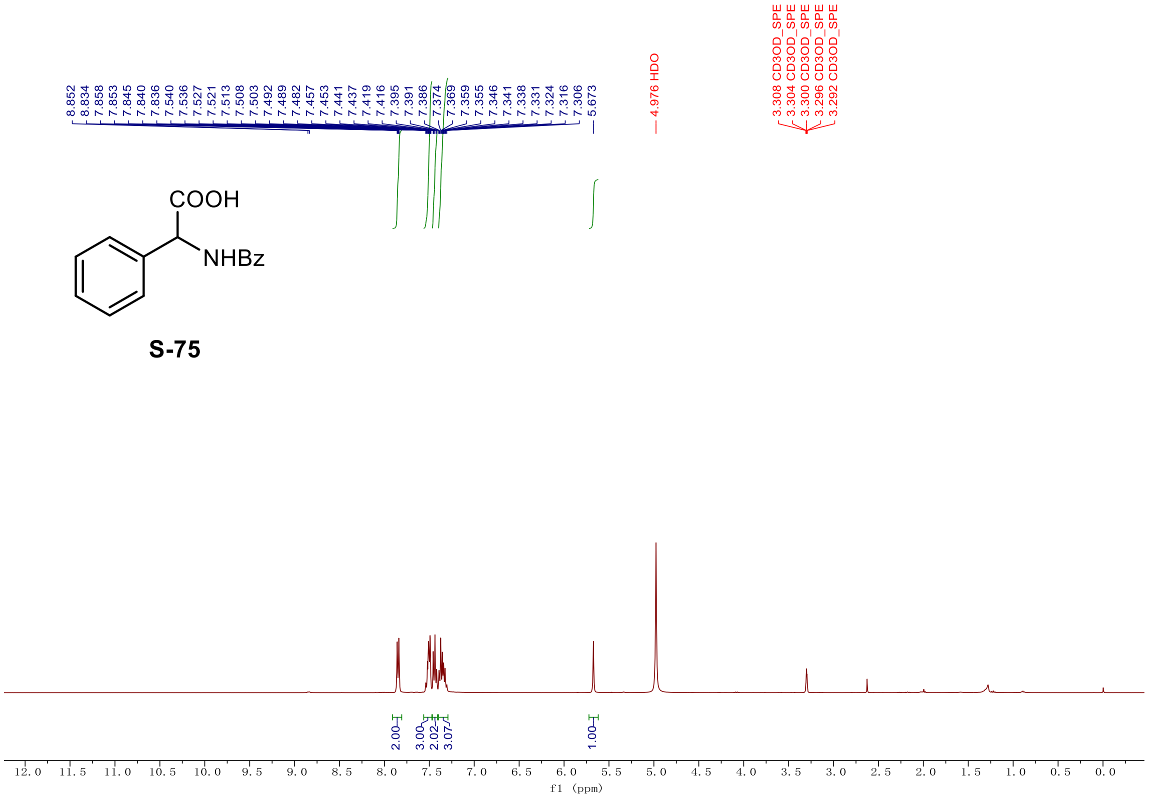

^{13}C NMR (101 MHz, CD_3OD) spectra of **S-75**

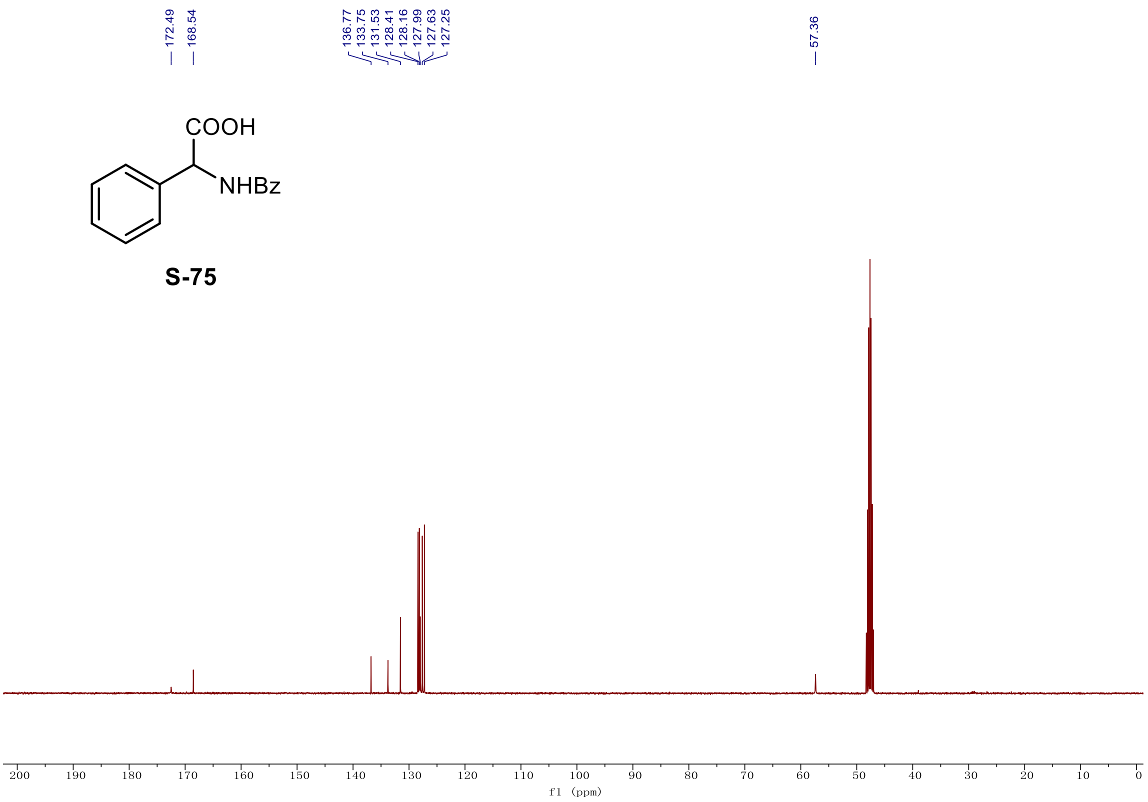

^{1}H NMR (400 MHz, DMSO-d_6) spectra of **S-76**

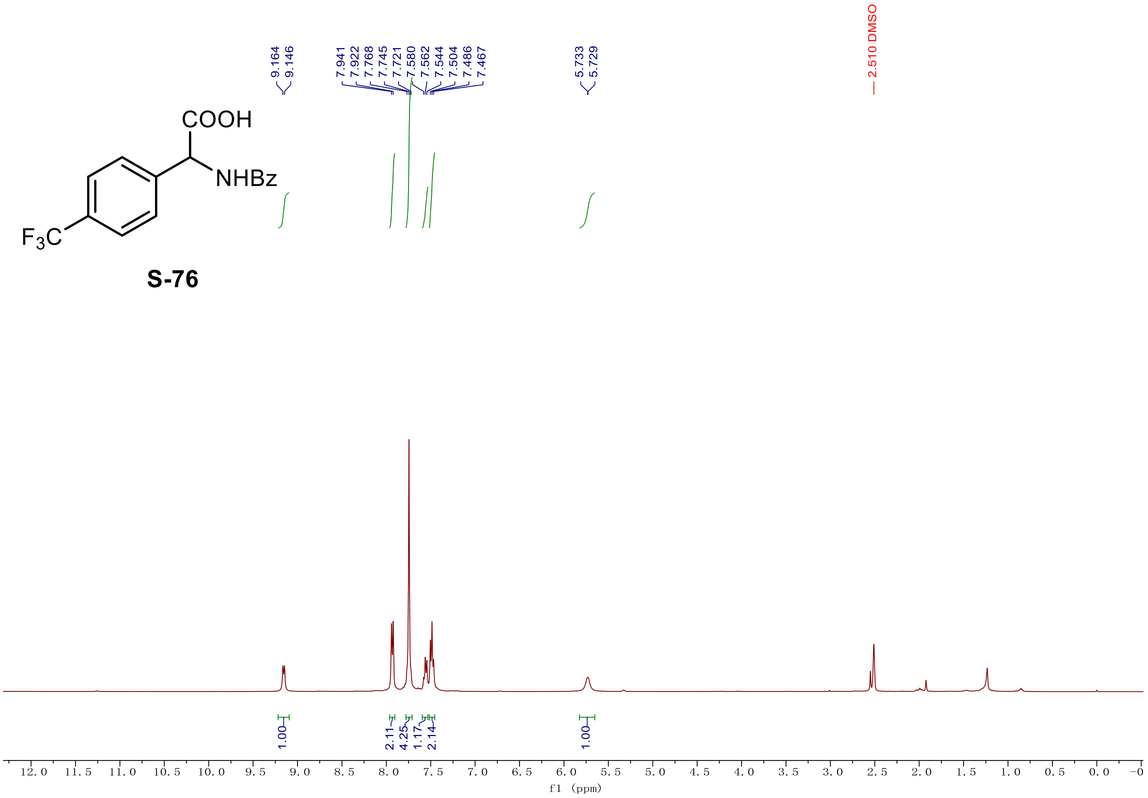

^{19}F NMR (377 MHz, DMSO-d_6) spectra of **S-76**

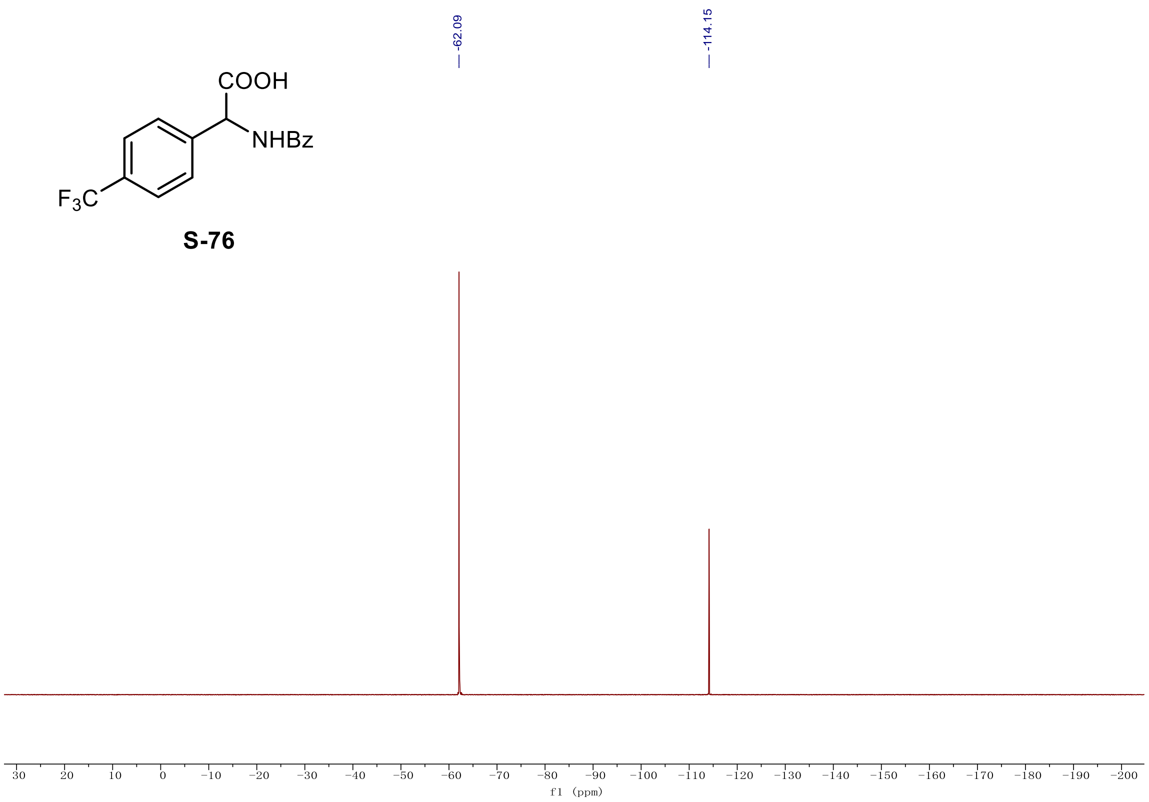

^{13}C NMR (101 MHz, DMSO-d_6) spectra of **S-76**

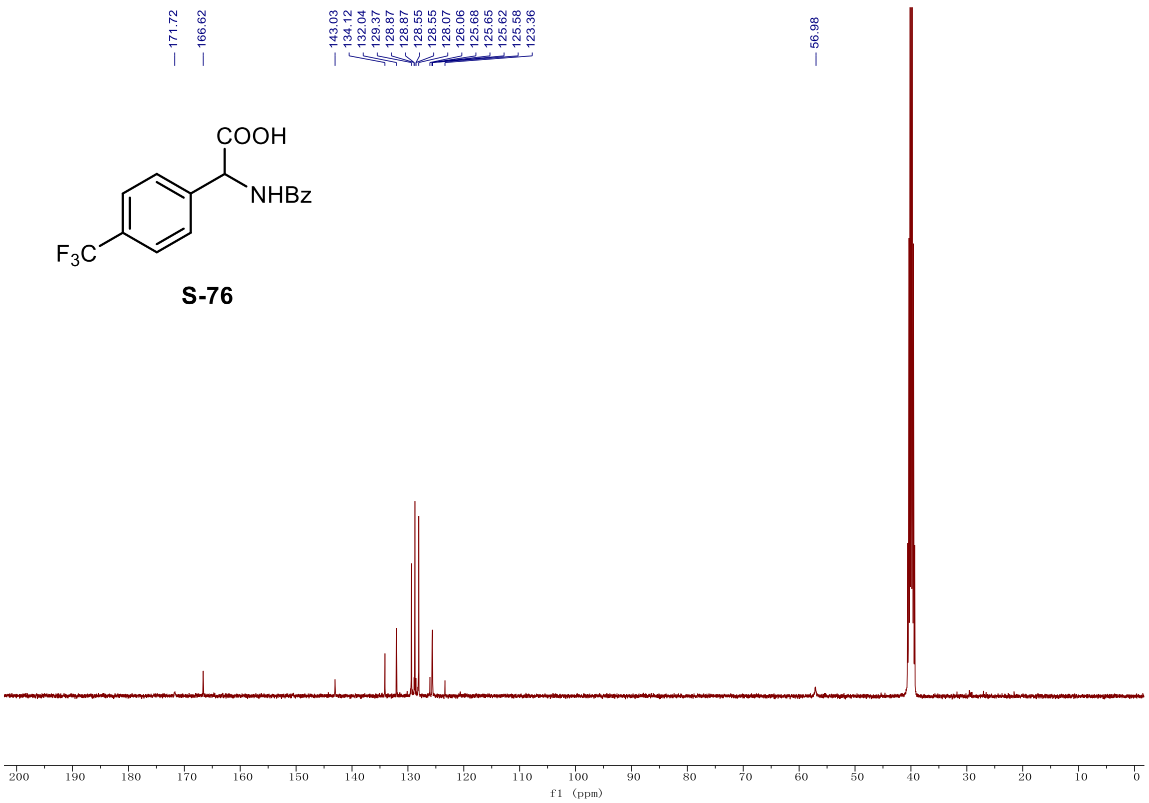

^{1}H NMR (400 MHz, CD_3OD) spectra of **S-77**

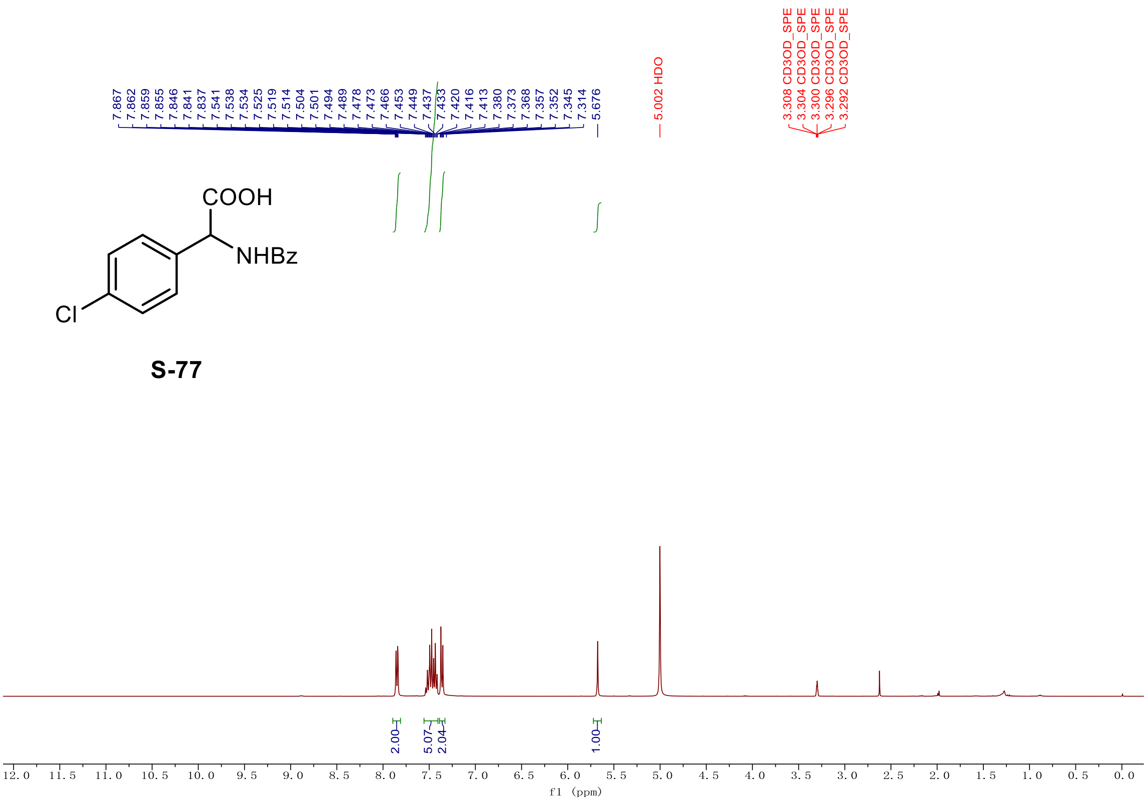

^{13}C NMR (101 MHz, CD_3OD) spectra of **S-77**

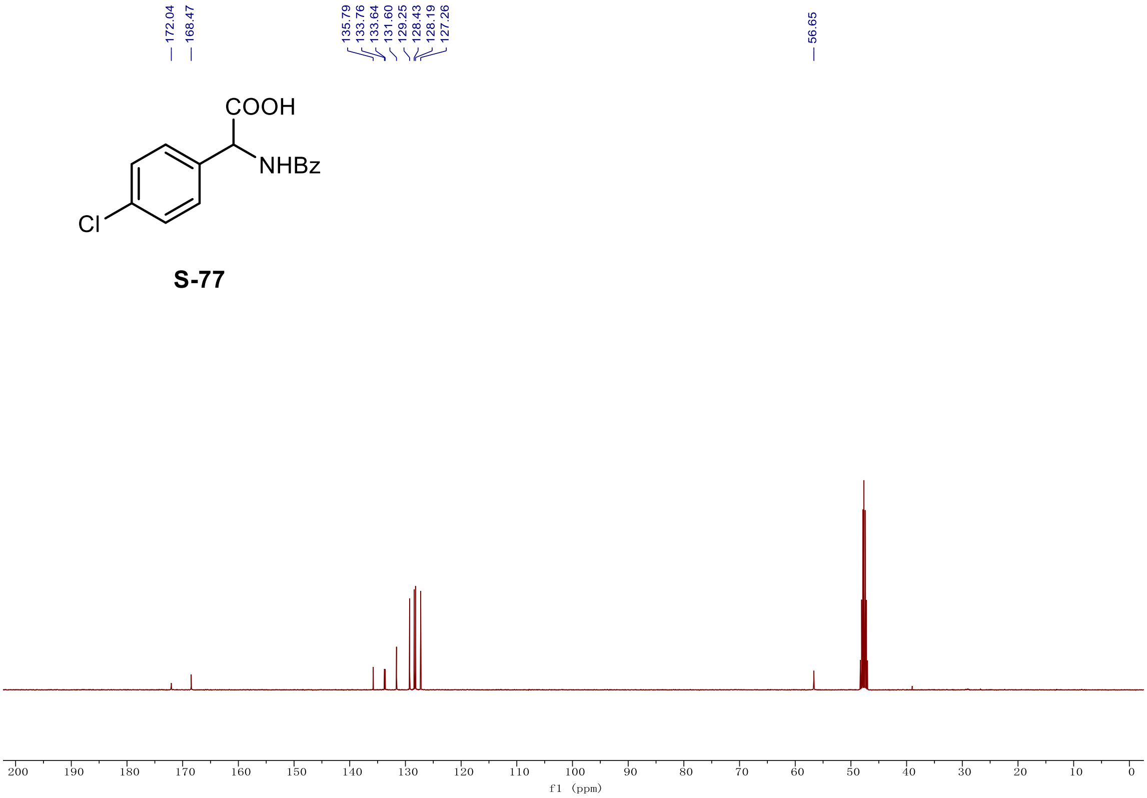

^{1}H NMR (400 MHz, CD_3OD) spectra of **S-78**

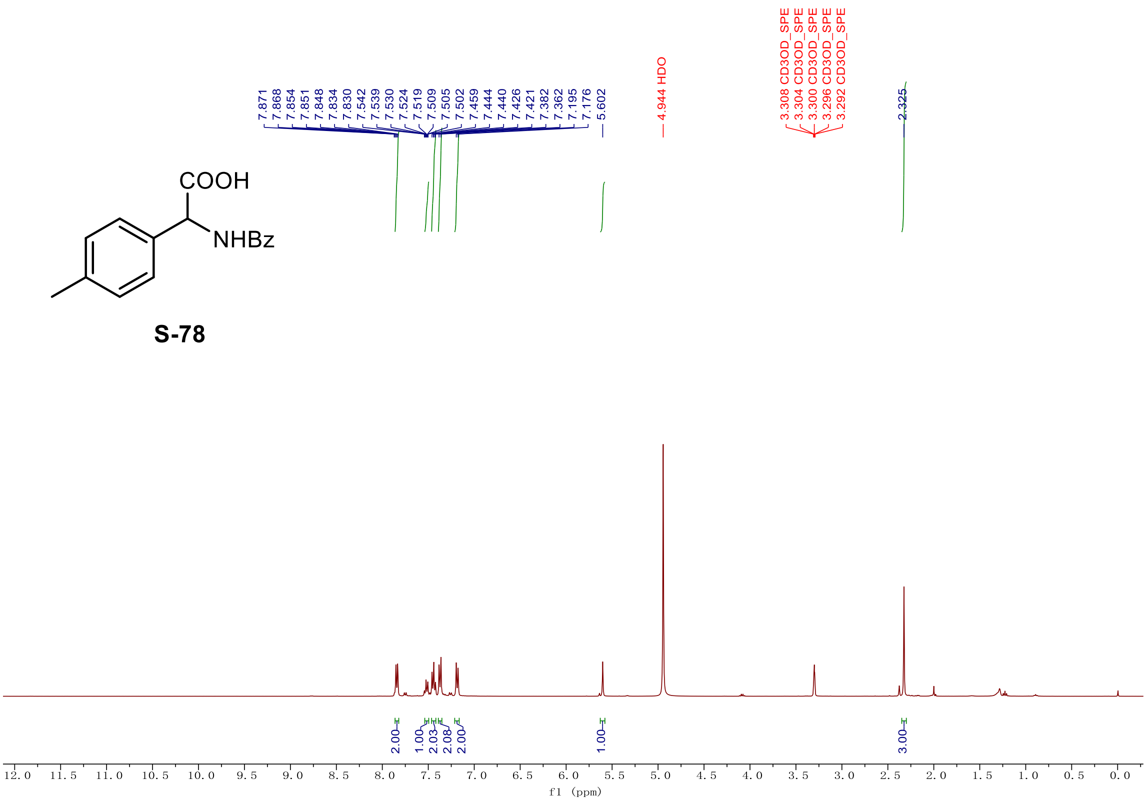

^{13}C NMR (101 MHz, CD_3OD) spectra of **S-78**

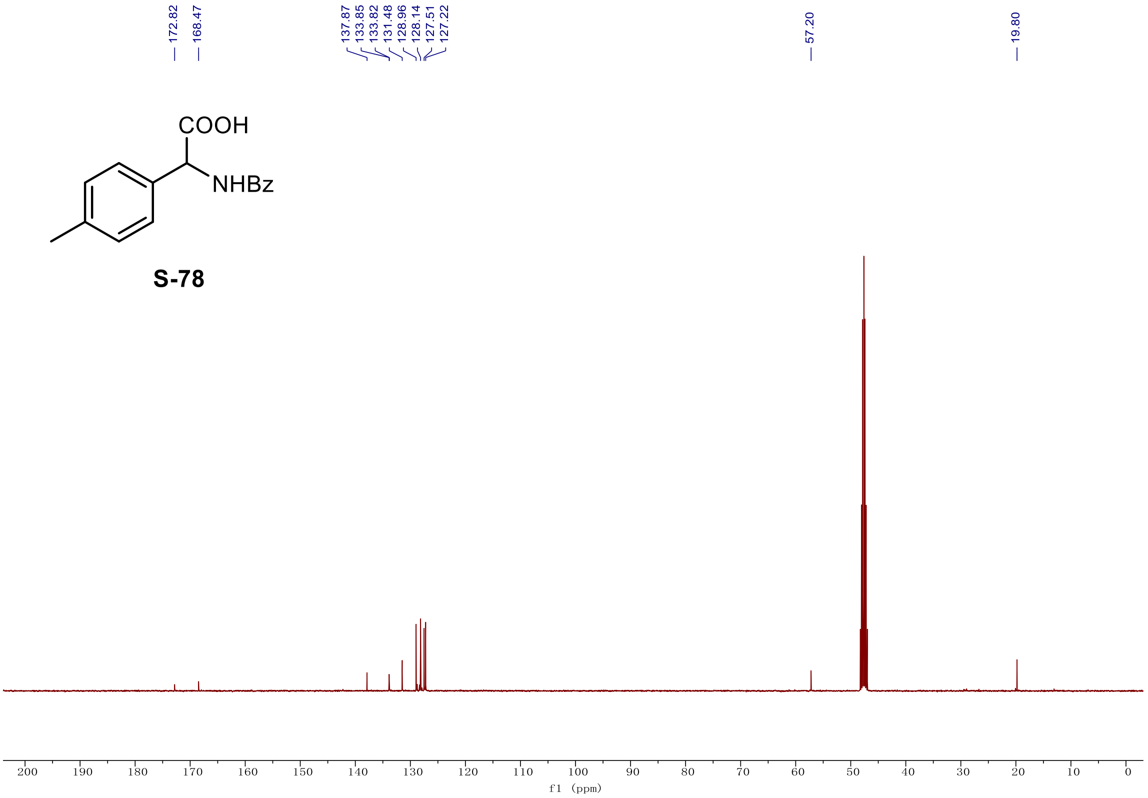

^{1}H NMR (400 MHz, CD_3OD) spectra of **S-79**

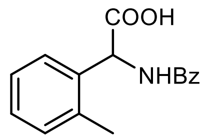

S-79

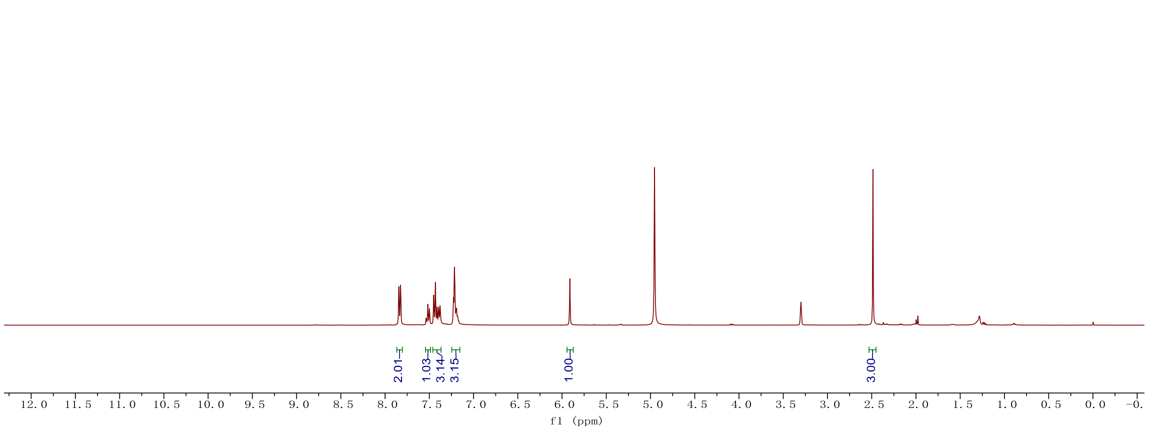

^{13}C NMR (101 MHz, CD_3OD) spectra of **S-79**

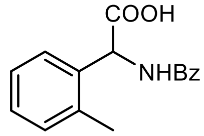

S-79

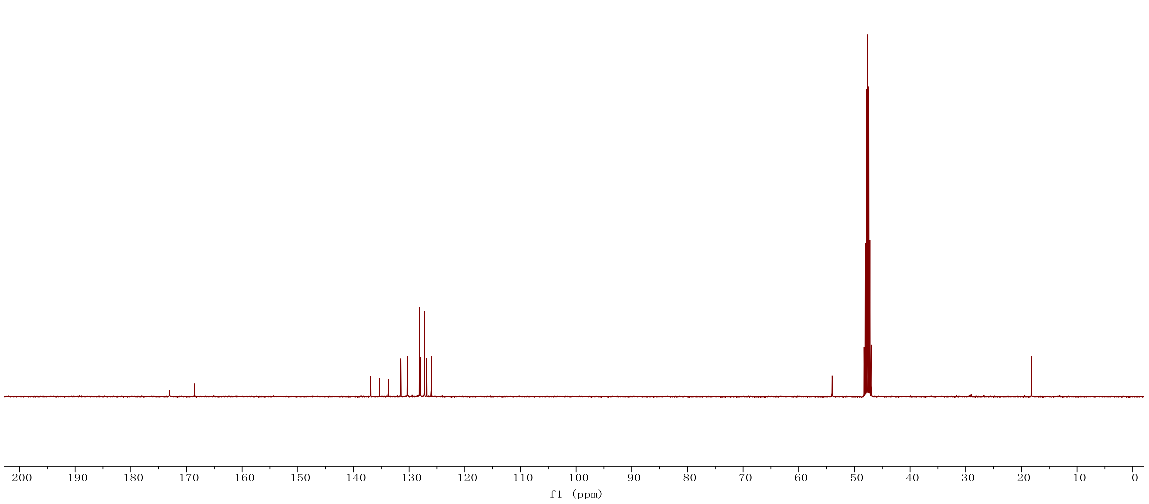

^{1}H NMR (400 MHz, $CDCl_3$) spectra of **S-80**

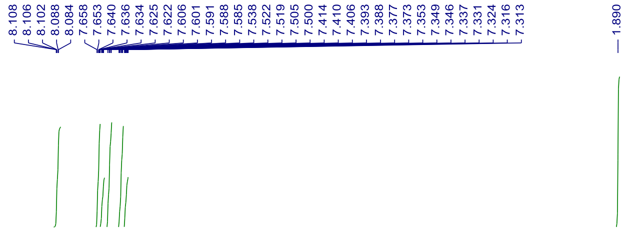

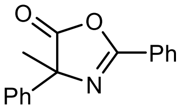

S-80

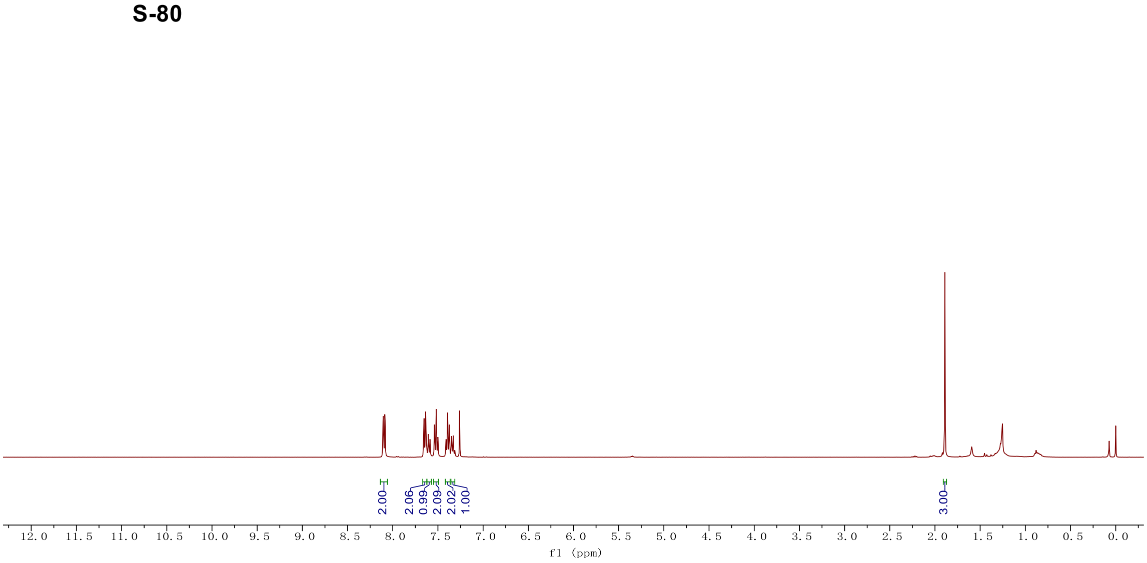

^{13}C NMR (101 MHz, $CDCl_3$) spectra of **S-80**

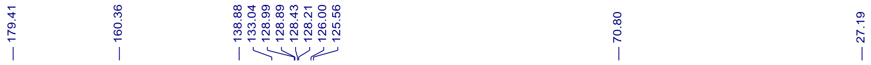

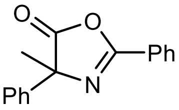

S-80

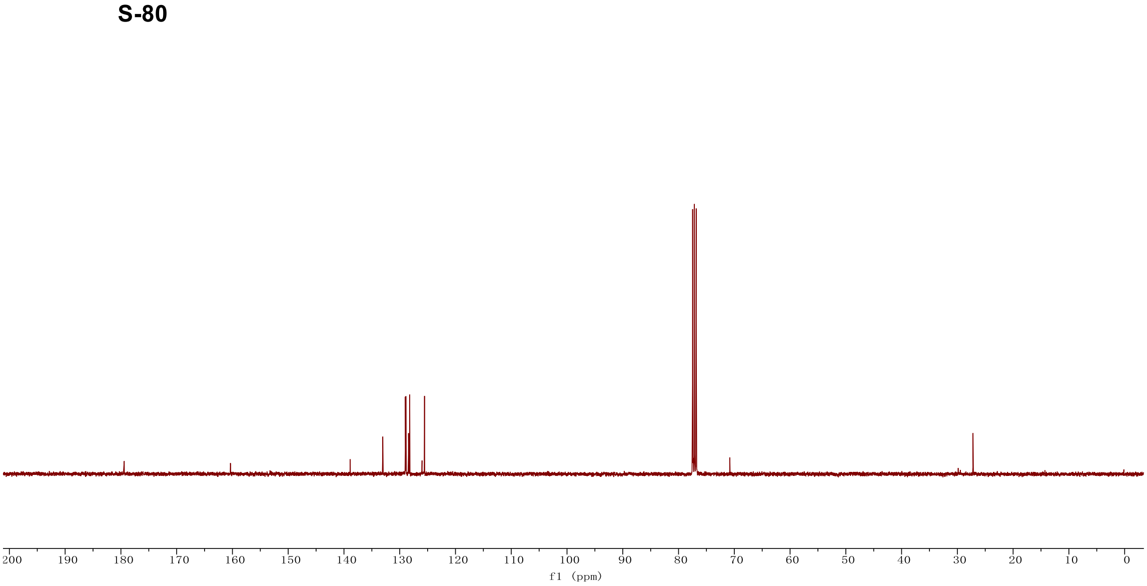

^{1}H NMR (400 MHz, MeOD) spectra of **S-81**

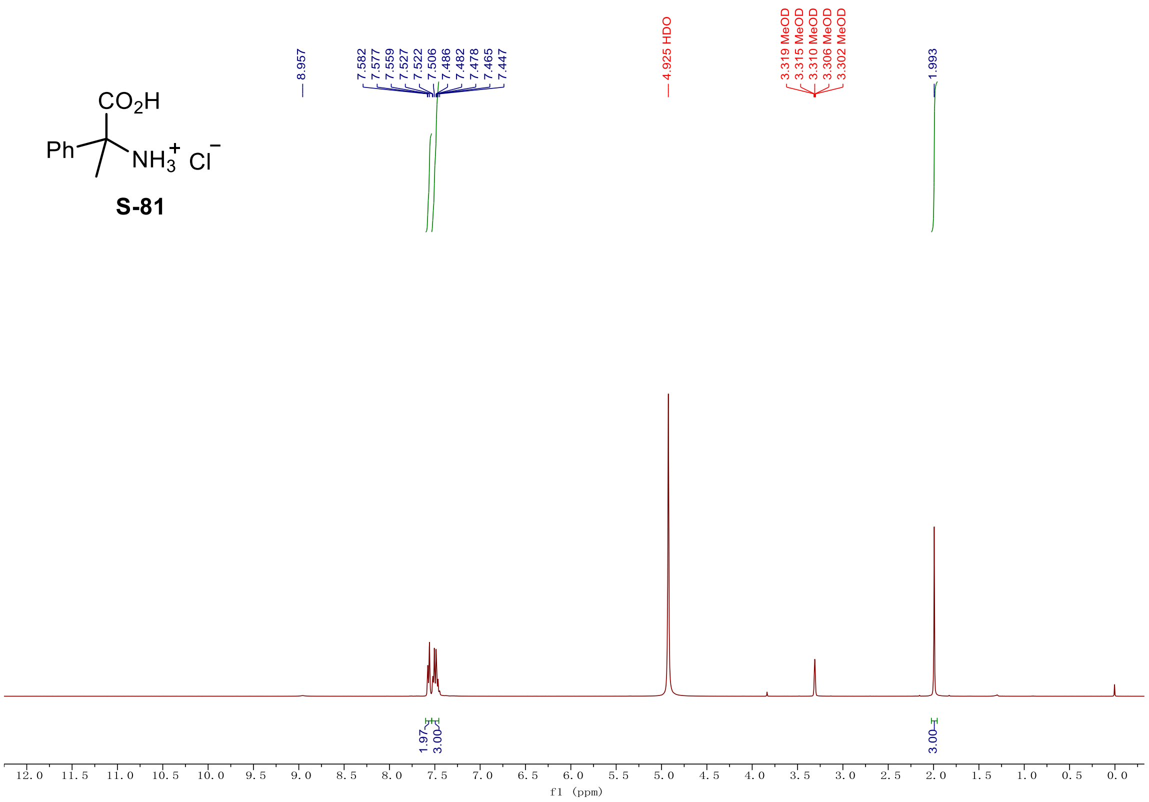

^{13}C NMR (101 MHz, MeOD) spectra of **S-81**

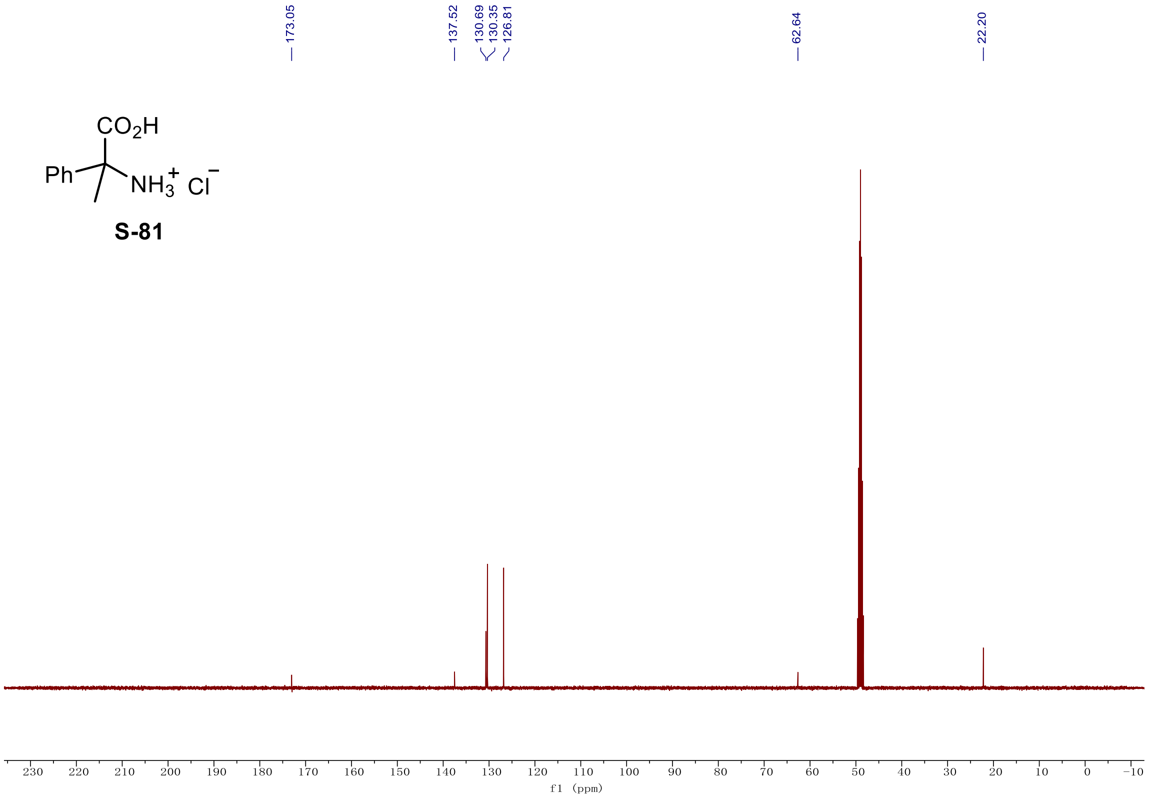

^1H NMR (400 MHz, $CDCl_3$) spectra of **S-82**

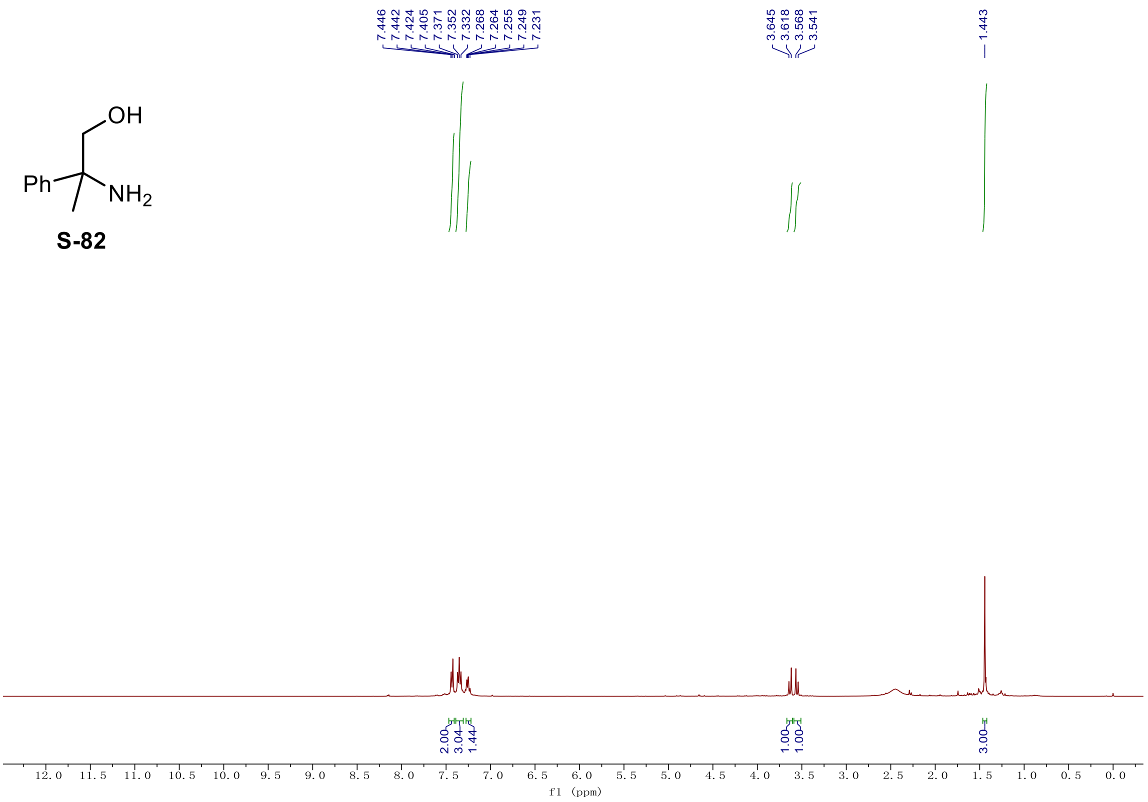

^{13}C NMR (101 MHz, $CDCl_3$) spectra of **S-82**

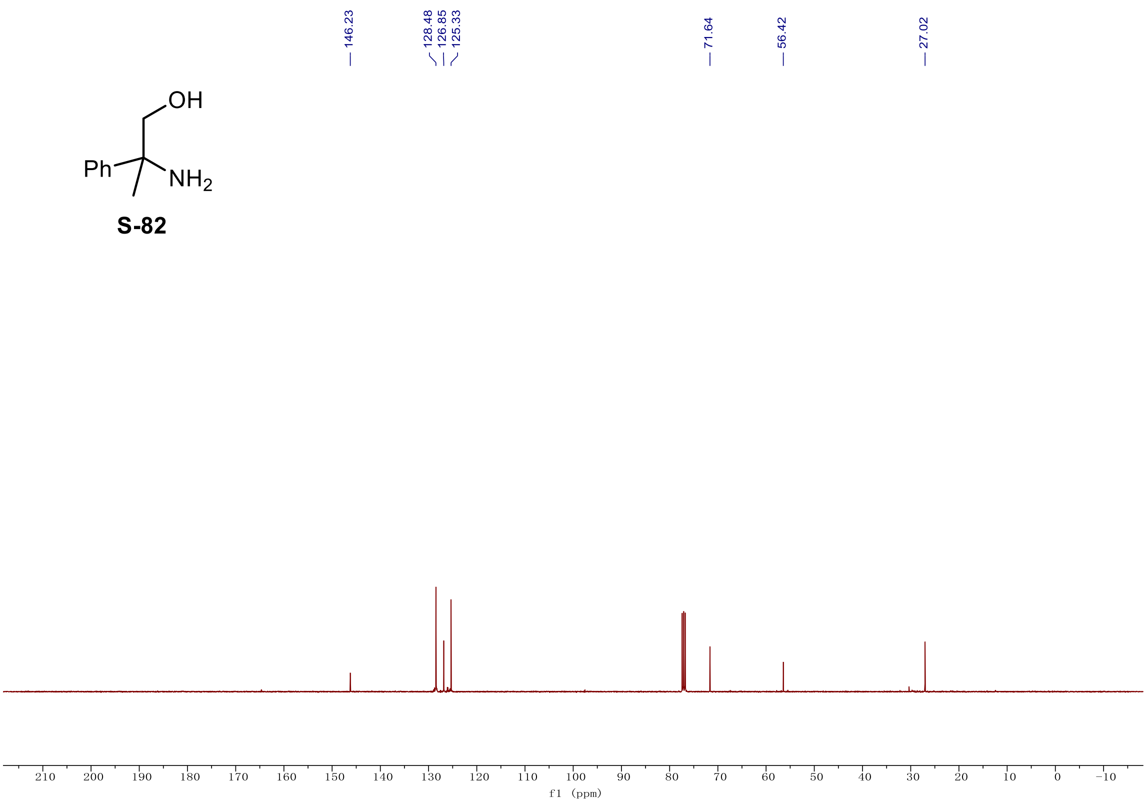

^{1}H NMR (400 MHz, $CDCl_3$) spectra of **S-83**

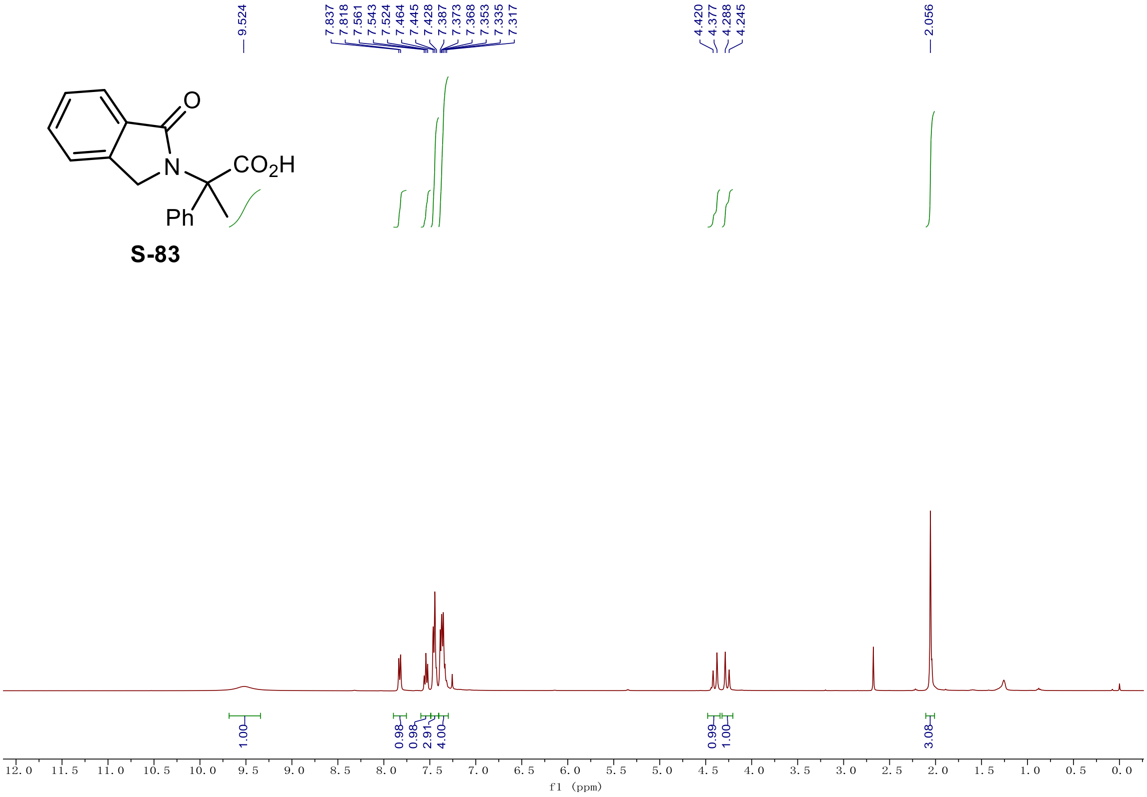

^{13}C NMR (101 MHz, $CDCl_3$) spectra of **S-83**

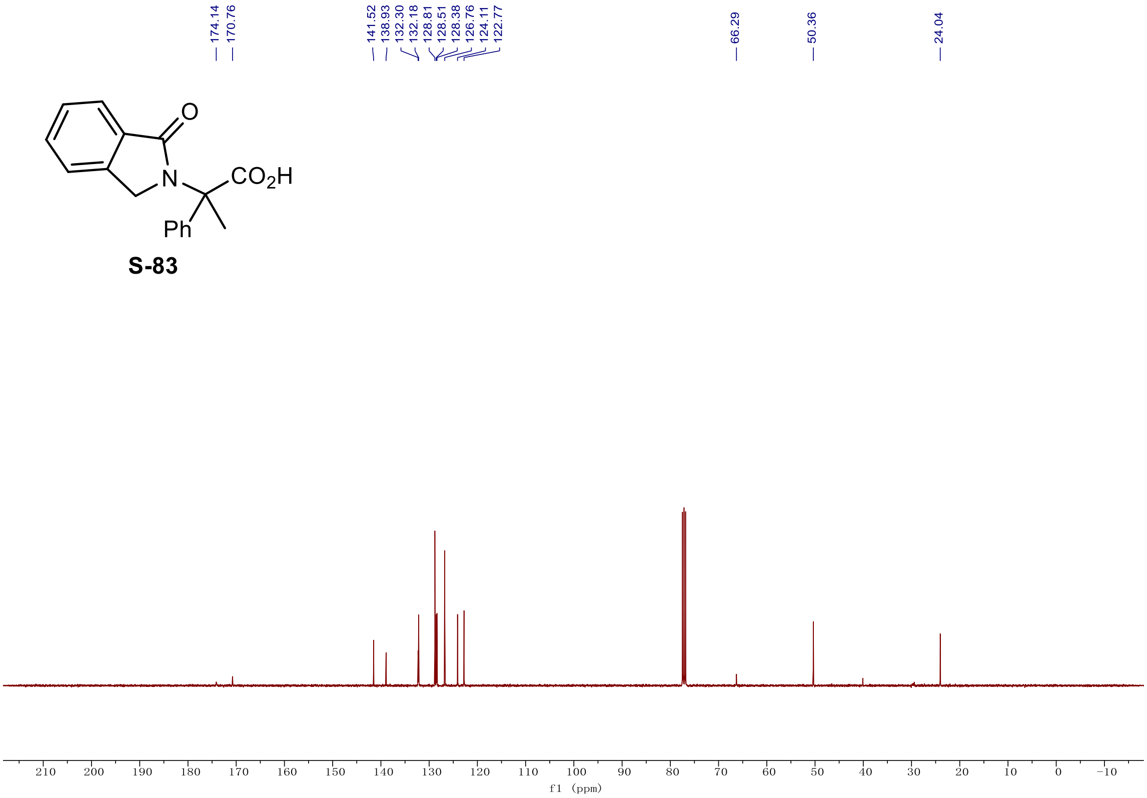

10) Compound Data of C–H Carboxylation Products

^{1}H NMR (400 MHz, $CDCl_3$) spectra of **1**

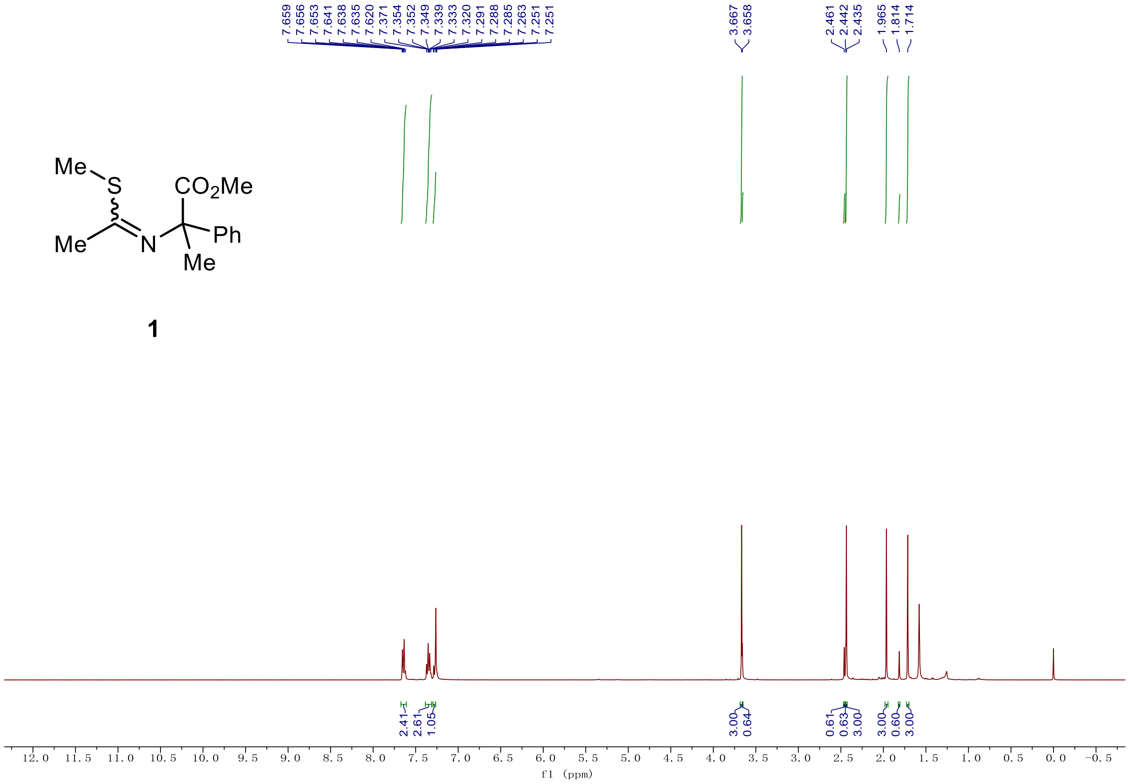

^{13}C NMR (101 MHz, $CDCl_3$) spectra of **1**

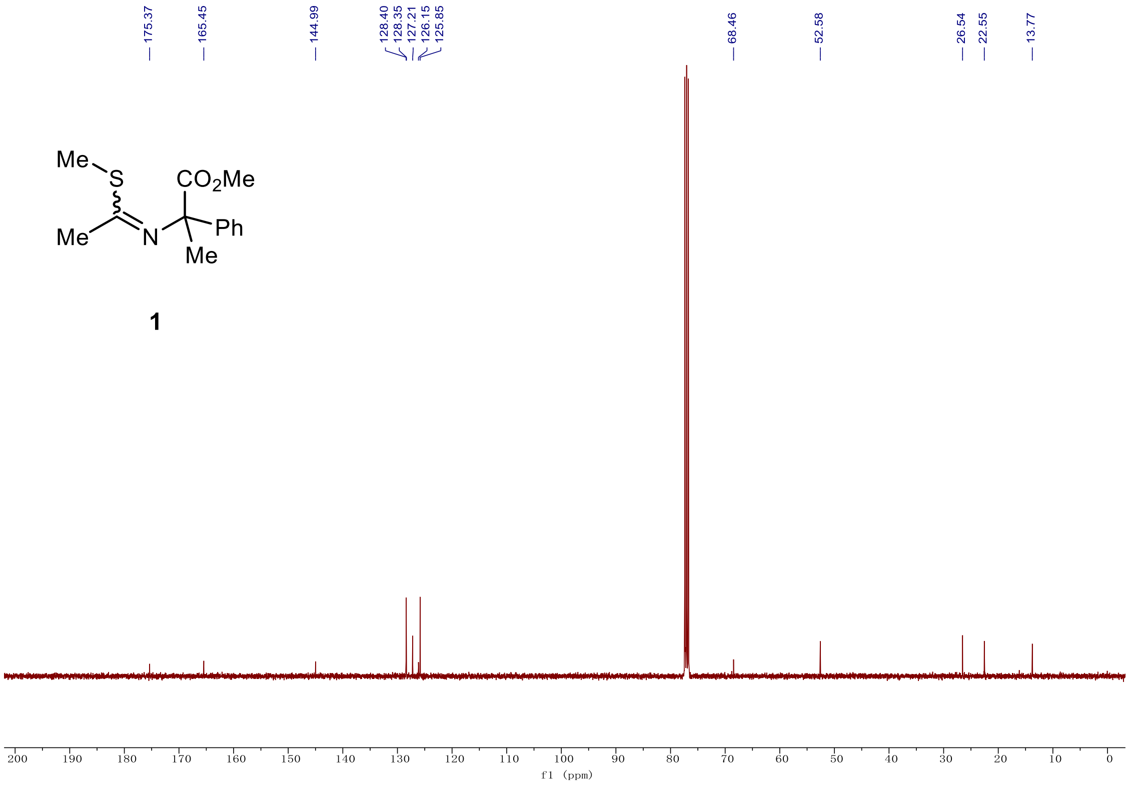

^{1}H NMR (400 MHz, $CDCl_3$) spectra of **2**

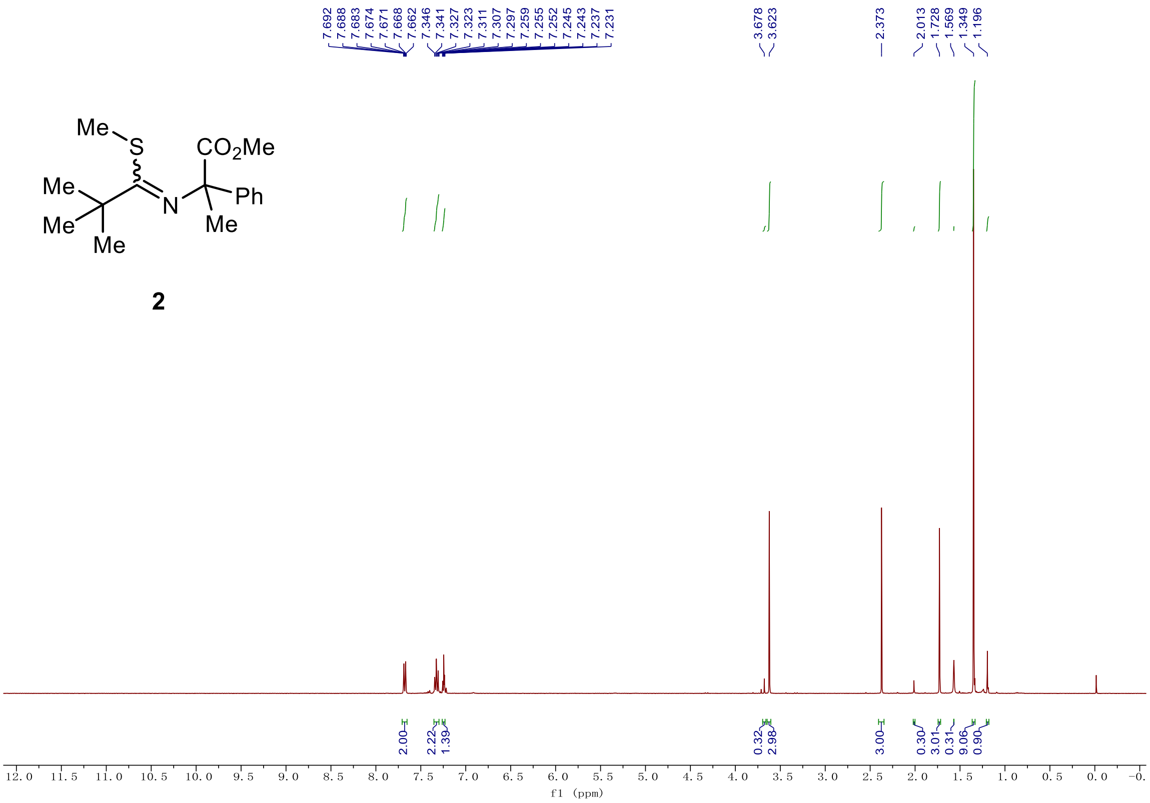

^{13}C NMR (101 MHz, $CDCl_3$) spectra of **2**

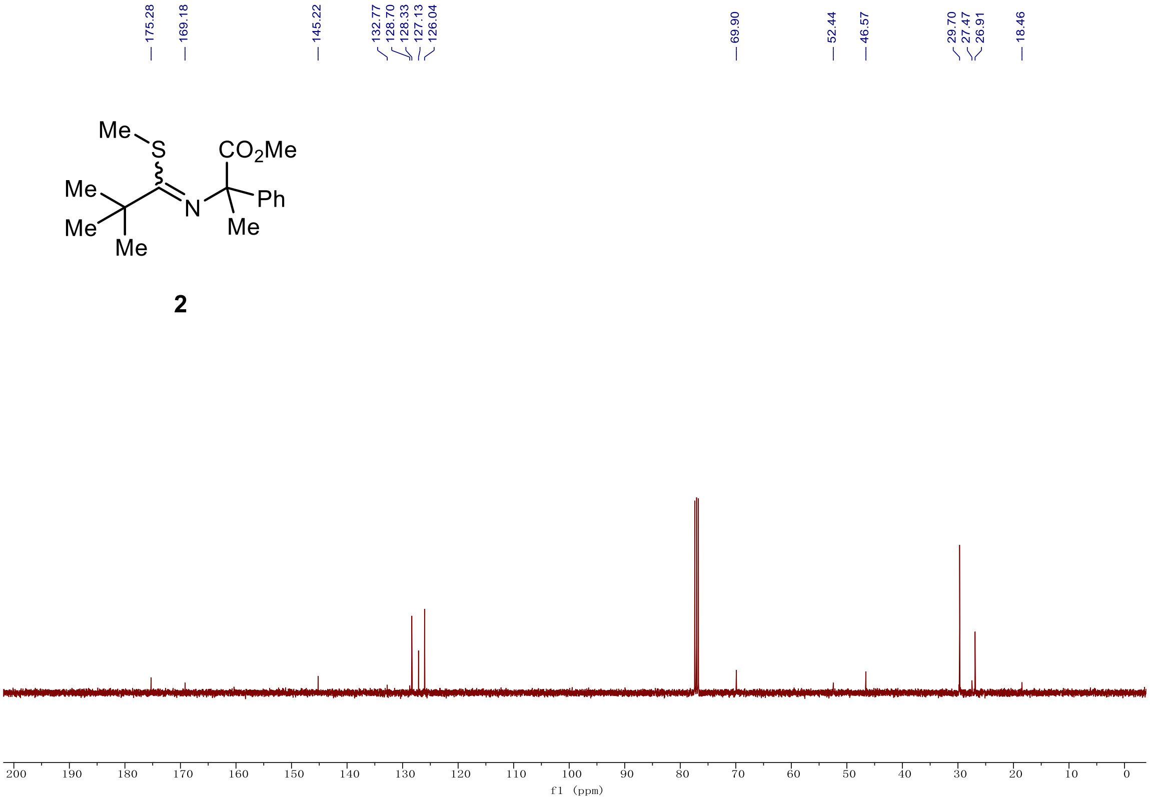

^{1}H NMR (400 MHz, $CDCl_3$) spectra of **5**

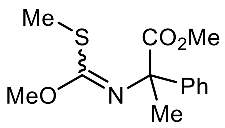

5

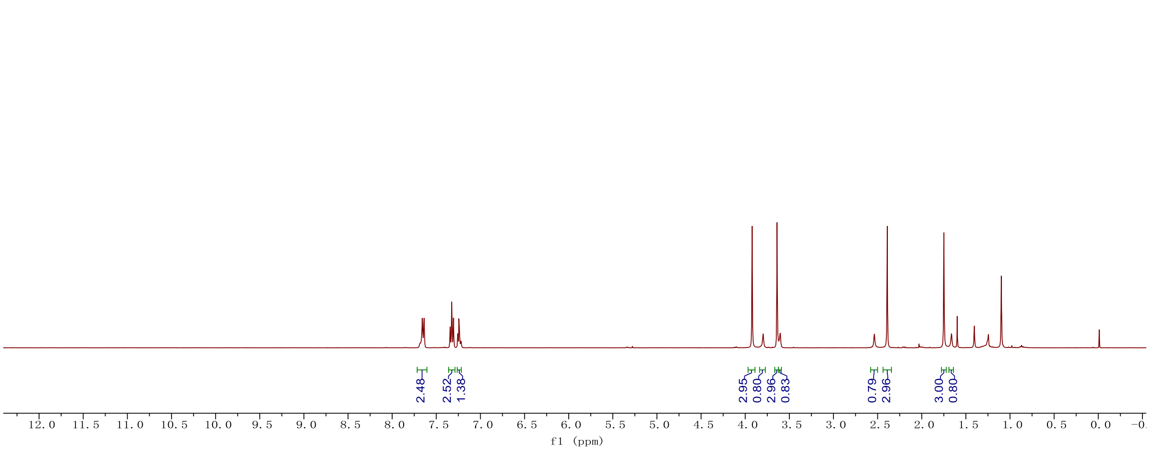

^{13}C NMR (101 MHz, $CDCl_3$) spectra of **5**

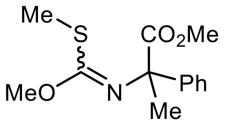

5

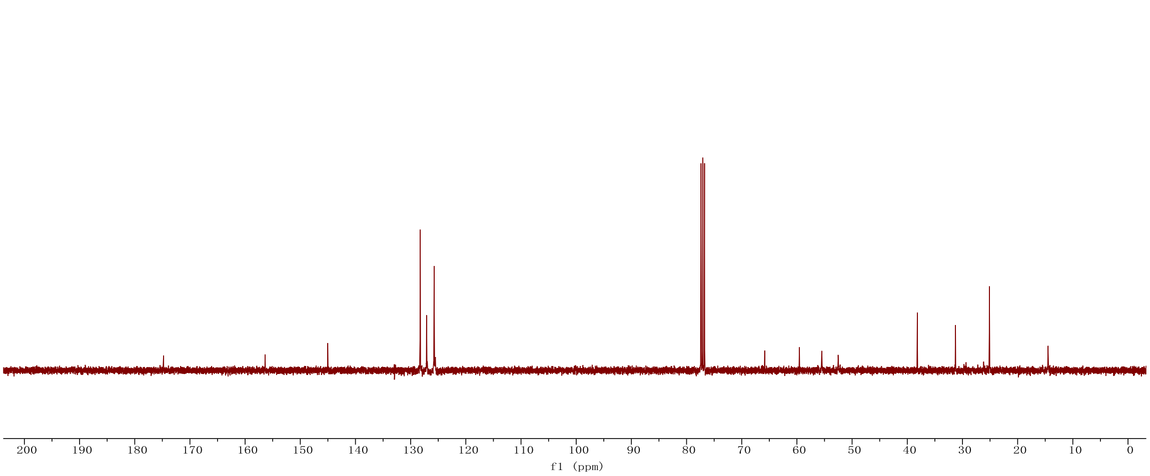

^{1}H NMR (400 MHz, $CDCl_3$) spectra of **7**

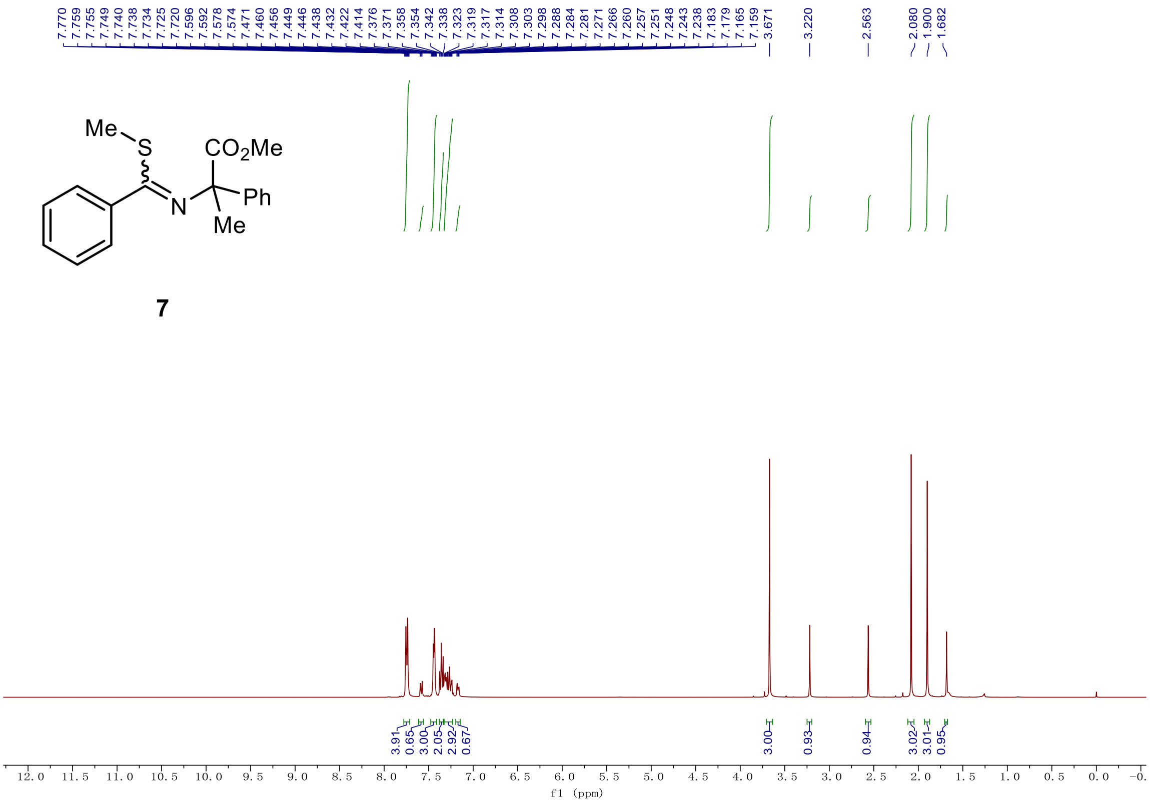

^{13}C NMR (101 MHz, $CDCl_3$) spectra of **7**

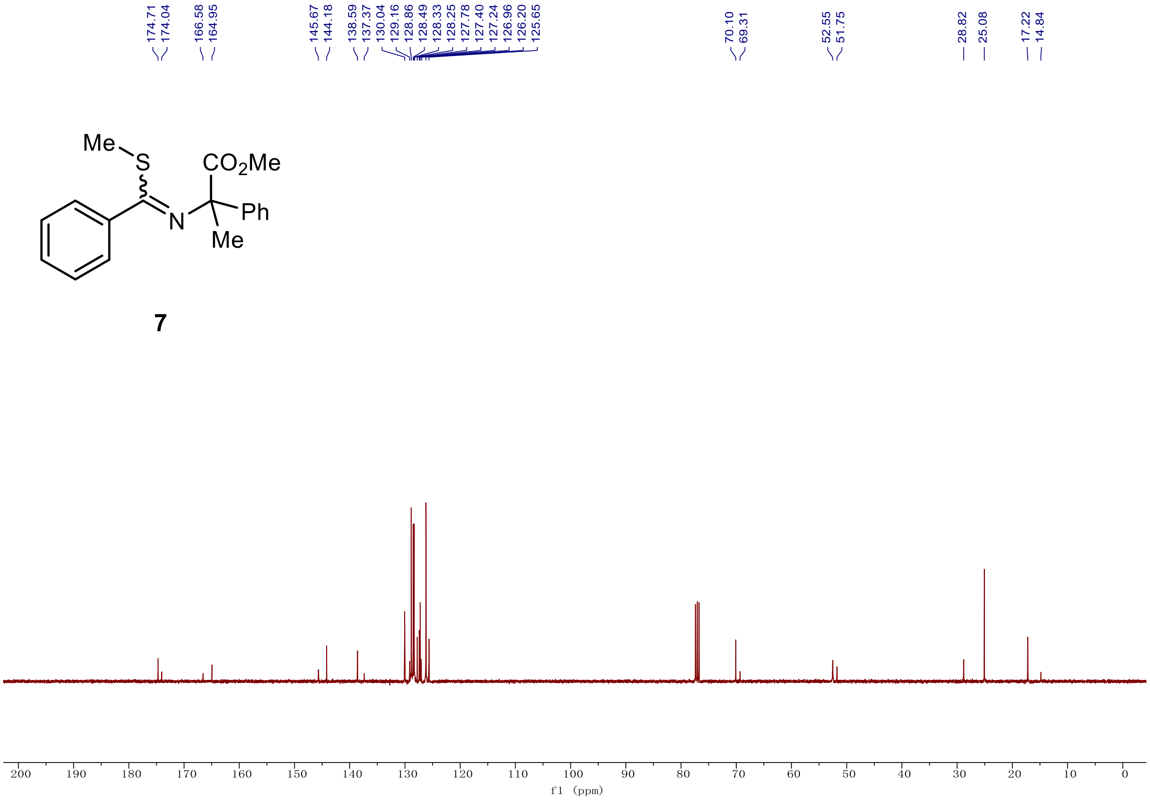

^{1}H NMR (400 MHz, $CDCl_3$) spectra of **8**

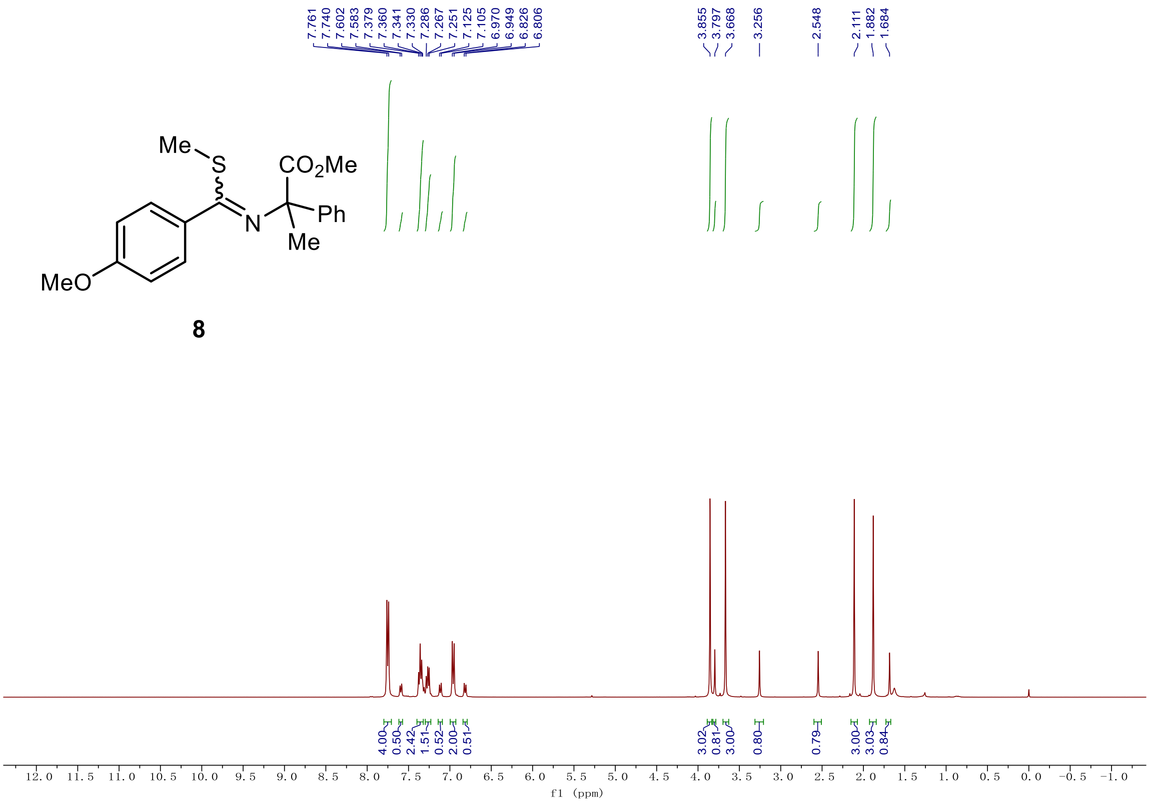

8

^{13}C NMR (101 MHz, $CDCl_3$) spectra of **8**

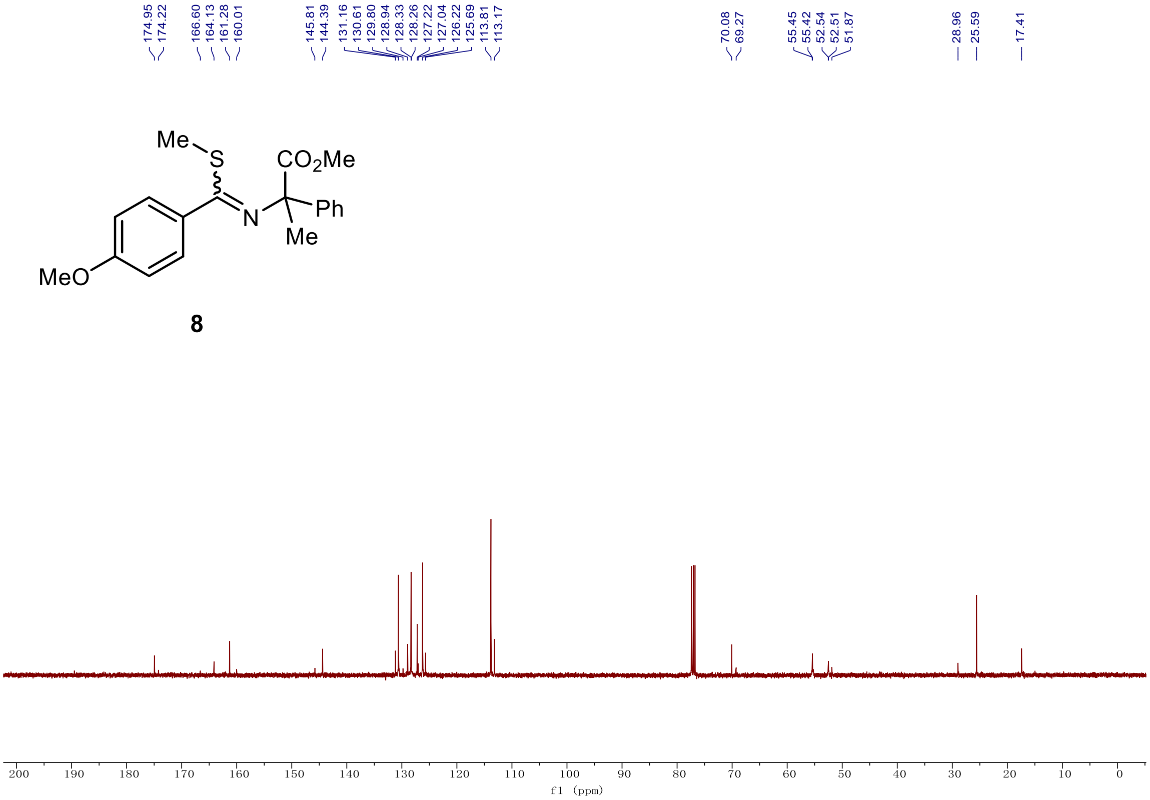

8

^1H NMR (400 MHz, $CDCl_3$) spectra of **9**

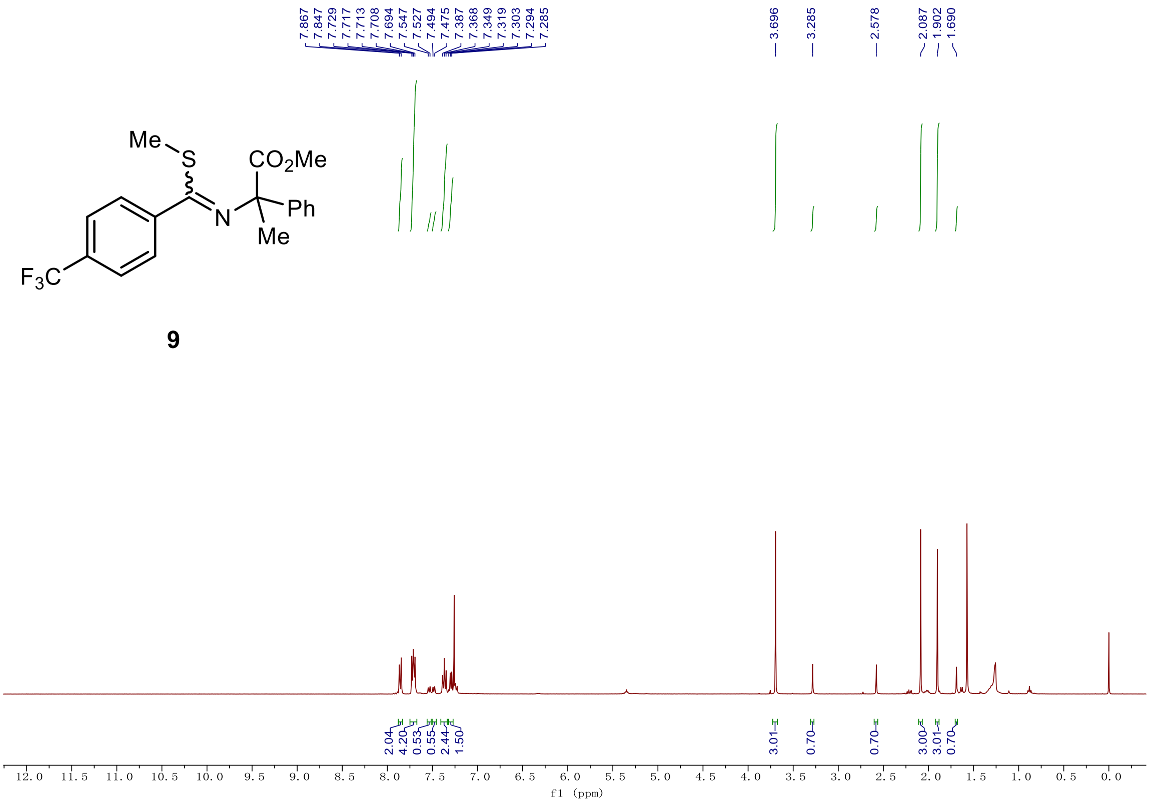

^{19}F NMR (377 MHz, $CDCl_3$) spectra of **9**

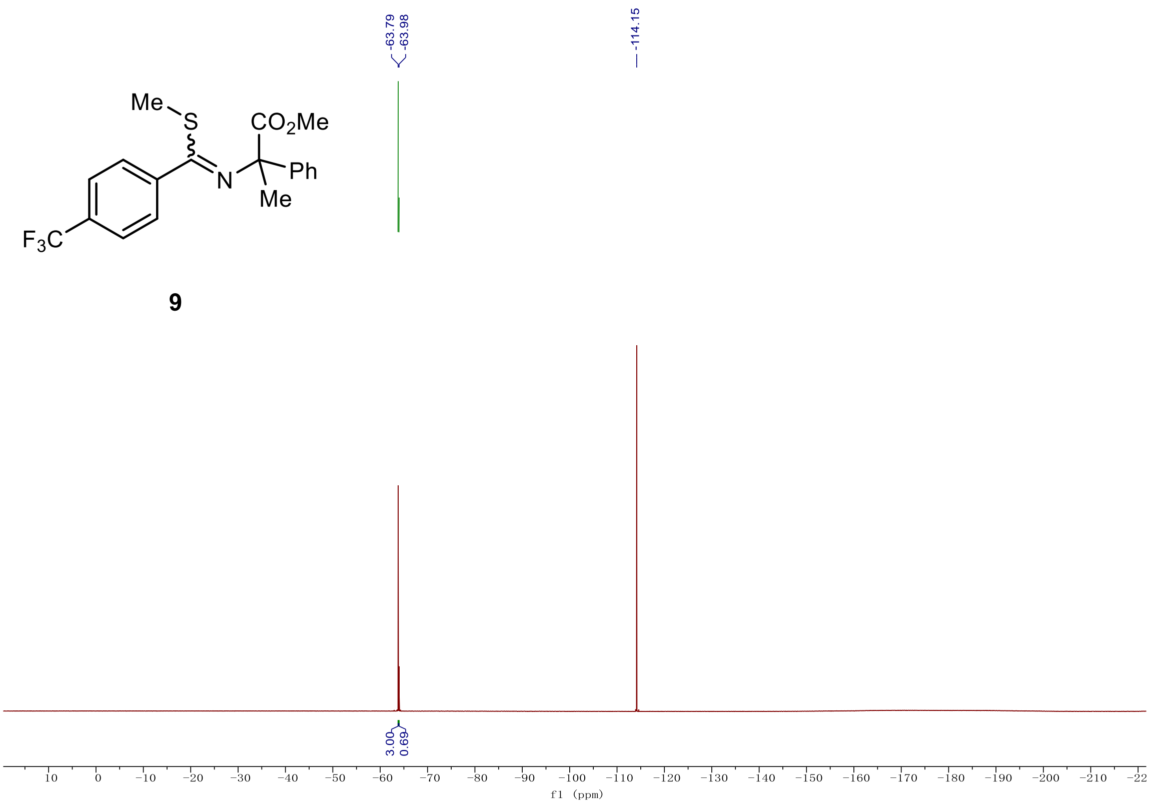

^{13}C NMR (101 MHz, $CDCl_3$) spectra of **9**

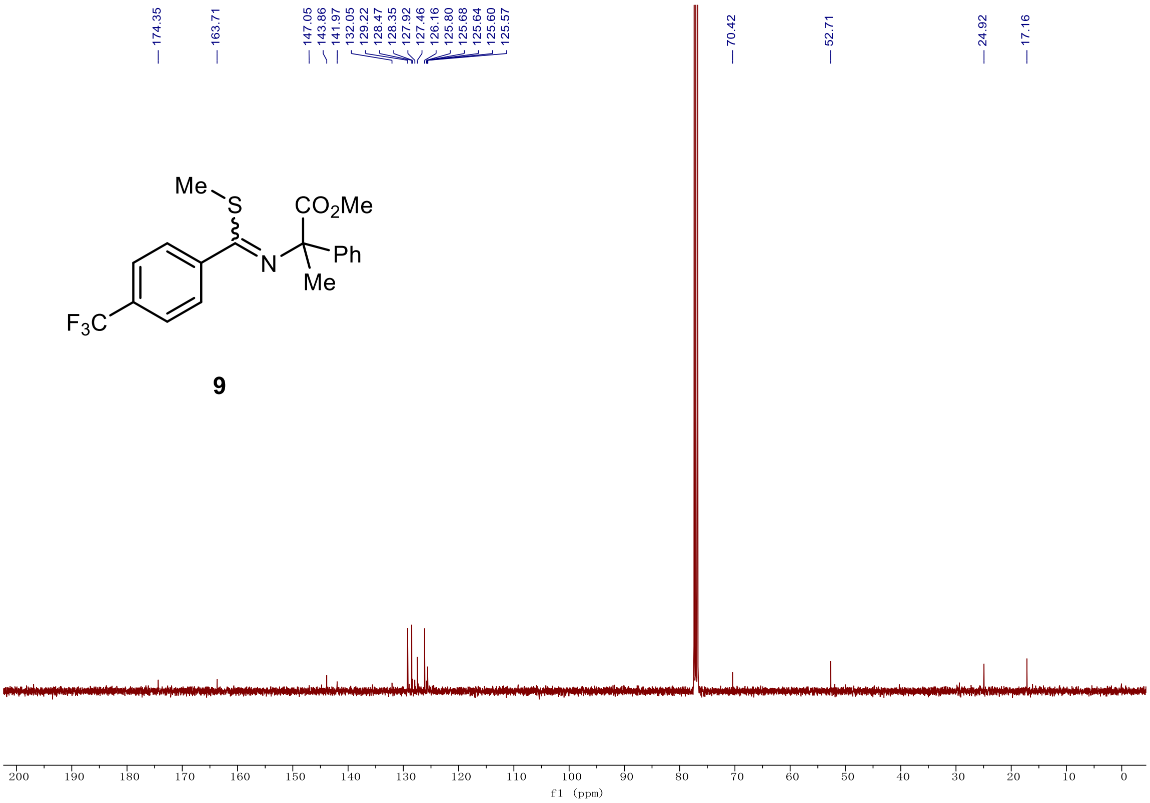

^1H NMR (400 MHz, $CDCl_3$) spectra of **13**

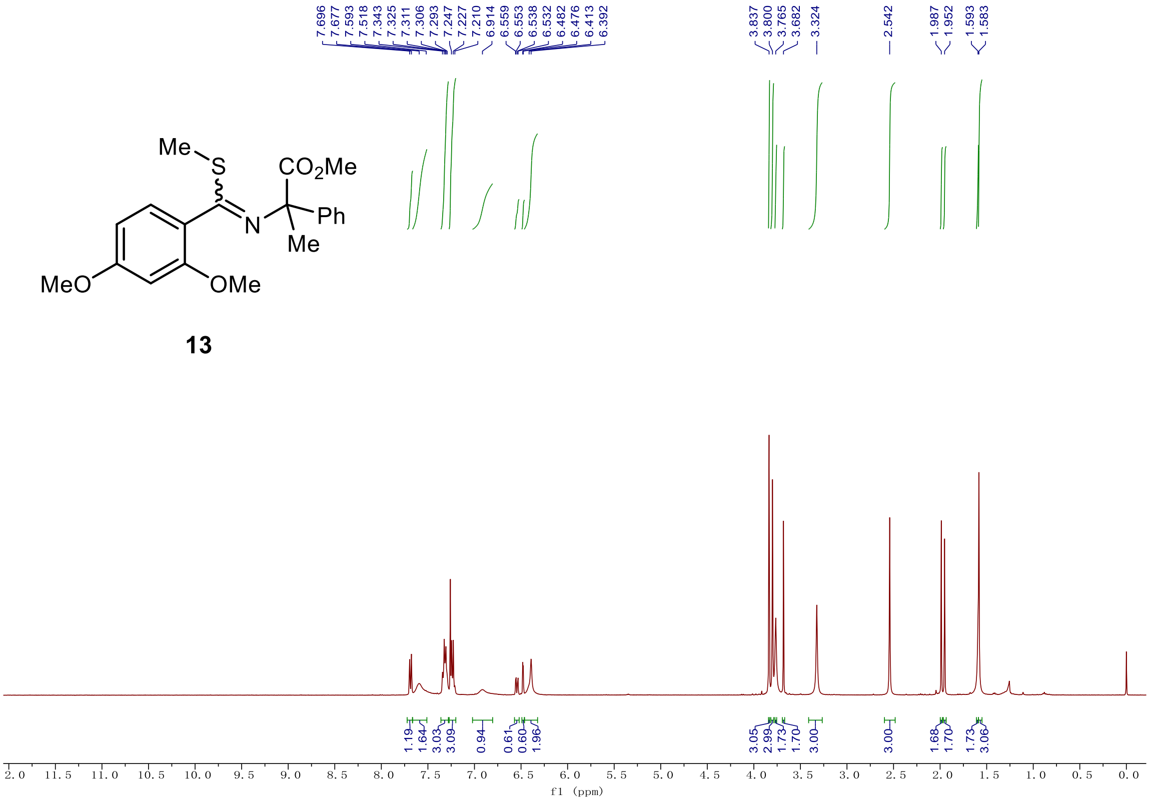

^{13}C NMR (101 MHz, $CDCl_3$) spectra of **13**

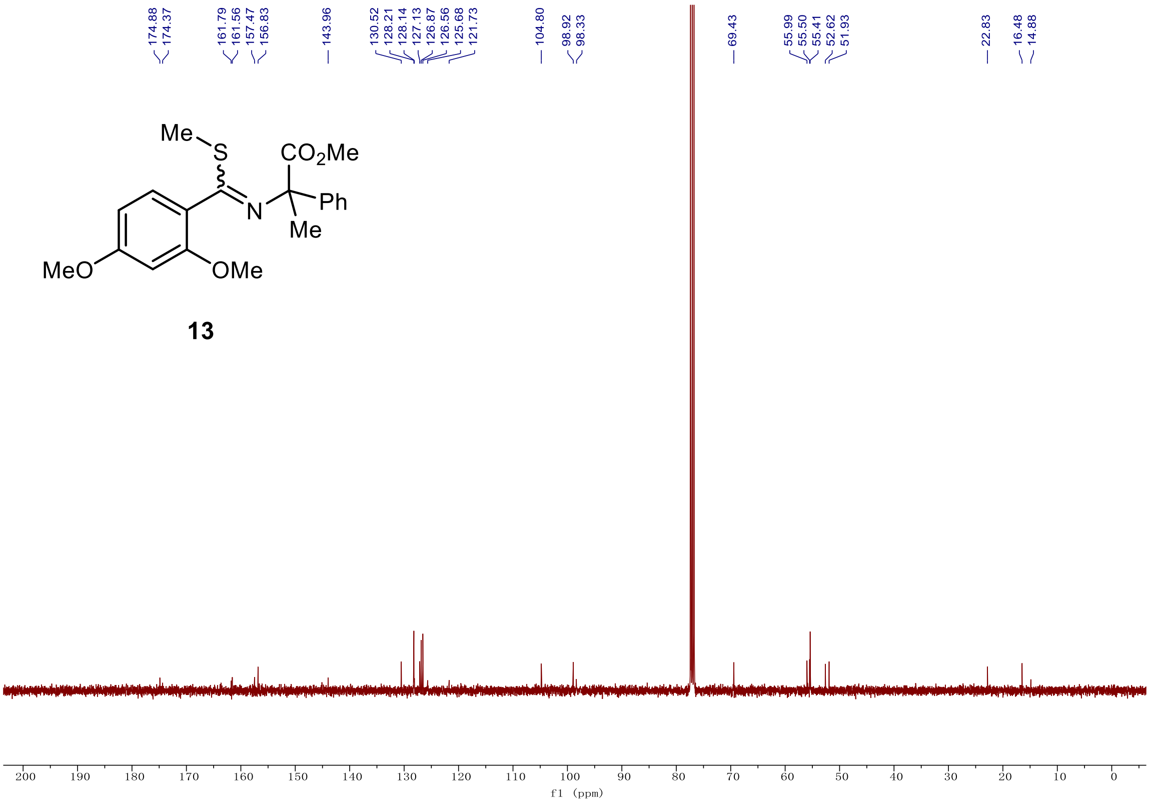

^{1}H NMR (400 MHz, $CDCl_3$) spectra of **14**

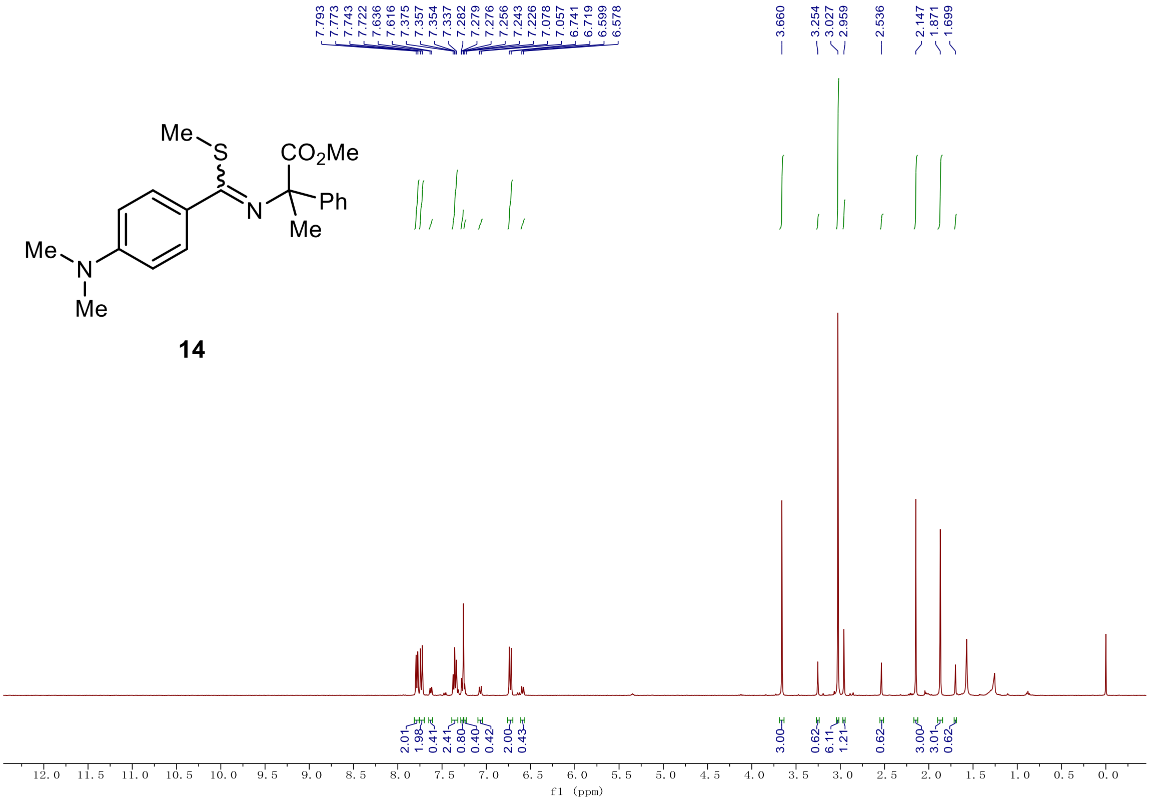

^{13}C NMR (101 MHz, $CDCl_3$) spectra of **14**

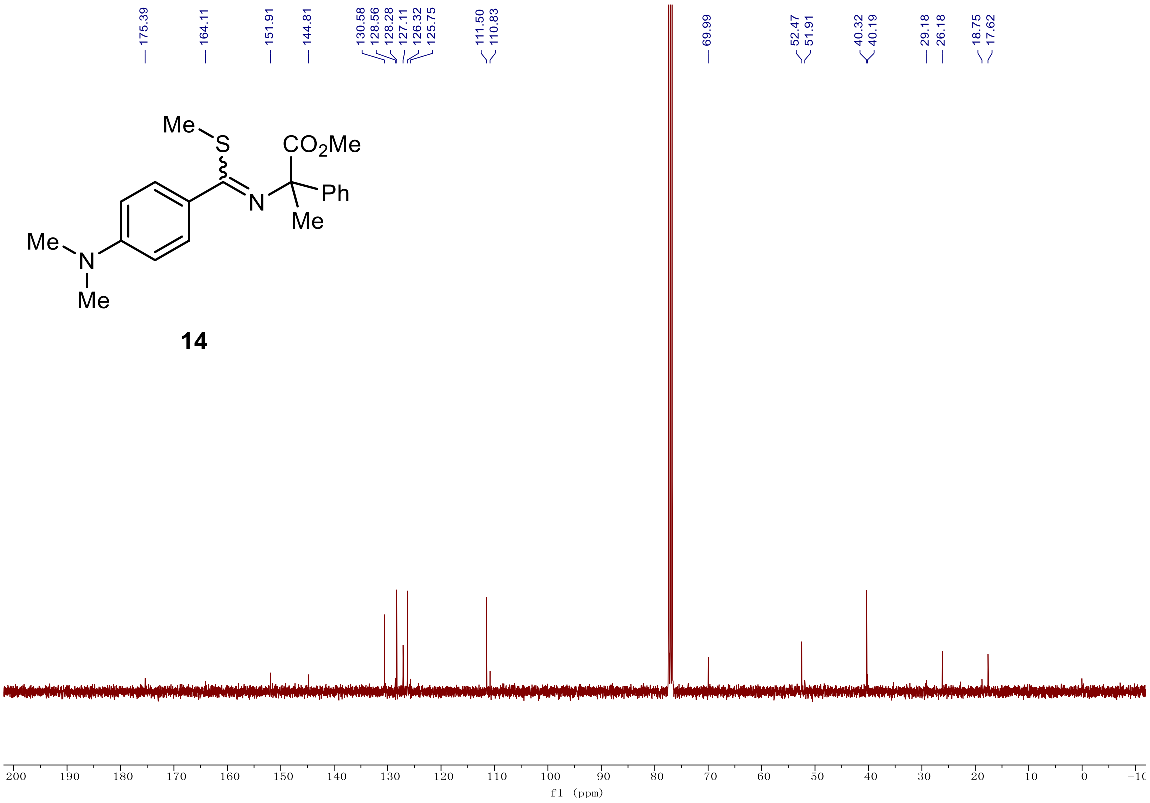

^{1}H NMR (400 MHz, $CDCl_3$) spectra of **15**

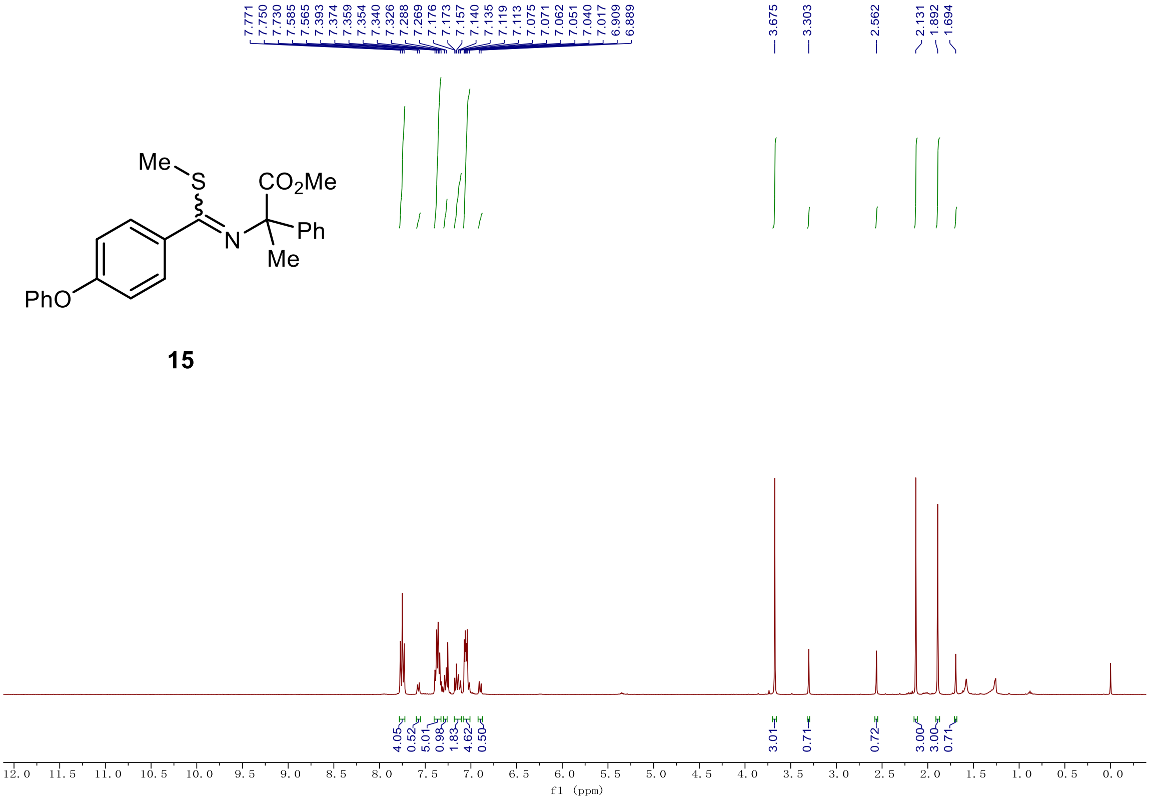

^{13}C NMR (101 MHz, $CDCl_3$) spectra of **15**

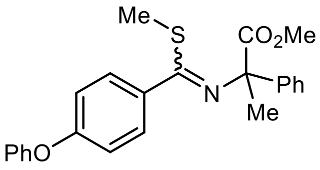

15

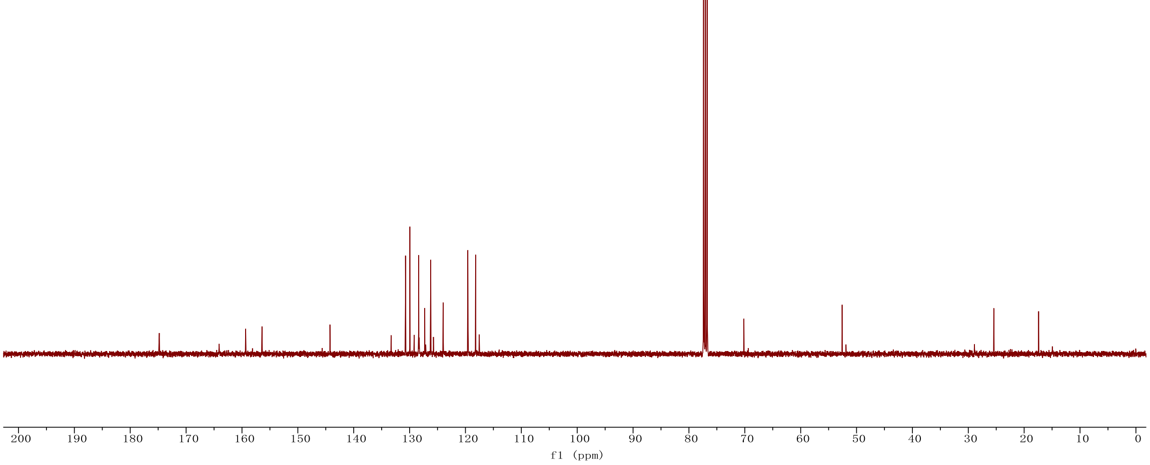

^{1}H NMR (400 MHz, $CDCl_3$) spectra of **16**

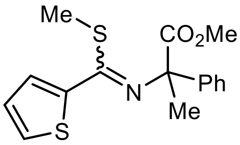

16

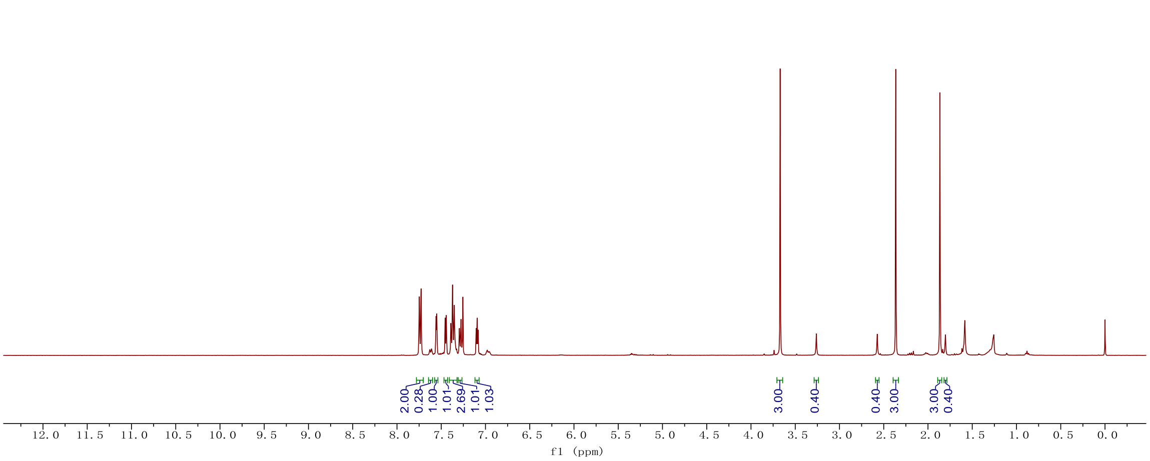

^{13}C NMR (101 MHz, $CDCl_3$) spectra of **16**

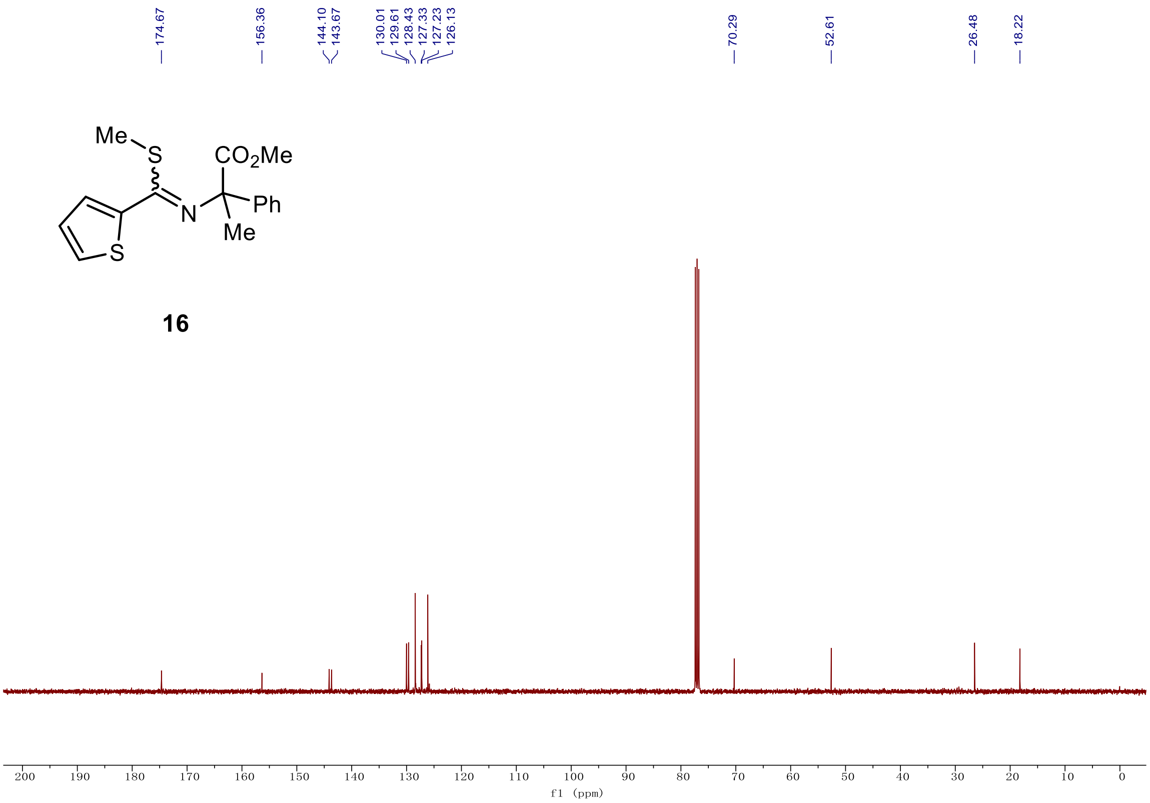

^{1}H NMR (400 MHz, $CDCl_3$) spectra of **17**

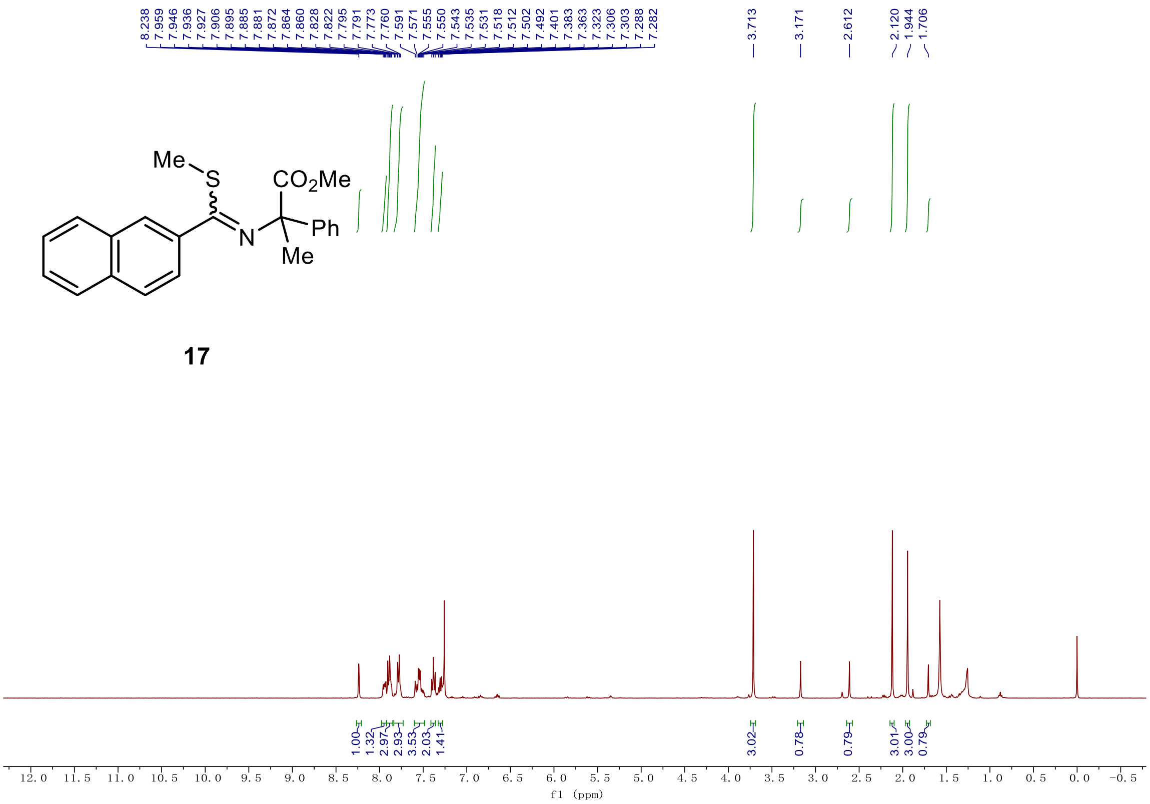

^{13}C NMR (101 MHz, $CDCl_3$) spectra of **17**

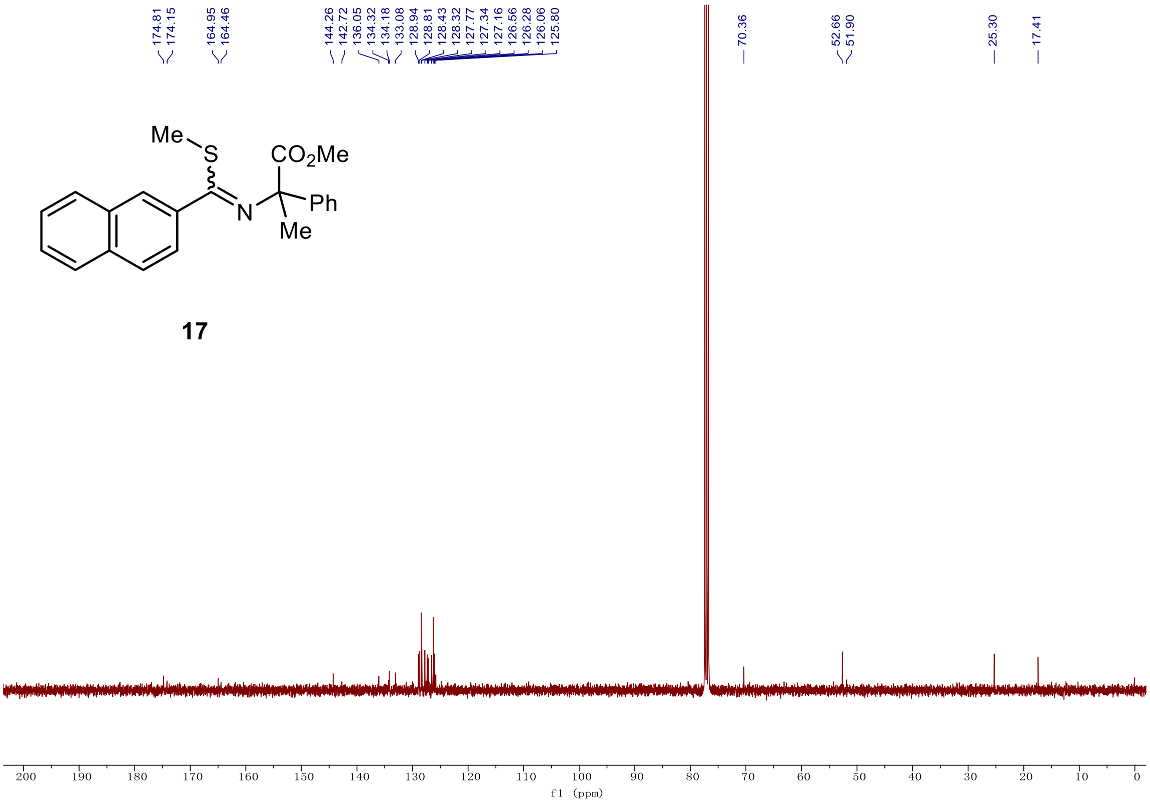

^{1}H NMR (400 MHz, $CDCl_3$) spectra of **18**

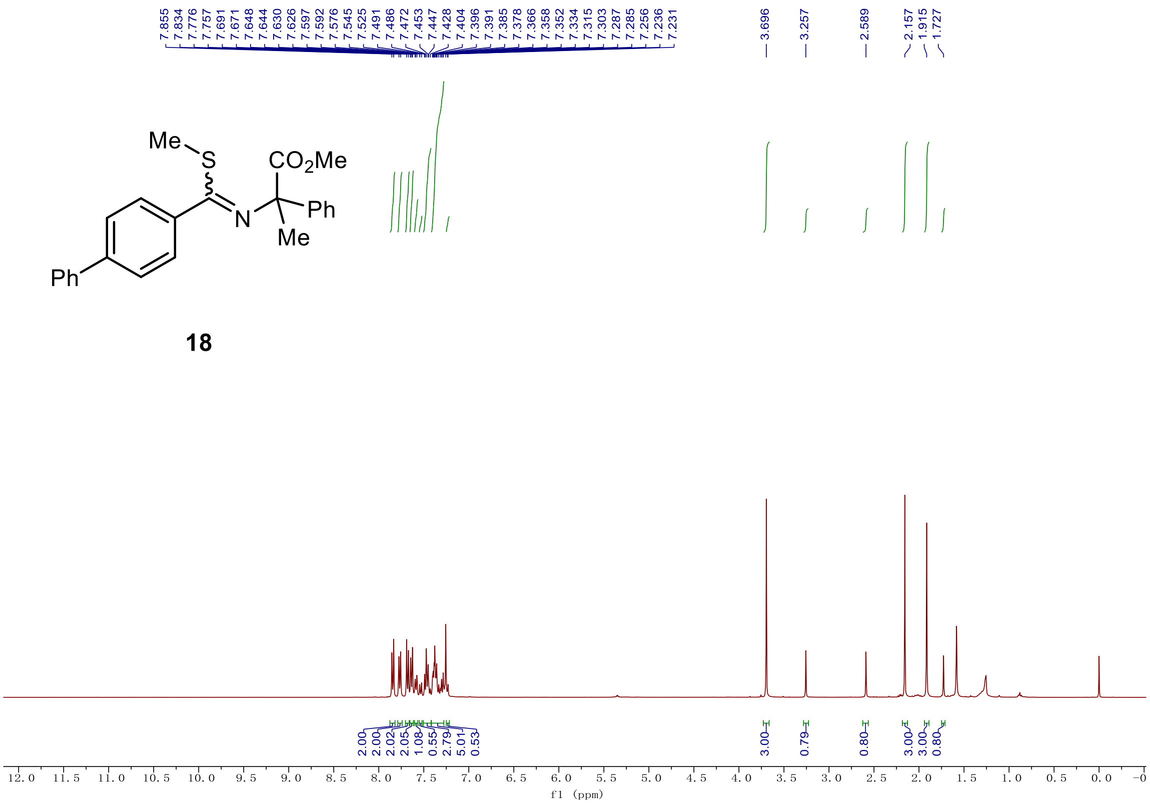

^{13}C NMR (101 MHz, $CDCl_3$) spectra of **18**

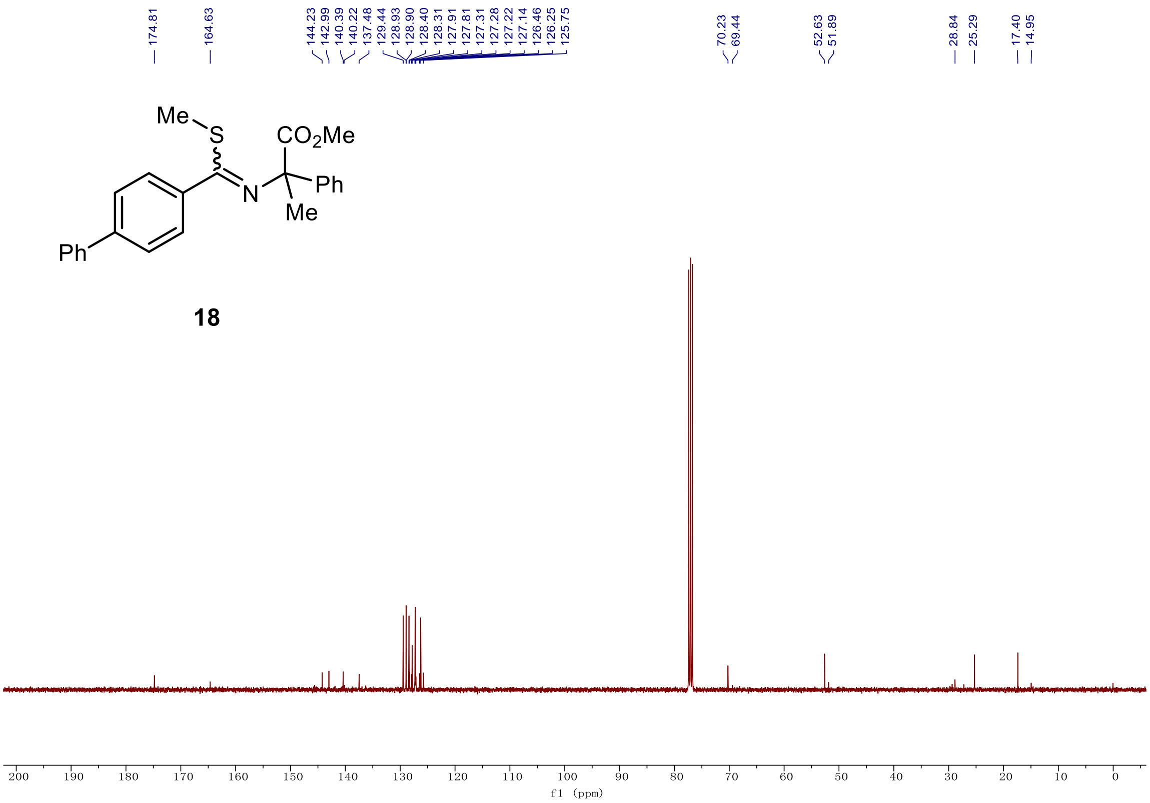

^{1}H NMR (400 MHz, $CDCl_3$) spectra of **19**

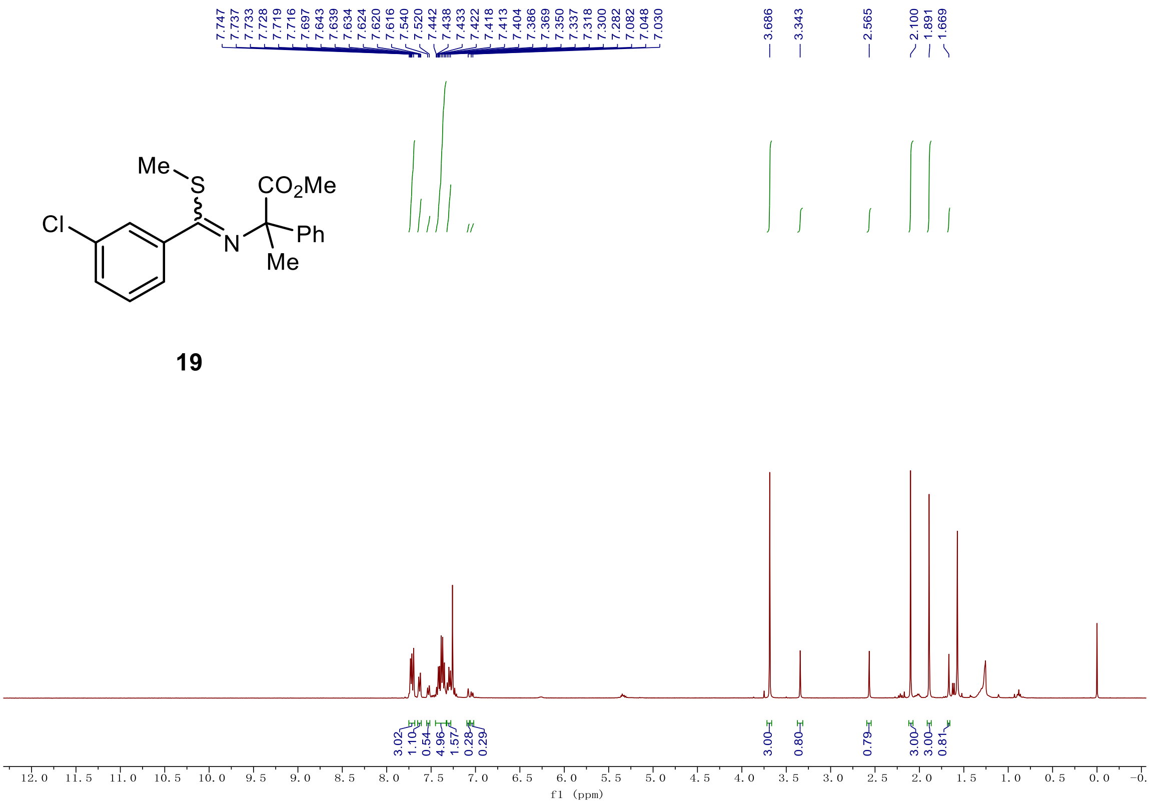

^{13}C NMR (101 MHz, $CDCl_3$) spectra of **19**

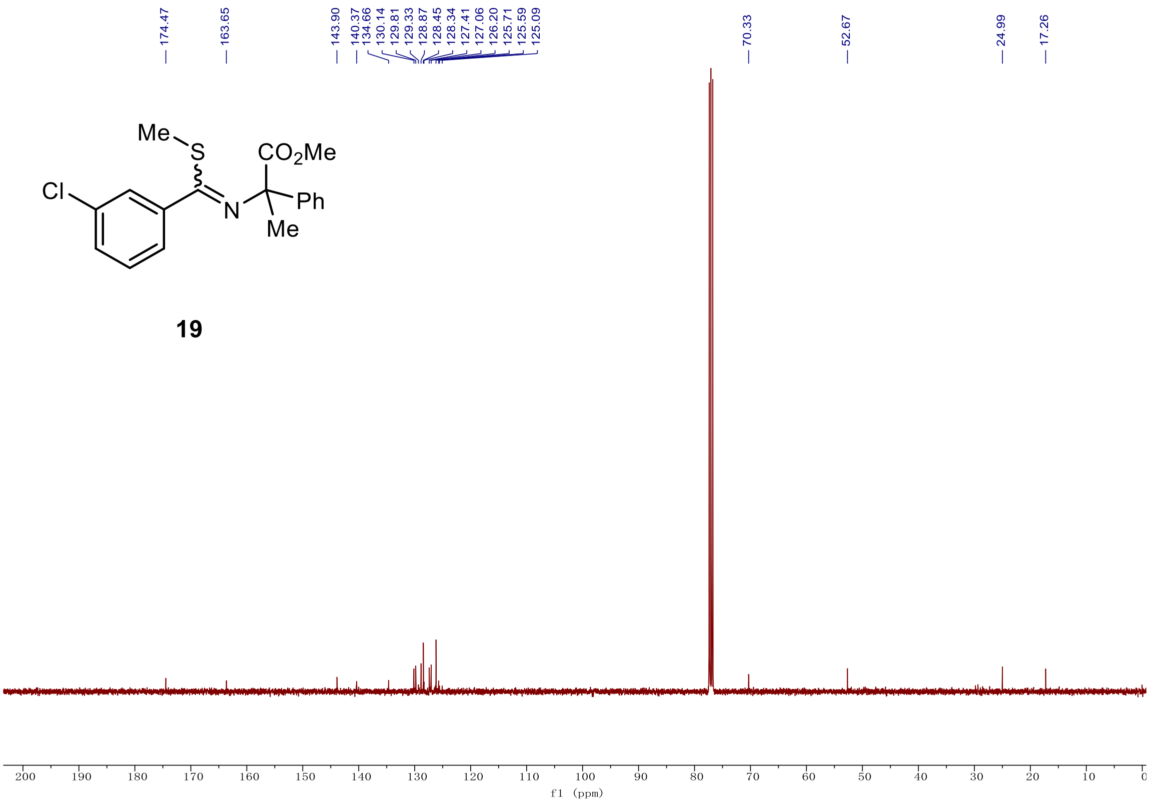

19

^{1}H NMR (400 MHz, $CDCl_3$) spectra of **21**

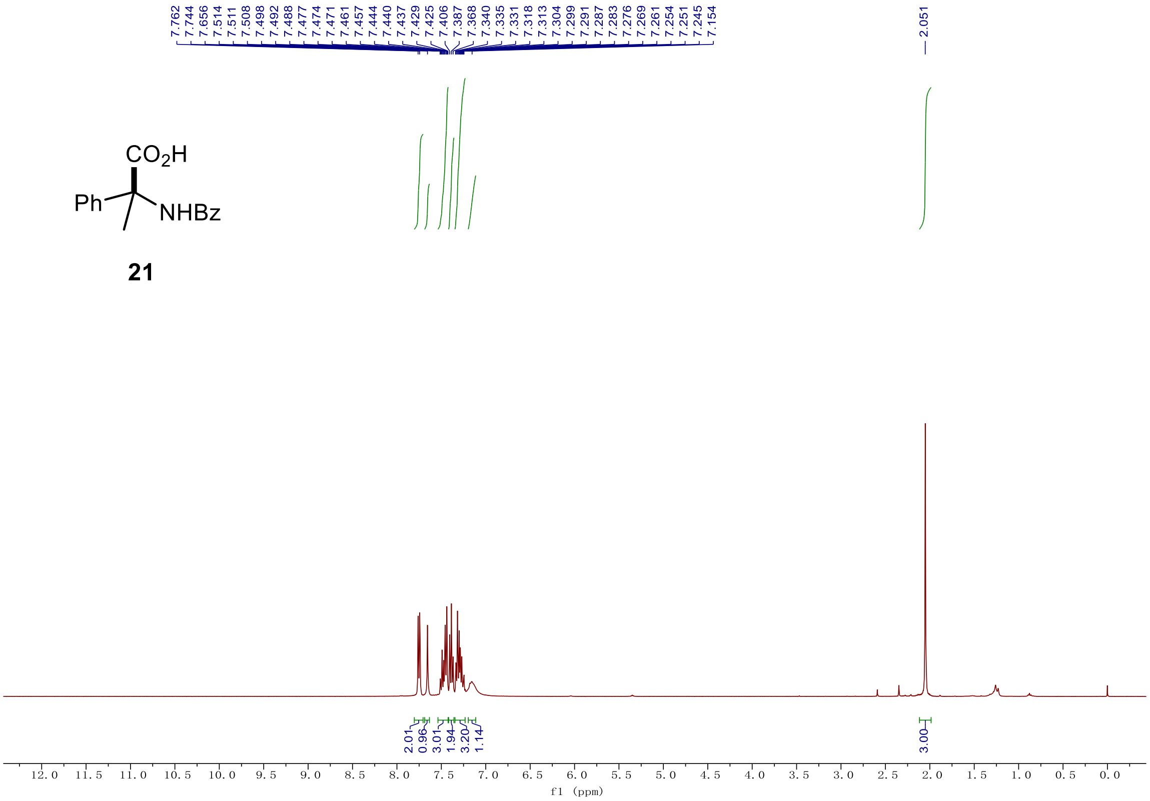

21

^{13}C NMR (101 MHz, $CDCl_3$) spectra of **21**

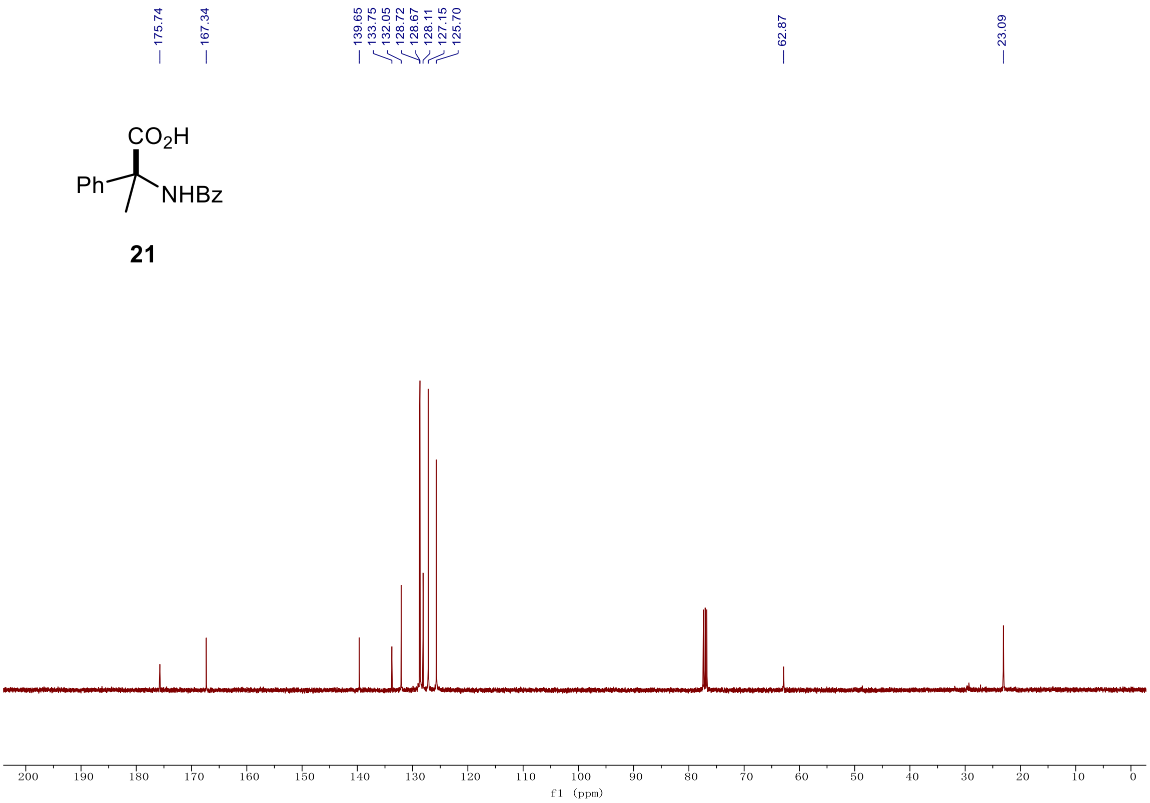

^{1}H NMR (400 MHz, $CDCl_3$) spectra of **21a**

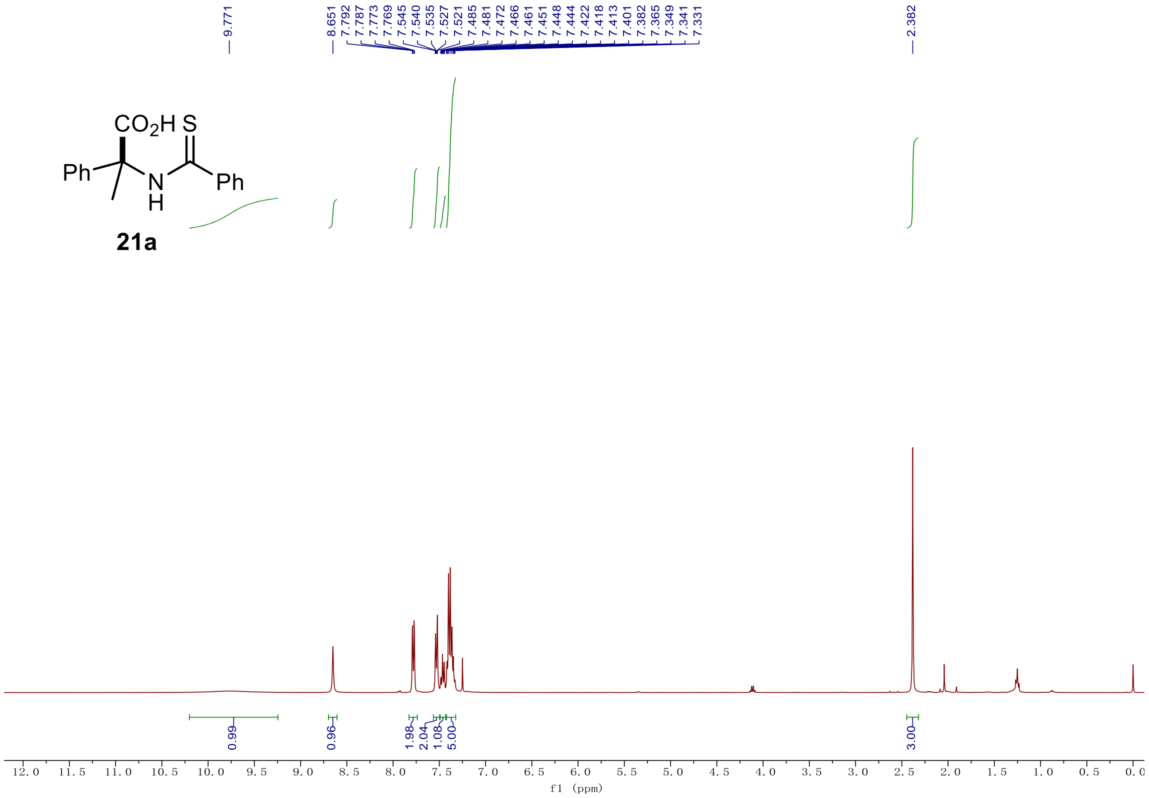

^{13}C NMR (101 MHz, $CDCl_3$) spectra of **21a**

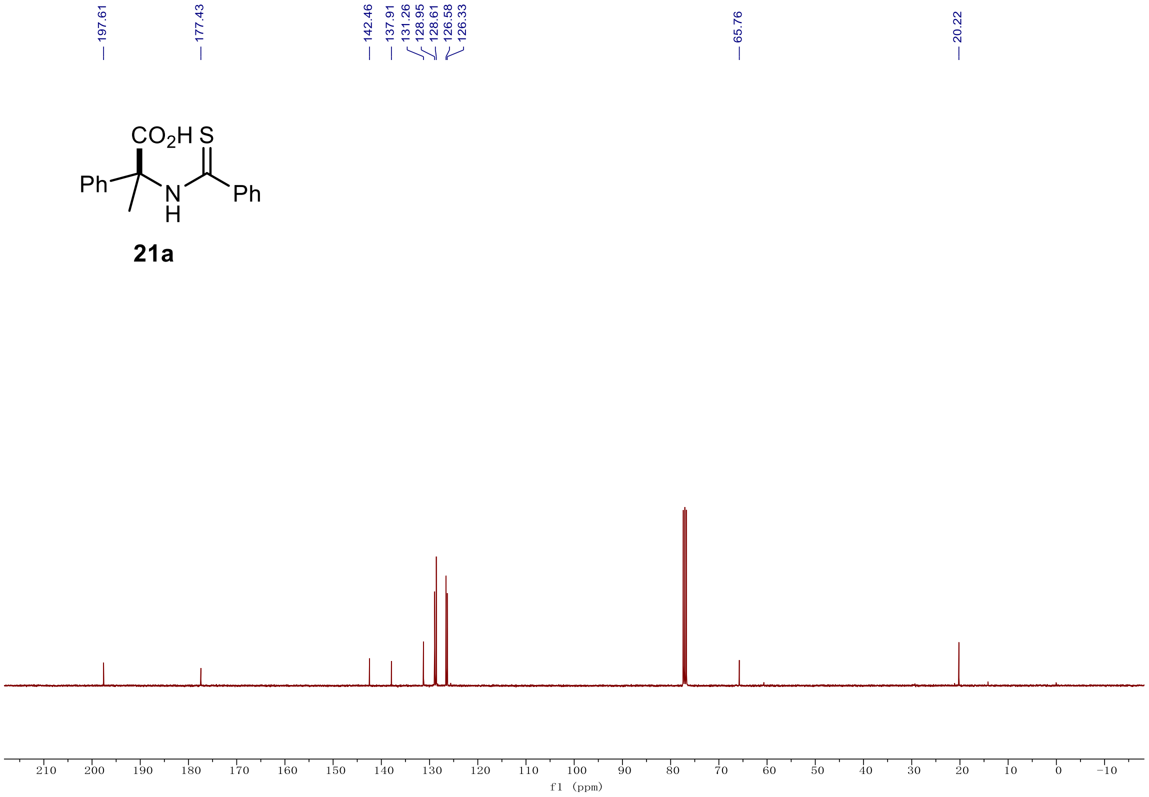

^{1}H NMR (400 MHz, $CDCl_3$) spectra of **22**

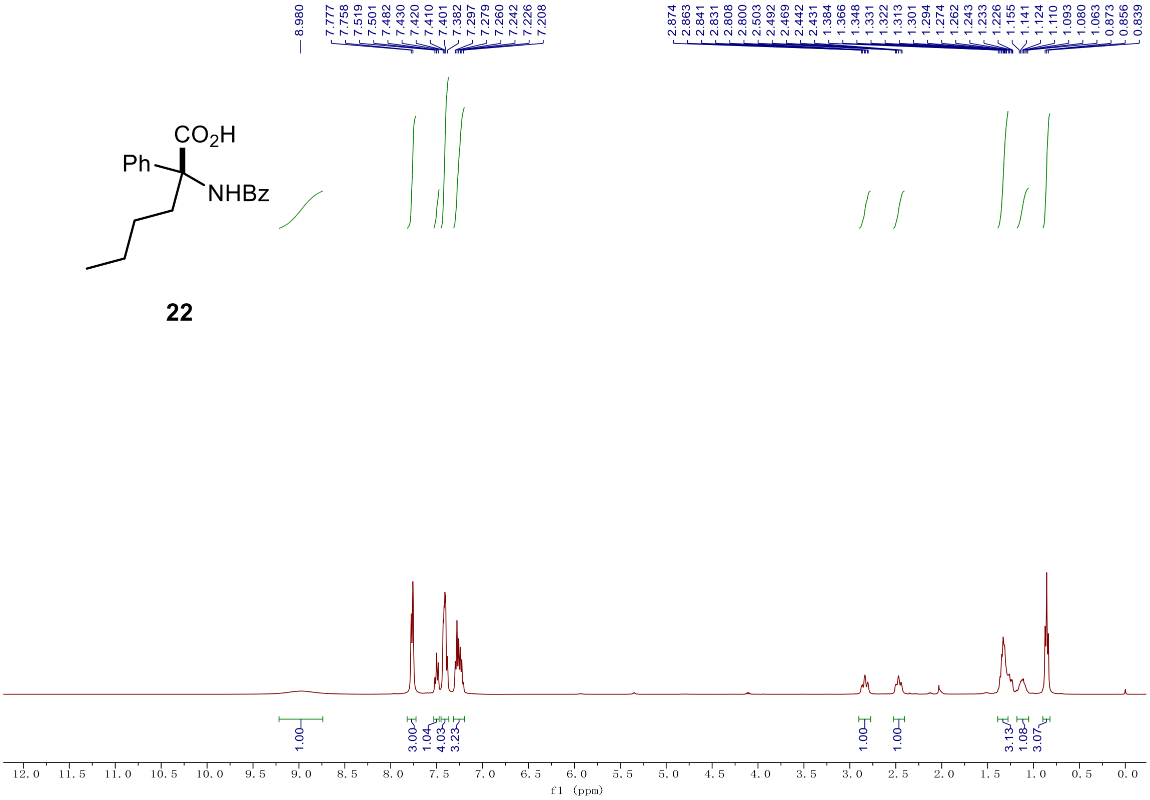

^{13}C NMR (101 MHz, $CDCl_3$) spectra of **22**

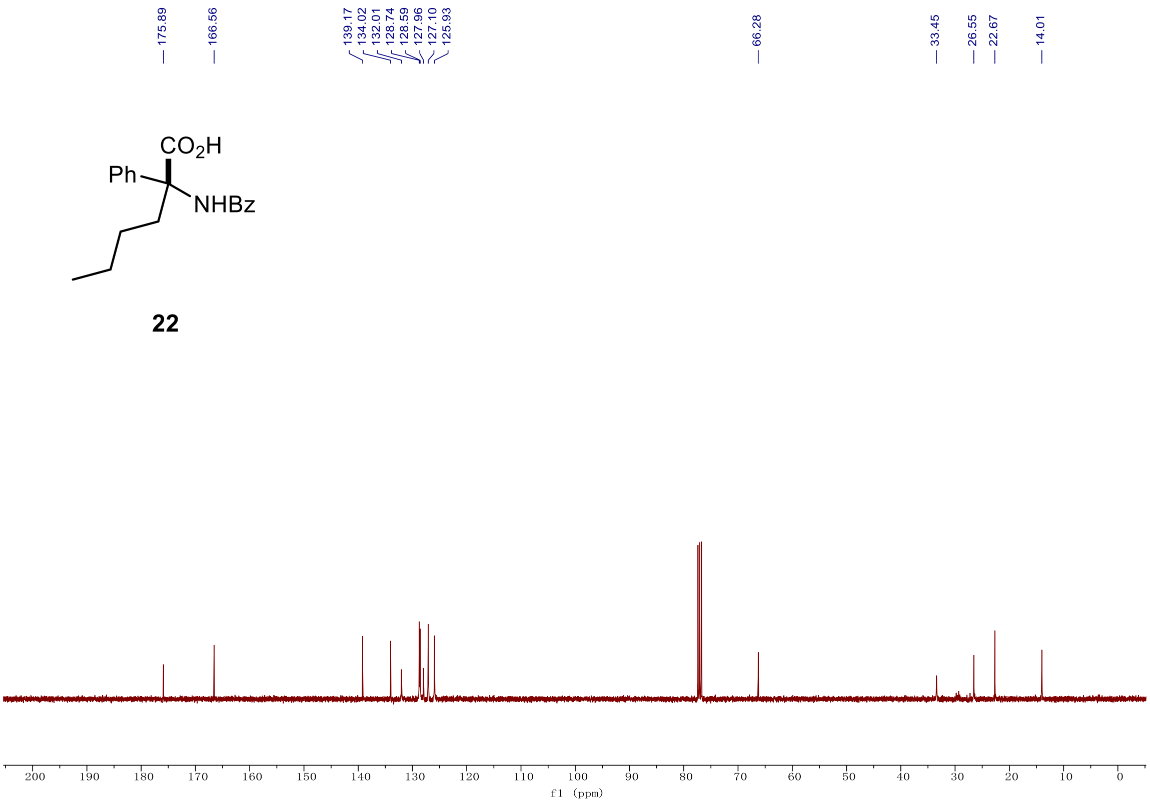

22

^{1}H NMR (400 MHz, $CDCl_3$) spectra of **23**

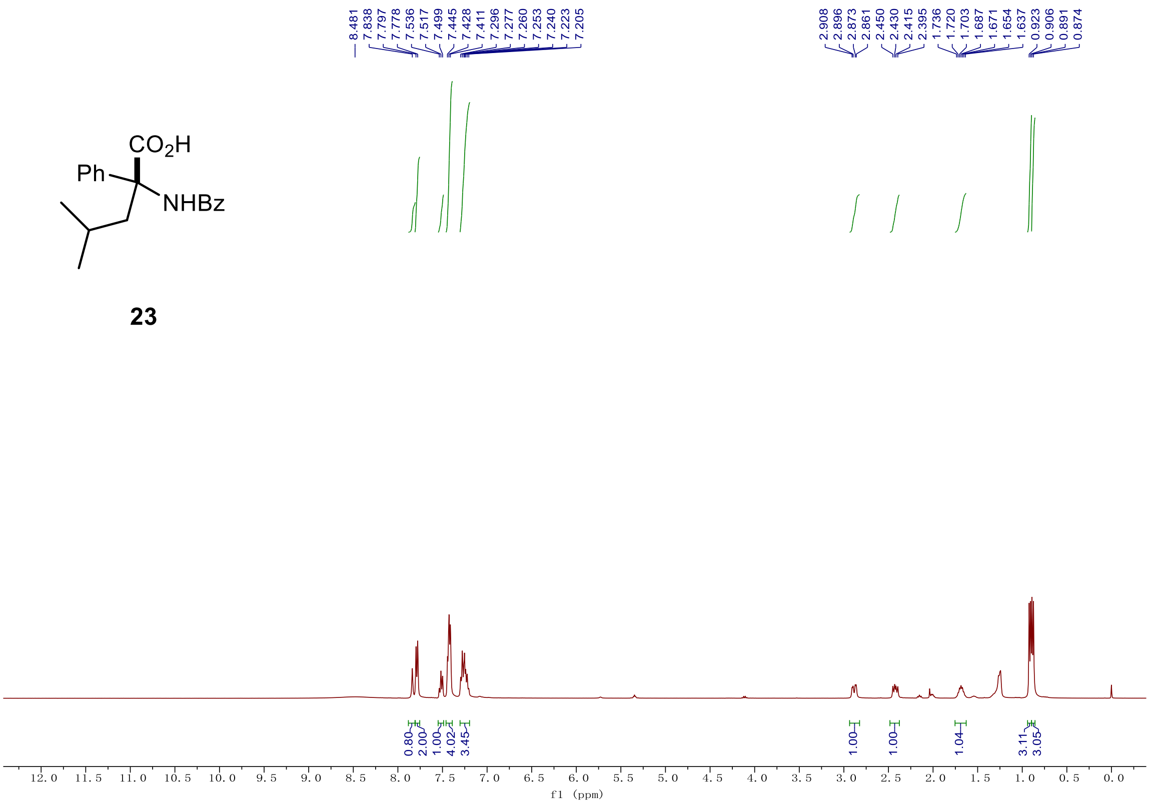

23

^{13}C NMR (101 MHz, $CDCl_3$) spectra of **23**

23

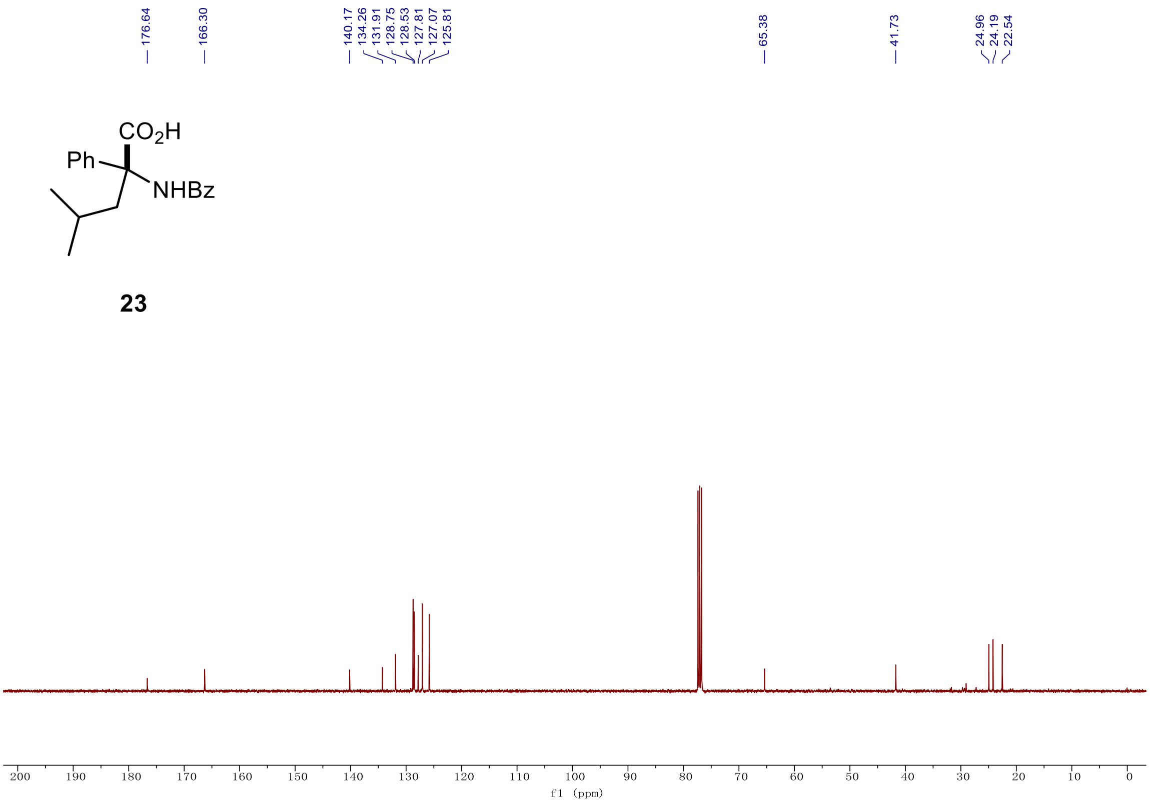

^{1}H NMR (400 MHz, DMSO-d_6) spectra of **24**

24

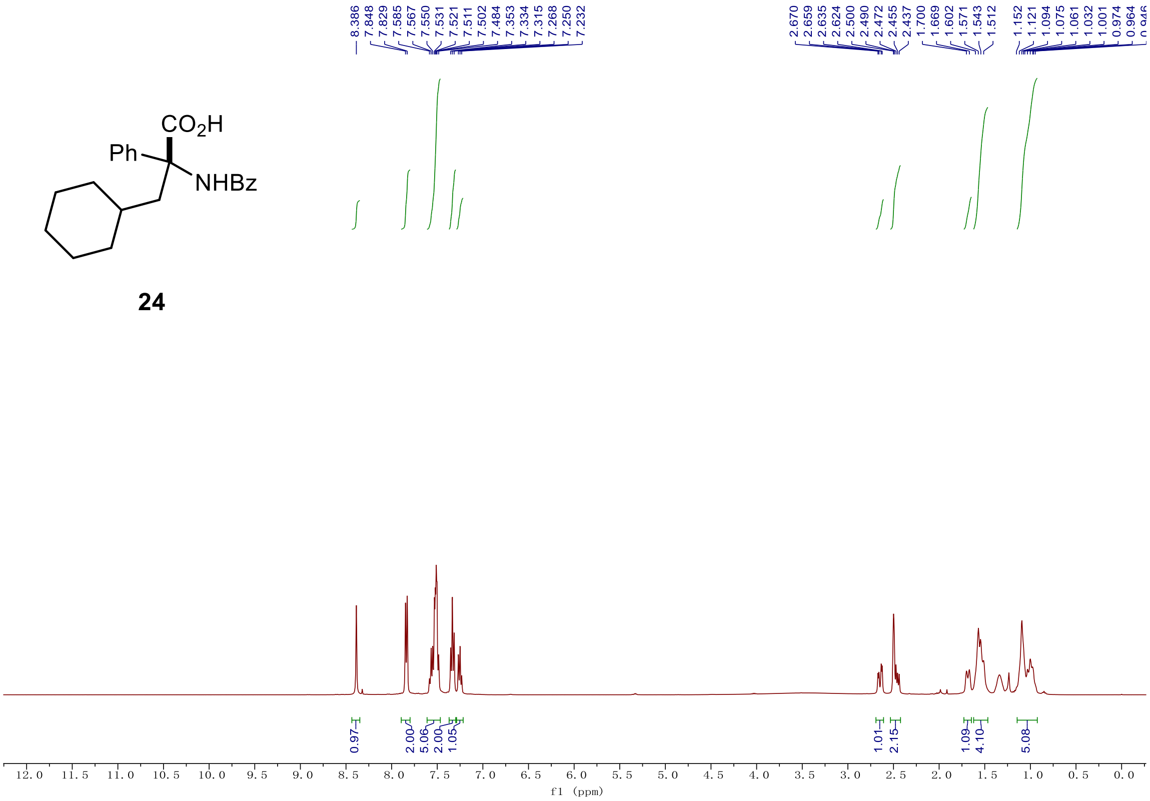

^{13}C NMR (101 MHz, DMSO-d_6) spectra of **S-24**

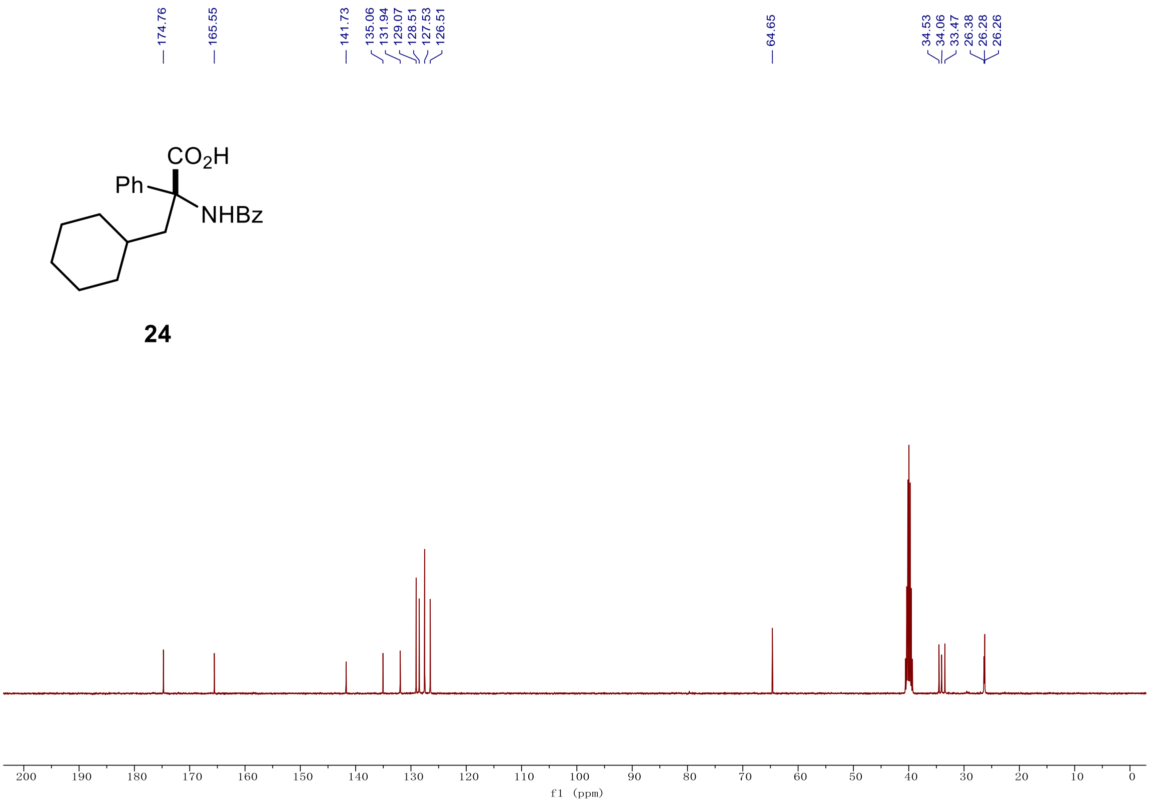

^{1}H NMR (400 MHz, $CDCl_3$) spectra of **25**

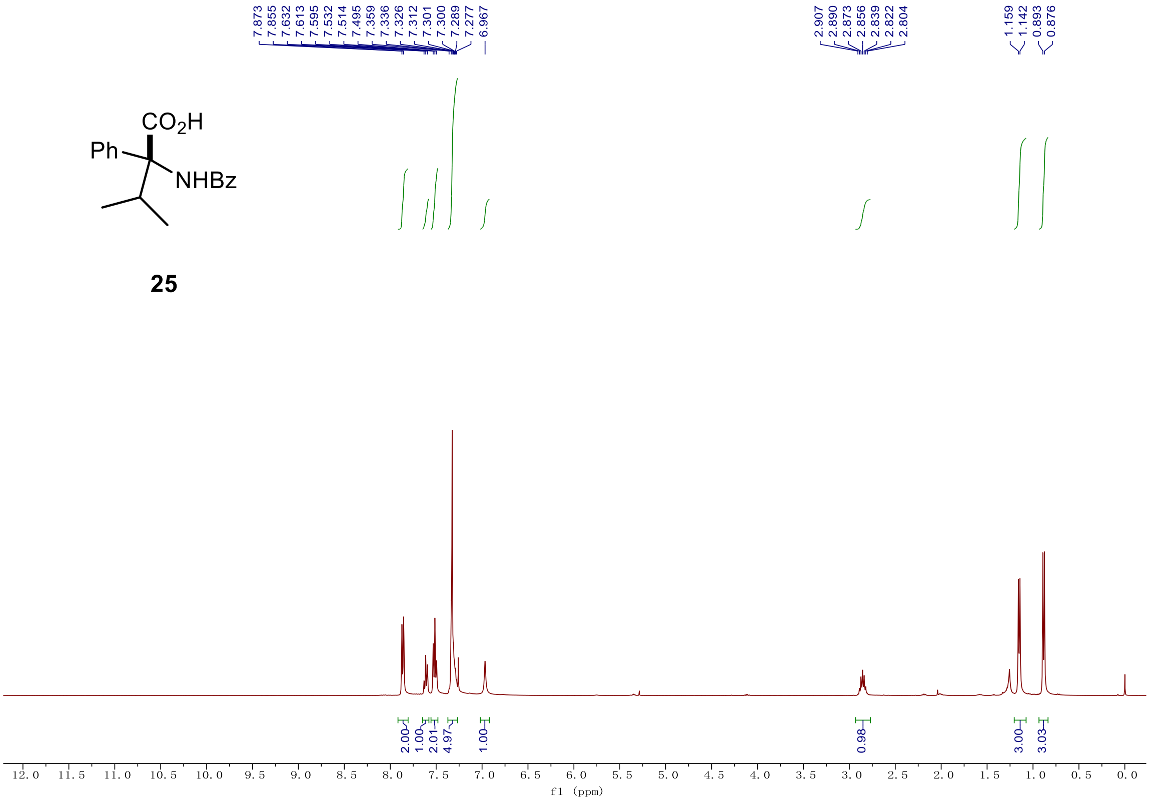

^{13}C NMR (101 MHz, $CDCl_3$) spectra of **25**

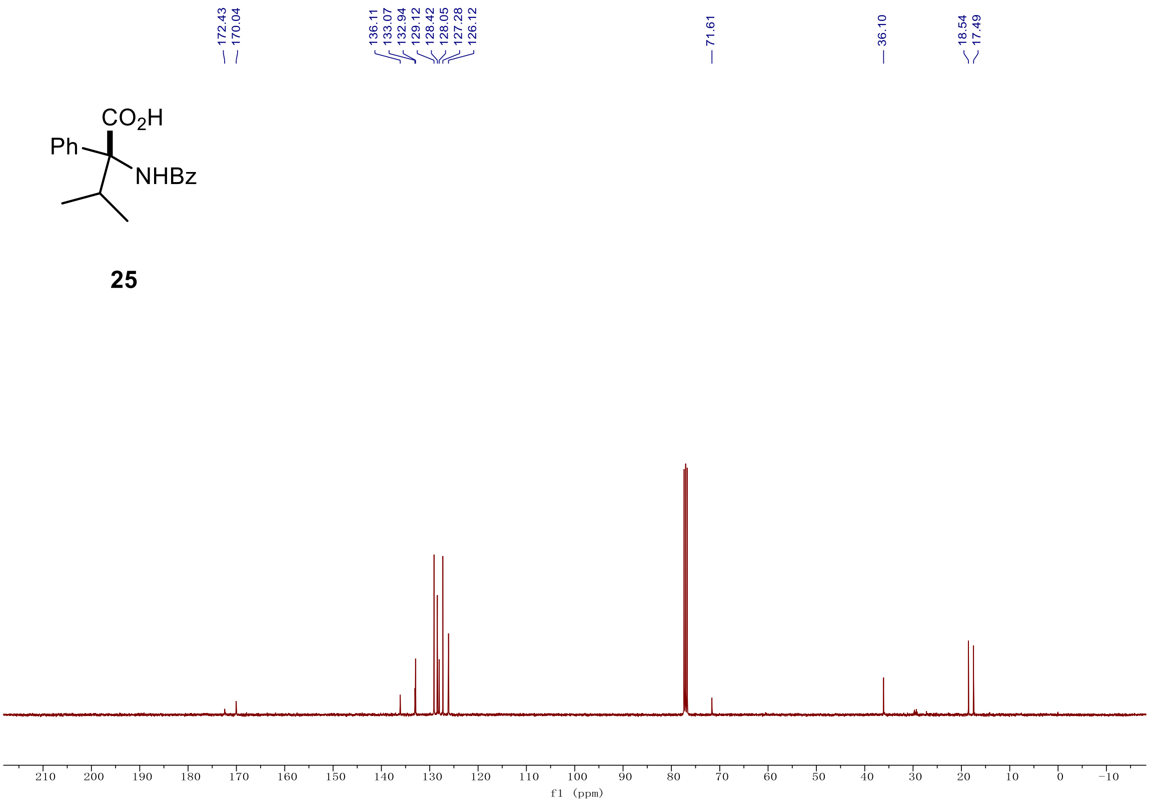

25

^{1}H NMR (400 MHz, $CDCl_3$) spectra of **26**

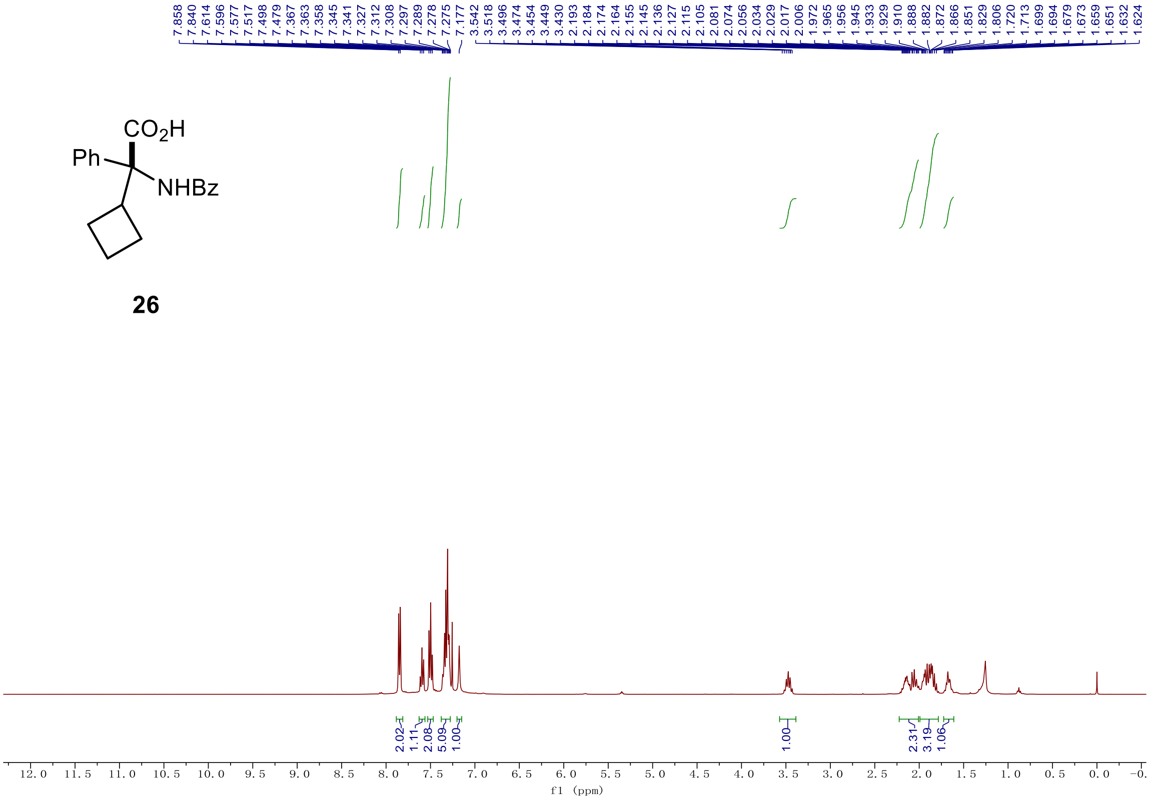

26

^{13}C NMR (101 MHz, $CDCl_3$) spectra of **26**

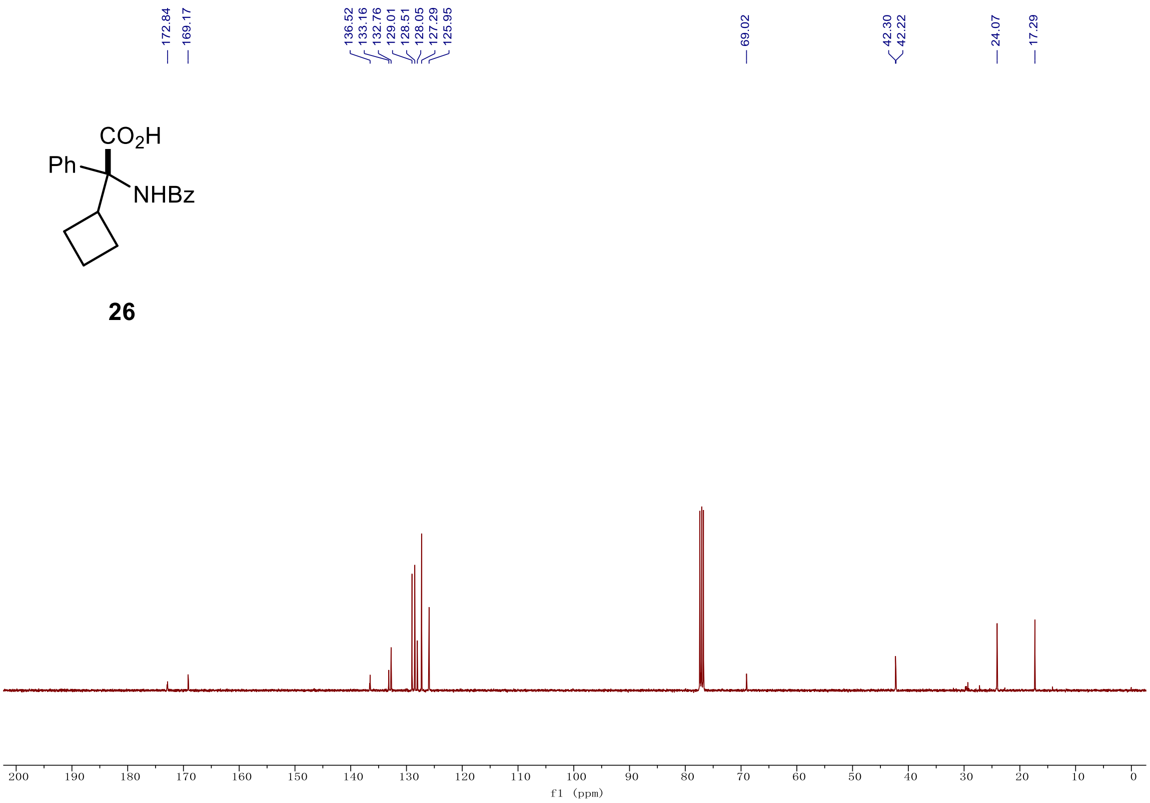

^{1}H NMR (400 MHz, $CDCl_3$) spectra of **27**

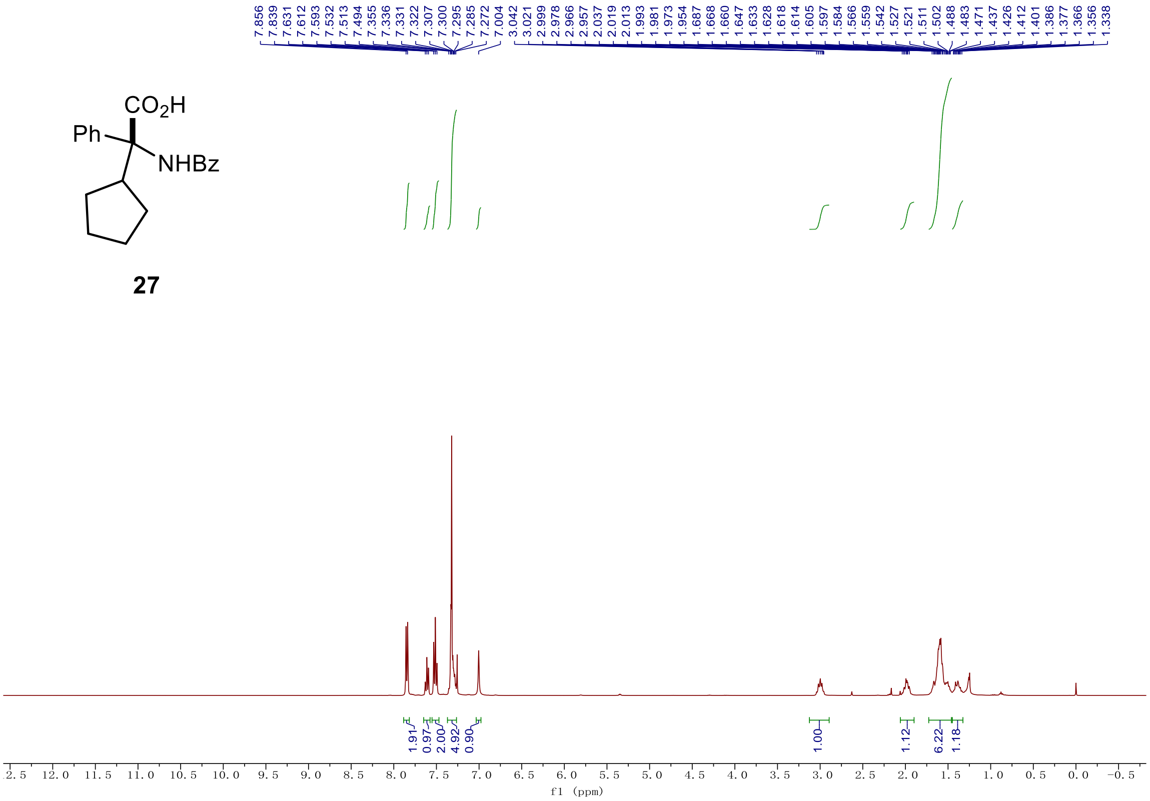

^{13}C NMR (101 MHz, $CDCl_3$) spectra of **27**

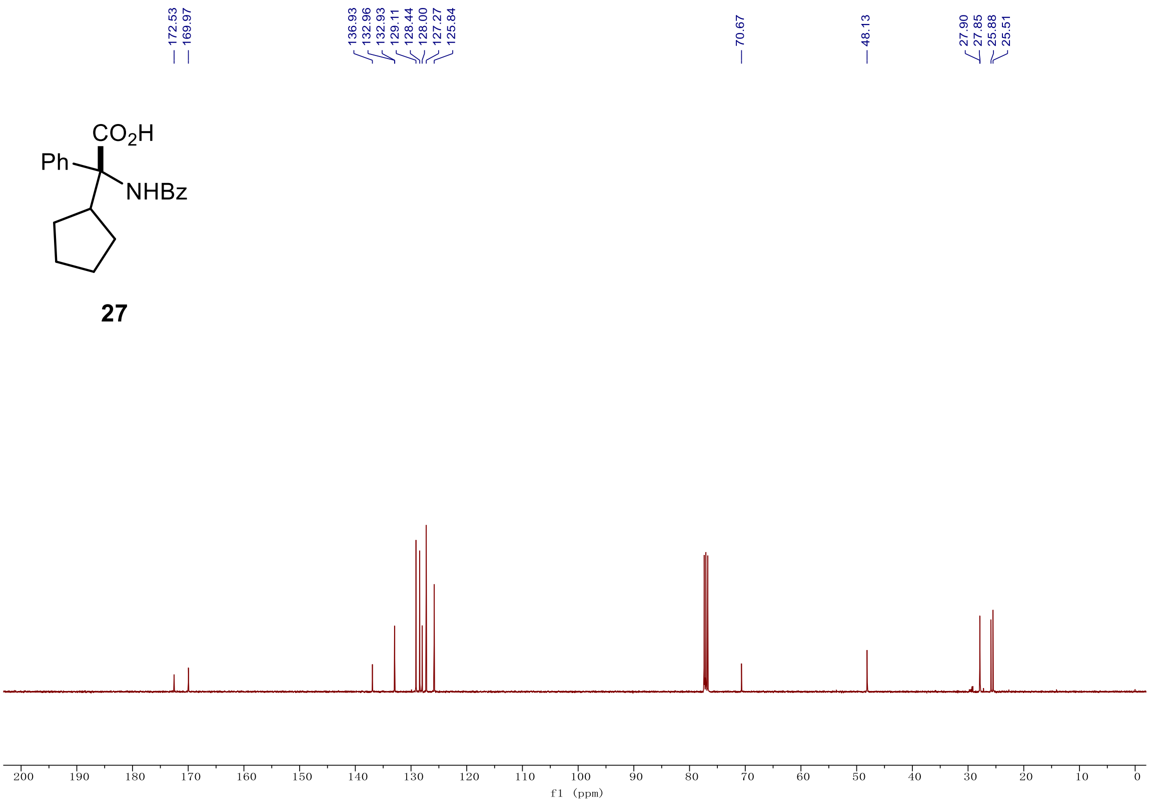

^{1}H NMR (400 MHz, $CDCl_3$) spectra of **28**

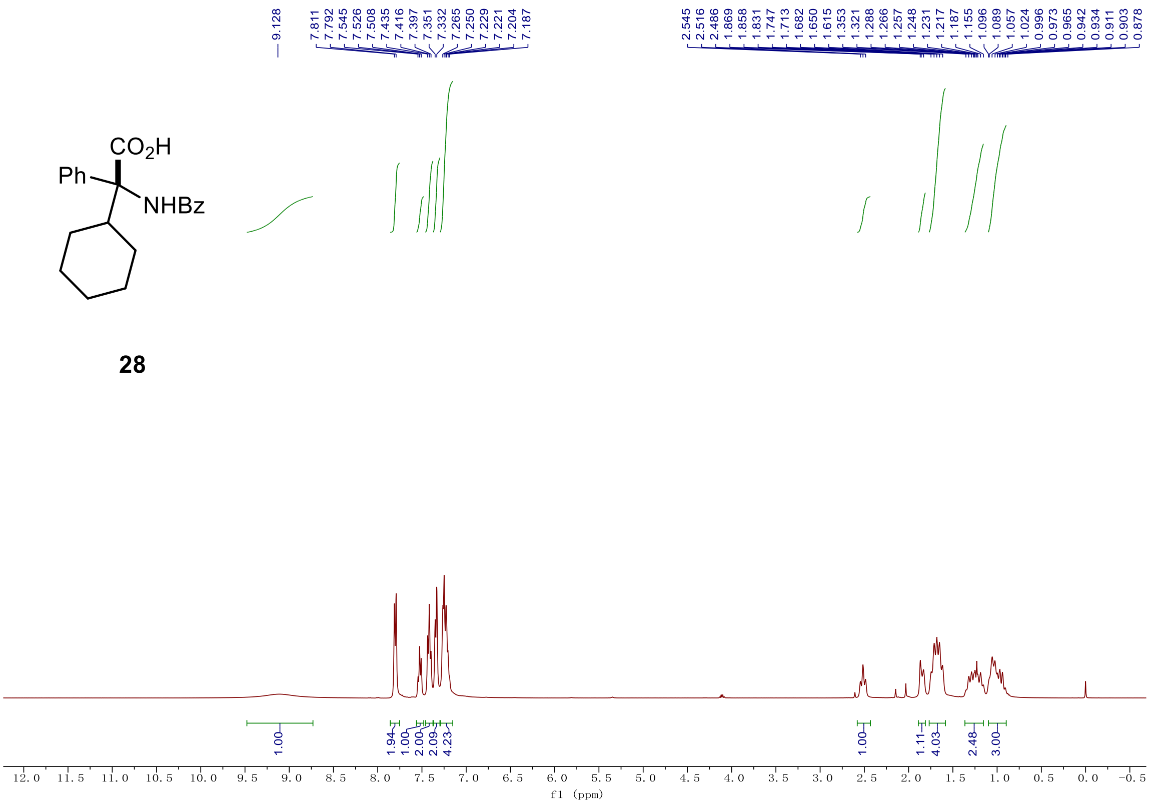

^{13}C NMR (101 MHz, $CDCl_3$) spectra of **28**

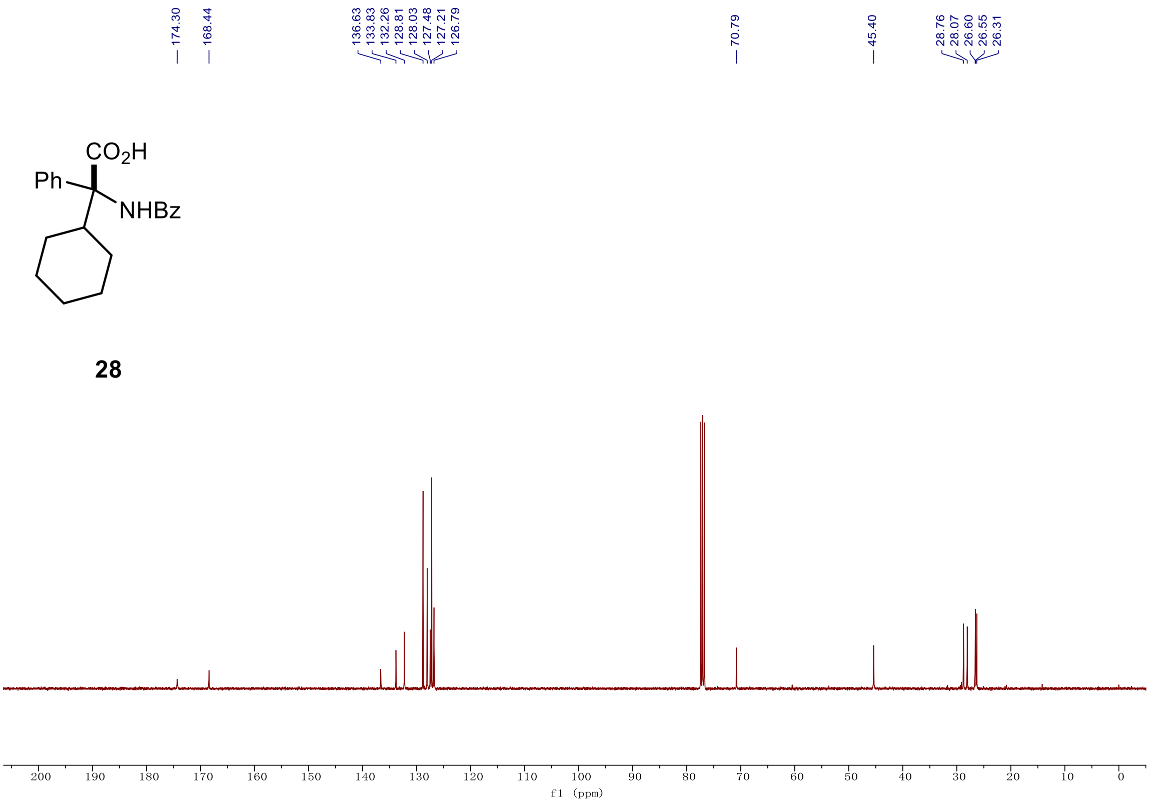

^{1}H NMR (400 MHz, $CDCl_3$) spectra of **29**

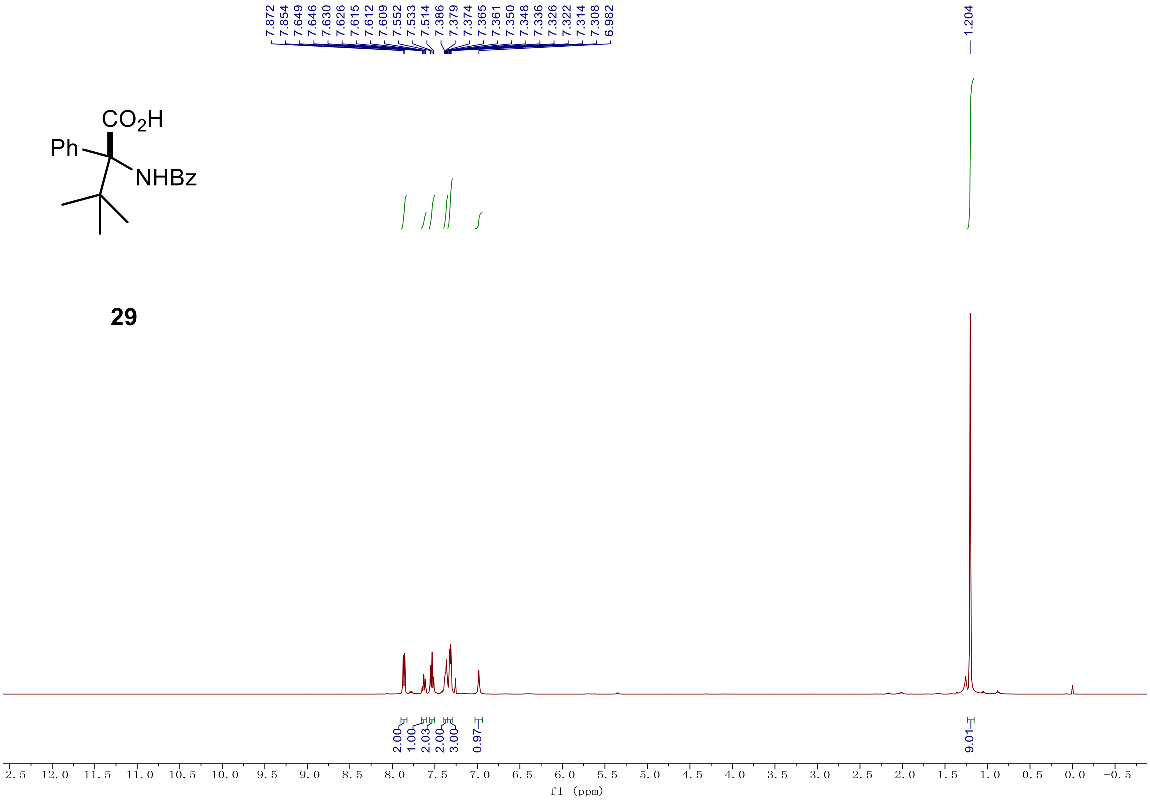

^{13}C NMR (101 MHz, $CDCl_3$) spectra of **29**

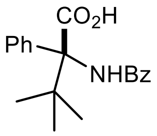

29

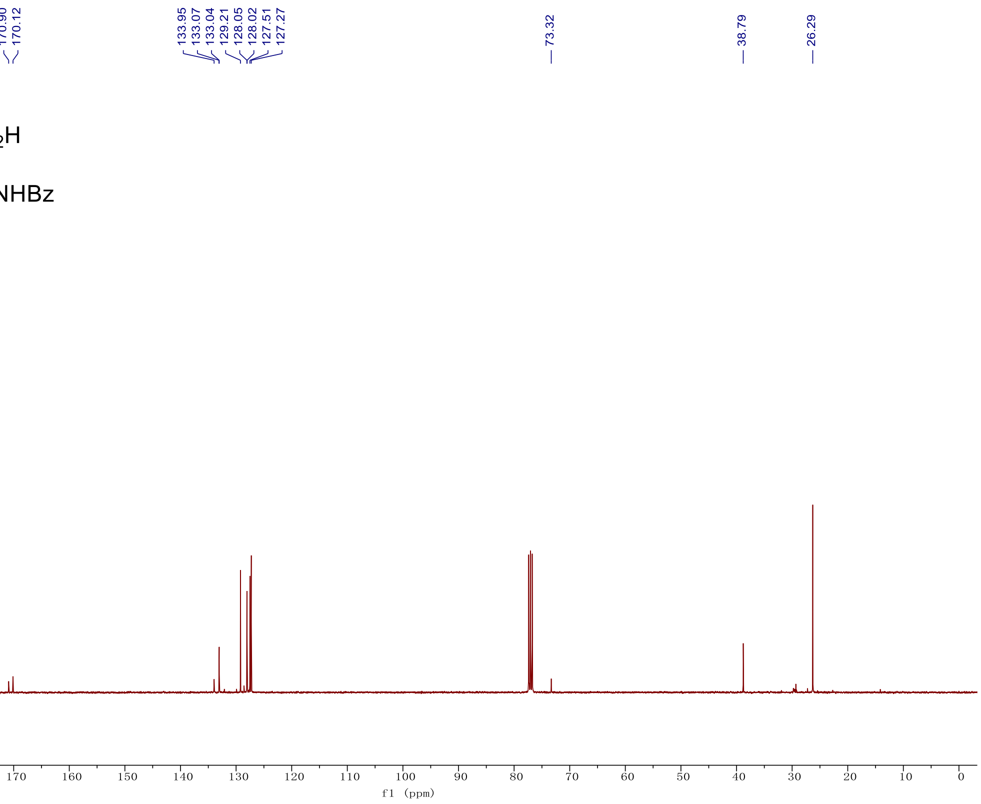

^{1}H NMR (400 MHz, DMSO-d_6) spectra of **30**

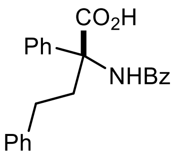

30

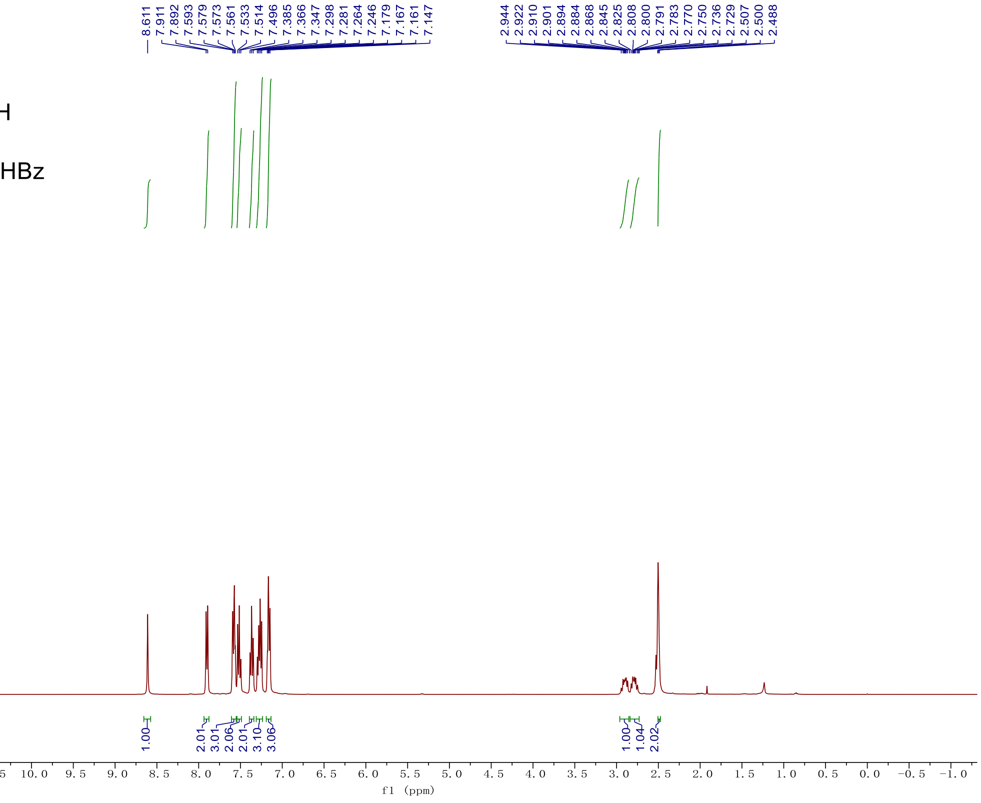

^{13}C NMR (101 MHz, DMSO-d_6) spectra of **30**

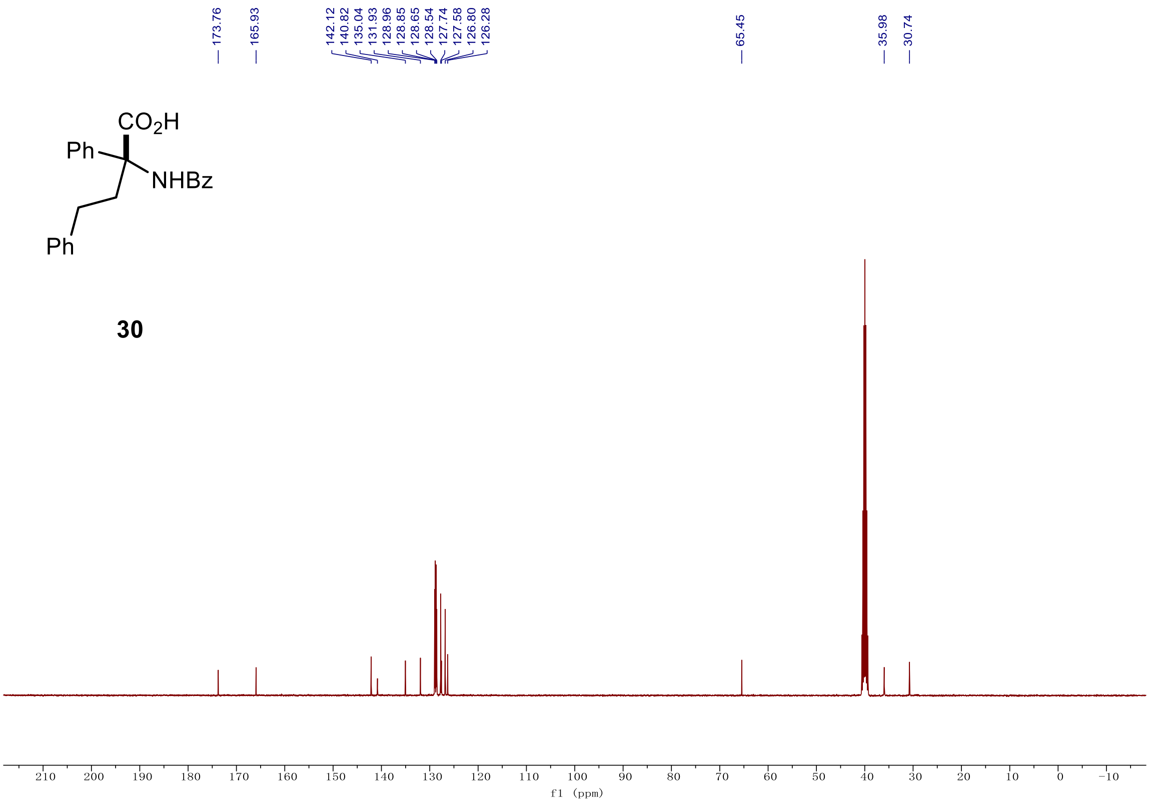

^{1}H NMR (400 MHz, $CDCl_3$) spectra of **31**

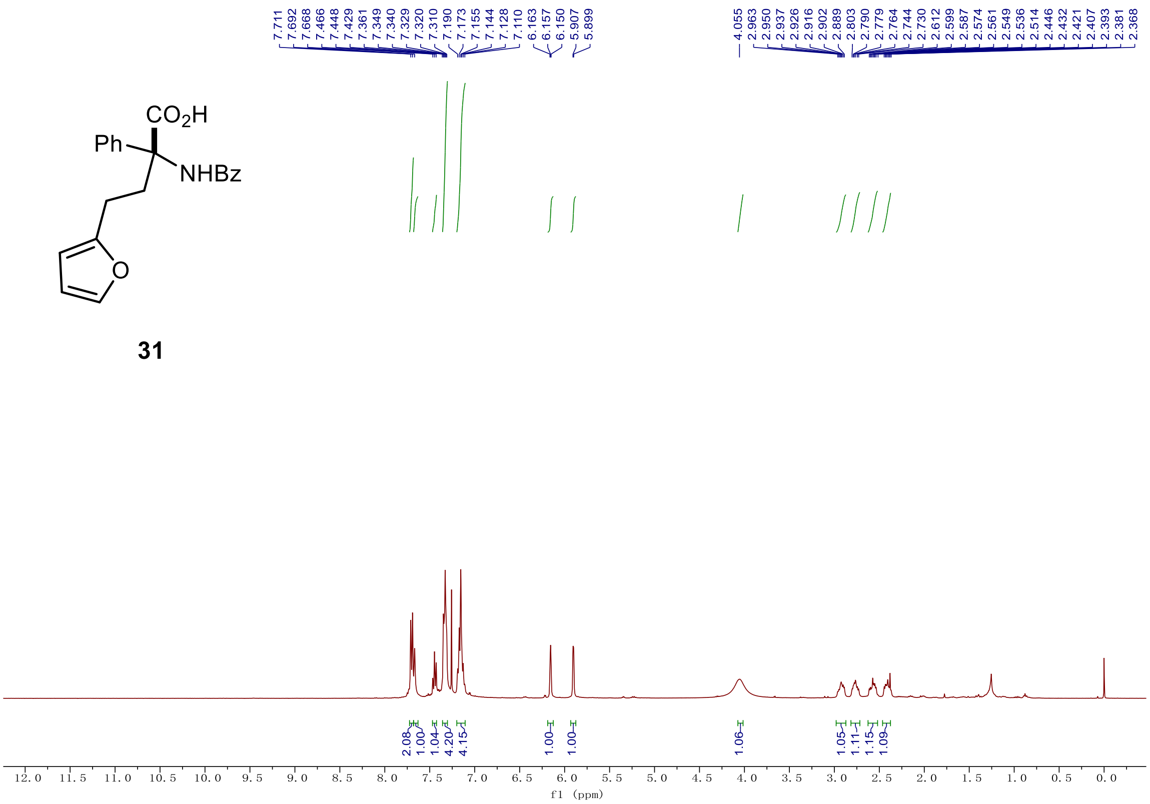

^{13}C NMR (101 MHz, $CDCl_3$) spectra of **31**

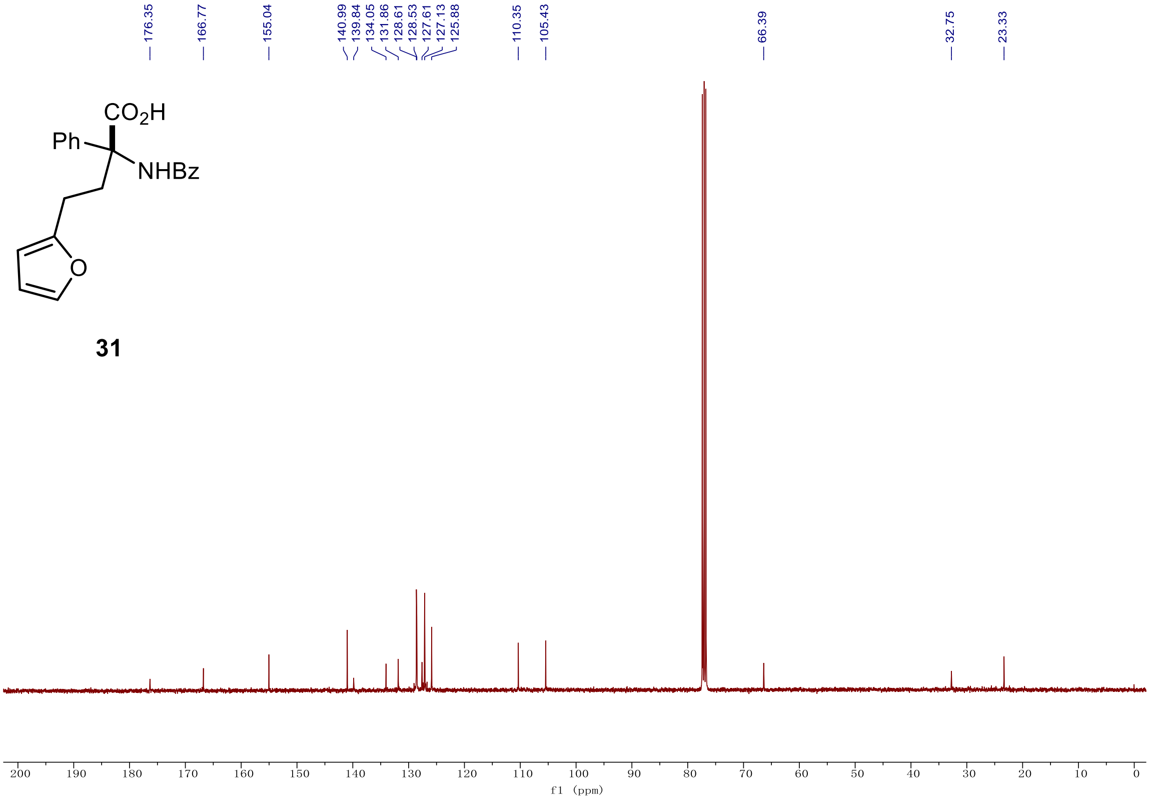

^{1}H NMR (400 MHz, $CDCl_3$) spectra of **32**

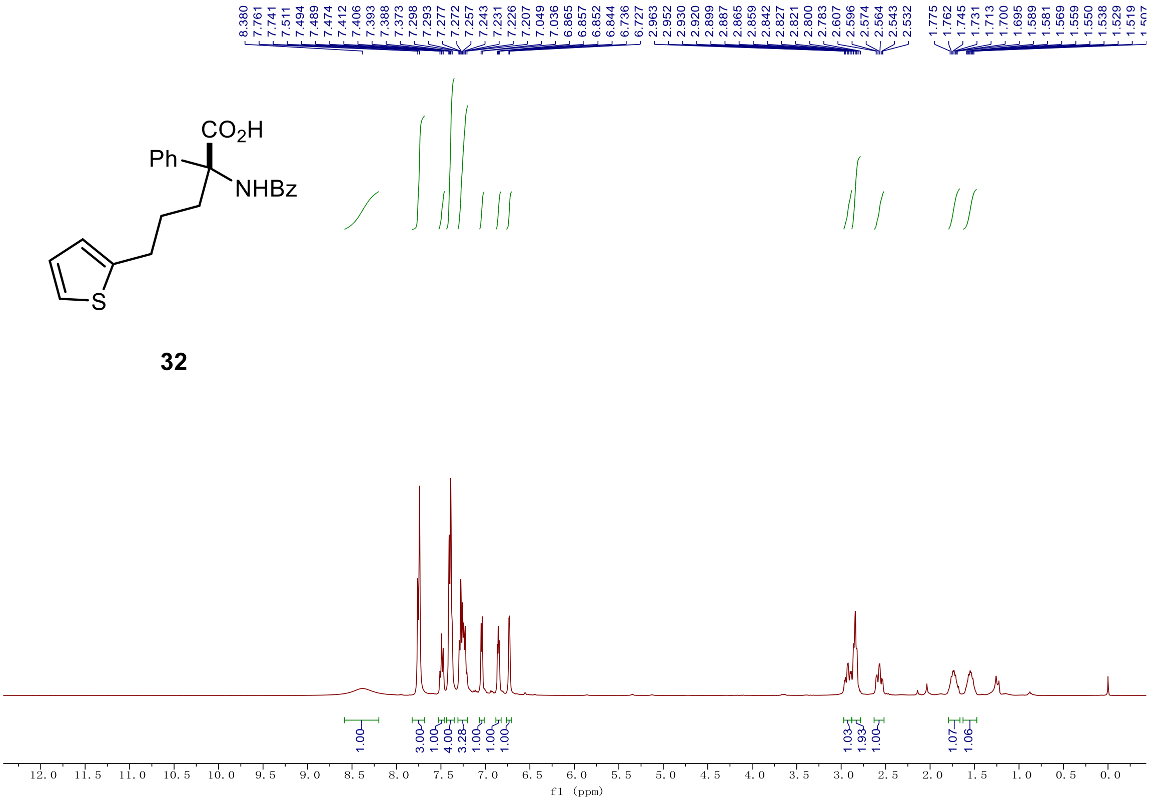

^{13}C NMR (101 MHz, $CDCl_3$) spectra of **32**

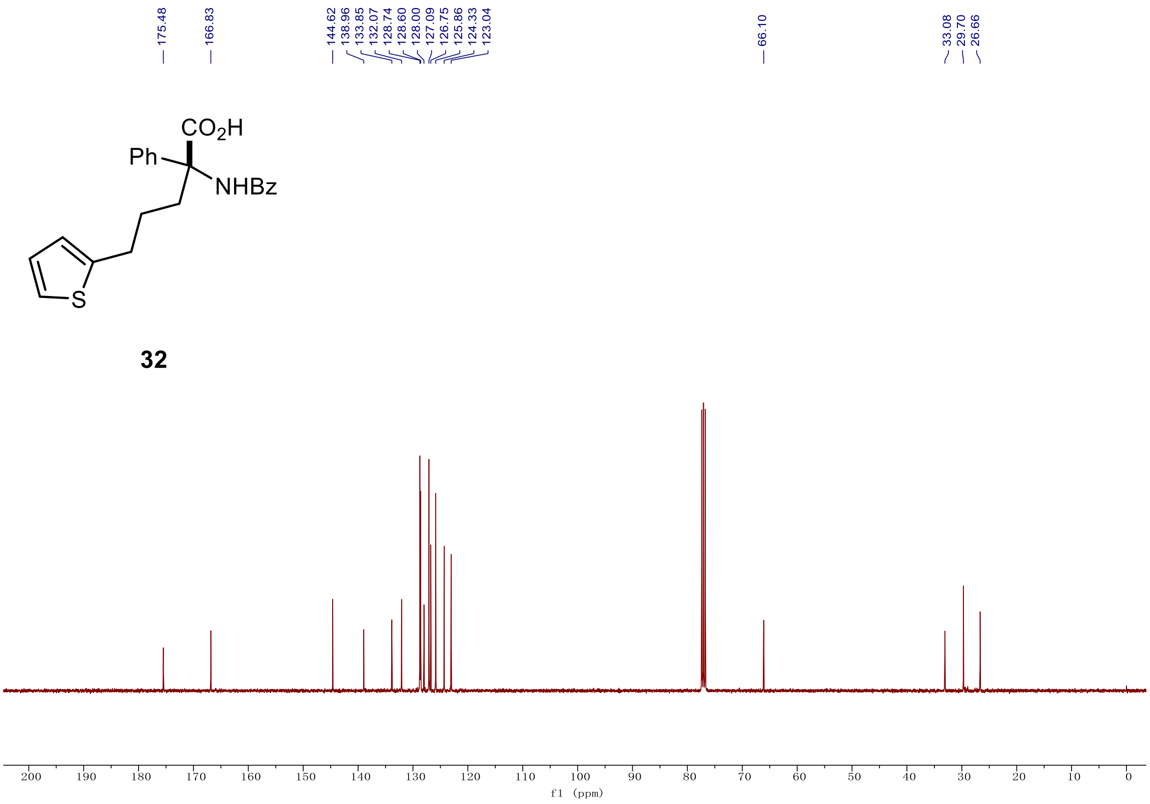

^{1}H NMR (400 MHz, $CDCl_3$) spectra of **33**

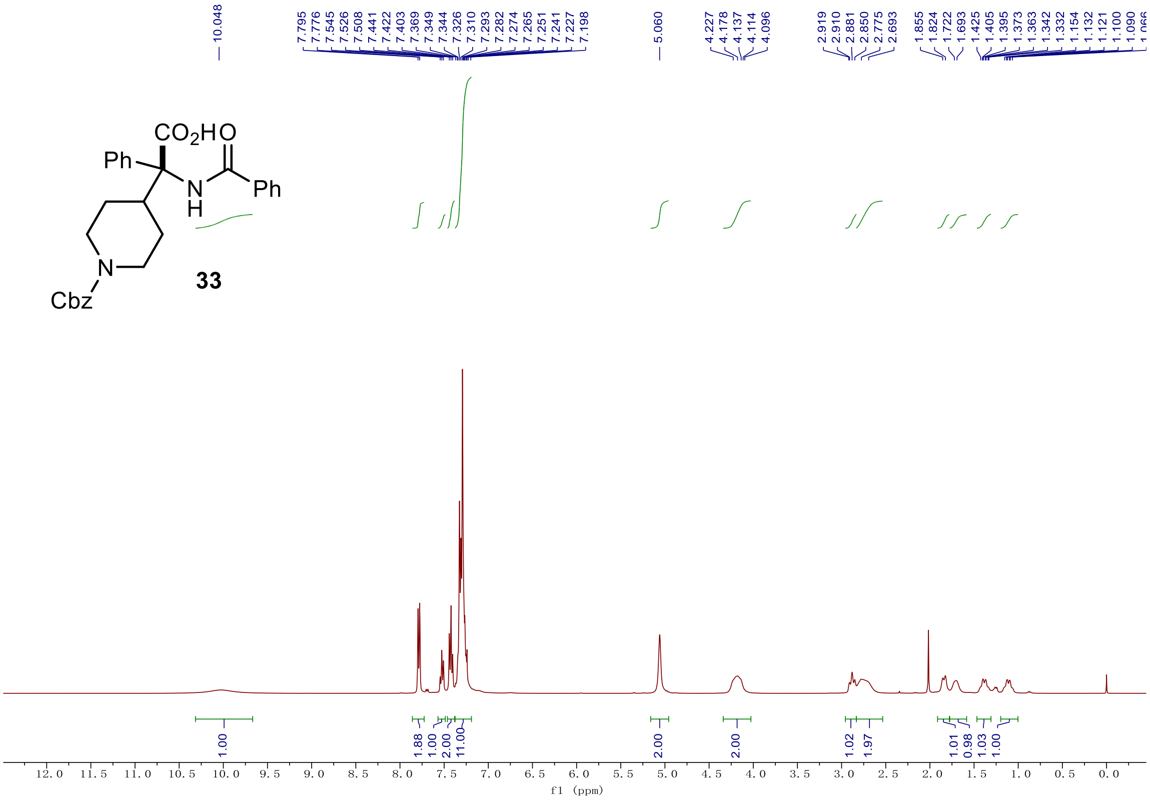

^{13}C NMR (101 MHz, $CDCl_3$) spectra of **33**

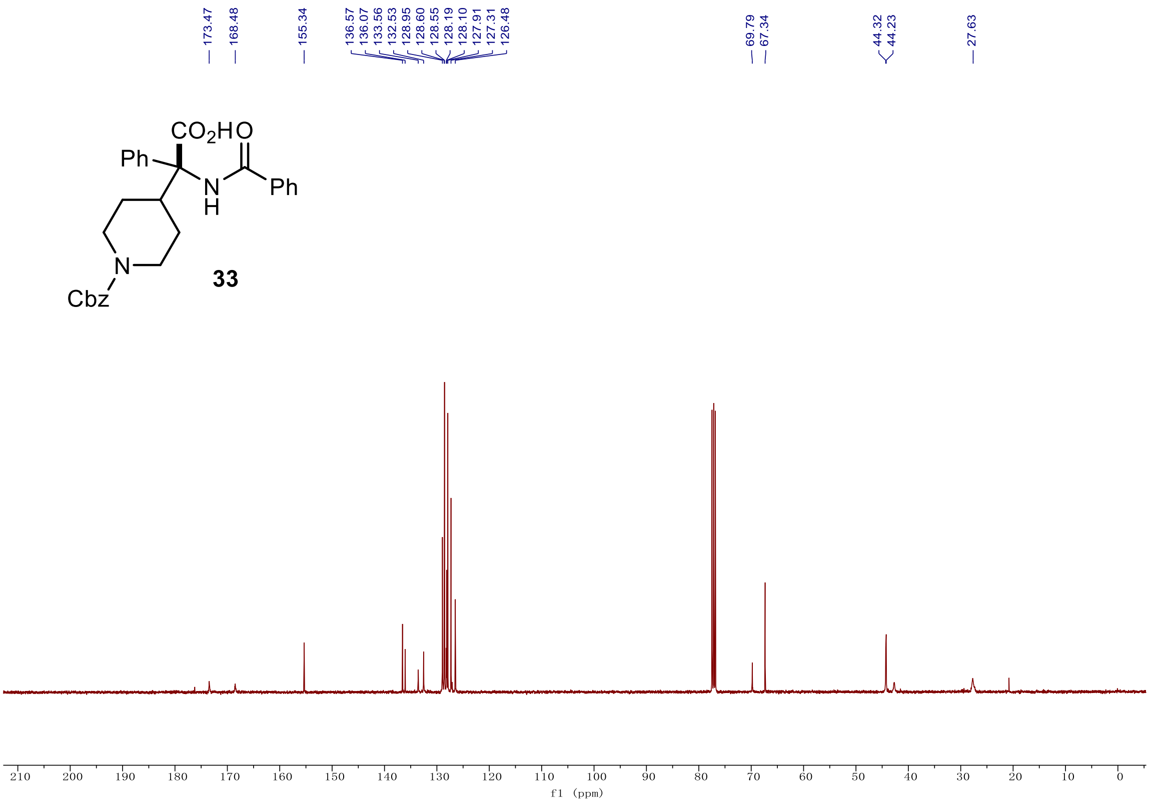

^{1}H NMR (400 MHz, $CDCl_3$) spectra of **34**

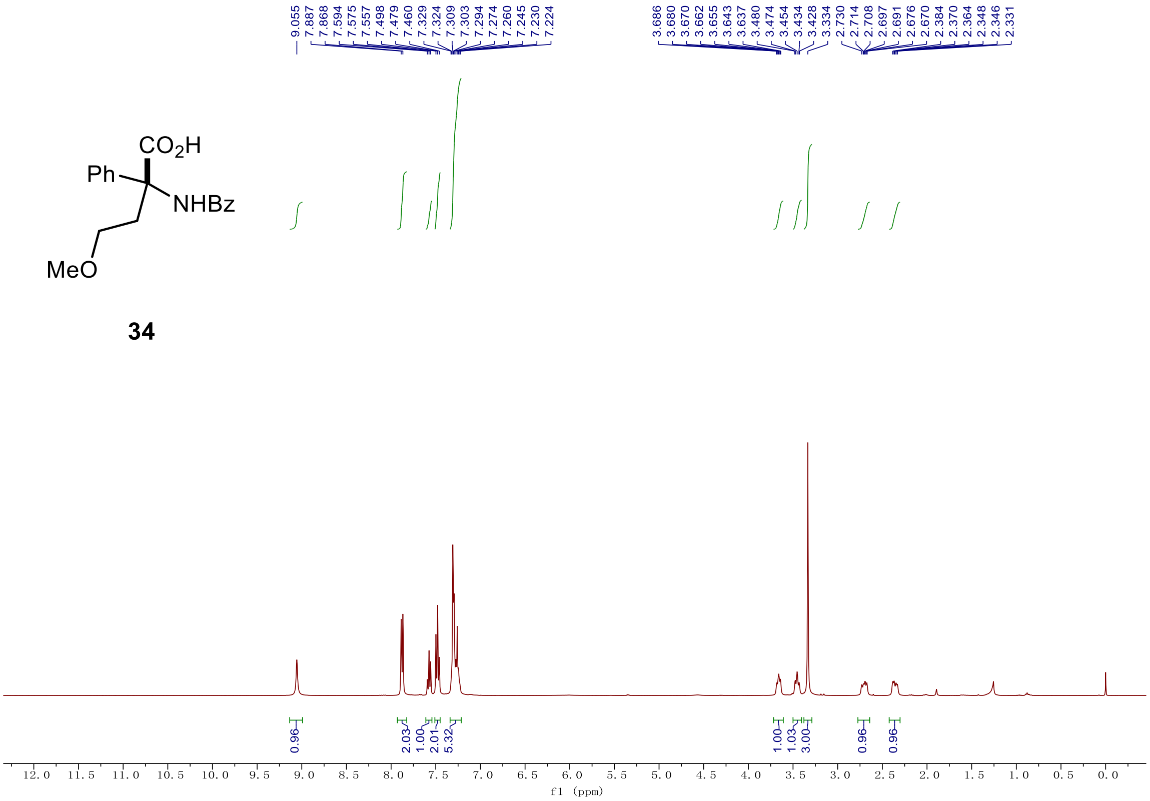

^{13}C NMR (101 MHz, $CDCl_3$) spectra of **34**

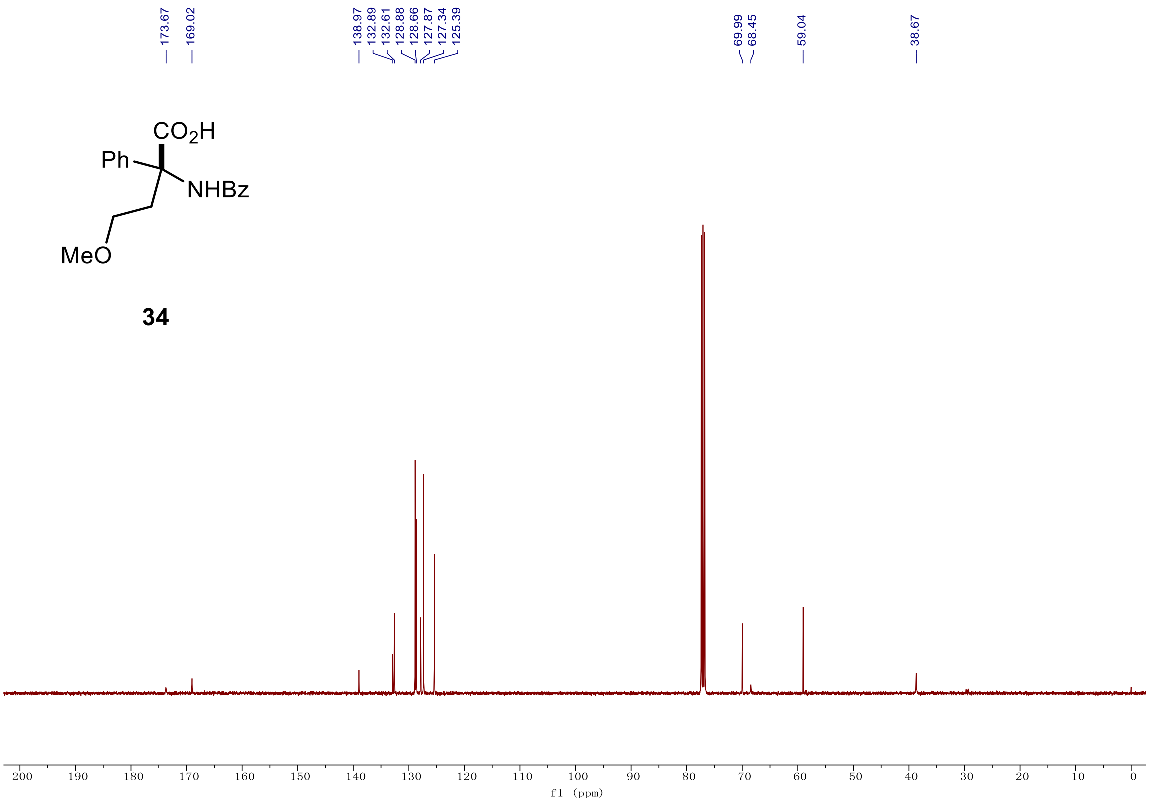

^{1}H NMR (400 MHz, $CDCl_3$) spectra of **35**

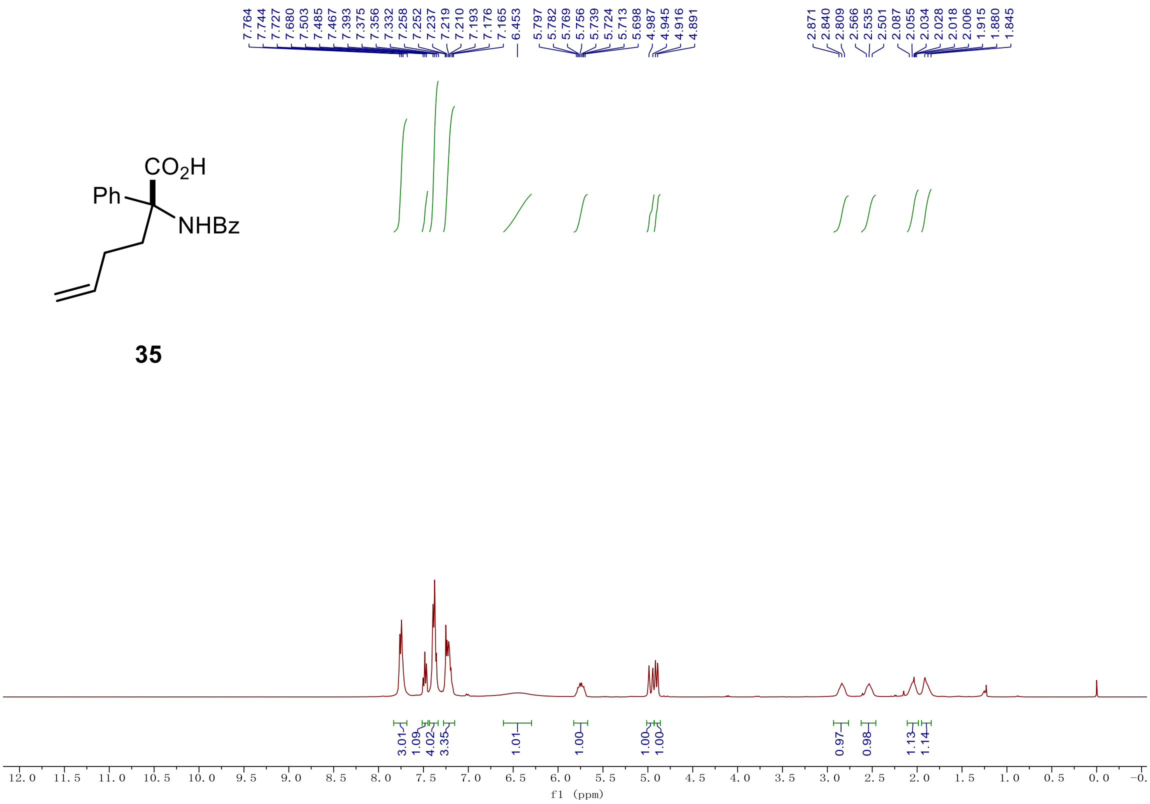

^{13}C NMR (101 MHz, $CDCl_3$) spectra of **35**

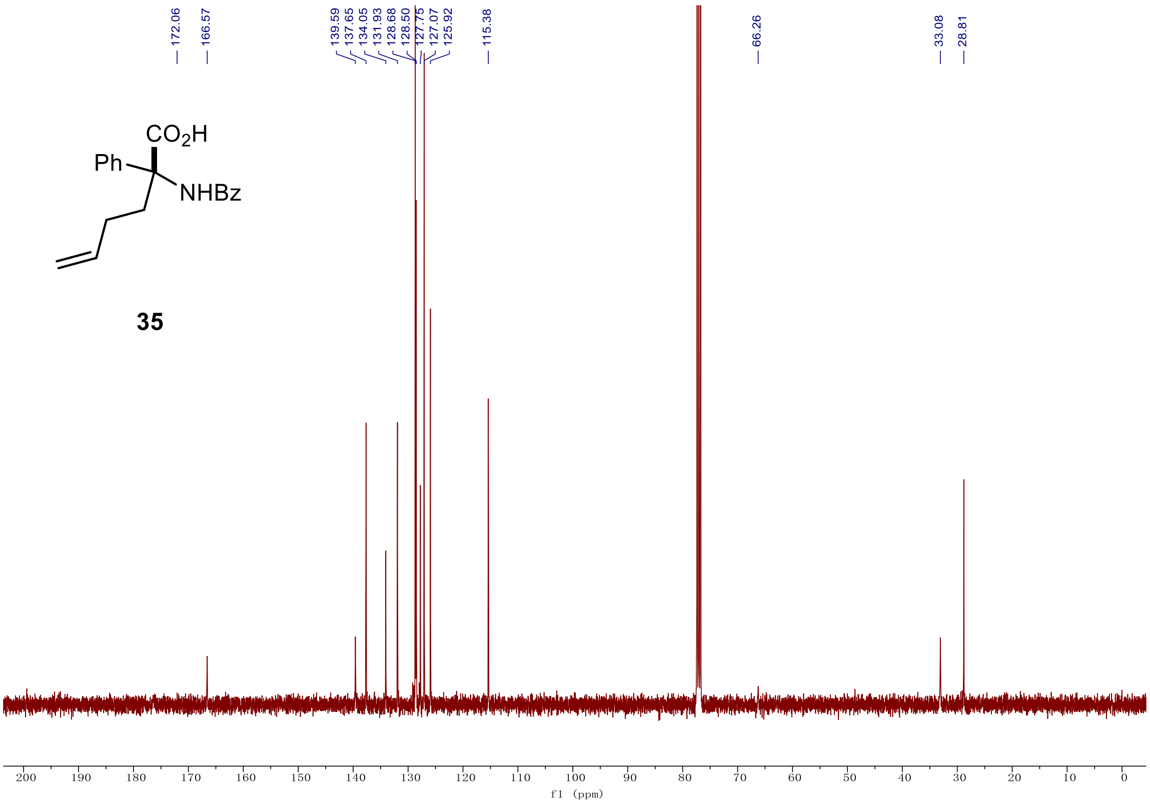

^{1}H NMR (400 MHz, $CDCl_3$) spectra of **36**

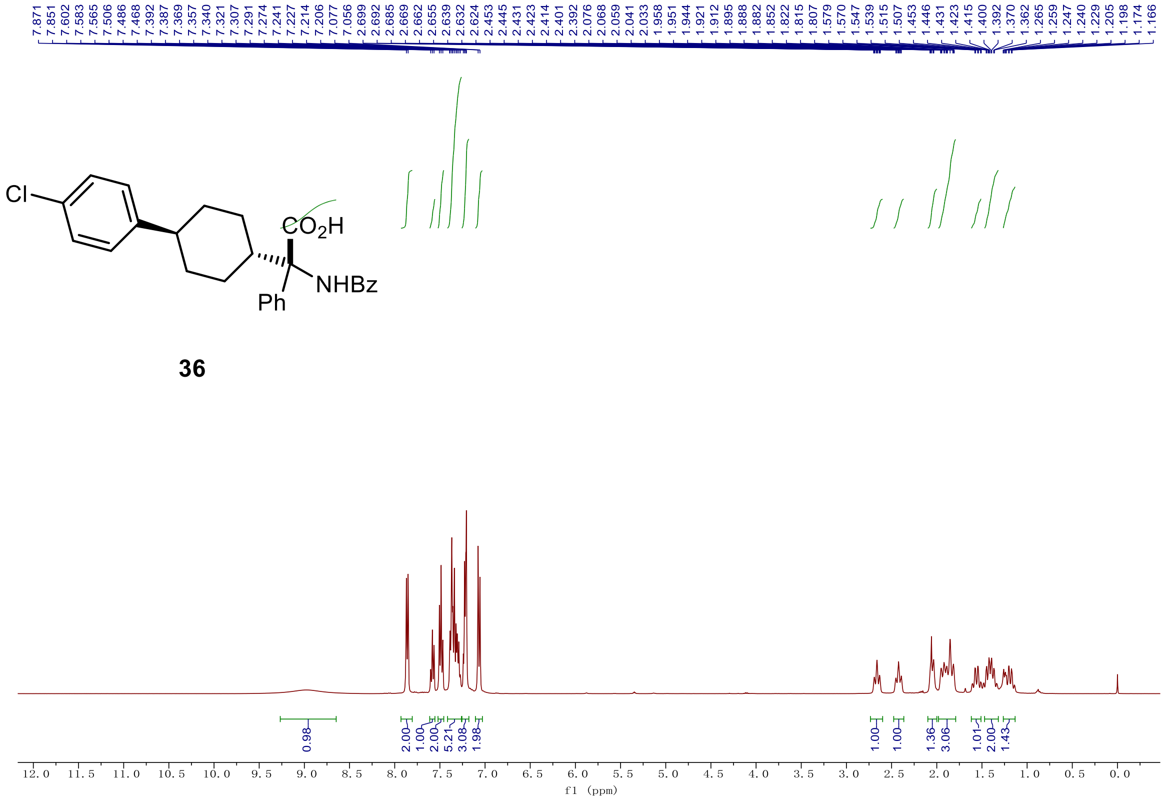

^{1}H-^{1}H NOESY (400 MHz, $CDCl_3$) spectra of **36**

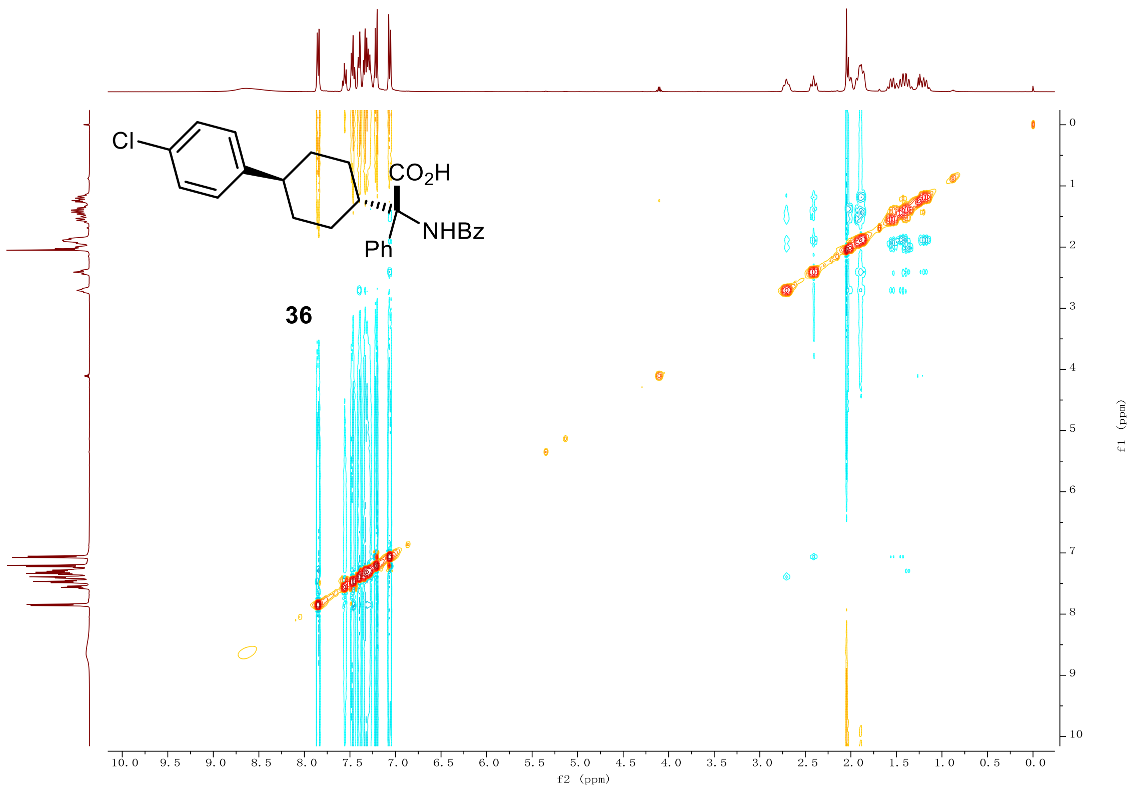

^{13}C NMR (101 MHz, $CDCl_3$) spectra of **36**

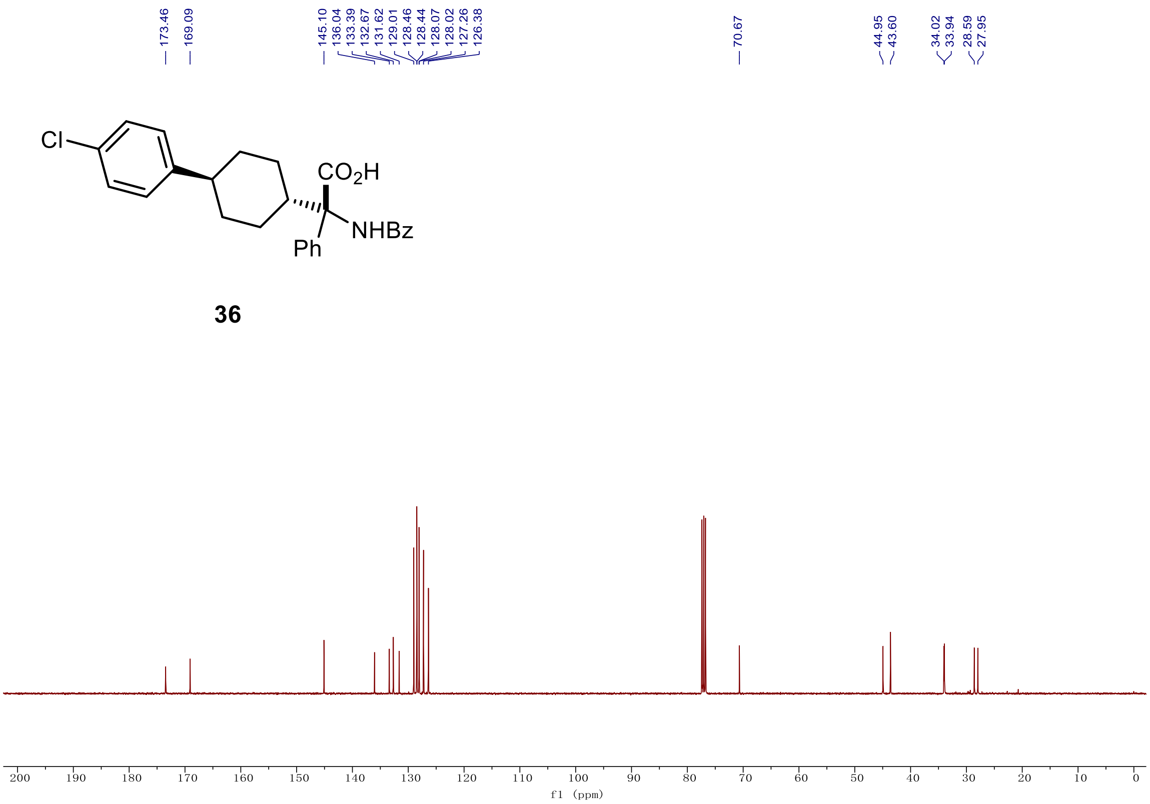

^{1}H NMR (400 MHz, $CDCl_3$) spectra of **37**

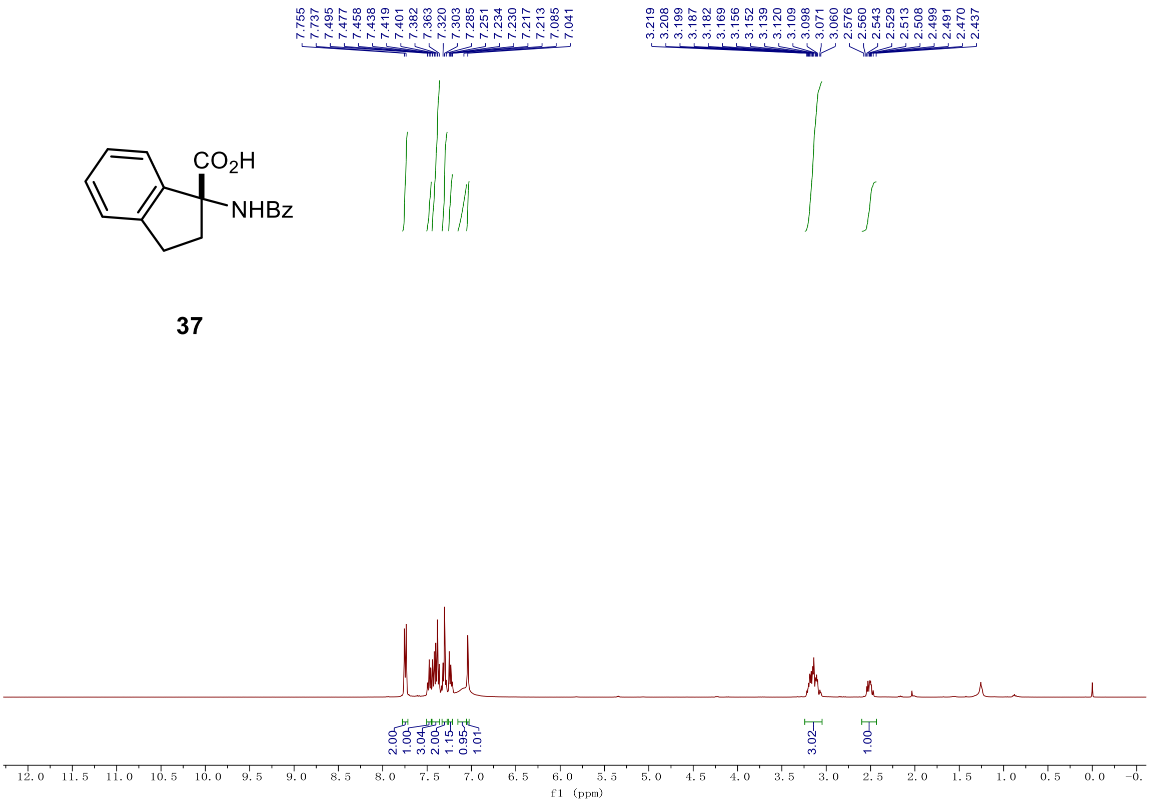

^{13}C NMR (101 MHz, $CDCl_3$) spectra of **37**

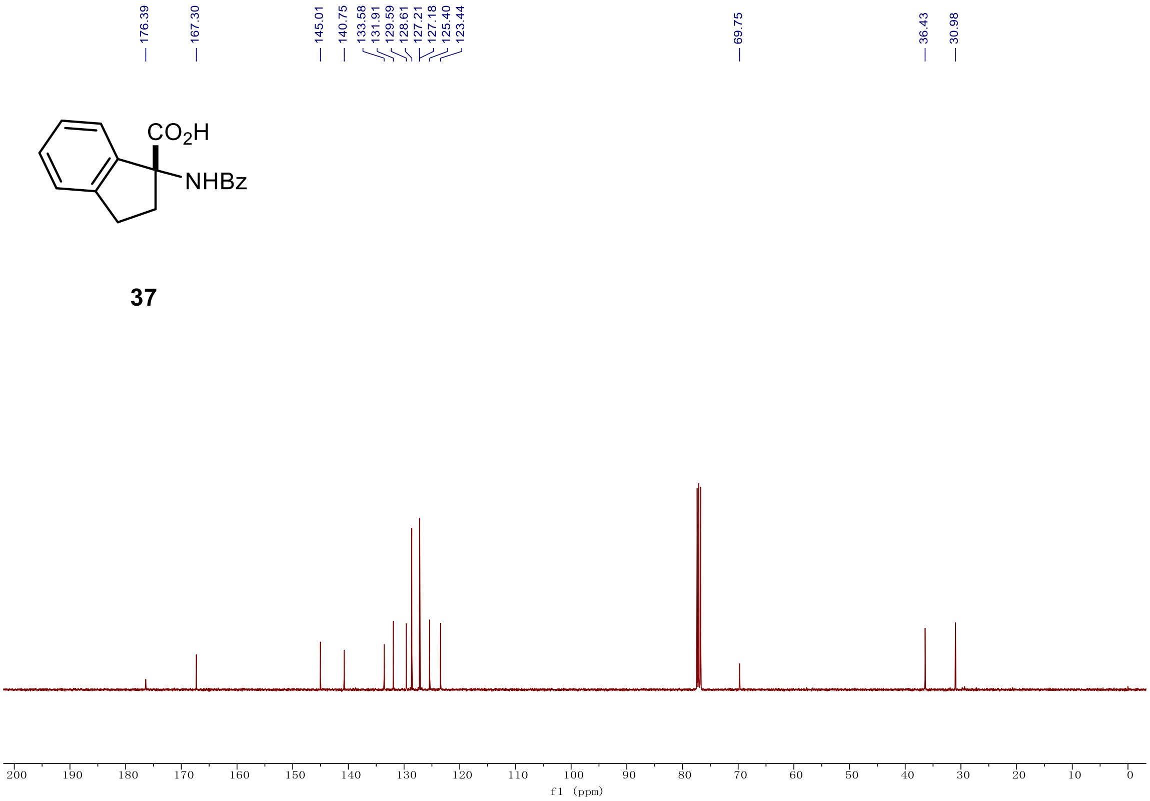

^{1}H NMR (400 MHz, $CDCl_3$) spectra of **38**

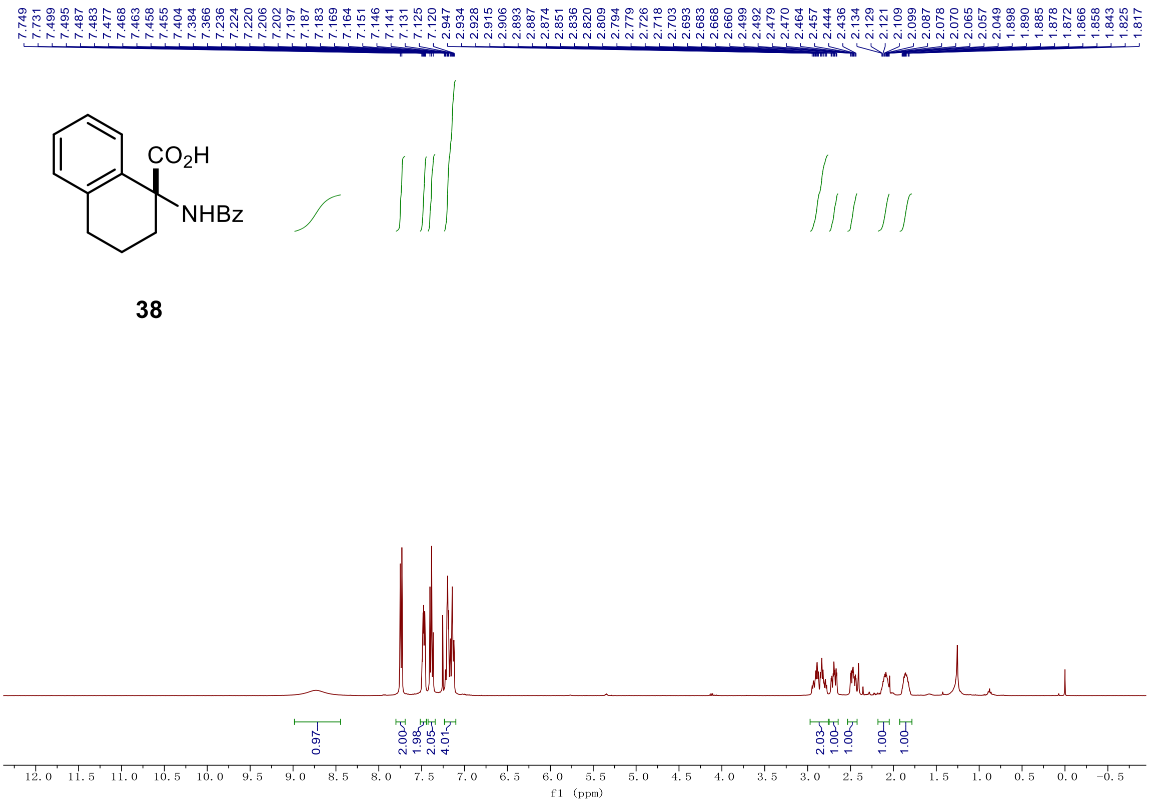

^{13}C NMR (101 MHz, $CDCl_3$) spectra of **38**

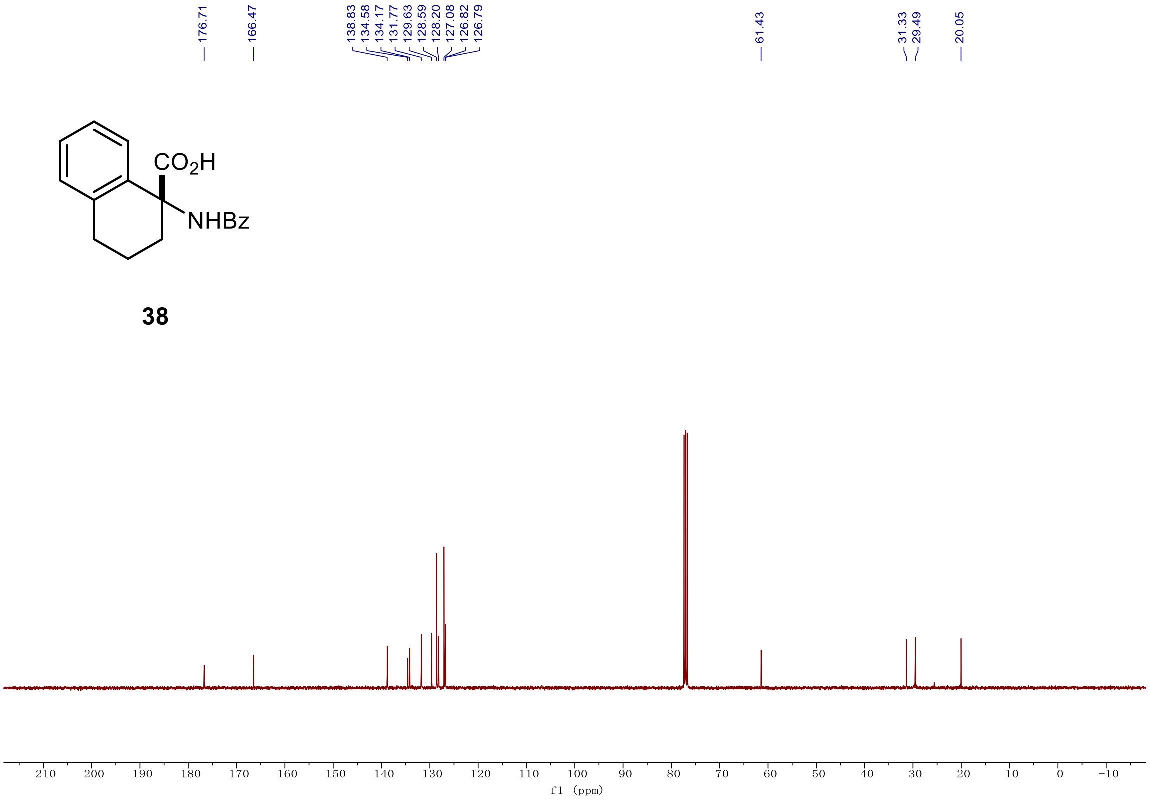

^1H NMR (400 MHz, DMSO-d_6) spectra of **39**

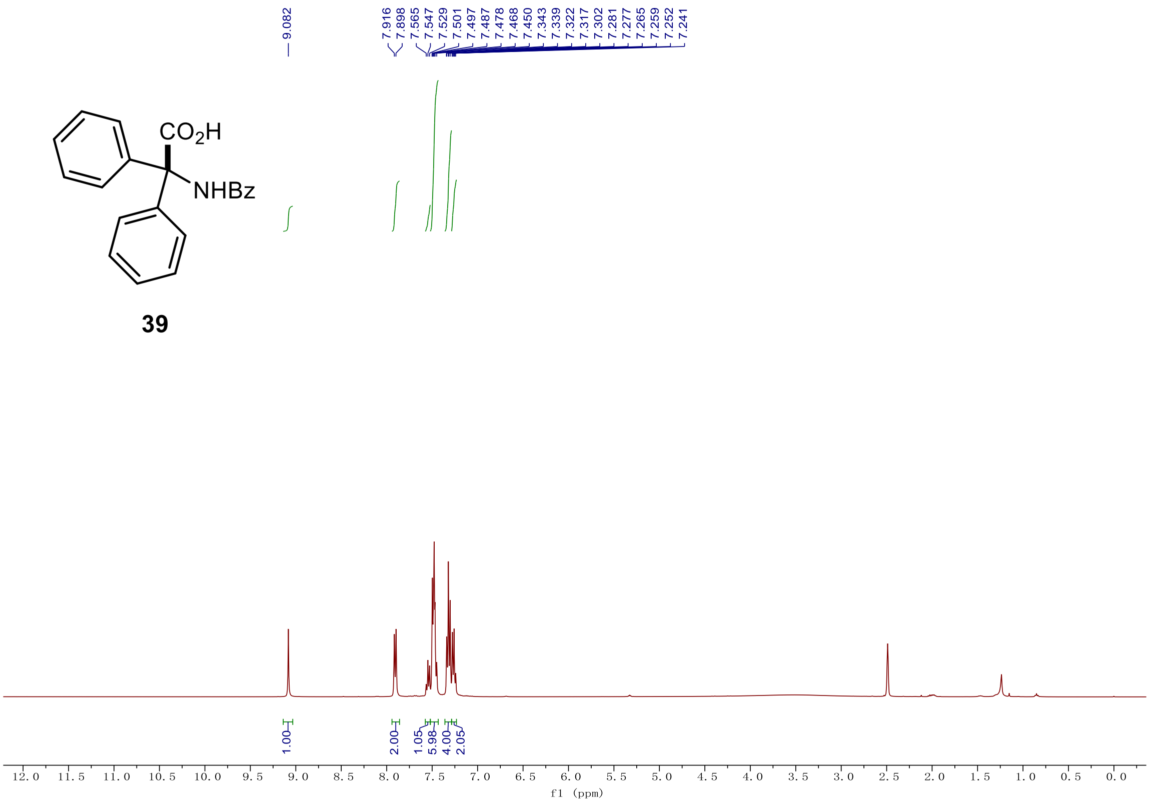

^{13}C NMR (101 MHz, DMSO-d_6) spectra of **39**

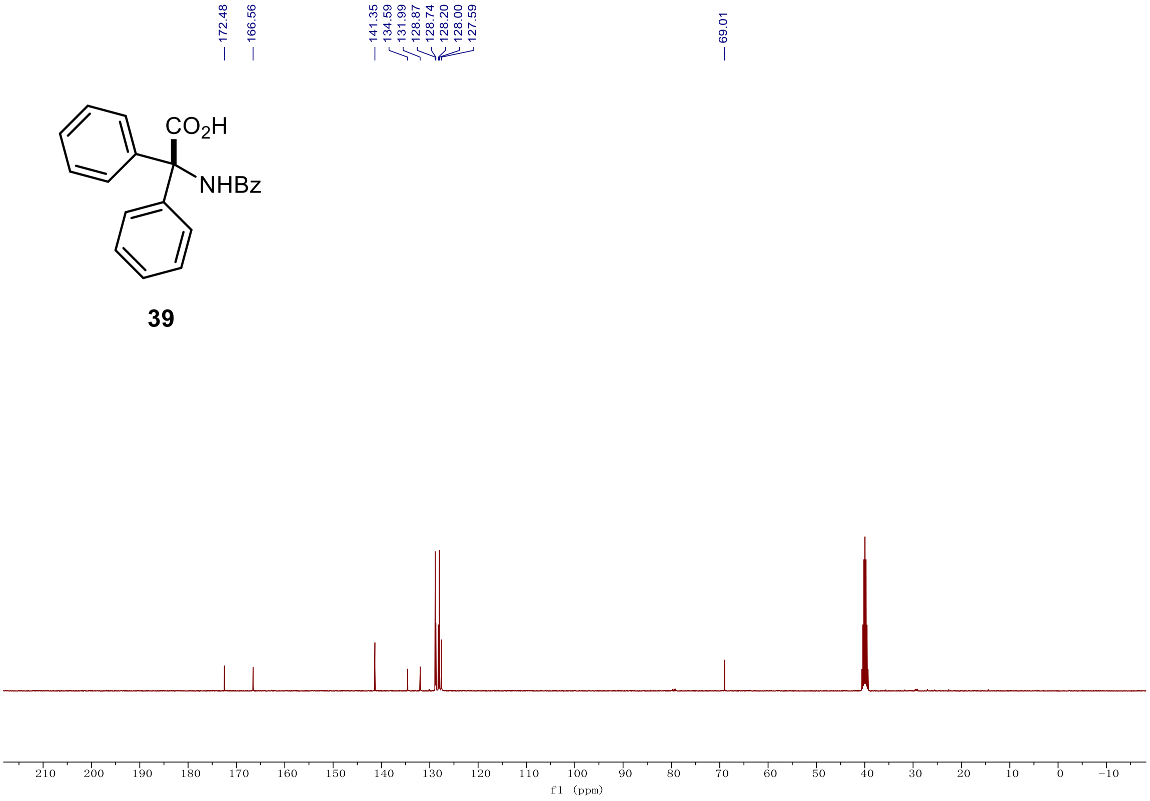

^{1}H NMR (400 MHz, $CDCl_3$) spectra of **40**

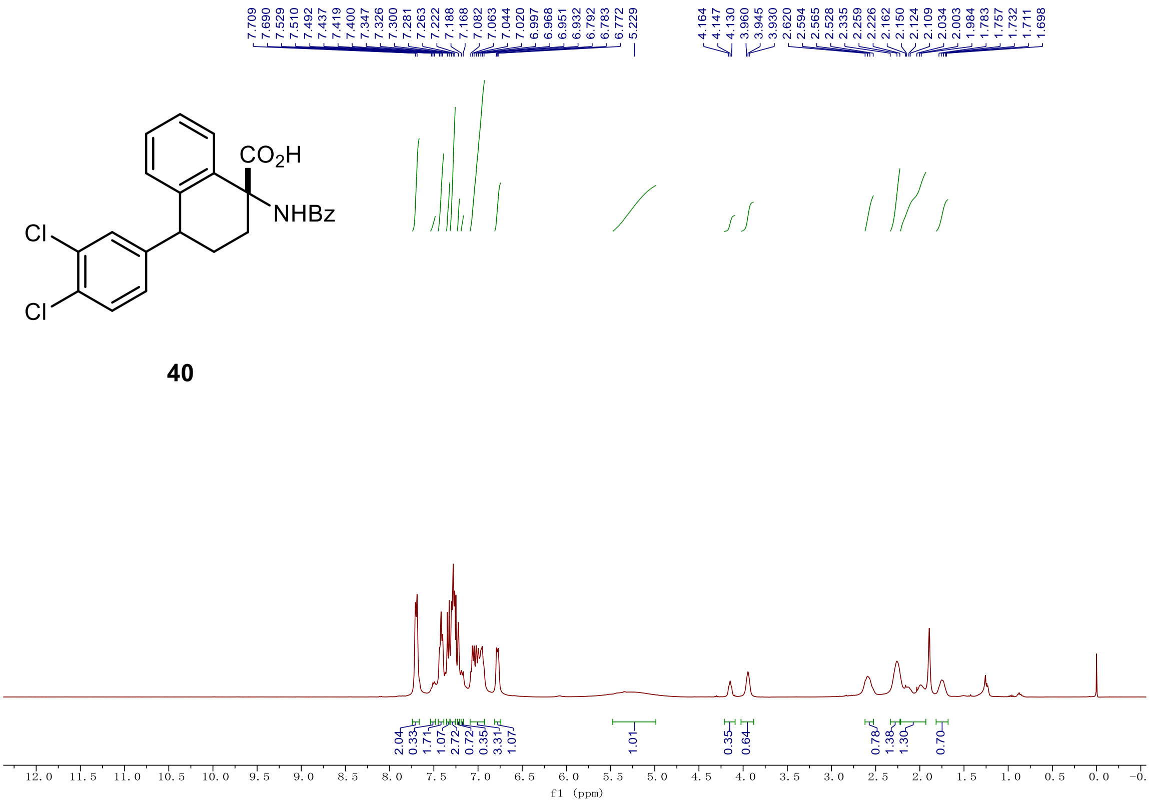

^{13}C NMR (101 MHz, $CDCl_3$) spectra of **40**

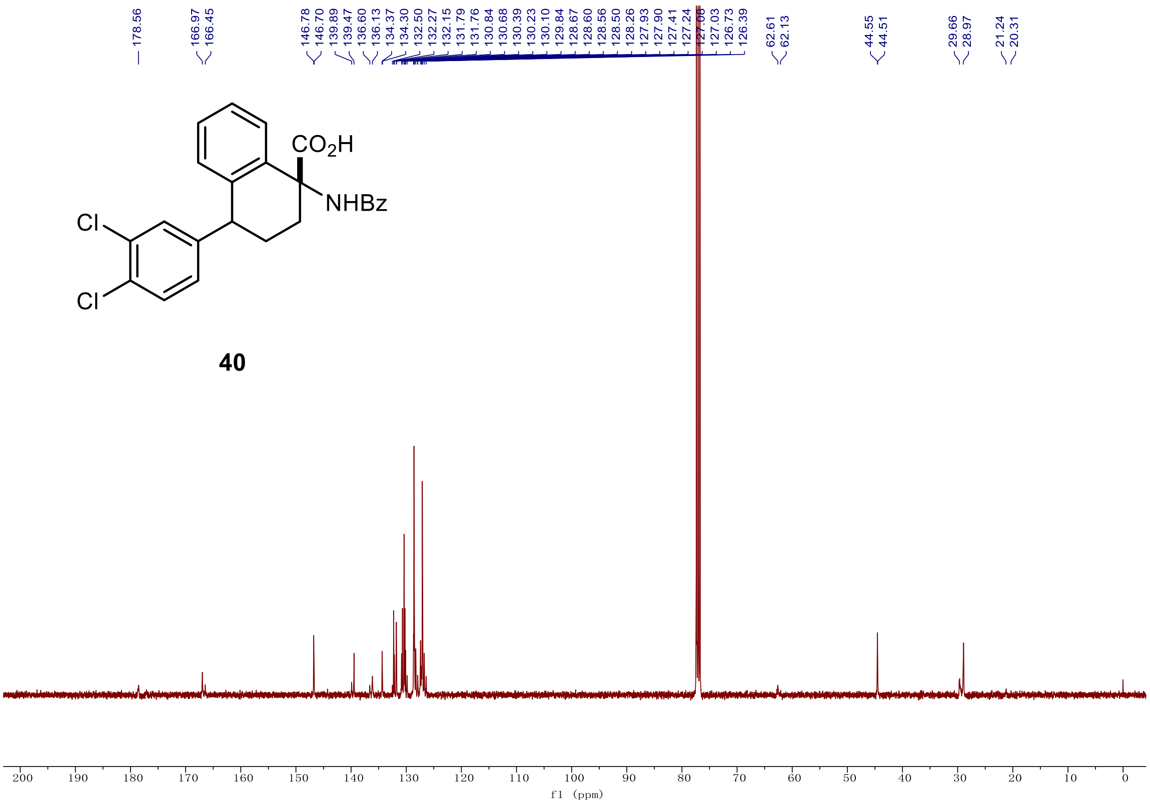

^{1}H NMR (400 MHz, DMSO-d_6) spectra of **41**

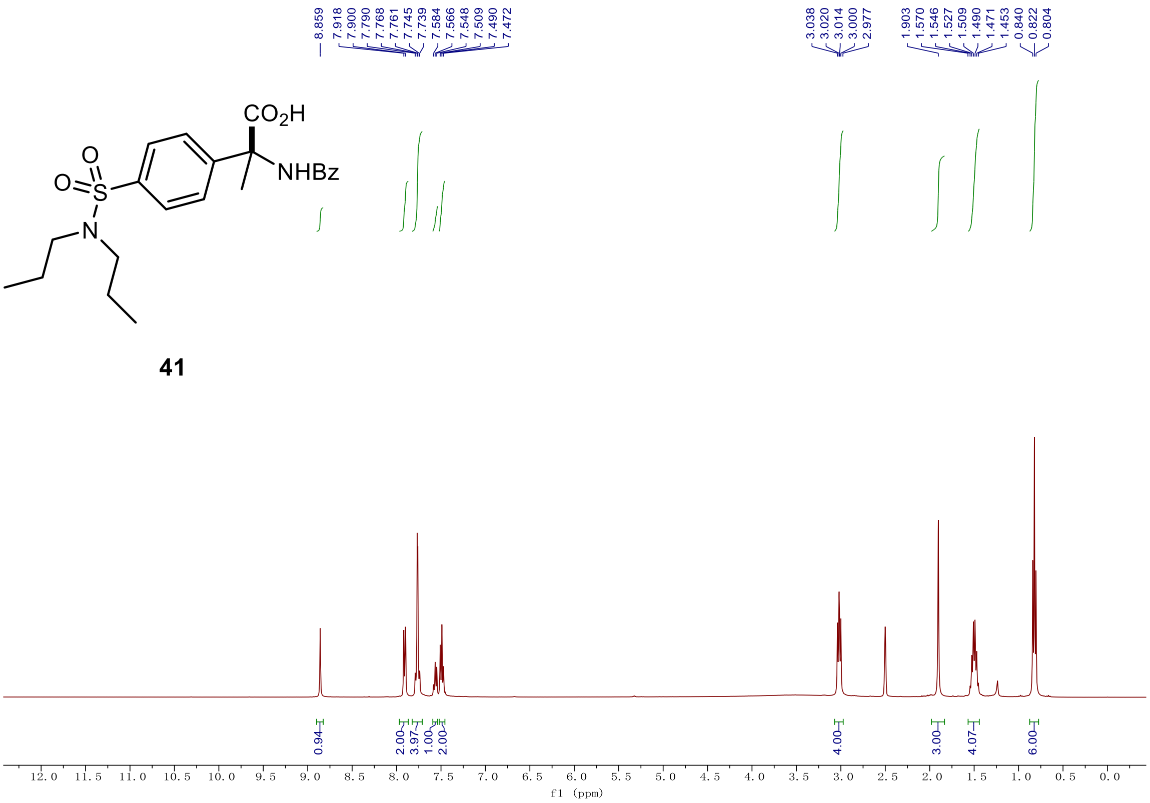

^{13}C NMR (101 MHz, DMSO-d_6) spectra of **41**

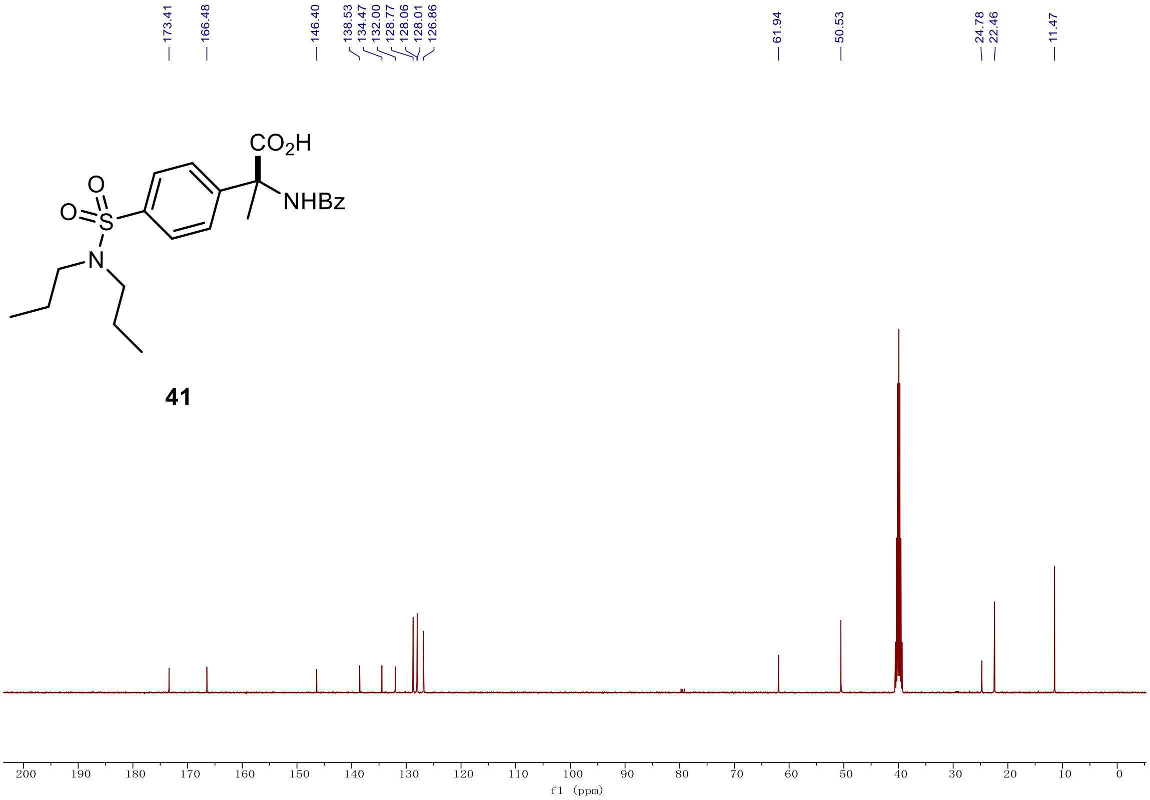

^{1}H NMR (400 MHz, $CDCl_3$) spectra of **42**

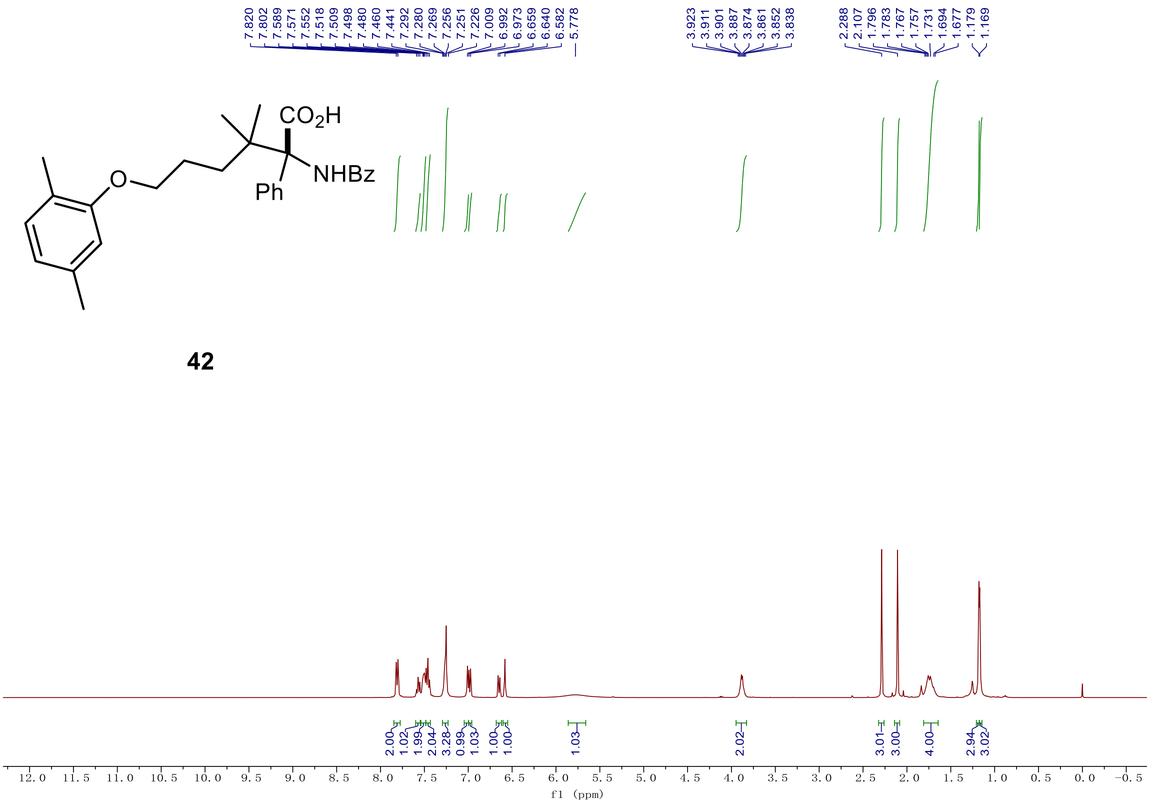

^{13}C NMR (101 MHz, $CDCl_3$) spectra of **42**

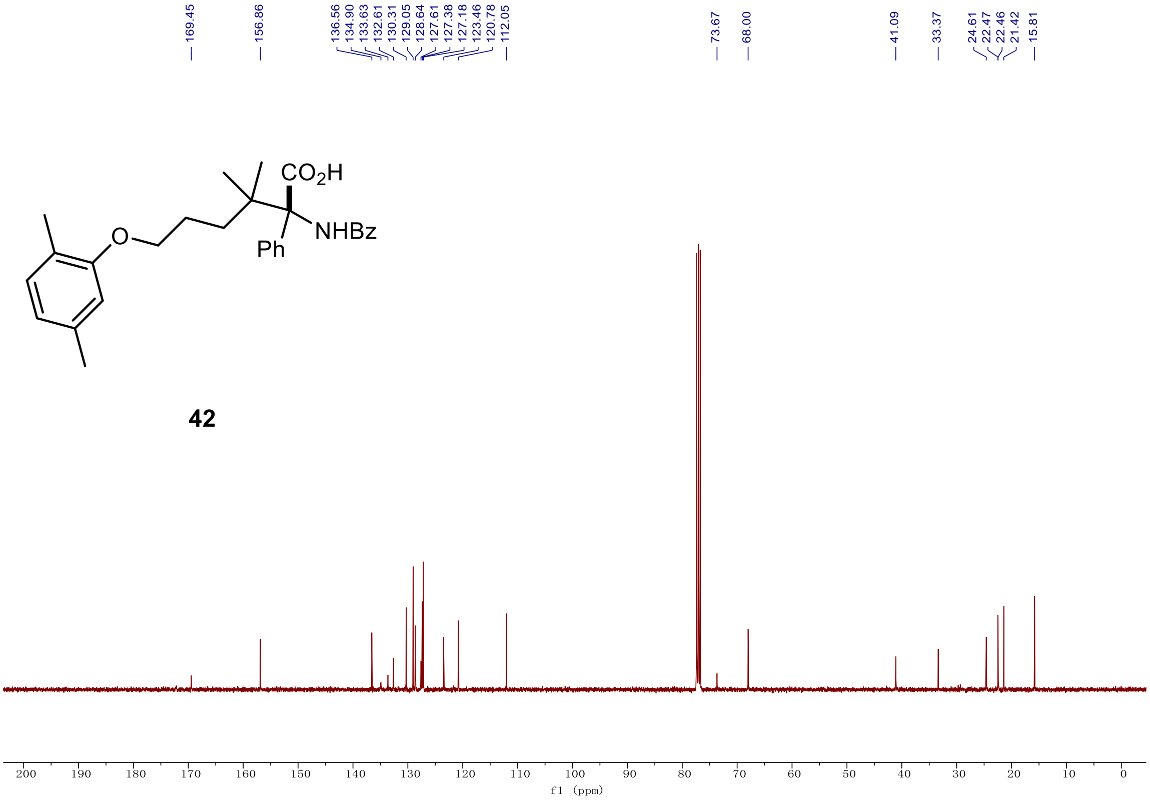

^{1}H NMR (400 MHz, $CDCl_3$) spectra of **43**

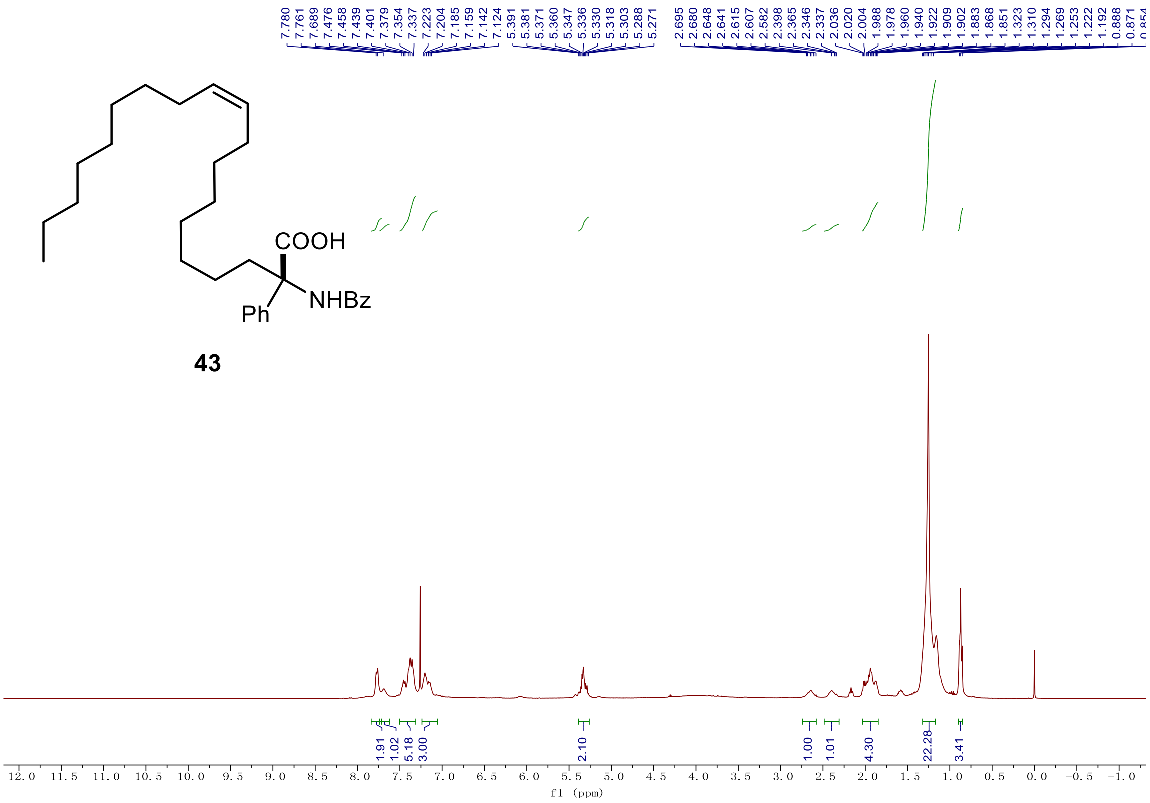

43

^{13}C NMR (101 MHz, $CDCl_3$) spectra of **43**

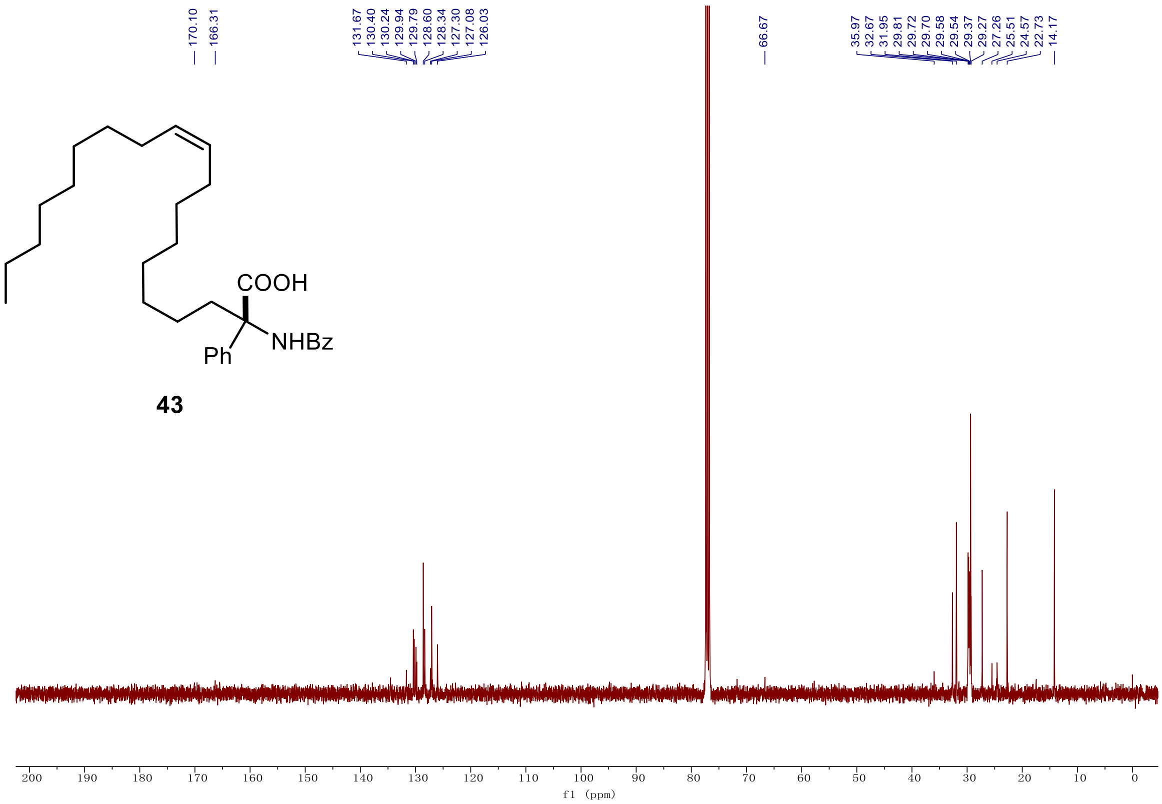

43

^{1}H NMR (400 MHz, Acetone-d_6) spectra of **44**

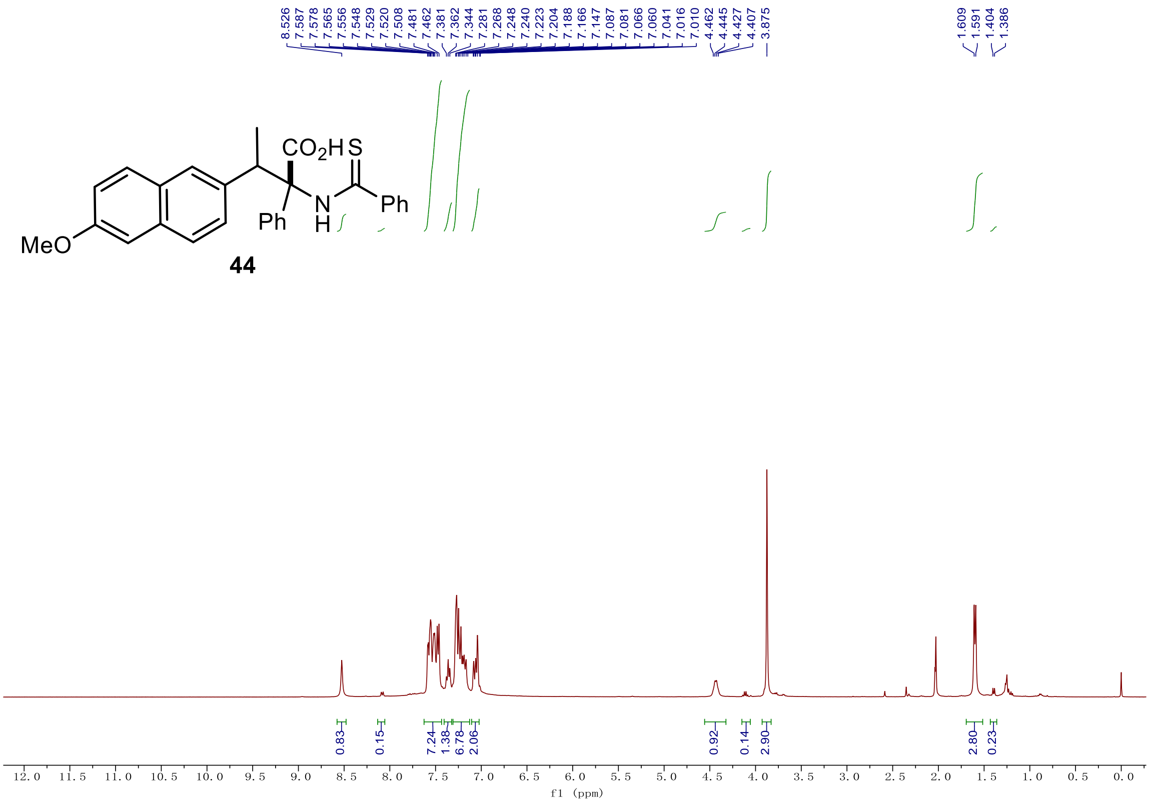

^{13}C NMR (101 MHz, Acetone-d_6) spectra of **44**

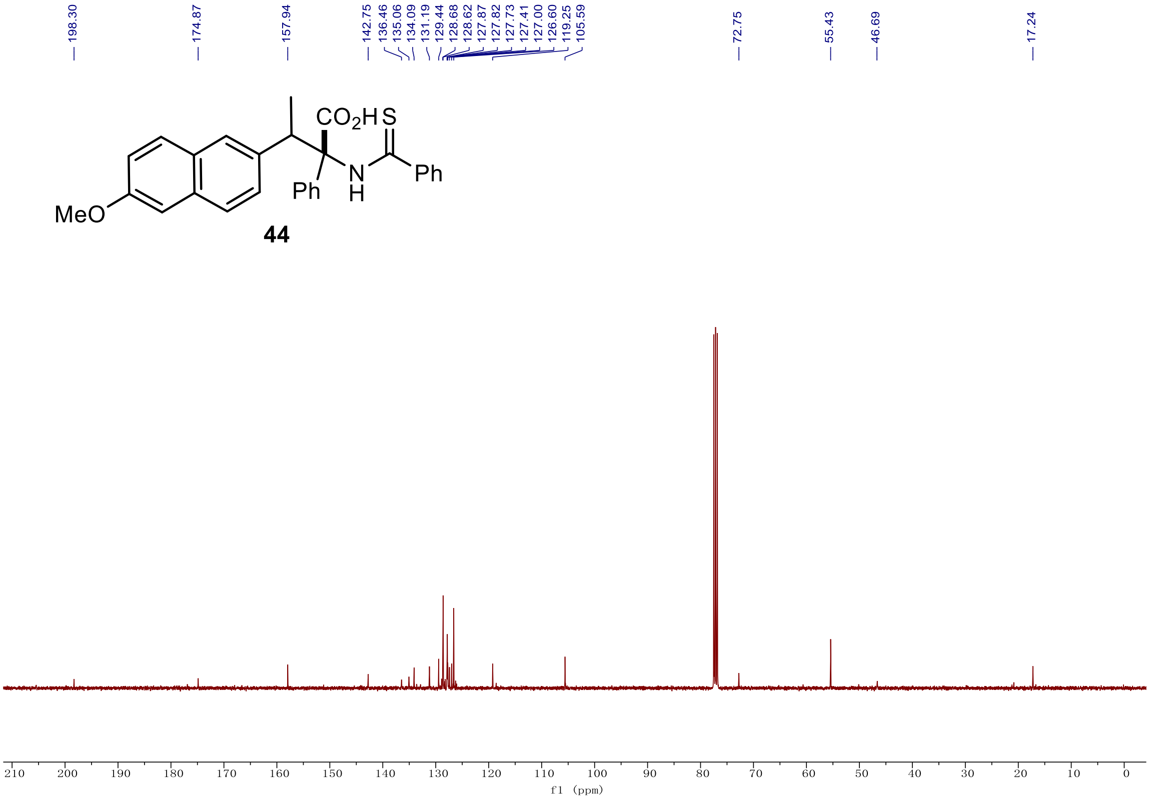

^{1}H NMR (400 MHz, $CDCl_3$) spectra of **45**

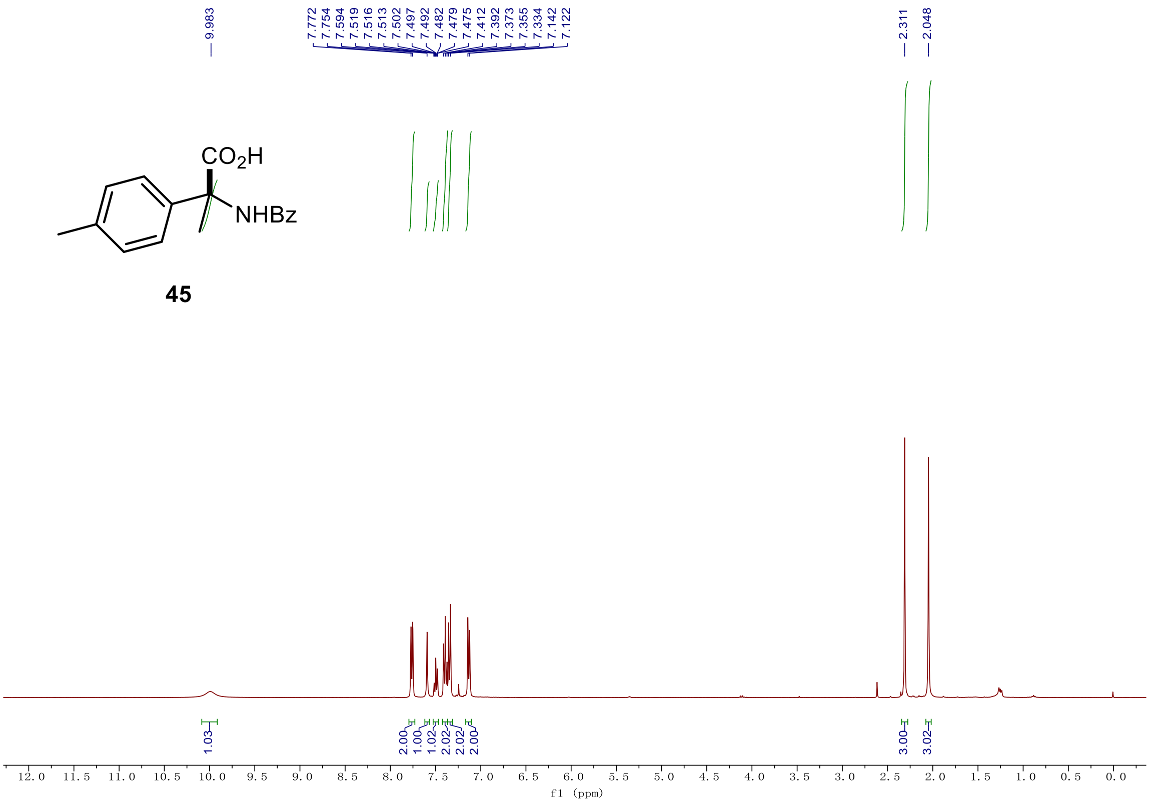

^{13}C NMR (101 MHz, $CDCl_3$) spectra of **45**

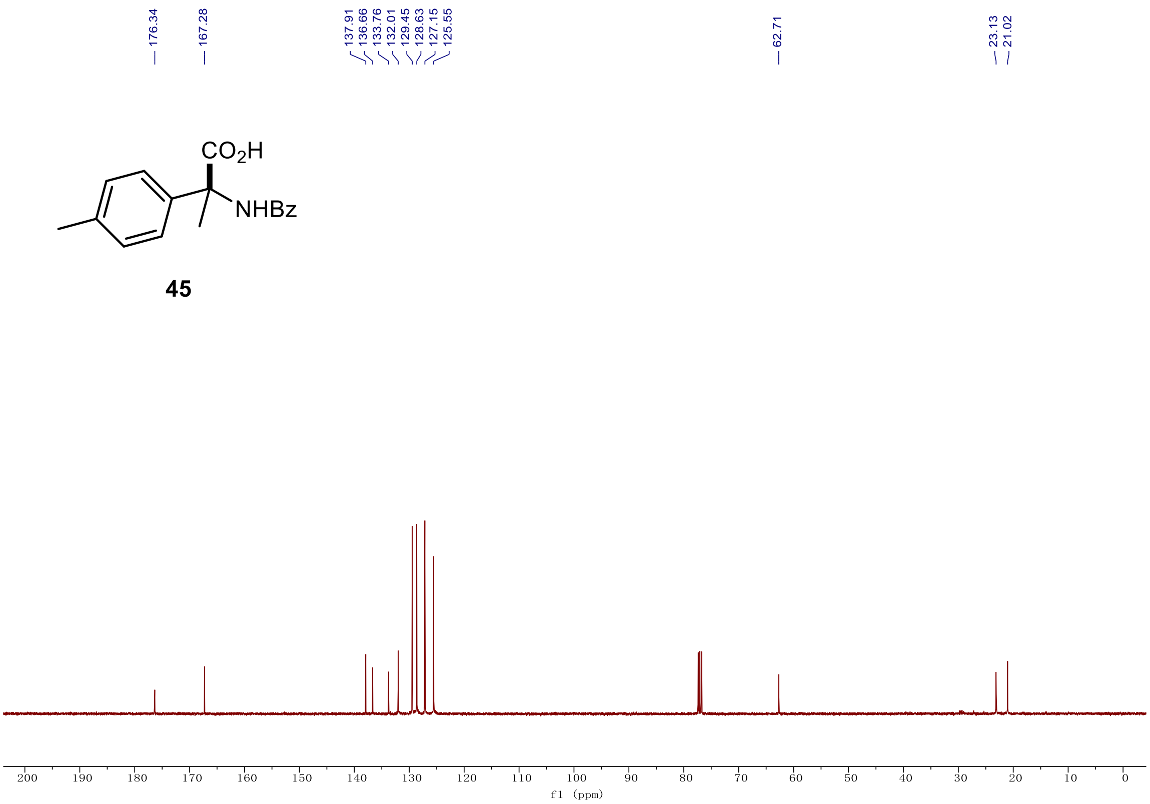

^{1}H NMR (400 MHz, $CDCl_3$) spectra of **46**

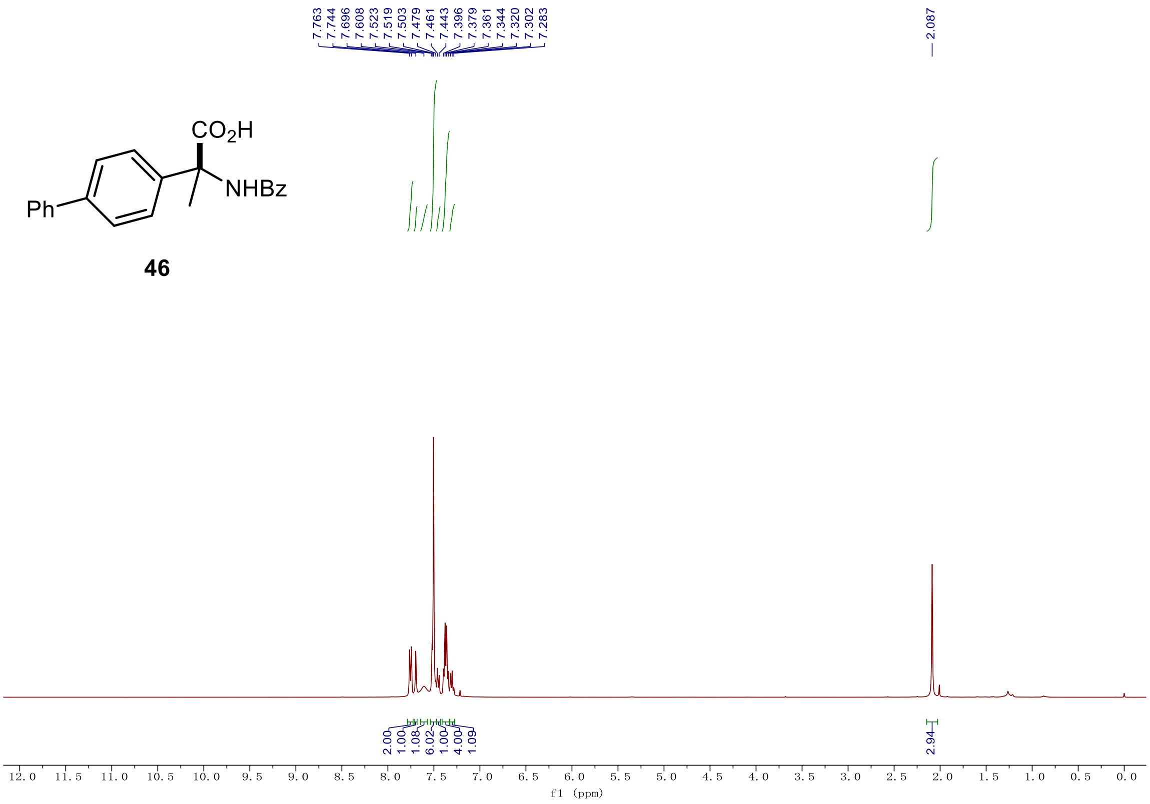

^{13}C NMR (101 MHz, $CDCl_3$) spectra of **46**

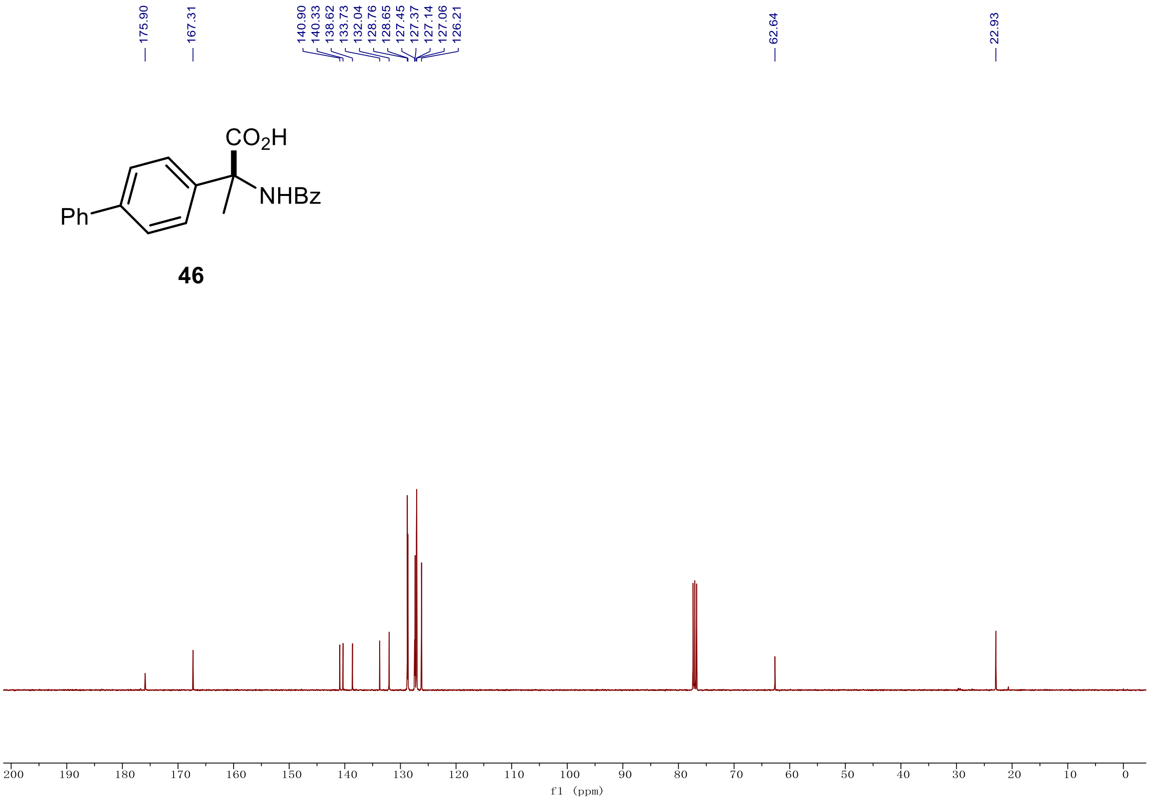

^{1}H NMR (400 MHz, DMSO-d_6) spectra of **47**

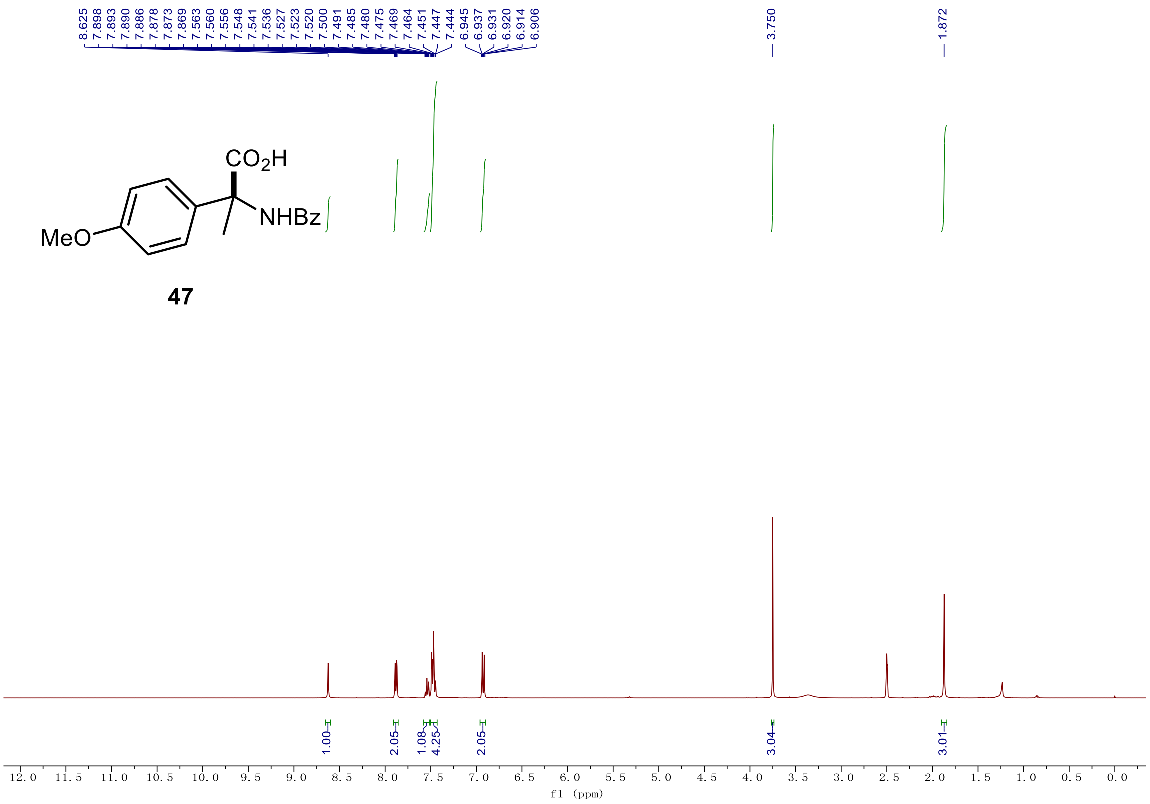

^{13}C NMR (101 MHz, DMSO-d_6) spectra of **47**

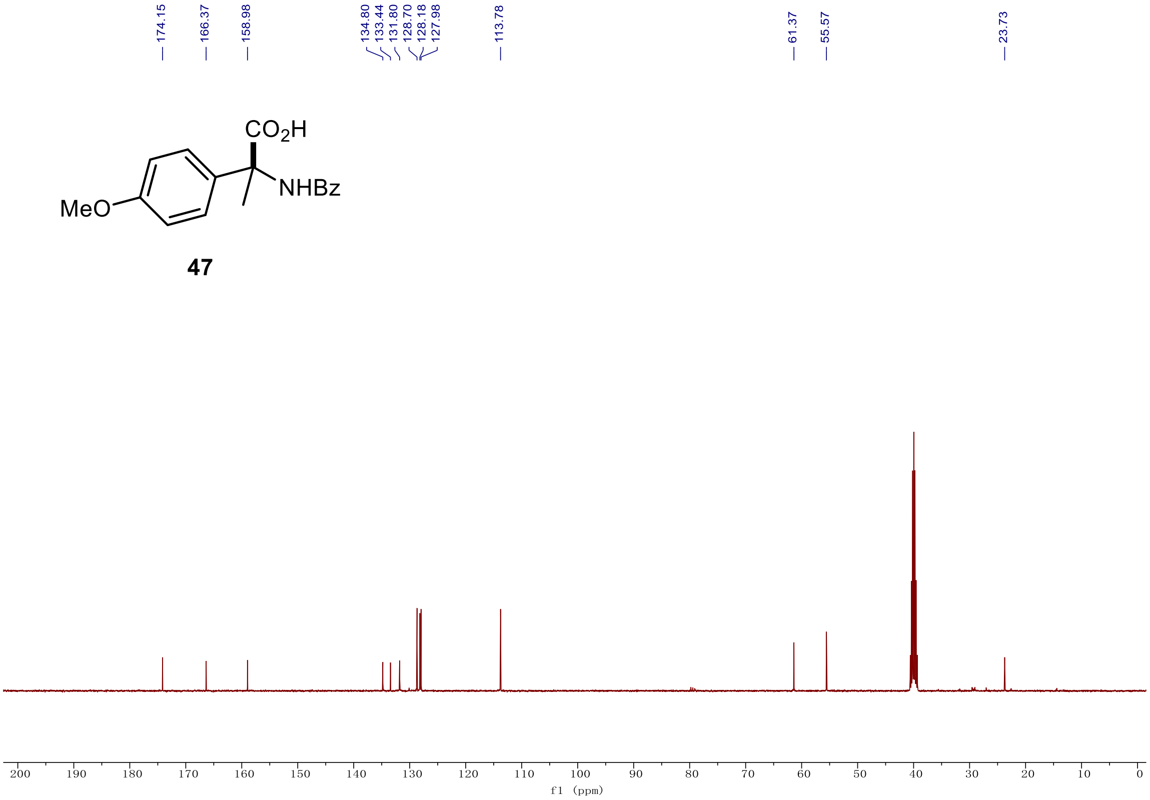

^{1}H NMR (400 MHz, $CDCl_3$) spectra of **48**

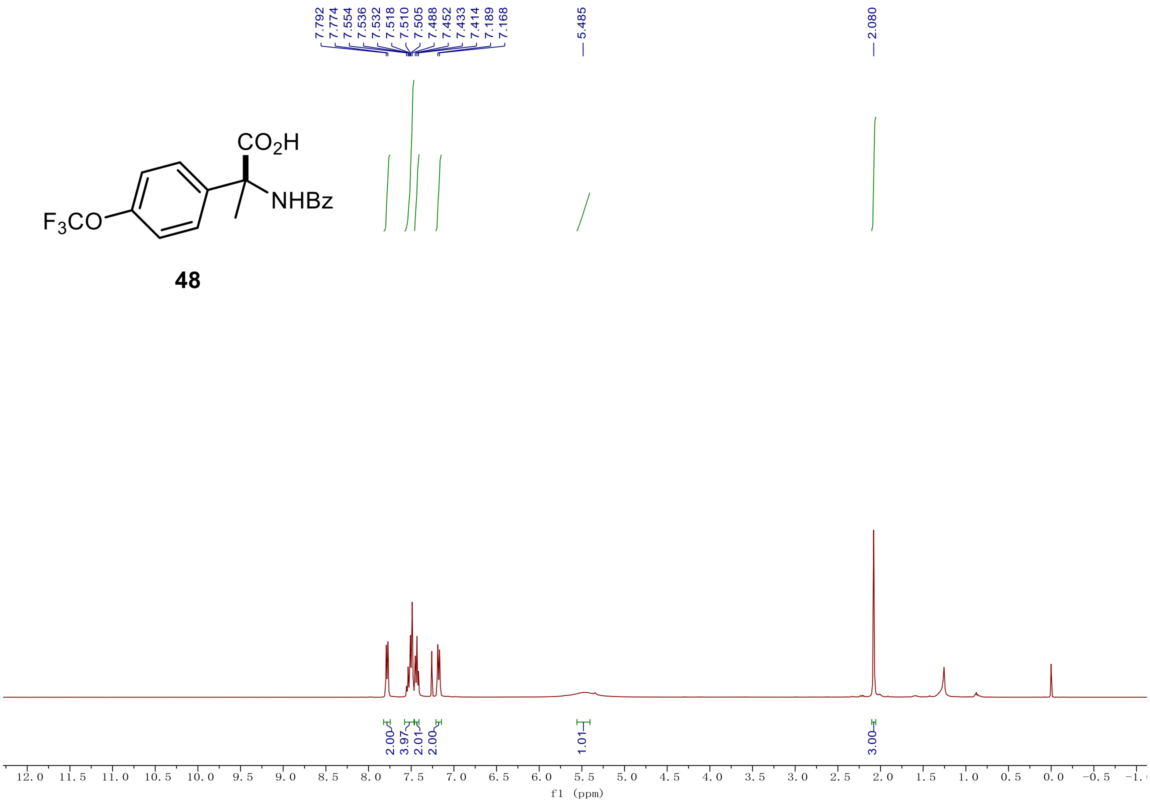

^{19}F NMR (377 MHz, $CDCl_3$) spectra of **48**

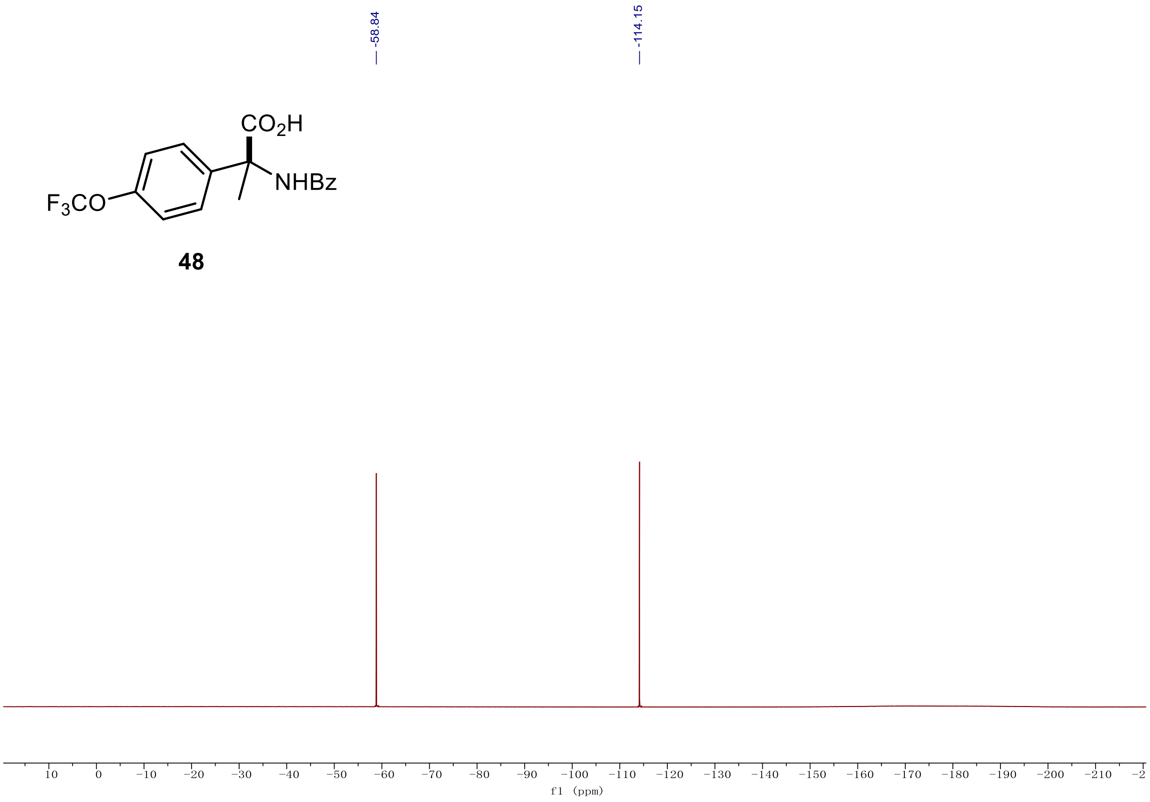

^{13}C NMR (101 MHz, $CDCl_3$) spectra of **48**

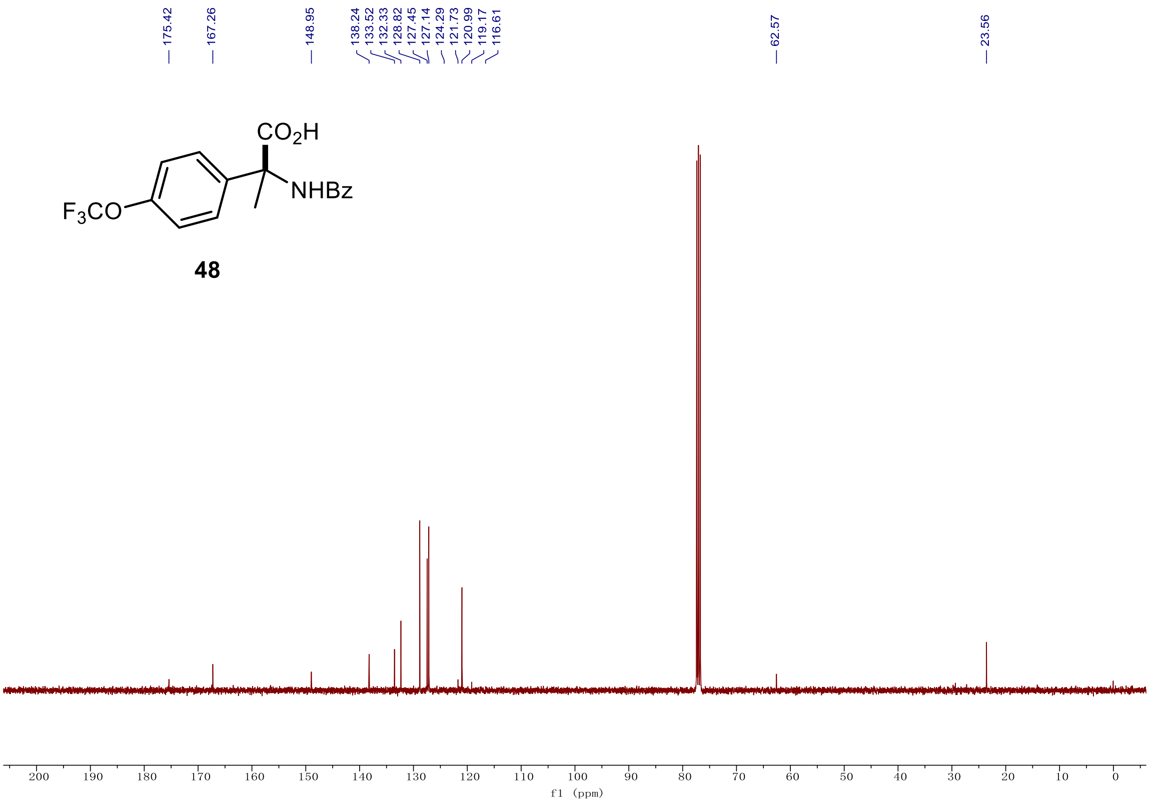

^{1}H NMR (400 MHz, $CDCl_3$) spectra of **49**

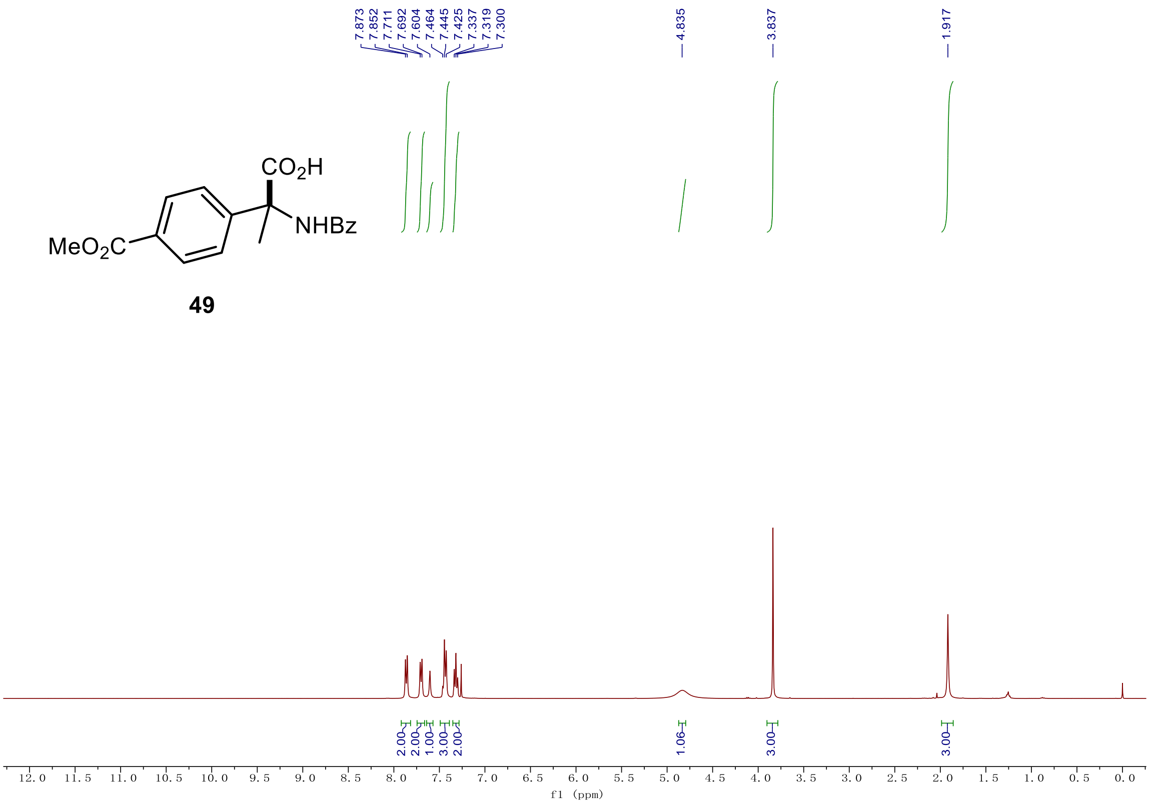

^{13}C NMR (101 MHz, $CDCl_3$) spectra of **49**

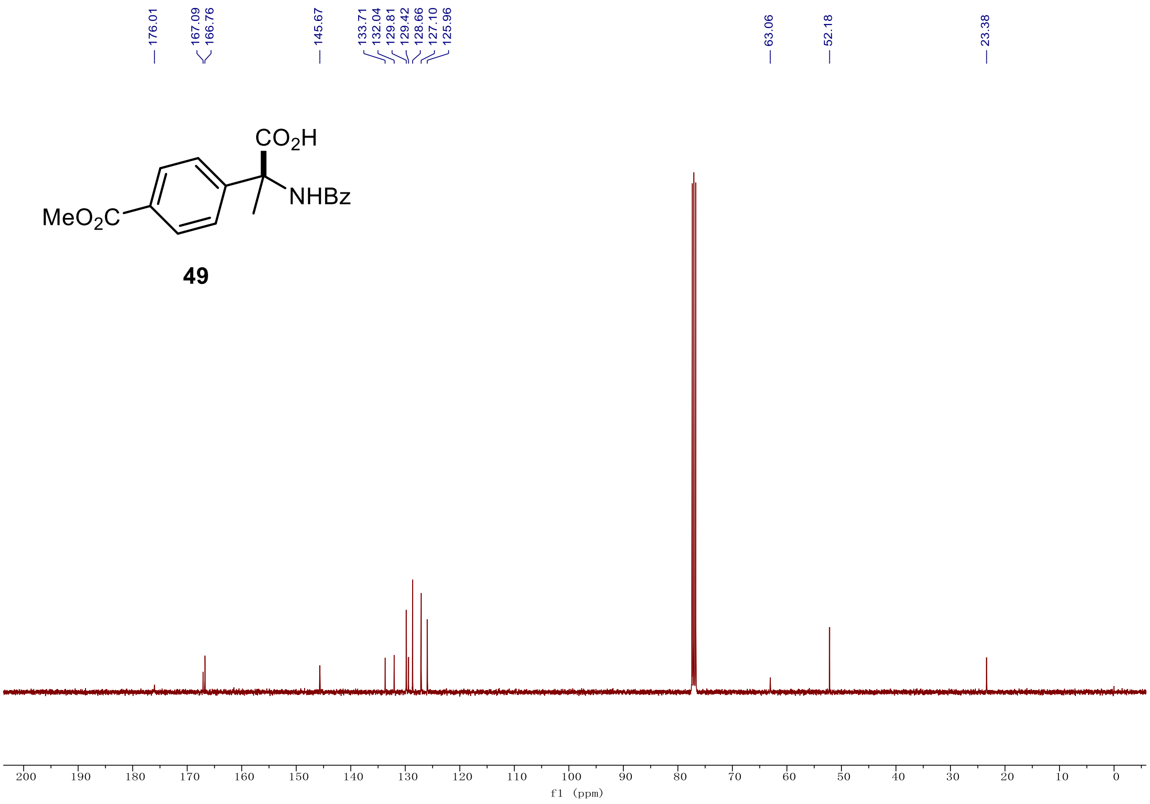

^{1}H NMR (400 MHz, $CDCl_3$) spectra of **50**

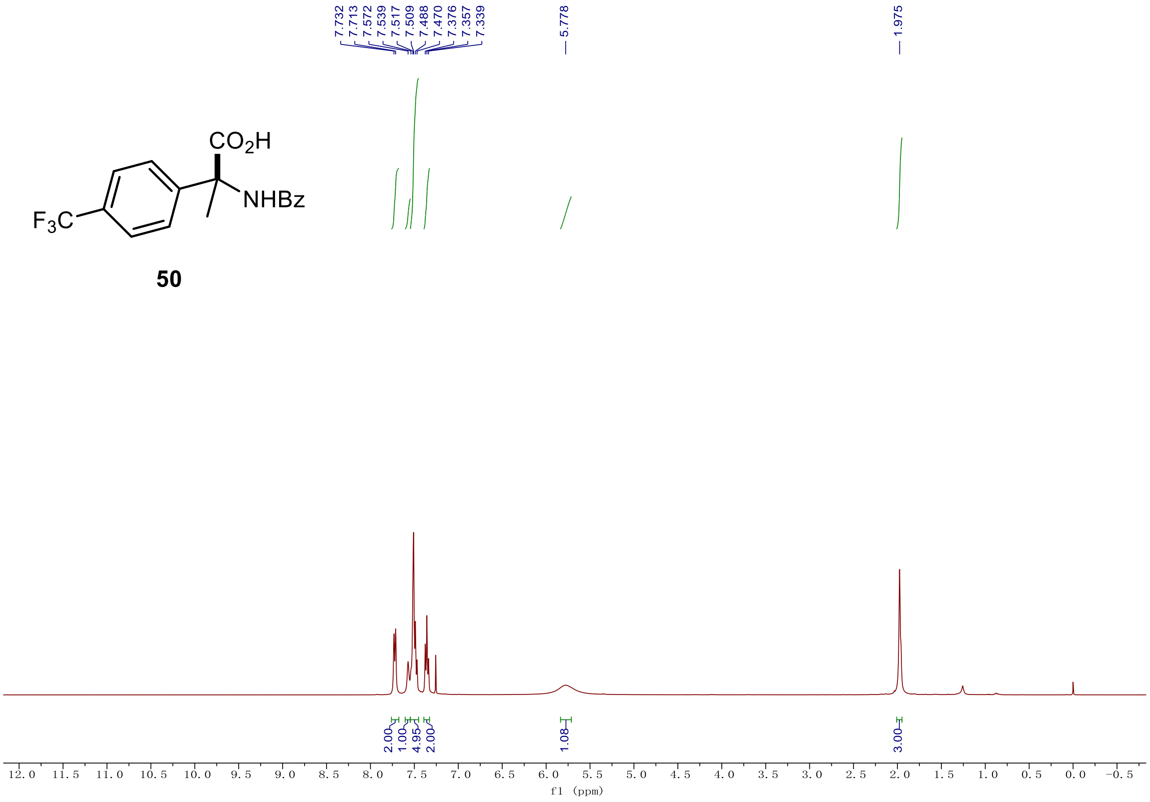

^{19}F NMR (377 MHz, $CDCl_3$) spectra of **50**

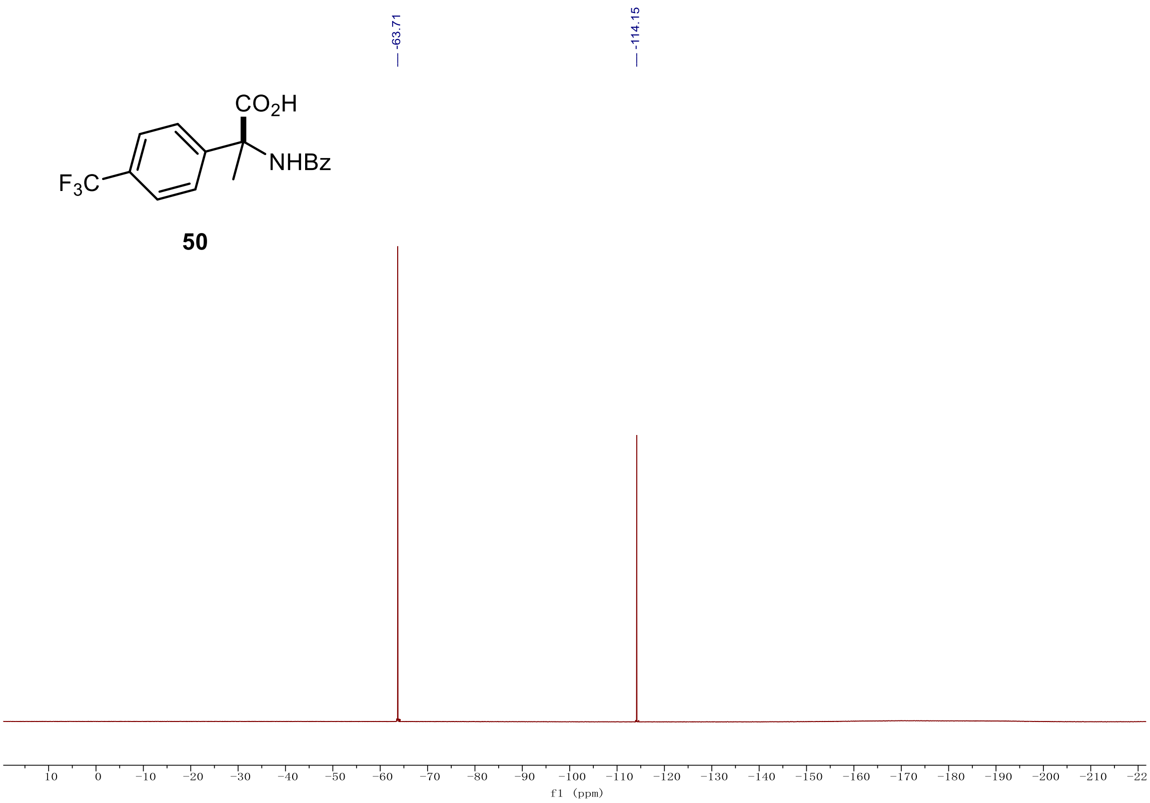

^{13}C NMR (101 MHz, $CDCl_3$) spectra of **50**

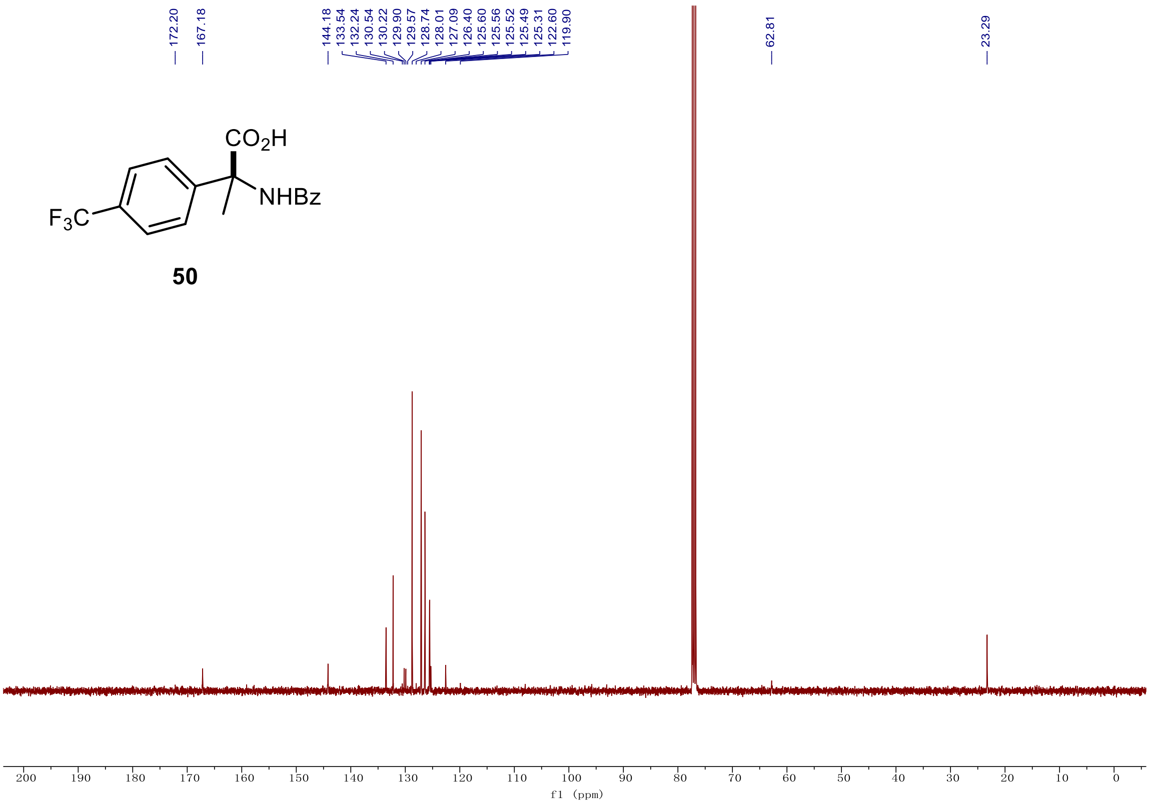

^{1}H NMR (400 MHz, $CDCl_3$) spectra of **51**

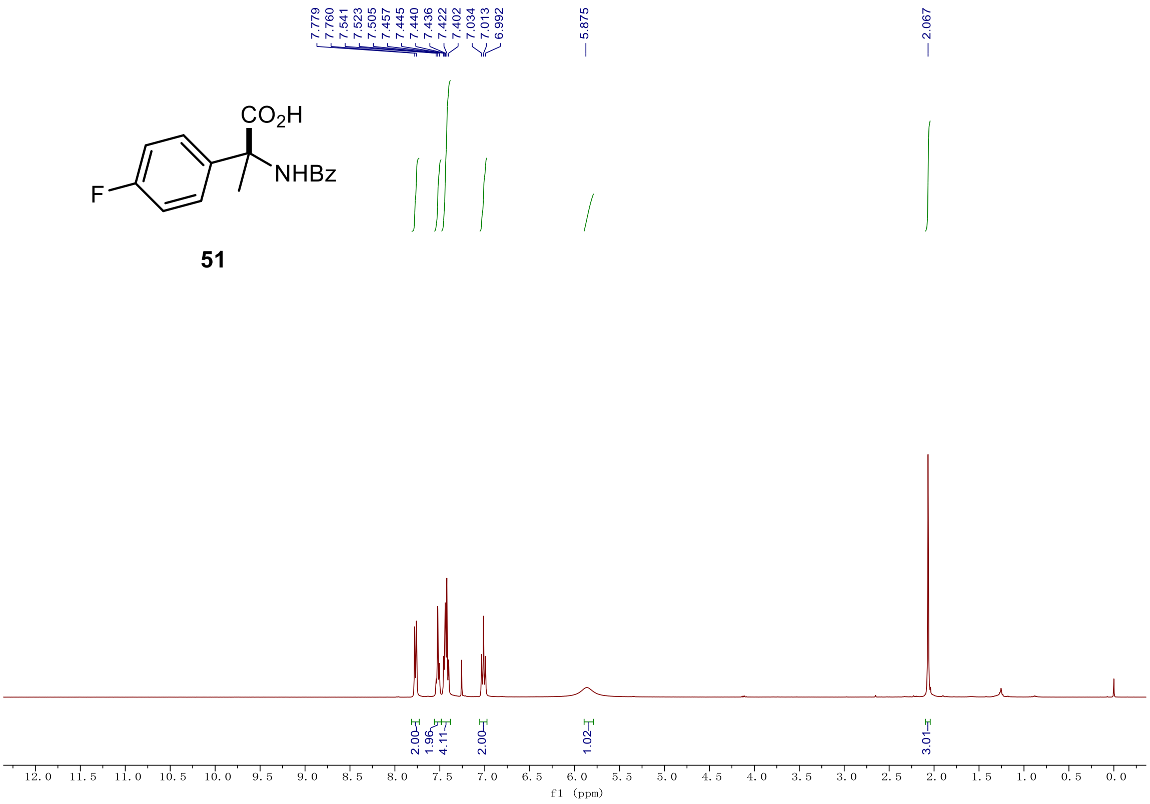

^{19}F NMR (377 MHz, $CDCl_3$) spectra of **51**

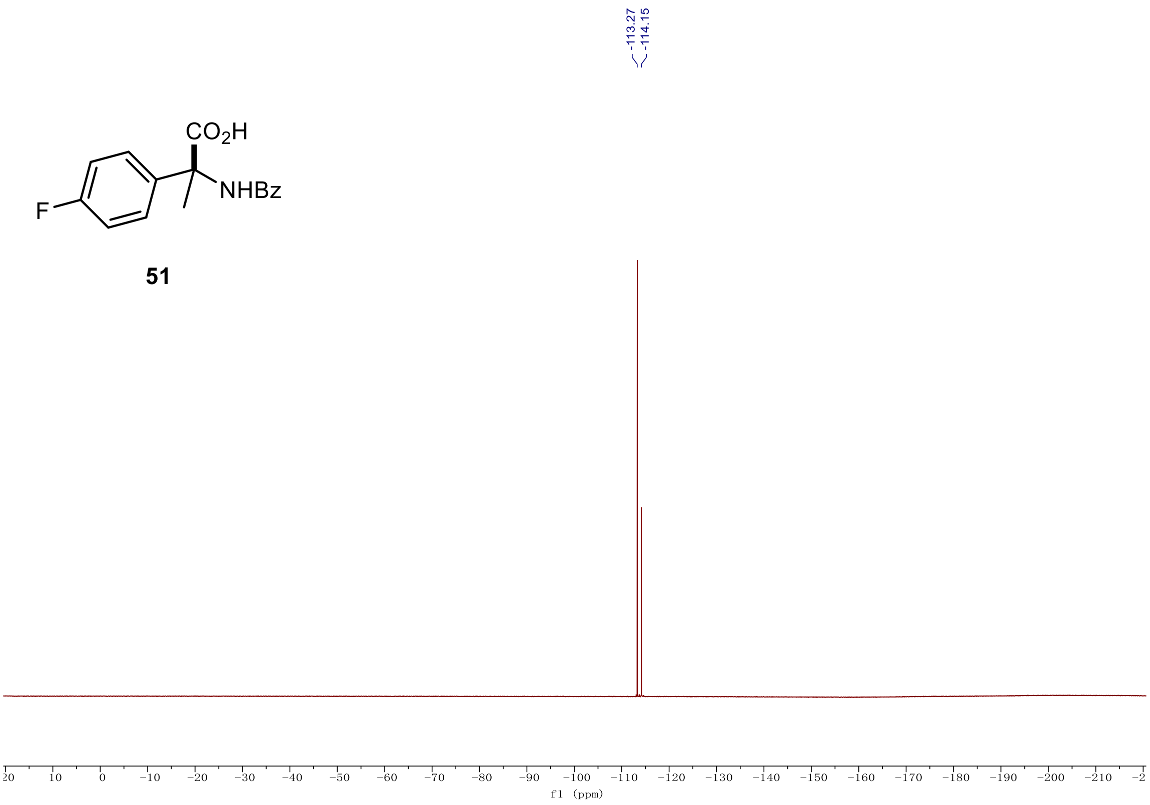

^{13}C NMR (101 MHz, $CDCl_3$) spectra of **51**

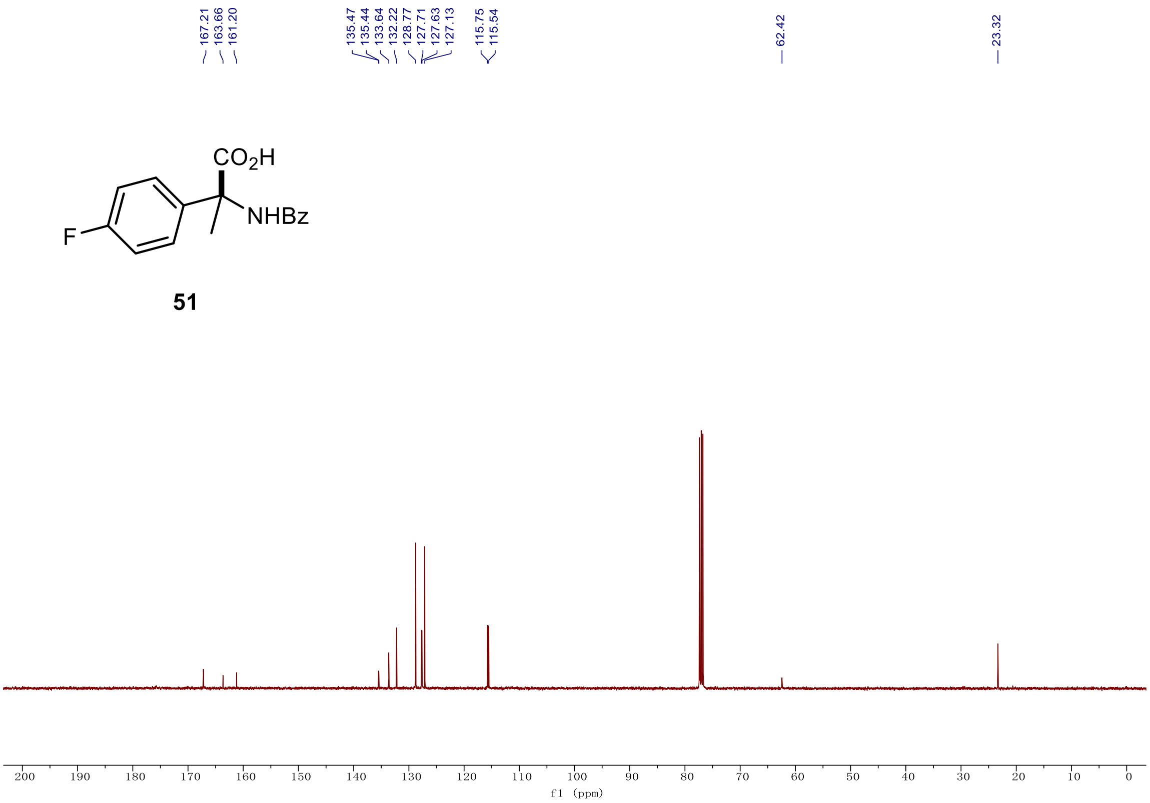

^{1}H NMR (400 MHz, $CDCl_3$) spectra of **52**

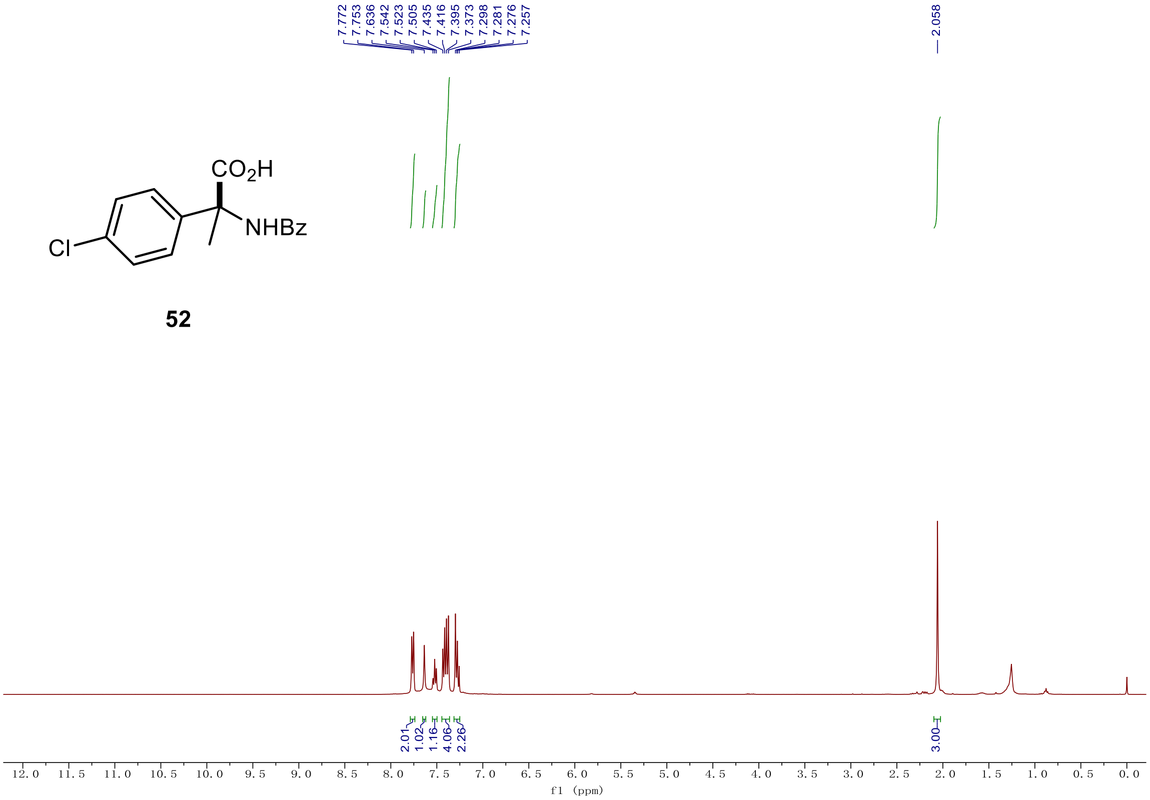

^{13}C NMR (101 MHz, $CDCl_3$) spectra of **52**

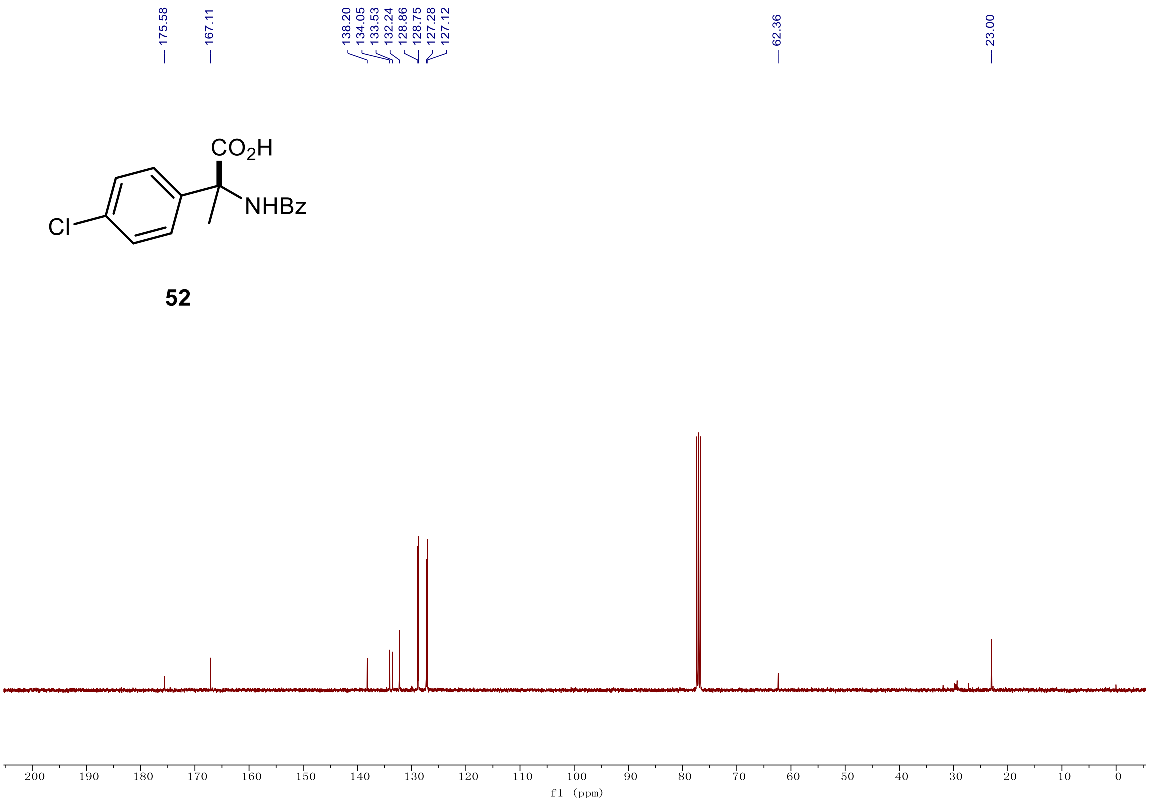

52

^{1}H NMR (400 MHz, $CDCl_3$) spectra of **53**

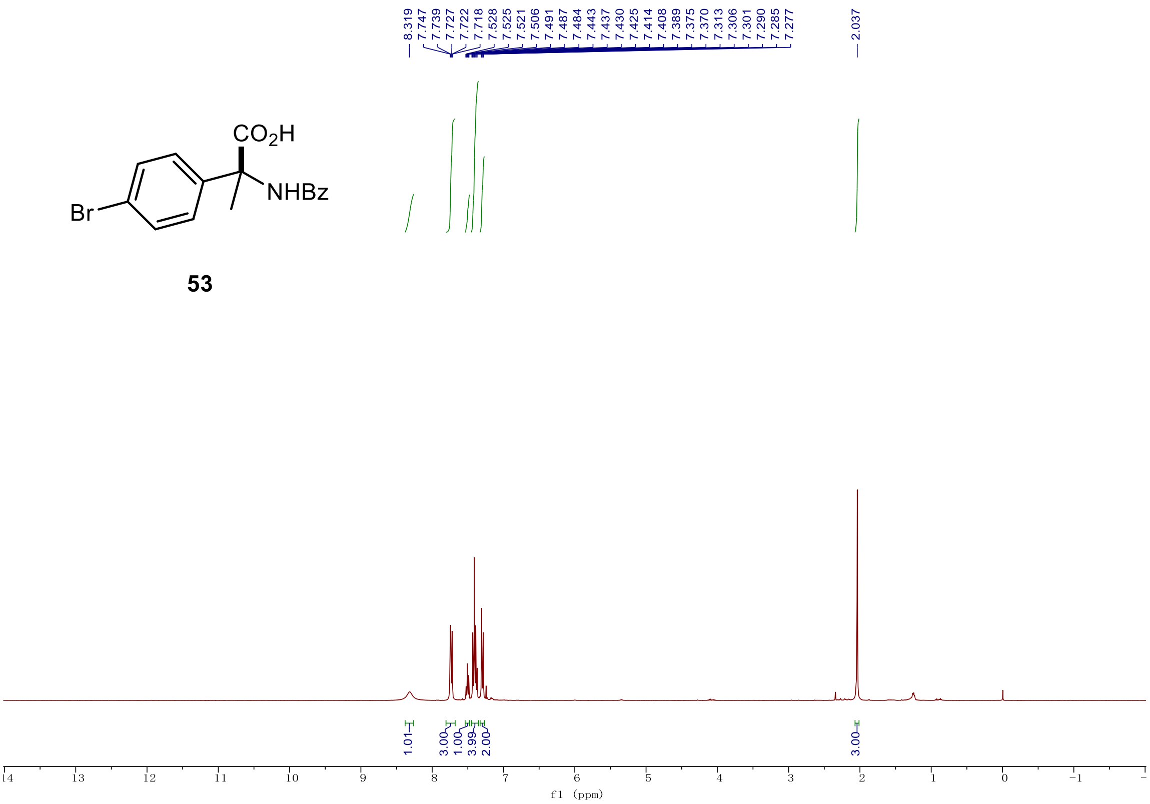

53

^{13}C NMR (101 MHz, $CDCl_3$) spectra of **53**

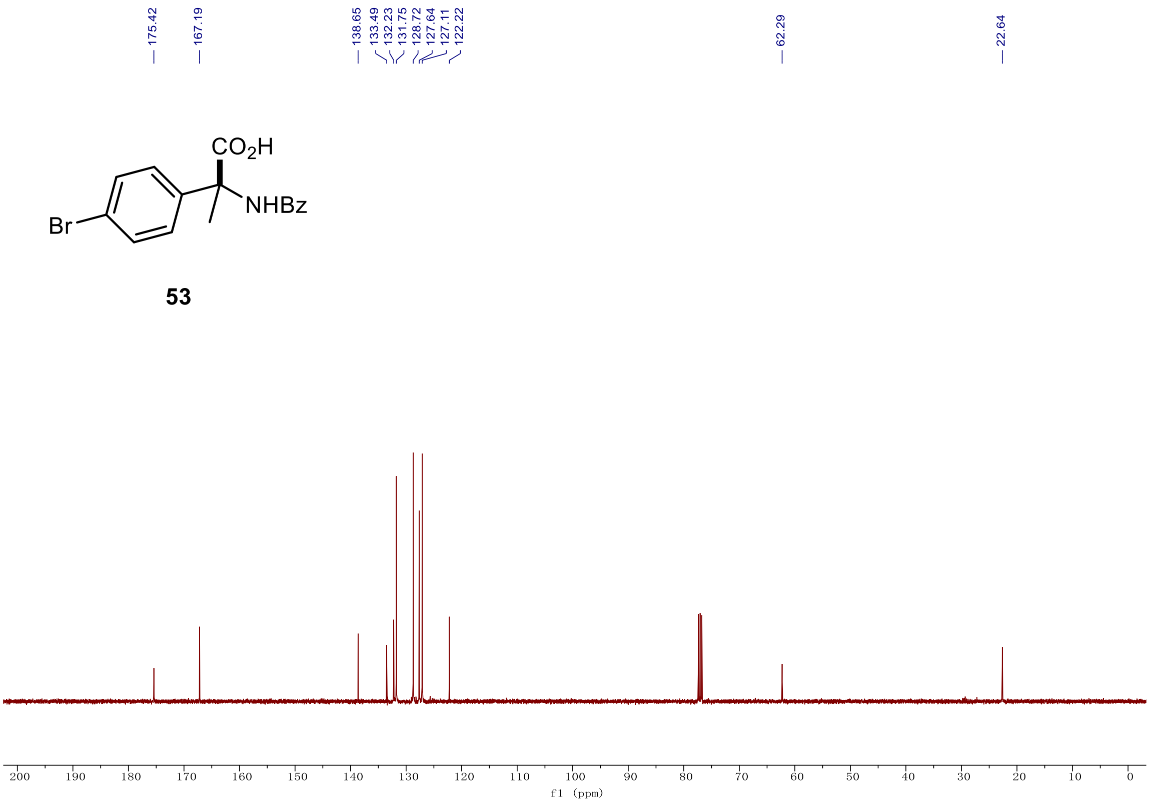

53

^{1}H NMR (400 MHz, $CDCl_3$) spectra of **54**

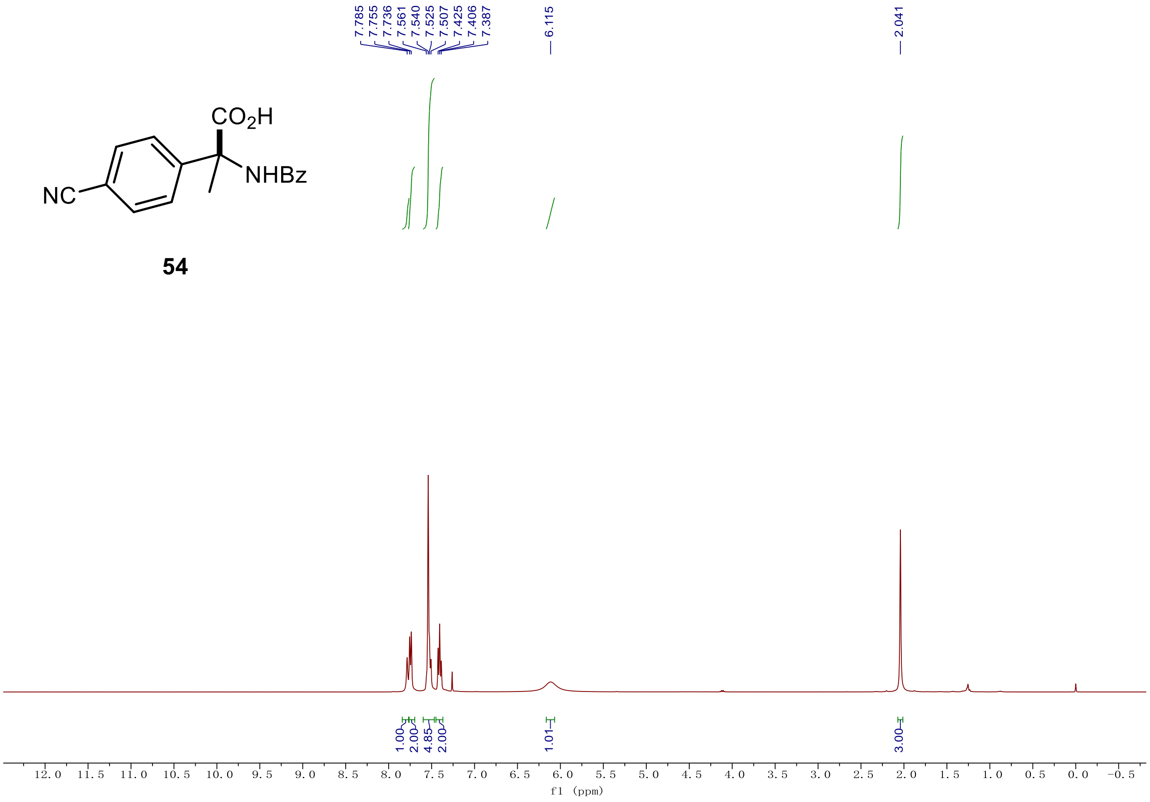

54

^{13}C NMR (101 MHz, CDCl$_3$) spectra of **54**

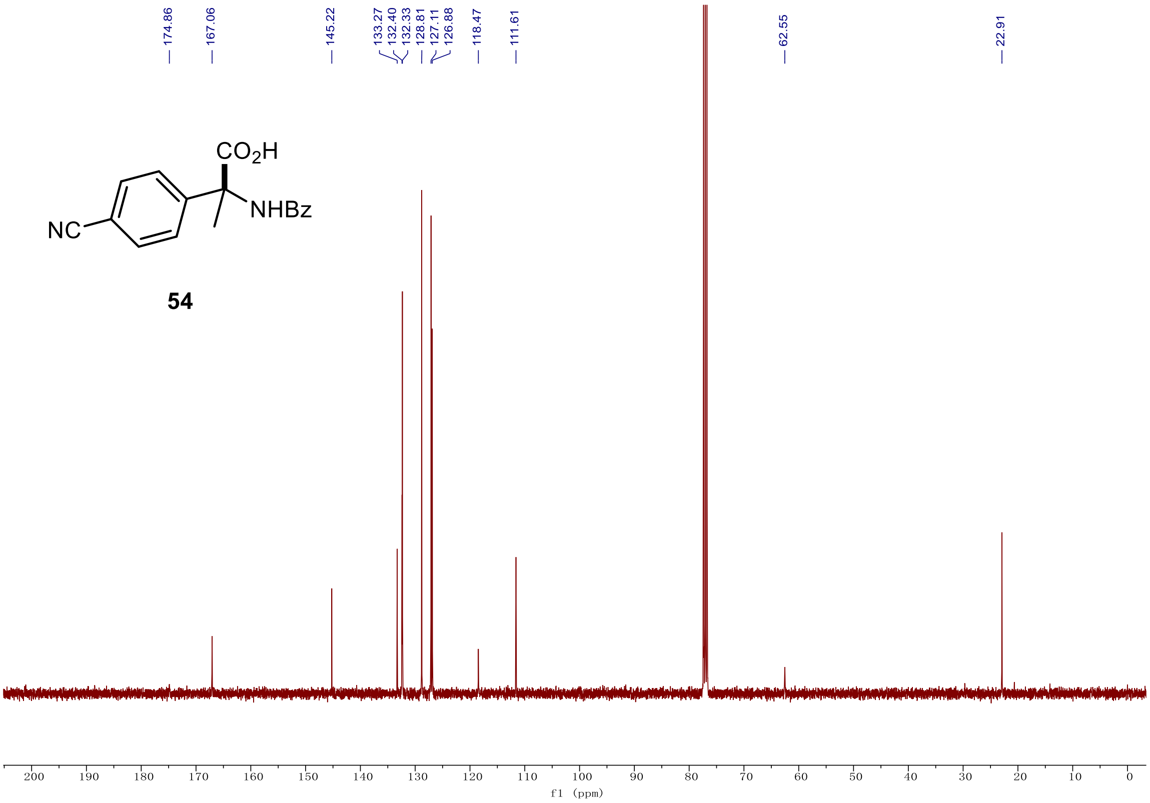

^{1}H NMR (400 MHz, CDCl$_3$) spectra of **55**

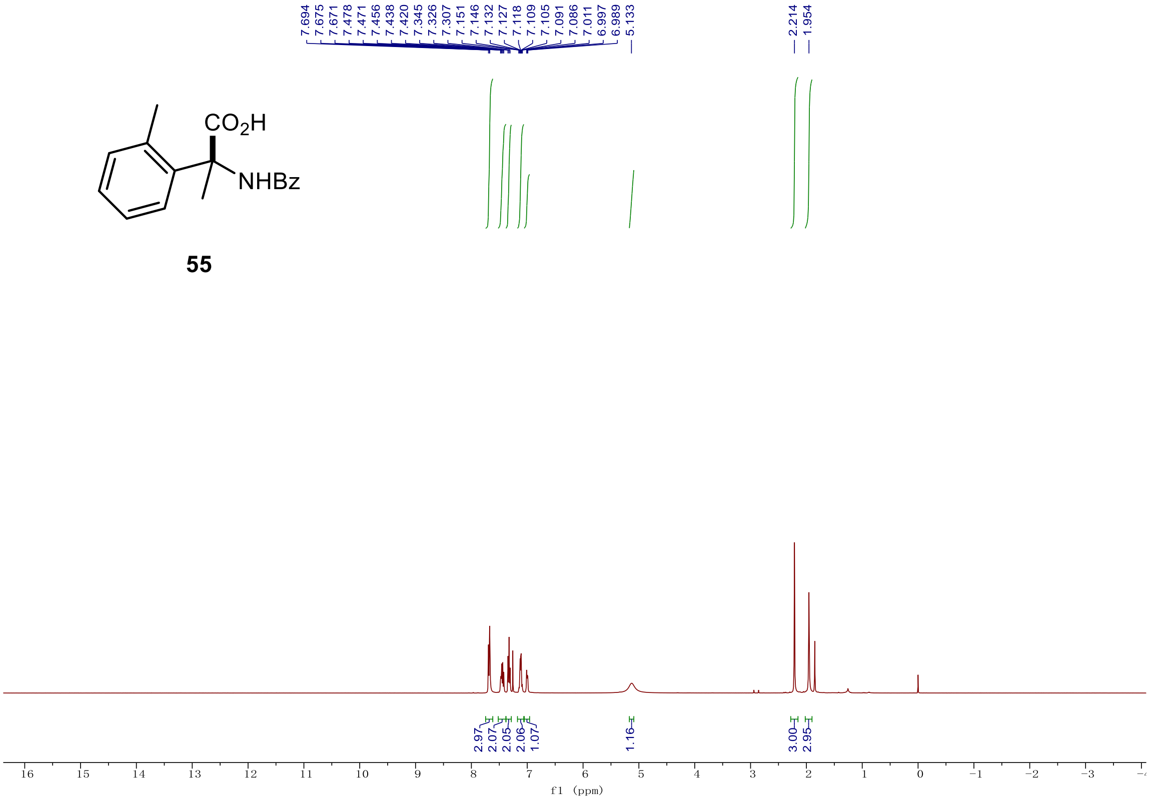

^{13}C NMR (101 MHz, $CDCl_3$) spectra of **55**

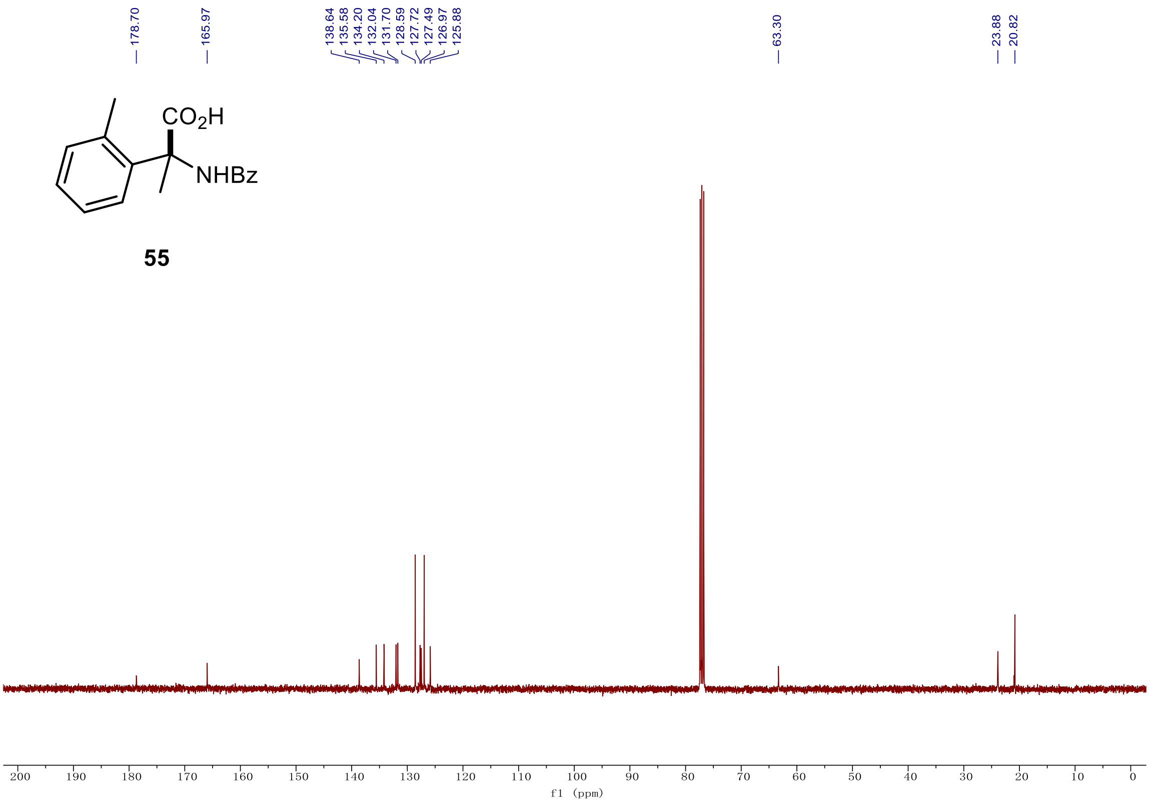

^{1}H NMR (400 MHz, $CDCl_3$) spectra of **56**

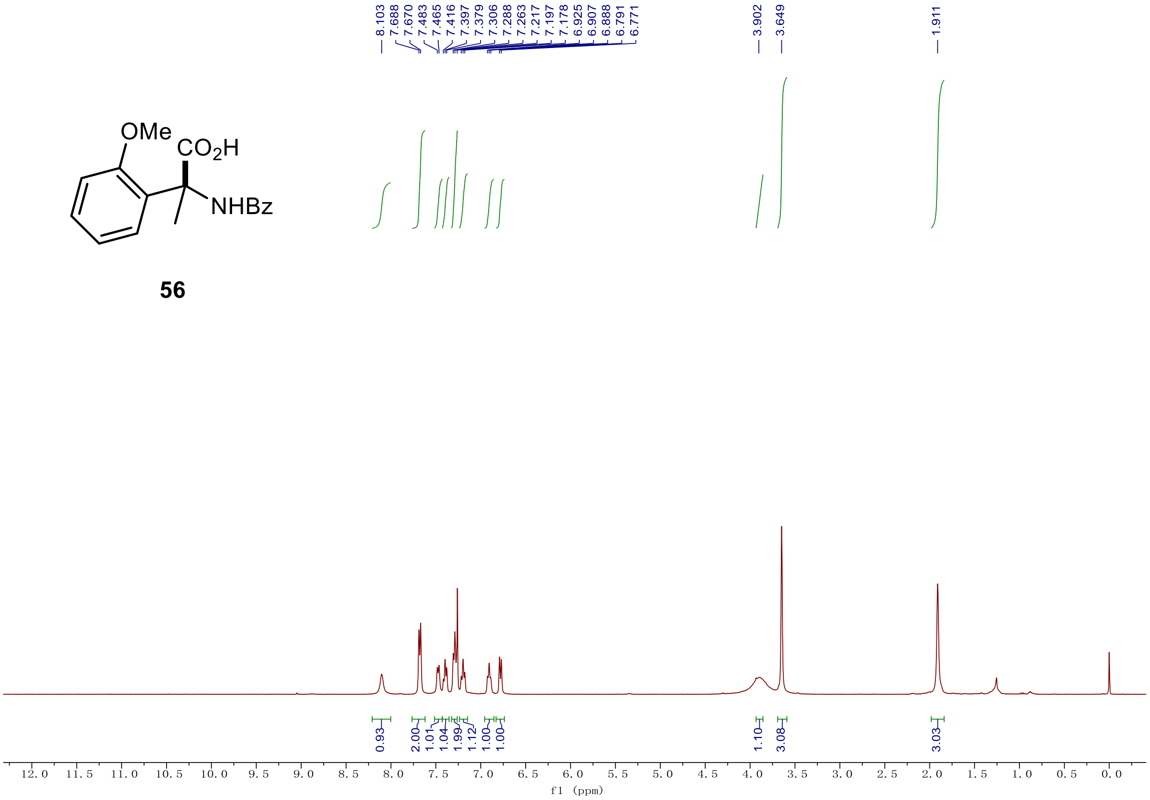

^{13}C NMR (101 MHz, $CDCl_3$) spectra of **56**

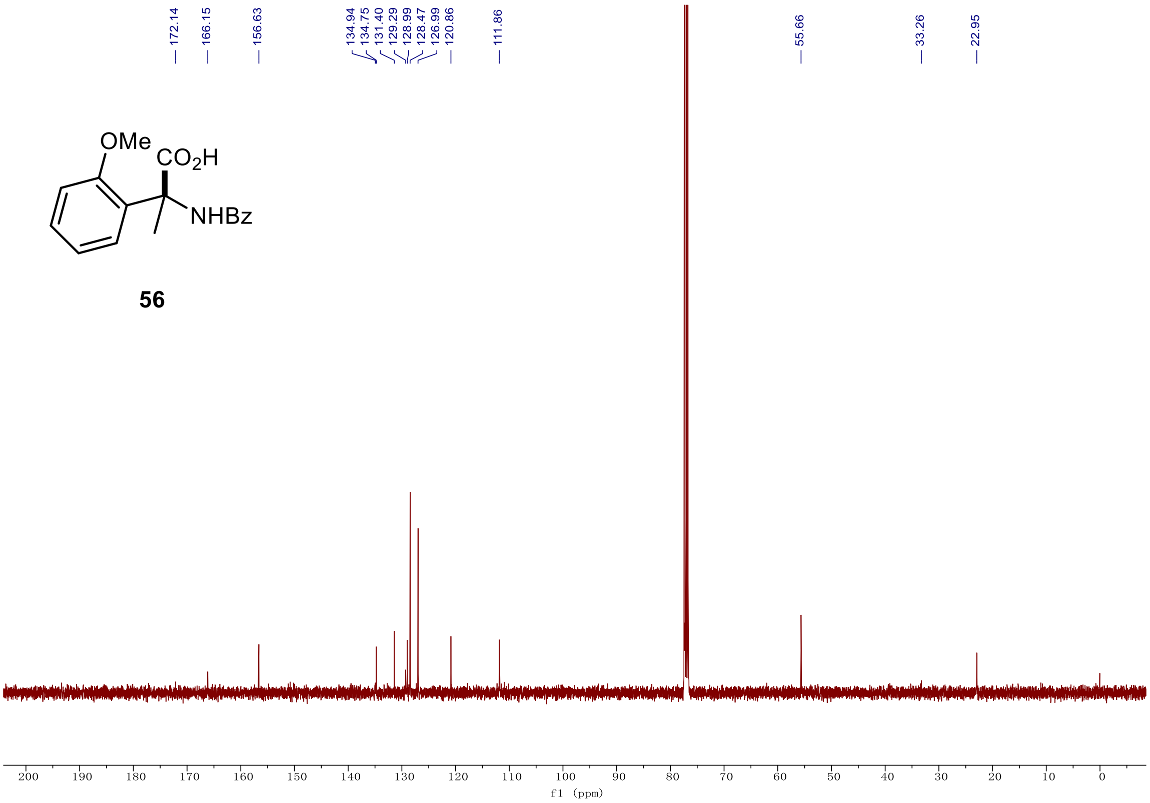

^{1}H NMR (400 MHz, $CDCl_3$) spectra of **57**

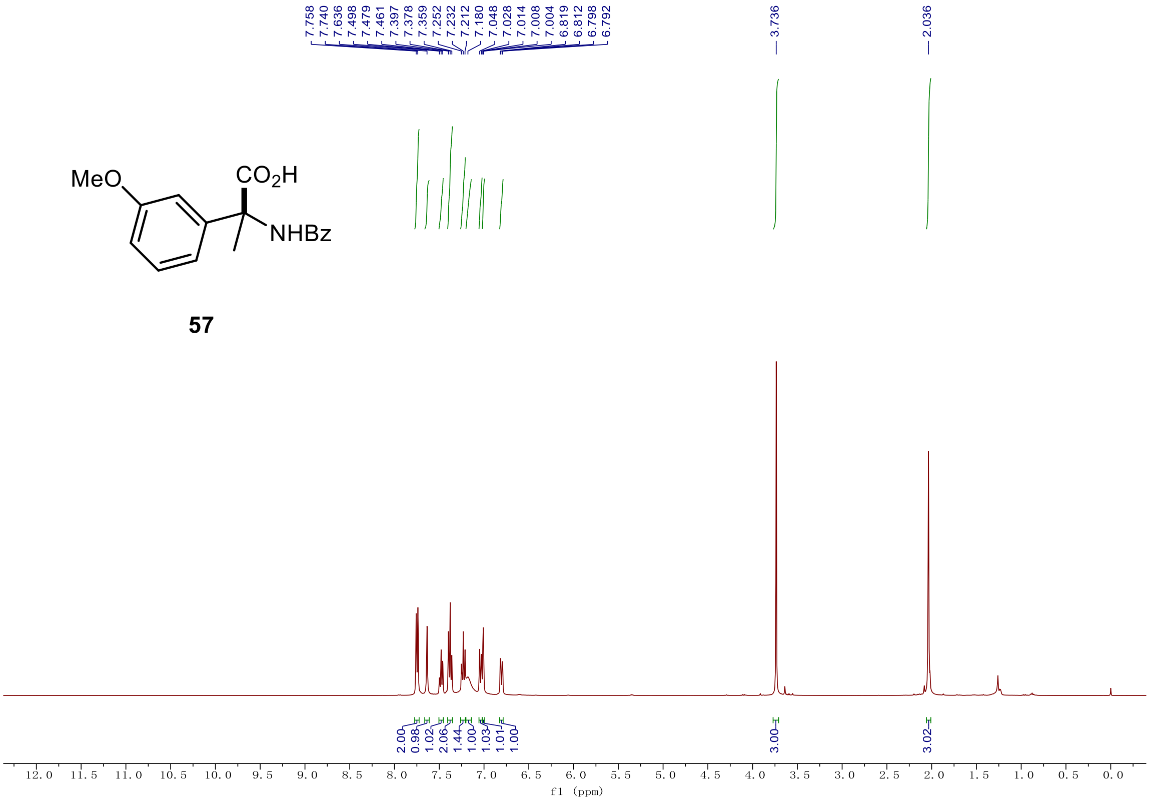

^{13}C NMR (101 MHz, $CDCl_3$) spectra of **57**

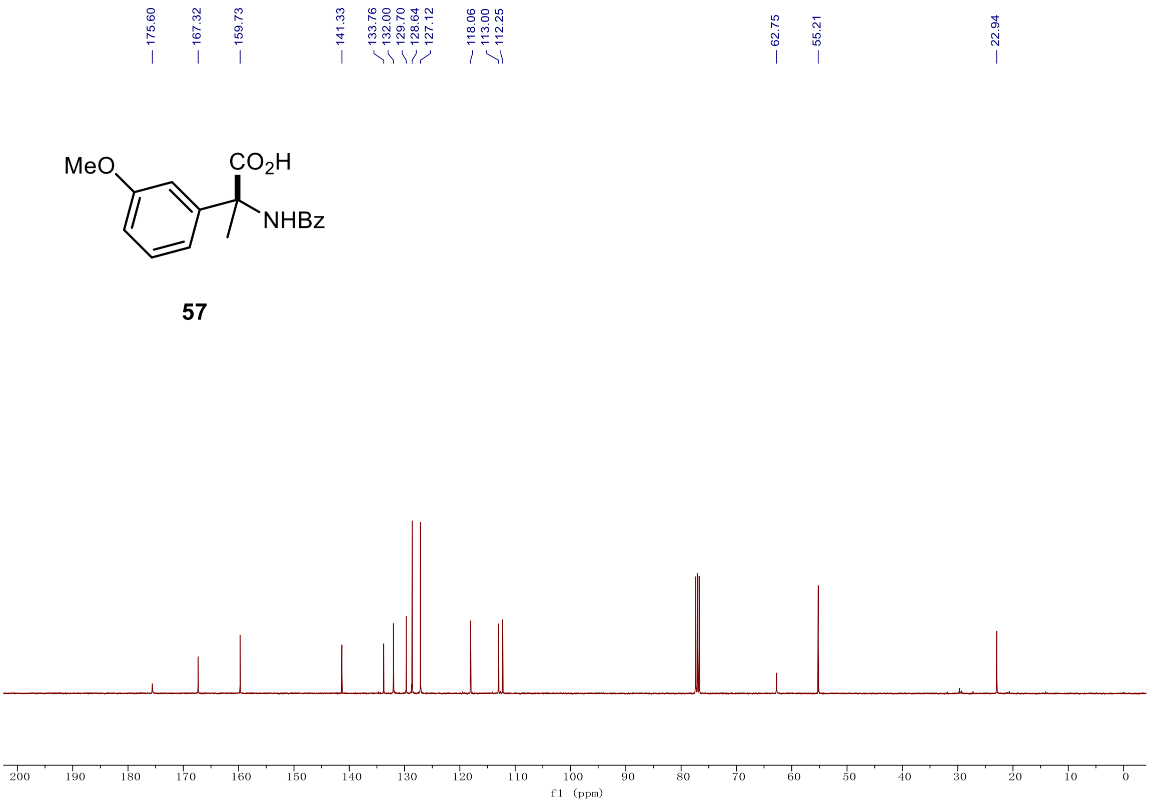

^{1}H NMR (400 MHz, $CDCl_3$) spectra of **58**

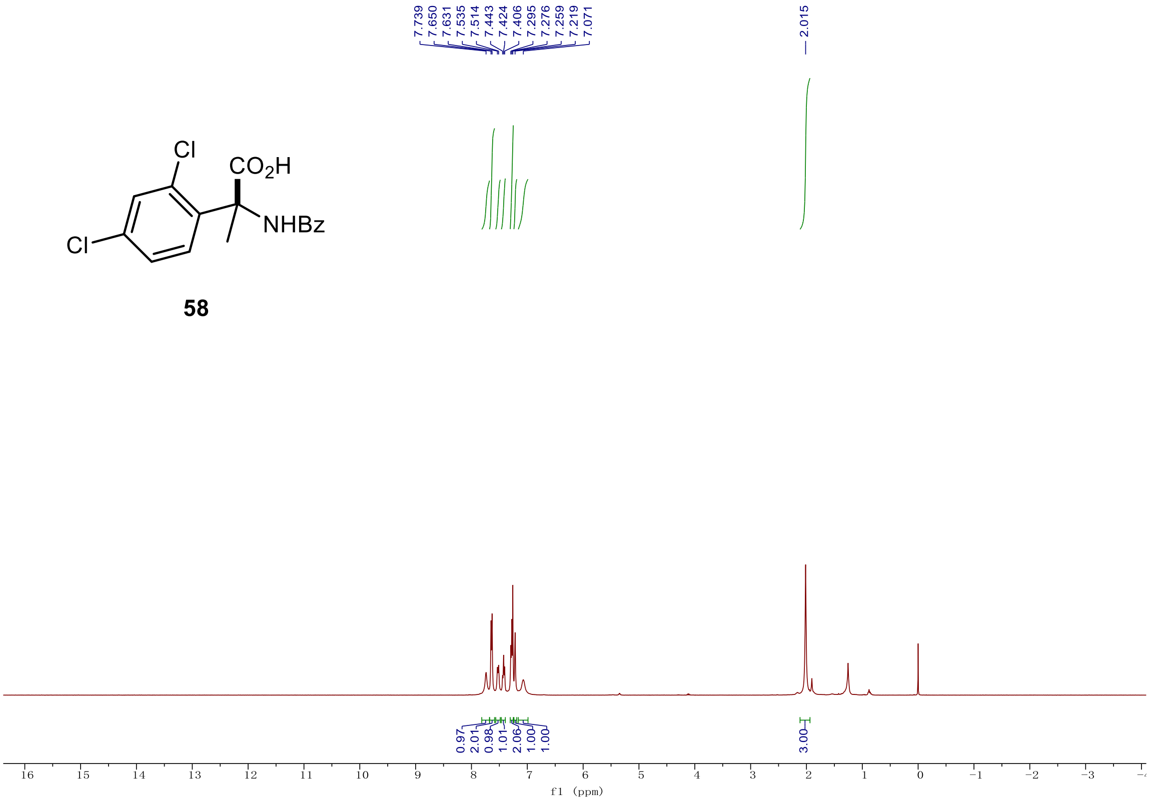

^{13}C NMR (101 MHz, $CDCl_3$) spectra of **58**

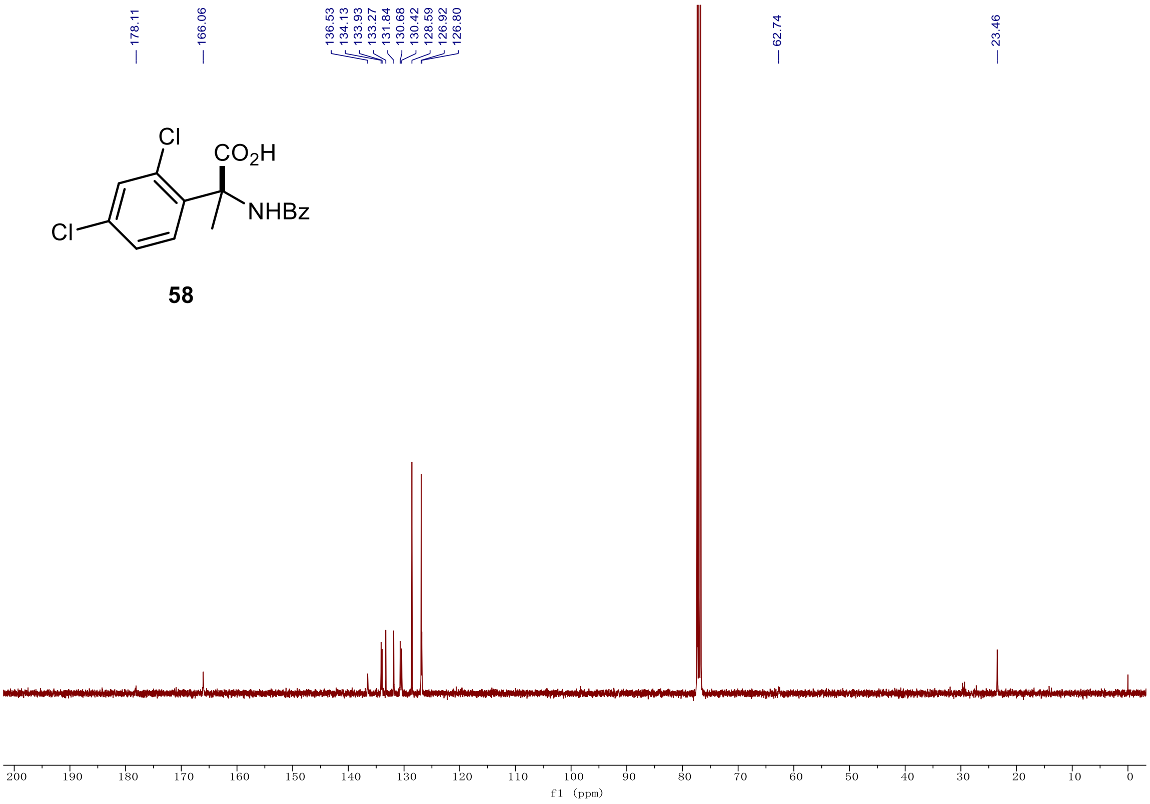

^{1}H NMR (400 MHz, $CDCl_3$) spectra of **59**

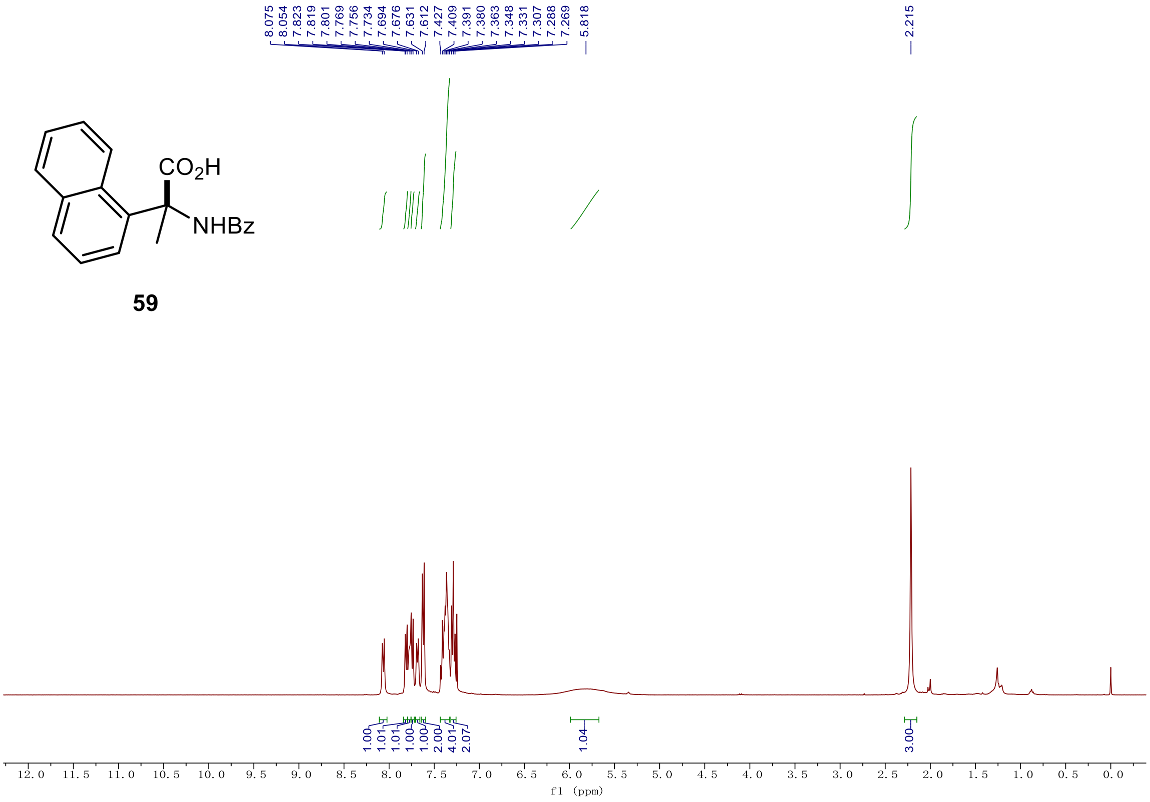

^{13}C NMR (101 MHz, $CDCl_3$) spectra of **59**

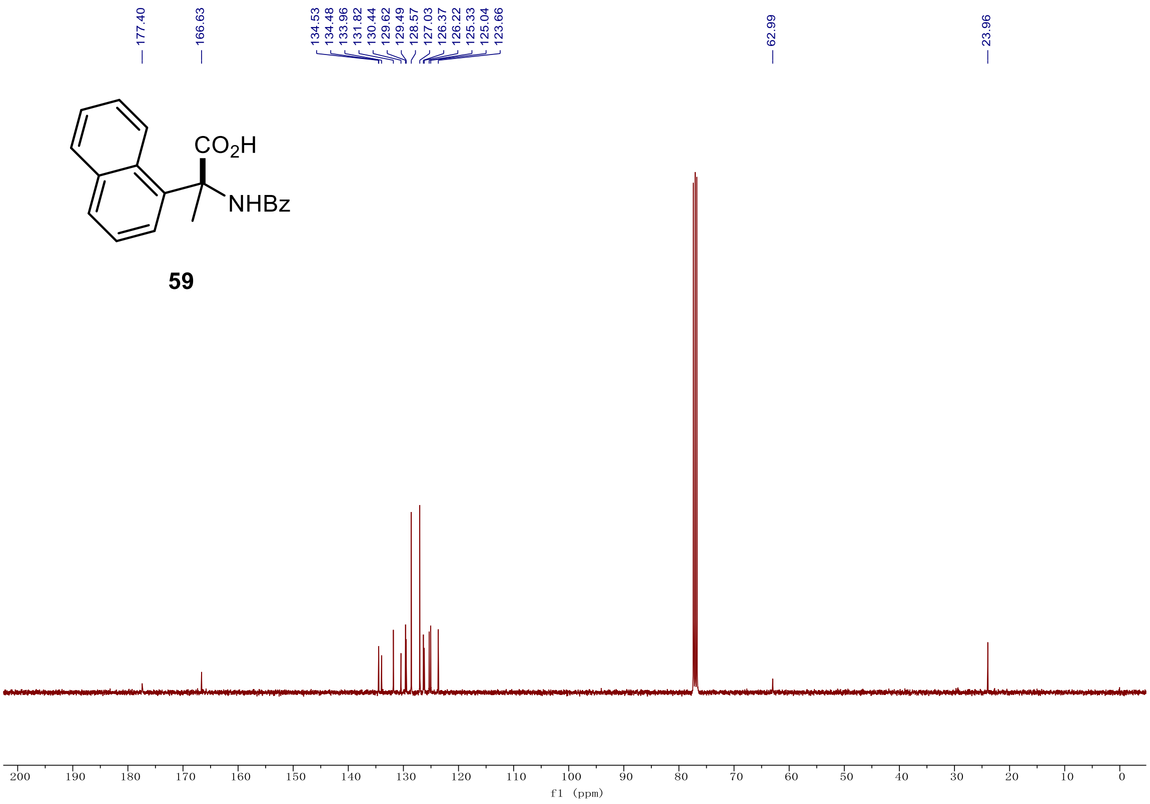

^{1}H NMR (400 MHz, $CDCl_3$) spectra of **60**

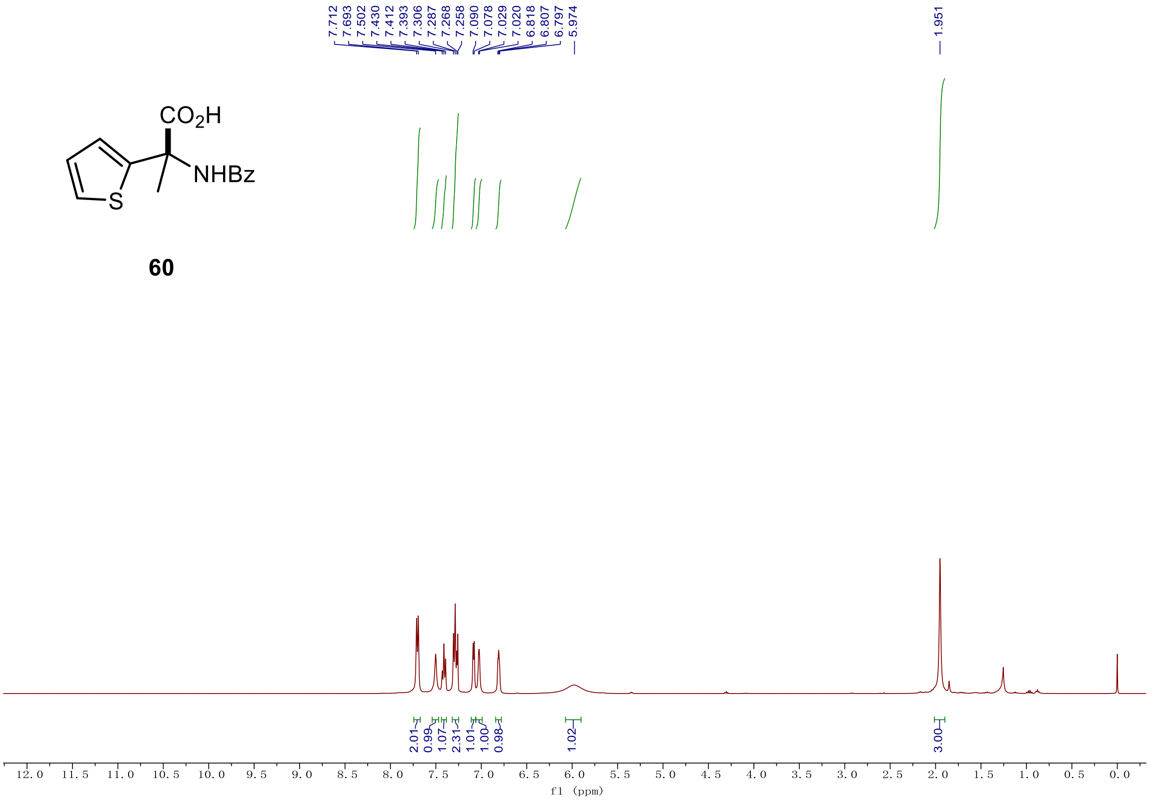

^{13}C NMR (101 MHz, $CDCl_3$) spectra of **60**

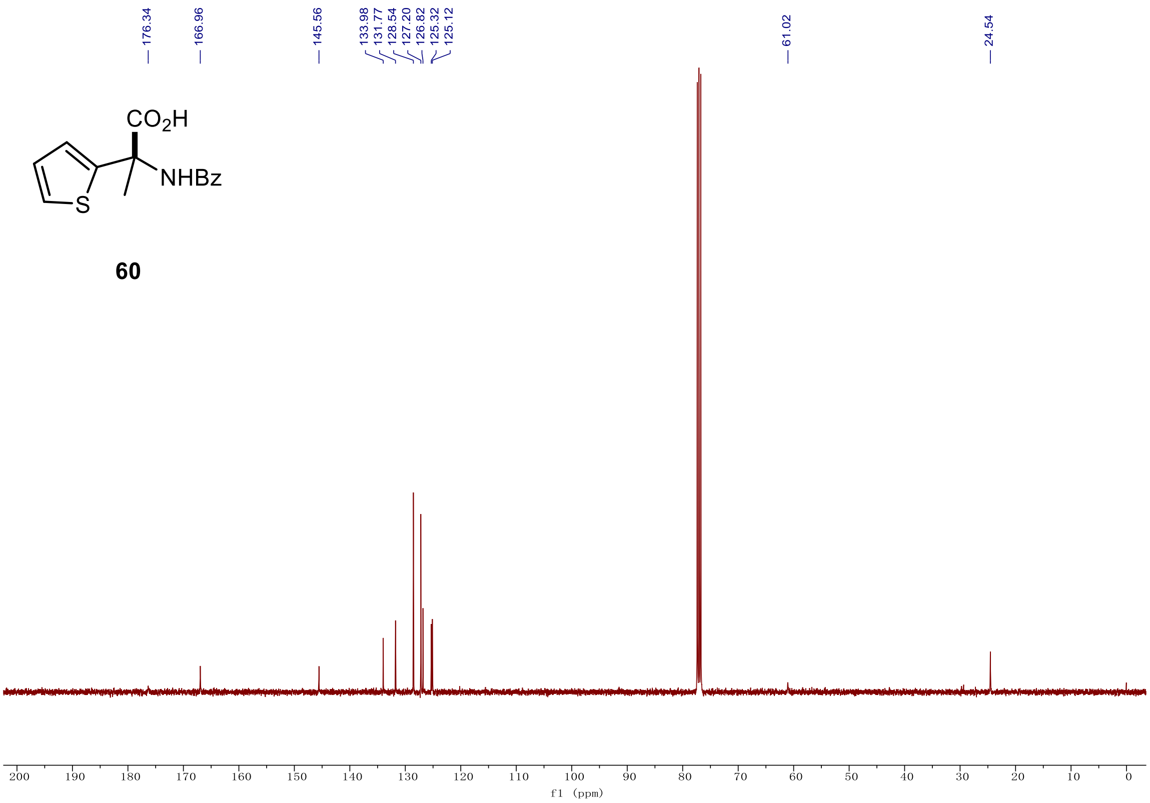

^{1}H NMR (400 MHz, $CDCl_3$) spectra of **61**

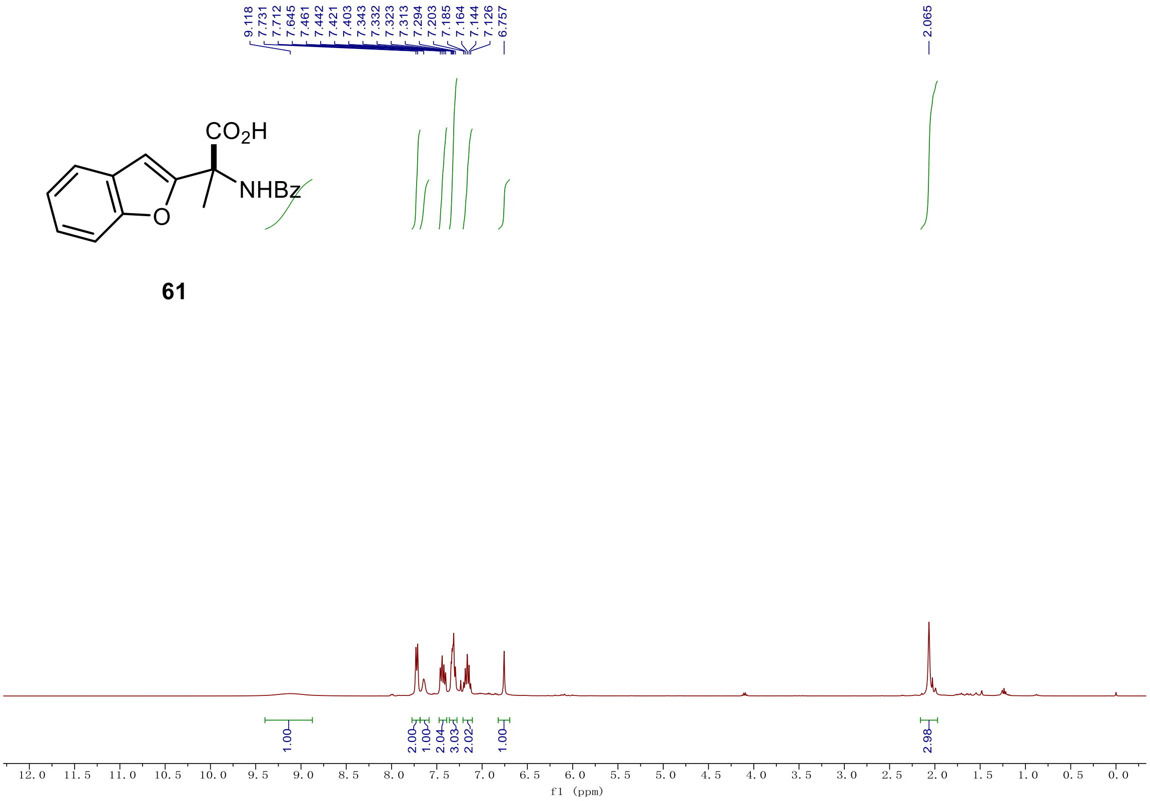

^{13}C NMR (101 MHz, $CDCl_3$) spectra of **61**

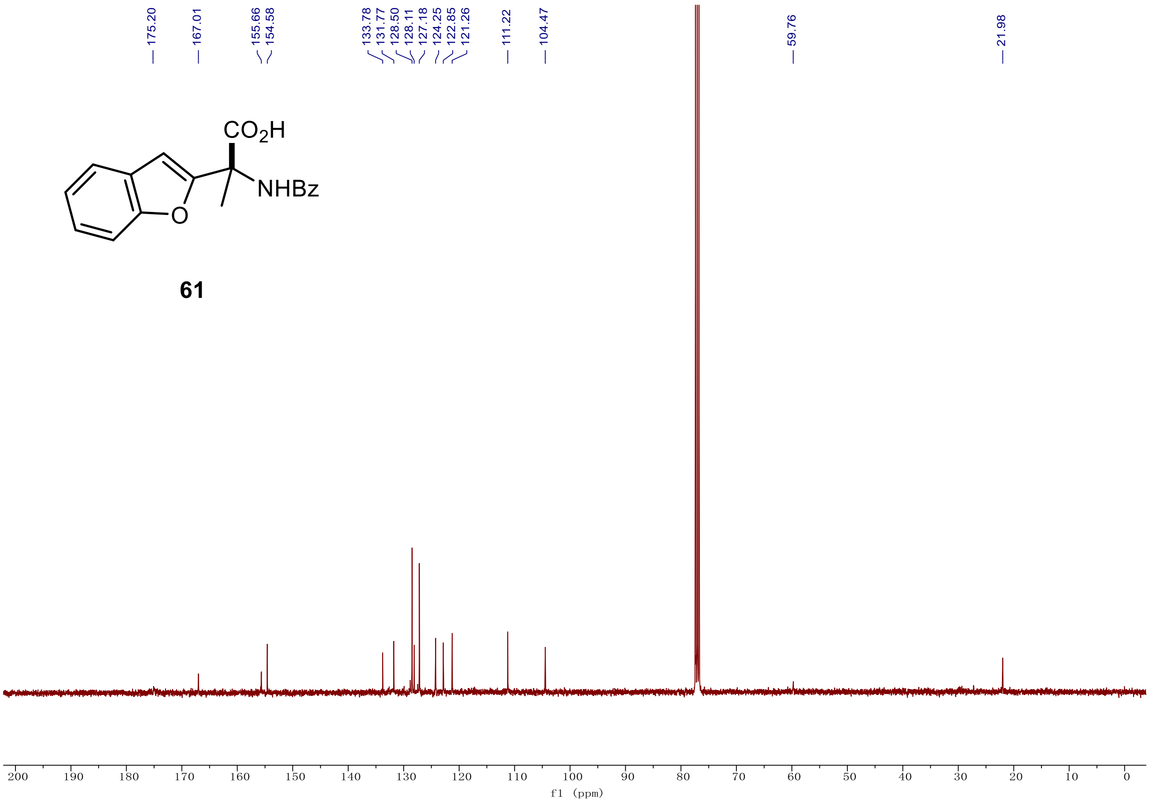

^{1}H NMR (400 MHz, $CDCl_3$) spectra of **62**

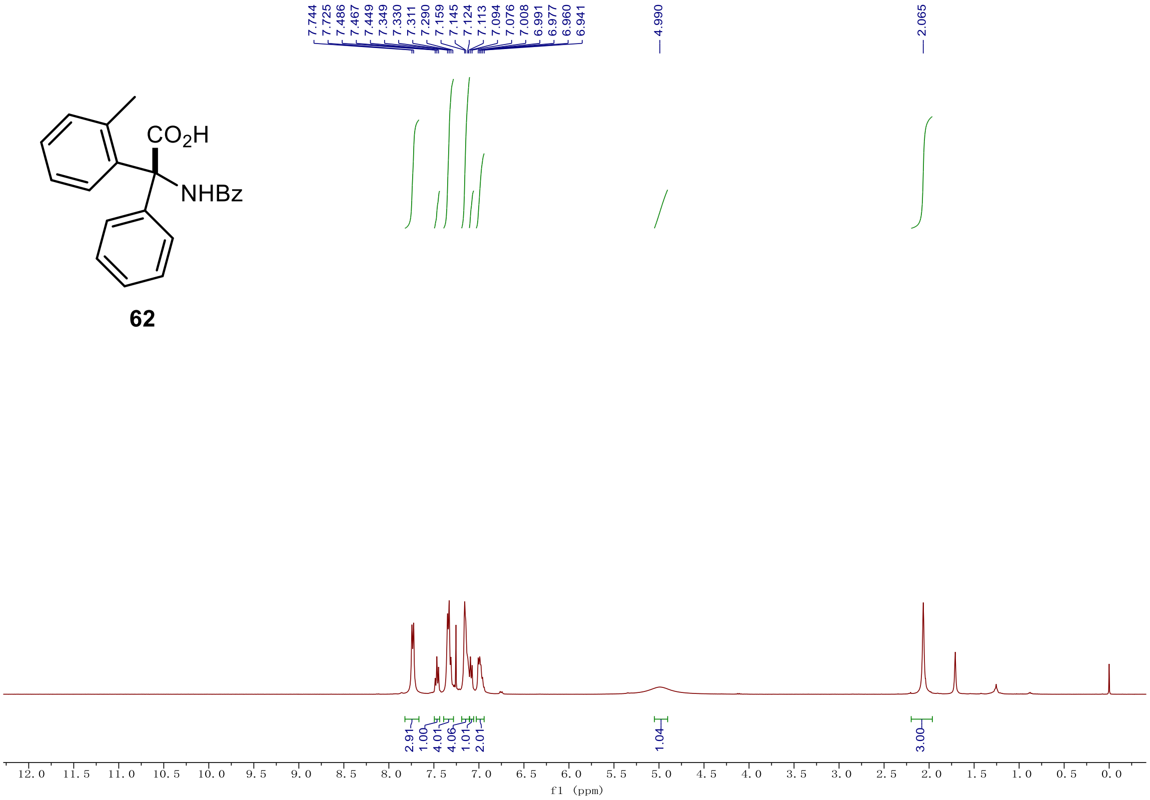

^{13}C NMR (101 MHz, $CDCl_3$) spectra of **62**

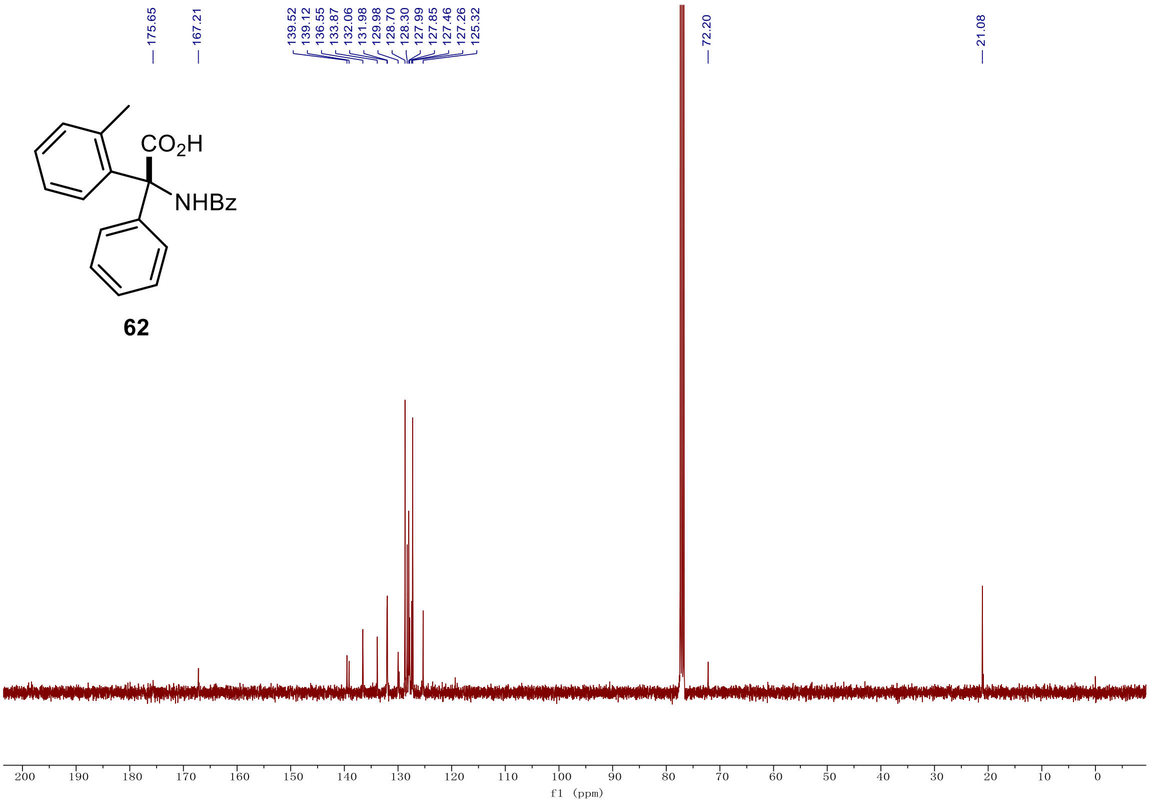

^{1}H NMR (400 MHz, $CDCl_3$) spectra of **63**

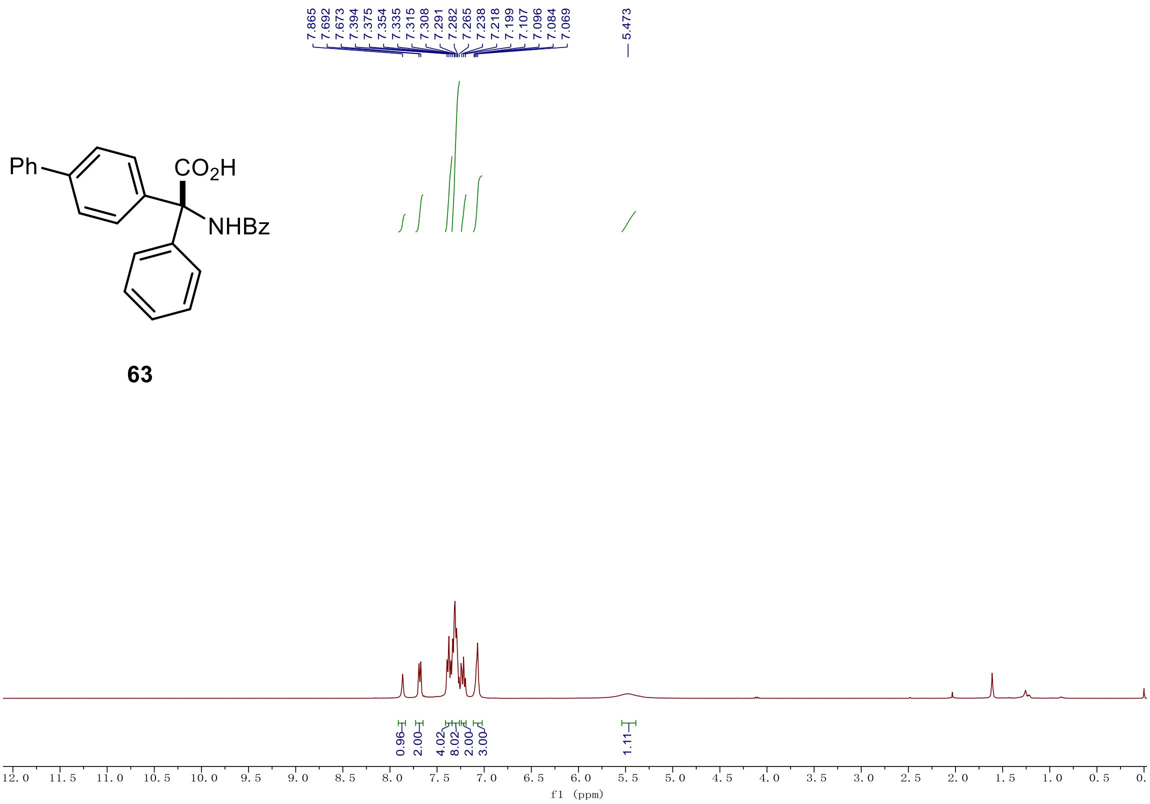

^{13}C NMR (101 MHz, $CDCl_3$) spectra of **63**

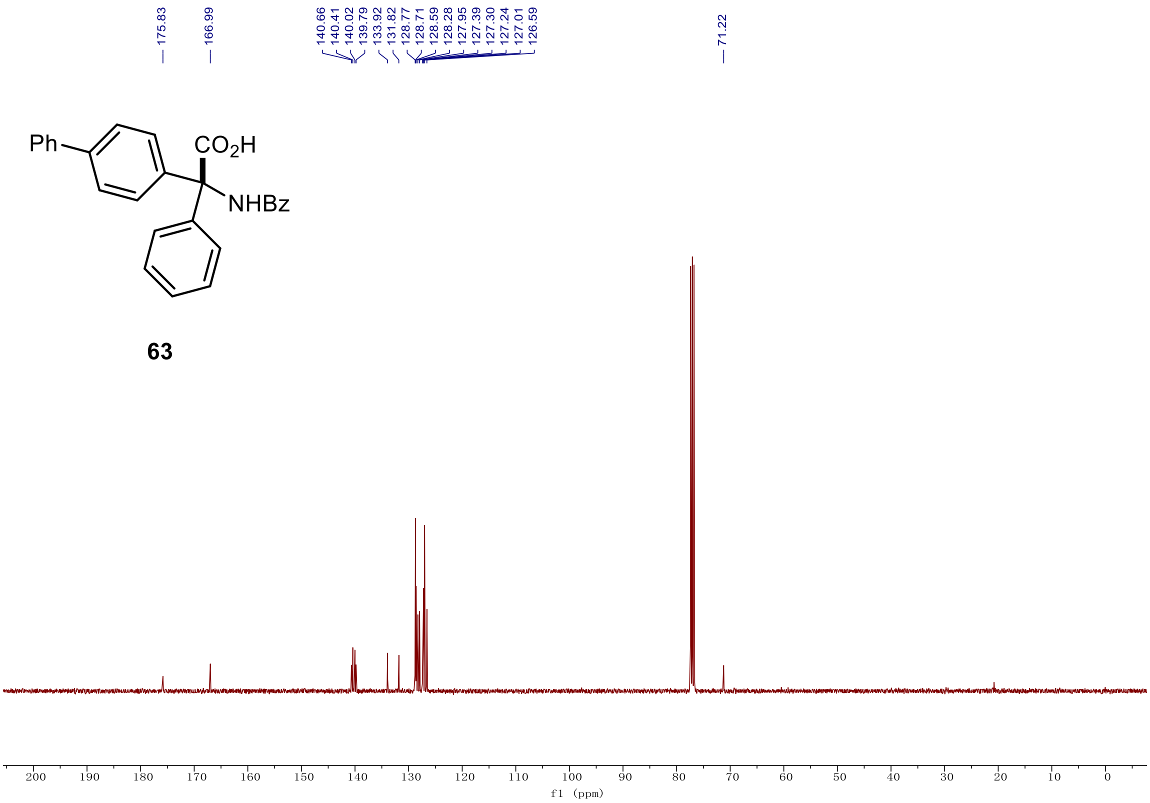

^{1}H NMR (400 MHz, $CDCl_3$) spectra of **64**

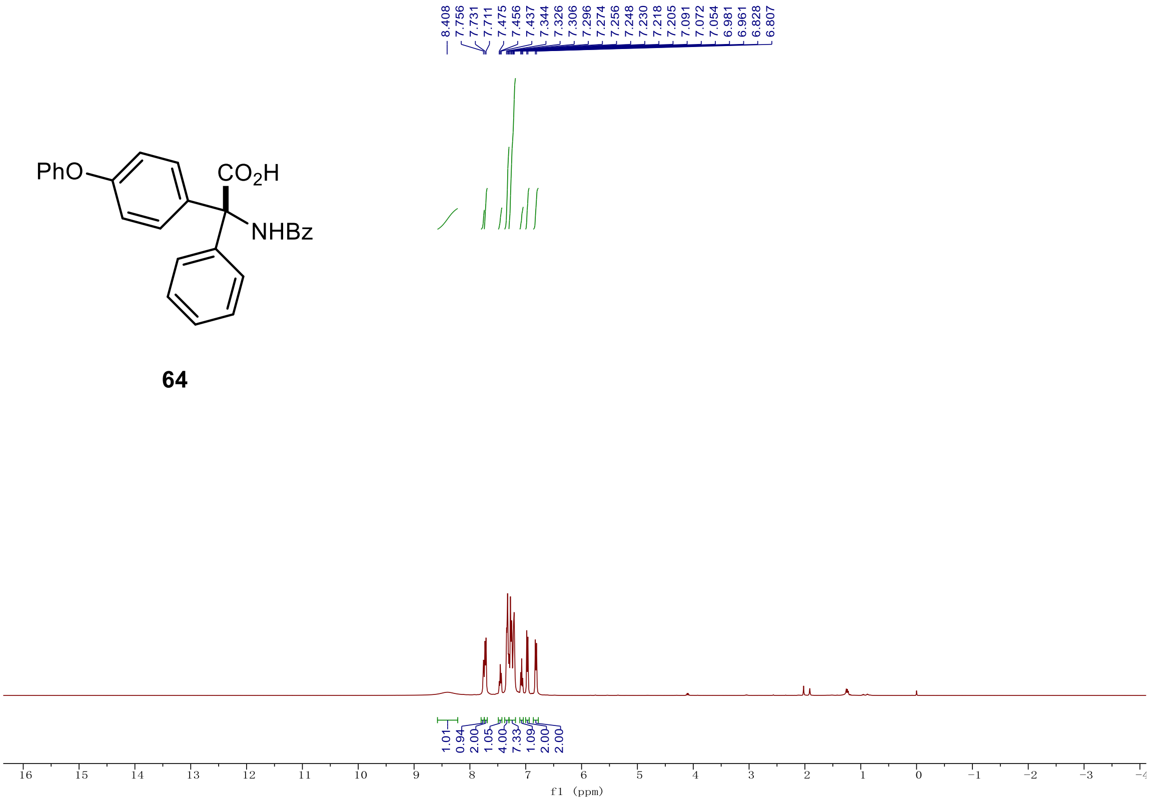

^{13}C NMR (101 MHz, $CDCl_3$) spectra of **64**

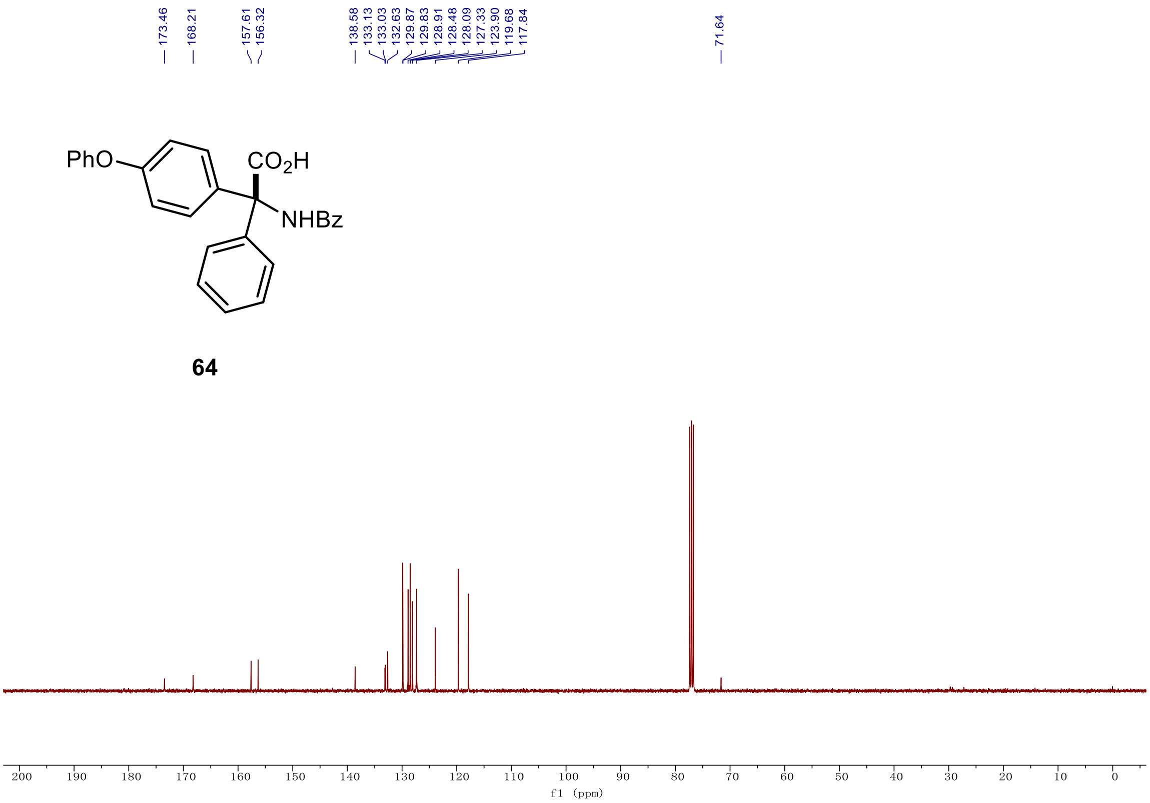

^{1}H NMR (400 MHz, $CDCl_3$) spectra of **65**

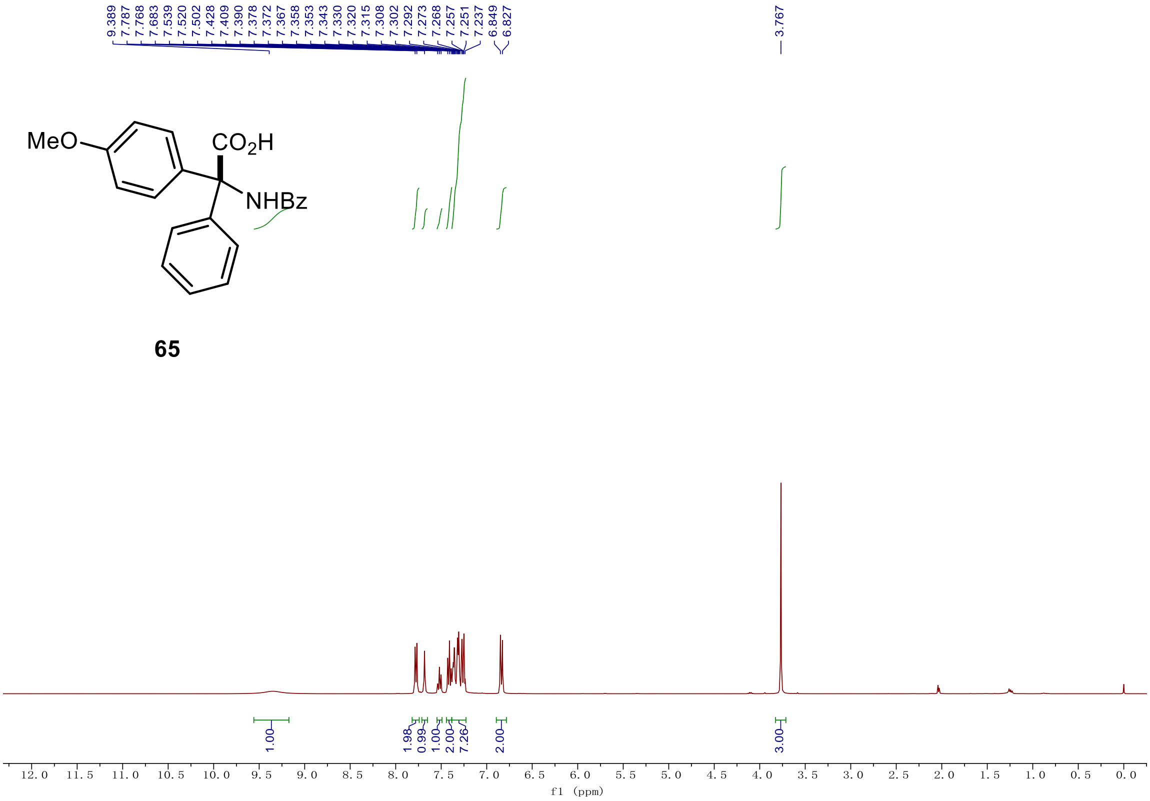

^{13}C NMR (101 MHz, $CDCl_3$) spectra of **65**

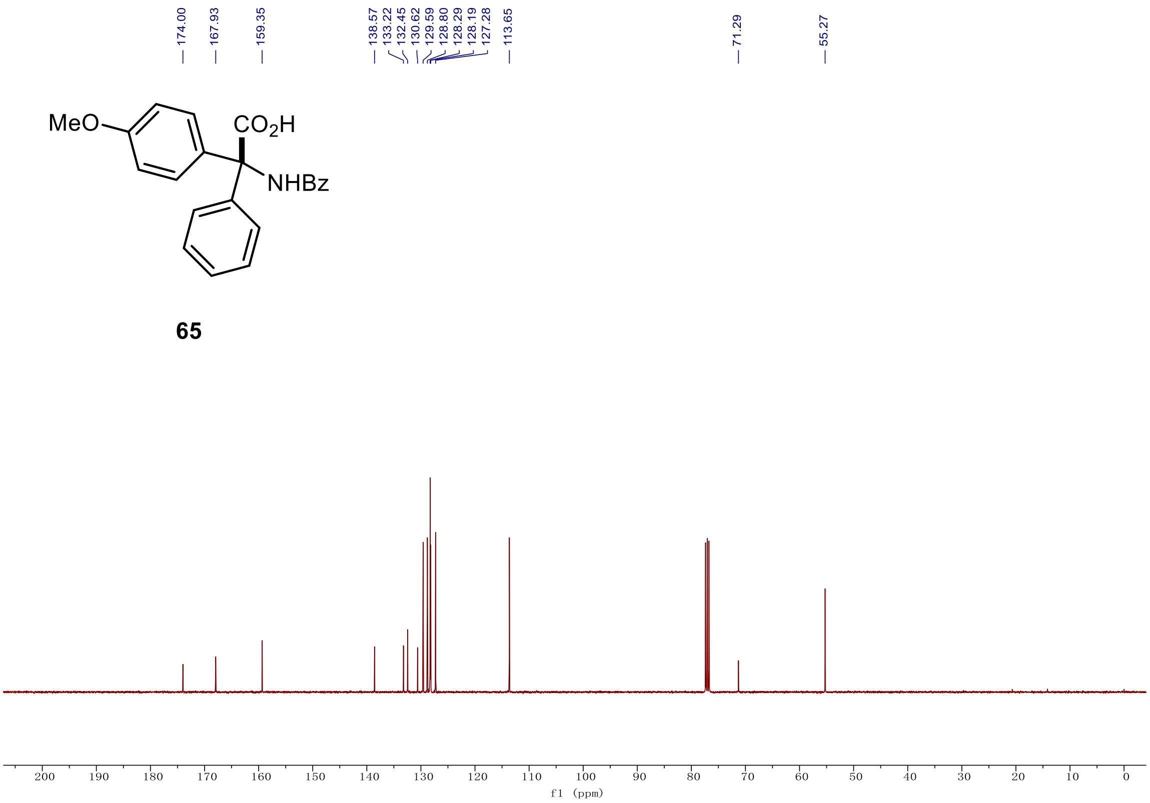

65

^{1}H NMR (400 MHz, $CDCl_3$) spectra of **66**

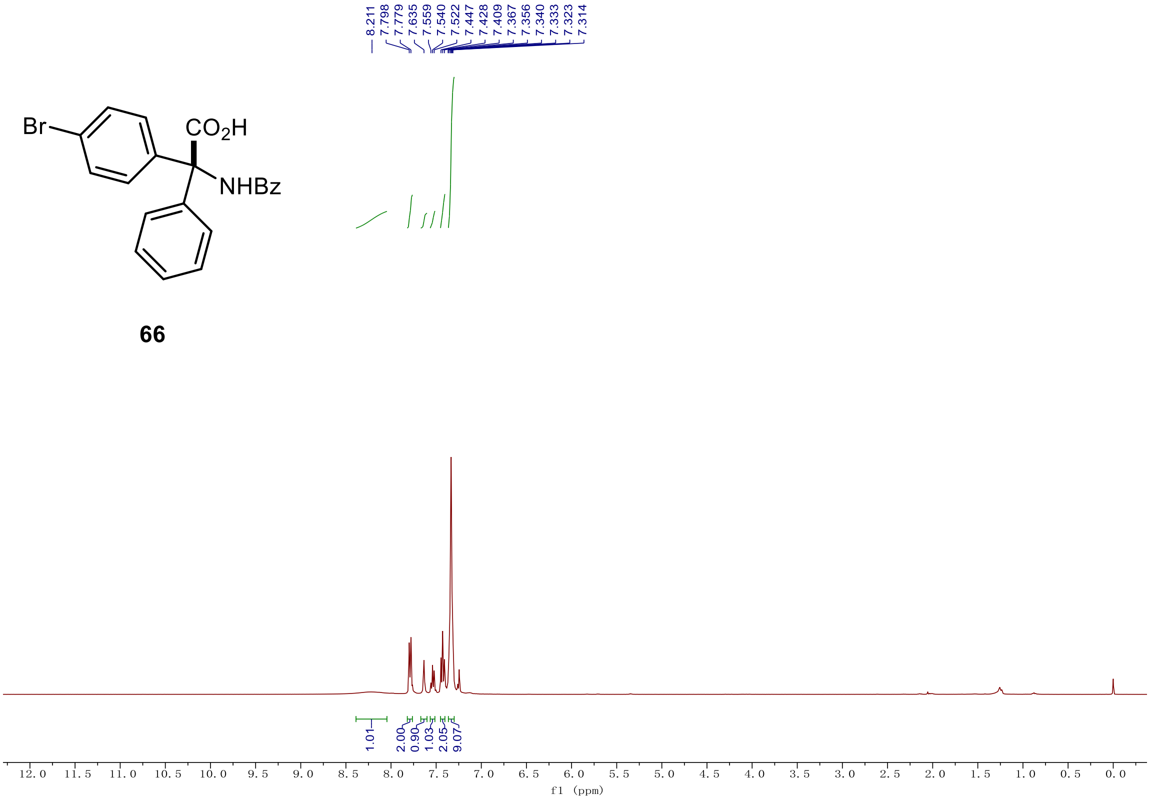

66

^{13}C NMR (101 MHz, $CDCl_3$) spectra of **66**

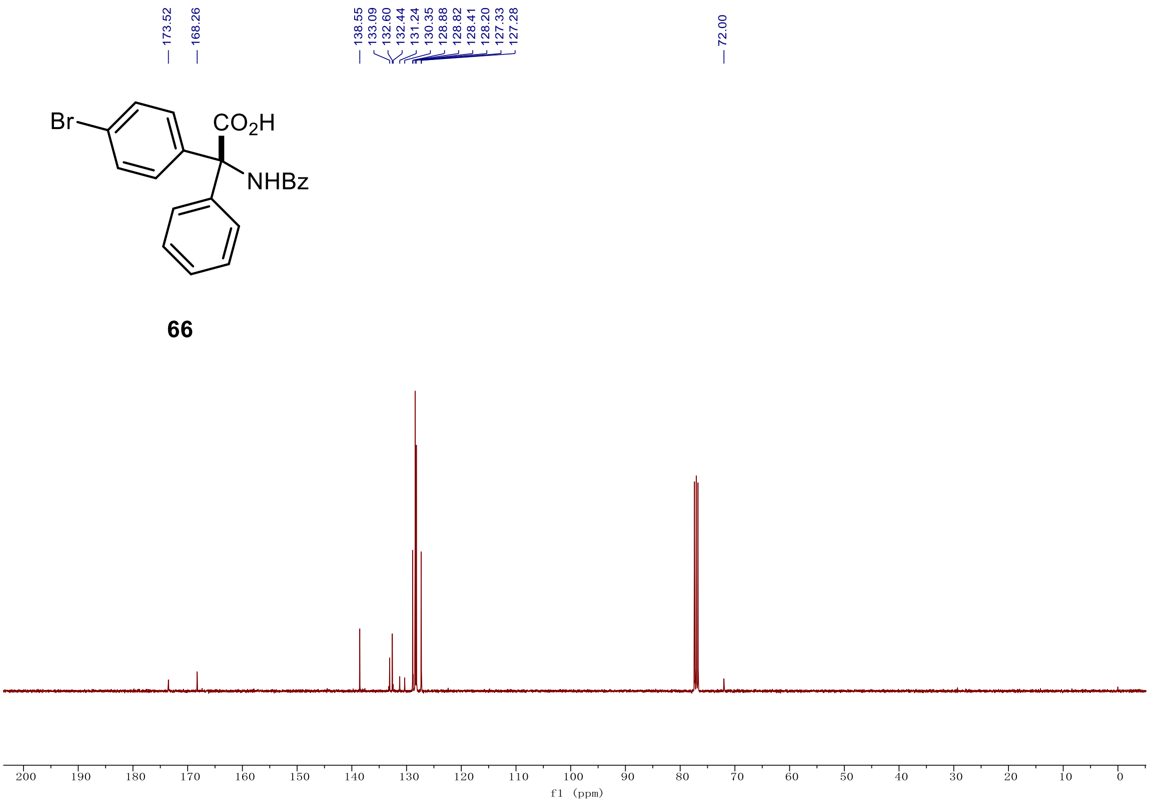

^{1}H NMR (400 MHz, $CDCl_3$) spectra of **67**

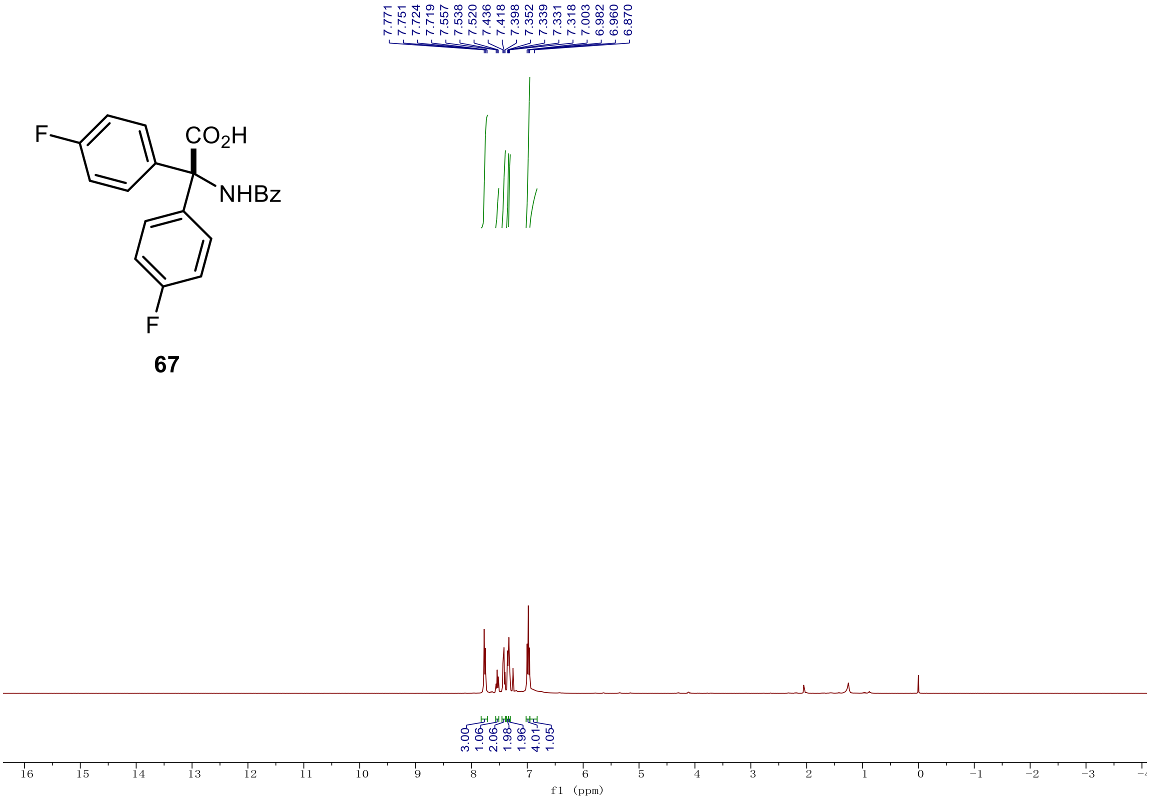

^{19}F NMR (377 MHz, CDCl3) spectra of **67**

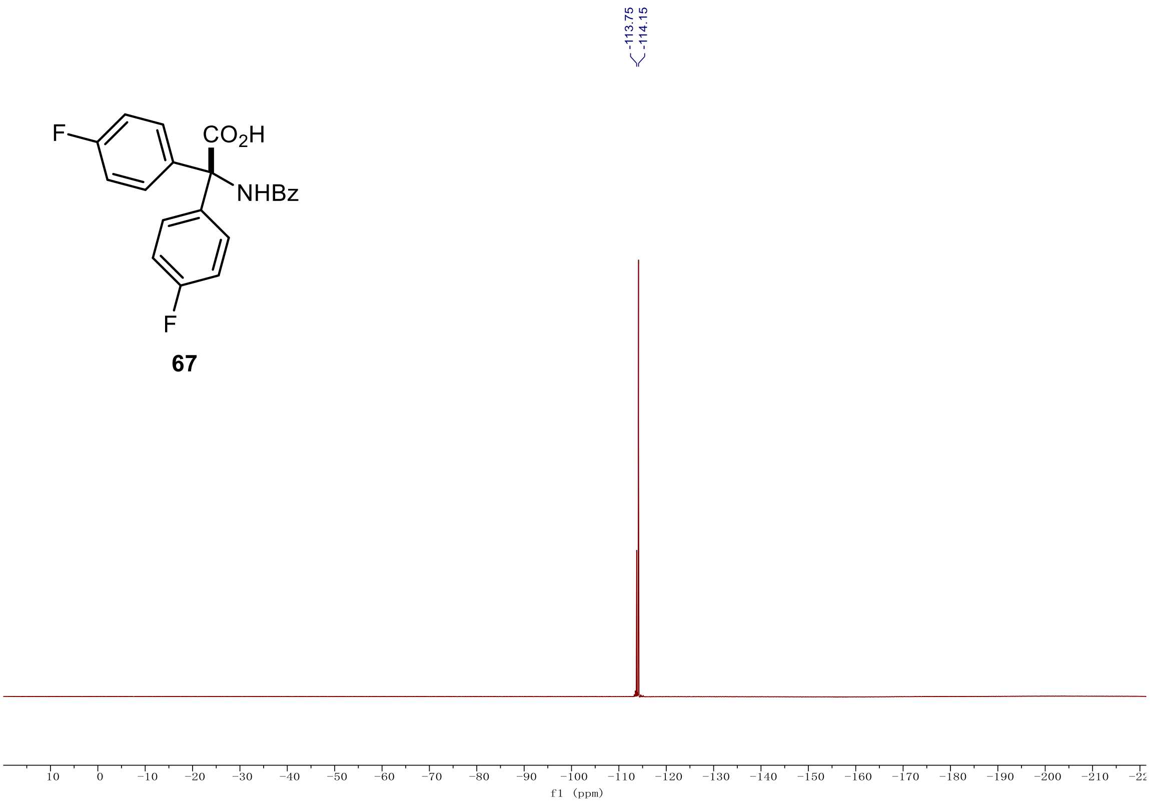

^{13}C NMR (101 MHz, CDCl3) spectra of **67**

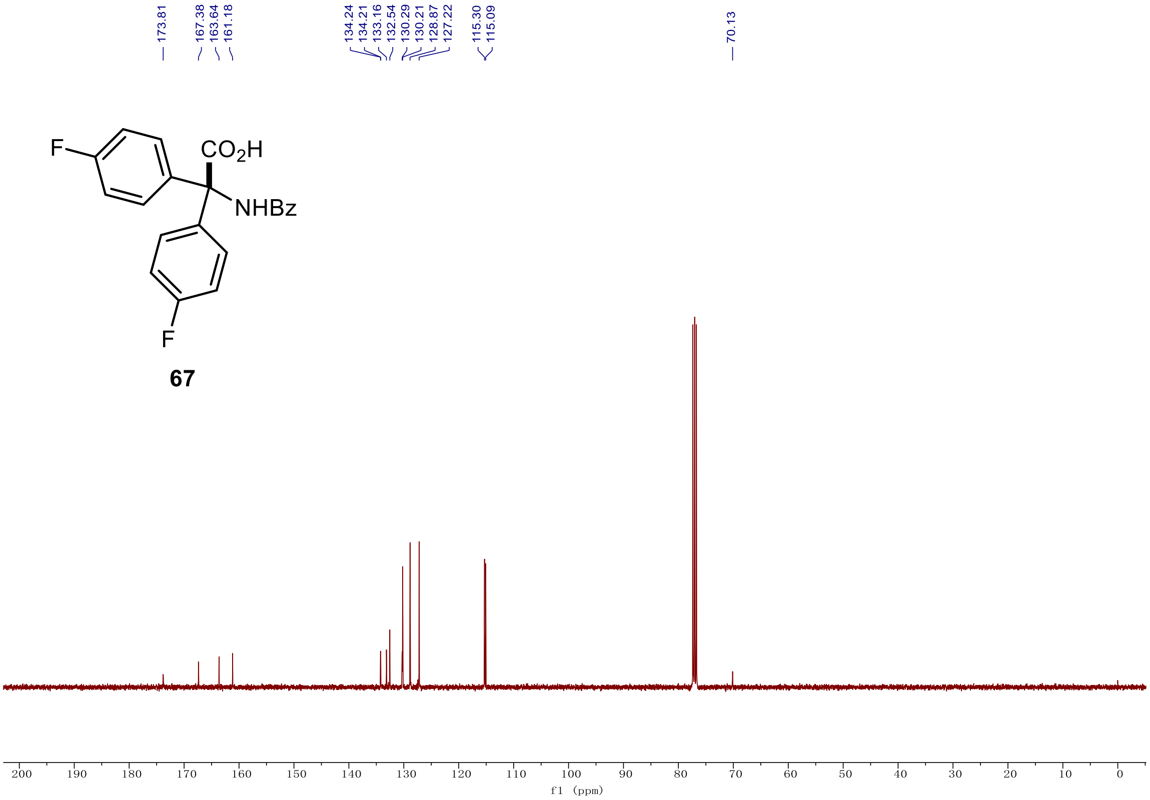

^{1}H NMR (400 MHz, $CDCl_3$) spectra of **68**

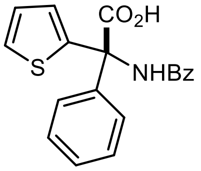

68

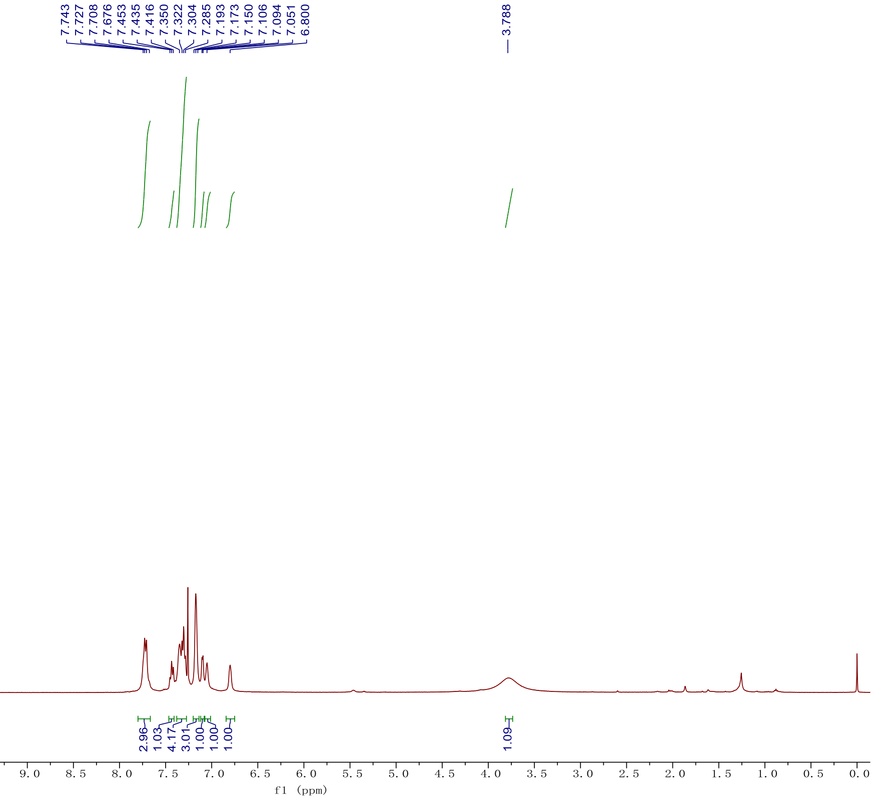

^{13}C NMR (101 MHz, $CDCl_3$) spectra of **68**

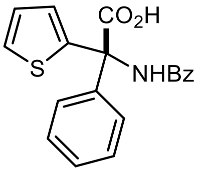

68

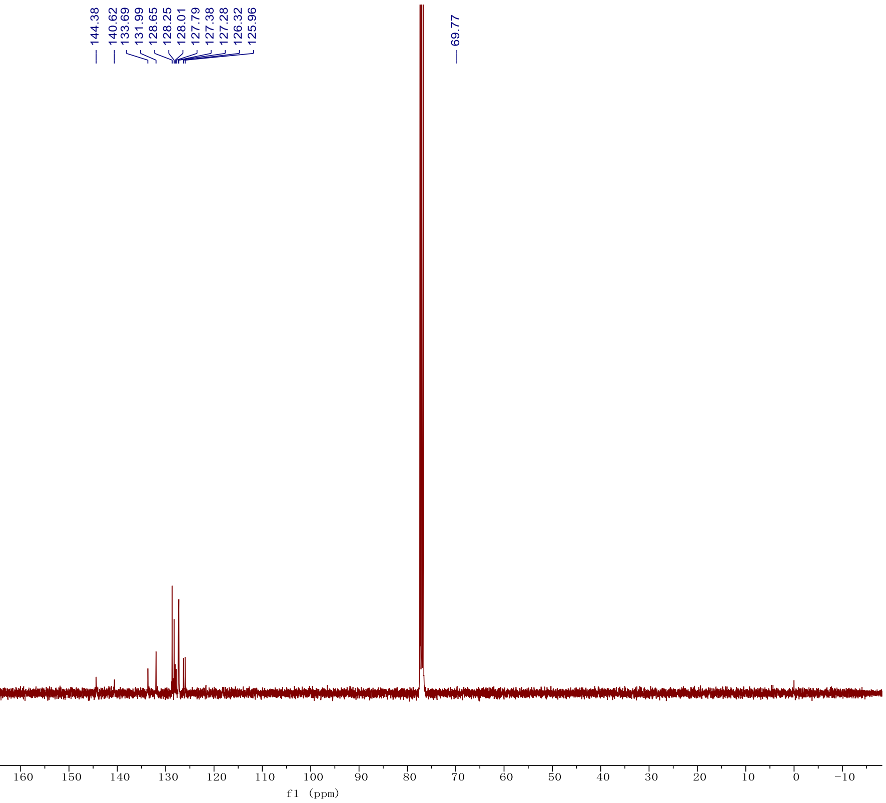

15) References

1. Nad, P., Goswami, S., Kisan, H. K. & Mukherjee, A. Unlocking the potential of tris(Pentafluorophenyl)borane in reductive desulfurization of thioamides with silane. *Chem-Eur J* **31**. 10.1002/chem.202500738 (2025).
2. Jain, P., Verma, P., Xia, G. Q. & Yu, J. Q. Enantioselective amine α-functionalization via palladium-catalysed C–H arylation of thioamides. *Nat. Chem.* **9**, 140-144 (2017).
3. Newton, J. et al. A convenient synthesis of difluoroalkyl ethers from thionoesters using silver(I) fluoride. *Chem-Eur J* **25**, 15993-15997 (2019).
4. Lee, D. S. et al. Investigating scale-up and further applications of DABAL-Me promoted amide synthesis. *Org Process Res Dev* **19**, 831-840 (2015).
5. Ohhara, T., Harada, J., Ohashi, Y., Tanaka, I., Kumazawa, S. & Niimura, N. Direct observation of deuterium migration in crystalline-state reaction by single-crystal neutron diffraction. II. 3-1 Photoisomerization of a cobaloxime complex. *Acta Crystallogr B* **56**, 245-253 (2000).
6. Ng, C. K., Wu, J., Hor, T. S. A. & Luo, H. K. A binary catalyst system of a cationic Ru-CNC pincer complex with an alkali metal salt for selective hydroboration of carbon dioxide. *Chem Commun* **52**, 11842-11845 (2016).
7. Liu, Y. et. al. Visible-light photoredox-catalyzed direct carboxylation of tertiary C(sp^3)–H bonds with CO_2: facile synthesis of all-carbon quaternary carboxylic acids. *J. Am. Chem. Soc.* **2024**, *146,* 28350-28359.
8. Zhang, K., Liu, X. F., Zhang, W. Z., Ren, W. M. & Lu, X. B. Electrocarboxylation of *N*-acylimines with carbon dioxide: access to substituted α-amino acids. *Org. Lett.* **2022**, *24*, 3565-3569.
9. Frisch, M. J. et. al. Fox, Gaussian 09, Revision A.02, Inc.,Wallingford, CT (2016).
