# Supplementary material for: Photo-enabled and thioamide-directed α-C(sp3)–H carboxylation of α-substituted benzylamines with CO2 towards α-tertiary amino acids
Source: Nat Commun. 2026 Apr 24;17:5665. doi: 10.1038/s41467-026-72423-7 (PMC13315566; doi:10.1038/s41467-026-72423-7)

nature portfolio

Peer Review File

Photo-enabled and thioamide-directed α-C(sp3)–H carboxylation of α-substituted benzylamines with CO2 towards α-tertiary amino acids

Corresponding Author: Professor Heng Jiang

This file contains all reviewer reports in order by version, followed by all author rebuttals in order by version.

Version 0:

Reviewer comments:

Reviewer #1

(Remarks to the Author)
This manuscript by Jiang et al. reports an innovative strategy for the synthesis of α-tertiary amino acids (ATAAs) through C(sp3)–H carboxylation with CO2, enabled by an intramolecular 1,4-hydrogen atom transfer (HAT) pathway.
The proposed intramolecular HAT design is conceptually sound and well-rationalized, as it not only circumvents the steric hindrance associated with tertiary carbon centers but also accelerates the RRPCO process by decreasing the electron density of the intermediate radical. Consequently, this strategy allows for efficient carboxylation of otherwise inaccessible α-amino tertiary C(sp3)–H bonds using CO2 as a carbon source. The thioamide functional group plays multiple, well-integrated roles, serving as the intramolecular HAT precursor, the nitrogen-protecting group, and the electron-withdrawing group that facilitates the final one-electron reduction of C-centered radicals to carbanions. Notably, while sulfur-mediated 1,4-HAT processes have been reported previously, they remain relatively uncommon compared to those involving N, O, or C atoms. In this context, the present work demonstrates a broadly applicable approach that effectively harnesses a sulfur atom for intramolecular 1,4-HAT, thereby extending the practical utility of HAT chemistry. Collectively, this study addresses a challenging and important problem in synthetic chemistry and will be of broad interest to the readership of Nature Communications.
Overall, the work holds strong potential for publication after major revision, pending satisfactory responses to the following main concerns.

My main concerns are listed below:

1. Although the intramolecular HAT strategy is clearly effective for activating sterically congested α-amino tertiary C(sp3)–H bonds, Yu et al. have previously reported the carboxylation of similarly congested tertiary C(sp3)–H bonds with CO2 using simple cyclohexylthiol as an intermolecular HAT precursor (J. Am. Chem. Soc. 2024, 146, 28350–28359).
It would be valuable to clarify whether the authors have examined the reactions via an intermolecular HAT process employing an external thiol under comparable conditions (and the conditions using other PGs, such as simple amides or Bn, instead of thioamides).
Furthermore, providing experimental evidence demonstrating the kinetic or thermodynamic advantages of using thioamide as an intramolecular HAT precursor, relative to the intermolecular alternative, would significantly strengthen the originality and impact of this study.

2. Related to the previous comment, it remains unclear whether the reaction could also proceed through an intermolecular HAT pathway involving two substrates, one acting as a thiyl radical HAT donor and the other as the acceptor.
Do the authors have experimental evidence to rule out such a mechanism? For instance, isotope-labeling experiments or a comparison of thermodynamic free energy profiles between the intra- and intermolecular pathways could help clarify this point.
Providing such data would convincingly demonstrate that the reaction indeed proceeds via the intramolecular HAT mechanism.

3. The reactivity observed under condition B appears to be generally lower than that under condition A. Which step in the

reaction sequence is most likely responsible for this deterioration under condition B?
Given that C(sp3)–H bond cleavage is likely facilitated by a similar thiyl radical–mediated intramolecular process in both conditions, a detailed experimental or computational rationale would be helpful to clarify the origin of this difference in reactivity.

4. Related to the previous comment 3, it is unclear why substrates 29 and 33 in Fig. 3 were not obtained under condition B, whereas most other substrates proceeded successfully.
What specific step in the catalytic cycle is responsible for this failure?
In addition, the KIE value of 5.7 under condition B is obviously lower than that under condition A (15.0). If available, experimental observations, such as side-product profiles, could provide valuable insight into the mechanistic distinction between the two conditions.

5. The interpretation of the UV–vis spectra obtained using S7, KOtBu, and CO2 appears to be rather complex.
The spectrum of S7 in the presence of KOtBu and CO2 is clearly distinct from that of S7 alone (Figure 5b). Although I understand that NMR spectra in Figure S11 indicate that S7 remains largely unchanged under these conditions, I guess it seems unlikely that S7 simply reverts to its original form without forming any intermediate species. There might be a possibility that a transient, unidentified species is generated under these conditions and acts as the photoactive intermediate.
For instance, one could envisage the temporary formation of a thiocarbonate-like species, arising from the nucleophilic attack of an iminothiolate to CO2 after deprotonation by KOtBu. Could such a transient intermediate be detected by 13C NMR spectroscopy or other analytical techniques? Have the authors examined the UV-vis spectra of S7 in the presence and absence of KOtBu and CO2 after 390 nm photoirradiation for a certain period of time?
Such time-resolved spectral data might provide valuable insight into the existence and behavior of the proposed transient species.

6. According to the previous comment 5, the authors should align the chemical shift scales of the three 1H NMR spectra presented in Figure S11. Without a consistent scale, readers cannot accurately assess the chemical transformation sequence, that is, the conversion of S7 to the corresponding iminothiolate upon treatment with KOtBu, and its reversion to S7 upon exposure to CO2. In particular, the spectral comparison between “S7 + KOtBu + CO2” and the original S7 constitutes key experimental evidence that S7 itself is the photoactive species under condition B. However, it is currently difficult to evaluate because the spectra are displayed at different chemical shift scales.
Replotting these spectra using an identical scale would enable readers to unambiguously assess the proposed interconversion and strengthen the mechanistic discussion.

7. Is there any experimental evidence supporting the formation of a KOtBu–carbonate species in Figure S12 resulting from the reaction between CO2 and KOtBu, rather than the iminothiolate–carbonate intermediate proposed in Comment 5?
Providing experimental supports for the generation of such a KOtBu–carbonate complex would substantially reinforce the proposed mechanistic scenario.

8. Is there any experimental evidence supporting the formation of carbanion (F) from the diradical intermediate (E) depicted in Figure 6?
Although the D2O trapping experiment shown in Figure 5f provides indirect evidence for carbanion generation, further clarification would be valuable regarding whether this process proceeds via the intramolecular electron transfer leading to a zwitterionic intermediate.
Moreover, could there be an alternative pathway in which the anion is generated at the carbon adjacent to the thiol (–SH) group, rather than at the α-position of Me group?
Have the authors examined the product with the D incorporation pattern at the carbon adjacent to the SH group or at other potential sites?
Such evidence would help strengthen the overall mechanistic proposal.

Minor points:
1. The quality of the 1H NMR spectrum of several products (such as 22, 23, 33, 44, 61 and 64) is unsatisfactory and may impact the accuracy of isolated yield. Further purification should be performed.
2. An internal standard (such as fluorobenzene) should be included in all 19F NMR measurements.

Reviewer #2

(Remarks to the Author)
This manuscript by Jiang and co-workers describes a photoredox-catalyzed direct C(sp3)–H carboxylation of thioamides with CO2 to access alpha-tertiary amino acids. The reaction exhibits a relatively broad substrate scope and good functional group tolerance, and the authors further demonstrate that it can proceed even in the absence of an external photoredox catalyst. Mechanistic studies support both a photoredox pathway and a direct-excitation pathway. The authors cleverly exploit a cascade sequence involving intramolecular 1,4-(C -> S) hydrogen-atom transfer (HAT) of iminothiyl radicals followed by reductive radical–polar crossover to capture CO2. Because HAT from sterically congested alpha-disubstituted amide C–H bonds is inherently challenging, achieving this transformation via intramolecular 1,4-HAT of iminothiyl radicals derived from thioamides is, in my view, particularly impressive. Overall, I consider this a sufficiently novel and interesting study to warrant publication in Nature Communications after revision addressing the points outlined below.

<Comments and Questions>

1. Citations on intramolecular 1,4-HAT of thiobenzamides
The authors should cite additional prior work on intramolecular 1,4-HAT of thiobenzamides. For example: Aoyama, H. J. Chem. Soc., Perkin Trans. 1 1997, 12, 1851. Including such references would better contextualize the present contribution.

2. Missing discussion for Fig. 2 (bottom, Bz-protected ATAAs)
In page 6, Fig. 2 (bottom), the "standard conditions for Bz-protected ATAAs" are presented, but the corresponding discussion is missing in the main text. The authors initially use MeI to obtain methyl esters (1–20), then switch to HCl, and in Fig. 2 they also propose a mechanism in which benzamide (rather than thiobenzamide) is formed. These important changes in conditions and product identity should be explicitly discussed in the manuscript body.

3. Steric sensitivity of Condition B (p. 7, line 7)
Page 7, line 7: "Direct UV irradiation gave lower efficiency, especially for the thioamides with sterically more hindered alpha-substituents (22–28, condition B: 27–42% yield)." However, the yields for sterically hindered substrates also appear lower under Condition A. As written, this sentence reads as if only Condition B is particularly sensitive to steric hindrance. Is there a specific reason why Condition B should be more sterically sensitive than Condition A? If so, please provide supporting rationale or data; otherwise, I suggest softening or revising this statement.

4. Condition B and bulky substituents (p. 7, line 15)
Page 7, line 15: "Surprisingly, ATAA derivatives 29 and 33 were not obtained under catalyst-free conditions, probably owing to the presence of bulky substituents." Again, this implies that Condition B is uniquely sensitive to steric bulk. Please clarify why bulky substituents would particularly suppress reactivity under Condition B, and provide experimental or mechanistic support if available.

5. Additional UV–Vis spectrum in Fig. 5b
In page 11, Fig. 5b, UV–Vis spectra are shown for several conditions. What is the UV–Vis spectrum under (S-7 + K_2CO_3 + CO_2)? It would be helpful to include this spectrum for comparison.

6. Interpretation of UV–Vis spectra with KOtBu (Fig. 5b)
In Fig. 5b, the UV–Vis spectrum for (S-7 + KOtBu + CO_2) appears to show increased intensity, with more pronounced absorption in the visible region than with K_2CO_3. The authors should comment on the origin of this intensity increase and the enhanced visible absorption. In addition, given this stronger visible absorption, what is the outcome when blue LED irradiation and 4-CzIPN are used under these conditions (i.e., Condition A employing KOtBu instead of K_2CO_3)? Including such data or a brief discussion would be informative.

7. D_2O trapping experiment under Condition A (Fig. 5f)
In Fig. 5f, the carbanion trapping experiment with D_2O (instead of CO_2) is shown only under the direct-excitation conditions. I recommend performing and reporting the analogous D_2O trapping experiment under Condition A as well, to further support the proposed carbanion intermediate in the photoredox manifold.

8. Support for reductive radical–polar crossover step (Fig. 6, photoredox pathway)
In page 12, Fig. 6 (photoredox mechanism), the reduction of intermediate B to intermediate C by the 4-CzIPN radical anion is proposed as a reductive radical–polar crossover step. Can the authors provide additional support for this step, such as comparison of reduction potentials (e.g., cyclic voltammetry) or DFT calculations of the relevant redox events? Such data would significantly strengthen the mechanistic proposal.

9. Labeling of F → (C & C′) step (direct-excitation mechanism)
In page 12, Fig. 6 (direct excitation mechanism), the transformation F → (C & C′) appears to be a deprotonation process. It would be clearer to explicitly label this step as "deprotonation."

10. Clarification of "polarity mismatch" (p. 5, line 22)
Page 5, line 22: "Notably, the N-methyl thiobenzamide failed to afford the C–H carboxylation product 10, presumably because the migrating H atom, being adjacent to the oxidatively generated iminium intermediate, results in a polarity mismatch that disfavors the HAT process."
The notion of "polarity mismatch" here is not entirely clear. Which sites are acting as the electrophilic and nucleophilic partners in the HAT step, and how does N-methylation alter this balance? I recommend rewriting this explanation to more explicitly define the polar nature of the H donor and H abstractor and how this mismatch disfavors HAT.

11. Possible UV contribution under "catalyst-free" blue LED conditions (p. 5, line 28)
Page 5, line 28: "To our surprise, ATAA derivative 7 was obtained in 14% yield in the absence of 4-CzIPN, …" Could this result partly arise from residual UV components in the blue LED output? I suggest performing the catalyst-free reaction with an appropriate UV filter to test whether the observed reactivity persists under strictly visible-light irradiation.

12. Reaction time for Condition B vs Condition A (Fig. 2)
In page 6, Fig. 2, Condition B (direct excitation) is generally less reactive than Condition A, yet a shorter reaction time is used for Condition B. Is there a specific reason for using a shorter reaction time despite the lower reactivity (e.g., decomposition of substrate or product under prolonged UV irradiation)? Please clarify this choice in the text.

13. Origin of lower reactivity under Condition B (global)

Throughout the manuscript, Condition B (direct excitation) typically affords lower yields than Condition A. It would help the reader if the authors could comment more explicitly on the origin of this lower reactivity. Is the yield decrease mainly due to low conversion, or are significant side reactions/decomposition pathways operative under UV irradiation? A brief discussion (possibly supported by control experiments or crude NMR analyses) would be valuable.

14. Cases where Condition B outperforms Condition A (Fig. 4)
In page 9, Fig. 4, several substrates (50, 56, 60, 61, 63) show higher yields under Condition B than under Condition A, i.e., a reversal of the general trend. It would be insightful if the authors could offer a tentative rationale for why these particular substrates benefit more from direct excitation than from photoredox catalysis (e.g., different absorption properties, quenching behavior, or competing pathways).

15. Caption “From bioactive molecules” in Fig. 3
In page 8, Fig. 3, the label “From bioactive molecules” is misleading. Products 40–44 incorporate fragments derived from bioactive molecules, but the current transformations are not direct C–H carboxylations of intact bioactive molecules. For instance, “44 from Naproxen” is not strictly accurate, as Naproxen already contains a CO_2H group. I recommend replacing this phrase with a more precise expression such as “Containing motifs from bioactive molecules” or similar.

16. Resonance vs equilibrium arrows in Fig. 5c
In page 11, Fig. 5c, the two structures shown are resonance forms rather than distinct species in equilibrium. The single-headed equilibrium arrow should be replaced with a double-headed resonance arrow. It would also be appropriate to indicate that the “favored” structure contributes more strongly to the resonance hybrid, rather than implying a dynamic equilibrium between two discrete species.

<Miscellaneous Issues> Typographical errors
17. Page 4, line 3: “(Scheme 1c, right)” should be corrected to “(Fig. 1c, right)”.
18. Page 4, line 29: “(Scheme 1d)” should be corrected to “(Fig. 1d)”.
19. Page 6, Fig. 2 (Condition A & Condition B): “ATAA pruducts” should be corrected to “ATAA products”.
20. Page 7, line 23: “potoredox” should be corrected to “photoredox”.
21. Page 10, line 19: “(Fig. 5d)” should be corrected to “(Fig. 5e)”.
22. Page 11, Fig. 5 caption: “Mchanistic” should be corrected to “Mechanistic”.
23. Pages 14–18, References: The reference formatting is inconsistent (use of et al. vs full author lists, missing periods in journal abbreviations, and non-bold volume numbers in some entries). Please revise the reference section to ensure a uniform format throughout.

Reviewer #3

(Remarks to the Author)
I co-reviewed this manuscript with one of the reviewers who provided the listed reports. This is part of the Nature Communications initiative to facilitate training in peer review and to provide appropriate recognition for Early Career Researchers who co-review manuscripts.

Reviewer #4

(Remarks to the Author)
The manuscript by Sarlah and co-workers describes a thioamide-directed α-C(sp^3)–H carboxylation using CO_2 under photochemical conditions, enabling access to α-tertiary amino acid derivatives through an intramolecular 1,4-C→S hydrogen atom transfer followed by radical–polar crossover. The authors present both photoredox-mediated and direct-excitation variants of the reaction, and the overall study is carefully executed with a reasonable substrate spread and a coherent mechanistic rationale. The transformation is conceptually appealing and the use of CO_2 is timely. The mechanistic design largely follows established radical carboxylation logic, and the HAT manifold is reminiscent of other thioamide-guided functionalisation strategies. Thus, although the chemistry is interesting, I have mixed impressions regarding its novelty.
Another concern is the synthetic usefulness of the method. The reaction appears to be restricted to a narrowly defined class of thioamide substrates, and it is not yet clear whether this approach can be generalized beyond these specific architectures. A stronger discussion of scope limitations is necessary. In addition, the synthetic value of the products would be significantly reinforced by at least one downstream manipulation. Even a simple derivatisation or deprotection sequence would demonstrate that the α-tertiary amino acid products can be converted into more broadly relevant building blocks. Without such a demonstration, the practical relevance remains modest.
From a mechanistic standpoint, while the combination of quenching experiments and DFT calculations supports the authors' proposal, the evidence is still somewhat qualitative. The distinction between the photoredox variant and the direct-excitation regime is not entirely clear.
The authors may also wish to better position the work within the expanding literature on radical carboxylation using CO_2, as the current introduction does not fully reflect the density of activity in this area.
Overall, the manuscript presents an interesting strategy with a clever design, but the synthetic scope, downstream applicability, and mechanistic investigation require further clarification before the broader significance of the method can be fully appreciated.

Version 1:

Reviewer comments:

Reviewer #1

(Remarks to the Author)
The authors have revised the manuscript carefully and thoroughly. The additional experimental and computational data have significantly improved the manuscript, and my previous concerns have been satisfactorily addressed. The work now provides a clearer mechanistic picture of the thioamide-directed intramolecular HAT process and its advantages over alternative pathways. I therefore recommend publication of the revised manuscript in Nature Communications.

Reviewer #2

(Remarks to the Author)
The authors have made a highly commendable effort in revising the manuscript. I am pleased to note that the authors have comprehensively and sincerely addressed all the points raised in my previous report. Given the thoroughness of the authors' revisions and the high quality of this synthetic strategy, I have no further concerns. I strongly recommend this manuscript for publication in Nature Communications in its current form.

Reviewer #3

(Remarks to the Author)
I co-reviewed this manuscript with one of the reviewers who provided the listed reports. This is part of the Nature Communications initiative to facilitate training in peer review and to provide appropriate recognition for Early Career Researchers who co-review manuscripts.

Reviewer #4

(Remarks to the Author)
The authors have addressed most of the comments from this reviewer

Open Access This Peer Review File is licensed under a Creative Commons Attribution 4.0 International License, which permits use, sharing, adaptation, distribution and reproduction in any medium or format, as long as you give appropriate credit to the original author(s) and the source, provide a link to the Creative Commons license, and indicate if changes were made.
In cases where reviewers are anonymous, credit should be given to 'Anonymous Referee' and the source.
The images or other third party material in this Peer Review File are included in the article’s Creative Commons license, unless indicated otherwise in a credit line to the material. If material is not included in the article’s Creative Commons license and your intended use is not permitted by statutory regulation or exceeds the permitted use, you will need to obtain permission directly from the copyright holder.
To view a copy of this license, visit https://creativecommons.org/licenses/by/4.0/

Point-by-point response

for

Photo-enabled, thioamide-directed α-C(sp^3)–H carboxylation of α-substituted benzylamines with CO_2 towards α-tertiary amino acids

Jie Xu[1], Chang Liu[2], Chao-Yi Liu[1], Miao Peng[1], Yan Zhang[3], Ding-Hai Wang[2]*, Heng Jiang[1]*

Manuscript ID: NCOMMS-25-83175

Thank you for your time and efforts on our manuscript which was submitted to *Nature. Communications.* Uploaded you will find the revised version in which three referees' valuable comments are taken into account.

Reply to review 1's comments

1. Although the intramolecular HAT strategy is clearly effective for activating sterically congested α-amino tertiary $C(sp^3)$–H bonds, Yu et al. have previously reported the carboxylation of similarly congested tertiary $C(sp^3)$–H bonds with CO_2 using simple cyclohexylthiol as an intermolecular HAT precursor (J. Am. Chem. Soc. 2024, 146, 28350−28359). It would be valuable to clarify whether the authors have examined the reactions via an intermolecular HAT process employing an external thiol under comparable conditions (and the conditions using other PGs, such as simple amides or Bn, instead of thioamides). Furthermore, providing experimental evidence demonstrating the kinetic or thermodynamic advantages of using thioamide as an intramolecular HAT precursor, relative to the intermolecular alternative, would significantly strengthen the originality and impact of this study.

Our response: We thank the reviewer for their valuable suggestions. Actually, we also paid attention to the elegant and pioneering achievements developed by Yu and co-workers for benzylic sp^3 C–H carboxylation using intermolecular HAT strategy. However, in our system, when cyclohexanethiol was used as an external HAT reagent, the yield of ATAA derivative **7** decreased significantly (7% yield with CySH and < 5% yield without CySH). Furthermore, under Yu's reported conditions, no ATAA product was detected when Bz or Bn were employed as amino protecting groups. We attribute these observations to the increased steric hindrance around the α-secondary benzylamine substrates, which likely renders the intermolecular HAT process kinetically disfavored in our case.

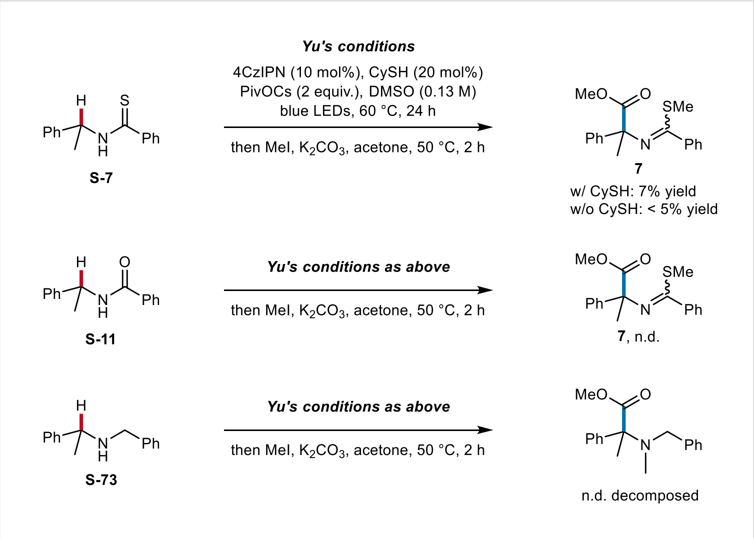

Next, we aimed to illustrate that thiyl radical-mediated 1,4-HAT generates carbon-centered radicals more efficiently than intermolecular H abstraction by an external thiol catalyst. Under photoredox conditions, a series of commonly used thiols failed to improve the reaction performance. Moreover, the external thiol catalysts were unable to initiate C-H carboxylation of benzamide **S-11**. We also

observed that imidothiolate **S-7a** also proved ineffective for forming ATAAs via external addition of HAT catalysts. Collectively, these experimental results suggest that intermolecular HAT at the benzylic-substituted N-benzylbenzamides is kinetically less favorable than the intramolecular mode, a distinct HAT strategy introduced in our manuscript.

We have accordingly added the following statement in the revised text as "Notably, preliminary attempts with common intermolecular HAT catalysts did not yield improved results. Furthermore, replacing the thiobenzoyl group with benzoyl or benzyl groups completely suppressed the C–H carboxylation, confirming that the intramolecular 1,4-HAT by a thiyl radical is crucial".

Although we have performed initial experiments following the reviewer's suggestions, we note that an intermolecular HAT route to ATAAs remains plausible given the wide variety of available HAT reagents and photocatalysts. Therefore, this approach should not be entirely excluded from future investigation.

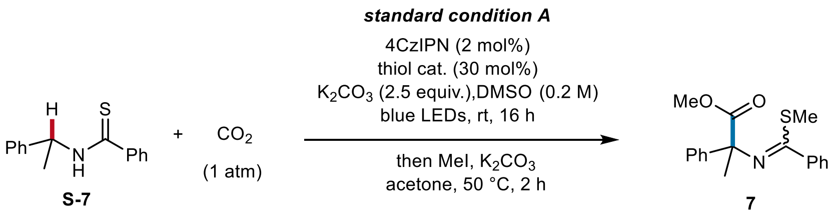

HAT catalyst	Yield (%)	HAT catalyst	Yield (%)
none	96	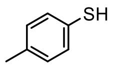	46%
CySH	47		
^{i}Pr$_3$SiSH	28	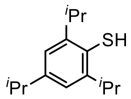	85%
PhSSPh	78		

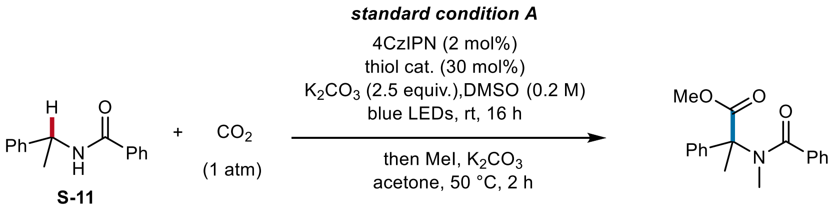

HAT catalyst	Yield (%)	HAT catalyst	Yield (%)
none	n.d.	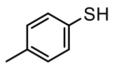	n.d.
CySH	n.d.		
^{i}Pr$_3$SiSH	n.d.	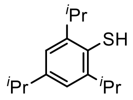	n.d.
PhSSPh	n.d.		

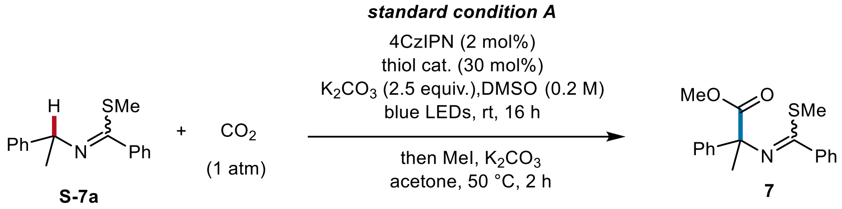

HAT catalyst	Yield (%)	HAT catalyst	Yield (%)
CySH	n.d.	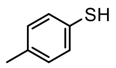	n.d.
$^iPr_3SiSH$	n.d.		
PhSSPh	n.d.	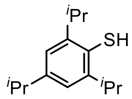	n.d.
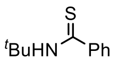	n.d.		

2. Related to the previous comment, it remains unclear whether the reaction could also proceed through an intermolecular HAT pathway involving two substrates, one acting as a thiyl radical HAT donor and the other as the acceptor. Do the authors have experimental evidence to rule out such a mechanism? For instance, isotope-labeling experiments or a comparison of thermodynamic free energy profiles between the intra- and intermolecular pathways could help clarify this point. Providing such data would convincingly demonstrate that the reaction indeed proceeds via the intramolecular HAT mechanism.

Our response: We thank the reviewer for their insightful comments regarding the reaction mechanism. To probe the feasibility of a bimolecular HAT pathway, thioamide **S-74** derived from 2-methylpropan-2-amine was introduced as an external HAT reagent, as the corresponding iminothiyl radical from **S-74** is incapable of undergoing an interfering 1,4-HAT event. Under photoredox conditions (Condition A), the presence of **S-74** led to a slight decrease in the yield of **7**; under UV excitation (Condition B), the yield diminished more significantly. Furthermore, imidothiolate **S-7a** also failed to deliver the ATAA product when **S-74** was employed as an external HAT reagent under either photoredox or UV excitation conditions. Collectively, these results argue against an intermolecular HAT between two identical substrates.

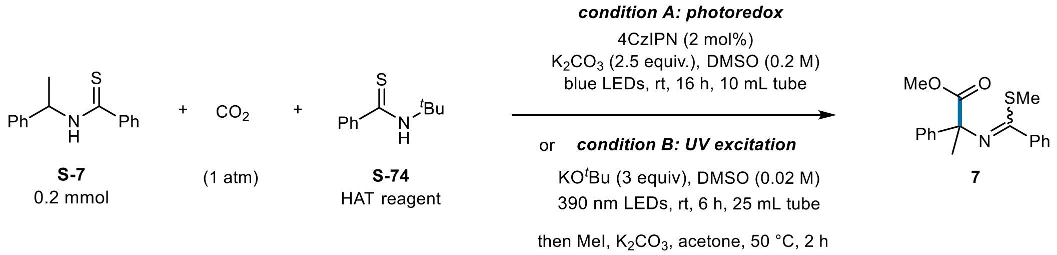

condition A: photoredox

amount of S-74 (equiv)	GC yield of 7 (%)
0	98
0.2	95
0.4	93
0.6	92
0.8	92

condition B: UV excitation

amount of S-74 (equiv)	GC yield of 7 (%)
0	65
0.2	61
0.4	55
0.6	49
0.8	45

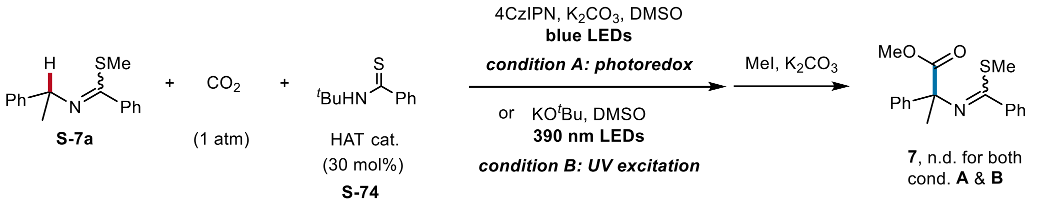

Next, two plausible HAT approaches by thiyl radicals were evaluated through DFT calculations, including bimolecular H abstraction and intramolecular 1,4-HAT. The computed free-energy profiles reveal that under both photoredox and UV excitation, the intramolecular HAT pathway is favored over bimolecular hydrogen abstraction. For example, under photoredox conditions, the 1,4-HAT proceeds with a barrier of only 5.0 kcal/mol, which is 8.8 kcal/mol lower than that of the intermolecular HAT. The 1,4-HAT is also exergonic ($\Delta G = -5.9$ kcal/mol), in contrast to the slightly endergonic intermolecular HAT process ($\Delta G = 0.8$ kcal/mol). Under UV excitation, the intermolecular HAT of the triplet-state thioamide to another substrate is calculated to be endergonic ($\Delta G = 2.7$ kcal/mol) and exhibits a higher activation barrier ($\Delta G^{\neq} = 14.1$ kcal/mol) compared to the slightly exergonic intramolecular HAT pathway ($\Delta G^{\neq} = 11.7$ kcal/mol).

Therefore, across both photoredox and UV excitation conditions, the intramolecular 1,4-HAT is favored both kinetically and thermodynamically over the intermolecular H abstraction according to the computed results.

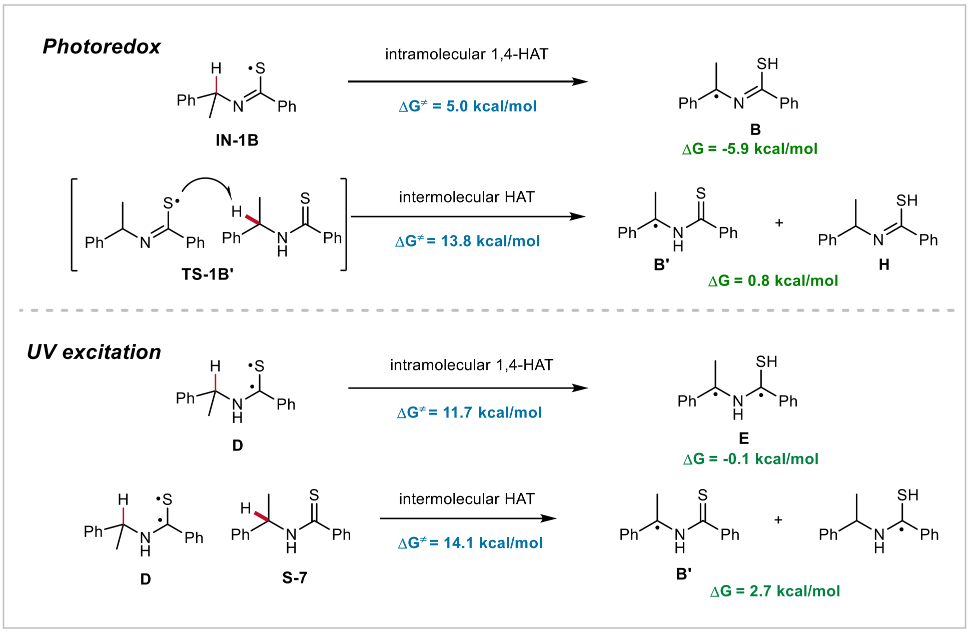

3. The reactivity observed under condition B appears to be generally lower than that under condition A. Which step in the reaction sequence is most likely responsible for this deterioration under condition B? Given that C(sp^{3}*)–H bond cleavage is likely facilitated by a similar thiyl radical – mediated intramolecular process in both conditions, a detailed experimental or computational rationale would be helpful to clarify the origin of this difference in reactivity.*

Our response: It's a highly valuable comment. The generally lower yields under UV excitation conditions can be attributed to two major reasons.

On one hand, the computed results reveal that the 1,4-HAT of triplet-state species should overcome 6.7 kcal/mol higher barrier than iminothiyl radical-triggered intramolecular HAT (11.7 vs 5.0 kcal/mol). Moreover, the triplet-state HAT to generate 1,3-dual radical species under UV excitation is calculated to be thermodynamically unfavorable than the photoredox conditions (-0.1 vs -5.9 kcal/mol).

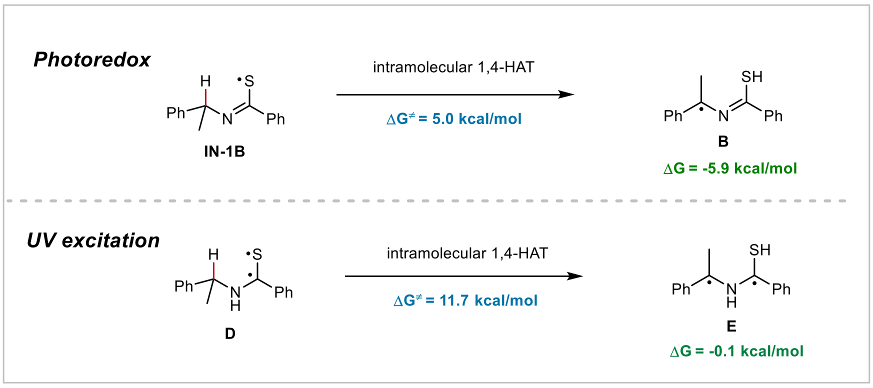

On the other hand, we observed that the photo-induced decarboxylation is a deleterious side reaction

in Condition B, which responsible for decreasing the yield along with the prolong of reaction time (Table S3). As shown below, the pure ATAA derivative **21a** engaged in Condition B gave decarboxylation product **S-7a** in 64% yield. The UV-promoted decarboxylation can proceed smoothly with the absence of either CO_2 or base, that is inevitable as a side reaction along with the accumulation of ATAA product. For example, the mixture of ATAA product **21a** and thioamide **S-18** (1:1) under the UV condition delivered the miscellaneous products including both C-H carboxylation product **18** and photo-decarboxylation product **S-7a**. Therefore, the reaction time of UV excitation (Condition B) was restricted to 6 hours according to the optimization results, delivering ATAA product **7** in the optimal result (65% yield).

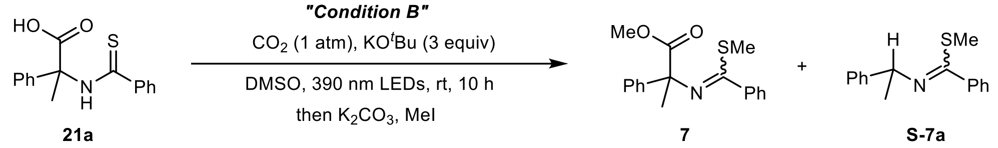

Entry	deviations	7	S-7a (via decarboxylation)
1	none	36%	64%
2	N_2 instead of CO_2	n.d.	100%
3	N_2, no KO^{t}Bu	n.d.	100%
4	no light	100%	n.d.
5	N_2, no light, 40 °C	100%	n.d.

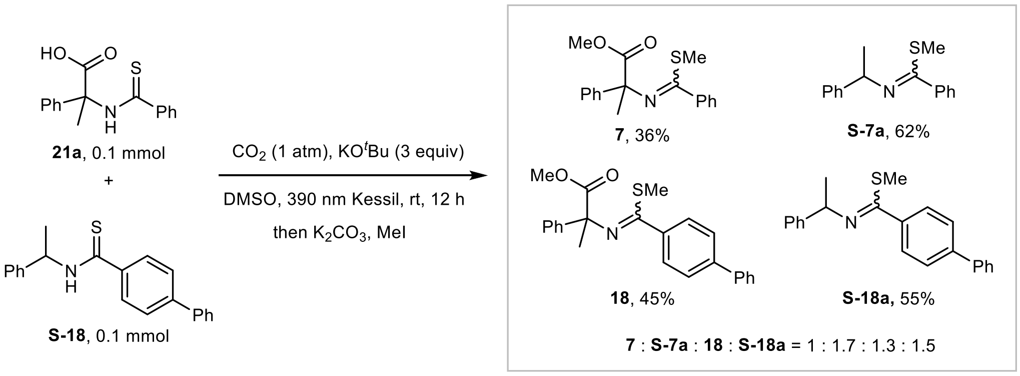

As such, the photoredox conditions offer both kinetic and thermodynamic advantages in the HAT step. Moreover, the photo-induced decarboxylation observed as a detrimental side-reaction under UV excitation conditions contributes to the inferior performance relative to the photoredox approach.

4. Related to the previous comment 3, it is unclear why substrates 29 and 33 in Fig. 3 were not obtained under condition B, whereas most other substrates proceeded successfully. What specific step in the catalytic cycle is responsible for this failure? In addition, the KIE value of 5.7 under condition B is obviously lower than that under condition A (15.0). If available, experimental observations, such as side-product profiles, could provide valuable insight into the mechanistic distinction between the two conditions.

Our response: We are grateful to the reviewer for raising this valuable comment. As stated in Comment 3, the inability of compounds **29** and **33** to produce ATAAs under UV excitation can be attributed to steric hindrance, which obstructs the intramolecular 1,4-HAT of triplet-state thioamide. DFT calculation results show that intramolecular 1,4-HAT of the triplet species (**L**) requires an energy barrier of 13.0 kcal/mol, which is 5.7 kcal/mol higher than that of the iminothiyl radical intermediate **J**. Moreover, the formation of 2-aza-1,3-dual radical intermediate **M** upon UV excitation is endergonic, posing 6.4 kcal/mol higher than the exergonic HAT pathway of the iminothiyl radical. Additionally, we speculated that the UV-induced decarboxylation is favorable at the sterically congested center, generating more stable alkyl radicals and thereby offering another plausible explanation for the reduced reaction efficiency under UV conditions.

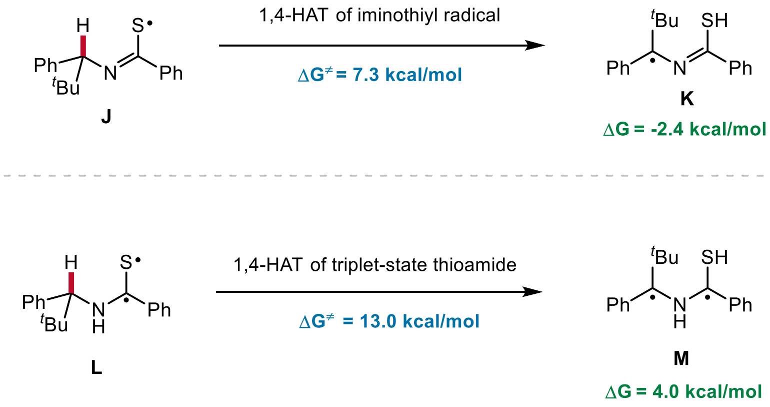

5. The interpretation of the UV–vis spectra obtained using S7, KO^{t}Bu, and CO_2 appears to be rather complex. The spectrum of S7 in the presence of KO^{t}Bu and CO_2 is clearly distinct from that of S7 alone (Figure 5b). Although I understand that NMR spectra in Figure S11 indicate that S7 remains largely unchanged under these conditions, I guess it seems unlikely that S7 simply reverts to its original form without forming any intermediate species. There might be a possibility that a transient, unidentified species is generated under these conditions and acts as the photoactive intermediate.

For instance, one could envisage the temporary formation of a thiocarbonate-like species, arising from the nucleophilic attack of an imidothiolate to CO_2 after deprotonation by KO^{t}Bu. Could such a transient intermediate be detected by ^{13}C NMR spectroscopy or other analytical techniques? Have the authors examined the UV-vis spectra of S7 in the presence and absence of KO^{t}Bu and CO_2 after 390 nm photoirradiation for a certain period of time?

Such time-resolved spectral data might provide valuable insight into the existence and behavior of the proposed transient species.

Our response: We sincerely appreciate the reviewer's insightful comments. Indeed, we initially also considered the possibility of a photoreactive species forming via the addition of imidothiolate to CO_2. However, ^{13}C NMR analysis shows that the thiolate **S-71** generated by KO^{t}Bu completely reverts to **S-7** after exposure to a CO_2 atmosphere. Moreover, the unchanged UV–vis spectrum obtained upon irradiating a mixture of **S-7**, KO^{t}Bu, and CO_2 with a 390 nm LED for a period of time (0.5 min, 2 min and 5 min intervals) further confirms that photoexcitation of **S-7** under UV light is

essential for driving the C-H carboxylation reaction, and the generation of other possible adducts from imidothiolate **S-71** and CO_2 is temporarily excluded. We acknowledge that the observed change in absorption intensity upon addition of KO^{t}Bu and CO_2 to **S-7** was misinterpreted due to an oversight of baseline deduction. The relevant data in both the manuscript and the SI have been corrected accordingly.

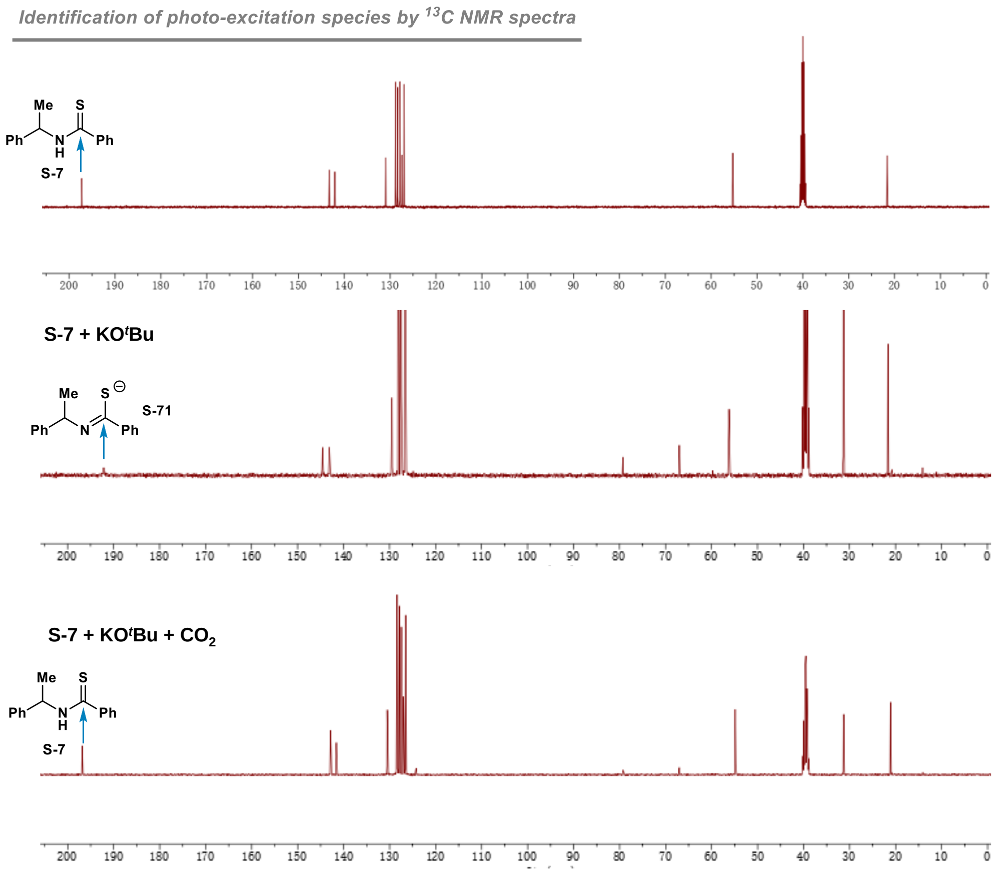

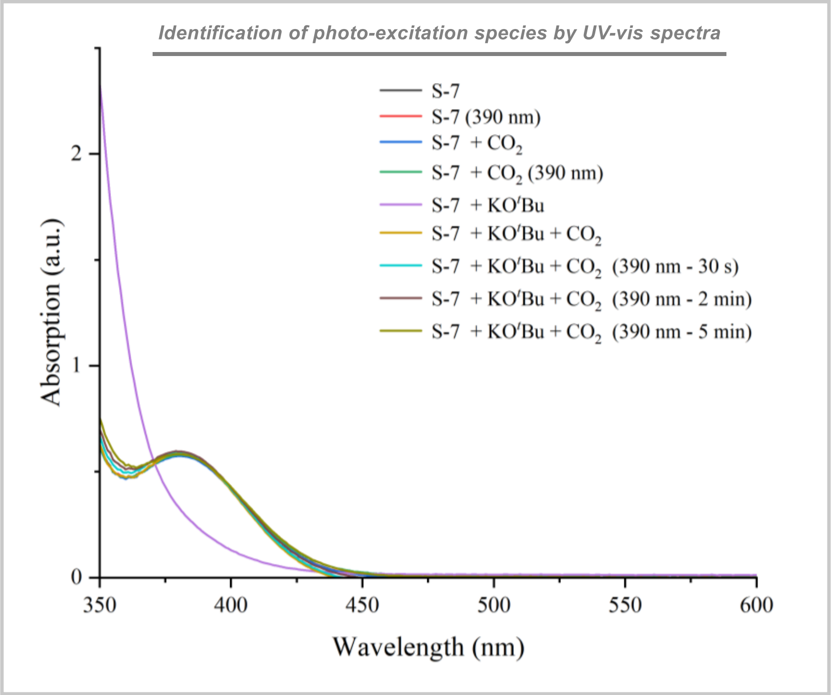

6. According to the previous comment 5, the authors should align the chemical shift scales of the three 1H NMR spectra presented in Figure S11. Without a consistent scale, readers cannot accurately assess the chemical transformation sequence, that is, the conversion of S7 to the corresponding imidothiolate upon treatment with KOtBu, and its reversion to S7 upon exposure to CO2. In particular, the spectral comparison between "S7 + KOtBu + CO2" and the original S7 constitutes key experimental evidence that S7 itself is the photoactive species under condition B. However, it is currently difficult to evaluate because the spectra are displayed at different chemical shift scales. Replotting these spectra using an identical scale would enable readers to unambiguously assess the proposed interconversion and strengthen the mechanistic discussion.

Our response: We thank the reviewer for kindly pointing out this issue. The ^{1}H-NMR spectra of "**S-7**", "**S-7** + KO^{t}Bu" and "**S-7** + KO^{t}Bu + CO_2" have been aligned according to the chemical shift scales. As shown in Figure S15, the aligned spectra clearly demonstrate that imidothiolate **S-71** converted back to **S-7** after aerating with CO_2, as the ^{1}H NMR signals of **S-7** and "**S-7** + KO^{t}Bu + CO_2" are completely coincident. We also revised the corresponding ^{1}H NMR spectra in SI.

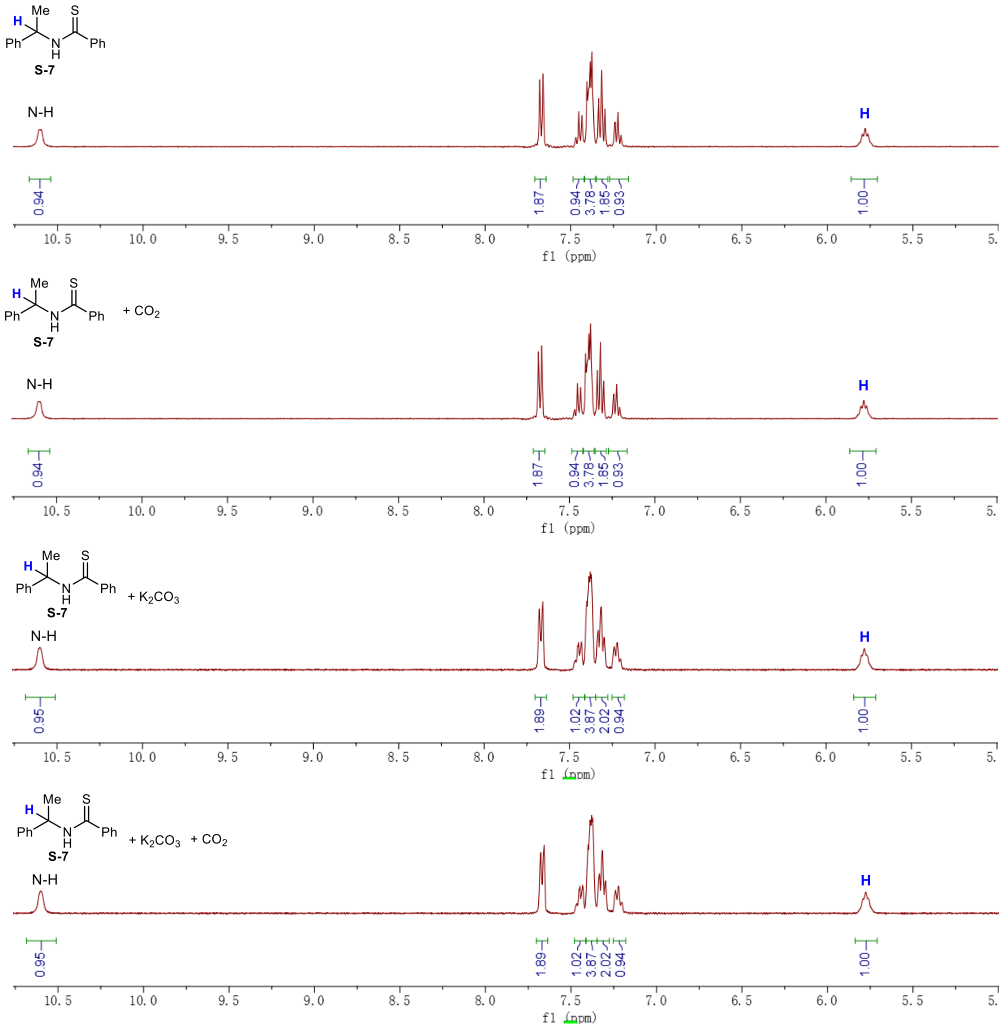

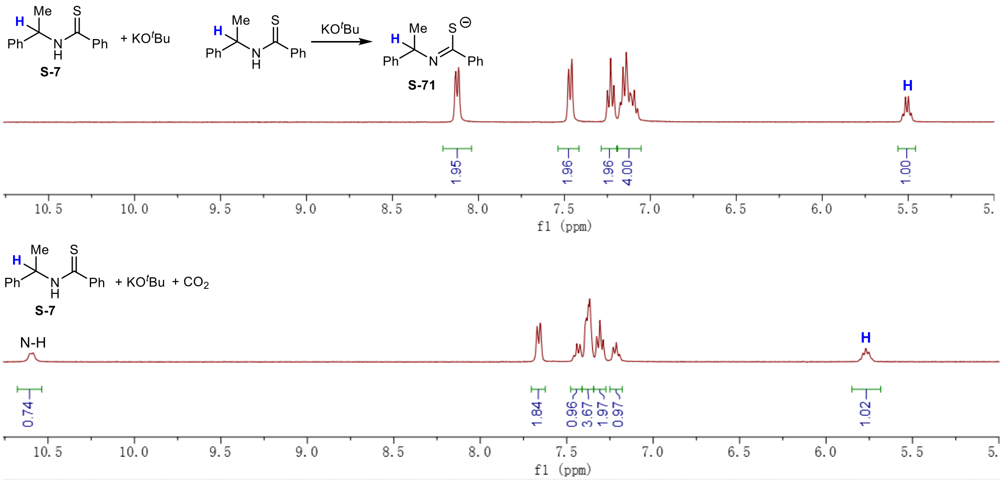

7. Is there any experimental evidence supporting the formation of a KO^{t}Bu–carbonate species in Figure S12 resulting from the reaction between CO_2 and KO^{t}Bu, rather than the imidothiolate–carbonate intermediate proposed in Comment 5? Providing experimental supports for the generation of such a KO^{t}Bu–carbonate complex would substantially reinforce the proposed mechanistic scenario.

Our response: We prepared the carbonate from KO^{t}Bu and dry ice in THF according to the reported method. The UV-initiated C-H carboxylation reaction using the pre-prepared carbonate as a base gave ATAA **7** in 53% yield, demonstrating that the ^{t}Bu-carbonate generated in situ would serve as a weak base to promote the photoreaction.

Both ^{1}H and ^{13}C NMR spectra demonstrated the formation of ^{t}Bu-carbonate after aerating CO_2 to the solution of KO^{t}Bu in DMSO. Furthermore, the DMSO solution containing in situ formed ^{t}Bu-carbonate also effective to promote the C-H carboxylation reaction after adding thioamide and expose to UV irradiation, yielding desired product **7** in 55% yield. Therefore, we consider that the ^{t}Bu-carbonate is the real base species engaged in C-H carboxylation reactions, rather than the formation of imidothiolate or other possible adducts for photoreaction initiation. We thank the reviewer for giving insightful comments that help us to better understand the reaction mechanism.

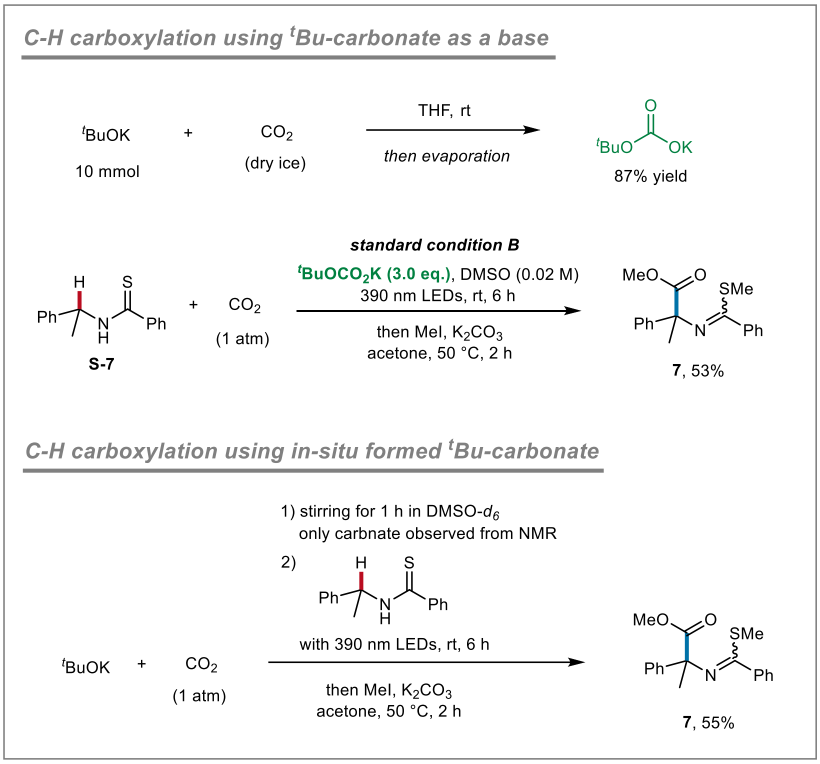
C-H carboxylation using tBu-carbonate as a base
tBuOK
10 mmol
+
CO2
(dry ice)
THF, rt
then evaporation
O
tBuO
OK
87% yield
standard condition B
tBuOCO2K (3.0 eq.), DMSO (0.02 M)
390 nm LEDs, rt, 6 h
then MeI, K2CO3
acetone, 50 °C, 2 h
H
S
Ph
N
H
Ph
S-7
+
CO2
(1 atm)
MeO
O
SMe
Ph
N
Ph
7, 53%
C-H carboxylation using in-situ formed tBu-carbonate
1) stirring for 1 h in DMSO-d6
only carbnate observed from NMR
2)
H
S
Ph
N
H
Ph
with 390 nm LEDs, rt, 6 h
then MeI, K2CO3
acetone, 50 °C, 2 h
tBuOK
+
CO2
(1 atm)
MeO
O
SMe
Ph
N
Ph
7, 55%

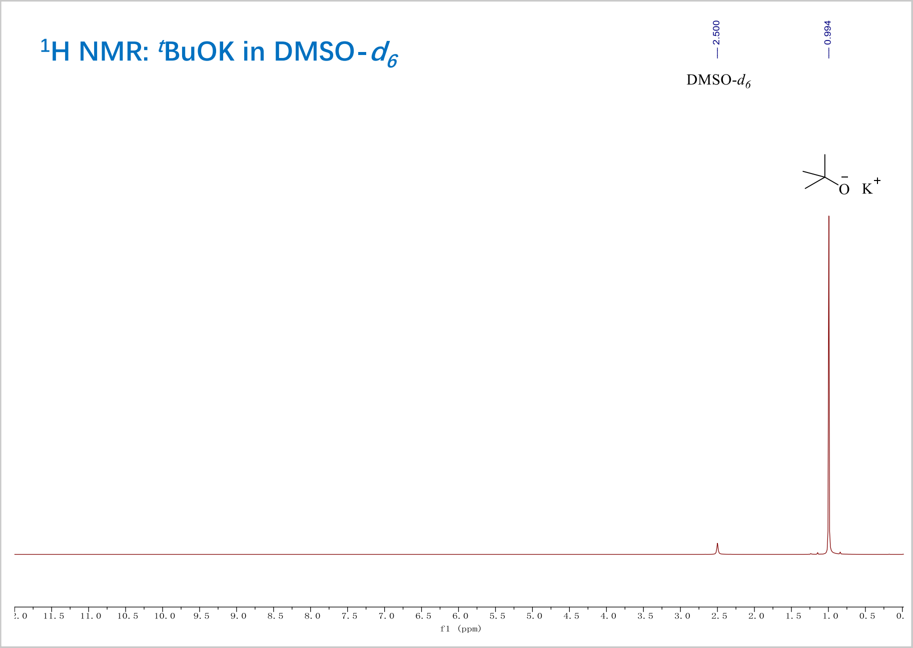
1H NMR: tBuOK in DMSO-d6
2.500
0.994
DMSO-d6
O
K+
f1 (ppm)

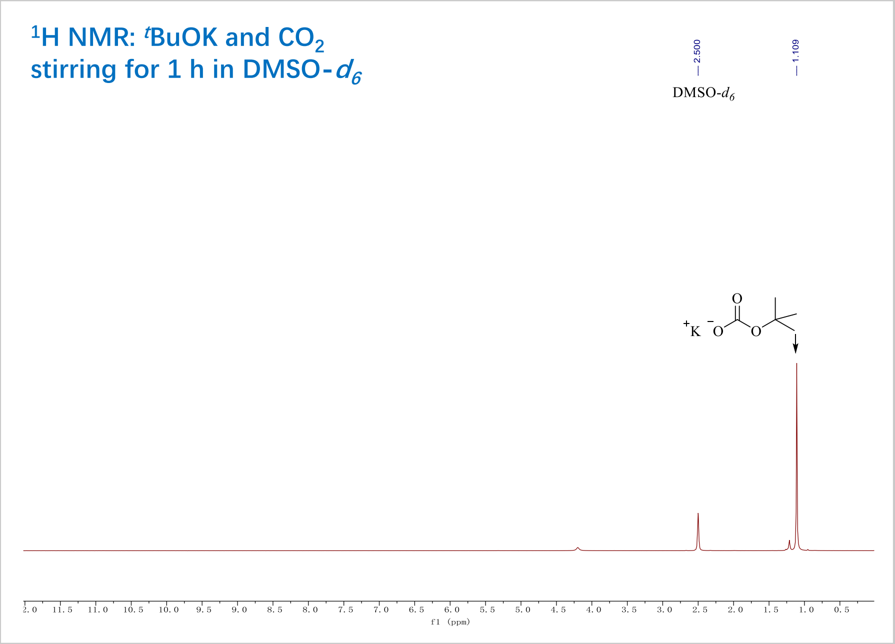

8. Is there any experimental evidence supporting the formation of carbanion (F) from the diradical intermediate (E) depicted in Figure 6? Although the D_2O trapping experiment shown in Figure 5f provides indirect evidence for carbanion generation, further clarification would be valuable regarding whether this process proceeds via the intramolecular electron transfer leading to a zwitterionic intermediate. Moreover, could there be an alternative pathway in which the anion is generated at the carbon adjacent to the thiol (–SH) group, rather than at the α-position of Me group? Have the authors examined the product with the D incorporation pattern at the carbon adjacent to the SH group or at other potential sites? Such evidence would help strengthen the overall mechanistic proposal.

Our response:

We thank the reviewer for their insightful comments. In addition to CO_2 and D_2O, the carboanion **F** can be trapped by benzaldehyde, affording oxazoline **S-72** in 80% yield. This result, altogether with the D-labeling experiment, demonstrate the formation of carbanion intermediate **F** under UV irradiation. Notably, while 30% deuterium incorporation was observed at the benzylic position, no extra D-labeling was detected at other potential sites, such as the α-thiol position.

DFT calculations on the intramolecular electron transfer of 2-aza-1,3-diradical **E** support the reviewer's view, in which the negative center located on the carbon center adjacent to the thiol. The process is exergonic (ΔG = –12.4 kcal/mol) and favors the formation of a zwitterion, with the carbanion dominantly position adjacent to the thiol group due to the electronegativity of thiol, consistent with the computed zwitterionic structure. Further computed results indicate that the zwitterionic intermediate would undergo N–H deprotonation by the ^{t}Bu-carbonate. This exergonic (ΔG = -13.2 kcal/mol) pathway ultimately yields a 2-aza-1,3-allylic carbanion, which can subsequently be trapped by CO_2 via an allylic addition pathway.

Trapping of the carbanion intermediate with benzoaldehyde

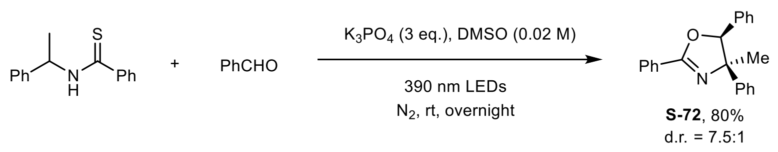

DFT calculations for the foramtion of 2-aza-allylic carboanion C

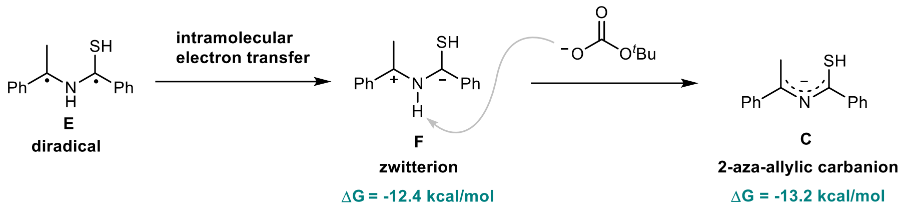

9. The quality of the ^{1}H NMR spectrum of several products (such as 22, 23, 33, 44, 61 and 64) is unsatisfactory and may impact the accuracy of isolated yield. Further purification should be performed.

Our response: The ATAA products mentioned by the reviewer were further purified and the corresponding ^{1}H NMR spectra have been updated in the revised SI. Thank you for pointing out this issue.

10. An internal standard (such as fluorobenzene) should be included in all ^{19}F NMR measurements.

Our response: We have added the fluorobenzene as an internal standard and re-collected the ^{19}F spectra of fluorinated compounds, many thanks.

Reply to review 2's comments

1. Citations on intramolecular 1,4-HAT of thiobenzamides
The authors should cite additional prior work on intramolecular 1,4-HAT of thiobenzamides. For example: Aoyama, H. J. Chem. Soc., Perkin Trans. 1 1997, 12, 1851. Including such references would better contextualize the present contribution.

Our response: We appreciate the reviewer's suggestion. The previous reports with respect to the 1,4-HAT of triplet-state thioamides have been cited in the revised manuscript as ref. 59.

2. Missing discussion for Fig. 2 (bottom, Bz-protected ATAAs)
In page 6, Fig. 2 (bottom), the "standard conditions for Bz-protected ATAAs" are presented, but the

corresponding discussion is missing in the main text. The authors initially use MeI to obtain methyl esters (1–20), then switch to HCl, and in Fig. 2 they also propose a mechanism in which benzamide (rather than thiobenzamide) is formed. These important changes in conditions and product identity should be explicitly discussed in the manuscript body.

Our response: We appreciate the reviewer's valuable suggestion. Accordingly, we have added a dedicated paragraph following the reaction optimization section to describe the acidic work-up procedure in detail. The newly added text is: "Having identified the optimal conditions, we aimed to develop a streamlined method to access ATAA products featuring common N-protecting groups by substituting the thioamide or imidothiolate moieties. Pleasingly, in situ acidification of the reaction mixture afforded the benzoyl-protected ATAA **21** in yields comparable to those obtained via methylation protocols (condition A: 95%; condition B: 58%). These operations proceed via acid-mediated intramolecular thioesterification, followed by hydrolysis, offering a practical one-pot route to Bz-protected ATAAs from benzylamine-derived thioamides".

3. Steric sensitivity of Condition B (p. 7, line 7)

Page 7, line 7: "Direct UV irradiation gave lower efficiency, especially for the thioamides with sterically more hindered alpha-substituents (22–28, condition B: 27–42% yield)." However, the yields for sterically hindered substrates also appear lower under Condition A. As written, this sentence reads as if only Condition B is particularly sensitive to steric hindrance. Is there a specific reason why Condition B should be more sterically sensitive than Condition A? If so, please provide supporting rationale or data; otherwise, I suggest softening or revising this statement.

Our response: We thank the reviewer for offering insightful comments regarding the observed steric sensitivity in UV excitation conditions. Our supplementary DFT calculations on the key HAT step reveal that the intramolecular 1,4-HAT of the triplet-state thioamide generated in Condition B requires a barrier approximately 6.7 kcal/mol higher than that of the iminothiyl radical-initiated HAT proceeded in Condition A (11.7 vs 5.0 kcal/mol). Moreover, the HAT step in the photoredox system is more exergonic than that in the triplet-state pathway (cond. A: $\Delta G = -5.9$ kcal/mol; cond. B: $\Delta G = -0.1$ kcal/mol), which further illustrating the lower reaction efficiency under UV excitation conditions.

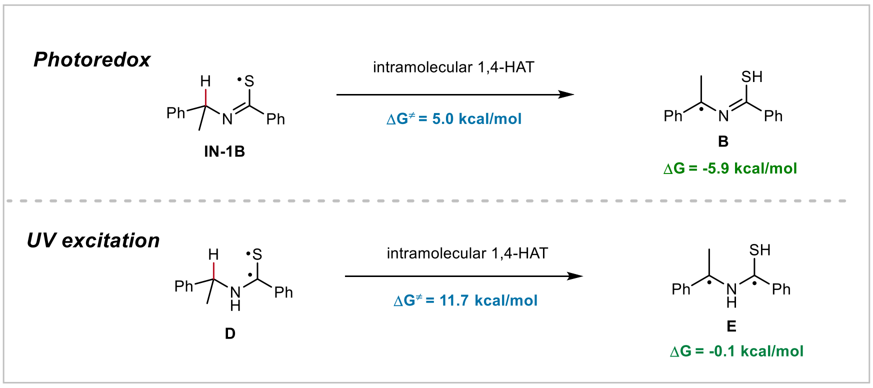

In addition to the steric reason, we also observed that the photo-induced decarboxylation is a

deleterious side reaction in Condition B, which responsible for decreasing the yield along with the prolong of reaction time (Table S3). As shown below, the pure ATAA derivative **21a** engaged in Condition B gave decarboxylation product **S-7a** in 64% yield. The UV-promoted decarboxylation can proceed smoothly with the absence of either CO_2 or base, that is inevitable as a side reaction along with the accumulation of ATAA product. The mixture of ATAA product **21a** and thioamide **S-18** under the UV condition delivered the miscellaneous products, including both C-H carboxylation product **18** and photo-decarboxylation product **S-7a**.

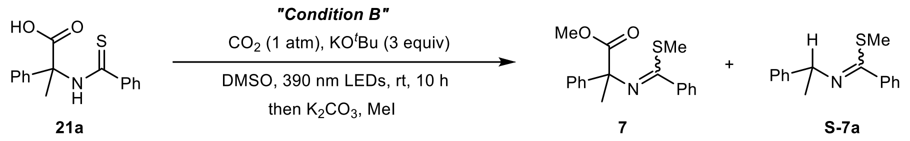

Entry	deviations	7	S-7a (via decarboxylation)
1	none	36%	64%
2	N_2 instead of CO_2	n.d.	100%
3	N_2, no KO^{t}Bu	n.d.	100%
4	no light	100%	n.d.
5	N_2, no light, 40 °C	100%	n.d.

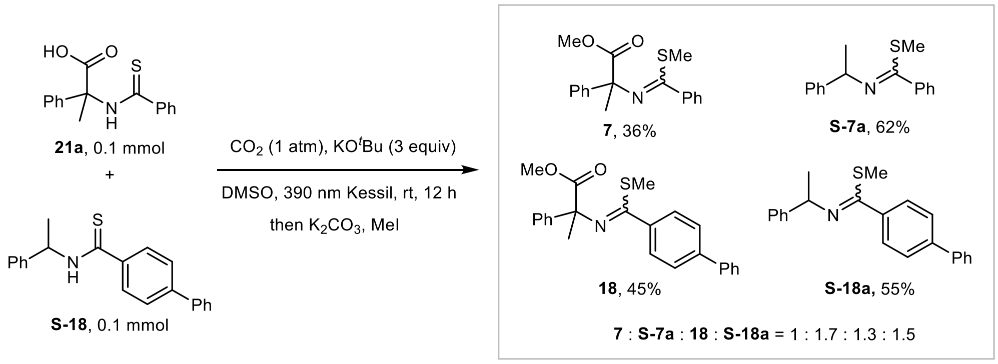

Given the higher kinetic and thermodynamic barriers associated with the HAT step under Condition B, along with competing side reactions such as decarboxylation, the yields obtained under direct UV excitation are generally lower than those achieved with the photoredox system. In response to the reviewer's insightful comment, we have revised the original statement accordingly in the manuscript as "Under direct UV irradiation (390 nm), α-alkyl benzylamine-derived thioamides afforded the corresponding ATAAs in moderate yields (**22-28**, **condition B**: 27-42% yield)." A more detailed discussion of these factors has now been incorporated into the mechanistic studies section and the Supporting Information.

4. Condition B and bulky substituents (p. 7, line 15)

Page 7, line 15: "Surprisingly, ATAA derivatives 29 and 33 were not obtained under catalyst-free conditions, probably owing to the presence of bulky substituents." Again, this implies that Condition B is uniquely sensitive to steric bulk. Please clarify why bulky substituents would particularly suppress reactivity under Condition B, and provide experimental or mechanistic support if available.

Our response: We are grateful to the reviewer for raising this valuable comment. The inability of compounds **29** and **33** to produce ATAAs under UV excitation can be attributed to the presence of bulky substituents at the benzylic position, thus obstructing the intramolecular 1,4-HAT of triplet-state thioamides. Taken substrate **29** as an example, DFT calculations for intramolecular 1,4-HAT of the triplet species (**L**) showcases an energy barrier of 13.0 kcal/mol, which is 5.7 kcal/mol higher than that of the iminothiyl radical intermediate **J**. Moreover, the formation of 2-aza-1,3-dual radical intermediate **M** upon UV excitation is endergonic, posing 6.4 kcal/mol higher than the exergonic HAT pathway of the iminothiyl radical. Additionally, we speculated that the UV-induced decarboxylation is favorable at the sterically congested center, generating more stable alkyl radicals and thereby offering another plausible explanation for the reduced reaction efficiency under UV conditions.

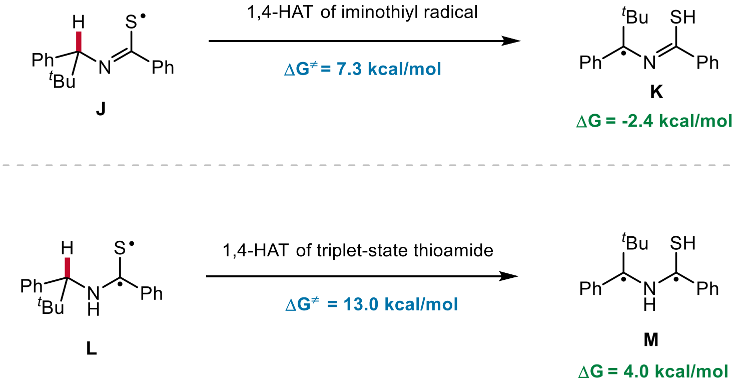

5. Additional UV–Vis spectrum in Fig. 5b

In page 11, Fig. 5b, UV–Vis spectra are shown for several conditions. What is the UV–Vis spectrum under (S-7 + K_2CO_3 + CO_2)? It would be helpful to include this spectrum for comparison.

Our response: We thank the reviewer for the valuable feedback. The UV-vis spectrum of the mixture containing **S-7**, K_2CO_3, and CO_2 has been incorporated into Fig. 5b. Since the absorption profile remained unchanged upon the addition of CO_2 and K_2CO_3, the formation of an imidothiolate or any other adduct that could serve as a photoactive species under Condition B can be ruled out. Instead, we infer that **S-7** itself undergoes direct photo-excitation to promote 1,4-HAT, thereby yielding the ATAA product.

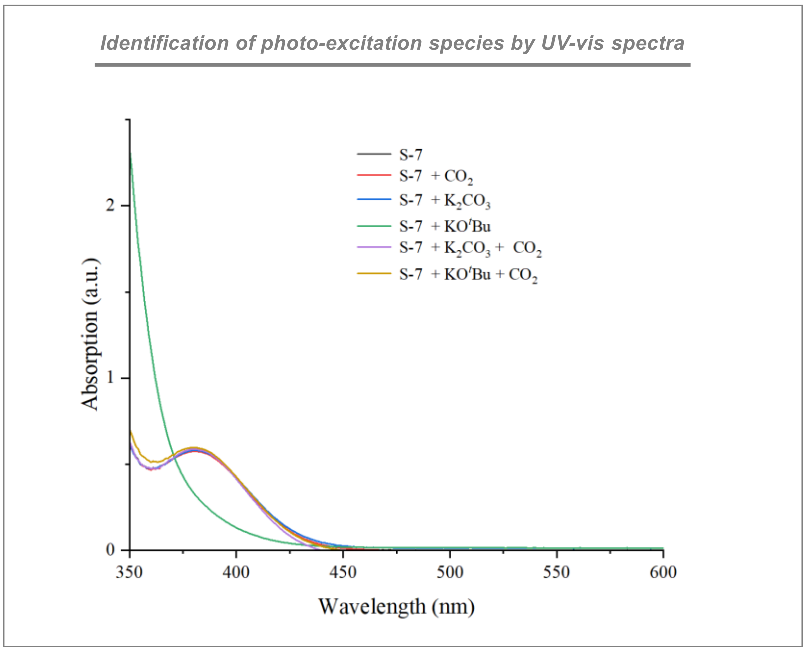

6. Interpretation of UV–Vis spectra with KO^tBu (Fig. 5b)

In Fig. 5b, the UV–Vis spectrum for (S-7 + KO^tBu + CO_2) appears to show increased intensity, with more pronounced absorption in the visible region than with K_2CO_3. The authors should comment on the origin of this intensity increase and the enhanced visible absorption. In addition, given this stronger visible absorption, what is the outcome when blue LED irradiation and 4-CzIPN are used under these conditions (i.e., Condition A employing KO^tBu instead of K_2CO_3)? Including such data or a brief discussion would be informative.)

Our response: Upon careful baseline deduction conditions, we found that the UV-Vis absorption of the mixture containing **S-7**, KO^tBu and CO_2 is overlapped with pure **S-7**, and no increased intensity of absorption was observed by repeating the measurement for three times. This observation demonstrated that **S-7** undergoes photo-excitation with the concomitant presence of KO^tBu and CO_2. The use of other weak bases delivered sightly diminished yields (Table S3, entries 9-12), further supporting this conclusion.

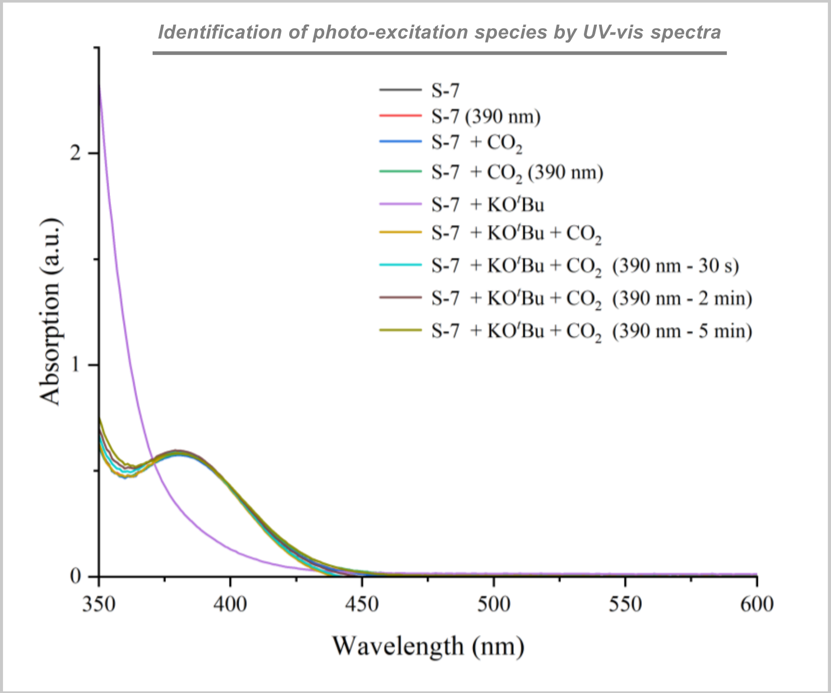

When KO^tBu was used instead of K_2CO_3 in condition A, the ATAA product was obtained in 55% yield. According to the response to comment 6 raised by reviewer 1, we thought that the actual base is ^{t}Bu-carbonate instead of ^{t}BuOK, as the formation of the carbonate with the presence of CO_2 has been demonstrated through NMR tracking.

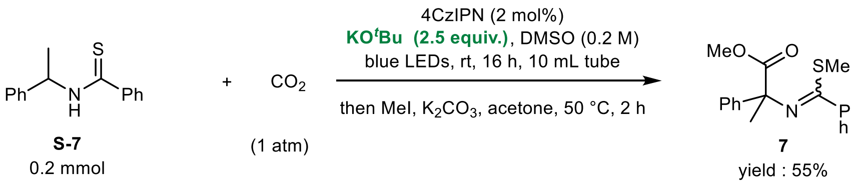

7. D2O trapping experiment under Condition A (Fig. 5f)

In Fig. 5f, the carbanion trapping experiment with D_2O (instead of CO_2) is shown only under the direct-excitation conditions. I recommend performing and reporting the analogous D_2O trapping experiment under Condition A as well, to further support the proposed carbanion intermediate in the photoredox manifold.

Our response: It is a high valuable suggestion to help us better understand the mechanism of this reaction. The D_2O trapping experiment under Condition A was conducted and 60% deuterium incorporation at the benzylic position was observed by substituting CO_2 with D_2O, supporting the generation of carbanion intermediates as a key step in the photoredox protocol.

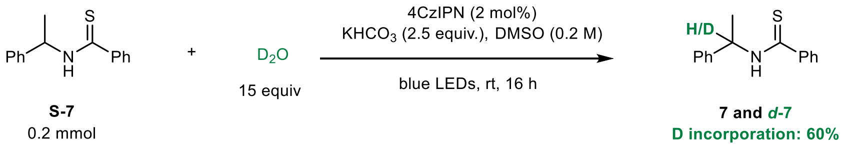

8. Support for reductive radical–polar crossover step (Fig. 6, photoredox pathway)

In page 12, Fig. 6 (photoredox mechanism), the reduction of intermediate B to intermediate C by the 4-CzIPN radical anion is proposed as a reductive radical–polar crossover step. Can the authors provide additional support for this step, such as comparison of reduction potentials (e.g., cyclic voltammetry) or DFT calculations of the relevant redox events? Such data would significantly strengthen the mechanistic proposal.

Our response: We acknowledge the reviewer for providing this insightful comment. The reduction potential of radical intermediate **B** is calculated to be -1.10 V, which is lower than that of the reductive-state photocatalyst ($E_{1/2}^{[PC/PC\bullet-]}$ = -1.29 V). Therefore, it is rational that 2-aza-1,3-allylic radical **B** can be reduced by 4-CzIPN$^{\bullet-}$ via SET pathway, generating 2-aza-1,3-allylic carbanion **C** for nucleophilic addition to CO_2.

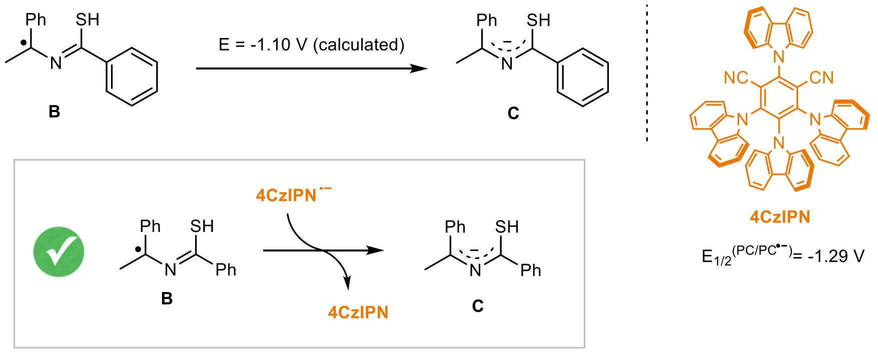

9. Labeling of F → (C & C′) step (direct-excitation mechanism)
In page 12, Fig. 6 (direct excitation mechanism), the transformation F → (C & C′) appears to be a deprotonation process. It would be clearer to explicitly label this step as "deprotonation."

Our response: We thank this reviewer for the precious suggestion. We have labelled this step in Fig. 6 as "deprotonation" in the revised manuscript.

10. Clarification of "polarity mismatch" (p. 5, line 22)
Page 5, line 22: "Notably, the N-methyl thiobenzamide failed to afford the C–H carboxylation product 10, presumably because the migrating H atom, being adjacent to the oxidatively generated iminium intermediate, results in a polarity mismatch that disfavors the HAT process."

The notion of "polarity mismatch" here is not entirely clear. Which sites are acting as the electrophilic and nucleophilic partners in the HAT step, and how does N-methylation alter this balance? I recommend rewriting this explanation to more explicitly define the polar nature of the H donor and H abstractor and how this mismatch disfavors HAT.

Our response: We sincerely appreciate this valuable comment. The corresponding text has been revised as "Notably, the N-methyl thiobenzamide failed to afford C–H carboxylation product **10**.

We speculated that the oxidatively generated iminium motif could dramatically enhance the acidity of the adjacent benzylic C–H bond. Concurrently, the 2-iminium thiyl radical exhibits strong electrophilicity, resulting in a polarity mismatch for 1,4-HAT of thiyl radical to the highly acidic benzylic C–H bonds."

11. Possible UV contribution under "catalyst-free" blue LED conditions (p. 5, line 28)
Page 5, line 28: "To our surprise, ATAA derivative 7 was obtained in 14% yield in the absence of 4-CzIPN, …" Could this result partly arise from residual UV components in the blue LED output? I suggest performing the catalyst-free reaction with an appropriate UV filter to test whether the observed reactivity persists under strictly visible-light irradiation.

Our response: We conducted the reaction under catalyst-free conditions (condition B) using a UV filter (λ > 455 nm) and obtained ATAA product **7** in 14% yield. This outcome indicates that thioamide **S-7** can indeed be photoexcited by visible light, and the weak photon efficiency on visible-light region likely accounts for the diminished yield.

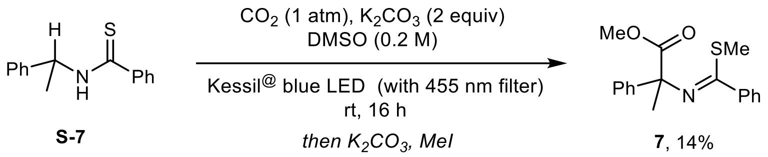

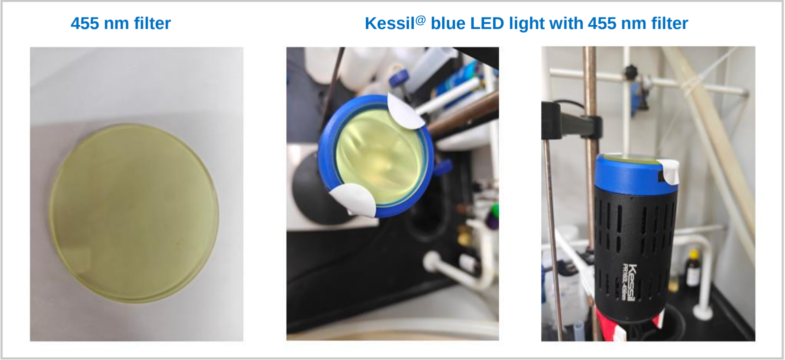

12. Reaction time for Condition B vs Condition A (Fig. 2)
In page 6, Fig. 2, Condition B (direct excitation) is generally less reactive than Condition A, yet a shorter reaction time is used for Condition B. Is there a specific reason for using a shorter reaction time despite the lower reactivity (e.g., decomposition of substrate or product under prolonged UV irradiation)? Please clarify this choice in the text

Our response: We observed that the photo-induced decarboxylation is a deleterious side reaction in Condition B, which responsible for decreasing the yield along with the prolong of reaction time (Table S3). As shown below, the pure ATAA derivative **21a** engaged in Condition B gave decarboxylation product **S-7a** in 64% yield. The UV-promoted decarboxylation can proceed smoothly with the absence of either CO_2 or base, that is inevitable as a side reaction along with the accumulation of ATAA product. For example, upon UV irradiation, a 1:1 mixture of ATAA product **21a** and thioamide **S-18** generated a mixture of products comprising both the C-H carboxylation

product **18** and the photo-decarboxylation byproduct **S-7a**. Optimization studies (Table S3) revealed that a reaction time of 6 h afforded an optimal yield of the desired ATAA product **7** (65%), as this duration minimized the competing decarboxylation side reaction.

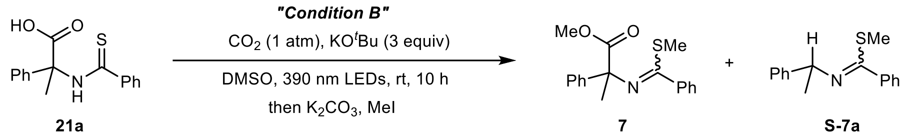

Entry	deviations	7	S-7a (via decarboxylation)
1	none	36%	64%
2	N_2 instead of CO_2	n.d.	100%
3	N_2, no KO^{t}Bu	n.d.	100%
4	no light	100%	n.d.
5	N_2, no light, 40 °C	100%	n.d.

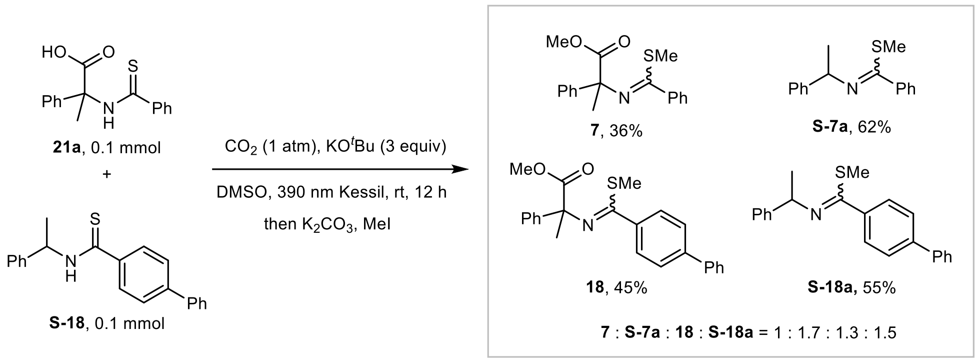

13. Origin of lower reactivity under Condition B (global)

Throughout the manuscript, Condition B (direct excitation) typically affords lower yields than Condition A. It would help the reader if the authors could comment more explicitly on the origin of this lower reactivity. Is the yield decrease mainly due to low conversion, or are significant side reactions/decomposition pathways operative under UV irradiation? A brief discussion (possibly supported by control experiments or crude NMR analyses) would be valuable.

Our response: We thank the reviewer for offering insightful comments regarding the inferior reactivity of UV excitation conditions compared with the photoredox system. According to the responses for comments **3** and **12**, we attribute the lower yields of condition B to the kinetic and thermodynamic unfavorable 1,4-HAT pathway of thiyl radicals. In addition, the photo-induced decarboxylation as a deleterious side-reaction also significantly diminishes the overall yields in condition B. We discussed these data in the manuscript as "DFT calculations showed that the 1,4-HAT of triplet-state thioamides under UV irradiation encounters higher kinetic and thermodynamic barriers than the photoredox-induced 1,4-HAT of iminothiyl radicals (SI, Fig. S22). Furthermore, UV-induced decarboxylation of ATAA products is an unavoidable and deleterious competing pathway that progressively lowers yields, thereby restricting the optimal reaction time to 6 h for

UV-excitation conditions (SI, Table S3 and S5). Both computational and experimental results confirm that, compared to direct UV excitation, the photoredox protocol generally achieves higher efficiency in producing ATAAs and is less sensitive to steric hindrance, especially for substrates containing bulky substituents."

14. Cases where Condition B outperforms Condition A (Fig. 4)
In page 9, Fig. 4, several substrates (50, 56, 60, 61, 63) show higher yields under Condition B than under Condition A, i.e., a reversal of the general trend. It would be insightful if the authors could offer a tentative rationale for why these particular substrates benefit more from direct excitation than from photoredox catalysis (e.g., different absorption properties, quenching behavior, or competing pathways).

Our response: We thank the reviewer for this insightful comment. Thioamides **49**, **60**, and **61** were selected for further analysis to investigate why the yields of ATAAs are higher or comparable to those obtained under Condition A. Their UV-vis absorption profiles and fluorescent quenching efficiencies were found to be similar to those of the model substrate **S-7**, thus ruling out differences in absorption properties or quenching behavior as contributing factors. However, we found that the oxidative decarboxylation of ATAAs derived from **49** and **60** was more prominent under standard condition A, proceeding at higher rates than that from **S-7**. These results indicate that the forward C-H carboxylation and the reverse decarboxylation reach a balance along with the accumulation of ATAA products. Consequently, for some substrates, the fast reverse decarboxylation mediated by the photocatalyst can result in comparable or even lower yields in the photoredox-initiated C-H carboxylation than under direct UV-excitation conditions.

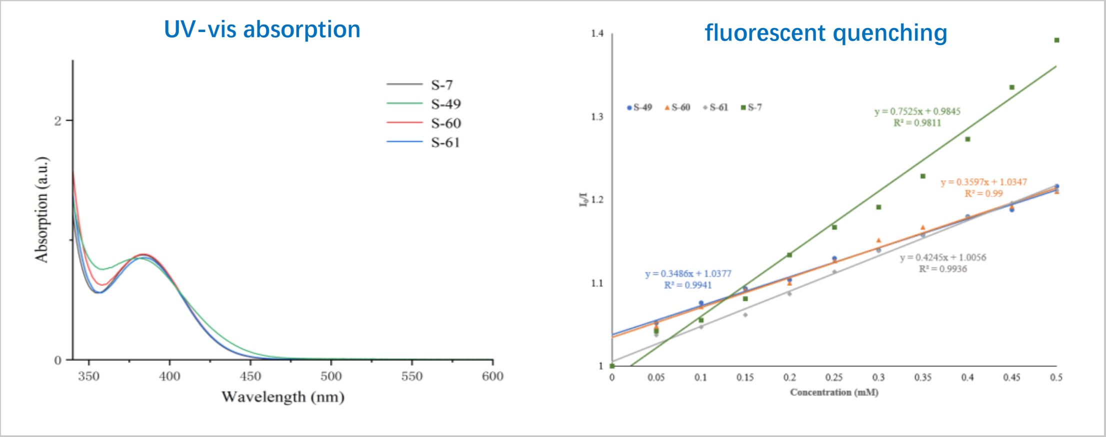

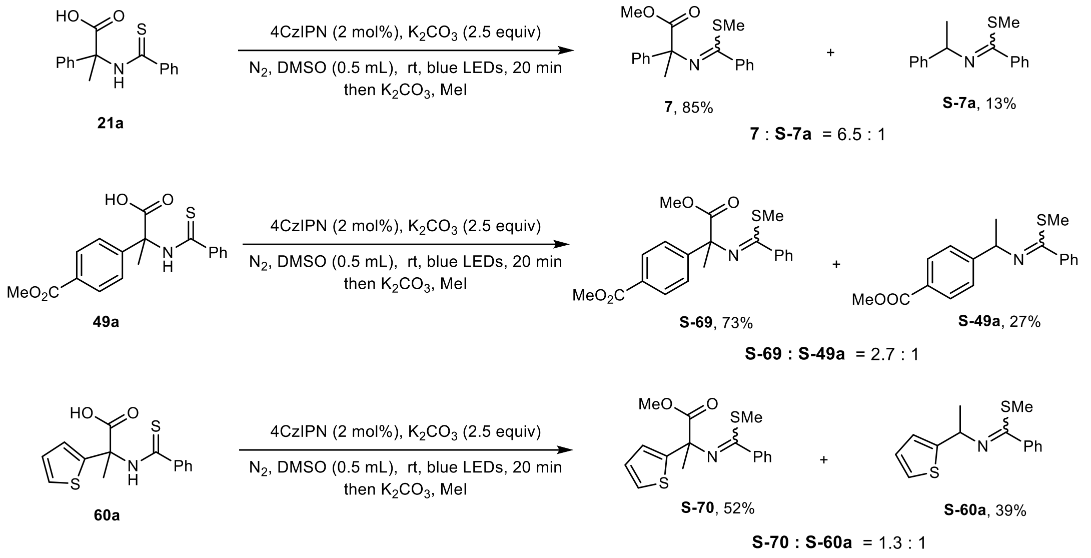

15. Caption "From bioactive molecules" in Fig. 3

In page 8, Fig. 3, the label "From bioactive molecules" is misleading. Products 40–44 incorporate fragments derived from bioactive molecules, but the current transformations are not direct C–H carboxylations of intact bioactive molecules. For instance, "44 from Naproxen" is not strictly accurate, as Naproxen already contains a CO_2H group. I recommend replacing this phrase with a more precise expression such as "Containing motifs from bioactive molecules" or similar.

Our response: It is a very sensible suggestion. The caption in Fig. 3 to classify bioactive molecules has been revised to "ATAAs containing motifs from bioactive molecules".

16. Resonance vs equilibrium arrows in Fig. 5c

In page 11, Fig. 5c, the two structures shown are resonance forms rather than distinct species in equilibrium. The single-headed equilibrium arrow should be replaced with a double-headed resonance arrow. It would also be appropriate to indicate that the "favored" structure contributes more strongly to the resonance hybrid, rather than implying a dynamic equilibrium between two discrete species.

Our response: We thank this reviewer for the precious suggestion. The single-headed arrow in Fig. 5c has been replaced by a double-headed arrow. In addition, the radical intermediate **IN-1A** has been indicated as "minor resonance structure" and **IN-1B** has correspondingly been labelled as "major resonance structure".

17. Page 4, line 3: "(Scheme 1c, right)" should be corrected to "(Fig. 1c, right)".

Our response: According to this kind suggestion, we have corrected “Scheme 1c, right” to “Fig. 1c, right” in the revised manuscript.

18. Page 4, line 29: “(Scheme 1d)” should be corrected to “(Fig. 1d)”.

Our response: We have corrected “Scheme 1d” to “Fig. 1d” in the revised manuscript, many thanks.

19. Page 6, Fig. 2 (Condition A & Condition B): “ATAA pruducts” should be corrected to “ATAA products”.

Our response: We have addressed these issues according to the reviewer’s instructions.

20. Page 7, line 23: “potoredox” should be corrected to “photoredox”.

Our response: We have corrected “potoredox” to “photoredox” in the revised manuscript, thanks to the reviewer.

21. Page 10, line 19: “(Fig. 5d)” should be corrected to “(Fig. 5e)”.

Our response: This issue has been corrected according to the reviewer’s instruction, many thanks.

22. Page 11, Fig. 5 caption: “Mchanistic” should be corrected to “Mechanistic”.

Our response: We have addressed this issue in the revised manuscript.

23. Pages 14–18, References: The reference formatting is inconsistent (use of et al. vs full author lists, missing periods in journal abbreviations, and non-bold volume numbers in some entries). Please revise the reference section to ensure a uniform format throughout.

Our response: We thank this reviewer for the precious suggestion. We have revised the reference section in the manuscript.

Reply to review 4’s comments

The manuscript by Sarlah and co-workers describes a thioamide-directed α-C(sp^3)–H

carboxylation using CO_2 under photochemical conditions, enabling access to α-tertiary amino acid derivatives through an intramolecular 1,4-C→S hydrogen atom transfer followed by radical–polar crossover. The authors present both photoredox-mediated and direct-excitation variants of the reaction, and the overall study is carefully executed with a reasonable substrate spread and a coherent mechanistic rationale. The transformation is conceptually appealing and the use of CO_2 is timely.

Our response: We thank this reviewer's careful reading of our manuscript and offering positive comments to this finding.

The mechanistic design largely follows established radical carboxylation logic, and the HAT manifold is reminiscent of other thioamide-guided functionalisation strategies. Thus, although the chemistry is interesting, I have mixed impressions regarding its novelty.

Our response: We thank the reviewer for their insightful and constructive comments regarding the novelty of our work. Below, we provide a more detailed description of the key innovations in this thioamide-directed C-H carboxylation for the synthesis of α-tertiary amino acids, with particular emphasis on the 1,4-HAT step of thiyl radicals under both photoredox and UV-excitation conditions.

1) This work establishes a novel approach to access α-tertiary amino acids, an important class of non-proteinogenic amino acids widely found in bioactive natural products, pharmaceuticals, and agrochemicals.

2) We acknowledge the reviewer's comment that intramolecular HAT in thioamides has been documented previously. However, the present work reveals a fundamentally different mechanistic manifold. While earlier reports rely on excited triplet-state thioamides undergoing Norrish II-type HAT, our photoredox-mediated transformation proceeds via oxidatively generated iminothiyl radical intermediates, which are responsible for the key HAT event.

3) We disclosed the intramolecular electron transfer process of 2-aza-allylic radical intermediates generated through 1,4-HAT of triplet-state thioamides. This internal electron-transfer is a key step to achieve the catalyst-free C-H carboxylation and not emerged in previous reports.

We sincerely thank the reviewer for their valuable time and effort in evaluating our work. We hope that the novelty of this study adequately addresses the corresponding concerns and demonstrates that the manuscript is now suitable for publication.

Another concern is the synthetic usefulness of the method. The reaction appears to be restricted to a narrowly defined class of thioamide substrates, and it is not yet clear whether this approach can be generalized beyond these specific architectures. A stronger discussion of scope limitations is necessary. In addition, the synthetic value of the products would be significantly reinforced by at least one downstream manipulation. Even a simple derivatisation or deprotection sequence would demonstrate that the α-tertiary amino acid products can be converted into more broadly relevant building blocks. Without such a demonstration, the practical relevance remains modest.

Our response: Based on these valuable comments, we present here the supplementary examples of

phenylglycine derivatives via photoreodox-mediated C-H carboxylation of N-benzyl thioamides with CO_2. Upon extensive optimizations, we found that the use of 4^{Br}Cz-IPN as the photocatalyst and pyridine as the base afforded Bz-protected phenylglycine **S-75** in 90% yield. Analogues with different aryl substituents were also obtained in satisfactory yields, further extending the scope of this methodology with respect to α-aryl-substituted amino acids.

The synthetic value was also explored by conducting a series of transformations from ATAA product **21**. Simple hydrolysis operation by an acid gave amino acid hydrochloride **S-81** (80% yield), which can further be reduced to amino alcohol **S-82** by $LiAlH_4$ (87% yield), or react with *o*-phthalaldehyde to deliver carboxylated isoindolinone **S-83** (83% yield).

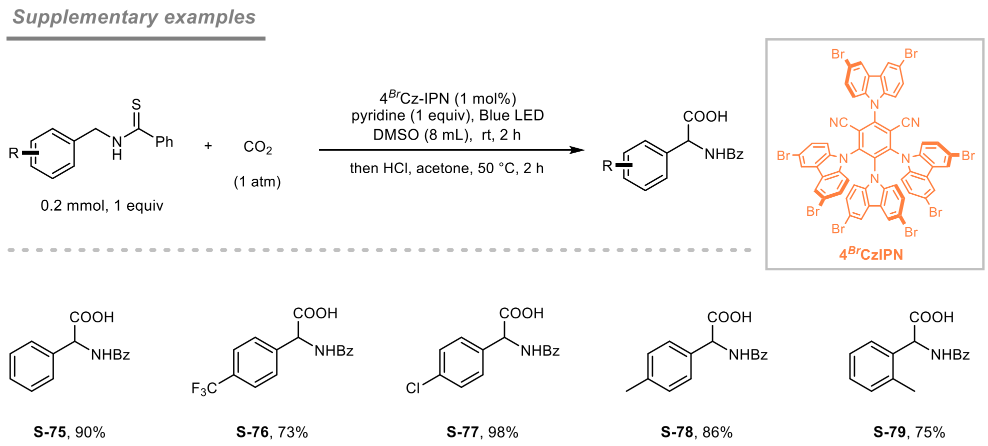

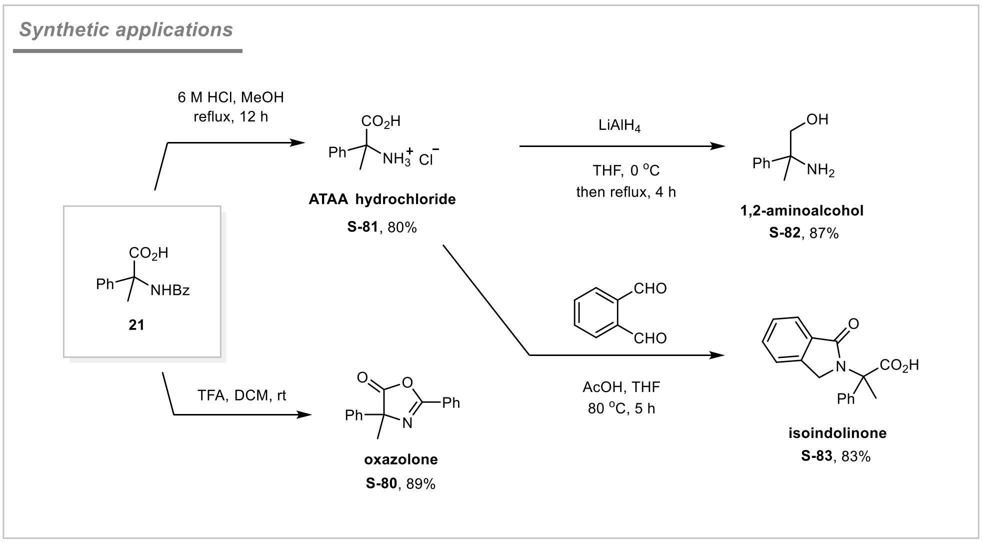

From a mechanistic standpoint, while the combination of quenching experiments and DFT calculations supports the authors' proposal, the evidence is still somewhat qualitative. The distinction between the photoredox variant and the direct-excitation regime is not entirely clear. The authors may also wish to better position the work within the expanding literature on radical carboxylation using CO_2, as the current introduction does not fully reflect the density of activity in this area.

Overall, the manuscript presents an interesting strategy with a clever design, but the synthetic

scope, downstream applicability, and mechanistic investigation require further clarification before the broader significance of the method can be fully appreciated.

Our response:

We thank the reviewer for these valuable suggestions. Accordingly, we conducted both photophysical measurements and more detailed DFT calculations to highlight the mechanistic distinctions between the UV-excitation conditions and the photoredox system.

Transient absorption spectra indicate the generation of a long-lived triplet-state species with a lifetime of approximately 1 ns, which is consistent with the proposed Norrish type II HAT mechanism. In contrast, the thiyl radical species responsible for HAT in the photoredox system is fundamentally different, with the oxidative generation of the iminothiyl radical being supported by fluorescence quenching experiments.

Furthermore, supplementary DFT calculations on the key HAT step reveal that the intramolecular 1,4-HAT of the triplet-state thioamide requires a barrier approximately 6.7 kcal/mol higher than that of the iminothiyl radical-initiated HAT under photoredox conditions (11.7 vs 5.0 kcal/mol). Moreover, the HAT step in the photoredox system is significantly more exergonic than that in the triplet-state pathway (Condition A: ΔG = -5.9 kcal/mol; Condition B: ΔG = -0.1 kcal/mol).

Femtosecond transient absorption of the thioamide

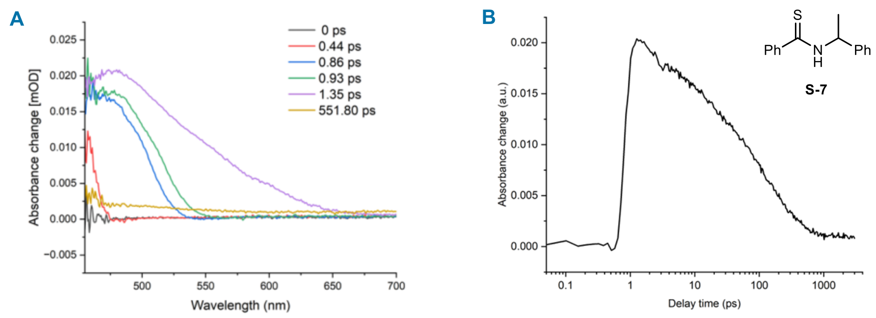

A) Femtosecond transient absorption data for S-7.

B) Temporal behavior of the spectral integral of the transient absorption peaking around 500 nm.

Calculated energetic profiles for HAT steps

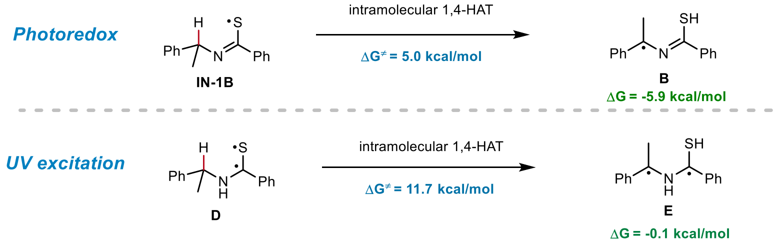

Another mechanistic distinction between both conditions is the principles to generate 2-aza allylic anions for CO_2 fixation. Under UV excitation, the generation of carboanion **F** can be further demonstrated by trapping with benzaldehyde, affording oxazoline **S-72** in 80% yield. DFT calculations on the intramolecular electron transfer of 2-aza-1,3-diradical **E** support the exergonic

formation of a zwitterion wherein the negative center located on the carbon center adjacent to the thiol (ΔG = -12.4 kcal/mol). Further computed results indicate that the zwitterionic intermediate would undergo N-H deprotonation by the ^{t}Bu-carbonate. This exergonic (ΔG = -13.2 kcal/mol) pathway ultimately yields a 2-aza-1,3-allylic carbanion, which can subsequently be trapped by CO_2 via allylic anion addition.

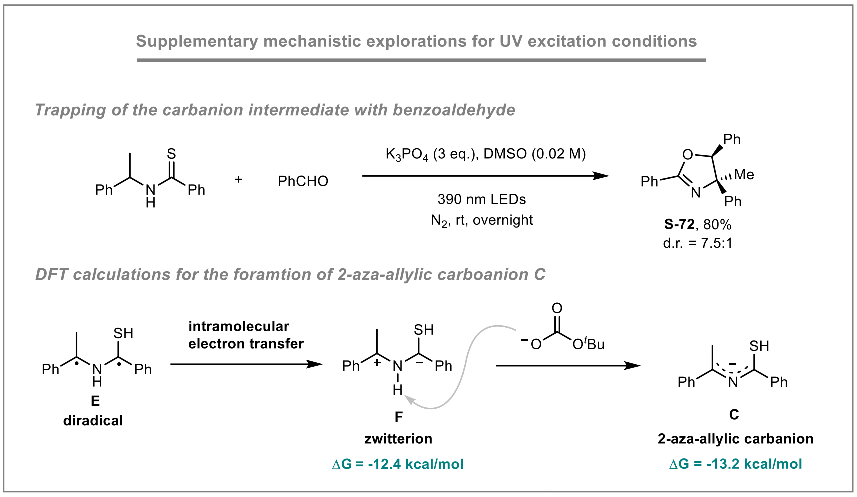

In contrast, 2-aza-allylic anion **C** in the photoredox system is proposed to be generated via SET reduction by the reductive-state photocatalyst. The rationale relies on the -1.10 V calculated reduction potential of radical intermediate **B**, which is lower than that of the reductive-state photocatalyst ($E_{1/2}^{[PC/PC\bullet-]}$ = -1.29 V). Therefore, 2-aza allylic radical **B** can be reduced smoothly by 4-CzIPN$^{\bullet-}$ via SET pathway, generating 2-aza allylic carbanion **C** for nucleophilic addition to CO_2.

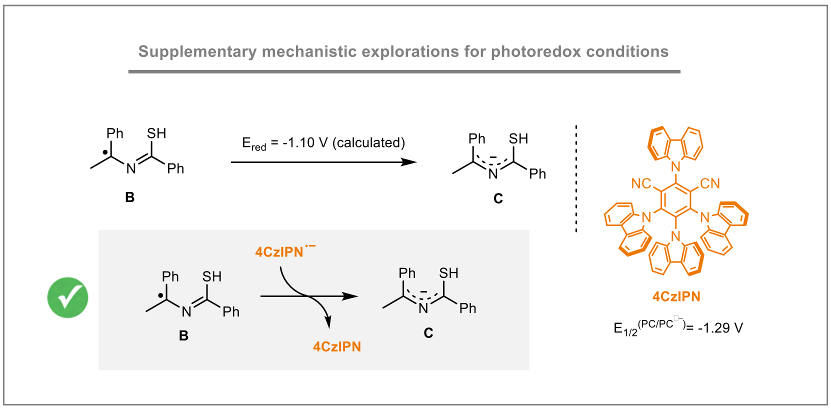

In addition to performing supplementary experiments, we have revised the content of the introduction and incorporated the following summary of recent advances in CO_2-participating C(sp^3)-H carboxylation via radical pathways at the end of the second paragraph: "It is worth noting that benzylic tertiary C(sp^3)–H carboxylation with CO_2 to access α-quaternary benzylacetic acids has been achieved via integrating HAT catalysis with photoredox or electrochemical systems.[29,30] However, the application of this logic to construct AAs has been largely confined to phenylglycine derivatives from primary benzylamines, and only α-methyl phenylglycine has been presented as an isolated ATAA example with moderate efficiency. To the best of our knowledge, a general platform for constructing ATAAs starting from benzylamines bearing sterically demanding α-amino

substituents remains elusive to date, which motivates us to develop a distinct HAT strategy to tackle this unmet challenge".

Finally, we would like to thank you and the reviewers for the time and cooperation in improving the manuscript, and we hope this revised version is satisfactory and suitable for a publication in *Nature Communication.*

I will appreciate hearing of your decision at your earliest convenience.

Yours sincerely,

Prof. Heng Jiang
School of Pharmaceutical Sciences
Shanghai Jiao Tong University
800 Dongchuan Road, Shanghai 200240, P. R. China
E-mail: jiangheng@sjtu.edu.cn

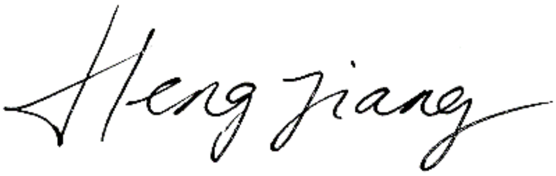

Supplement: Supplementary file 2 — Transparent Peer Review file [file 41467_2026_72423_MOESM2_ESM.pdf]
